# Mapping the Phosphoproteome of Influenza A and B Viruses by Mass Spectrometry

**DOI:** 10.1371/journal.ppat.1002993

**Published:** 2012-11-08

**Authors:** Edward C. Hutchinson, Eleanor M. Denham, Benjamin Thomas, David C. Trudgian, Svenja S. Hester, Gabriela Ridlova, Ashley York, Lauren Turrell, Ervin Fodor

**Affiliations:** Sir William Dunn School of Pathology, University of Oxford, Oxford, United Kingdom; Harvard Medical School, United States of America

## Abstract

Protein phosphorylation is a common post-translational modification in eukaryotic cells and has a wide range of functional effects. Here, we used mass spectrometry to search for phosphorylated residues in all the proteins of influenza A and B viruses – to the best of our knowledge, the first time such a comprehensive approach has been applied to a virus. We identified 36 novel phosphorylation sites, as well as confirming 3 previously-identified sites. N-terminal processing and ubiquitination of viral proteins was also detected. Phosphorylation was detected in the polymerase proteins (PB2, PB1 and PA), glycoproteins (HA and NA), nucleoprotein (NP), matrix protein (M1), ion channel (M2), non-structural protein (NS1) and nuclear export protein (NEP). Many of the phosphorylation sites detected were conserved between influenza virus genera, indicating the fundamental importance of phosphorylation for all influenza viruses. Their structural context indicates roles for phosphorylation in regulating viral entry and exit (HA and NA); nuclear localisation (PB2, M1, NP, NS1 and, through NP and NEP, of the viral RNA genome); and protein multimerisation (NS1 dimers, M2 tetramers and NP oligomers). Using reverse genetics we show that for NP of influenza A viruses phosphorylation sites in the N-terminal NLS are important for viral growth, whereas mutating sites in the C-terminus has little or no effect. Mutating phosphorylation sites in the oligomerisation domains of NP inhibits viral growth and in some cases transcription and replication of the viral RNA genome. However, constitutive phosphorylation of these sites is not optimal. Taken together, the conservation, structural context and functional significance of phosphorylation sites implies a key role for phosphorylation in influenza biology. By identifying phosphorylation sites throughout the proteomes of influenza A and B viruses we provide a framework for further study of phosphorylation events in the viral life cycle and suggest a range of potential antiviral targets.

## Author Summary

Eukaryotic cells regulate the function of many of their proteins through the reversible phosphorylation of serine, threonine or tyrosine residues. It is known that some influenza virus proteins are phosphorylated, but few sites of phosphorylation have been identified. We used mass spectrometry to identify 39 sites of phosphorylation, most of them novel, in proteins from influenza A viruses and an influenza B virus (a separate genus in the orthomyxovirus family) - to the best of our knowledge, this is the first time this has been attempted for all the proteins in a virus. By integrating sequence conservation data and structural information we were able to propose functions for most of these sites, providing a foundation for further studies, and we assessed experimentally the contribution of multiple phosphorylation sites in the influenza A virus nucleoprotein (NP) to viral growth and to transcription and replication of the genome. In addition, by identifying phosphorylation sites that are common to both influenza A and B viruses, we suggest that phosphorylation at these sites is a highly conserved aspect of influenza biology that may provide targets for antiviral therapy.

## Introduction

Influenza viruses cause serious and widespread disease in humans and livestock. Influenza A viruses can infect a wide range of birds and mammals, including humans [1]. Adaptation of novel influenza A viruses to humans appears to have caused pandemics for much of recorded history, including those of the devastating 1918 ‘Spanish’ influenza and the recent 2009 swine-origin influenza virus [2]. Established influenza A virus strains are responsible for seasonal influenza epidemics in humans, with additional cases of seasonal influenza caused by influenza B viruses, which have a much more restricted host range [3]. Humans are also infected by influenza C viruses, which typically only cause mild infections [4].

The proteins encoded by influenza viruses undergo a variety of post-translational modifications. In eukaryotic cells, phosphorylation of serine, threonine or, less frequently, tyrosine, is a common reversible protein modification that can have a wide range of effects on activity, stability, subcellular localisation and protein-protein interactions [5]. Phosphorylation can be readily detected using classical biochemical techniques, and a number of studies have identified phosphorylation of influenza virus proteins [6]–[23]. However, it is difficult to determine specific sites of phosphorylation using such techniques [24] and, to date, relatively few sites of influenza virus phosphorylation have been identified. In influenza A viruses phosphorylation has been found at T157 in the polymerase protein PA [25], T27 and S35 in the virulence factor PB1-F2 [16], S3 in the nucleoprotein (NP) [7], [13], S64, S82, S89, and S93 in the ion channel M2 (with S64 the major site of phosphorylation) [11] and S42, S48 and T215 in the non-structural protein NS1 [26], [27]. In addition, phosphorylation has been identified for S78 and S103 of influenza C virus M2, with S78 the major site of phosphorylation [28].

Here, we use liquid chromatography and tandem mass spectrometry (LC-MS/MS) to search for sites of phosphorylation in the proteomes of influenza A and B viruses. The same approach also allowed N-terminal processing to be identified, as well as a site of ubiquitination (in the influenza B virus M1 protein). To the best of our knowledge, this is the first time mass spectrometry has been used to simultaneously assess the phosphorylation of all proteins in a virus. In addition to three previously-identified sites, we report 22 novel sites of phosphorylation in influenza A viruses and14 novel sites of phosphorylation in influenza B viruses. Comparisons of experimental data and consensus sequences show that phosphorylation sites are conserved within and in some cases between genera. As the influenza A and B virus genera are estimated to have diverged several thousand years ago [29], [30], this conservation shows the fundamental importance of phosphorylation in influenza virus life cycles and identifies numerous potential targets for specific antiviral inhibition.

In the following sections we give an overview of the viruses and methodologies used in the study, give details of N-terminal processing of viral proteins, and then describe the location of phosphorylation sites in the matrix protein M1 and the ion channel M2, in the non-structural protein NS1 and the nuclear export protein NEP, in the viral glycoproteins, and in the polymerase and NP. By analysing the conservation and structural context of phosphorylation sites we propose that in influenza A and B viruses phosphorylation sites can affect viral entry and exit (HA and NA), regulate nuclear localisation (PB2, NP, M1, NEP, and possibly PB1 and NS1) and affect protein multimerisation (NP, M2 and NS1). Finally, we present experimental evidence that phosphorylation sites in influenza A virus NP contribute to viral growth in tissue culture and play a role at various points in the viral life cycle.

## Results/Discussion

### Overview of the Viruses and Methodologies Used in the Study

**Figure 1:**
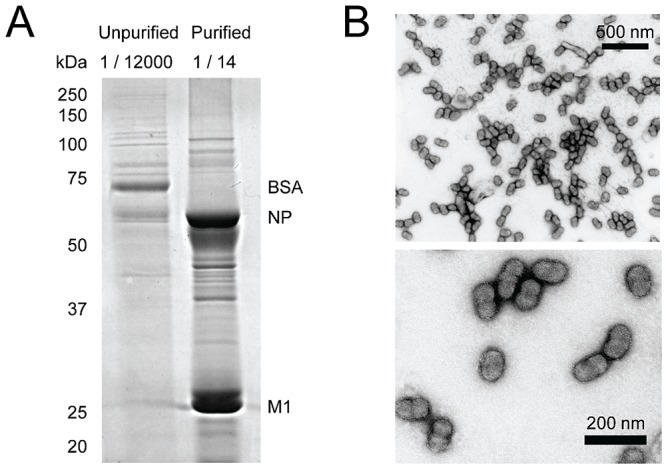
Purification of viral proteins. (A) Purification of WSN virus from the growth medium of infected MDBK cells. Samples of unpurified and purified material (0.01 ml from 120 ml and from 0.14 ml, respectively) were separated by 12% SDS-PAGE and stained with Coomassie Brilliant Blue. Key proteins are identified by electrophoretic mobility. (B) Negative-stain transmission electron micrographs of purified WSN virus. Two different magnifications are shown.

For influenza A viruses, we focussed on the well-studied H1N1 laboratory strain A/WSN/33 (WSN), using virions purified from the growth media of infected MDBK cells (Figure 1). In addition to the laboratory-adapted WSN virus, candidate vaccine viruses (CVVs) were considered. Influenza A CVVs were reassortants of the H1N1 laboratory strain influenza A/Puerto Rico/8/1934 (PR8) with clinical isolates of pandemic H1N1 and seasonal H3N2 viruses (Figure S1A; see Materials and Methods for details). CVV samples consisted of virions purified from embryonated chicken eggs and also (in one case) from the growth media of infected MDCK cells. For an influenza B virus, the directly egg-adapted CVV influenza B/Brisbane/60/2008 was used, purified from embryonated chicken eggs. Purification of viral proteins was assessed by PAGE and Coomassie or silver staining, which demonstrated the presence of highly concentrated viral proteins and the exclusion of the majority of cellular contaminants (Figures 1A, S1A). Purified influenza WSN virus was visualised by negative-staining and transmission electron microscopy, demonstrating intact virions of the expected morphology, with little contaminating material (Figure 1B). For purified virions, the entire protein content of the sample was processed without fractionation.

In addition to protein harvested from virions, proteins of the WSN virus were purified from lysates of human 293 T cells and from MDBK cells. In one approach tagged PB2 was used to purify material from infected cells (Figure S1B); all co-purifying proteins were analysed. In an alternative approach material co-purified with tagged proteins from transfected cells (Figure S1C) or unpurified lysates of infected cells (data not shown), were separated by PAGE, and bands were cut at the appropriate position to obtain the major viral proteins. Phosphorylation can alter electrophoretic mobility, and it is possible that cutting bands would cause modified proteins to be missed. In an attempt to counter this, Coomassie staining was used to identify the required proteins in the gel (data not shown).

Proteins were prepared for mass spectrometry by either excising them from polyacrylamide gels or by precipitation. Proteins were digested with trypsin to produce charged peptides, which were analysed by LC-MS/MS using the Central Proteomics Facilities Pipeline (CPFP) [31]. Localisation of phosphorylation sites was assessed using the Modification Localisation Score (ModLS) tool within CPFP, which is based on the PTMScore and AScore methods [32], [33]. To determine the most probable localisations for each phosphopeptide, ModLS scored all possible localisations using the mass-spectral evidence (see Materials and Methods for details). Detecting and identifying phosphorylated peptides by mass spectrometry has inherent difficulties [24], and initially we identified only a small number of sites. During the course of the investigation, the introduction of improved protocols and technology (notably, enrichment for phosphopeptides using TiO_2_ or IMAC resin, and the use of a more sensitive mass spectrometer, referred to in the text as ‘optimised methods’) greatly increased the number of phosphorylation sites detected. The phosphorylation sites found in influenza A and B viruses are summarised in Tables 1 and 2, respectively. Details of protein sequence coverage for all combinations of viruses and hosts are given in Table S1 and Figure S2, and details of phosphorylation sites found using different methods are given in Table S2. Representative fragment spectra for each modification identified are given in Figure S3. In all cases peptides containing the unmodified site were also identified.

**Table 1:** Summary of influenza A virus phosphorylation sites.

Protein	Putative phosphorylation site	Peptide Identification Probability^a^	Residue Conservation (%)	Kinase predictions^b^	Sample^c^	Ref
PB2	S742	1.000	99.9	PKA, PKB, RSK	S	
PB1	T223	0.985	99.8	N	T	
PA	S224/S225	0.998	99.5/69.2	N/N	S	
HA	T358	1.000	99.6	N	V	
NP	S9/Y10	1.000	99.1/99.99	CKI, PKA/INSR	V, S	
	S165	0.999	99.9	PKA	V, S	
	Y296/S297	1.000	99.9/99.98	P/Cdc2	S	
	S377/T378	0.798	58.6/99.95	CKII/N	S	
	S402/S403	0.999	99.9/0.1	PKC/PKA	V, E, S, T	
	S457	0.970	99.97	N	V	
	T472/S473	1.000	96.0/2.83	N/GSK3	V	
NA	S160/S164/S166	0.991	99.8/99.98/99.8	PKA, RSK/N/N	V	
M1	S2/T5	0.968	99.97/99.91	PKA/N	V, E	
	T9/Y10	1.000	98.5/98.5	N/N	V, E	
	T37	1.000	98.6	CKII, PKG	V, E	
	T108	0.978	99.97	CKI	V	
	T168/T169	0.981	93.1/99.9	P/N	V	
	S195/S196	1.000	99.9/99.8	CKI/N	V	
	S224/S225/S226	1.000	92.3/98.7/99.97	N/Cdc2/P	V	
M2	S64/T65	0.998	98.4/99.4	PKA/P	V, E	[11]
	S64 and T65	0.995	98.4/99.4	PKA/P	V	
NS1	S48	1.000	45.1	PKA	2	[27]
	T197	0.996	37.8	N	V	
	T215	1.000	27.1	Cdk5, GSK3, p38MAPK	V, 2, M	[26], [27]
NEP	S23/S24/S25	1.000	99.2/99.8/99.8	CKII/P/CKII	V	

NB: Residue conservation was calculated for H1-subtype HA and N1-subtype NA.

P = phosphorylation predicted by NetPhos 2.0, with no kinase predicted by NetPhosK 1.0.

N = no phosphorylation predicted.

a of clearest peptide spectral match.

b from WSN sequence.

c V: purified WSN virus, S: PB2-CStrep purification from an infection, T: TAP purification from a transfection, E: purified egg-grown CVV, 2: 293 T cell lysates, M: MDCK cell lysates.

**Table 2:** Summary of influenza B virus phosphorylation sites.

Protein	Putative phosphorylation site	Peptide Identification Probability^a^	Residue Conservation (%)	Kinase predictions
HA	S135/T136	0.913	100/98.2	PKA, PKC/PKC
	S465	1.000	100	ATM, CKI
NP	S50	0.999	100	GSK3, Cdk5
	T55/T56/S57/S58	0.999	99.6/99.4/79.7/88.8	Cdc2/CKII, PKG/P/CKI, CKII
	S223	1.000	100	N
	Y352/Y357/Y363	0.926	100/100/100	P/P/N
	S459/S463/S465	1.000	100/99.6/99.8	N/N/Cdc2, Cdk5, p38MAPK
	S486	1.000	98.9	PKA, RSK
M1	S2/T7/Y10	0.983	100/100/100	CKI, PKA/N/N
	S41	0.922	100	CKI, CKII
	S84/T88/T89/T91	0.862	100/100/100/100	Cdc2/PKC/Cdc2, PKC/PKC
	T188 + K194/K200 (Ub)	0.999	99.8+100/99.1	PKC
	S214/S218	0.996	100/99.8	PKC/ATM
	S236/S237	0.898	100/100	Cdc2/PKA

P = phosphorylation predicted by NetPhos 2.0, with no kinase predicted by NetPhosK 1.0.

N = no phosphorylation predicted.

a of clearest peptide spectral match.

When analysing WSN and B/Brisbane/60/2008 virions, we pooled data from multiple experiments, providing between 43% and 94% coverage of each protein detected, with each tryptic peptide detected an average of 18 times (Table S1). For WSN, the database of proteins searched included all known viral proteins, as well as the translations of hypothetical open reading frames. No peptides were found from PB1-F2, or the putative ambisense gene product NSP/NEG8 [34], [35], and no peptides were found matching the unique sequences of the PA-X [36] or PB1-N40 proteins [37]. Somewhat surprisingly, the non-structural protein NS1 was readily detected in all preparations of influenza A and B viruses. While attempts were made to achieve a high degree of viral purity (Figure 1) the current study cannot definitively distinguish structural proteins from carry-over of unincorporated proteins, and it is possible that NS1 was present in cellular debris that was co-purified with the virus. Consistent with this hypothesis, label-free quantitation (by SINQ [38] and iBAQ [39], data not shown) suggested that NS1 was found in the samples only at a low level, similar to that of many host proteins.

### N-terminal Acetylation and Methionine Excision

In addition to phosphorylation it was possible to detect N-terminal acetylation and methionine excision, which are both common post-translational modifications in eukaryotic cells [40]. While N-terminal acetylation has been linked to specific functions for a small number of proteins, its clearest general function is in preventing protein degradation [41]. As described in Tables 3 and S3, we detected N-terminal peptides from PB1, PA, NP, M1, M2, NS1 and NEP of influenza A virus and from PA, NP, M1, NS1 and NEP of influenza B virus, and in all cases N-terminal processing was detected. In most cases it is unclear whether N-terminal modifications would affect the function of these proteins, though structural studies suggest that N-terminal acetylation or methionine excision of PB1 should not affect its ability to interact with PA (data not shown; [42], [43]).

**Table 3:** Summary of N-terminal modifications.

Protein	None	Methionine excision	N-terminal acetylation	Methionine excision + N-terminal acetylation
PB1			A	A
PA			A+B	
NP				A+B
M1	A+B	A+B	A+B	A+B
M2		A		A
NS1			A	B
NEP		A	A	B

A: detected in influenza A virus.

B: detected in influenza B virus.

### Phosphorylation of the Viral Matrix Protein M1 and the Ion Channel M2

The matrix protein M1 of influenza A and B viruses is known to be phosphorylated at multiple sites, predominantly serines but also threonine [9], [10]. M1 is the most abundant protein in the virus (Figure 1A) [44], and the sequence coverage of M1 was the highest of any protein analysed (Table S1).

For influenza A viruses, even without enrichment for phosphopeptides we detected phosphorylation in the N-terminus of WSN, though it was unclear from the mass spectrum whether this was at T9 or Y10. Using optimised conditions, we again detected phosphorylation at this position, with phosphorylation of Y10 giving the best match to the observed mass spectrum (Tables 1, S2). We also detected phosphorylation at S2/T5, T108, T168/T169, S195/S196 (S195 has previously been noted to be in the S-x-E recognition motif of casein kinase [22], [45]), and S224/S225/S226 (with S226 matching the spectrum best; Table S2). In the PR8-derived M1 proteins of influenza A CVVs, even without using optimised conditions we once again detected phosphorylation at S2/T5 (or potentially at T9/Y10) and at S224/S225/S226, and we also detected phosphorylation at T37.

For the influenza B virus we observed a similar pattern of M1 phosphorylation. Without using optimised conditions we detected phosphorylation in the N-terminus, most likely at S2 or T7, though its localisation to Y10 cannot be ruled out (Tables 2, S2). With optimised conditions we detected this site again, with additional phosphorylations at residues S41, S84/T88/T89/T91, S214/S218, and S236/S237. We also detected phosphorylation at T188, on two peptides which also had di-glycine conjugated to the side-chain of either K194 or K200. Tryptic digestion of conjugated ubiquitin leaves a di-glycine tag on the ubiquitinated protein. This modification therefore provides evidence that M1 of influenza B virus can be ubiquitinated at either K194 or K200. M1 of influenza A virus was recently shown to be ubiquitinated [46]; this observation shows that influenza B virus M1 is also ubiquitinated and for the first time identifies a site of ubiquitination in an influenza M1 protein.

**Figure 2:**
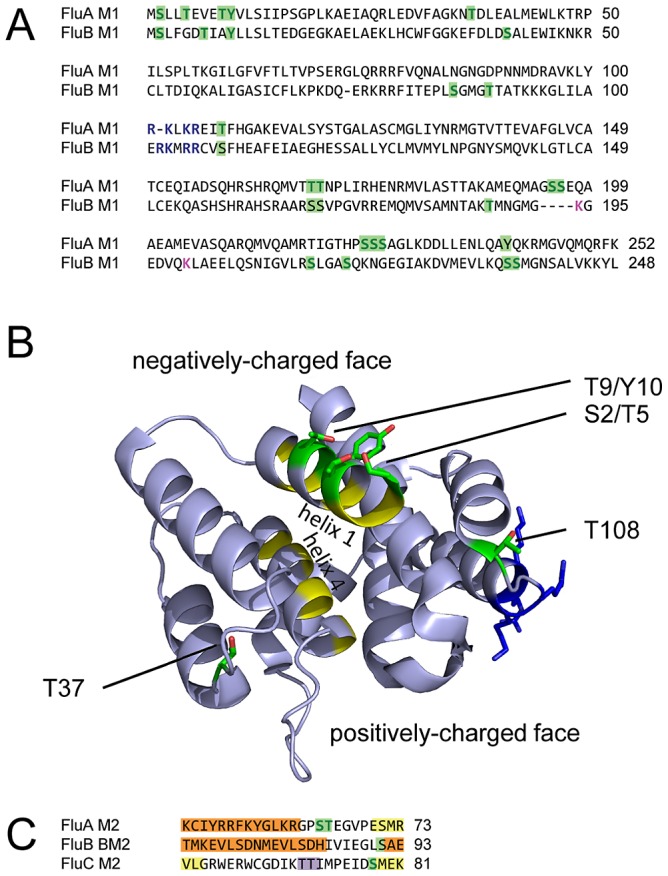
Location of phosphorylated residues in the matrix protein M1 and the ion channel M2. (A) The M1 consensus sequences of influenza A and B viruses, aligned using ClustalW2. Letters are coloured green for experimentally-confirmed phosphorylation sites, blue for the nuclear localisation signal (NLS) of influenza A virus and orthologous basic residues in influenza B virus, and purple for a ubiquitination site. Where modifications could be assigned to more than one residue, all probable residues are coloured (see text for details). Confirmed phosphorylation sites, and their possible orthologues, are highlighted in green. (B) Location of phosphorylated residues in the N-terminal portion of influenza A virus M1 (PDB 1EA3 [47]). Hydrophobic residues in helices 1 and 4 are coloured yellow and basic residues of the NLS dark blue. (C) Sections of the M2 consensus sequences of influenza A, B and C viruses. Colours are as (A), with additional highlighting of structural features: alpha helices are orange (where experimentally determined; PDB 2L0J [57] and PDB 2KJ1 [56]) or yellow (where predicted by JPred 3), and a beta sheet is purple (predicted by JPred 3).

Comparison of the sites of phosphorylation in M1 proteins of influenza A and B viruses indicates a number of common features (Figure 2A). In alignments of the primary sequences, we found four phosphorylation sites at similar positions in both genera. These are the sites in the N-terminal 10 amino acids, at T37 (A)/S41 (B) (both predicted to be targets of the kinase CKII; Tables 1, 2), at S195/S196 (A)/T188 (B) and at S224-S226 (A)/S214/S218 (B). In addition, three sites (T108 (A), T168/T169 (A) and S236/S237 (B)), are close to a serine, threonine or tyrosine in the other genus which could potentially be phosphorylated. Only one of the sites detected (S84-T91 (B)) does not correspond to a possible site of phosphorylation in the other genus. Thus, despite estimates that influenza A and B viruses have been evolving as separate genera for several thousand years [29], [30] their patterns of M1 phosphorylation appear to have been conserved.

The N-terminal domain of influenza A virus M1 has been crystallised and has a flattened shape, with its opposing faces being positively and negatively charged [47], [48]. The phosphorylation of S2, T5, T9 or Y10 would contribute to the net negative charge of one face of M1 (Figure 2B). It has been proposed that M1 may undergo a conformational change to bind to the inner leaflet of the plasma membrane, exposing hydrophobic residues in helix 1 and helix 4 [48]. S2, T5, T9 and Y10 are oriented away from the hydrophobic face of helix 1, and so their phosphorylation would not necessarily prevent lipid binding (Figure 2B). It is probable that these residues account for biochemical observations that a major site of M1 phosphorylation lies within or close to a stretch of hydrophobic residues [10]. A similar pattern of charged and hydrophobic residues, and of potential sites of phosphorylation, is conserved in the N-terminal M1 sequences of influenza A and B viruses (Figure 2A), though not in influenza C viruses (data not shown).

T37 and T108 both form part of another surface of the N-terminal domain, in this case in loops that pass between the positively and negatively charged faces (Figure 2B). T108 is close to the NLS of M1 (Figure 2B; [49]). An identical spacing can be seen in M1 of influenza B viruses between basic residues orthologous to this NLS (presumably the influenza B virus M1 NLS) and a conserved serine (S108; Figure 2A). This pattern is not seen in influenza C viruses (data not shown). Phosphorylation at or adjacent to NLSs is an important regulator of nuclear import, acting through a range of stimulatory and inhibitory mechanisms [50]–[52]. The conserved spacing of an NLS and a nearby phosphorylation site in influenza A and B viruses suggests a role for phosphorylation in regulating the nuclear import of M1, though whether phosphorylation in this context promotes or inhibits nuclear import remains to be determined.

The C-terminal domain of influenza A virus M1 has not been resolved by X-ray crystallography, but a combination of modelling and experimental studies suggest that it consists of alpha helices connected by loops [53]. Comparing N- and C-terminal secondary structures to the phosphorylation sites shows that, with the exception of phosphorylations in the N-terminal helix 1, phosphorylations throughout M1 take place on loops (data not shown).

The M2 protein forms a tetrameric ion channel in the viral envelope and is subject to a number of post-translational modifications, including disulphide bond formation, palmitoylation, fatty acylation, and phosphorylation [11], [54]. Previous studies have shown that for influenza A viruses the majority of M2 phosphorylation takes place at S64 [11], [21].

We clearly detected phosphorylation of either S64 or T65 in M2 of influenza A viruses even, in the case of the PR8-derived M2 of the CVVs, without using optimised conditions (Tables 1, S2). While some spectra favoured assignment of the phosphorylation to S64, others were ambiguous as to whether S64 or T65 was modified. Using optimised conditions, we detected a peptide in which S64 and T65 were simultaneously phosphorylated (Table 1). While consistent with previous data suggesting that the majority of M2 phosphorylation is of S64, this demonstrates that phosphorylation of T65 is also possible.

The M2 protein is translated from a spliced version of the mRNA encoding M1, with splicing taking place in codon nine of the M1 open reading frame. As the nine N-terminal residues common to M1 and M2 do not include a tryptic cleavage site, the N-termini of the two proteins could be clearly distinguished in this study. Despite being common to both proteins, residues S2/T5 and T9 are phosphorylated in M1 but not in M2. This is presumably a difference in phosphorylation, due to the different N-terminal structures of the two proteins. However, we cannot exclude the possibility that this difference is due to a chance failure to detect phosphorylated M2 peptides.

The M2 proteins of influenza A, B and C viruses are all phosphoproteins [11], [28], [55], but have little primary sequence homology ([56] and data not shown). By combining partial structures of the influenza A virus M2 and influenza B virus BM2 cytoplasmic domains [56], [57] with structures predicted from the primary sequence of influenza A and C viruses, we found that in all three genera the cytoplasmic tail contains a loop between two alpha helices, within which is a conserved S-x-E casein kinase recognition motif (Figure 2C; [45]). The phosphorylation prediction methods NetPhos 2.0 and NetPhosK 1.0 [58] predict phosphorylation for all three serines – S64 in influenza A viruses, S91 in influenza B viruses and S78 in influenza C viruses. Phosphorylation of influenza BM2 was not detected in this study, but S64 and S78 are known to be the primary sites of M2 phosphorylation in influenza A and C viruses, respectively [11], [28]. Thus, phosphorylation of a cytoplasmic loop in M2 appears to be a general feature of influenza viruses.

Mutating the cytoplasmic loop serine of influenza A and C viruses reduces the ability of M2 dimers to assemble into tetramers [21], [28]. In influenza A viruses this does not appear to inhibit viral replication in cell culture or in experimentally infected mice [21], but these systems are more permissive to viral growth than natural infections [21], and although the mutation prevented the majority of M2 phosphorylation it is possible that low levels of T65 phosphorylation may have reduced any phenotype further. It is, therefore, plausible that phosphorylation of a loop in the cytoplasmic tail of M2 promotes tetramer formation in influenza A, B and C viruses.

### Phosphorylation of the Non-Structural Protein NS1 and the Nuclear Export Protein

The non-structural protein NS1 is known to be phosphorylated, predominantly on threonine [9], [17], [18], [26], [27], though the pattern of phosphorylation may vary between strains [59]. In this study, we detected three sites of phosphorylation in WSN NS1. Phosphorylation of S48 was detected in the lysates of 293 T cells, phosphorylation of T197 in preparations of WSN virus, and phosphorylation of T215 in lysates of 293 T and MDBK cells, as well as (with a weaker spectrum) in preparations of WSN virus (Tables 1, S2).

**Figure 3:**
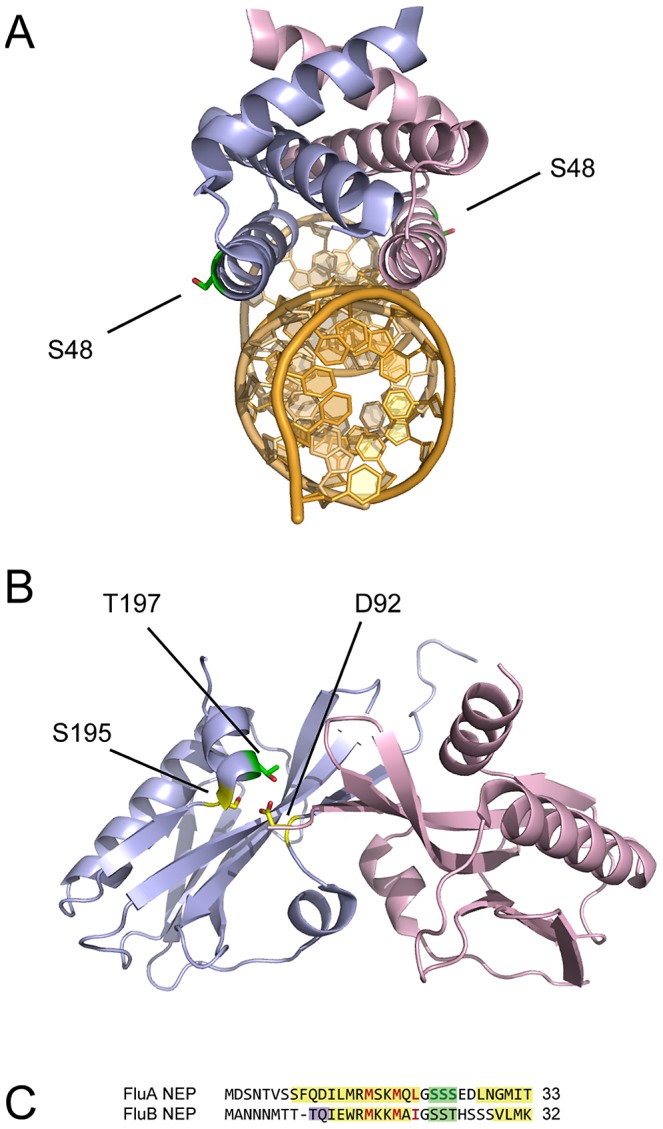
Location of phosphorylated residues in the non-structural protein NS1 and the nuclear export protein NEP. (A) Location of S48 in the dimeric NS1 RNA binding domain (PDB 2ZKO [60]). The subunits of the dimeric NS1 RNA binding domain are shown in light blue and pink, and RNA in gold. (B) Location of T197 and interacting residues in the dimeric NS1 effector domain (PDB 2GX9 [61]). (C) Consensus sequences of the N-terminal residues of influenza A and B virus NEP. Colours are as in Figure 2, with key hydrophobic residues of the nuclear export signal (NES) in red.

A recently published report noted phosphorylation of S48 [27], observing that although it is part of the RNA-binding domain of NS1, it is positioned so that it does not participate in RNA binding (Figure 3A, [60]). Mutational analysis suggested that phosphorylation at S48 does not affect virus replication in tissue culture; consistent with this, the residue is asparagine in a number of human isolates [27]. T197 is part of the NS1 effector domain (Figure 3B). With the nearby S195, it makes strong hydrogen-bonding interactions with D92, a virulence determinant in H5N1 strains of the virus, and it is adjacent to the dimer interface of NS1 effector domains [61]. It has been proposed that phosphorylation of either S195 or T197 may destabilise NS1, potentially disrupting its dimerisation [61], and may regulate its nuclear localisation [62]. T215 is in the disordered C-terminal tail of NS1 [62], and is adjacent to a second NLS in some strains of the virus – though not in WSN [63]. Phosphorylation of T215 has previously been detected, but although the residue is important for viral growth, mutational analysis suggests that its phosphorylation is not required in tissue culture [26], [27].

The nuclear export protein (NEP) of influenza A and B viruses is involved in the nuclear export of the viral genome in the form of ribonucleoprotein complexes (RNPs) [64]–[66] and despite initial descriptions of it as a second non-structural protein (NS2) it has been shown to be incorporated into virions [19], [67], [68]. The NEP of influenza A virus is known to be phosphorylated [19]. Both with and without optimised conditions, we detected phosphorylation in the NEP of WSN at S23, S24 or S25 (Table 1). In the clearest spectra S24 is unambiguously phosphorylated but in others the localisation is less distinct, and phosphorylation of S23 or S25 in a proportion of cases cannot be excluded.

The NEP phosphorylation site is adjacent to a previously identified nuclear export signal (NES) [64], [65] (Figure 3C), and is predicted to lie on a loop between an N-terminal alpha helix containing the NES and another alpha helix. The same arrangement of three serines or threonines with respect to the NES and to predicted alpha helices is found in NEP of influenza B viruses (Figure 3C; phosphorylated peptides were not detected from influenza B virus NEP in this study), though not in influenza C viruses where the NES is located in a different region of the protein [66].

As with NLSs, phosphorylation in or near to NESs can promote or inhibit nuclear export through a range of mechanisms [51], [52]. The conserved spacing of the NES and phosphorylation site in influenza A and B viruses (Figure 3C) suggests that phosphorylation regulates the interaction of NEP with its nuclear export factor Crm1 [64]. Blocking activation of the MAP kinase ERK has been shown to prevent the nuclear export of NEP, indicating that phosphorylation has a stimulatory effect on the export of NEP, and thereby of RNPs [69]. A possible mechanism for this is suggested by the cellular MK2 protein, which also requires phosphorylation for Crm1-mediated export. In MK2 the NES is part of an autoinhibitory alpha helix that interacts with an adjacent domain of the molecule. Phosphorylation of a hinge region induces a conformational change, reducing the interaction between the alpha helix and the rest of the molecule and unmasking the NES [70], [71]. The position of the NEP phosphorylation site on a loop between the NES and an adjacent alpha helix suggests that phosphorylation may unmask the NES in a similar fashion. The nuclear export of RNPs necessarily precedes viral assembly, and consistent with this phosphorylated NEP was readily detected in virions of WSN (Table 1).

### Phosphorylation of the Viral Glycoproteins

The haemagglutinin (HA) and neuraminidase (NA) proteins of influenza viruses are known to be subject to post-translational modification, notably glycosylation [72]–[74], but we were not aware of reported phosphorylation of these proteins. Indeed, we found comparatively few sites of phosphorylation in the glycoproteins, with modifications only detected when optimised conditions for phosphopeptide detection were used (Table S2).

**Figure 4:**
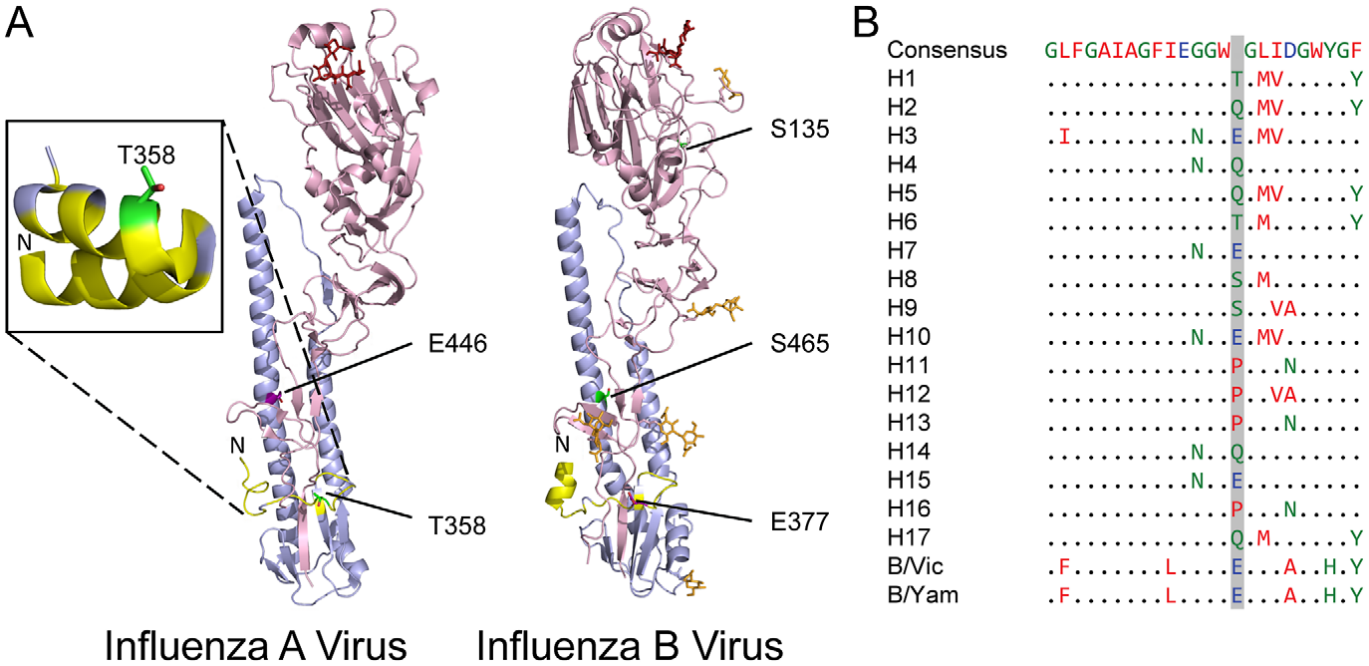
Location of phosphorylated residues in haemagglutinin. (A) An influenza A virus H1-subtype HA monomer (PDB 1RVZ [75]) and an influenza B virus HA monomer (PDB 2RFU [81]). In the influenza A virus HA, T358 is indicated in the N terminus of HA2 and, after conformational rearrangement, in the fusion peptide (inset; PDB 2KXA [76]); the corresponding residue E377 is indicated in influenza B virus HA2. In influenza B virus HA S135 in indicated in the head domain (in the structure shown, position 136 is alanine), and S465 in the stem; the corresponding E446 residue is indicated in influenza A virus HA2. HA1 is shaded pink, HA2 light blue, hydrophobic residues of the fusion peptide yellow, glycosylations of the influenza B HA orange, the α(2,6)-sialic acid-containing pentasaccharide LSTc red and glutamic acids orthologous to phosphorylation sites purple. (B) The overall consensus sequence of the HA fusion peptide, compared to the consensus sequences of individual subtypes and lineages. Residue 15 of the fusion peptide, which corresponds to T358 of the H1 sequence, is shaded.

For HA of the influenza A virus WSN (H1 subtype), we detected phosphorylation of T358. After cleavage of HA0, T358 forms residue 15 of the fusion peptide of the HA2 fragment, which inserts into the endosomal membrane to allow viral fusion (Figure 4A) [72], [75]. T358 is oriented away from the majority of the hydrophobic residues in the fusion peptide, and is expected to remain exposed to solvent during fusion rather than being buried in the membrane [76]. As a result, the presence of a negatively-charged phosphate at this position should not interfere with the fusion process (Figure 4A, inset).

Residues in the fusion peptide, including T358 (Table 1), are highly conserved among H1 haemagglutinins. When consensus sequences of the fusion peptides of different HA subtypes from influenza A and B viruses are compared, they conform to an overall consensus sequence, with a small number of biochemically conservative changes (Figure 4B). We found that the only position not to conform to a clear overall consensus is position 15. This can take the form of the potentially-phosphorylated, small polar residues serine and threonine (H1, H6, H8 and H9 subtypes); of glutamic acid, which has similar physicochemical properties to phosphoserine or phosphothreonine (H3, H7, H10 and H15 subtypes, as well as both influenza B virus lineages); of glutamine, which is a similar size to glutamic acid but polar rather than charged (H2, H4, H5, H14 and H17 subtypes); or of proline, a small secondary amine which, unlike the other possibilities, is hydrophobic (H11, H12, H13 and H16 subtypes). This diversity between subtypes suggests that, unlike the rest of the fusion peptide, position 15 can tolerate a range of physicochemical properties. In support of this, an E15V mutation in the H3 fusion peptide does not affect the fusogenic properties of HA [77]. It is therefore likely that phosphorylation of T358 in H1 subtypes, as detected here, would be compatible with HA function. Within subtypes, however, position 15 is highly conserved, suggesting that each subtype has an optimal amino acid.

Prior to fusion, HA is maintained in a metastable conformation by hydrogen bonding between the fusion peptide and a pocket formed from residues in both the HA1 and HA2 fragments. Interactions between the fusion peptide and the pocket are subtype-dependent, and disrupting these interactions by mutation has been shown to regulate the pH at which HA is activated [78], [79]. A recent study showed that a threonine to isoleucine mutation proximal to the fusion peptide was an important determinant of the pH of HA activation and, consequently, of the respiratory droplet transmissibility of an H5 HA/H1N1 reassortant virus in ferrets [80]. The presence of charged, polar or hydrophobic amino acids at position 15, as shown here, would be expected modulate the pH at which activation occurs for a given HA subtype. If this is the case, phosphorylation of position 15 (possible for the H1, H6, H8 and H9 subtypes) could provide an additional mechanism for fine-tuning the activation of HA.

In influenza B viruses position 15 of the fusion peptide is glutamic acid (E377), and hence cannot be phosphorylated (Figure 4A, B). However, two additional phosphorylation sites were found. We detected phosphorylation in the HA1 fragment, at one of two conserved residues, S135 or T136, and also in the HA2 fragment, at the conserved residue S465. The S135/T136 site, which has no obvious ortholog in the influenza A virus HA structure (Figure 4A), is surface-exposed on a loop in the head domain of HA, away from the interface of the trimer subunits [81]. It is not part of the receptor binding site of HA, and so would not be expected to interfere directly with sialic acid binding. It has, however, been noted that in influenza A viruses the net charge of the HA1 affects the ability of the virus to interact non-specifically with negatively-charged cell surfaces [82], [83]. The virus is known to require a balance of HA and NA functional activity, and charged amino acid substitutions reducing the binding affinity of HA have been shown to compensate for mismatched NA activities [84]. Phosphorylation of the S135/T136 site would be expected to reduce the avidity of HA for cell surfaces. As only a proportion of the viral HA is phosphorylated (unmodified peptides from the same region were also detected) this suggests a novel mechanism, of more subtle effect than the substitution of charged residues, for achieving optimal HA activity.

Phosphorylation of S465 is harder to explain, as it is internal to the HA trimer and unlikely to be accessible to kinases (Figure 4A). S465 is in one of the short regions of primary sequence similarity between influenza A and B virus haemagglutinins, and it is orthologous to a glutamic acid present in all influenza A subtypes (E446 in H1 subtypes; data not shown). At the pH of fusion influenza A virus HA undergoes a drastic conformational change, exposing this glutamic acid as the HA2 stem folds back on itself [85]. Assuming HA of influenza B virus undergoes a similar conformational change, S465 would be exposed to kinases in the fusion conformation; indeed, it is present in a conserved S-x-E casein kinase recognition motif. It is interesting to note that the phosphorylated form of this residue would then have similar physicochemical properties to the glutamic acid exposed in refolded influenza A virus HA. However, it seems unlikely that fusion conformation HA is a major component of the purified virus preparation, and the functional significance of this residue is unclear.

**Figure 5:**
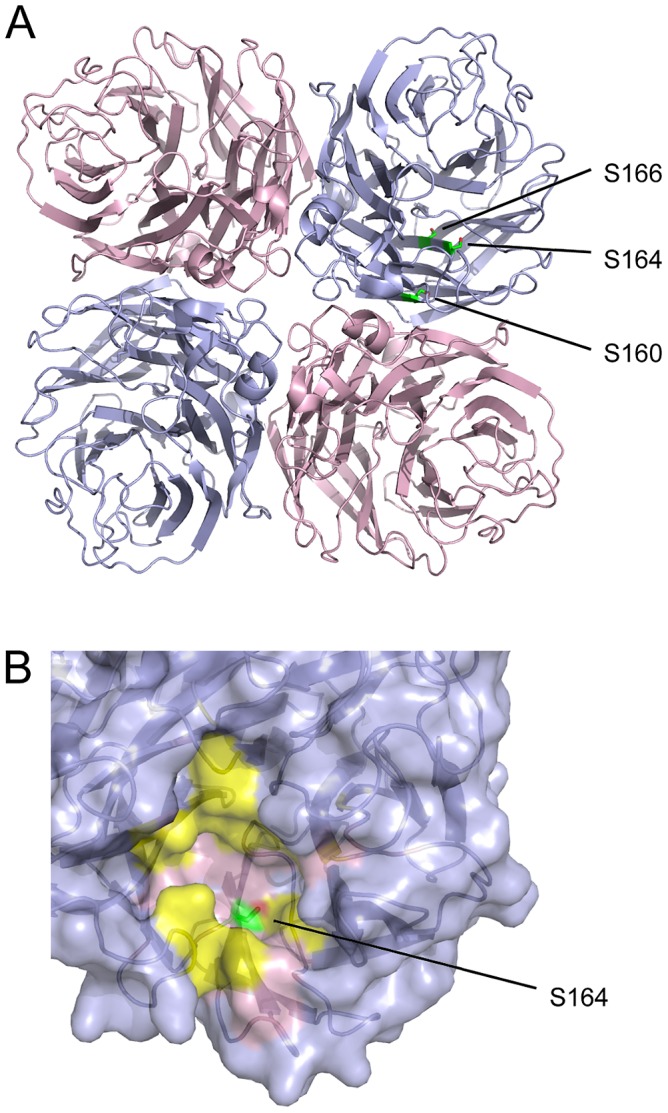
Location of phosphorylated residues in neuraminidase. (A) The position of S160/S164/S166 in the head domain of an N1-subtype NA, viewed facing the virion surface (PDB 3BEQ [86]). Head domains of the NA tetramer are shown in light blue and pink. (B) The position of S164 in the NA active site, with catalytic residues in yellow and framework residues in pink.

For the NA of the influenza A virus WSN (N1 subtype), we detected phosphorylation (along with an artefactual carbamidomethylation of C168) which could be plausibly assigned to one of three serines: residues 160, 164 or 166. All three serines are highly conserved in N1 neuraminidases (Table 1). When the NA consensus sequences of different influenza A virus subtypes and influenza B virus lineages are compared, S160 is not conserved, S164 is serine for all subtypes and lineages, and S166 is either serine or threonine (data not shown). S166 is buried and so is unlikely to be phosphorylated (Figure 5A [86]). S160 is positioned at the interface of two head domains in the NA tetramer (Figure 5A). Assuming that all structural NA is assembled into tetramers, this site would presumably only be accessible to kinases prior to tetramer assembly in the endoplasmic reticulum. S164, as well as being conserved in all influenza A and B virus NAs, is positioned in a more obviously accessible location. It lies at the base of a pocket containing the neuraminidase active site, and is one of the supporting framework residues of the active site (Figure 5A, B) [87]. Phosphorylation would interfere with its polar contact with the framework residue E212, and the negative charge it introduces could potentially disrupt interactions with sialic acid, reducing the ability of newly formed viruses to leave the cell. Mutations shown to confer neuraminidase inhibitor resistance lie on the opposite side of the pocket to S164 [88], suggesting that phosphorylation would not affect known mechanisms of drug resistance.

### Phosphorylation of the Polymerase and Nucleoprotein

Despite previous reports that PB1 and PA were phosphoproteins within infected cells [14], [20], [25], we did not detect phosphorylations in the polymerase proteins of any of the purified viruses, whether or not optimised conditions were used (Table S2). Two alternative approaches were used to analyse the polymerase proteins of WSN in 293 T cells. In one approach, cells were infected with a modified WSN virus which expressed PB2 protein fused to a C-terminal tag [89]. The tag was used to isolate PB2-containing complexes, including RNPs (Figure S1B). In a second approach, the polymerase proteins were expressed by transfection and a tag fused to the C-terminus of PB1 was used to purify it from cell lysates, along with bound PA and PB2 (Figure S1C). In infected cells we detected phosphorylation of PB2 at S742 and of PA at S224 or S225, with S225 the more likely assignment. We also detected phosphorylation of NP, as discussed below (Table 1). In the transfected cells we identified a single phosphopeptide, derived from PB1, with phosphorylation of T223 (as well as artefactual oxidation of M227; Table 1). The failure to detect phosphorylated polymerase protein in virions suggests that only non-phosphorylated polymerase proteins are packaged into the virus, though this may merely reflect a stochastic failure to detect the relevant phosphopeptides in the samples analysed.

**Figure 6:**
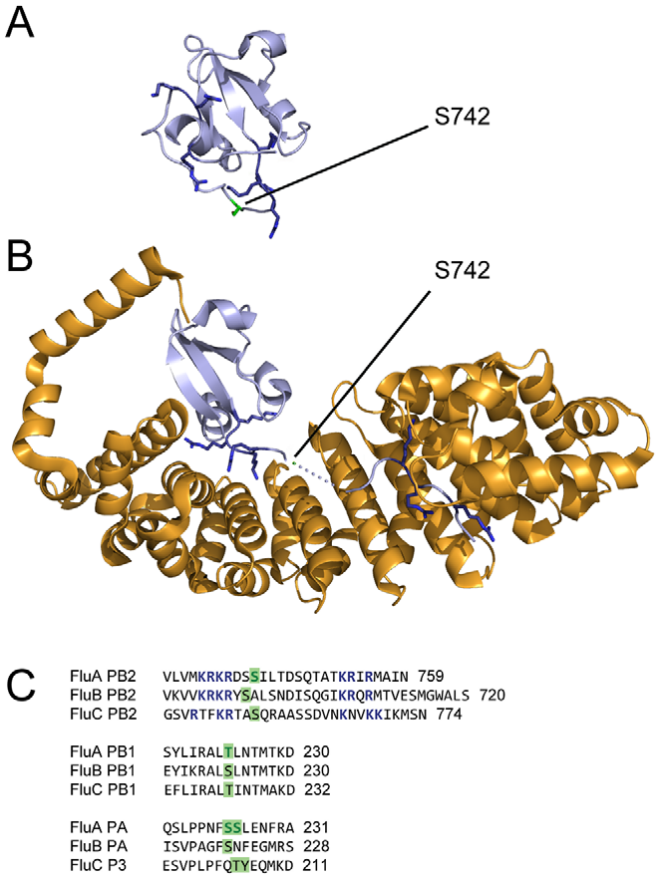
Location of phosphorylated residues in the polymerase. The position of S472 in the C-terminal tail of PB2 when (A) unbound (PDB 2GMO [91]) and (B) bound (PDB 2JDQ [91]) to importin α5. PB2 is shown in light blue and the importin in gold. In the bound form residues 742–747 are not resolved in the structure, and are indicated by a dotted line; the position of S742 has been estimated. (C) Portions of the consensus sequences of PB2, PB1 and PA from influenza A, B and C viruses. Colours are as in Figure 2; basic residues of the bipartite NLS of influenza A virus PB2, and orthologous residues in the influenza B and C virus sequences, are blue.

In PB2, S742 forms part of a flexible C-terminal tail containing the protein's bipartite NLS [90], [91]. This tail unfolds to allow the protein to bind to alpha importins (Figure 6A, B) [91]. The phosphorylation site consists of highly conserved residues between the two parts of the NLS. This arrangement is conserved in influenza B viruses and apparently also in influenza C viruses, suggesting a functional role (Figure 6C). As discussed above, phosphorylation at or near to NLSs regulates interactions with nuclear import factors [50]–[52]. In the case of PB2, a co-crystal structure of the C-terminus of PB2 bound to importin α5 [91] shows that S742, although in a flexible region not resolved in the structure, is positioned near to the surface of the importin (Figure 6B; the adjacent 741 residue is present in the structure close the importin surface). Phosphorylation of this residue is therefore highly likely to affect importin binding, thereby regulating the nuclear import of PB2. Whether phosphorylation would promote or inhibit nuclear import is unclear from the structure alone, and as the approach used here does not distinguish monomeric PB2, prior to nuclear import, from PB2 present in an RNP, the stage at which this regulation is applied cannot yet be determined.

In PB1 T223 is highly conserved, and serines or threonines are conserved at the corresponding residue in influenza B and C viruses (Figure 6C). In the primary sequence of PB1 T223 is between the NLS/RanBP5-binding site [92], [93] and the core promoter binding and polymerase motifs [94]–[98], suggesting a possible role in regulating nuclear import or RNA binding. However, as the structure and function of this region of PB1 are unknown it is hard to draw firm conclusions about the effect of its phosphorylation.

In PA S224 is highly conserved in influenza A viruses. In contrast, S225 (a better match to the spectrum, and in an S-x-E casein kinase consensus) is only present in 69% of isolates, with most of the remainder having cysteine at this position (Table 1). Conserved serines or threonines can be found in a similar position in influenza B and C viruses (Figure 6C). TheS224/S225 site is positioned in a region of unknown structure and function, between the N-terminal endonuclease domain and the C-terminal PB1-interacting domain of PA [99]. In a previous analysis of possible phosphorylation sites in influenza A/Victoria/3/75, a strain in which position 225 is cysteine, mutation of S224 to alanine was shown not to affect RNP activity or the apparent proteolytic activity of PA [25]. The effect of phosphorylation at this site is therefore currently unclear.

The nucleoprotein (NP) is, after M1, the most abundant protein in the virus (Figure 1A) [44], and is known to be a phosphoprotein [6], [17], [18]. Phosphorylation occurs at multiple sites, predominantly serines, and can vary between viral strains and host species, as well as during the course of an infection [7], [12], [13]. Serine 3 (the residue is, very unusually, threonine in WSN), accounts for the majority of N-terminal phosphorylation in infected cells [7], but is not detected in virions [13]. Additional phosphorylation has been mapped to the C-terminal 196 residues of the protein [7].

For WSN virus without the use of optimised conditions, phosphorylation was readily detected at either S402 or S403 (Table 1). We detected the same phosphorylation in WSN NP from cell lysates, both when RNPs were purified from infected cells, and when an N-terminal tag was used to purify NP expressed by transfection in uninfected 293 T cells (Figure S1B, C; Table S2). While S402 is highly conserved, S403 is an unusual feature of WSN and is more typically an alanine (Table 1). In the PR8-derived NP of the influenza A CVVs, for which residue 403 is an alanine, S402 was unambiguously phosphorylated (Table S2). It is likely that this residue accounts for much of the previously observed phosphorylation of the C-terminal portion of NP [7].

Optimised mass spectrometry conditions allowed us to detect additional sites of NP phosphorylation in the influenza A virus WSN. In virions, phosphorylation was detected for S9/Y10, S165 (with an artefactual carbamidomethylation of C164), S457 and T472/S473. In RNPs purified from infected cell lysates phosphorylation was detected at S9/Y10, S165 (again with an artefactual carbamidomethylation), Y296/S297, and S377/S378. All of these residues are highly conserved in influenza A viruses, with the exception of S377 (59% conserved, with the majority of other usages being asparagines), and S473 (which is typically asparagine, and which has been speculated to play a role in strain-specific phosphorylation of NP [13]). Peptides containing residue 3 were hard to identify due to tryptic cleavage sites present very close to the N-terminus (K4, K7, R8). However, when a peptide containing T3 was identified in purified virions it was N-terminally modified but not phosphorylated (Tables 3, S3), consistent with previous observations that residue 3 was not phosphorylated in virions, and that phosphorylation of this residue may be strain-specific [13].

NP has been reported to contain multiple NLSs [100], but the primary signal for nuclear import of free NP and of RNPs is an unconventional NLS located in the N-terminus [101]–[103]. Treatment with the phosphorylation stimulator TPA and the protein kinase inhibitor H7 showed that the nuclear accumulation of NP is inhibited by phosphorylation [101]. Mutational analysis suggests that phosphorylation of S3, which is adjacent to the N-terminal NLS of NP, inhibits its nuclear accumulation [8]. S9 and Y10, whose phosphorylation is detected here, are within the sequence of the N-terminal NLS [101], [102], and their phosphorylation would also be expected to inhibit nuclear import [104]. It therefore appears that phosphorylation may regulate the nuclear import not only of M1 and PB2 (and potentially of NS1 and PB1) but also of NP, and through it of the viral genome.

**Figure 7:**
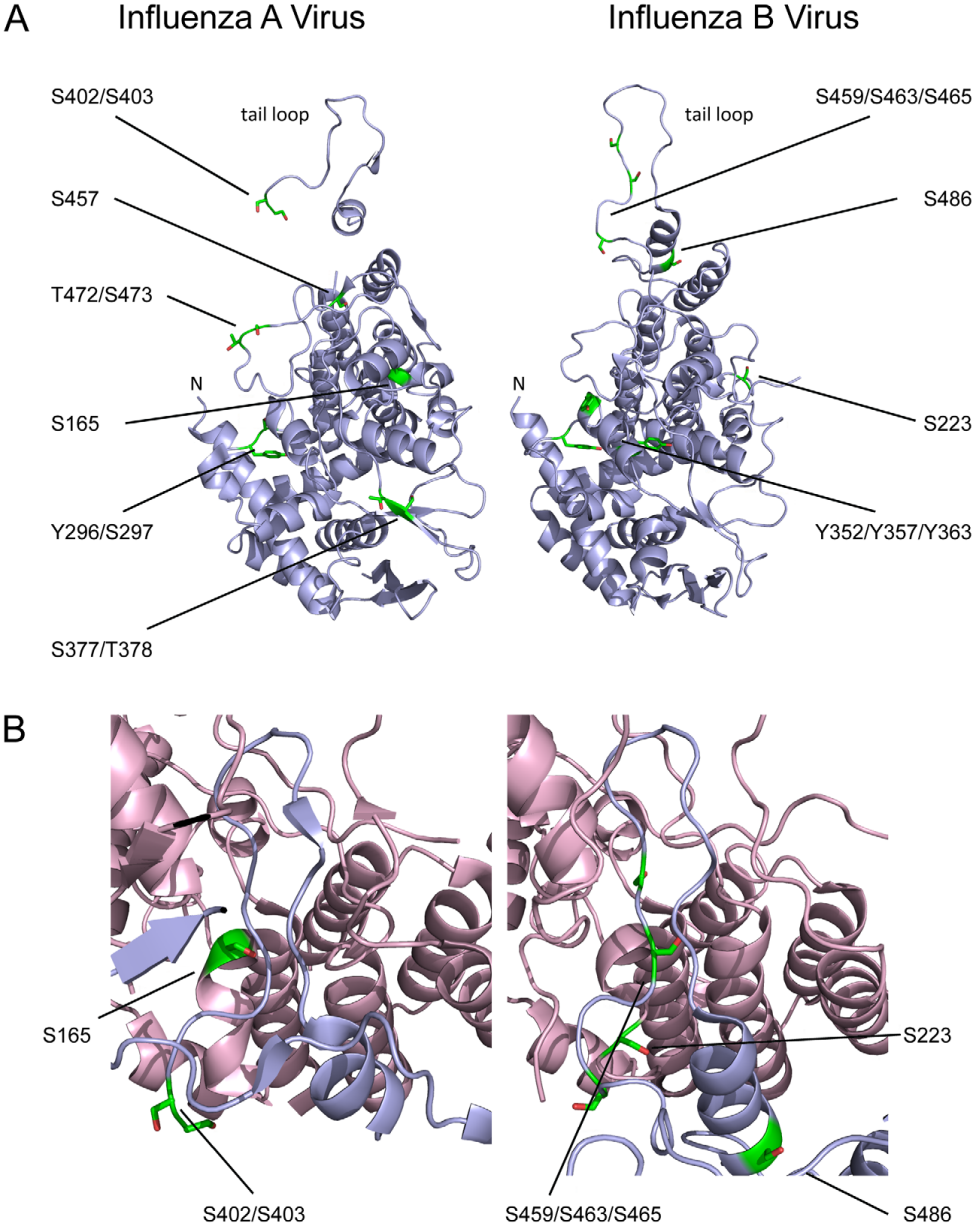
Location of phosphorylated residues in the nucleoprotein. (A) Location of phosphorylated residues in NP of influenza A virus WSN (PDB 2IQH [105]) and influenza B virus (PDB 3TJ0 [108]). The structures do not include N-terminal residues, including S9 and Y10 of influenza A virus NP and S50 and T55-S58 of influenza B virus NP. The N-termini of the resolved structures and tail loops are indicated; in the orientation shown, the RNA-binding grooves are on the far side of the molecules. (B) The oligomerisation of NP of influenza A virus or influenza B virus via the tail loop (blue) and groove (pink), with phosphorylated residues highlighted.

The structure of the N-terminus of NP, including S3/T3, S9 and Y10, is not known. All other sites detected in this study are located on the surface of the NP monomer (Figure 7A), supporting their identification as phosphorylated residues. As none of the residues were part of the RNA-binding groove of NP [105], it is unlikely that phosphorylation would interfere directly with RNA binding. In the structure of a WSN NP trimer, S165 and S457 participate in intermolecular van der Waals bonds, and S165, S402, S403 and S457 participate in intermolecular hydrogen bonding [105] – interactions that might be disrupted by phosphorylation. Of particular interest, S402/S403 and S165 are present in the ‘tail loop’ and ‘groove’ (respectively) which mediate NP oligomerisation (Figure 7B) [105], [106]. Phosphorylation could therefore plausibly interfere with the oligomerisation of NP.

In influenza B viruses we detected phosphorylation of S50 even without the use of optimised conditions (Tables 2, S2). This residue is found in a disordered N-terminal region with no homology to influenza A virus NP sequences [107], [108] and, despite the relatively high sequence variation of this region (data not shown), is absolutely conserved. Using optimised mass spectrometry conditions we identified additional phosphorylation sites, at T55/T56/S57/S58, S223, Y352/Y357/Y363, S459/S463/S465 and S486 (Figure 7A). With the exception of S57, which is an isoleucine in 20% of isolates, all of these residues are highly conserved (Tables 2, S2). Despite significant differences in the primary sequences of NP from influenza A and B viruses, the locations of several phosphorylation sites are conserved in the tertiary structures of influenza A and B viruses [105], [108]. Y296/S297 (A) and Y352-Y363 (B) are both predicted to be targets of the kinase Cdc2 (Tables 1, 2 and S2) and occupy similar positions on a loop in the tertiary structure (Figure 7A), though the functional significance of this site is unclear. Strikingly, in the tail loop/groove region, phosphorylation is detected in both genera of virus at conserved serines in the N-terminal end of the tail loop (S402/S403 (A), S459/S463/S465 (B); Figure 7B), and at a conserved serine within the groove (S165 (A), S223 (B); Figure 7B).

### The Effects of Mutating Phosphorylation Sites in Influenza A Virus Nucleoprotein

**Figure 8:**
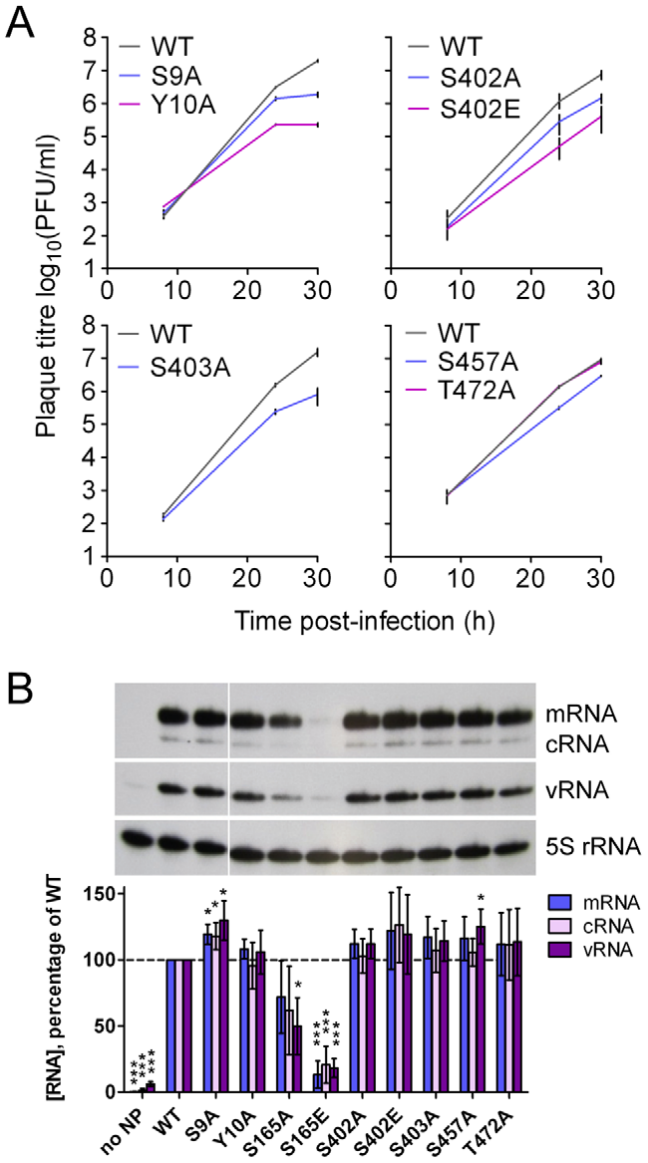
Effect of mutating phosphorylated residues in the nucleoprotein. (**A**) WSN viruses were generated containing the indicated mutations in NP. MDBK cells were infected at an MOI of 0.001, and virus harvested at the indicated time points. The mean and range of two experiments, or, for S402A and S402E, the mean and standard deviation of 4 experiments, is shown. For S402A and S402E differences from WT were tested by Student's unpaired 2-tailed t-tests at each time point; for both mutants the differences at 8 h are not significant, those at 24 h significant at p<0.05 and those at 30 h significant at p<0.005. (B) To assess the function of mutated NP proteins in transcription and replication, RNP reconstitutions were performed in 293 T cells and RNA species at 22 h post-transfection measured by primer extension and autoradiography. A representative image is shown, along with the mean and s.d. of 4 experiments, relative to WT. Differences from WT were tested using one-sample t-tests: * p<0.05, *** p<0.0005.

To assess the importance of phosphorylation sites found in NP in influenza A virions, we introduced alanine mutations into WSN viruses at the N-terminus, in the tail loop/groove oligomerisation domain, and in the C-terminus (Figure 8A). Mutations at the N-terminus that removed phosphorylation sites had pronounced effects on viral growth kinetics: S9A reduced viral titre by approximately 10-fold at 30 h post-infection (p.i.), and Y10A by 100-fold. In contrast, and consistent with their distance from known functional sites in NP, mutations at the C-terminus had little or no effect on viral growth: S457A caused a slight reduction in titre (3-fold at 30 h p.i.), whereas T472A had no effect.

In the tail loop-groove region, virus with the S165A mutation could not be produced in three separate attempts, despite the generation of WT virus, suggesting that this residue may be essential for viral growth. Despite phosphorylation of S402 being readily detected, an S402A mutation caused a relatively small defect in growth (5-fold at 30 h p.i.). Replacing the residue with glutamic acid, which is approximately similar in size and charge to phosphoserine, reduced the titre by approximately 20-fold at 30 h p.i., suggesting that although S402 phosphorylation is readily detected in the virion, constitutive phosphorylation of this residue is not optimal for viral growth. Although S403 is an alanine in the majority of influenza A virus strains, an S403A mutation caused a 10–100-fold growth defect at 30 h p.i., suggesting a specific requirement of WSN for serine at this position. A similar defect was seen when both S402 and S403 were mutated to alanines, removing the phosphorylation sites entirely (data not shown).

To assess the roles of NP phosphorylation sites in transcription and replication (including the S165 site, which could not be mutated in a virus) we performed RNP reconstitutions in 293 T cells (Figure 8B). The majority of the mutations did not reduce the transcription or replication of the genome – indeed, very small though statistically significant increases in activity were seen for the S9A and S457A mutations. This suggests that the growth defects caused by these mutations relate to NP functions not required for transcription and replication during RNP reconstitutions, for example viral entry, trafficking to the cell surface, viral assembly or immune regulation. The only residue which appeared to be important for RNP activity was S165. Mutating this residue to alanine caused a moderate decrease in RNP activity, but the phosphomimetic glutamic acid mutation caused a substantial and significant reduction in both transcription and replication. This is consistent with structural predictions, which suggest that phosphorylation of this residue would be inhibitory to NP oligomerisation and hence to RNP assembly (Figure 7B). The presence of a phosphorylation site in the oligomerisation groove of NP is a conserved feature of both influenza A and B viruses (Figure 7B). This could be due to a structural requirement for serine at this position, with phosphorylation simply a deleterious side effect. However, it is interesting to note that the reversible nature of phosphorylation could provide the virus with a mechanism for regulating NP oligomerisation and RNP assembly.

### Concluding Remarks

Mass spectrometry allows the detection of specific sites of protein phosphorylation. By applying this technique to a selection of influenza A viruses and an influenza B virus we have identified 39 phosphorylation sites, 36 of them novel. Unmodified peptides were also detected, consistent with phosphorylation typically acting on a subset of the proteins present in the cell. Strikingly, we found that a number of phosphorylation sites were conserved between different strains and even genera, underlining the fundamental importance of phosphorylation in the life cycles of influenza viruses.

Patterns of phosphorylation were similar between WSN grown in MDBK cells and reassortants of the similar PR8 virus grown in embryonated eggs and MDCK cells. This suggests that the similarities between these viruses are more important in determining phosphorylation patterns than the substantial differences between the hosts in which they were grown. From this it is reasonable to infer that the phosphorylation sites reported in this study will, in most cases, be similar to those found in other hosts, including in natural infections. Of the phosphorylation sites detected in PR8 reassortants only one was not also detected in WSN (M1 T37, detected in an MDCK-grown virus). As detection of phosphorylation by mass spectrometry is a stochastic process we consider this likely to reflect sampling variation rather than a difference in phosphorylation patterns. For the same reason, failure to detect phosphorylation at particular sites in this study (for example, at residues orthologous to phosphorylation sites in influenza A and B virus M1; Figure 2A) does not exclude phosphorylation at these positions.

By considering the position of sites of phosphorylation with respect to known structural and functional motifs we have been able to suggest cases where phosphorylation is likely to affect viral protein function. Phosphorylation appears to regulate three broad categories of function: viral entry and exit (HA and NA), nuclear localisation (PB2, NP, M1, NEP, and possibly PB1 and NS1), and multimerisation (NP, M2 and NS1). In addition, a number of phosphorylations did not have an obvious function, and it is likely that some of these phosphorylations are non-essential, arising through interactions with cellular kinases that confer no fitness advantage to the virus [109]. This has previously been suggested for phosphorylation of M2 S64 [11], [21], though we identify here an alternative, though relatively uncommon, phosphorylation of T65 which may compensate for S64 loss.

The functions of a number of phosphorylation sites in NP of the influenza A virus WSN were tested experimentally, and were found to contribute to viral growth in cell culture and to RNP activity to varying extents. In arguing for a functional role for phosphorylation, studies of this sort are suggestive, though further studies will be required to address the possibility that the mutations may introduce unrelated structural changes, or that viral fitness and/or RNP activity may depend on the residues being present but unphosphorylated. However, by combining arguments from evolutionary conservation, structural context and experimental evidence, a convincing case can be made for the existence of multiple functional phosphorylation sites.

Like most viral proteins NP is multi-functional [110], and, as discussed above, its phosphorylation state has been shown to change during the course of an infection. This study concentrates on proteins packaged into viral particles, but it is important to recognise that these proteins may be subject to a series of phosphorylation and de-phosphorylation events during the viral life cycle. As such, the patterns of phosphorylation reported here represent, for the most part, the final stage in a series of interactions, functional and non-functional, between the proteins of influenza viruses and the kinases and phosphatases of the cells they infect. The increasing power of mass spectrometry to identify phosphorylation patterns in complex samples will make it possible in the future to map the dynamics of phosphorylation over the entire course of an infection [24].

Phosphorylation provides a promising target for antiviral chemotherapy, particularly as targeting cellular kinases reduces the capacity for viral escape mutations. Treatment of cells with protein kinase inhibitors interferes with multiple stages in the influenza virus life cycle, including nuclear import, transcription, protein synthesis, nuclear export and viral budding [69], [111]–[114]. Some of these effects are due to changes in phosphorylation of host factors [115], [116], but altered phosphorylation of viral proteins also has direct effects on the viral life cycle. It is interesting to note that some of the kinases predicted to phosphorylate sites found in this study, in particular protein kinase C, are targeted by kinase inhibitors that are known to affect influenza viruses (Tables 1, 2 and S2; [101], [111], [112]). Phosphorylation stimulators and kinase inhibitors affect the nuclear import of NP, and kinase inhibitors prevent the nuclear export of NEP, as discussed above. A number of kinase inhibitors, some already approved for cancer treatment, are being investigated as antiviral drugs [117], [118], including as treatments for influenza [119], [120]. Narrow-spectrum kinase inhibitors such as these, effective at sub-toxic concentrations, provide a promising route for antiviral therapy. The identification in this study of specific and highly conserved phosphorylation sites suggests that influenza viruses have a fundamental requirement for cellular kinases (predictions of which are given Tables 1, 2 and S2) and therefore provides a foundation for the targeted development of novel antiviral strategies.

## Materials and Methods

### Cells, Viruses and Plasmids

Madin-Darby Bovine Kidney epithelial (MDBK) cells, Madin-Darby Canine Kidney epithelial (MDCK) cells and 293 T human embryonic kidney cells were maintained at 37°C and 5% CO_2_ in Modified Eagle Medium with Earle's salts (MEM; PAA) supplemented with 2 mM L-glutamine and 10% fetal calf serum (FCS).

Influenza A/WSN/33 virus (WSN) [121] was cultured on MDBK cells in MEM supplemented with 2 mM L-glutamine and 0.5% fetal calf serum (FCS). Candidate Vaccine Viruses (CVVs) were a kind gift of Dr Othmar Engelhardt (National Institute of Biological Standards and Controls, UK). NIB-74xp and NYMC X-187 have HA and NA genes of the influenza viruses A/Christchurch/16/2010 (A(H1N1)pdm09) and A/Victoria/210/2009 (H3N2), respectively, with the remaining genes from influenza A/Puerto Rico/8/1934 (PR8). NYMC X-181 has HA, NA and PB1 genes of influenza A/California/7/2009 (A(H1N1)pdm09) with the remaining genes from PR8. Influenza B/Brisbane/60/2008 virus is an egg-adapted clinical isolate. CVVs were propagated in embryonated chicken eggs. Additionally, NIB-74xp was cultured in MDCK cells in MEM with 2 mM L-glutamine, 0.14% bovine serum albumin (Sigma) and 0.75 µg/ml bovine pancreatic trypsin (Sigma). Plasmids for use in affinity purification [122]–[124], RNP reconstitution [125] and reverse genetics [121] have been described previously. Specific mutations were introduced into the plasmids by site-directed mutagenesis and confirmed by sequencing. Mutated WSN viruses were generated by reverse genetics, as previously described [121], [126]. Influenza A/WSN/33 PB2-Cstrep was generated using pPR7-PB2-Cstrep [89], [122] in place of pPOLI-PB2.

Plaque assays were performed on MDBK cells using standard techniques. RNP reconstitutions were performed in 293 T cells using segment 6 (NA) vRNA as a template, and RNA accumulation measured by primer extension, PAGE, autoradiography and phosphorimaging, as described previously [125].

### Affinity Purifications

Affinity purifications of PB1-TAP using a Tandem Affinity Purification (TAP) tag [123], [124] were carried out in transfected 293 T cells as previously described. To purify RNPs, 293 T cells were infected with influenza A/WSN/33 PB2-Cstrep [89], [122] at an MOI of 5. At 6 h post-infection (p.i.) cells were harvested and resuspended in phosphate buffered saline, pelleted at 450 *g*/5 min/4°C, and placed on a rotating wheel for 1 h at 4°C in lysis buffer (50 mM Tris-HCl pH 8.0, 200 mM NaCl, 33% glycerol, 0.5% NP-40 and 1 mM dithiothreitol with protease inhibitor cocktail (Roche)). The soluble fraction was separated by centrifugation at 17 000 *g*/3 min/4°C, diluted 1∶5 with binding buffer (20 mM Tris-HCl pH 8.0, 200 mM NaCl and protease inhibitor cocktail (Roche)) and incubated overnight at 4°C with 200 µl of 50% suspension Strep-Tactin Superflow high capacity resin (IBA GmbH). The resin was washed four times with wash buffer (100 mM Tris-HCl pH 8.0, 150 mM NaCl, 1 mM EDTA, 0.1% NP-40, 10% glycerol, 1 mM phenylmethylsulfonyl fluoride), and proteins were eluted in 2 ml of elution buffer (100 mM Tris-HCl pH 8.0, 150 mM NaCl, 1 mM EDTA, 0.1% NP-40, 10% glycerol, protease inhibitor cocktail (Roche), 2 mM d-desthiobiotin) for 2 h on a rotating wheel at 4°C. Elution fractions were subsequently concentrated using Amicon Ultra-4 (3K MWCO) centrifugation devices.

### Virus Purification

Viruses grown in cell culture were harvested from the growth media of four T175 flasks of infected cells (120 ml; 10^9^–10^10^ plaque-forming units (PFU) of virus for WSN) at 2 days p.i. The medium was clarified by low-speed centrifugation (2000 *g*/30 min then 18 000 *g*/30 min, at 4°C), then layered onto a cushion of 30% sucrose in NTC (100 mM NaCl, 20 mM Tris-HCl pH 7.4, 5 mM CaCl_2_) and pelleted by ultracentrifugation (112 000 *g*/90 min/4°C in an SW 28 rotor (Beckman Coulter)). Pellets were resuspended in NTC and spun through a 30–60% sucrose gradient in NTC (209 000 *g*/150 min/4°C in an SW 41 Ti rotor (Beckman Coulter)) to produce a visible band of virus which was drawn off with a needle and pelleted through NTC (154 000 *g*/60 min/4°C in an SW 41 Ti rotor) and resuspended in a small volume of NTC (typically 120 µl, containing 10^8^–10^9^ PFU WSN). A similar method was used to purify CVVs from infected eggs. Briefly, infected allantoic fluid was harvested, filtered and mixed with sodium azide. Virus was pelleted by ultracentrifugation, resuspended and spun on 10–40% sucrose gradients to produce a visible band of virus which was harvested and pelleted by ultracentrifugation. Samples of virus were taken to determine plaque titre; separated by SDS-PAGE and Coomassie or Silver stained according to standard techniques; or fixed with 2.5% glutaraldehyde, 2% paraformadehyde and 0.1% picric acid in 100 mM cacodylate buffer (pH 7.0), adsorbed onto formvar-coated grids, negative-stained with 2% aqueous uranyl acetate and examined by transmission electron microscopy using a FEI Tecnai 12 electron microscope.

### Mass Spectrometry

Samples of purified virus or PB2-Cstrep purified material were prepared for mass spectrometry by boiling in Laemmli buffer and running a short distance into a polyacrylamide gel (typically a precast 8–16% Precise Protein Gel (Thermo Scientific)) to remove detergent and salts; the entire sample was then cut out of the gel with a clean scalpel. As an alternative method, some WSN samples were boiled in 1.25% SDS, precipitated in −20°C acetone, and resuspended in 8 M urea, 25 mM ammonium bicarbonate. Whole cell lysates and TAP-purified samples were separated by SDS-PAGE and stained with Coomassie; bands of the appropriate electrophoretic mobility were excised with a clean scalpel. Samples were then washed with 50 mM ammonium bicarbonate in 50% acetonitrile, reduced with 10 mM DTT and 55 mM iodoacetamide or chloroacetamide and digested with 0.5 µg trypsin (Promega) at 37°C for 16 h. Peptides were extracted with 0.1% formic acid in 50% acetonitrile, lyophilised in a SpeedVac (Thermo Savant) and desalted using an in-house manufactured C18 purification tip. Enrichment for phosphopeptides using TiO_2_ [127] or IMAC [128] were carried out essentially as described previously; flow-through samples were also retained and analysed, and the data pooled with that of the enriched sample. Samples were lyophilised and stored at −20°C, then dissolved in 0.1% formic acid prior to mass spectrometry analysis.

All LC-MS/MS experiments were performed using either an Ultimate 3000 nano HPLC system (Dionex, Camberley, UK) run in direct injection mode, coupled to an LTQ XL Orbitrap mass spectrometer or a Q Exactive mass spectrometer (Thermo Electron, Hemel Hempstead, UK). Separation of peptides was performed by reverse-phase chromatography using a 15 cm (LTQ XL Orbitrap) or 25 cm (Q Exactive) by 75 µm inner diameter picotip analytical column (New Objective, Woburn, MA, USA), packed in house with Reprosil-Pur C18-AQ phase, 3 µm particle size (Dr Maisch, Germany), at a flow rate of 300 nl/min. Samples were typically resolved on a 120 min gradient. The LTQ XL Orbitrap mass spectrometer was operated in a “Top 5” and the Q Exactive in a “Top 10” data-dependent acquisition mode. Charge state +1 ions were rejected from selection and fragmentation and dynamic exclusion with 40 s was enabled.

### Analysis of Mass Spectra

Mass spectra were analysed using the Central Proteomics Facilities Pipeline (CPFP) [31]. For purified virions data from repeat experiments were merged to increase sample coverage; for searches of CVVs data from two injections of sample were merged. Peptide spectral matches were made to custom databases that concatenated the proteome of the relevant virus with that of the host (*Bos taurus* for MDBK cells, *Canis lupus familiaris* for MDCK cells, *Gallus gallus* for chicken eggs and *Homo sapiens* for 293 T cells) and with common contaminants and decoy sequences. For WSN the viral proteome was expanded to include experimentally confirmed and hypothetical proteins, as well as a translation of all six complete forward and reverse-sense reading frames from each viral segment. To identify peptides, CPFP uses iProphet [129] to combine searches made with Mascot (Matrix Science, London, UK), OMSSA [130] and X!TANDEM [131], with peptide identifications validated using PeptideProphet [132]. Combined protein identifications were then assigned using ProteinProphet [133] with a 1% false discovery rate. Searches were made for peptides with up to two missed cleavages and with common post-translational modifications including phosphorylation at S, T or Y. The Modification Localisation Score (ModLS) algorithm within CPFP was applied to phosphopeptide identifications to assess the confidence of phosphorylation site assignments. MS/MS search engines may not always assign phosphorylation at the site that is most probable given the spectral evidence, since their emphasis is on peptide identification rather than phosphorylation localisation. The ModLS algorithm re-assigns phosphorylation sites when the search-engine reported assignment is a poorer fit to the spectral evidence than an alternative localisation. PTMScores are calculated as previously described [32], with the exception that the peak depth per 100 m/z units is varied between 1 and 10, and the highest scoring result obtained is used. ModLS assesses the confidence with which phosphorylation sites can be assigned on a peptide by the difference between the highest and second-highest PTMScores calculated for all possible localisations of phosphorylation on a given peptide. Assessment of the ModLS algorithm using spectra from mixtures of known phosphopeptides [134] shows that a ModLS of <14 gives a false localisation rate of <1% (data not shown). As N-terminal modifications are not reported correctly in the version of CPFP used, peptide spectra matched to N-terminal peptides by Mascot were searched manually. All spectra of reported modified peptides were manually inspected, and representative spectra are given in Figure S3.

### Sequence and Structural Analysis

Full-length influenza protein sequences were downloaded from GISAID (http://platform.gisaid.org) or from the NCBI influenza virus resource (http://www.ncbi.nlm.nih.gov/genomes/FLU/FLU.html) and aligned with MAFFT [135] using the FFT-NS-2 method. The number of sequences analysed for each protein is given in Table S4. Alignments were edited using BioEdit [136] and consensus sequences were generated with Jalview [137]. Protein structures were visualised using PyMOL (Schrödinger LLC); predictions of secondary structure were made using JPred 3 [138]. Phosphorylation sites were predicted using NetPhos 2.0 and NetPhosK 1.0 [58].

## Supporting Information

Figure S1 **Purification of viral proteins.** (A) Candidate vaccine viruses (CVVs) were purified from embryonated chicken eggs or the culture medium of infected MDCK cells, separated by SDS-PAGE and silver stained. (B) 293 T cells were infected with A/WSN/33 PB2-Cstrep, and affinity purification was used to purify PB2-Cstrep and associated proteins from cell lysates. Proteins were separated by SDS-PAGE and silver stained. Key proteins are identified by electrophoretic mobility. (C) Affinity purification of PB1-TAP (with co-purifying PB2 and PA) and, separately, TAP-NP from transfected 293 T cells. Protein was separated by SDS-PAGE and stained with Coomassie Brilliant Blue; the indicated viral proteins were excised from the gel and submitted for LC-MS/MS.
(TIF)

Figure S2 **Coverage of sequences.** The full sequences of all proteins to which peptides were matched, with peptides assigned by CPFP shaded. Peptides with N-terminal acetylation (Table S3) were scored separately and are not necessarily shown.
(PDF)

Figure S3 **Representative spectra.** Fragment ion spectra for N-terminal peptides and for phosphorylated peptides, annotated by Mascot or CPFP. The clearest spectrum showing each modification referred to in the text is presented (including overlapping peptides and unmodified peptides where available). Each spectrum was manually inspected.
(PDF)

Table S1 **Protein sequence coverage.**
(DOC)

Table S2 **Summary of phosphopeptides.**
(DOC)

Table S3 **N-terminal modifications.**
(DOC)

Table S4 **Number of sequences used in alignments.**
(DOC)
