# Supplementary figures and images for: Mapping the Phosphoproteome of Influenza A and B Viruses by Mass Spectrometry
Source: PLoS Pathog. 2012 Nov 8;8(11):e1002993. doi: 10.1371/journal.ppat.1002993 (PMC3493474; doi:10.1371/journal.ppat.1002993)

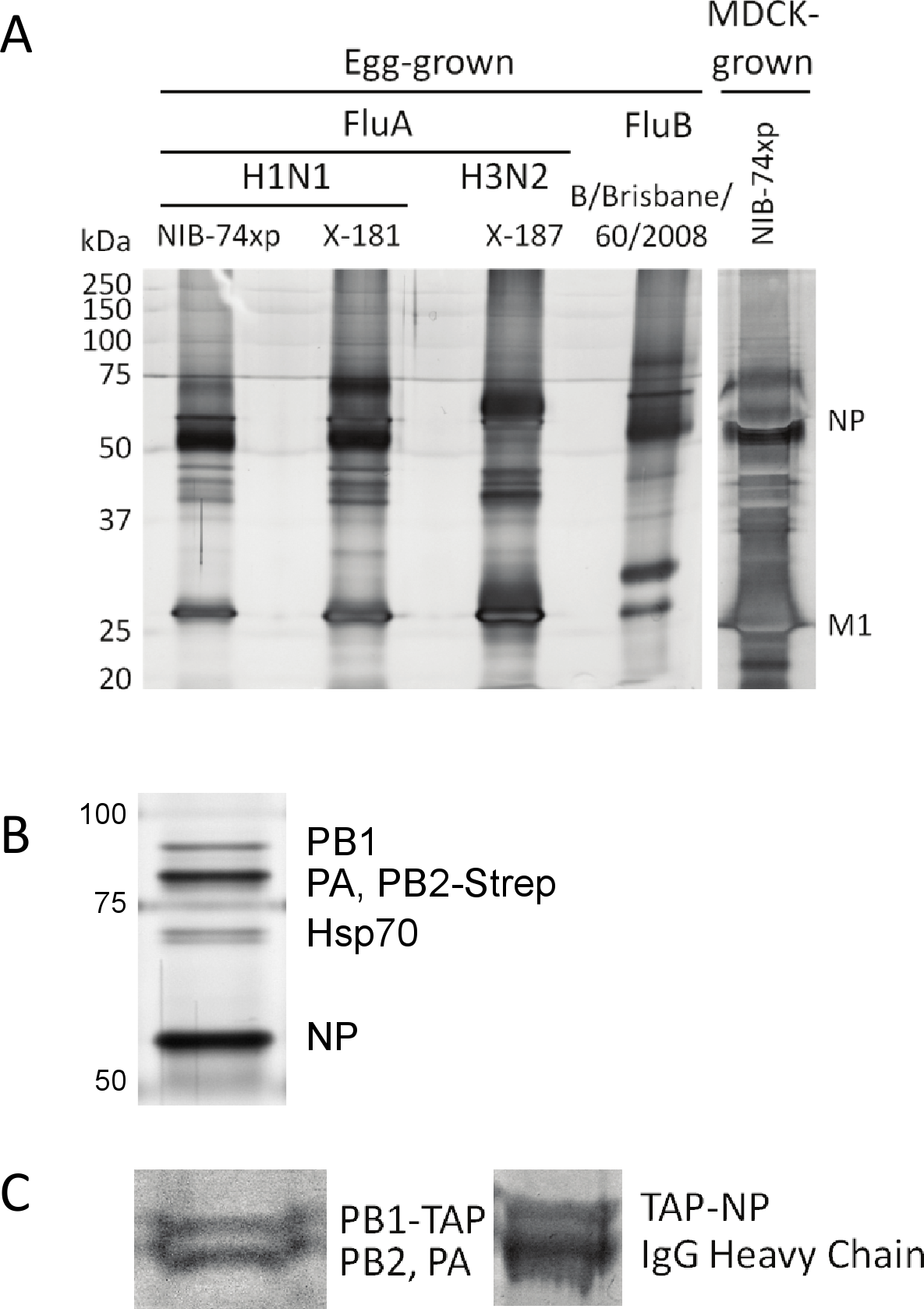

Supplement: Figure S1 — Purification of viral proteins. (A) Candidate vaccine viruses (CVVs) were purified from embryonated chicken eggs or the culture medium of infected MDCK cells, separated by SDS-PAGE and silver stained. (B) 293 T cells were infected with A/WSN/33 PB2-Cstrep, and affinity purification was used to purify PB2-Cstrep and associated proteins from cell lysates. Proteins were separated by SDS-PAGE and silver stained. Key proteins are identified by electrophoretic mobility. (C) Affinity purification of PB1-TAP (with co-purifying PB2 and PA) and, separately, TAP-NP from transfected 293 T cells. Protein was separated by SDS-PAGE and stained with Coomassie Brilliant Blue; the indicated viral proteins were excised from the gel and submitted for LC-MS/MS. (TIF) [file ppat.1002993.s001.tif]
