# Supplementary material for: Mapping the Phosphoproteome of Influenza A and B Viruses by Mass Spectrometry
Source: PLoS Pathog. 2012 Nov 8;8(11):e1002993. doi: 10.1371/journal.ppat.1002993 (PMC3493474; doi:10.1371/journal.ppat.1002993)
Supplement: Figure S2 — Coverage of sequences. The full sequences of all proteins to which peptides were matched, with peptides assigned by CPFP shaded. Peptides with N-terminal acetylation (Table S3) were scored separately and are not necessarily shown. (PDF) [file ppat.1002993.s002.pdf]

WSN (not enriched for phosphopeptides)

PB2

MERIKELRNL MSQSRTREIL TKTTVDHMAI IKKYTSGRQE KNPALRMKWM MAMKYPITAD KRITEMIPER NEQGQTLWSK
MNDAGSDRVM VSPLAVTWWN RNGPVTSTVH YPKIYKTYFE KVERLKHGTF GPVHFRNQVK IRRRVDINPG HADLSAKEAQ
DVIMEVVFPN EVGARILTSE SQLTTTKEKK EELQGCKISP LMVAYMLERE LVRKTRFLPV AGGTSSVYIE VLHLTQGTCW
EQMYTPGGEA RNDDVDQSLI IAARNIVRRA TVSADPLASL LEMCHSTQIG GIRMVNILRQ NPTEEQAVDI CKAAMGLRIS
SSFSFGGFTF KRTSGSSVKR EEEVLTGNLQ TLKIRVHEGY EEFTMVGRRA TAILRKATRR LIQLIVSGRD EQSIAEAIIV
AMVFSQEDCM IKAVRGDLNF VNRANQRLNP MHQLLRHFQK DAKVLFQNWG IESIDNVMGM IGILPDMTPS TEMSMRGVRI
SKMGVDEYSS AEKIVVSIDR FLRVRDQRGN VLLSPEEVSE TQGTEKLTIT YSSSMMWEIN GPESVLVNTY QWIIRNWETV
KIQWSQNPTM LYNKMEFEPF QSLVPKAVRG QYSGFVRTLF QQMRDVLGTF DTAQIIKLLP FAAAPPKQSG MQFSSLTINV
RGSGMRILVR GNSPVFNYNK TTKRLTVLGK DAGPLTEDPD EGTAGVESAV LRGFLILGKE DRRYGPALSI NELSNLAKGE
KANVLIGQGD VVLVMKRKRN SSILTDSQTA TKRIRMAIN

PB1

MDVNPTLLFL KVPAQNAIST TFPYTGDPPY SHGTGTGYTM DTVNRTHQYS ERGRWTTNTE TGAPQLNPID GPLPEDNEPS
GYAQTDCVLE AMAFLEESHP GIFETSCLET MEVVQQTRVD KLTQGRQTYD WTLNRNQPAA TALANTIEVF RSNGLTANES
GRLIDFLKDV MESMNKEEME ITTHFQRKRR VRDNMTKKMV TQRTIGKRKQ RLNKRSYLIR ALTLNTMTKD AERGKLKRRA
IATPGMQIRG FVYFVETLAR SICEKLEQSG LPVGGNEKKA KLANVVRKMM TNSQDTEISF TITGDNTKWN ENQNPRMFLA
MITYITRNQP EWFRNVLSIA PIMFSNKMAR LGKGYMFESK SMKLRTQIPA EMLASIDLKY FNDSTRKKIE KIRPLLIDGT
ASLSPGMMMG MFNMLSTVLG VSILNLGQKR HTKTTYWWDG LQSSDDFALI VNAPNHEGIQ AGVNRFYRTC KLLGINMSKK
KSYINRTGTF EFTSFFYRYG FVANFSMELP SFGVSGINES ADMSIGVTVI KNNMINNDLG PATAQMALQL FIKDYRYTYR
CHRGDTQIQT RRSFEIKKLW EQTHSKAGLL VSDGGPNLYN IRNLHIPEVC LKWELMDEDY QGRLCNPLNP FVNHKDIESV
NNAVIMPAHG PAKNMEYDAV ATTHSWIPKR NRSILNTSQR GILEDEQMYQ KCCNLFEKFF PSSSYRRPVG ISSMVEAMVS
RARIDARIDF ESGRIKKEEF TEIMKICSTI EELRRQK

PA

MEDFVRQCFN PMIVELAEKA MKEYGEDLKI ETNKFAAICT HLEVCFMYSD FHFIDEQGES IVVELGDPNA LLKHRFEIIE
GRDRTIAWTV INSICNTTGA EKPKFLPDLY DYKKNRFIEI GVTRREVHIY YLEKANKIKS EKTHIHIFSF TGEEMATKAD
YTLDEESRAR IKTRLFTIRQ EMASRGLWDS FRQSERGEET IEERFEITGT MRKLADQSLP PNFSSLENFR AYVDGFEPNG
YIEGKLSQMS KEVNARIEPF LKSTPRPLRL PDGPPCSQRS KFLLMDALKL SIEDPSHEGE GIPLYDAIKC MRTFFGWKEP
NVVKPHEKGI NPNYLLSWKQ VLAELQDIEN EEKIPRTKNM KKTSQLKWAL GENMAPEKVD FDDCKDVGDL KQYDSDEPEL
RSLASWIQNE FNKACELTDS SWIELDEIGE DAAPIEHIAS MRRNYFTAEV SHCRATEYIM KGVYINTALL NASCAAMDDF
QLIPMISKCR TKEGRRKTNL YGFIIKGRSH LRNDTDVVNF VSMEFSLTDP RLEPHKWEKY CVLEVGDMLL RSAIGHVSRP
MFLYVRTNGT SKIKMKWGME MRRCLLQSLQ QIESMIEAES SVKEKDMTKE FFENKSETWP VGESPKGVEE GSIGKVCRTL
LAKSVFNSLY ASPQLEGFSA ESRKLLLIVQ ALRDNLEPGT FDLGGLYEAI EECLINDPWV LLNASWFNSF LTHALR

HA

MKAFVLVLLY AFVATDADTI CIGYHANNST DTVDTIFEKN VAVTHSVNLL EDRHNGKLCK LKGIAPLQLG KCNITGWLLG
NPECDSLLPA RSWSYIVETP NSENGACYPG DFIDYEELRE QLSSVSSLER FEIFPKESSW PNHTFNGVTV SCSHRGKSSF
YRNLLWLTKK GDSYPKLTNS YVNNKGKEVL VLWGVHHPSS SDEQQSLYSN GNAYVSVASS NYNRRFTPEI AARPKVKDQH
GRMNYYWTLL EPGDTIIFEA TGNLIAPWYA FALSRGFESG IITSNASMHE CNTKCQTPQG SINSNLPFQN IHPVTIGECP
KYVRSTKLRM VTGLRNIPSI QYRGLFGAIA GFIEGGWTGM IDGWYGYHHQ NEQGSGYAAD QKSTQNAING ITNKVNSVIE
KMNTQFTAVG KEFNNLEKRM ENLNKKVDDG FLDIWTYNAE LLVLLENERT LDFHDLNVKN LYEKVKSQLK NNAKEIGNGC
FEFYHKCDNE CMESVRNGTY DYPKYSEESK LNREKIDGVK LESMGVYQIL AIYSTVASSL VLLVSLGAIS FWMCSNGSLQ
CRICI

NP

MATKGTKRSY EQMETDGERQ NATEIRASVG KMIDGIGRFY IQMCTELKLS DYEGRLIQNS LTIERMVLSA FDERRNKYLE
EHPSAGKDPK KTGGPIYRRV DGKWRRELIL YDKEEIRRIW RQANNGDDAT AGLTHMMIWH SNLNDATYQR TRALVRTGMD
PRMCSLMQGS TLPRRSGAAG AAVKGVGTMV MELIRMIKRG INDRNFWRGE NGRRTRIAYE RMCNILKGKF QTAAQRTMVD
QVRESRNPGN AEFEDLIFLA RSALILRGSV AHKSCLPACV YGSAVASGYD FEREGYSLVG IDPFRLLQNS QVYSLIRPNE
NPAHKSQLVW MACHSAAFED LRVSSFIRGT KVVPRGKLST RGVQIASNEN METMESSTLE LRSRYWAIRT RSGGNTNQQR
ASSGQISIQP TFSVQRNLPF DRPTIMAAFT GNTEGRTSDM RTEIIRLMES ARPEDVSFQG RGVFELSDEK ATSPIVPSFD
MSNEGSYFFG DNAEEYDN

NA

MNPNQKIITI GSICMVVGII SLILQIGNII SIWISHSIQT GNQNHTGICN QGSITYKVVA GQDSTSVILT GNSSLCPIRG
WAIHSKDNGI RIGSKGDVFV IREPFISCSH LECRTFFLTQ GALLNDKHSR GTFKDRSPYR ALMSCPVGEA PSPYNSRFES
VAWSASACHD GMGWLTIGIS GPDDGAVAVL KYNGIITETI KSWRKNILRT QESECTCVNG SCFTIMTDGP SDGLASYKIF
KIEKGKVTKS IELNAPNSHY EECSCYPDTG KVMCVCRDNW HGSNRPWVSF DQNLDYKIGY ICSGVFGDNP RPKDGTGSCG
PVSADGANGV KGFSYKYGNG VWIGRTKSDS SRHGFEMIWD PNGWTETDSR FSMRQDVVAM TDRSGYSGSF VQHPELTGLD
CMRPCFWVEL IRGLPEEDAI WTSGSIISFC GVNGDTVDWS WPDGAELPFT IDK

M1

MSLLTEVETY VLSIVPSGPL KAEIAQRLED VFAGKNTDLE VLMEWLKTRP ILSPLTKGIL GFVFTLTVPS ERGLQRRRFV
QNALNGNGDP NNMDKAVKLY RKLKREITFH GAKEIALSYS AGALASCMGL IYNRMGAVTT EVAFGLVCAT CEQIADSQHR
SHRQMVTTTN PLIRHENRMV LASTTAKAME QMAGSSEQAA EAMDIASQAR QMVQAMRTIG THPSSSAGLK DDLLENLQAY
QKRMGVQMQR FK

M2

MSLLTEVETP IRNEWGCRCN DSSDPLVIAA NIIEILHLIL WILDRLFFKC IYRRFKYGLK RGPSTEGVPE SMREEYRKEQ
QNAVDVDDGH FVNIELE

NS1

MDPNTVSSFQ VDCFLWHVRK RVADQELGDA PFLDRLRRDQ KSLRGRGSTL GLDIETATRA GKQIVERILK EESDEALKMT
MASVPASRYL TDMTLEEMSR HWFMLMPKQK VAGPLCIRMD QAIMDKNIIL KANFSVIFDR LETLILLRAF TEEGTIVGEI
SPLPSLPGHT DEDVKNAVGV LIGGLEWNNN TVRVSETLQR FAWRSSNENG RPPLTPKQKR KMAGTIRSEV

NEP

MDPNTVSSFQ DILMRMSKMQ LGSSSEDLNG IITQFESLKL YRDSLGEAVM RMGDLHSLQN RNGKWREQLG QKFEEIRWLI
EEVRHRLKIT ENSFEQITFM QALQLLLEVE QEIRTFSFQL I

WSN (enriched for phosphopeptides)

PB2

MERIKELRNL MSQSRTREIL TKTTVDHMAI IKKYTSGRQE KNPALRMKWM MAMKYPITAD KRITEMIPER NEQGQTLWSK
MNDAGSDRVM VSPLAVTWWN RNGPVTSTVH YPKIYKTYFE KVERLKHGTF GPVHFRNQVK IRRRVDINPG HADLSAKEAQ
DVIMEVVFPN EVGARILTSE SQLTTTKEKK EELQGCKISP LMVAYMLERE LVRKTRFLPV AGGTSSVYIE VLHLTQGTCW
EQMYTPGGEA RNDDVDQSLI IAARNIVRRA TVSADPLASL LEMCHSTQIG GIRMVNILRQ NPTEEQAVDI CKAAMGLRIS
SSFSFGGFTF KRTSGSSVKR EEEVLTGNLQ TLKIRVHEGY EEFTMVGRRA TAILRKATRR LIQLIVSGRD EQSIAEAIIV
AMVFSQEDCM IKAVRGDLNF VNRANQRLNP MHQLLRHFQK DAKVLFQNWG IESIDNVMGM IGILPDMTPS TEMSMRGVRI
SKMGVDEYSS AEKIVVSIDR FLRVRDQRGN VLLSPEEVSE TQGTEKLTIT YSSSMMWEIN GPESVLVNTY QWIIRNWETV
KIQWSQNPTM LYNKMEFEPF QSLVPKAVRG QYSGFVRTLF QQMRDVLGTF DTAQIIKLLP FAAAPPKQSG MQFSSLTINV
RGSGMRILVR GNSPVFNYNK TTKRLTVLGK DAGPLTEDPD EGTAGVESAV LRGFLILGKE DRRYGPALSI NELSNLAKGE
KANVLIGQGD VVLVMKRKRN SSILTDSQTA TKRIRMAIN

PB1

MDVNPTLLFL KVPAQNAIST TFPYTGDPPY SHGTGTGYTM DTVNRTHQYS ERGRWTTNTE TGAPQLNPID GPLPEDNEPS
GYAQTDCVLE AMAFLEESHP GIFETSCLET MEVVQQTRVD KLTQGRQTYD WTLNRNQPAA TALANTIEVF RSNGLTANES
GRLIDFLKDV MESMNKEEME ITTHFQRKRR VRDNMTKKMV TQRTIGKRKQ RLNKRSYLIR ALTLNTMTKD AERGKLKRRA
IATPGMQIRG FVYFVETLAR SICEKLEQSG LPVGGNEKKA KLANVVRKMM TNSQDTEISF TITGDNTKWN ENQNPRMFLA
MITYITRNQP EWFRNVLSIA PIMFSNKMAR LGKGYMFESK SMKLRTQIPA EMLASIDLKY FNDSTRKKIE KIRPLLIDGT
ASLSPGMMMG MFNMLSTVLG VSILNLGQKR HTKTTYWWDG LQSSDDFALI VNAPNHEGIQ AGVNRFYRTC KLLGINMSKK
KSYINRTGTF EFTSFFYRYG FVANFSMELP SFGVSGINES ADMSIGVTVI KNNMINNDLG PATAQMALQL FIKDYRYTYR
CHRGDTQIQT RRSFEIKKLW EQTHSKAGLL VSDGGPNLYN IRNLHIPEVC LKWELMDEDY QGRLCNPLNP FVNHKDIESV
NNAVIMPAHG PAKNMEYDAV ATTHSWIPKR NRSILNTSQR GILEDEQMYQ KCCNLFEKFF PSSSYRRPVG ISSMVEAMVS
RARIDARIDF ESGRIKKEEF TEIMKICSTI EELRRQK

PA

MEDFVRQCFN PMIVELAEKA MKEYGEDLKI ETNKFAAICT HLEVCFMYSD FHFIDEQGES IVVELGDPNA LLKHRFEIIE
GRDRTIAWTV INSICNTTGA EKPKFLPDLY DYKKNRFIEI GVTRREVHIY YLEKANKIKS EKTHIHIFSF TGEEMATKAD
YTLDEESRAR IKTRLFTIRQ EMASRGLWDS FRQSERGEET IEERFEITGT MRKLADQSLP PNFSSLENFR AYVDGFEPNG
YIEGKLSQMS KEVNARIEPF LKSTPRPLRL PDGPPCSQRS KFLLMDALKL SIEDPSHEGE GIPLYDAIKC MRTFFGWKEP
NVVKPHEKGI NPNYLLSWKQ VLAELQDIEN EEKIPRTKNM KKTSQLKWAL GENMAPEKVD FDDCKDVGDL KQYDSDEPEL
RSLASWIQNE FNKACELTDS SWIELDEIGE DAAPIEHIAS MRRNYFTAEV SHCRATEYIM KGVYINTALL NASCAAMDDF
QLIPMISKCR TKEGRRKTNL YGFIIKGRSH LRNDTDVVNF VSMEFSLTDP RLEPHKWEKY CVLEVGDMLL RSAIGHVSRP
MFLYVRTNGT SKIKMKWGME MRRCLLQSLQ QIESMIEAES SVKEKDMTKE FFENKSETWP VGESPKGVEE GSIGKVCRTL
LAKSVFNSLY ASPQLEGFSA ESRKLLLIVQ ALRDNLEPGT FDLGGLYEAI EECLINDPWV LLNASWFNSF LTHALR

HA

MKAFVLVLLY AFVATDADTI CIGYHANNST DTVDTIFEKN VAVTHSVNLL EDRHNGKLCK LKGIAPLQLG KCNITGWLLG
NPECDSLLPA RSWSYIVETP NSENGACYPG DFIDYEELRE QLSSVSSLER FEIFPKESSW PNHTFNGVTV SCSHRGKSSF
YRNLLWLTKK GDSYPKLTNS YVNNKGKEVL VLWGVHHPSS SDEQQSLYSN GNAYVSVASS NYNRRFTPEI AARPKVKDQH
GRMNYYWTLL EPGDTIIFEA TGNLIAPWYA FALSRGFESG IITSNASMHE CNTKCQTPQG SINSNLPFQN IHPVTIGECP
KYVRSTKLRM VTGLRNIPSI QYRGLFGAIA GFIEGGWTGM IDGWYGYHHQ NEQGSGYAAD QKSTQNAING ITNKVNSVIE
KMNTQFTAVG KEFNNLEKRM ENLNKKVDDG FLDIWTYNAE LLVLLENERT LDFHDLNVKN LYEKVKSQLK NNAKEIGNGC
FEFYHKCDNE CMESVRNGTY DYPKYSEESK LNREKIDGVK LESMGVYQIL AIYSTVASSL VLLVSLGAIS FWMCSNGSLQ
CRICI

NP

MATKGTKRSY EQMETDGERQ NATEIRASVG KMIDGIGRFY IQMCTELKLS DYEGRLIQNS LTIERMVLSA FDERRNKYLE
EHPSAGKDPK KTGGPIYRRV DGKWRRELIL YDKEEIRRIW RQANNGDDAT AGLTHMMIWH SNLNDATYQR TRALVRTGMD
PRMCSLMQGS TLPRRSGAAG AAVKGVGTMV MELIRMIKRG INDRNFWRGE NGRRTRIAYE RMCNILKGKF QTAAQRTMVD
QVRESRNPGN AEFEDLIFLA RSALILRGSV AHKSCLPACV YGSAVASGYD FEREGYSLVG IDPFRLLQNS QVYSLIRPNE
NPAHKSQLVW MACHSAAFED LRVSSFIRGT KVVPRGKLST RGVQIASNEN METMESSTLE LRSRYWAIRT RSGGNTNQQR
ASSGQISIQP TFSVQRNLPF DRPTIMAAFT GNTEGRTSDM RTEIIRLMES ARPEDVSFQG RGVFELSDEK ATSPIVPSFD
MSNEGSYFFG DNAEEYDN

NA

MNPNQKIITI GSICMVVGII SLILQIGNII SIWISHSIQT GNQNHTGICN QGSITYKVVA GQDSTSVILT GNSSLCPIRG
WAIHSKDNGI RIGSKGDVFV IREPFISCSH LECRTFFLTQ GALLNDKHSR GTFKDRSPYR ALMSCPVGEA PSPYNSRFES
VAWSASACHD GMGWLTIGIS GPDDGAVAVL KYNGIITETI KSWRKNILRT QESECTCVNG SCFTIMTDGP SDGLASYKIF
KIEKGKVTKS IELNAPNSHY EECSCYPDTG KVMCVCRDNW HGSNRPWVSF DQNLDYKIGY ICSGVFGDNP RPKDGTGSCG
PVSADGANGV KGFSYKYGNG VWIGRTKSDS SRHGFEMIWD PNGWTETDSR FSMRQDVVAM TDRSGYSGSF VQHPELTGLD
CMRPCFWVEL IRGLPEEDAI WTSGSIISFC GVNGDTVDWS WPDGAELPFT IDK

M1

MSLLTEVETY VLSIVPSGPL KAEIAQRLED VFAGKNTDLE VLMEWLKTRP ILSPLTKGIL GFVFTLTVPS ERGLQRRRFV
QNALNGNGDP NNMDKAVKLY RKLKREITFH GAKEIALSYS AGALASCMGL IYNRMGAVTT EVAFGLVCAT CEQIADSQHR
SHRQMVTTTN PLIRHENRMV LASTTAKAME QMAGSSEQAA EAMDIASQAR QMVQAMRTIG THPSSSAGLK DDLLENLQAY
QKRMGVQMQR FK

M2

MSLLTEVETP IRNEWGCRCN DSSDPLVIAA NIIEILHLIL WILDRLFFKC IYRRFKYGLK RGPSTEGVPE SMREEYRKEQ
QNAVDVDDGH FVNIELE

NS1

MDPNTVSSFQ VDCFLWHVRK RVADQELGDA PFLDRLRRDQ KSLRGRGSTL GLDIETATRA GKQIVERILK EESDEALKMT
MASVPASRYL TDMTLEEMSR HWFMLMPKQK VAGPLCIRMD QAIMDKNIIL KANFSVIFDR LETLILLRAF TEEGTIVGEI
SPLPSLPGHT DEDVKNAVGV LIGGLEWNNN TVRVSETLQR FAWRSSNENG RPPLTPKQKR KMAGTIRSEV

NEP

MDPNTVSSFQ DILMRMSKMQ LGSSSEDLNG IITQFESLKL YRDSLGEAVM RMGDLHSLQN RNGKWREQLG QKFEEIRWLI
EEVRHRLKIT ENSFEQITFM QALQLLLEVE QEIRTFSFQL I

X-181

PB2

```
MERIKELRNL MSQSRTREIL TKTTVDHMAI IKKYTSGRQE KNPALRMKWM MAMKYPITAD KRITEMIPER NEQGQTLWSK
MNDAGSDRVM VSPLAVTWWN RNGPITNTVH YPKIYKTYFE RVERLKHGTF GPVHFRNQVK IRRRVDINPG HADLSAKEAQ
DVIMEVVFPN EVGARILTSE SQLTITKEKK EELQDCKISP LMVAYMLERE LVRKTRFLPV AGGTSSVYIE VLHLTQGTCW
EQMYTPGGEV RNDDVDQSLI IAARNIVRRA AVSADPLASL LEMCHSTQIG GIRMVDILRQ NPTEEQAVDI CKAAMGLRIS
SSFSFGGFTF KRTSGSSVKR EEEVLTGNLQ TLKIRVHEGY EEFTMVGRRA TAILRKATRR LIQLIVSGRD EQSIAEAIIV
AMVFSQEDCM IKAVRGDLNF VNRANQRLNP MHQLLRHFQK DAKVLFQNWG VEPIDNVMGM IGILPDMTPS IEMSMRGVRI
SKMGVDEYSS TERVVVSIDR FLRIRDQRGN VLLSPEEVSE TQGTEKLTIT YSSSMMWEIN GPESVLVNTY QWIIRNWETV
KIQWSQNPTM LYNKMEFEPF QSLVPKAIRG QYSGFVRTLF QQMRDVLGTF DTAQIIKLLP FAAAPPKQSR MQFSSFTVNV
RGSGMRILVR GNSPVFNYNK ATKRLTVLGK DAGTLTEDPD EGTAGVESAV LRGFLILGKE NKRYGPALSI NELSNLAKGE
KANVLIGQGD VVLVMKRKRD SSILTDSQTA TKRIRMAIN
```

PB1

```
MDVNPTLLFL KIPAQNAIST TFPYTGDPPY SHGTGTGYTM DTVNRTHQYS EKGKWTTNTE TGAPQLNPID GPLPEDNEPS
GYAQTDCVLE AMAFLEESHP GIFENSCLET MEVVQQTRVD KLTQGRQTYD WTLNRNQPAA TALANTIEVF RSNGLTANES
GRLIDFLKDV MESMNKEEIE ITTHFQRKRR VRDNMTKKMV TQRTIGKKKQ RLNKRGYLIR ALTLNTMTKD AERGKLKRRA
IATPGMQIRG FVYFVETLAR SICEKLEQSG LPVGGNEKKA KLANVVRKMM TNSQDTEISF TITGDNTKWN ENQNPRMFLA
MITYITRNQP EWFRNILSMA PIMFSNKMAR LGKGYMFESK RMKIRTQIPA EMLASIDLKY FNESTKKKIE KIRPLLIDGT
ASLSPGMMMG MFNMLSTVLG VSILNLGQKK YTKTIYWWDG LQSSDDFALI VNAPNHEGIQ AGVDRFYRTC KLVGINMSKK
KSYINKTGTF EFTSFFYRYG FVANFSMELP SFGVSGVNES ADMSIGVTVI KNNMINNDLG PATAQMALQL FIKDYRYTYR
CHRGDTQIQT RRSFELKKLW DQTQSKVGLL VSDGGPNLYN IRNLHIPEVC LKWELMDDDY RGRLCNPLNP FVSHKEIDSV
NNAVVMPAHG PAKSMEYDAV ATTHSWIPKR NRSILNTSQR GILEDEQMYQ KCCNLFEKFF PSSSYRRPVG ISSMVEAMVS
RARIDARVDF ESGRIKKEEF SEIMKICSTI EELRRQK
```

PA

```
MEDFVRQCFN PMIVELAEKT MKEYGEDLKI ETNKFAAICT HLEVCFMYSD FHFINEQGES IIVELGDPNA LLKHRFEIIE
GRDRTMAWTV VNSICNTTGA EKPKFLPDLY DYKENRFIEI GVTRREVHIY YLEKANKIKS EKTHIHIFSF TGEEMATKAD
YTLDEESRAR IKTRLFTIRQ EMASRGLWDS FRQSERGEET IEERFEITGT MRKLADQSLP PNFSSLENFR AYVDGFEPNG
YIEGKLSQMS KEVNARIEPF LKTTPRPLRL PNGPPCSQRS KFLLMDALKL SIEDPSHEGE GIPLYDAIKC MRTFFGWKEP
NVVKPHEKGI NPNYLLSWKQ VLAELQDIEN EEKIPKTKNM KKTSQLKWAL GENMAPEKVD FDDCKDVGDL KQYDSDEPEL
RSLASWIQNE FNKACELTDS SWIELDEIGE DVAPIEHIAS MRRNYFTSEV SHCRATEYIM KGVYINTALL NASCAAMDDF
QLIPMISKCR TKEGRRKTNL YGFIIKGRSH LRNDTDVVNF VSMEFSLTDP RLEPHKWEKY CVLEIGDMLI RSAIGQVSRP
MFLYVRTNGT SKIKMKWGME MRRCLLQSLQ QIESMIEAES SVKEKDMTKE FFENKSETWP IGESPKGVEE SSIGKVCRTL
LAKSVFNSLY ASPQLEGFSA ESRKLLLIVQ ALRDNLEPGT FDLGGLYEAI EECLINDPWV LLNASWFNSF LTHALS
```

HA

```
MKAILVVLLY TFATANADTL CIGYHANNST DTVDTVLEKN VTVTHSVNLL EDKHNGKLCK LRGVAPLHLG KCNIAGWILG
NPECESLSTA SSWSYIVETP SSDNGTCYPG DFIDYEELRE QLSSVSSFER FEIFPKTSSW PNHDSNKGVT AACPHAGAKS
FYKNLIWLVK KGNSYPKLSK SYINDKGKEV LVLWGIHHPS TSADQQSLYQ NADAYVFVGS SRYSKKFKPE IAIRPKVRDQ
EGRMNYYWTL VEPGDKITFE ATGNLVVPRY AFAMERNAGS GIIISDTPVH DCNTTCQTPK GAINTSLPFQ NIHPITIGKC
PKYVKSTKLR LATGLRNIPS IQSRGLFGAI AGFIEGGWTG MVDGWYGYHH QNEQGSGYAA DLKSTQNAID EITNKVNSVI
EKMNTQFTAV GKEFNHLEKR IENLNKKVDD GFLDIWTYNA ELLVLLENER TLDYHDSNVK NLYEKVRSQL KNNAKEIGNG
CFEFYHKCDN TCMESVKNGT YDYPKYSEEA KLNREEIDGV KLESTRIYQI LAIYSTVASS LVLVVSLGAI SFWMCSNGSL
QCRICI
```

NP

```
MASQGTKRSY EQMETDGERQ NATEIRASVG KMIGGIGRFY IQMCTELKLS DYEGRLIQNS LTIERMVLSA FDERRNKYLE
EHPSAGKDPK KTGGPIYRRV NGKWMRELIL YDKEEIRRIW RQANNGDDAT AGLTHMMIWH SNLNDATYQR TRALVRTGMD
PRMCSLMQGS TLPRRSGAAG AAVKGVGTMV MELVRMIKRG INDRNFWRGE NGRKTRIAYE RMCNILKGKF QTAAQKAMMD
QVRESRNPGN AEFEDLTFLA RSALILRGSV AHKSCLPACV YGPAVASGYD FEREGYSLVG IDPFRLLQNS QVYSLIRPNE
NPAHKSQLVW MACHSAAFED LRVLSFIKGT KVLPRGKLST RGVQIASNEN METMESSTLE LRSRYWAIRT RSGGNTNQQR
ASAGQISIQP TFSVQRNLPF DRTTIMAAFN GNTEGRTSDM RTEIIRMMES ARPEDVSFQG RGVFELSDEK AASPIVPSFD
MSNEGSYFFG DNAEEYDN
```

NA

```
MNPNQKIITI GSVCMTIGMA NLILQIGNII SIWISHSIQL GNQNQIETCN QSVITYENNT WVNQTYVNIS NTNFAAGQSV
VSVKLAGNSS LCPVSGWAIY SKDNSVRIGS KGDVFVIREP FISCSPLECR TFFLTQGALL NDKHSNGTIK DRSPYRTLMS
CPIGEVPSPY NSRFESVAWS ASACHDGINW LTIGISGPDN GAVAVLKYNG IITDTIKSWR NNILRTQESE CACVNGSCFT
VMTDGPSNGQ ASYKIFRIEK GKIVKSVEMN APNYHYEECS CYPDSSEITC VCRDNWHGSN RPWVSFNQNL EYQIGYICSG
IFGDNPRPND KTGSCGPVSS NGANGVKGFS FKYGNGVWIG RTKSISSRNG FEMIWDPNGW TGTDNNFSIK QDIVGINEWS
GYSGSFVQHP ELTGLDCIRP CFWVELIRGR PKENTIWTSG SSISFCGVNS DTVGWSWPDG AELPFTIDK
```

M1

```
MSLLTEVETY VLSIIPSGPL KAEIAQRLED VFAGKNTDLE VLMEWLKTRP ILSPLTKGIL GFVFTLTVPS ERGLQRRRFV
QNALNGNGDP NNMDKAVKLY RKLKREITFH GAKEISLSYS AGALASCMGL IYNRMGAVTT EVAFGLVCAT CEQIADSQHR
SHRQMVTTTN PLIRHENRMV LASTTAKAME QMAGSSEQAA EAMEVASQAR QMVQAMRTIG THPSSSAGLK NDLLENLQAY
QKRMGVQMQR FK
```

M2

```
MSLLTEVETP IRNEWGCRCN GSSDPLTIAA NIIGILHLTL WILDRLFFKC IYRRFKYGLK GGPSTEGVPK SMREEYRKEQ
QSAVDADDGH FVSIELE
```

NS1

```
MDPNTVSSFQ VDCFLWHVRK RVADQELGDA PFLDRLRRDQ KSLRGRGSTL GLDIKTATRA GKQIVERILK EESDEALKMT
MASVPASRYL TDMTLEEMSR DWSMLIPKQK VAGPLCIRMD QAIMDKNIIL KANFSVIFDR LETLILLRAF TEEGAIVGEI
SPLPSLPGHT AEDVKNAVGV LIGGLEWNDN TVRVSETLQR FAWRSSNENG RPPLTPKQKR EMAGTIRSEV
```

NEP

```
MDPNTVSSFQ DILLRMSKMQ LESSSEDLNG MITQFESLKL YRDSLGEAVM RMGDLHSLQN RNEKWREQLG QKFEEIRWLI
EEVRHKLKIT ENSFEQITFM QALHLLLEVE QEIRTFSFQL I
```

X-187

PB2

MERIKELRNL MSQSRTREIL TKTTVDHMAI IKKYTSGRQE KNPALRMKWM MAMKYPITAD KRITEMIPER NEQGQTLWSK
MNDAGSDRVM VSPLAVTWWN RNGPITNTVH YPKIYKTYFE RVERLKHGTF GPVHFRNQVK IRRRVDINPG HADLSAKEAQ
DVIMEVVFPN EVGARILTSE SQLTITKEKK EELQDCKISP LMVAYMLERE LVRKTRFLPV AGGTSSVYIE VLHLTQGTCW
EQMYTPGGEV RNDDVDQSLI IAARNIVRRA AVSADPLASL LEMCHSTQIG GIRMVDILRQ NPTEEQAVDI CKAAMGLRIS
SSFSFGGFTF KRTSGSSVKR EEEVLTGNLQ TLKIRVHEGY EEFTMVGRRA TAILRKATRR LIQLIVSGRD EQSIAEAIIV
AMVFSQEDCM IKAVRGDLNF VNRANQRLNP MHQLLRHFQK DAKVLFQNWG VEPIDNVMGM IGILPDMTPS IEMSMRGVRI
SKMGVDEYSS TERVVVSIDR FLRIRDQRGN VLLSPEEVSE TQGTEKLTIT YSSSMMWEIN GPESVLVNTY QWIIRNWETV
KIQWSQNPTM LYNKMEFEPF QSLVPKAIRG QYSGFVRTLF QQMRDVLGTF DTAQIIKLLP FAAAPKQSR MQFSSFTVNV
RGSGMRILVR GNSPVFNYNK ATKRLTVLGK DAGTLTEDPD EGTAGVESAV LRGFLILGKE NKRYGPALSI NELSNLAKGE
KANVLIGQGD VVLVMKRKRD SSILTDSQTA TKRIRMAIN

PB1

MDVNPTLLFL KVPAQNAIST TFPYTGDPPY SHGTGTGYTM DTVNRTHQYS EKGRWTTNTE TGAPQLNPID GPLPEDNEPS
GYAQTDCVLE AMAFLEESHP GIFENSCIET MEVVQQTRVD KLTQGRQTYD WTLNRNQPAA TALANTIEVF RSNGLTANES
GRLIDFLKDV MESMNKEEMG ITTHFQRKRR VRDNMTKKMI TQRTMGKKKQ RLNKRSYLIR ALTLNTMTKD AERGKLKRRA
IATPGMQIRG FVYFVETLAR SICEKLEQSG LPVGGNEKKA KLANVVRKMM TNSQDTELSF TITGDNTKWN ENQNPRMFLA
MITYMTRNQP EWFRNVLSIA PIMFSNKMAR LGKGYMFESK SMKLRTQIPA EMLASIDLKY FNDSTRKKIE KIRSLLIEGT
ASLSPGMMMG MFNMLSTVLG VSILNLGQKR YTKTTYWWDG LQSSDDFALI VNAPNHEGIQ AGVDRFYRTC KLLGINMSKK
KSYINRTGTF EFTSFFYRYG FVANFSMELP SFGVSGINES ADMSIGVTVI KNNMINNDLG PATAQMALQL FIKDYRYTYR
CHRGDTQIQT RRSFEIKKLW EQTRSKAGLL VSDGGPNLYN IRNLHIPEVC LKWELMDEDY QGRLCNPLNP FVSHKEIESM
NNAVMMPAHG PAKNMEYDAV ATTHSWIPKR NRSILNTSQR GVLEDEQMYQ RCCNLFEKFF PSSSYRRPVG ISSMVEAMVS
RARIDARIDF ESGRIKKEEF TEIMKICSTI EELRRQK

PA

MEDFVRQCFN PMIVELAEKT MKEYGEDLKI ETNKFAAICT HLEVCFMYSD FHFINEQGES IIVELGDPNA LLKHRFEIIE
GRDRTMAWTV VNSICNTTGA EKPKFLPDLY DYKENRFIEI GVTRREVHIY YLEKANKIKS EKTHIHIFSF TGEEMATKAD
YTLDEESRAR IKTRLFTIRQ EMASRGLWDS FRQSERGEET IEERFEITGT MRKLADQSLP PNFSSLENFR AYVDGFEPNG
YIEGKLSQMS KEVNARIEPF LKTTPRPLRL PNGPPCSQRS KFLLMDALKL SIEDPSHEGE GIPLYDAIKC MRTFFGWKEP
NVVKPHEKGI NPNYLLSWKQ VLAELQDIEN EEKIPKTKNM KKTSQLKWAL GENMAPEKVD FDDCKDVGDL KQYDSDEPEL
RSLASWIQNE FNKACELTDS SWIELDEIGE DVAPIEHIAS MRRNYFTSEV SHCRATEYIM KGVYINTALL NASCAAMDDF
QLIPMISKCR TKEGRRKTNL YGFIIKGRSH LRNDTDVVNF VSMEFSLTDP RLEPHKWEKY CVLEIGDMLI RSAIGQVSRP
MFLYVRTNGT SKIKMKWGME MRRCLLQSLQ QIESMIEAES SVKEKDMTKE FFENKSETWP IGESPKGVEE SSIGKVCRTL
LAKSVFNSLY ASPQLEGFSA ESRKLLLIVQ ALRDNLEPGT FDLGGLYEAI EECLINDPWV LLNASWFNSF LTHALS

HA

MKTIIALSYI LCLVFAQKLP GNDNSTATLC LGHHAVPNGT IVKTITNDQI EVTNATELVQ NSSTGEICDS PHQILDGKNC
TLIDALLGDP QCDGFQNKKW DLFVERSKAY SNCYPYDVPD YASLRSLVAS SGTLEFNNES FNWTGVTQNG TSSACIRRSK
NSFFSRLNWL TNLNFKYPAL NVTMPNNEQF DKLYIWGVHH PVTDKDQIFL YAQASGRITV STKRSQQTVI PNIGSRPRVR
NIPSRISIYW TIVKPGDILL INSTGNLIAP RGYFKMQSGK SSIMRSDAPI GKCNSECITP NGSIPNDKPF QNVNRITYGA
CPRYVKQNTL KLATGMRNVP EKQTRGIFGA IAGFIENGWE GMVDGWYGFR HQNSEGRGQA ADLKSTQAAI DQINGKLNRL
IGKTNEKFHQ IEKEFSEVEG RIQDLEKYVE DTKIDLWSYN AELLVALENQ HTIDLTDSEM NKLFEKTKKQ LRENAEDMGN
GCFKIYHKCD NACIGSIRNG TYDHNVYRDE ALNNRFQIKG VELKSGYKDW ILWISFAISC FLLCVALLGF IMWACQKGNI
RCNICI

NP

MASQGTKRSY EQMETDGERQ NATEIRASVG KMIGGIGRFY IQMCTELKLS DYEGRLIQNS LTIERMVLSA FDERRNKYLE
EHPSAGKDPK KTGGPIYRRV NGKWMRELIL YDKEEIRRIW RQANNGDDAT AGLTHMMIWH SNLNDATYQR TRALVRTGMD
PRMCSLMQGS TLPRRSGAAG AAVKGVGTMV MELVRMIKRG INDRNFWRGE NGRKTRIAYE RMCNILKGKF QTAAQKAMMD
QVRESRNPGN AEFEDLTFLA RSALILRGSV AHKSCLPACV YGPAVASGYD FEREGYSLVG IDPFRLLQNS QVYSLIRPNE
NPAHKSQLVW MACHSAAFED LRVLSFIKGT KVLPRGKLST RGVQIASNEN METMESSTLE LRSRYWAIRT RSGGNTNQQR
ASAGQISIQP TFSVQRNLPF DRTTIMAAFN GNTEGRTSDM RTEIIRMMES ARPEDVSFQG RGVFELSDEK AASPIVPSFD
MSNEGSYFFG DNAEEYDN

NA

MNPNQKIITI GSVSLTISTI CFFMQIAILI TTVTLHFKQY EFNSPPNNQV MLCEPTIIER NITEIVYLTN TTIEKEICPK
LAEYRNWSKP QCDITGFAPF SKDNSIRLSA GGDIWVTREP YVSCDPDKCY QFALGQGTTL NNVHSNNTVR DRTPYRTLLM
NELGVPFHLG TKQVCIAWSS SSCHDGKAWL HVCITGDDKN ATASFIYNGR LVDSVVSWSK EILRTQESEC VCINGTCTVV
MTDGSASGKA DTKILFIEEG KIVHTSTLSG SAQHVEECSC YPRYPGVRCV CRDNWKGSNR PIVDINIKDH SIVSSYVCSG
LVGDTPRKND SSSSSHCLDP NNEEGGHGVK GWAFDDGNDV WMGRTISEKS RLGYETFKVI EGWSNPKSKL QINRQVIVDR
GNRSGYSGIF SVEGKSCINR CFYVELIRGR KEETEVLWTS NSIVVFCGTS GTYGTGSWPD GADINLMPI

M1

MSLLTEVETY VLSIIPSGPL KAEIAQRLED VFAGKNTDLE VLMEWLKTRP ILSPLTKGIL GFVFTLTVPS ERGLQRRRFV
QNALNGNGDP NNMDKAVKLY RKLKREITFH GAKEISLSYS AGALASCMGL IYNRMGAVTT EVAFGLVCAT CEQIADSQHR
SHRQMVTTTN PLIRHENRMV LASTTAKAME QMAGSSEQAA EAMEVASQAR QMVQAMRTIG THPSSSAGLK NDLLENLQAY
QKRMGVQMQR FK

M2

MSLLTEVETP IRNEWGCRCN GSSDPLTIAA NIIGILHLTL WILDRLFFKC IYRRFKYGLK GGPSTEGVPK SMREEYRKEQ
QSAVDADDGH FVSIELE

NS1

MDPNTVSSFQ VDCFLWHVRK RVADQELGDA PFLDRLRRDQ KSLRGRGSTL GLDIKTATRA GKQIVERILK EESDEALKMT
MASVPASRYL TDMTLEEMSR DWSMLIPKQK VAGPLCIRMD QAIMDKNIIL KANFSVIFDR LETLILLRAF TEEGAIVGEI
SPLPSLPGHT AEDVKNAVGV LIGGLEWNDN TVRVSETLQR FAWRSSNENG RPPLTPKQKR EMAGTIRSEV

NEP

MDPNTVSSFQ DILLRMSKMQ LESSSEDLNG MITQFESLKL YRDSLGEAVM RMGDLHSLQN RNEKWREQLG QKFEEIRWLI
EEVRHKLKIT ENSFEQITFM QALHLLLEVE QEIRTFSFQL I

NIB-74xp (egg-grown)

PB2

MERIKELRNL MSQSRTREIL TKTTVDHMAI IKKYTSGRQE KNPALRMKWM MAMKYPITAD KRITEMIPER NEQGQTLWSK
MNDAGSDRVM VSPLAVTWWN RNGPITNTVH YPKIYKTYFE RVERLKHGTF GPVHFRNQVK IRRRVDINPG HADLSAKEAQ
DVIMEVVFPN EVGARILTSE SQLTITKEKK EELQDCKISP LMVAYMLERE LVRKTRFLPV AGGTSSVYIE VLHLTQGTCW
EQMYTPGGEV RNDDVDQSLI IAARNIVRRA AVSADPLASL LEMCHSTQIG GIRMVDILRQ NPTEEQAVDI CKAAMGLRIS
SSFSFGGFTF KRTSGSSVKR EEEVLTGNLQ TLKIRVHEGY EEFTMVGRRA TAILRKATRR LIQLIVSGRD EQSIAEAIIV
AMVFSQEDCM IKAVRGDLNF VNRANQRLNP MHQLLRHFQK DAKVLFQNWG VEPIDNVMGM IGILPDMTPS IEMSMRGVRI
SKMGVDEYSS TERVVVSIDR FLRIRDQRGN VLLSPEEVSE TQGTEKLTIT YSSSMMWEIN GPESVLVNTY QWIIRNWETV
KIQWSQNPTM LYNKMEFEPF QSLVPKAIRG QYSGFVRTLF QQMRDVLGTF DTAQIIKLLP FAAAPPKQSR MQFSSFTVNV
RGSGMRILVR GNSPVFNYNK ATKRLTVLGK DAGTLTEDPD EGTAGVESAV LRGFLILGKE NKRYGPALSI NELSNLAKGE
KANVLIGQGD VVLVMKRKRD SSILTDSQTA TKRIRMAIN

PB1

MDVNPTLLFL KVPAQNAIST TFPYTGDPPY SHGTGTGYTM DTVNRTHQYS EKGRWTTNTE TGAPQLNPID GPLPEDNEPS
GYAQTDCVLE AMAFLEESHP GIFENSCIET MEVVQQTRVD KLTQGRQTYD WTLNRNQPAA TALANTIEVF RSNGLTANES
GRLIDFLKDV MESMNKEEMG ITTHFQRKRR VRDNMTKKMI TQRTMGKKKQ RLNKRSYLIR ALTLNTMTKD AERGKLKRRA
IATPGMQIRG FVYFVETLAR SICEKLEQSG LPVGGNEKKA KLANVVRKMM TNSQDTELSF TITGDNTKWN ENQNPRMFLA
MITYMTRNQP EWFRNVLSIA PIMFSNKMAR LGKGYMFESK SMKLRTQIPA EMLASIDLKY FNDSTRKKIE KIRSLLIEGT
ASLSPGMMMG MFNMLSTVLG VSILNLGQKR YTKTTYWWDG LQSSDDFALI VNAPNHEGIQ AGVDRFYRTC KLLGINMSKK
KSYINRTGTF EFTSFFYRYG FVANFSMELP SFGVSGINES ADMSIGVTVI KNNMINNDLG PATAQMALQL FIKDYRYTYR
CHRGDTQIQT RRSFEIKKLW EQTRSKAGLL VSDGGPNLYN IRNLHIPEVC LKWELMDEDY QGRLCNPLNP FVSHKEIESM
NNAVMMPAHG PAKNMEYDAV ATTHSWIPKR NRSILNTSQR GVLEDEQMYQ RCCNLFEKFF PSSSYRRPVG ISSMVEAMVS
RARIDARIDF ESGRIKKEEF TEIMKICSTI EELRRQK

PA

MEDFVRQCFN PMIVELAEKT MKEYGEDLKI ETNKFAAICT HLEVCFMYSD FHFINEQGES IIVELGDPNA LLKHRFEIIE
GRDRTMAWTV VNSICNTTGA EKPKFLPDLY DYKENRFIEI GVTRREVHIY YLEKANKIKS EKTHIHIFSF TGEEMATKAD
YTLDEESRAR IKTRLFTIRQ EMASRGLWDS FRQSERGEET IEERFEITGT MRKLADQSLP PNFSSLENFR AYVDGFEPNG
YIEGKLSQMS KEVNARIEPF LKTTPRPLRL PNGPPCSQRS KFLLMDALKL SIEDPSHEGE GIPLYDAIKC MRTFFGWKEP
NVVKPHEKGI NPNYLLSWKQ VLAELQDIEN EEKIPKTKNM KKTSQLKWAL GENMAPEKVD FDDCKDVGDL KQYDSDEPEL
RSLASWIQNE FNKACELTDS SWIELDEIGE DVAPIEHIAS MRRNYFTSEV SHCRATEYIM KGVYINTALL NASCAAMDDF
QLIPMISKCR TKEGRRKTNL YGFIIKGRSH LRNDTDVVNF VSMEFSLTDP RLEPHKWEKY CVLEIGDMLI RSAIGQVSRP
MFLYVRTNGT SKIKMKWGME MRRCLLQSLQ QIESMIEAES SVKEKDMTKE FFENKSETWP IGESPKGVEE SSIGKVCRTL
LAKSVFNSLY ASPQLEGFSA ESRKLLLIVQ ALRDNLEPGT FDLGGLYEAI EECLINDPWV LLNASWFNSF LTHALS

HA

MKAILVVLLH TFATANADTL CIGYHANNST DTVDTVLEKN VTVTHSVNLL EDKHNGKLCK LRGVAPLHLG KCNIAGWILG
NPECESLSTA SSWSYIVETS SSDNGTCYPG NFIDYEELRE QLSSVSSFER FEIFPKTSSW PDHDSNKGVT AACPHAGAKS
FYKNLIWLVK KGNSYPTLSK SYINDKGKEV LVLWGIHHPS TSADQQSLYQ NADAYVFVGT SRYSKKFKPE IAIRPKVRNQ
EGRMNYYWTL VEPGDKITFE ATGNLVAPRY AFAMERNAGS GIIISDTPVH DCNTTCQTPK GAINTSLPFQ NIHPITIGKC
PKYVKSTKLR LATGLRNVPS IQSRGLFGAI AGFIEGGWTG MVDGWYGYHH QNEQGSGYAA DLKSTQNAID KITNKVNSVI
EKMNTQFTAV GKEFNHLEKR IENLNKKVDD GFLDIWTYNA ELLVLLENER TLDYHDSNVK NLYEKVRSQL KNNAKEIGNG
CFEFYHKCDN TCMESVKNGT YDYPKYSEEA KLNREEIDGV KLESTRIYQI LAIYSTVASS LVLVVSLGAI SFWMCSNGSL
QCRICI

NP

MASQGTKRSY EQMETDGERQ NATEIRASVG KMIGGIGRFY IQMCTELKLS DYEGRLIQNS LTIERMVLSA FDERRNKYLE
EHPSAGKDPK KTGGPIYRRV NGKWMRELIL YDKEEIRRIW RQANNGDDAT AGLTHMMIWH SNLNDATYQR TRALVRTGMD
PRMCSLMQGS TLPRRSGAAG AAVKGVGTMV MELVRMIKRG INDRNFWRGE NGRKTRIAYE RMCNILKGKF QTAAQKAMMD
QVRESRNPGN AEFEDLTFLA RSALILRGSV AHKSCLPACV YGPAVASGYD FEREGYSLVG IDPFRLLQNS QVYSLIRPNE
NPAHKSQLVW MACHSAAFED LRVLSFIKGT KVLPRGKLST RGVQIASNEN METMESSTLE LRSRYWAIRT RSGGNTNQQR
ASAGQISIQP TFSVQRNLPF DRTTIMAAFN GNTEGRTSDM RTEIIRMMES ARPEDVSFQG RGVFELSDEK AASPIVPSFD
MSNEGSYFFG DNAEEYDN

NA

MNPNQKIITI GSVCITIGMA NLILQIGNII SIWISHSIQL GNQNQIETCN QSVITYENNT WVNQTYVNIS NTNFAAGQSV
VSVKLAGNSS LCPVSGWAIY SKDNSIRIGS KGDVFVIREP FISCSPLECR TFFLTQGALL NDKHSNGTIK DRSPYRTLMS
CPIGEVPSPY NSRFESVAWS ASACHDGISW LTIGISGPDN GAVAVLKYNG IITDTIKSWR NNILRTQESE CACVNGSCFT
VMTDGPSDGQ ASYKIFRIEK GKIVKSVEMN APNYHYEECS CYPDSSEITC VCRDNWHGSN RPWVSFNQNL EYQIGYICSG
IFGDNPRPND KTGSCGPVSS NGANGVKGFS FKYGNGVWIG RTKSISSRNG FEIIWDPNGW TGTDNNFSIK QDIVGINEWS
GYSGSFVQHP ELTGLDCIRP CFWVELIRGR PKENTIWTSG SSISFCGVNS DTVGWSWPDG AELPFTIDK

M1

MSLLTEVETY VLSIIPSGPL KAEIAQRLED VFAGKNTDLE VLMEWLKTRP ILSPLTKGIL GFVFTLTVPS ERGLQRRRFV
QNALNGNGDP NNMDKAVKLY RKLKREITFH GAKEISLSYS AGALASCMGL IYNRMGAVTT EVAFGLVCAT CEQIADSQHR
SHRQMVTTTN PLIRHENRMV LASTTAKAME QMAGSSEQAA EAMEVASQAR QMVQAMRTIG THPSSSAGLK NDLLENLQAY
QKRMGVQMQR FK

M2

MSLLTEVETP IRNEWGCRCN GSSDPLTIAA NIIGILHLTL WILDRLFFKC IYRRFKYGLK GGPSTEGVPK SMREEYRKEQ
QSAVDADDGH FVSIELE

NS1

MDPNTVSSFQ VDCFLWHVRK RVADQELGDA PFLDRLRRDQ KSLRGRGSTL GLDIKTATRA GKQIVERILK EESDEALKMT
MASVPASRYL TDMTLEEMSR DWSMLIPKQK VAGPLCIRMD QAIMDKNIIL KANFSVIFDR LETLILLRAF TEEGAIVGEI
SPLPSLPGHT AEDVKNAVGV LIGGLEWNDN TVRVSETLQR FAWRSSNENG RPPLTPKQKR EMAGTIRSEV

NEP

MDPNTVSSFQ DILLRMSKMQ LESSSEDLNG MITQFESLKL YRDSLGEAVM RMGDLHSLQN RNEKWREQLG QKFEEIRWLI
EEVRHKLKIT ENSFEQITFM QALHLLLEVE QEIRTFSFQL I

NIB-74xp (MDCK-grown)

PB2

MERIKELRNL MSQSRTREIL TKTTVDHMAI IKKYTSGRQE KNPALRMKWM MAMKYPITAD KRITEMIPER NEQGQTLWSK
MNDAGSDRVM VSPLAVTWWN RNGPITNTVH YPKIYKTYFE RVERLKHGTF GPVHFRNQVK IRRRVDINPG HADLSAKEAQ
DVIMEVVFPN EVGARILTSE SQLTITKEKK EELQDCKISP LMVAYMLERE LVRKTRFLPV AGGTSSVYIE VLHLTQGTCW
EQMYTPGGEV RNDDVDQSLI IAARNIVRRA AVSADPLASL LEMCHSTQIG GIRMVDILRQ NPTEEQAVDI CKAAMGLRIS
SSFSFGGFTF KRTSGSSVKR EEEVLTGNLQ TLKIRVHEGY EEFTMVGRRA TAILRKATRR LIQLIVSGRD EQSIAEAIIV
AMVFSQEDCM IKAVRGDLNF VNRANQRLNP MHQLLRHFQK DAKVLFQNWG VEPIDNVMGM IGILPDMTPS IEMSMRGVRI
SKMGVDEYSS TERVVVSIDR FLRIRDQRGN VLLSPEEVSE TQGTEKLTIT YSSSMMWEIN GPESVLVNTY QWIIRNWETV
KIQWSQNPTM LYNKMEFEPF QSLVPKAIRG QYSGFVRTLF QQMRDVLGTF DTAQIIKLLP FAAAPPKQSR MQFSSFTVNV
RGSGMRILVR GNSPVFNYNK ATKRLTVLGK DAGTLTEDPD EGTAGVESAV LRGFLILGKE NKRYGPALSI NELSNLAKGE
KANVLIGQGD VVLVMKRKRD SSILTDSQTA TKRIRMAIN

PB1

MDVNPTLLFL KVPAQNAIST TFPYTGDPPY SHGTGTGYTM DTVNRTHQYS EKGRWTTNTE TGAPQLNPID GPLPEDNEPS
GYAQTDCVLE AMAFLEESHP GIFENSCIET MEVVQQTRVD KLTQGRQTYD WTLNRNQPAA TALANTIEVF RSNGLTANES
GRLIDFLKDV MESMNKEEMG ITTHFQRKRR VRDNMTKKMI TQRTMGKKKQ RLNKRSYLIR ALTLNTMTKD AERGKLKRRA
IATPGMQIRG FVYFVETLAR SICEKLEQSG LPVGGNEKKA KLANVVRKMM TNSQDTELSF TITGDNTKWN ENQNPRMFLA
MITYMTRNQP EWFRNVLSIA PIMFSNKMAR LGKGYMFESK SMKLRTQIPA EMLASIDLKY FNDSTRKKIE KIRSLLIEGT
ASLSPGMMMG MFNMLSTVLG VSILNLGQKR YTKTTYWWDG LQSSDDFALI VNAPNHEGIQ AGVDRFYRTC KLLGINMSKK
KSYINRTGTF EFTSFFYRYG FVANFSMELP SFGVSGINES ADMSIGVTVI KNNMINNDLG PATAQMALQL FIKDYRYTYR
CHRGDTQIQT RRSFEIKKLW EQTRSKAGLL VSDGGPNLYN IRNLHIPEVC LKWELMDEDY QGRLCNPLNP FVSHKEIESM
NNAVMMPAHG PAKNMEYDAV ATTHSWIPKR NRSILNTSQR GVLEDEQMYQ RCCNLFEKFF PSSSYRRPVG ISSMVEAMVS
RARIDARIDF ESGRIKKEEF TEIMKICSTI EELRRQK

PA

MEDFVRQCFN PMIVELAEKT MKEYGEDLKI ETNKFAAICT HLEVCFMYSD FHFINEQGES IIVELGDPNA LLKHRFEIIE
GRDRTMAWTV VNSICNTTGA EKPKFLPDLY DYKENRFIEI GVTRREVHIY YLEKANKIKS EKTHIHIFSF TGEEMATKAD
YTLDEESRAR IKTRLFTIRQ EMASRGLWDS FRQSERGEET IEERFEITGT MRKLADQSLP PNFSSLENFR AYVDGFEPNG
YIEGKLSQMS KEVNARIEPF LKTTPRPLRL PNGPPCSQRS KFLLMDALKL SIEDPSHEGE GIPLYDAIKC MRTFFGWKEP
NVVKPHEKGI NPNYLLSWKQ VLAELQDIEN EEKIPKTKNM KKTSQLKWAL GENMAPEKVD FDDCKDVGDL KQYDSDEPEL
RSLASWIQNE FNKACELTDS SWIELDEIGE DVAPIEHIAS MRRNYFTSEV SHCRATEYIM KGVYINTALL NASCAAMDDF
QLIPMISKCR TKEGRRKTNL YGFIIKGRSH LRNDTDVVNF VSMEFSLTDP RLEPHKWEKY CVLEIGDMLI RSAIGQVSRP
MFLYVRTNGT SKIKMKWGME MRRCLLQSLQ QIESMIEAES SVKEKDMTKE FFENKSETWP IGESPKGVEE SSIGKVCRTL
LAKSVFNSLY ASPQLEGFSA ESRKLLLIVQ ALRDNLEPGT FDLGGLYEAI EECLINDPWV LLNASWFNSF LTHALS

HA

MKAILVVLLH TFATANADTL CIGYHANNST DTVDTVLEKN VTVTHSVNLL EDKHNGKLCK LRGVAPLHLG KCNIAGWILG
NPECESLSTA SSWSYIVETS SSDNGTCYPG NFIDYEELRE QLSSVSSFER FEIFPKTSSW PDHDSNKGVT AACPHAGAKS
FYKNLIWLVK KGNSYPTLSK SYINDKGKEV LVLWGIHHPS TSADQQSLYQ NADAYVFVGT SRYSKKFKPE IAIRPKVRNQ
EGRMNYYWTL VEPGDKITFE ATGNLVAPRY AFAMERNAGS GIIISDTPVH DCNTTCQTPK GAINTSLPFQ NIHPITIGKC
PKYVKSTKLR LATGLRNVPS IQSRGLFGAI AGFIEGGWTG MVDGWYGYHH QNEQGSGYAA DLKSTQNAID KITNKVNSVI
EKMNTQFTAV GKEFNHLEKR IENLNKKVDD GFLDIWTYNA ELLVLLENER TLDYHDSNVK NLYEKVRSQL KNNAKEIGNG
CFEFYHKCDN TCMESVKNGT YDYPKYSEEA KLNREEIDGV KLESTRIYQI LAIYSTVASS LVLVVSLGAI SFWMCSNGSL
QCRICI

NP

MASQGTKRSY EQMETDGERQ NATEIRASVG KMIGGIGRFY IQMCTELKLS DYEGRLIQNS LTIERMVLSA FDERRNKYLE
EHPSAGKDPK KTGGPIYRRV NGKWMRELIL YDKEEIRRIW RQANNGDDAT AGLTHMMIWH SNLNDATYQR TRALVRTGMD
PRMCSLMQGS TLPRRSGAAG AAVKGVGTMV MELVRMIKRG INDRNFWRGE NGRKTRIAYE RMCNILKGKF QTAAQKAMMD
QVRESRNPGN AEFEDLTFLA RSALILRGSV AHKSCLPACV YGPAVASGYD FEREGYSLVG IDPFRLLQNS QVYSLIRPNE
NPAHKSQLVW MACHSAAFED LRVLSFIKGT KVLPRGKLST RGVQIASNEN METMESSTLE LRSRYWAIRT RSGGNTNQQR
ASAGQISIQP TFSVQRNLPF DRTTIMAAFN GNTEGRTSDM RTEIIRMMES ARPEDVSFQG RGVFELSDEK AASPIVPSFD
MSNEGSYFFG DNAEEYDN

NA

MNPNQKIITI GSVCITIGMA NLILQIGNII SIWISHSIQL GNQNQIETCN QSVITYENNT WVNQTYVNIS NTNFAAGQSV
VSVKLAGNSS LCPVSGWAIY SKDNSIRIGS KGDVFVIREP FISCSPLECR TFFLTQGALL NDKHSNGTIK DRSPYRTLMS
CPIGEVPSPY NSRFESVAWS ASACHDGISW LTIGISGPDN GAVAVLKYNG IITDTIKSWR NNILRTQESE CACVNGSCFT
VMTDGPSDGQ ASYKIFRIEK GKIVKSVEMN APNYHYEECS CYPDSSEITC VCRDNWHGSN RPWVSFNQNL EYQIGYICSG
IFGDNPRPND KTGSCGPVSS NGANGVKGFS FKYGNGVWIG RTKSISSRNG FEIIWDPNGW TGTDNNFSIK QDIVGINEWS
GYSGSFVQHP ELTGLDCIRP CFWVELIRGR PKENTIWTSG SSISFCGVNS DTVGWSWPDG AELPFTIDK

M1

MSLLTEVETY VLSIIPSGPL KAEIAQRLED VFAGKNTDLE VLMEWLKTRP ILSPLTKGIL GFVFTLTVPS ERGLQRRRFV
QNALNGNGDP NNMDKAVKLY RKLKREITFH GAKEISLSYS AGALASCMGL IYNRMGAVTT EVAFGLVCAT CEQIADSQHR
SHRQMVTTTN PLIRHENRMV LASTTAKAME QMAGSSEQAA EAMEVASQAR QMVQAMRTIG THPSSSAGLK NDLLENLQAY
QKRMGVQMQR FK

M2

MSLLTEVETP IRNEWGCRCN GSSDPLTIAA NIIGILHLTL WILDRLFFKC IYRRFKYGLK GGPSTEGVPK SMREEYRKEQ
QSAVDADDGH FVSIELE

NS1

MDPNTVSSFQ VDCFLWHVRK RVADQELGDA PFLDRLRRDQ KSLRGRGSTL GLDIKTATRA GKQIVERILK EESDEALKMT
MASVPASRYL TDMTLEEMSR DWSMLIPKQK VAGPLCIRMD QAIMDKNIIL KANFSVIFDR LETLILLRAF TEEGAIVGEI
SPLPSLPGHT AEDVKNAVGV LIGGLEWNDN TVRVSETLQR FAWRSSNENG RPPLTPKQKR EMAGTIRSEV

NEP

MDPNTVSSFQ DILLRMSKMQ LESSSEDLNG MITQFESLKL YRDSLGEAVM RMGDLHSLQN RNEKWREQLG QKFEEIRWLI
EEVRHKLKIT ENSFEQITFM QALHLLLEVE QEIRTFSFQL I

B/Brisbane/60/2008

PB2

MTLAKIELLK QLLRDNEAKT VLKQTTVDQY NIIRKFNTSR IEKNPSLRMK WAMCSNFPLA LTKGDMANRI PLEYKGIQLK
TNAEDIGTKG QMCSIAAVTW WNTYGPIGDT EGFERVYESF FLRKMRLDNA TWGRITFGPV ERVRKRVLLN PLTKEMPPDE
ASNVIMEILF PKEAGIPRES TWIHRELIKE KREKLKGTMI TPIVLAYMLE RELVARRRFL PVAGATSAEF IEMLHCLQGE
NWRQIYHPGG NKLTESRSQS MIVACRKIIR RSIVASNPLE LAVEIANKTV IDTEPLKSCL AAIDGGDVAC DIIRAALGLK
IRQRQRFGRL ELKRISGRGF KNDEEILIGN GTIQKIGIWD GEEEFHVRCG ECRGILKKSK MKLEKLLINS AKKEDMRDLI
ILCMVFSQDT RMFQGVRGEI NFLNRAGQLL SPMYQLQRYF LNRSNDLFDQ WGYEESPKAS ELHGINESMN ASDYTLKGIV
VTRNVIDDFS STETEKVSIT KNLSLIKRTG EVIMGANDVS ELESQAQLMI TYDTPKMWEM GTTKELVQNT YQWVLKNLVT
LKAQFLLGKE DMFQWDAFEA FESIIPQKMA GQYSGFARAV LKQMRDQEVM KTDQFIKLLP FCFSPPKLRS NGEPYQFLKL
VLKGGGENFI EVRKGSPLFS YNPQTEVLTI CGRMMSLKGK IEDEERNRSM GNAVLAGFLV SGKYDPDLGD FKTIEELEKL

PB1

MNINPYFLFI DVPVQAAIST TFPYTGVPPY SHGTGTGYTI DTVIRTHEYS NKGKQYISDV TGCTMVDPTN GPLPEDNEPS
AYAQLDCVLE ALDRMDEEHP GLFQAASQNA MEALMVTTVD KLTQGRQTFD WTVCRNQPAA TALNTTITSF RLNDLNGADK
GGLIPFCQDI IDSLDRPEMT FFSVKNIKKK LPAKNKKGFL IKRIPMKVKD KITKVEYIKR ALSLNTMTKD AERGKLKRRA
IATAGIQIRG FVLVVENLAK NICENLEQSG LPVGGNEKKA KLSNAVAKML SNCPPGGISM TVTGDNTKWN ECLNPRIFLA
MTERITRDSP IWFRDFCSIA PVLFSNKIAR LGKGFMITSK TKRLKAQIPC PDLFSIPLER YNEETRAKLK KLKPFFNEEG
TASLSPGMMM GMFNMLSTVL GVAALGIKNI GNKEYLWDGL QSSDDFALFV NAKDEETCME GINDFYRTCK LLGVNMSKKK
SYCNETGMFE FTSMFYRDGF VSNFAMELPS FGVAGVNESA DMAIGMTIIK NNMINNGMGP ATAQTAIQLF IADYRYTYKC
HRGDSKVEGK RMKIIKELWE NTKGRDGLLV ADGGPNIYNL RNLHIPEIVL KYNLMDPEYK GRLLHPQNPF VGHLSIEGIK
EADITPAHGP VKKMDYDAVS GTHSWRTKRN RSILNTDQRN MILEEQCYAK CCNLFEACFN SASYRKPVGQ HSMLEAMAHR

PA

MDTFITRNFQ TTIIQKAKNT MAEFSEDPEL QPAMLFNICV HLEVCYVISD MNFLDEEGKA YTALEGQGKE QNLRPQYEVI
EGMPRTIAWM VQRSLAQEHG IETPKYLADL FDYKTKRFIE VGITKGLADD YFWKKKEKLG NSMELMIFSY NQDYSLSNES
SLDEEGKGRV LSRLTELQAE LSLKNLWQVL IGEEDVEKGI DFKLGQTISR LRDISVPAGF SNFEGMRSYI DNMDPKGAIE
RNLARMSPLV SVTPKKLTWE DLRPIGPHIY DHELPEVPYN AFLLMSDELG LANMTEGKSK KPKTLAKECL EKYSTLRDQT
DPILIMKSEK ANENFLWKLW RDCVNTISNE ETSNELQKTN YAKWATGDGL TYQKIMKEVA IDDETMCQEE PKIPNKCRVA
AWVQTEMNLL STLTSKRALD LPEIGPDIAP VEHVGSERRK YFVNEINYCK ASTVMMKYVL FHTSLLNESN ASMGKYKVIP
ITNRVVNEKG ESFDMLYGLA VKGQSHLRGD TDVVTVVTFE FSSTDPRVDS GKWPKYTVFR IGSLFVSGRE KSVYLYCRVN
GTNKIQMKWG MEARRCLLQS MQQMEAIVEQ ESSIQGYDMT KACFKGDRVN SPKTFSIGTQ EGKLVKGSFG KALRVIFTKC
LMHYVFGNAQ LEGFSAESRR LLLLIQALKD RKGPWVFDLE GMYSGIEECI SNNPWVIQSV YWFNEWLGFE KEGSKVLESV
DEIMDE

HA

MKAIIVLLMV VTSNADRICT GITSSNSPHV VKTATQGEVN VTGVIPLTTT PTKSHFANLK GTETRGKLCP KCLNCTDLDV
ALGRPKCTGK IPSARVSILH EVRPVTSGCF PIMHDRTKIR QLPNLLRGYE HIRLSTHNVI NAENAPGGPY KIGTSGSCPN
ITNGNGFFAT MAWAVPKNDK NKTATNPLTI EVPYICTEGE DQITVWGFHS DXETQMAKLY GDSKPQKFTS SANGVTTHYV
SQIGGFPNQT EDGGLPQSGR IVVDYMVQKS GKTGTITYQR GILLPQKVWC ASGRSKVIKG SLPLIGEADC LHEKYGGLNK
SKPYYTGEHA KAIGNCPIWV KTPLKLANGT KYRPPAKLLK ERGFFGAIAG FLEGGWEGMI AGWHGYTSHG AHGVAVAADL
KSTQEAINKI TKNLNSLSEL EVKNLQRLSG AMDELHNEIL ELDEKVDDLR ADTISSQIEL AVLLSNEGII NSEDEHLLAL
ERKLKKMLGP SAVEIGNGCF ETKHKCNQTC LDRIAAGTFD AGEFSLPTFD SLNITAASLN DDGLDNHTIL LYYSTAASSL
AVTLMIAIFV VYMVSRDNVS CSICL

NP

MSNMDIDGIN TGTIDKTPEE ITSGTSGTTR PIIRPATLAP PSNKRTRNPS PERATTSSED DVGRKTQKKQ TPTEIKKSVY
NMVVKLGEFY NQMMVKAGLN DDMERNLIQN AHAVERILLA ATDDKKTEFQ KKKNARDVKE GKEEIDHNKT GGTFYKMVRD
DKTIYFSPIR ITFLKEEVKT MYKTTMGSDG FSGLNHIMIG HSQMNDVCFQ RSKALKRVGL DPSLISTFAG STVPRRSGAT
GVAIKGGGTL VAEAIRFIGR AMADRGLLRD IKAKTAYEKI LLNLKNKCSA PQQKALVDQV IGSRNPGIAD IEDLTLLARS
MVVVRPSVAS KVVLPISIYA KIPQLGFNVE EYSMVGYEAM ALYNMATPVS ILRMGDDAKD KSQLFFMSCF GAAYEDLRVL
SALTGTEFKP RSALKCKGFH VPAKEQVEGM GAALMSIKLQ FWAPMTRSGG NEVGGDGGSG QISCSPVFAV ERPIALSKQA
VRRMLSMNIE GRDADVKGNL LKMMNDSMAK KTSGNAFIGK KMFQISDKNK TNPIEIPIKQ TIPNFFFGRD TAEDYDDLDY

NA

MLPSTIQTLT LFLTSGGVLL SLYVSASLSY LLYSDILLKF SPTEITAPTM PLDCANASNV QAVNRSATKG VTLLLPEPEW
TYPRLSCPGS TFQKALLISP HRFGETKGNS APLIIREPFI ACGPNECKHF ALTHYAAQPG GYYNGTRGDR NKLRHLISVK
LGKIPTVENS IFHMAAWSGS ACHDGKEWTY IGVDGPDNNA LLKVKYGEAY TDTYHSYANK ILRTQESACN CIGGNCYLMI
TDGSASGVSE CRFLKIREGR IIKEIFPTGR VKHTEECTCG FASNKTIECA CRDNSYTAKR PFVKLNVETD TAEIRLMCTD
TYLDTPRPND GSITGPCESN GDKGSGGIKG GFVHQRMESK IGRWYSRTMS KTERMGMGLY VKYDGDPWAD SDALAFSGVM
VSMKEPGWYS FGFEIKDKKC DVPCIGIEMV HDGGKETWHS AATAIYCLMG SGQLLWDTVT GVDMAL

NB

MNNATFNYTN VNPIPHIRGS VIITICVSFI IILTIFGYIA KILTNRNNCT NNAIGLCKRI KCSGCEPFCN KRGDTSSPRT
GVDIPAFILP GLNLSESTPN

M1

MSLFGDTIAY LLSLTEDGEG KAELAEKLHC WFGGKEFDLD SALEWIKNKR CLTDIQKALI GASICFLKPK DQERKRRFIT
EPLSGMGTTA TKKKGLILAE RKMRRCVSFH EAFEIAEGHE SSALLYCLMV MYLNPGNYSM QVKLGTLCAL CEKQASHSHR
AHSRAARSSV PGVRREMQMV SAMNTAKTMN GMGKGEDVQK LAEELQSNIG VLRSLGASQK NGEGIAKDVM EVLKQSSMGN
SALVKKYL

BM2

MLEPFQILTI CSFILSALHF MAWTIGHLNQ IKRGINMKIR IKGPNKETIN REVSILRHSY QKEIQAKETM KEVLSDNMEV
LNDHIIIEGL SAEEIIKMGE TVLEIEELH

NS1

MANNNMTTTQ IEVGPGATNA TINFEAGILE CYERLSWQRA LDYPGQDRLN RLKRKLESRI KTHNKSEPES KRMSLEERKA
IGVKMMKVLL FMNPSAGIEG FEPYCMKSSS NSNCTKYNWT DYPSTPERCL DDIEEEPEDV DGPTEIVLRD MNNKDARQKI
KEEVNTQKEG KFRLTIKRDM RNVLSLRVLV NGTFLKHPNG HKSLSTLHRL NAYDQSGRLV AKLVATDDLT VEDEEDGHRI
LNSLFERLNE GHSKPIRAAE TAVGVLSQFG QEHRLSPEEG DN

NEP

MANNNMTTTQ IEWRMKKMAI GSSTHSSSVL MKDIQSQFEQ LKLRWESYPN LVKSTDYHQK RETIRLVTEE LYLLSKRIDD
NILFHKTVIA NSSIIADMVV SLSLLETLYE MKDVVEVYSR QCL

PB2, PB1, PA (TAP-purified)

PB2

```
MERIKELRNL MSQSRTREIL TKTTVDHMAI IKKYTSGRQE KNPALRMKWM MAMKYPITAD KRITEMIPER NEQGQTLWSK
MNDAGSDRVM VSPLAVTWWN RNGPVTSTVH YPKIYKTYFE KVERLKHGTF GPVHFRNQVK IRRRVDINPG HADLSAKEAQ
DVIMEVVFPN EVGARILTSE SQLTTTKEKK EELQGCKISP LMVAYMLERE LVRKTRFLPV AGGTSSVYIE VLHLTQGTCW
EQMYTPGGEA RNDDVDQSLI IAARNIVRRA TVSADPLASL LEMCHSTQIG GIRMVNILRQ NPTEEQAVDI CKAAMGLRIS
SSFSFGGFTF KRTSGSSVKR EEEVLTGNLQ TLKIRVHEGY EEFTMVGRRA TAILRKATRR LIQLIVSGRD EQSIAEAIIV
AMVFSQEDCM IKAVRGDLNF VNRANQRLNP MHQLLRHFQK DAKVLFQNWG IESIDNVMGM IGILPDMTPS TEMSMRGVRI
SKMGVDEYSS AEKIVVSIDR FLRVRDQRGN VLLSPEEVSE TQGTEKLTIT YSSSMMWEIN GPESVLVNTY QWIIRNWETV
KIQWSQNPTM LYNKMEFEPF QSLVPKAVRG QYSGFVRTLF QQMRDVLGTF DTAQIIKLLP FAAAPPKQSG MQFSSLTINV
RGSGMRILVR GNSPVFNYNK TTKRLTVLGK DAGPLTEDPD EGTAGVESAV LRGFLILGKE DRRYGPALSI NELSNLAKGE
KANVLIGQGD VVLVMKRKRN SSILTDSQTA TKRIRMAIN
```

PB1

```
MDVNPTLLFL KVPAQNAIST TFPYTGDPPY SHGTGTGYTM DTVNRTHQYS ERGRWTTNTE TGAPQLNPID GPLPEDNEPS
GYAQTDCVLE AMAFLEESHP GIFETSCLET MEVVQQTRVD KLTQGRQTYD WTLNRNQPAA TALANTIEVF RSNGLTANES
GRLIDFLKDV MESMNKEEME ITTHFQRKRR VRDNMTKKMV TQRTIGKRKQ RLNKRSYLIR ALTLNTMTKD AERGKLKRRA
IATPGMQIRG FVYFVETLAR SICEKLEQSG LPVGGNEKKA KLANVVRKMM TNSQDTEISF TITGDNTKWN ENQNPRMFLA
MITYITRNQP EWFRNVLSIA PIMFSNKMAR LGKGYMFESK SMKLRTQIPA EMLASIDLKY FNDSTRKKIE KIRPLLIDGT
ASLSPGMMMG MFNMLSTVLG VSILNLGQKR HTKTTYWWDG LQSSDDFALI VNAPNHEGIQ AGVNRFYRTC KLLGINMSKK
KSYINRTGTF EFTSFFYRYG FVANFSMELP SFGVSGINES ADMSIGVTVI KNNMINNDLG PATAQMALQL FIKDYRYTYR
CHRGDTQIQT RRSFEIKKLW EQTHSKAGLL VSDGGPNLYN IRNLHIPEVC LKWELMDEDY QGRLCNPLNP FVNHKDIESV
NNAVIMPAHG PAKNMEYDAV ATTHSWIPKR NRSILNTSQR GILEDEQMYQ KCCNLFEKFF PSSSYRRPVG ISSMVEAMVS
RARIDARIDF ESGRIKKEEF TEIMKICSTI EELRRQK
```

PA

```
MEDFVRQCFN PMIVELAEKA MKEYGEDLKI ETNKFAAICT HLEVCFMYSD FHFIDEQGES IVVELGDPNA LLKHRFEIIE
GRDRTIAWTV INSICNTTGA EKPKFLPDLY DYKKNRFIEI GVTRREVHIY YLEKANKIKS EKTHIHIFSF TGEEMATKAD
YTLDEESRAR IKTRLFTIRQ EMASRGLWDS FRQSERGEET IEERFEITGT MRKLADQSLP PNFSSLENFR AYVDGFEPNG
YIEGKLSQMS KEVNARIEPF LKSTPRPLRL PDGPPCSQRS KFLLMDALKL SIEDPSHEGE GIPLYDAIKC MRTFFGWKEP
NVVKPHEKGI NPNYLLSWKQ VLAELQDIEN EEKIPRTKNM KKTSQLKWAL GENMAPEKVD FDDCKDVGDL KQYDSDEPEL
RSLASWIQNE FNKACELTDS SWIELDEIGE DAAPIEHIAS MRRNYFTAEV SHCRATEYIM KGVYINTALL NASCAAMDDF
QLIPMISKCR TKEGRRKTNL YGFIIKGRSH LRNDTDVVNF VSMEFSLTDP RLEPHKWEKY CVLEVGDMLL RSAIGHVSRP
MFLYVRTNGT SKIKMKWGME MRRCLLQSLQ QIESMIEAES SVKEKDMTKE FFENKSETWP VGESPKGVEE GSIGKVCRTL
LAKSVFNSLY ASPQLEGFSA ESRKLLLIVQ ALRDNLEPGT FDLGGLYEAI EECLINDPWV LLNASWFNSF LTHALR
```

NP (TAP-purified)

NP

```
MATKGTKRSY EQMETDGERQ NATEIRASVG KMIDGIGRFY IQMCTELKLS DYEGRLIQNS LTIERMVLSA FDERRNKYLE
EHPSAGKDPK KTGGPIYRRV DGKWRRELIL YDKEEIRRIW RQANNGDDAT AGLTHMMIWH SNLNDATYQR TRALVRTGMD
PRMCSLMQGS TLPRRSGAAG AAVKGVGTMV MELIRMIKRG INDRNFWRGE NGRRTRIAYE RMCNILKGKF QTAAQRTMVD
QVRESRNPGN AEFEDLIFLA RSALILRGSV AHKSCLPACV YGSAVASGYD FEREGYSLVG IDPFRLLQNS QVYSLIRPNE
NPAHKSQLVW MACHSAAFED LRVSSFIRGT KVVPRGKLST RGVQIASNEN METMESSTLE LRSRYWAIRT RSGGNTNQQR
ASSGQISIQP TFSVQRNLPF DRPTIMAAFT GNTEGRTSDM RTEIIRLMES ARPEDVSFQG RGVFELSDEK ATSPIVPSFD
MSNEGSYFFG DNAEEYDN
```

Strep-purified WSN proteins

PB2

MERIKELRNL MSQSRTREIL TKTTVDHMAI IKKYTSGRQE KNPALRMKWM MAMKYPITAD KRITEMIPER NEQGQTLWSK
MNDAGSDRVM VSPLAVTWWN RNGPVTSTVH YPKIYKTYFE KVERLKHGTF GPVHFRNQVK IRRRVDINPG HADLSAKEAQ
DVIMEVVFPN EVGARILTSE SQLTTTKEKK EELQGCKISP LMVAYMLERE LVRKTRFLPV AGGTSSVYIE VLHLTQGTCW
EQMYTPGGEA RNDDVDQSLI IAARNIVRRA TVSADPLASL LEMCHSTQIG GIRMVNILRQ NPTEEQAVDI CKAAMGLRIS
SSFSFGGFTF KRTSGSSVKR EEEVLTGNLQ TLKIRVHEGY EEFTMVGRRA TAILRKATRR LIQLIVSGRD EQSIAEAIIV
AMVFSQEDCM IKAVRGDLNF VNRANQRLNP MHQLLRHFQK DAKVLFQNWG IESIDNVMGM IGILPDMTPS TEMSMRGVRI
SKMGVDEYSS AEKIVVSIDR FLRVRDQRGN VLLSPEEVSE TQGTEKLTIT YSSSMMWEIN GPESVLVNTY QWIIRNWETV
KIQWSQNPTM LYNKMEFEPF QSLVPKAVRG QYSGFVRTLF QQMRDVLGTF DTAQIIKLLP FAAAPPKQSG MQFSSLTINV
RGSGMRILVR GNSPVFNYNK TTKRLTVLGK DAGPLTEDPD EGTAGVESAV LRGFLILGKE DRRYGPALSI NELSNLAKGE
KANVLIGQGD VVLVMKRKRN SSILTDSQTA TKRIRMAIN

PB1

MDVNPTLLFL KVPAQNAIST TFPYTGDPPY SHGTGTGYTM DTVNRTHQYS ERGRWTTNTE TGAPQLNPID GPLPEDNEPS
GYAQTDCVLE AMAFLEESHP GIFETSCLET MEVVQQTRVD KLTQGRQTYD WTLNRNQPAA TALANTIEVF RSNGLTANES
GRLIDFLKDV MESMNKEEME ITTHFQRKRR VRDNMTKKMV TQRTIGKRKQ RLNKRSYLIR ALTLNTMTKD AERGKLKRRA
IATPGMQIRG FVYFVETLAR SICEKLEQSG LPVGGNEKKA KLANVVRKMM TNSQDTEISF TITGDNTKWN ENQNPRMFLA
MITYITRNQP EWFRNVLSIA PIMFSNKMAR LGKGYMFESK SMKLRTQIPA EMLASIDLKY FNDSTRKKIE KIRPLLIDGT
ASLSPGMMMG MFNMLSTVLG VSILNLGQKR HTKTTYWWDG LQSSDDFALI VNAPNHEGIQ AGVNRFYRTC KLLGINMSKK
KSYINRTGTF EFTSFFYRYG FVANFSMELP SFGVSGINES ADMSIGVTVI KNNMINNDLG PATAQMALQL FIKDYRYTYR
CHRGDTQIQT RRSFEIKKLW EQTHSKAGLL VSDGGPNLYN IRNLHIPEVC LKWELMDEDY QGRLCNPLNP FVNHKDIESV
NNAVIMPAHG PAKNMEYDAV ATTHSWIPKR NRSILNTSQR GILEDEQMYQ KCCNLFEKFF PSSSYRRPVG ISSMVEAMVS
RARIDARIDF ESGRIKKEEF TEIMKICSTI EELRRQK

PA

MEDFVRQCFN PMIVELAEKA MKEYGEDLKI ETNKFAAICT HLEVCFMYSD FHFIDEQGES IVVELGDPNA LLKHRFEIIE
GRDRTIAWTV INSICNTTGA EKPKFLPDLY DYKKNRFIEI GVTRREVHIY YLEKANKIKS EKTHIHIFSF TGEEMATKAD
YTLDEESRAR IKTRLFTIRQ EMASRGLWDS FRQSERGEET IEERFEITGT MRKLADQSLP PNFSSLENFR AYVDGFEPNG
YIEGKLSQMS KEVNARIEPF LKSTPRPLRL PDGPPCSQRS KFLLMDALKL SIEDPSHEGE GIPLYDAIKC MRTFFGWKEP
NVVKPHEKGI NPNYLLSWKQ VLAELQDIEN EEKIPRTKNM KKTSQLKWAL GENMAPEKVD FDDCKDVGDL KQYDSDEPEL
RSLASWIQNE FNKACELTDS SWIELDEIGE DAAPIEHIAS MRRNYFTAEV SHCRATEYIM KGVYINTALL NASCAAMDDF
QLIPMISKCR TKEGRRKTNL YGFIIKGRSH LRNDTDVVNF VSMEFSLTDP RLEPHKWEKY CVLEVGDMLL RSAIGHVSRP
MFLYVRTNGT SKIKMKWGME MRRCLLQSLQ QIESMIEAES SVKEKDMTKE FFENKSETWP VGESPKGVEE GSIGKVCRTL
LAKSVFNSLY ASPQLEGFSA ESRKLLLIVQ ALRDNLEPGT FDLGGLYEAI EECLINDPWV LLNASWFNSF LTHALR

HA

MKAFVLVLLY AFVATDADTI CIGYHANNST DTVDTIFEKN VAVTHSVNLL EDRHNGKLCK LKGIAPLQLG KCNITGWLLG
NPECDSLLPA RSWSYIVETP NSENGACYPG DFIDYEELRE QLSSVSSLER FEIFPKESSW PNHTFNGVTV SCSHRGKSSF
YRNLLWLTKK GDSYPKLTNS YVNNKGKEVL VLWGVHHPSS SDEQQSLYSN GNAYVSVASS NYNRRFTPEI AARPKVKDQH
GRMNYYWTLL EPGDTIIFEA TGNLIAPWYA FALSRGFESG IITSNASMHE CNTKCQTPQG SINSNLPFQN IHPVTIGECP
KYVRSTKLRM VTGLRNIPSI QYRGLFGAIA GFIEGGWTGM IDGWYGYHHQ NEQGSGYAAD QKSTQNAING ITNKVNSVIE
KMNTQFTAVG KEFNNLEKRM ENLNKKVDDG FLDIWTYNAE LLVLLENERT LDFHDLNVKN LYEKVKSQLK NNAKEIGNGC
FEFYHKCDNE CMESVRNGTY DYPKYSEESK LNREKIDGVK LESMGVYQIL AIYSTVASSL VLLVSLGAIS FWMCSNGSLQ
CRICI

NP

MATKGTKRSY EQMETDGERQ NATEIRASVG KMIDGIGRFY IQMCTELKLS DYEGRLIQNS LTIERMVLSA FDERRNKYLE
EHPSAGKDPK KTGGPIYRRV DGKWRRELIL YDKEEIRRIW RQANNGDDAT AGLTHMMIWH SNLNDATYQR TRALVRTGMD
PRMCSLMQGS TLPRRSGAAG AAVKGVGTMV MELIRMIKRG INDRNFWRGE NGRRTRIAYE RMCNILKGKF QTAAQRTMVD
QVRESRNPGN AEFEDLIFLA RSALILRGSV AHKSCLPACV YGSAVASGYD FEREGYSLVG IDPFRLLQNS QVYSLIRPNE
NPAHKSQLVW MACHSAAFED LRVSSFIRGT KVVPRGKLST RGVQIASNEN METMESSTLE LRSRYWAIRT RSGGNTNQQR
ASSGQISIQP TFSVQRNLPF DRPTIMAAFT GNTEGRTSDM RTEIIRLMES ARPEDVSFQG RGVFELSDEK ATSPIVPSFD
MSNEGSYFFG DNAEEYDN

M1

MSLLTEVETY VLSIVPSGPL KAEIAQRLED VFAGKNTDLE VLMEWLKTRP ILSPLTKGIL GFVFTLTVPS ERGLQRRRFV
QNALNGNGDP NNMDKAVKLY RKLKREITFH GAKEIALSYS AGALASCMGL IYNRMGAVTT EVAFGLVCAT CEQIADSQHR
SHRQMVTTTN PLIRHENRMV LASTTAKAME QMAGSSEQAA EAMDIASQAR QMVQAMRTIG THPSSSAGLK DDLLENLQAY
QKRMGVQMQR FK

NS1

MDPNTVSSFQ VDCFLWHVRK RVADQELGDA PFLDRLRRDQ KSLRGRGSTL GLDIETATRA GKQIVERILK EESDEALKMT
MASVPASRYL TDMTLEEMSR HWFMLMPKQK VAGPLCIRMD QAIMDKNIIL KANFSVIFDR LETLILLRAF TEEGTIVGEI
SPLPSLPGHT DEDVKNAVGV LIGGLEWNNN TVRVSETLQR FAWRSSNENG RPPLTPKQKR KMAGTIRSEV

WSN proteins from 293T cell lysates

NP

MATKGTKRSY EQMETDGERQ NATEIRASVG KMIDGIGRFY IQMCTELKLS DYEGRLIQNS LTIERMVLSA FDERRNKYLE
EHPSAGKDPK KTGGPIYRRV DGKWRRELIL YDKEEIRRIW RQANNGDDAT AGLTHMMIWH SNLNDATYQR TRALVRTGMD
PRMCSLMQGS TLPRRSGAAG AAVKGVGTMV MELIRMIKRG INDRNFWRGE NGRRTRIAYE RMCNILKGKF QTAAQRTMVD
QVRESRNPGN AEFEDLIFLA RSALILRGSV AHKSCLPACV YGSAVASGYD FEREGYSLVG IDPFRLLQNS QVYSLIRPNE
NPAHKSQLVW MACHSAAFED LRVSSFIRGT KVVPRGKLST RGVQIASNEN METMESSTLE LRSRYWAIRT RSGGNTNQQR
ASSGQISIQP TFSVQRNLPF DRPTIMAAFT GNTEGRTSDM RTEIIRLMES ARPEDVSFQG RGVFELSDEK ATSPIVPSFD
MSNEGSYFFG DNAEEYDN

M1

MSLLTEVETY VLSIVPSGPL KAEIAQRLED VFAGKNTDLE VLMEWLKTRP ILSPLTKGIL GFVFTLTVPS ERGLQRRRFV
QNALNGNGDP NNMDKAVKLY RKLKREITFH GAKEIALSYS AGALASCMGL IYNRMGAVTT EVAFGLVCAT CEQIADSQHR
SHRQMVTTTN PLIRHENRMV LASTTAKAME QMAGSSEQAA EAMDIASQAR QMVQAMRTIG THPSSSAGLK DDLLENLQAY
QKRMGVQMQR FK

M2

MSLLTEVETP IRNEWGCRCN DSSDPLVIAA NIIEILHLIL WILDRLFFKC IYRRFKYGLK RGPSTEGVPE SMREEYRKEQ
QNAVDVDDGH FVNIELE

NS1

MDPNTVSSFQ VDCFLWHVRK RVADQELGDA PFLDRLRRDQ KSLRGRGSTL GLDIETATRA GKQIVERILK EESDEALKMT
MASVPASRYL TDMTLEEMSR HWFMLMPKQK VAGPLCIRMD QAIMDKNIIL KANFSVIFDR LETLILLRAF TEEGTIVGEI
SPLPSLPGHT DEDVKNAVGV LIGGLEWNNN TVRVSETLQR FAWRSSNENG RPPLTPKQKR KMAGTIRSEV

WSN proteins from MDBK cell lysates

NP

MATKGTKRSY EQMETDGERQ NATEIRASVG KMIDGIGRFY IQMCTELKLS DYEGRLIQNS LTIERMVLSA FDERRNKYLE
EHPSAGKDPK KTGGPIYRRV DGKWRRELIL YDKEEIRRIW RQANNGDDAT AGLTHMMIWH SNLNDATYQR TRALVRTGMD
PRMCSLMQGS TLPRRSGAAG AAVKGVGTMV MELIRMIKRG INDRNFWRGE NGRRTRIAYE RMCNILKGKF QTAAQRTMVD
QVRESRNPGN AEFEDLIFLA RSALILRGSV AHKSCLPACV YGSAVASGYD FEREGYSLVG IDPFRLLQNS QVYSLIRPNE
NPAHKSQLVW MACHSAAFED LRVSSFIRGT KVVPRGKLST RGVQIASNEN METMESSTLE LRSRYWAIRT RSGGNTNQQR
ASSGQISIQP TFSVQRNLPF DRPTIMAAFT GNTEGRTSDM RTEIIRLMES ARPEDVSFQG RGVFELSDEK ATSPIVPSFD
MSNEGSYFFG DNAEEYDN

M1

MSLLTEVETY VLSIVPSGPL KAEIAQRLED VFAGKNTDLE VLMEWLKTRP ILSPLTKGIL GFVFTLTVPS ERGLQRRRFV
QNALNGNGDP NNMDKAVKLY RKLKREITFH GAKEIALSYS AGALASCMGL IYNRMGAVTT EVAFGLVCAT CEQIADSQHR
SHRQMVTTTN PLIRHENRMV LASTTAKAME QMAGSSEQAA EAMDIASQAR QMVQAMRTIG THPSSSAGLK DDLLENLQAY
QKRMGVQMQR FK

NS1

MDPNTVSSFQ VDCFLWHVRK RVADQELGDA PFLDRLRRDQ KSLRGRGSTL GLDIETATRA GKQIVERILK EESDEALKMT
MASVPASRYL TDMTLEEMSR HWFMLMPKQK VAGPLCIRMD QAIMDKNIIL KANFSVIFDR LETLILLRAF TEEGTIVGEI
SPLPSLPGHT DEDVKNAVGV LIGGLEWNNN TVRVSETLQR FAWRSSNENG RPPLTPKQKR KMAGTIRSEV
