# Supplementary material for: Mapping the Phosphoproteome of Influenza A and B Viruses by Mass Spectrometry
Source: PLoS Pathog. 2012 Nov 8;8(11):e1002993. doi: 10.1371/journal.ppat.1002993 (PMC3493474; doi:10.1371/journal.ppat.1002993)
Supplement: Figure S3 — Representative spectra. Fragment ion spectra for N-terminal peptides and for phosphorylated peptides, annotated by Mascot or CPFP. The clearest spectrum showing each modification referred to in the text is presented (including overlapping peptides and unmodified peptides where available). Each spectrum was manually inspected. (PDF) [file ppat.1002993.s003.pdf]

Influenza A/WSN/33
N-terminal peptide spectra

MATRIX SCIENCE

Mascot Search Results

Peptide View

MS/MS Fragmentation of **MDVNPTLLFLK**
Found in **PB1**

Match to Query 1987: 1331.714600 from(666.864576,2+)
Title: Elution from: 40.937 to 40.937 period: 0 experiment: 1 cycles: 1 precIntensity: 462945.0 FinneganScanNumber: 3064 MStype: enumIsNormalMS rawFile: B110401_016.RAW
Data file file1_B110401_016.mgf

Click mouse within plot area to zoom in by factor of two about that point
Or, Plot from 100 to 1400 Da Full range
Label all possible matches ○ Label matches used for scoring ◉

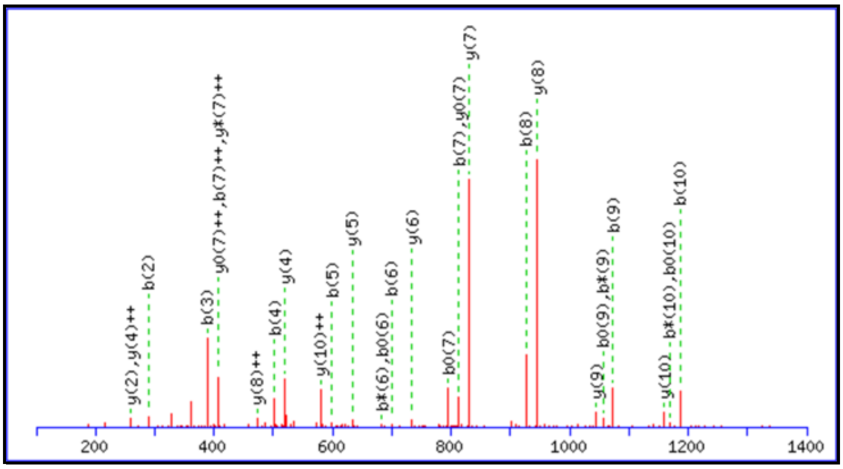

Monoisotopic mass of neutral peptide Mr(calc): 1331.7159
Fixed modifications: Carbamidomethyl (C) (apply to specified residues or termini only)
Variable modifications:
N-term : Acetyl (Protein N-term)
Ions Score: 77 **Expect:** 3.8e-06
Matches : 32/102 fragment ions using 34 most intense peaks (help)

#	b	b^{++}	b*	b^{*++}	b^0	b^{0++}	Seq.	y	y^{++}	y*	y^{*++}	y^0	y^{0++}	#
1	174.0583	87.5328					M							11
2	***289.0853***	145.0463			271.0747	136.0410	D	***1159.6721***	580.3397	1142.6456	571.8264	1141.6616	571.3344	10
3	***388.1537***	194.5805			370.1431	185.5752	V	***1044.6452***	522.8262	1027.6186	514.3130	1026.6346	513.8210	9
4	***502.1966***	251.6019	485.1701	243.0887	484.1860	242.5967	N	***945.5768***	473.2920	928.5502	464.7788	927.5662	464.2867	8
5	***599.2494***	300.1283	582.2228	291.6151	581.2388	291.1230	P	***831.5339***	416.2706	814.5073	407.7573	813.5233	407.2653	7
6	***700.2971***	350.6522	683.2705	342.1389	682.2865	341.6469	T	***734.4811***	367.7442	717.4545	359.2309	716.4705	358.7389	6
7	***813.3811***	407.1942	796.3546	398.6809	795.3706	398.1889	L	***633.4334***	317.2203	616.4069	308.7071			5
8	***926.4652***	463.7362	909.4386	455.2230	908.4546	454.7309	L	***520.3493***	260.6783	503.3228	252.1650			4
9	***1073.5336***	537.2704	1056.5070	528.7572	1055.5230	528.2652	F	***407.2653***	204.1363	390.2387	195.6230			3
10	***1186.6177***	593.8125	1169.5911	585.2992	1168.6071	584.8072	L	***260.1969***	130.6021	243.1703	122.0888			2
11							K	147.1128	74.0600	130.0863	65.5468			1

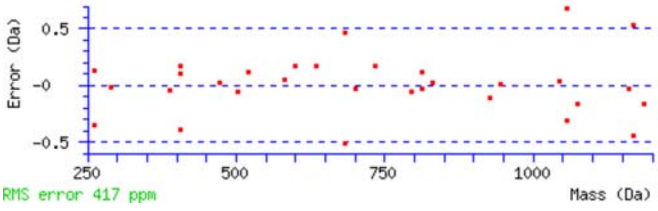

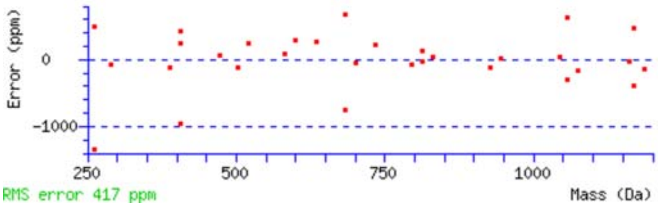

NCBI **BLAST** search of MDVNPTLLFLK
(Parameters: blastp, nr protein database, expect=20000, no filter, PAM30)
Other BLAST web gateways

All matches to this query

Score	Mr(calc):	Delta	Sequence
76.8	1331.7159	-0.0013	MDVNPTLLFLK
20.4	1331.7074	0.0072	RKSLHENKLK
16.4	1331.7384	-0.0238	HIHLMPLSQIK
14.9	1331.6907	0.0239	YIKDYMXSIK
14.8	1331.7006	0.0140	LESMQLELAGLK
14.5	1331.6962	0.0184	QLEISHRKLK
13.2	1331.7296	-0.0150	DKIKENSEKIK
12.5	1331.7318	-0.0172	QMKMKVHRFK
11.2	1331.7006	0.0140	LKQQVMDEVIK
11.1	1331.7214	-0.0068	NVLLPQLSLAGK

Mascot: http://www.matrixscience.com/

Mascot Search Results

Peptide View

MS/MS Fragmentation of **MSLLTEVETYVLSIVPSGPLK**
Found in **M1**

Match to Query 4783: 2275.235906 from(1138.625229,2+)
Title: Elution from: 102.078 to 102.078 period: 0 experiment: 1 cycles: 1 precIntensity: 221931.0 FinneganScanNumber: 8062 MStype: enumIsNormalMS rawFile: B110210_012.RAW
Data file file1_B110210_012.mgf

Click mouse within plot area to zoom in by factor of two about that point
Or, Plot from 300 to 2000 Da Full range
Label all possible matches ○ Label matches used for scoring ◉

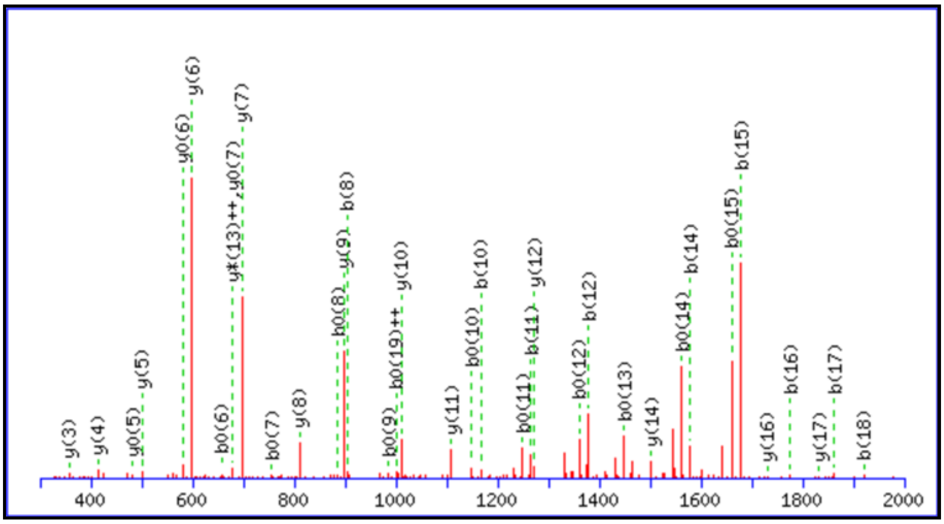

Monoisotopic mass of neutral peptide Mr(calc): 2275.2385
Fixed modifications: Carbamidomethyl (C) (apply to specified residues or termini only)
Ions Score: 98 **Expect:** 1.6e-08
Matches : 37/190 fragment ions using 49 most intense peaks (help)

#	b	b^{++}	b^{0}	b^{0++}	Seq.	y	y^{++}	y*	y^{*++}	y^{0}	y^{0++}	#
1	132.0478	66.5275			M							21
2	219.0798	110.0435	201.0692	101.0382	S	2145.2053	1073.1063	2128.1788	1064.5930	2127.1947	1064.1010	20
3	332.1639	166.5856	314.1533	157.5803	L	2058.1733	1029.5903	2041.1467	1021.0770	2040.1627	1020.5850	19
4	445.2479	223.1276	427.2374	214.1223	L	1945.0892	973.0482	1928.0627	964.5350	1927.0787	964.0430	18
5	546.2956	273.6514	528.2850	264.6462	T	1832.0052	916.5062	1814.9786	907.9929	1813.9946	907.5009	17
6	675.3382	338.1727	657.3276	329.1675	E	1730.9575	865.9824	1713.9309	857.4691	1712.9469	856.9771	16
7	774.4066	387.7069	756.3960	378.7017	V	1601.9149	801.4611	1584.8883	792.9478	1583.9043	792.4558	15
8	903.4492	452.2282	885.4386	443.2230	E	1502.8465	751.9269	1485.8199	743.4136	1484.8359	742.9216	14
9	1004.4969	502.7521	986.4863	493.7468	T	1373.8039	687.4056	1356.7773	678.8923	1355.7933	678.4003	13
10	1167.5602	584.2837	1149.5496	575.2785	Y	1272.7562	636.8817	1255.7297	628.3685	1254.7456	627.8765	12
11	1266.6286	633.8179	1248.6181	624.8127	V	1109.6929	555.3501	1092.6663	546.8368	1091.6823	546.3448	11
12	1379.7127	690.3600	1361.7021	681.3547	L	1010.6245	505.8159	993.5979	497.3026	992.6139	496.8106	10
13	1466.7447	733.8760	1448.7341	724.8707	S	897.5404	449.2738	880.5138	440.7606	879.5298	440.2686	9
14	1579.8288	790.4180	1561.8182	781.4127	I	810.5084	405.7578	793.4818	397.2445	792.4978	396.7525	8
15	1678.8972	839.9522	1660.8866	830.9469	V	697.4243	349.2158	680.3978	340.7025	679.4137	340.2105	7
16	1775.9500	888.4786	1757.9394	879.4733	P	598.3559	299.6816	581.3293	291.1683	580.3453	290.6763	6
17	1862.9820	931.9946	1844.9714	922.9893	S	501.3031	251.1552	484.2766	242.6419	483.2926	242.1499	5
18	1920.0034	960.5054	1901.9929	951.5001	G	414.2711	207.6392	397.2445	199.1259			4

**19**	2017.0562	1009.0317	1999.0456	1000.0265	**P**	***357.2496***	179.1285	340.2231	170.6152			**3**
**20**	2130.1403	1065.5738	2112.1297	1056.5685	**L**	260.1969	130.6021	243.1703	122.0888			**2**
**21**					**K**	147.1128	74.0600	130.0863	65.5468			**1**

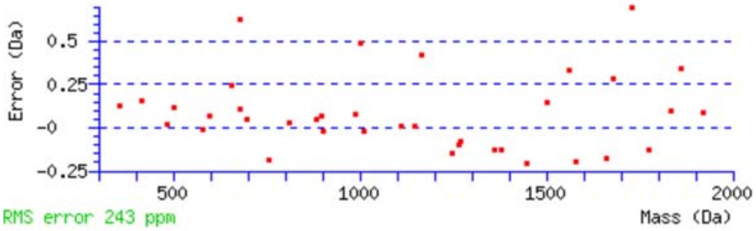

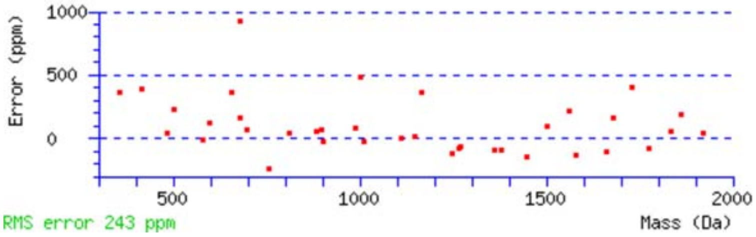

NCBI **BLAST** search of MSLLTEVETYVLSIVPSGPLK
(Parameters: blastp, nr protein database, expect=20000, no filter, PAM30)
Other BLAST web gateways

All matches to this query

Score	Mr(calc):	Delta	Sequence
98.4	2275.2385	-0.0026	MSLLTEVETYVLSIVPSGPLK
14.4	2275.2089	0.0270	LALFRNNENKVSVIKPPEK
8.1	2275.2341	0.0018	TRIAEVGGVPYLLPLVNEKK
4.9	2275.2246	0.0113	QILLGPNTGLSGGMPGALPSLPGK
4.9	2275.2246	0.0113	QILLGPNTGLSGGMPGALPSLPGK
3.3	2275.2228	0.0131	DLKGALQKLLLSKNPFAQPL
2.0	2275.2220	0.0139	MEPDIKMKIAWRYQLLPK
1.9	2275.2664	-0.0305	VLDKISIGRLVSLLSNNLNK
1.9	2275.2228	0.0131	DLKGALQKLLLSKNPFAQPL
1.7	2275.2172	0.0187	AAAPSLGEGGGIVSVSVSPGPLPTR

Mascot: http://www.matrixscience.com/

{MATRIX SCIENCE} Mascot Search Results

Peptide View

MS/MS Fragmentation of **MSLLTEVETYVLSIVPSGPLK**
Found in **M1**

Match to Query 4937: 2317.247888 from(1159.631220,2+)
Title: Elution from: 109.168 to 109.168 period: 0 experiment: 1 cycles: 1 precIntensity: 25399.0 FinneganScanNumber: 8597
MStype: enumIsNormalMS rawFile: B110210_012.RAW
Data file file1_B110210_012.mgf

Click mouse within plot area to zoom in by factor of two about that point
Or, Plot from 300 to 1900 Da Full range
Label all possible matches ○ Label matches used for scoring ◉

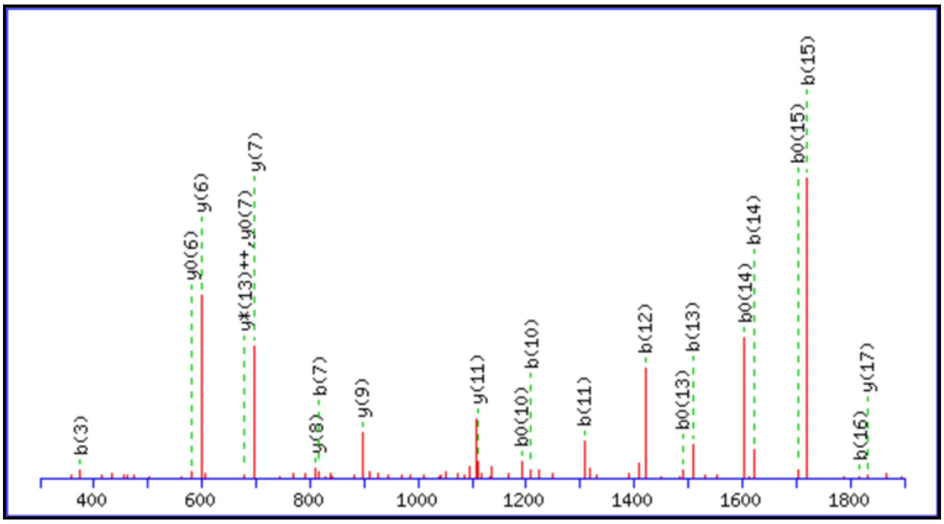

Monoisotopic mass of neutral peptide Mr(calc): 2317.2491
Fixed modifications: Carbamidomethyl (C) (apply to specified residues or termini only)
Variable modifications:
N-term : Acetyl (Protein N-term)
Ions Score: 56 **Expect:** 4.3e-05
Matches : 22/190 fragment ions using 32 most intense peaks (help)

#	b	b^{++}	b^0	b^{0++}	Seq.	y	y^{++}	y*	y^{*++}	y^0	y^{0++}	#
1	174.0583	87.5328			M							21
2	261.0904	131.0488	243.0798	122.0435	S	2145.2053	1073.1063	2128.1788	1064.5930	2127.1947	1064.1010	20
3	**374.1744**	187.5908	356.1639	178.5856	L	2058.1733	1029.5903	2041.1467	1021.0770	2040.1627	1020.5850	19
4	487.2585	244.1329	469.2479	235.1276	L	1945.0892	973.0482	1928.0627	964.5350	1927.0787	964.0430	18
5	588.3062	294.6567	570.2956	285.6514	T	**1832.0052**	916.5062	1814.9786	907.9929	1813.9946	907.5009	17
6	717.3488	359.1780	699.3382	350.1727	E	1730.9575	865.9824	1713.9309	857.4691	1712.9469	856.9771	16
7	**816.4172**	408.7122	798.4066	399.7069	V	1601.9149	801.4611	1584.8883	792.9478	1583.9043	792.4558	15
8	945.4598	473.2335	927.4492	464.2282	E	1502.8465	751.9269	1485.8199	743.4136	1484.8359	742.9216	14
9	1046.5074	523.7574	1028.4969	514.7521	T	1373.8039	687.4056	1356.7773	678.8923	1355.7933	678.4003	13
10	**1209.5708**	605.2890	1191.5602	596.2837	Y	1272.7562	636.8817	1255.7297	628.3685	1254.7456	627.8765	12
11	**1308.6392**	654.8232	1290.6286	645.8179	V	**1109.6929**	555.3501	1092.6663	546.8368	1091.6823	546.3448	11
12	**1421.7232**	711.3653	1403.7127	702.3600	L	1010.6245	505.8159	993.5979	497.3026	992.6139	496.8106	10
13	**1508.7553**	754.8813	1490.7447	745.8760	S	**897.5404**	449.2738	880.5138	440.7606	879.5298	440.2686	9
14	**1621.8393**	811.4233	1603.8288	802.4180	I	**810.5084**	405.7578	793.4818	397.2445	792.4978	396.7525	8
15	**1720.9078**	860.9575	1702.8972	851.9522	V	**697.4243**	349.2158	680.3978	340.7025	679.4137	340.2105	7
16	**1817.9605**	909.4839	1799.9500	900.4786	P	**598.3559**	299.6816	581.3293	291.1683	580.3453	290.6763	6
17	1904.9925	952.9999	1886.9820	943.9946	S	501.3031	251.1552	484.2766	242.6419	483.2926	242.1499	5

**18**	1962.0140	981.5106	1944.0034	972.5054	**G**	414.2711	207.6392	397.2445	199.1259			**4**
**19**	2059.0668	1030.0370	2041.0562	1021.0317	**P**	357.2496	179.1285	340.2231	170.6152			**3**
**20**	2172.1508	1086.5791	2154.1403	1077.5738	**L**	260.1969	130.6021	243.1703	122.0888			**2**
**21**					**K**	147.1128	74.0600	130.0863	65.5468			**1**

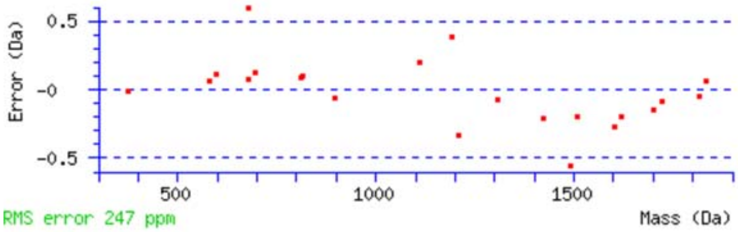

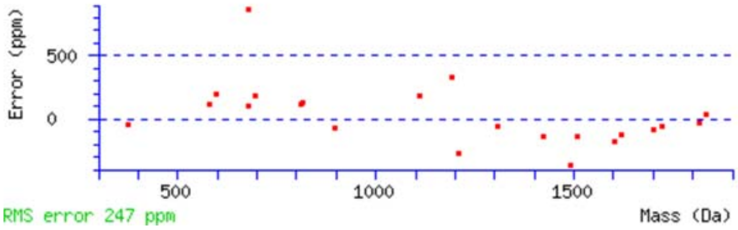

NCBI **BLAST** search of MSLLTEVETYVLSIVPSGPLK
(Parameters: blastp, nr protein database, expect=20000, no filter, PAM30)
Other BLAST web gateways

All matches to this query

Score	Mr(calc):	Delta	Sequence
56.4	2317.2491	-0.0012	MSLLTEVETYVLSIVPSGPLK
9.3	2317.2294	0.0185	QELQNLVAIPEKEKTKLQK
9.3	2317.2294	0.0185	QELQNLVAIPEKEKTKLQK
9.3	2317.2294	0.0185	QELQNLVAIPEKEKTKLQK
8.6	2317.2502	-0.0023	DPLLSQRISSLTASLHAHSRK
8.1	2317.2364	0.0115	KRVQIVGYLAWIISLGTIK
6.8	2317.2053	0.0426	QLAVGKQNGTVVQYLPTLQEK
6.8	2317.2053	0.0426	QLAVGKQNGTVVQYLPTLQEK
6.8	2317.2364	0.0115	KRVQIVGYLAWIISLGTIK
6.5	2317.2901	-0.0422	MLPLSLLKTAQNHPMLVELK

Mascot: http://www.matrixscience.com/

Mascot Search Results

Peptide View

MS/MS Fragmentation of **MSLLTEVETYVLSIVPSGPLKAEIAQR**
Found in **M1**

Match to Query 6470: 2943.599085 from(982.206971,3+)
Title: Elution from: 99.832 to 99.832 period: 0 experiment: 1 cycles: 1 precIntensity: 11664.0 FinneganScanNumber: 7886 MStype: enumIsNormalMS rawFile: B110210_012.RAW
Data file file1_B110210_012.mgf

Click mouse within plot area to zoom in by factor of two about that point
Or, Plot from 200 to 2000 Da Full range
Label all possible matches ○ Label matches used for scoring ◉

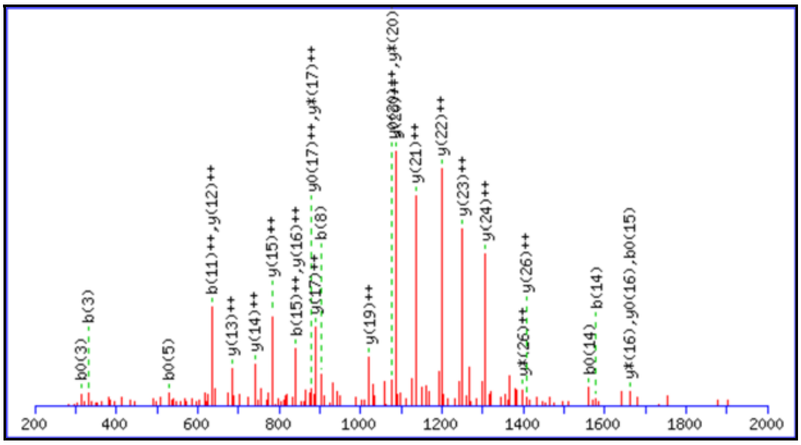

Monoisotopic mass of neutral peptide Mr(calc): 2943.5991
Fixed modifications: Carbamidomethyl (C) (apply to specified residues or termini only)
Ions Score: 86 **Expect:** 5.1e-07
Matches : 29/262 fragment ions using 32 most intense peaks (help)

#	b	b^{++}	b*	b^{*++}	b^0	b^{0++}	Seq.	y	y^{++}	y*	y^{*++}	y^0	y^{0++}	#
1	132.0478	66.5275					M							27
2	219.0798	110.0435			201.0692	101.0382	S	2813.5659	1407.2866	2796.5393	1398.7733	2795.5553	1398.2813	26
3	**332.1639**	166.5856			314.1533	157.5803	L	2726.5339	1363.7706	2709.5073	1355.2573	2708.5233	1354.7653	25
4	445.2479	223.1276			427.2374	214.1223	L	2613.4498	1307.2285	2596.4232	1298.7153	2595.4392	1298.2233	24
5	546.2956	273.6514			528.2850	264.6462	T	2500.3657	1250.6865	2483.3392	1242.1732	2482.3552	1241.6812	23
6	675.3382	338.1727			657.3276	329.1675	E	2399.3181	1200.1627	2382.2915	1191.6494	2381.3075	1191.1574	22
7	774.4066	387.7069			756.3960	378.7017	V	2270.2755	1135.6414	2253.2489	1127.1281	2252.2649	1126.6361	21
8	**903.4492**	452.2282			885.4386	443.2230	E	2171.2070	1086.1072	2154.1805	1077.5939	2153.1965	1077.1019	20
9	1004.4969	502.7521			986.4863	493.7468	T	2042.1645	1021.5859	2025.1379	1013.0726	2024.1539	1012.5806	19
10	1167.5602	584.2837			1149.5496	575.2785	Y	1941.1168	971.0620	1924.0902	962.5488	1923.1062	962.0567	18
11	1266.6286	633.8179			1248.6181	624.8127	V	1778.0534	889.5304	1761.0269	881.0171	1760.0429	880.5251	17
12	1379.7127	690.3600			1361.7021	681.3547	L	1678.9850	839.9962	1661.9585	831.4829	1660.9745	830.9909	16
13	1466.7447	733.8760			1448.7341	724.8707	S	1565.9010	783.4541	1548.8744	774.9408	1547.8904	774.4488	15
14	**1579.8288**	790.4180			1561.8182	781.4127	I	1478.8689	739.9381	1461.8424	731.4248	1460.8584	730.9328	14
15	1678.8972	839.9522			1660.8866	830.9469	V	1365.7849	683.3961	1348.7583	674.8828	1347.7743	674.3908	13
16	1775.9500	888.4786			1757.9394	879.4733	P	1266.7165	633.8619	1249.6899	625.3486	1248.7059	624.8566	12
17	1862.9820	931.9946			1844.9714	922.9893	S	1169.6637	585.3355	1152.6371	576.8222	1151.6531	576.3302	11
18	1920.0034	960.5054			1901.9929	951.5001	G	1082.6317	541.8195	1065.6051	533.3062	1064.6211	532.8142	10
19	2017.0562	1009.0317			1999.0456	1000.0265	P	1025.6102	513.3087	1008.5837	504.7955	1007.5996	504.3035	9
20	2130.1403	1065.5738			2112.1297	1056.5685	L	928.5574	464.7824	911.5309	456.2691	910.5469	455.7771	8
21	2258.2352	1129.6213	2241.2087	1121.1080	2240.2247	1120.6160	K	815.4734	408.2403	798.4468	399.7271	797.4628	399.2350	7
22	2329.2723	1165.1398	2312.2458	1156.6265	2311.2618	1156.1345	A	687.3784	344.1928	670.3519	335.6796	669.3679	335.1876	6
23	2458.3149	1229.6611	2441.2884	1221.1478	2440.3044	1220.6558	E	616.3413	308.6743	599.3148	300.1610	598.3307	299.6690	5
24	2571.3990	1286.2031	2554.3725	1277.6899	2553.3884	1277.1979	I	487.2987	244.1530	470.2722	235.6397			4
25	2642.4361	1321.7217	2625.4096	1313.2084	2624.4256	1312.7164	A	374.2146	187.6110	357.1881	179.0977			3
26	2770.4947	1385.7510	2753.4681	1377.2377	2752.4841	1376.7457	Q	303.1775	152.0924	286.1510	143.5791			2
27							R	175.1190	88.0631	158.0924	79.5498			1

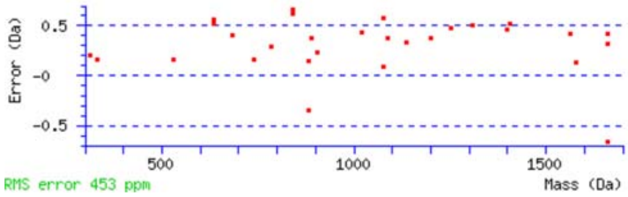

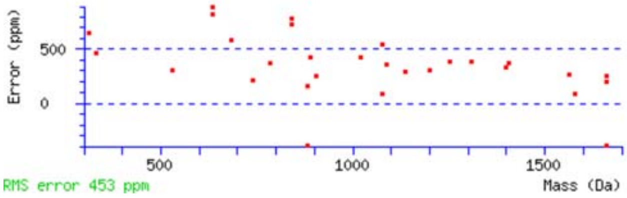

NCBI **BLAST** search of MSLLTEVETYVLSIVPSGPLKAEIAQR
(Parameters: blastp, nr protein database, expect=20000, no filter, PAM30)
Other BLAST web gateways

All matches to this query

Score	Mr(calc):	Delta	Sequence
85.6	2943.5991	0.0000	MSLLTEVETYVLSIVPSGPLKAEIAQR
19.5	2943.5655	0.0336	GSPVLADSAGRVRILSGGRQLQISVAEK
8.3	2943.6301	-0.0310	RMLQQQEQLRALQGRQAALLALQHK
6.8	2943.5948	0.0042	HVLILLENLYLLVPPTMNPIVYGVK
6.6	2943.5601	0.0389	NKLMSLSYSLVTPMLNPFIYTLRNK
6.6	2943.5601	0.0389	NKLMSLSYSLVTPMLNPFIYTLRNK
5.9	2943.5892	0.0099	AIALVGAVLQNSKVVDFIMQGSVPWRK
5.1	2943.6103	-0.0112	LTVQTKFMELLEPLNERKQNLLASK
4.6	2943.5892	0.0099	AIALVGAVLQNSKVVDFIMQGSVPWRK
4.6	2943.5826	0.0164	AALKWADGKMIKHEVDMQVLHLLGPK

Mascot: http://www.matrixscience.com/

Mascot Search Results

Peptide View

MS/MS Fragmentation of **MSLLTEVETYVLSIVPSGPLKAEIAQR**
Found in **M1**, Predicted protein of Fodor Group RG strain of A/WSN/33

Match to Query 3694: 2943.608604 from(982.210144,3+) intensity(58529.6000)
Title: B110905_011.06190.06190.3
Data file B110905_011.mgf.mascot

Click mouse within plot area to zoom in by factor of two about that point
Or, Plot from 300 to 1800 Da Full range
Label all possible matches ○ Label matches used for scoring ◉

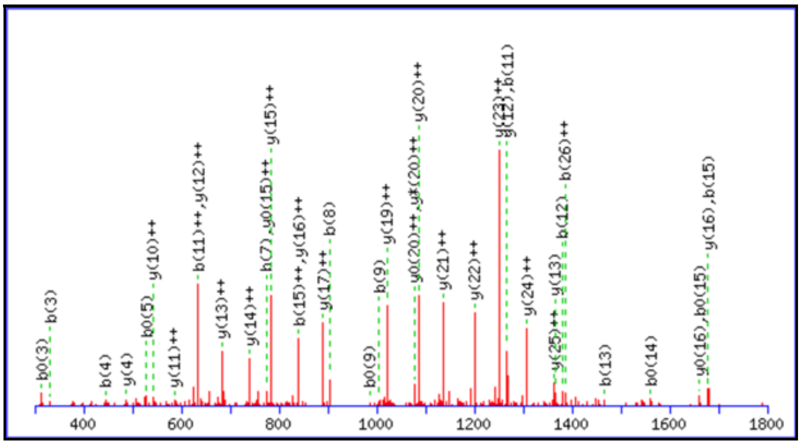

Monoisotopic mass of neutral peptide Mr(calc): 2943.5991
Fixed modifications: Carbamidomethyl (C) (apply to specified residues or termini only)
Ions Score: 92 **Expect:** 8.4e-09
Matches : 40/262 fragment ions using 57 most intense peaks (help)

#	b	b^{++}	b*	b^{*++}	b^0	b^{0++}	Seq.	y	y^{++}	y*	y^{*++}	y^0	y^{0++}	#
1	132.0478	66.5275					M							27
2	219.0798	110.0435			201.0692	101.0382	S	2813.5659	1407.2866	2796.5393	1398.7733	2795.5553	1398.2813	26
3	**332.1639**	166.5856			314.1533	157.5803	L	2726.5339	1363.7706	2709.5073	1355.2573	2708.5233	1354.7653	25
4	**445.2479**	223.1276			427.2374	214.1223	L	2613.4498	1307.2285	2596.4232	1298.7153	2595.4392	1298.2233	24
5	546.2956	273.6514			528.2850	264.6462	T	2500.3657	1250.6865	2483.3392	1242.1732	2482.3552	1241.6812	23
6	675.3382	338.1727			657.3276	329.1675	E	2399.3181	1200.1627	2382.2915	1191.6494	2381.3075	1191.1574	22
7	**774.4066**	387.7069			756.3960	378.7017	V	2270.2755	1135.6414	2253.2489	1127.1281	2252.2649	1126.6361	21
8	**903.4492**	452.2282			885.4386	443.2230	E	2171.2070	1086.1072	2154.1805	1077.5939	2153.1965	1077.1019	20
9	**1004.4969**	502.7521			986.4863	493.7468	T	2042.1645	1021.5859	2025.1379	1013.0726	2024.1539	1012.5806	19
10	1167.5602	584.2837			1149.5496	575.2785	Y	1941.1168	971.0620	1924.0902	962.5488	1923.1062	962.0567	18
11	**1266.6286**	**633.8179**			1248.6181	624.8127	V	1778.0534	889.5304	1761.0269	881.0171	1760.0429	880.5251	17
12	**1379.7127**	690.3600			1361.7021	681.3547	L	**1678.9850**	839.9962	1661.9585	831.4829	1660.9745	830.9909	16
13	**1466.7447**	733.8760			1448.7341	724.8707	S	1565.9010	783.4541	1548.8744	774.9408	1547.8904	774.4488	15
14	1579.8288	790.4180			1561.8182	781.4127	I	1478.8689	739.9381	1461.8424	731.4248	1460.8584	730.9328	14
15	**1678.8972**	**839.9522**			1660.8866	830.9469	V	**1365.7849**	683.3961	1348.7583	674.8828	1347.7743	674.3908	13
16	1775.9500	888.4786			1757.9394	879.4733	P	**1266.7165**	633.8619	1249.6899	625.3486	1248.7059	624.8566	12
17	1862.9820	931.9946			1844.9714	922.9893	S	1169.6637	585.3355	1152.6371	576.8222	1151.6531	576.3302	11
18	1920.0034	960.5054			1901.9929	951.5001	G	1082.6317	541.8195	1065.6051	533.3062	1064.6211	532.8142	10
19	2017.0562	1009.0317			1999.0456	1000.0265	P	1025.6102	513.3087	1008.5837	504.7955	1007.5996	504.3035	9
20	2130.1403	1065.5738			2112.1297	1056.5685	L	928.5574	464.7824	911.5309	456.2691	910.5469	455.7771	8
21	2258.2352	1129.6213	2241.2087	1121.1080	2240.2247	1120.6160	K	815.4734	408.2403	798.4468	399.7271	797.4628	399.2350	7
22	2329.2723	1165.1398	2312.2458	1156.6265	2311.2618	1156.1345	A	687.3784	344.1928	670.3519	335.6796	669.3679	335.1876	6
23	2458.3149	1229.6611	2441.2884	1221.1478	2440.3044	1220.6558	E	616.3413	308.6743	599.3148	300.1610	598.3307	299.6690	5
24	2571.3990	1286.2031	2554.3725	1277.6899	2553.3884	1277.1979	I	**487.2987**	244.1530	470.2722	235.6397			4
25	2642.4361	1321.7217	2625.4096	1313.2084	2624.4256	1312.7164	A	374.2146	187.6110	357.1881	179.0977			3
26	2770.4947	**1385.7510**	2753.4681	1377.2377	2752.4841	1376.7457	Q	303.1775	152.0924	286.1510	143.5791			2
27							R	175.1190	88.0631	158.0924	79.5498			1

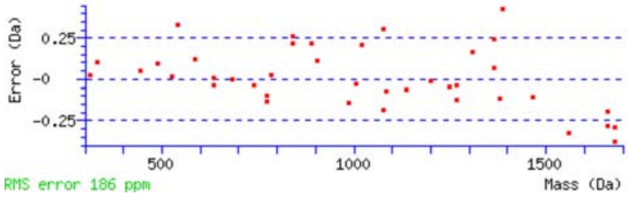

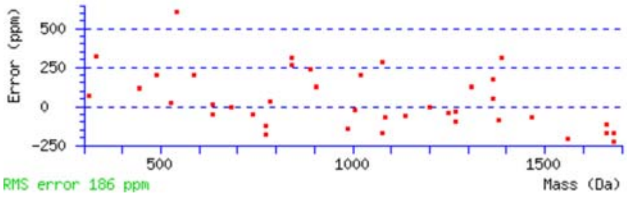

NCBI **BLAST** search of MSLLTEVETYVLSIVPSGPLKAEIAQR
(Parameters: blastp, nr protein database, expect=20000, no filter, PAM30)
Other BLAST web gateways

All matches to this query

Score	Mr(calc):	Delta	Sequence
92.2	2943.5991	0.0095	MSLLTEVETYVLSIVPSGPLKAEIAQR
5.7	2943.5601	0.0485	NKLMSLSYSLVTPMLNPFIYTLRNK
4.5	2941.5695	2.0391	KPGIIRCLVPQNKGISFSLAGELSDNK
3.3	2943.6099	-0.0013	LNVSPPPPSSPLPPPPLSPLPPLRRNK
2.2	2941.5490	2.0596	QSHRYEYSLLVLFFFINPIIAESR
1.7	2941.6188	1.9898	MGPRTLLLLLSGVLVLTETRAGSHSLK
1.6	2943.6586	-0.0500	SLLIFYVLVNSSQTLHNKILWSVLR
1.3	2943.6586	-0.0500	SLLIFYVLVNSSQTLHNKILWSVLR
1.2	2941.6307	1.9779	LQVLSLGFPGARILGALPNFPTLAAGLR
1.2	2941.6543	1.9543	KAIFLDMLGVRPFGFNEFLILLFLK

Mascot: http://www.matrixscience.com/

Mascot Search Results

Peptide View

MS/MS Fragmentation of **SLLTEVETYVLSIVPSGPLK**
Found in **M1**

Match to Query 4414: 2186.213164 from(1094.113858,2+)
Title: Elution from: 106.897 to 106.897 period: 0 experiment: 1 cycles: 1 precIntensity: 37516348.0 FinneganScanNumber: 8428 MStype: enumIsNormalMS rawFile: B110210_012.RAW
Data file file1_B110210_012.mgf

Click mouse within plot area to zoom in by factor of two about that point
Or, Plot from 300 to 1900 Da Full range
Label all possible matches ○ Label matches used for scoring ◉

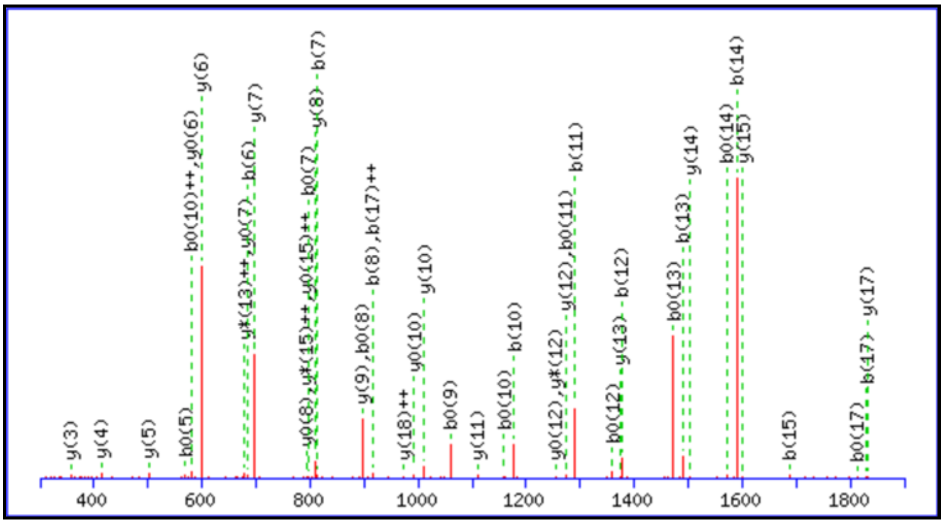

Monoisotopic mass of neutral peptide Mr(calc): 2186.2086
Fixed modifications: Carbamidomethyl (C) (apply to specified residues or termini only)
Variable modifications:
N-term : Acetyl (Protein N-term)
Ions Score: 122 **Expect:** 7.1e-11
Matches : 46/182 fragment ions using 46 most intense peaks (help)

#	b	b^{++}	b^0	b^{0++}	Seq.	y	y^{++}	y*	y^{*++}	y^0	y^{0++}	#
1	130.0499	65.5286	112.0393	56.5233	S							20
2	243.1339	122.0706	225.1234	113.0653	L	2058.1733	1029.5903	2041.1467	1021.0770	2040.1627	1020.5850	19
3	356.2180	178.6126	338.2074	169.6074	L	1945.0892	973.0482	1928.0627	964.5350	1927.0787	964.0430	18
4	457.2657	229.1365	439.2551	220.1312	T	1832.0052	916.5062	1814.9786	907.9929	1813.9946	907.5009	17
5	586.3083	293.6578	568.2977	284.6525	E	1730.9575	865.9824	1713.9309	857.4691	1712.9469	856.9771	16
6	685.3767	343.1920	667.3661	334.1867	V	1601.9149	801.4611	1584.8883	792.9478	1583.9043	792.4558	15
7	814.4193	407.7133	796.4087	398.7080	E	1502.8465	751.9269	1485.8199	743.4136	1484.8359	742.9216	14
8	915.4670	458.2371	897.4564	449.2318	T	1373.8039	687.4056	1356.7773	678.8923	1355.7933	678.4003	13
9	1078.5303	539.7688	1060.5197	530.7635	Y	1272.7562	636.8817	1255.7297	628.3685	1254.7456	627.8765	12
10	1177.5987	589.3030	1159.5881	580.2977	V	1109.6929	555.3501	1092.6663	546.8368	1091.6823	546.3448	11
11	1290.6828	645.8450	1272.6722	636.8397	L	1010.6245	505.8159	993.5979	497.3026	992.6139	496.8106	10
12	1377.7148	689.3610	1359.7042	680.3558	S	897.5404	449.2738	880.5138	440.7606	879.5298	440.2686	9
13	1490.7989	745.9031	1472.7883	736.8978	I	810.5084	405.7578	793.4818	397.2445	792.4978	396.7525	8
14	1589.8673	795.4373	1571.8567	786.4320	V	697.4243	349.2158	680.3978	340.7025	679.4137	340.2105	7
15	1686.9200	843.9637	1668.9095	834.9584	P	598.3559	299.6816	581.3293	291.1683	580.3453	290.6763	6
16	1773.9521	887.4797	1755.9415	878.4744	S	501.3031	251.1552	484.2766	242.6419	483.2926	242.1499	5
17	1830.9735	915.9904	1812.9630	906.9851	G	414.2711	207.6392	397.2445	199.1259			4

**18**	1928.0263	964.5168	1910.0157	955.5115	**P**	*357.2496*	179.1285	340.2231	170.6152			**3**
**19**	2041.1104	1021.0588	2023.0998	1012.0535	**L**	260.1969	130.6021	243.1703	122.0888			**2**
**20**					**K**	147.1128	74.0600	130.0863	65.5468			**1**

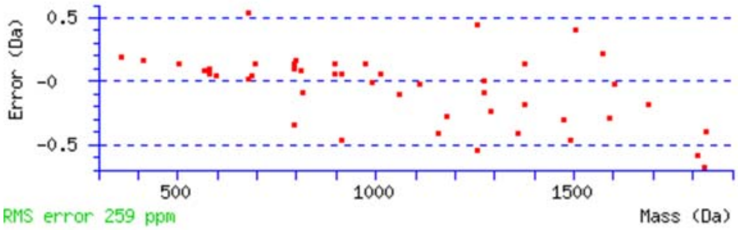

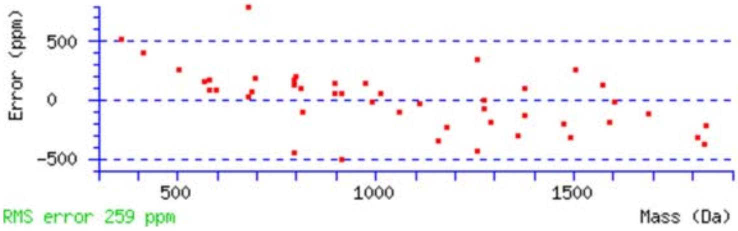

NCBI **BLAST** search of SLLTEVETYVLSIVPSGPLK
(Parameters: blastp, nr protein database, expect=20000, no filter, PAM30)
Other BLAST web gateways

All matches to this query

Score	Mr(calc):	Delta	Sequence
122.4	2186.2086	0.0046	SLLTEVETYVLSIVPSGPLK
25.6	2186.1881	0.0250	RSLITIENHLVTLKNFCK
17.2	2186.2205	-0.0073	GGNKILARSLMTQTLEAVKR
13.7	2186.2205	-0.0073	GGNKILARSLMTQTLEAVKR
11.8	2186.1993	0.0138	SLRPQNQIQKMVRSYIPK
8.8	2186.1993	0.0138	SLRPQNQIQKMVRSYIPK
7.4	2186.1919	0.0212	AERLRAELLDLNRGGFSIR
6.5	2186.1752	0.0380	IYSPHVLNLTLIDLPGITK
6.2	2186.1752	0.0380	IYSPHVLNLTLIDLPGITK
5.4	2186.2017	0.0115	ASVVSKLFFSWTRPILKK

Mascot: http://www.matrixscience.com/

Mascot Search Results

Peptide View

MS/MS Fragmentation of **SLLTEVETYVLSIVPSGPLKAEIAQR**
Found in **M1**

Match to Query 6270: 2854.574656 from(1428.294604,2+)
Title: Elution from: 104.552 to 104.552 period: 0 experiment: 1 cycles: 1 precIntensity: 65671.0 FinneganScanNumber: 8256 MStype: enumIsNormalMS rawFile: B110210_012.RAW
Data file file1_B110210_012.mgf

Click mouse within plot area to zoom in by factor of two about that point
Or, Plot from 300 to 2000 Da Full range
Label all possible matches Label matches used for scoring

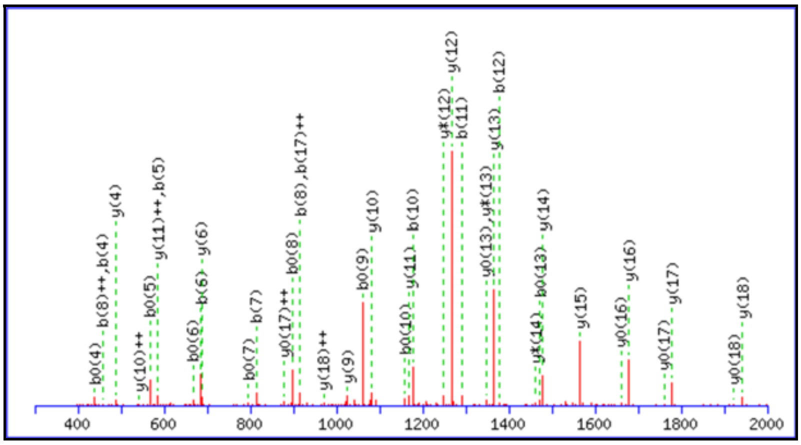

Monoisotopic mass of neutral peptide Mr(calc): 2854.5691
Fixed modifications: Carbamidomethyl (C) (apply to specified residues or termini only)
Variable modifications:
N-term : Acetyl (Protein N-term)
Ions Score: 85 **Expect:** 5.9e-08
Matches : 41/254 fragment ions using 49 most intense peaks (help)

#	b	b++	b*	b*++	b0	b0++	Seq.	y	y++	y*	y*++	y0	y0++	#
1	130.0499	65.5286			112.0393	56.5233	S							26
2	243.1339	122.0706			225.1234	113.0653	L	2726.5339	1363.7706	2709.5073	1355.2573	2708.5233	1354.7653	25
3	356.2180	178.6126			338.2074	169.6074	L	2613.4498	1307.2285	2596.4232	1298.7153	2595.4392	1298.2233	24
4	***457.2657***	229.1365			439.2551	220.1312	T	2500.3657	1250.6865	2483.3392	1242.1732	2482.3552	1241.6812	23
5	***586.3083***	293.6578			568.2977	284.6525	E	2399.3181	1200.1627	2382.2915	1191.6494	2381.3075	1191.1574	22
6	***685.3767***	343.1920			667.3661	334.1867	V	2270.2755	1135.6414	2253.2489	1127.1281	2252.2649	1126.6361	21
7	***814.4193***	407.7133			796.4087	398.7080	E	2171.2070	1086.1072	2154.1805	1077.5939	2153.1965	1077.1019	20
8	***915.4670***	458.2371			897.4564	449.2318	T	2042.1645	1021.5859	2025.1379	1013.0726	2024.1539	1012.5806	19
9	1078.5303	539.7688			1060.5197	530.7635	Y	***1941.1168***	971.0620	1924.0902	962.5488	1923.1062	962.0567	18
10	***1177.5987***	589.3030			1159.5881	580.2977	V	***1778.0534***	889.5304	1761.0269	881.0171	1760.0429	880.5251	17
11	***1290.6828***	645.8450			1272.6722	636.8397	L	***1678.9850***	839.9962	1661.9585	831.4829	1660.9745	830.9909	16
12	***1377.7148***	689.3610			1359.7042	680.3558	S	***1565.9010***	783.4541	1548.8744	774.9408	1547.8904	774.4488	15
13	1490.7989	745.9031			1472.7883	736.8978	I	***1478.8689***	739.9381	1461.8424	731.4248	1460.8584	730.9328	14
14	1589.8673	795.4373			1571.8567	786.4320	V	***1365.7849***	683.3961	1348.7583	674.8828	1347.7743	674.3908	13
15	1686.9200	843.9637			1668.9095	834.9584	P	***1266.7165***	633.8619	1249.6899	625.3486	1248.7059	624.8566	12
16	1773.9521	887.4797			1755.9415	878.4744	S	***1169.6637***	585.3355	1152.6371	576.8222	1151.6531	576.3302	11
17	1830.9735	915.9904			1812.9630	906.9851	G	***1082.6317***	541.8195	1065.6051	533.3062	1064.6211	532.8142	10
18	1928.0263	964.5168			1910.0157	955.5115	P	***1025.6102***	513.3087	1008.5837	504.7955	1007.5996	504.3035	9
19	2041.1104	1021.0588			2023.0998	1012.0535	L	928.5574	464.7824	911.5309	456.2691	910.5469	455.7771	8
20	2169.2053	1085.1063	2152.1788	1076.5930	2151.1948	1076.1010	K	815.4734	408.2403	798.4468	399.7271	797.4628	399.2350	7
21	2240.2424	1120.6249	2223.2159	1112.1116	2222.2319	1111.6196	A	***687.3784***	344.1928	670.3519	335.6796	669.3679	335.1876	6
22	2369.2850	1185.1461	2352.2585	1176.6329	2351.2745	1176.1409	E	616.3413	308.6743	599.3148	300.1610	598.3307	299.6690	5
23	2482.3691	1241.6882	2465.3425	1233.1749	2464.3585	1232.6829	I	***487.2987***	244.1530	470.2722	235.6397			4
24	2553.4062	1277.2067	2536.3797	1268.6935	2535.3956	1268.2015	A	374.2146	187.6110	357.1881	179.0977			3
25	2681.4648	1341.2360	2664.4382	1332.7228	2663.4542	1332.2307	Q	303.1775	152.0924	286.1510	143.5791			2
26							R	175.1190	88.0631	158.0924	79.5498			1

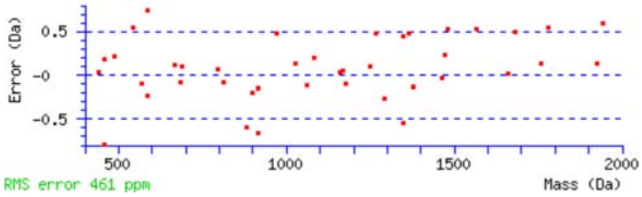

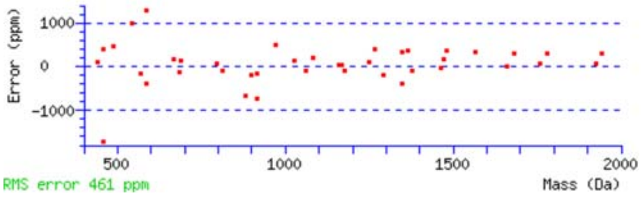

NCBI **BLAST** search of SLLTEVETYVLSIVPSGPLKAEIAQR
(Parameters: blastp, nr protein database, expect=20000, no filter, PAM30)
Other BLAST web gateways

All matches to this query

Score	Mr(calc):	Delta	Sequence
84.8	2854.5691	0.0055	SLLTEVETYVLSIVPSGPLKAEIAQR
7.3	2854.5683	0.0064	MLAKTVPDFSLLALKILQVLFVTR
7.1	2854.5375	0.0372	QGLSRLGISLLGAIAGIVDQPMQNFQK
7.1	2854.5375	0.0372	QGLSRLGISLLGAIAGIVDQPMQNFQK
3.6	2854.5489	0.0257	VVMAWVAVLWLTAMAEGLQVTVPDKK
3.2	2854.5566	0.0181	RNAPAPHPSRSSPLPLVSSAQAFVLVR
2.7	2854.5189	0.0558	KPPVPQKLTLVAPPGGPNGDSSGGTQGVAK
2.3	2854.5237	0.0510	SDRLLKVLFLVLGYTVLAGMVFIK
2.2	2854.5189	0.0558	KPPVPQKLTLVAPPGGPNGDSSGGTQGVAK
2.2	2854.5189	0.0558	KPPVPQKLTLVAPPGGPNGDSSGGTQGVAK

Mascot: http://www.matrixscience.com/

{MATRIX SCIENCE} Mascot Search Results

Peptide View

MS/MS Fragmentation of **SLLTEVETYVLSIVPSGPLKAEIAQRLEDVFAGK**
Found in **M1**, Predicted proteins of Fodor Group RG strain of A/WSN/33

Match to Query 5312: 3714.033150 from(1239.018326,3+)
Title: Elution from: 107.990 to 107.990 period: 0 experiment: 1 cycles: 1 precIntensity: 63818.0 FinneganScanNumber: 6576 MStype: enumIsNormalMS rawFile: B110602_036.RAW
Data file file1_B110602_036.mgf

Click mouse within plot area to zoom in by factor of two about that point
Or, Plot from 300 to 2000 Da Full range
Label all possible matches ○ Label matches used for scoring ◉

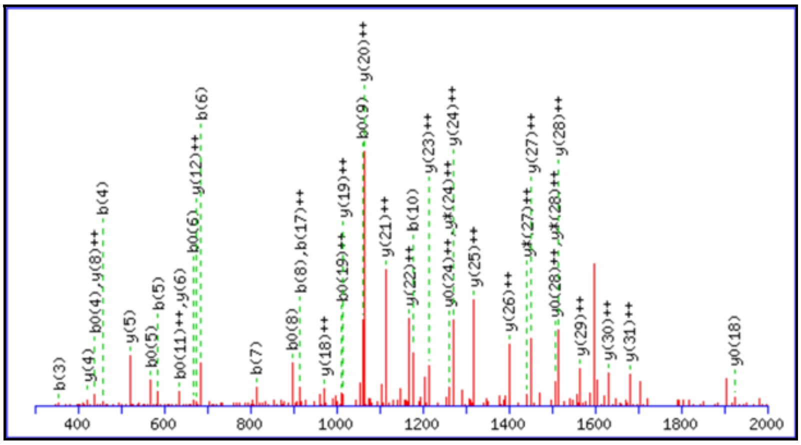

Monoisotopic mass of neutral peptide Mr(calc): 3714.0131
Fixed modifications: Carbamidomethyl (C) (apply to specified residues or termini only)
Variable modifications:
N-term : Acetyl (Protein N-term)
Ions Score: 107 **Expect:** 4.6e-10
Matches : 40/348 fragment ions using 51 most intense peaks (help)

#	b	b^{++}	b*	b^{*++}	b^0	b^{0++}	Seq.	y	y^{++}	y*	y^{*++}	y^0	y^{0++}	#
1	130.0499	65.5286			112.0393	56.5233	S							34
2	243.1339	122.0706			225.1234	113.0653	L	3585.9778	1793.4926	3568.9513	1784.9793	3567.9673	1784.4873	33
3	**356.2180**	178.6126			338.2074	169.6074	L	3472.8938	1736.9505	3455.8672	1728.4372	3454.8832	1727.9452	32
4	**457.2657**	229.1365			439.2551	220.1312	T	3359.8097	1680.4085	3342.7832	1671.8952	3341.7991	1671.4032	31
5	**586.3083**	293.6578			568.2977	284.6525	E	3258.7620	1629.8846	3241.7355	1621.3714	3240.7515	1620.8794	30
6	**685.3767**	343.1920			667.3661	334.1867	V	3129.7194	1565.3634	3112.6929	1556.8501	3111.7089	1556.3581	29
7	**814.4193**	407.7133			796.4087	398.7080	E	3030.6510	1515.8291	3013.6245	1507.3159	3012.6405	1506.8239	28
8	**915.4670**	458.2371			897.4564	449.2318	T	2901.6084	1451.3078	2884.5819	1442.7946	2883.5979	1442.3026	27
9	1078.5303	539.7688			1060.5197	530.7635	Y	2800.5607	1400.7840	2783.5342	1392.2707	2782.5502	1391.7787	26
10	**1177.5987**	589.3030			1159.5881	580.2977	V	2637.4974	1319.2523	2620.4709	1310.7391	2619.4869	1310.2471	25
11	1290.6828	645.8450			1272.6722	636.8397	L	2538.4290	1269.7181	2521.4025	1261.2049	2520.4184	1260.7129	24
12	1377.7148	689.3610			1359.7042	680.3558	S	2425.3449	1213.1761	2408.3184	1204.6628	2407.3344	1204.1708	23
13	1490.7989	745.9031			1472.7883	736.8978	I	2338.3129	1169.6601	2321.2864	1161.1468	2320.3023	1160.6548	22
14	1589.8673	795.4373			1571.8567	786.4320	V	2225.2288	1113.1181	2208.2023	1104.6048	2207.2183	1104.1128	21
15	1686.9200	843.9637			1668.9095	834.9584	P	2126.1604	1063.5839	2109.1339	1055.0706	2108.1499	1054.5786	20
16	1773.9521	887.4797			1755.9415	878.4744	S	2029.1077	1015.0575	2012.0811	1006.5442	2011.0971	1006.0522	19
17	1830.9735	915.9904			1812.9630	906.9851	G	1942.0756	971.5415	1925.0491	963.0282	1924.0651	962.5362	18
18	1928.0263	964.5168			1910.0157	955.5115	P	1885.0542	943.0307	1868.0276	934.5175	1867.0436	934.0254	17
19	2041.1104	1021.0588			2023.0998	1012.0535	L	1788.0014	894.5043	1770.9749	885.9911	1769.9908	885.4991	16
20	2169.2053	1085.1063	2152.1788	1076.5930	2151.1948	1076.1010	K	1674.9173	837.9623	1657.8908	829.4490	1656.9068	828.9570	15
21	2240.2424	1120.6249	2223.2159	1112.1116	2222.2319	1111.6196	A	1546.8224	773.9148	1529.7958	765.4016	1528.8118	764.9095	14
22	2369.2850	1185.1461	2352.2585	1176.6329	2351.2745	1176.1409	E	1475.7853	738.3963	1458.7587	729.8830	1457.7747	729.3910	13
23	2482.3691	1241.6882	2465.3425	1233.1749	2464.3585	1232.6829	I	1346.7427	673.8750	1329.7161	665.3617	1328.7321	664.8697	12
24	2553.4062	1277.2067	2536.3797	1268.6935	2535.3956	1268.2015	A	1233.6586	617.3329	1216.6321	608.8197	1215.6480	608.3277	11
25	2681.4648	1341.2360	2664.4382	1332.7228	2663.4542	1332.2307	Q	1162.6215	581.8144	1145.5950	573.3011	1144.6109	572.8091	10
26	2837.5659	1419.2866	2820.5393	1410.7733	2819.5553	1410.2813	R	1034.5629	517.7851	1017.5364	509.2718	1016.5524	508.7798	9

**27**	2950.6500	1475.8286	2933.6234	1467.3153	2932.6394	1466.8233	**L**	878.4618	439.7345	861.4353	431.2213	860.4512	430.7293	**8**
**28**	3079.6925	1540.3499	3062.6660	1531.8366	3061.6820	1531.3446	**E**	765.3777	383.1925	748.3512	374.6792	747.3672	374.1872	**7**
**29**	3194.7195	1597.8634	3177.6929	1589.3501	3176.7089	1588.8581	**D**	**636.3352**	318.6712	619.3086	310.1579	618.3246	309.6659	**6**
**30**	3293.7879	1647.3976	3276.7614	1638.8843	3275.7773	1638.3923	**V**	**521.3082**	261.1577	504.2817	252.6445			**5**
**31**	3440.8563	1720.9318	3423.8298	1712.4185	3422.8458	1711.9265	**F**	**422.2398**	211.6235	405.2132	203.1103			**4**
**32**	3511.8934	1756.4504	3494.8669	1747.9371	3493.8829	1747.4451	**A**	275.1714	138.0893	258.1448	129.5761			**3**
**33**	3568.9149	1784.9611	3551.8883	1776.4478	3550.9043	1775.9558	**G**	204.1343	102.5708	187.1077	94.0575			**2**
**34**							**K**	147.1128	74.0600	130.0863	65.5468			**1**

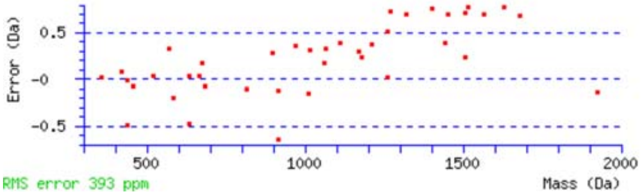

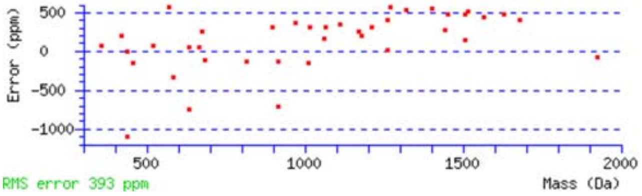

NCBI **BLAST** search of SLLTEVETYVLSIVPSGPLKAEIAQRLEDVFAGK
(Parameters: blastp, nr protein database, expect=20000, no filter, PAM30)
Other BLAST web gateways

All matches to this query

Score	Mr(calc):	Delta	Sequence
107.3	3714.0131	0.0200	SLLTEVETYVLSIVPSGPLKAEIAQRLEDVFAGK
7.2	3713.9764	0.0567	QPKYNTLYGNMSDSDITTYNSDGESDR
7.2	3713.9764	0.0567	QPKYNTLYGNMSDSDITTYNSDGESDR
7.2	3713.9764	0.0567	QPKYNTLYGNMSDSDITTYNSDGESDR
6.1	3714.1041	-0.0710	HAVLGLYLLVFLILVGIFILAVSRPRSSPDDLK
4.1	3713.9831	0.0501	IXDADSLAVGRFSVEGPPGETPLPLVLKKLLLSR
4.1	3714.0161	0.0170	IXDADSLAVGRFSVEGPPGETPLPLVLKKLLLSR
3.9	3714.1041	-0.0710	HAVLGLYLLVFLILVGIFILAVSRPRSSPDDLK
3.9	3713.9797	0.0535	TPSVVYRQRLYELLILLSPETYEGNLSVILR
3.9	3713.9797	0.0535	TPSVVYRQRLYELLILLSPETYEGNLSVILR

Mascot: http://www.matrixscience.com/

Mascot Search Results

Peptide View

MS/MS Fragmentation of SLLTEVETYVLSIVPSGPLKAEIAQRLEDVFAGKNTDLEVLMEWLK
Found in M1, Predicted proteins of Fodor Group RG strain of A/WSN/33

Match to Query 5717: 5187.799036 from(1297.957035,4+)
Title: Elution from: 108.993 to 108.993 period: 0 experiment: 1 cycles: 1 precIntensity: 15909.0 FinneganScanNumber: 6633 MStype: enumIsNormalMS rawFile: B110602_036.RAW
Data file file1_B110602_036.mgf

Click mouse within plot area to zoom in by factor of two about that point
Or, Plot from 300 to 2000 Da Full range
Label all possible matches ○ Label matches used for scoring ⦿

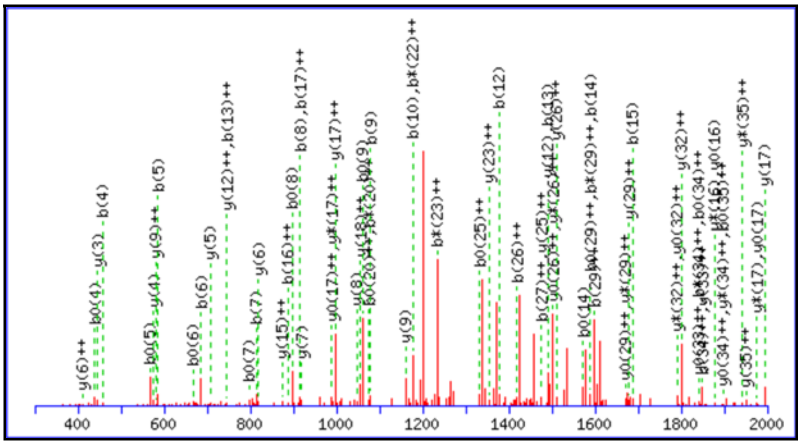

Monoisotopic mass of neutral peptide Mr(calc): 5187.7192
Fixed modifications: Carbamidomethyl (C) (apply to specified residues or termini only)
Variable modifications:
N-term : Acetyl (Protein N-term)
Q25 : Deamidated (NQ)
N35 : Deamidated (NQ)
Ions Score: 57 **Expect:** 9e-05
Matches : 73/496 fragment ions using 107 most intense peaks (help)

#	b	b++	b*	b*++	b0	b0++	Seq.	y	y++	y*	y*++	y0	y0++	#
1	130.0499	65.5286			112.0393	56.5233	S							46
2	243.1339	122.0706			225.1234	113.0653	L	5059.6840	2530.3456	5042.6574	2521.8323	5041.6734	2521.3403	45
3	356.2180	178.6126			338.2074	169.6074	L	4946.5999	2473.8036	4929.5734	2465.2903	4928.5893	2464.7983	44
4	457.2657	229.1365			439.2551	220.1312	T	4833.5158	2417.2616	4816.4893	2408.7483	4815.5053	2408.2563	43
5	586.3083	293.6578			568.2977	284.6525	E	4732.4682	2366.7377	4715.4416	2358.2244	4714.4576	2357.7324	42
6	685.3767	343.1920			667.3661	334.1867	V	4603.4256	2302.2164	4586.3990	2293.7031	4585.4150	2293.2111	41
7	814.4193	407.7133			796.4087	398.7080	E	4504.3572	2252.6822	4487.3306	2244.1689	4486.3466	2243.6769	40
8	915.4670	458.2371			897.4564	449.2318	T	4375.3146	2188.1609	4358.2880	2179.6476	4357.3040	2179.1556	39
9	1078.5303	539.7688			1060.5197	530.7635	Y	4274.2669	2137.6371	4257.2403	2129.1238	4256.2563	2128.6318	38
10	1177.5987	589.3030			1159.5881	580.2977	V	4111.2035	2056.1054	4094.1770	2047.5921	4093.1930	2047.1001	37
11	1290.6828	645.8450			1272.6722	636.8397	L	4012.1351	2006.5712	3995.1086	1998.0579	3994.1246	1997.5659	36
12	1377.7148	689.3610			1359.7042	680.3558	S	3899.0511	1950.0292	3882.0245	1941.5159	3881.0405	1941.0239	35
13	1490.7989	745.9031			1472.7883	736.8978	I	3812.0190	1906.5132	3794.9925	1897.9999	3794.0085	1897.5079	34
14	1589.8673	795.4373			1571.8567	786.4320	V	3698.9350	1849.9711	3681.9084	1841.4579	3680.9244	1840.9658	33
15	1686.9200	843.9637			1668.9095	834.9584	P	3599.8666	1800.4369	3582.8400	1791.9236	3581.8560	1791.4316	32
16	1773.9521	887.4797			1755.9415	878.4744	S	3502.8138	1751.9105	3485.7873	1743.3973	3484.8032	1742.9053	31
17	1830.9735	915.9904			1812.9630	906.9851	G	3415.7818	1708.3945	3398.7552	1699.8812	3397.7712	1699.3892	30
18	1928.0263	964.5168			1910.0157	955.5115	P	3358.7603	1679.8838	3341.7338	1671.3705	3340.7497	1670.8785	29
19	2041.1104	1021.0588			2023.0998	1012.0535	L	3261.7075	1631.3574	3244.6810	1622.8441	3243.6970	1622.3521	28
20	2169.2053	1085.1063	2152.1788	1076.5930	2151.1948	1076.1010	K	3148.6235	1574.8154	3131.5969	1566.3021	3130.6129	1565.8101	27
21	2240.2424	1120.6249	2223.2159	1112.1116	2222.2319	1111.6196	A	3020.5285	1510.7679	3003.5020	1502.2546	3002.5180	1501.7626	26
22	2369.2850	1185.1461	2352.2585	1176.6329	2351.2745	1176.1409	E	2949.4914	1475.2493	2932.4649	1466.7361	2931.4808	1466.2441	25
23	2482.3691	1241.6882	2465.3425	1233.1749	2464.3585	1232.6829	I	2820.4488	1410.7280	2803.4223	1402.2148	2802.4382	1401.7228	24
24	2553.4062	1277.2067	2536.3797	1268.6935	2535.3956	1268.2015	A	2707.3647	1354.1860	2690.3382	1345.6727	2689.3542	1345.1807	23
25	2682.4488	1341.7280	2665.4222	1333.2148	2664.4382	1332.7228	Q	2636.3276	1318.6675	2619.3011	1310.1542	2618.3171	1309.6622	22

26	2838.5499	1419.7786	2821.5234	1411.2653	2820.5393	1410.7733	R	2507.2850	1254.1462	2490.2585	1245.6329	2489.2745	1245.1409	21
27	2951.6340	1476.3206	2934.6074	1467.8073	2933.6234	1467.3153	L	2351.1839	1176.0956	2334.1574	1167.5823	2333.1734	1167.0903	20
28	3080.6766	1540.8419	3063.6500	1532.3286	3062.6660	1531.8366	E	2238.0999	1119.5536	2221.0733	1111.0403	2220.0893	1110.5483	19
29	3195.7035	1598.3554	3178.6770	1589.8421	3177.6929	1589.3501	D	2109.0573	1055.0323	2092.0307	1046.5190	2091.0467	1046.0270	18
30	3294.7719	1647.8896	3277.7454	1639.3763	3276.7614	1638.8843	V	1994.0303	997.5188	1977.0038	989.0055	1976.0198	988.5135	17
31	3441.8403	1721.4238	3424.8138	1712.9105	3423.8298	1712.4185	F	1894.9619	947.9846	1877.9354	939.4713	1876.9513	938.9793	16
32	3512.8774	1756.9424	3495.8509	1748.4291	3494.8669	1747.9371	A	1747.8935	874.4504	1730.8670	865.9371	1729.8829	865.4451	15
33	3569.8989	1785.4531	3552.8724	1776.9398	3551.8883	1776.4478	G	1676.8564	838.9318	1659.8298	830.4186	1658.8458	829.9265	14
34	3697.9939	1849.5006	3680.9673	1840.9873	3679.9833	1840.4953	K	1619.8349	810.4211	1602.8084	801.9078	1601.8244	801.4158	13
35	3813.0208	1907.0140	3795.9943	1898.5008	3795.0103	1898.0088	N	1491.7400	746.3736	1474.7134	737.8603	1473.7294	737.3683	12
36	3914.0685	1957.5379	3897.0419	1949.0246	3896.0579	1948.5326	T	1376.7130	688.8601	1359.6865	680.3469	1358.7025	679.8549	11
37	4029.0954	2015.0514	4012.0689	2006.5381	4011.0849	2006.0461	D	1275.6653	638.3363	1258.6388	629.8230	1257.6548	629.3310	10
38	4142.1795	2071.5934	4125.1530	2063.0801	4124.1689	2062.5881	L	1160.6384	580.8228	1143.6118	572.3096	1142.6278	571.8176	9
39	4271.2221	2136.1147	4254.1955	2127.6014	4253.2115	2127.1094	E	1047.5543	524.2808	1030.5278	515.7675	1029.5438	515.2755	8
40	4370.2905	2185.6489	4353.2640	2177.1356	4352.2799	2176.6436	V	918.5117	459.7595	901.4852	451.2462	900.5012	450.7542	7
41	4483.3746	2242.1909	4466.3480	2233.6777	4465.3640	2233.1856	L	819.4433	410.2253	802.4168	401.7120	801.4328	401.2200	6
42	4614.4151	2307.7112	4597.3885	2299.1979	4596.4045	2298.7059	M	706.3593	353.6833	689.3327	345.1700	688.3487	344.6780	5
43	4743.4577	2372.2325	4726.4311	2363.7192	4725.4471	2363.2272	E	575.3188	288.1630	558.2922	279.6498	557.3082	279.1577	4
44	4929.5370	2465.2721	4912.5104	2456.7588	4911.5264	2456.2668	W	446.2762	223.6417	429.2496	215.1285			3
45	5042.6210	2521.8142	5025.5945	2513.3009	5024.6105	2512.8089	L	260.1969	130.6021	243.1703	122.0888			2
46							K	147.1128	74.0600	130.0863	65.5468			1

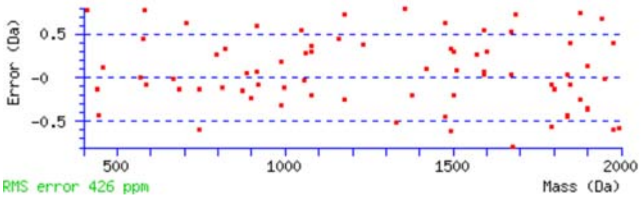

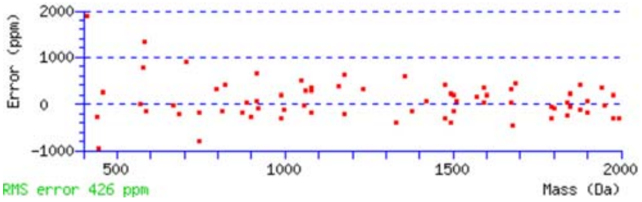

NCBI **BLAST** search of SLLTEVETYVLSIVPSGPLKAEIAQRLEDVFAGKNTDLEVLMEWLK
(Parameters: blastp, nr protein database, expect=20000, no filter, PAM30)
Other BLAST web gateways

All matches to this query

Score	Mr(calc):	Delta	Sequence
56.8	5187.7192	0.0798	SLLTEVETYVLSIVPSGPLKAEIAQRLEDVFAGKNTDLEVLMEWLK
13.2	5187.7865	0.0125	ASKLTQVLRDSFIGQNSSTCMIATISPGMASCENTLNTLR
13.1	5187.7868	0.0123	RWGLSSLSQNHNSEPDSQNFSQESEGRNTGPWLSSSLRNR
12.8	5187.7111	0.0879	ATNGSGQATSTAELLVTAETAPPNFTQRLQSMTVRQGSQVR
12.3	5187.7694	0.0296	NWNEGTLSAQSSESGIEEELHRESGQCQMERDGSPSFSKSR
12.0	5187.6988	0.1002	QGQSSHYDETDRHGLSTRYSQTDRQGVNSQYGQSETGETR
11.5	5187.8549	-0.0558	QMDPLNRPNAEGDSDKYICTTSDNLASVCSPSQLQDDENIDR
11.5	5187.7608	0.0382	EPAEPWASQAALQEDVDMSGGSSGNETSENGSTGRDSQGSDCGDSGR
10.9	5187.7911	0.0079	EAEQAAGEAASGDTTPGSSPSSLYYEEPLGQPPRFTQKLRSR
10.8	5187.8108	-0.0118	NDKGCSLGSALLSPTSASPATSSSTVTTAKTSAVVSFMSNSPLR

Mascot: http://www.matrixscience.com/

(MATRIX SCIENCE) Mascot Search Results

Peptide View

MS/MS Fragmentation of **SLLTEVETPIR**
Found in **M2**, Predicted proteins of Fodor Group RG strain of A/WSN/33

Match to Query 1253: 1256.704088 from(629.359320,2+)
Title: Elution from: 32.037 to 32.037 period: 0 experiment: 1 cycles: 1 precIntensity: 1051617.0 FinneganScanNumber: 2214 MStype: enumIsNormalMS rawFile: B110602_036.RAW
Data file file1_B110602_036.mgf

Click mouse within plot area to zoom in by factor of two about that point
Or, Plot from 150 to 1200 Da Full range
Label all possible matches ○ Label matches used for scoring ◉

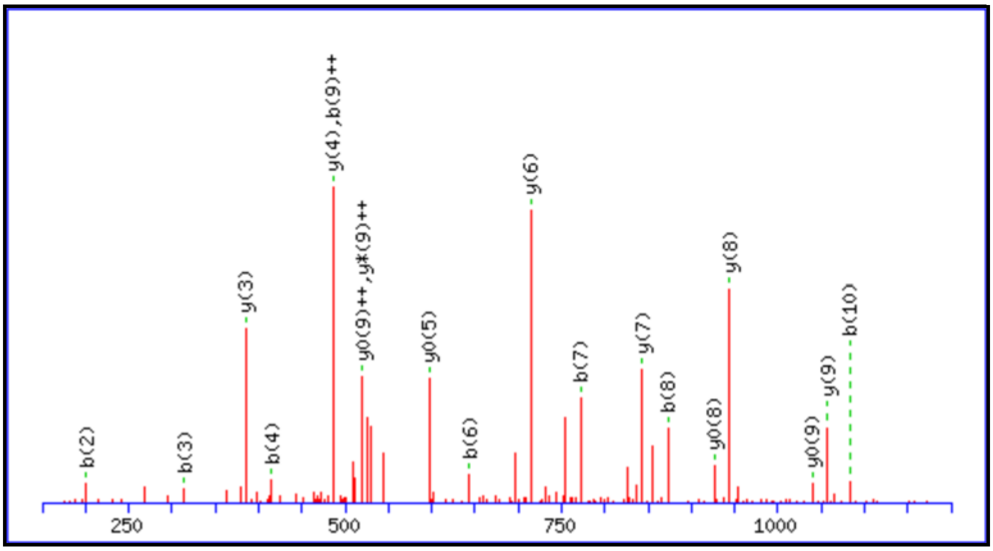

Monoisotopic mass of neutral peptide Mr(calc): 1256.6976
Fixed modifications: Carbamidomethyl (C) (apply to specified residues or termini only)
Ions Score: 65 **Expect:** 1.3e-05
Matches : 19/94 fragment ions using 20 most intense peaks (help)

#	b	b^{++}	b^{0}	b^{0++}	Seq.	y	y^{++}	y*	y^{*++}	y^{0}	y^{0++}	#
1	88.0393	44.5233	70.0287	35.5180	S							11
2	201.1234	101.0653	183.1128	92.0600	L	1170.6729	585.8401	1153.6463	577.3268	1152.6623	576.8348	10
3	314.2074	157.6074	296.1969	148.6021	L	1057.5888	529.2980	1040.5623	520.7848	1039.5782	520.2928	9
4	415.2551	208.1312	397.2445	199.1259	T	944.5047	472.7560	927.4782	464.2427	926.4942	463.7507	8
5	544.2977	272.6525	526.2871	263.6472	E	843.4571	422.2322	826.4305	413.7189	825.4465	413.2269	7
6	643.3661	322.1867	625.3556	313.1814	V	714.4145	357.7109	697.3879	349.1976	696.4039	348.7056	6
7	772.4087	386.7080	754.3981	377.7027	E	615.3461	308.1767	598.3195	299.6634	597.3355	299.1714	5
8	873.4564	437.2318	855.4458	428.2266	T	486.3035	243.6554	469.2769	235.1421	468.2929	234.6501	4
9	970.5092	485.7582	952.4986	476.7529	P	385.2558	193.1315	368.2292	184.6183			3
10	1083.5932	542.3002	1065.5827	533.2950	I	288.2030	144.6051	271.1765	136.0919			2
11					R	175.1190	88.0631	158.0924	79.5498			1

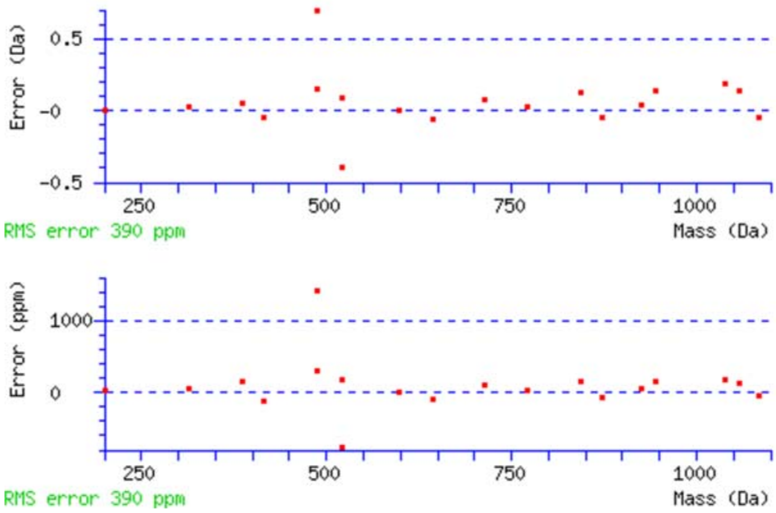

NCBI **BLAST** search of SLLTEVETPIR
(Parameters: blastp, nr protein database, expect=20000, no filter, PAM30)
Other BLAST web gateways

All matches to this query

Score	Mr(calc):	Delta	Sequence
64.8	1256.6976	0.0065	SLLTEVETPIR
12.0	1256.6949	0.0092	LSLIGDRRGDR
10.9	1256.6837	0.0204	SAISEVVQRAR
10.6	1256.6976	0.0065	TPSVTQVTPAKK
9.9	1256.7088	-0.0048	SLIVAGLGDGSIR
8.3	1256.6837	0.0204	QAREERLVQK
7.4	1256.7088	-0.0047	AEIEKLKAAQR
7.4	1256.7281	-0.0240	TVLIFFFSKR
7.4	1256.7088	-0.0047	TVLQLPSKSER
6.7	1256.7088	-0.0047	LQTVALSQQIR

Mascot: http://www.matrixscience.com/

Mascot Search Results

Peptide View

MS/MS Fragmentation of **SLLTEVETPIR**
Found in **M2**, Predicted proteins of Fodor Group RG strain of A/WSN/33

Match to Query 1328: 1298.714508 from(650.364530,2+)
Title: Elution from: 48.605 to 48.605 period: 0 experiment: 1 cycles: 1 precIntensity: 999360.0 FinneganScanNumber: 3241 MStype: enumIsNormalMS rawFile: B110602_036.RAW
Data file file1_B110602_036.mgf

Click mouse within plot area to zoom in by factor of two about that point
Or, Plot from 100 to 1300 Da Full range
Label all possible matches ○ Label matches used for scoring ◉

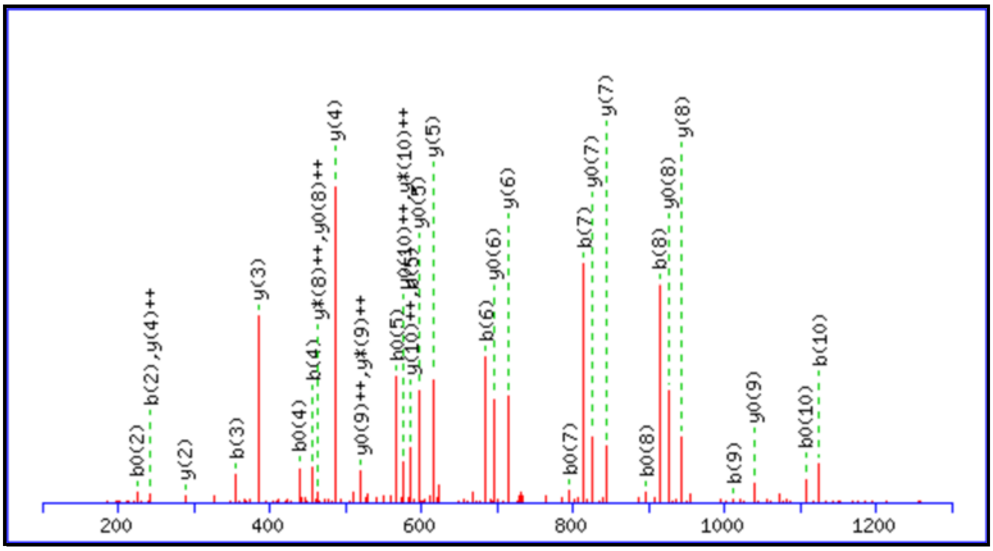


```
Monoisotopic mass of neutral peptide Mr(calc): 1298.7082
Fixed modifications: Carbamidomethyl (C) (apply to specified residues or termini only)
Variable modifications:
N-term : Acetyl (Protein N-term)
Ions Score: 70  Expect: 6.2e-06
Matches : 35/94 fragment ions using 44 most intense peaks   (help)
```

#	b	b^{++}	b^{0}	b^{0++}	Seq.	y	y^{++}	y*	y^{*++}	y^{0}	y^{0++}	#
1	130.0499	65.5286	112.0393	56.5233	S							11
2	**243.1339**	122.0706	225.1234	113.0653	L	1170.6729	585.8401	1153.6463	577.3268	1152.6623	576.8348	10
3	**356.2180**	178.6126	338.2074	169.6074	L	1057.5888	529.2980	1040.5623	520.7848	1039.5782	520.2928	9
4	**457.2657**	229.1365	439.2551	220.1312	T	**944.5047**	472.7560	927.4782	464.2427	926.4942	463.7507	8
5	**586.3083**	293.6578	568.2977	284.6525	E	**843.4571**	422.2322	826.4305	413.7189	825.4465	413.2269	7
6	**685.3767**	343.1920	667.3661	334.1867	V	**714.4145**	357.7109	697.3879	349.1976	696.4039	348.7056	6
7	**814.4193**	407.7133	796.4087	398.7080	E	**615.3461**	308.1767	598.3195	299.6634	597.3355	299.1714	5
8	**915.4670**	458.2371	897.4564	449.2318	T	**486.3035**	243.6554	469.2769	235.1421	468.2929	234.6501	4
9	**1012.5197**	506.7635	994.5092	497.7582	P	**385.2558**	193.1315	368.2292	184.6183			3
10	**1125.6038**	563.3055	1107.5932	554.3002	I	**288.2030**	144.6051	271.1765	136.0919			2
11					R	175.1190	88.0631	158.0924	79.5498			1

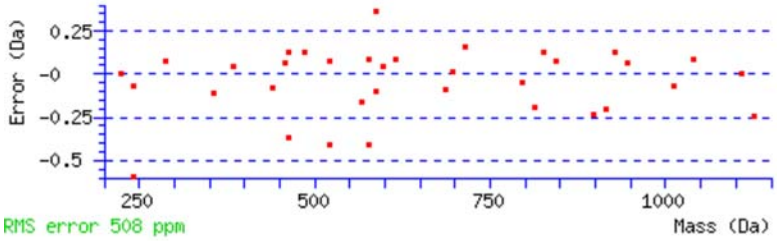

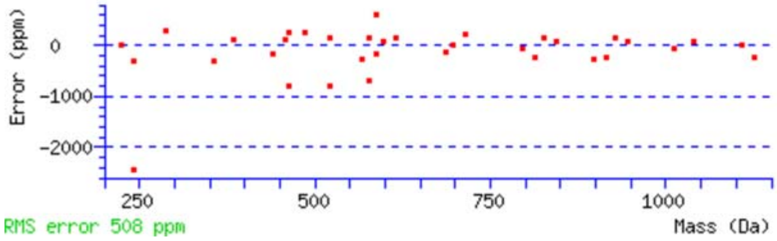

NCBI **BLAST** search of SLLTEVETPIR
(Parameters: blastp, nr protein database, expect=20000, no filter, PAM30)
Other BLAST web gateways

All matches to this query

Score	Mr(calc):	Delta	Sequence
70.1	1298.7082	0.0063	SLLTEVETPIR
13.7	1298.6943	0.0202	VVEGTPLTDGRR
11.5	1298.7234	-0.0089	FLLQVQSPPLR
11.2	1298.7167	-0.0022	RDLGSRLQAQR
8.8	1298.7306	-0.0161	LQGDAAVLRISR
7.7	1298.6942	0.0203	IQENVRNAIGGK
7.5	1298.7194	-0.0049	DLASVSLKNPVR
7.4	1298.6999	0.0146	TLIQFTVKLR
7.0	1298.6943	0.0202	VQEEIDRVVGR
7.0	1298.6942	0.0203	VQEEINRVVGR

Mascot: http://www.matrixscience.com/

Mascot Search Results

Peptide View

MS/MS Fragmentation of **MDPNTVSSFQVDCFLWHVR**
Found in **NS1**

Match to Query 5125: 2379.075384 from(1190.544968,2+)
Title: Elution from: 68.492 to 68.492 period: 0 experiment: 1 cycles: 1 precIntensity: 1018488.0 FinneganScanNumber: 5396 MStype: enumIsNormalMS rawFile: B110210_012.RAW
Data file file1_B110210_012.mgf

Click mouse within plot area to zoom in by factor of two about that point
Or, Plot from 300 to 2000 Da Full range
Label all possible matches ○ Label matches used for scoring ◉

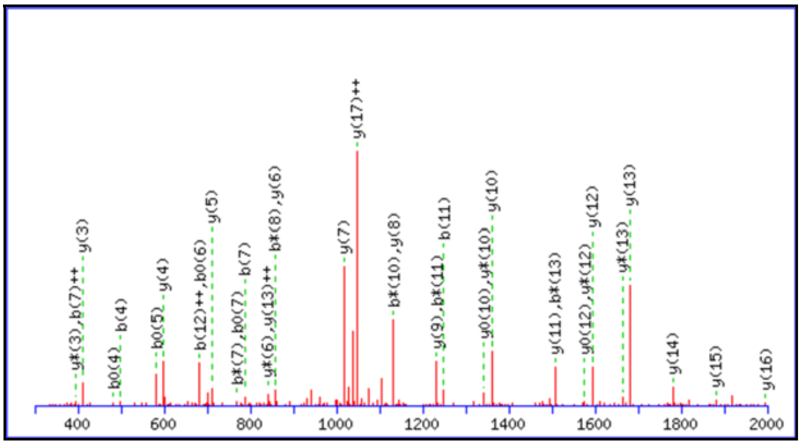

Monoisotopic mass of neutral peptide Mr(calc): 2379.0777
Fixed modifications: Carbamidomethyl (C) (apply to specified residues or termini only)
Variable modifications:
N-term : Acetyl (Protein N-term)
Ions Score: 117 **Expect:** 2.1e-10
Matches : 37/194 fragment ions using 34 most intense peaks (help)

#	b	b^{++}	b*	b^{*++}	b^0	b^{0++}	Seq.	y	y^{++}	y*	y^{*++}	y^0	y^{0++}	#
1	174.0583	87.5328					M							19
2	289.0853	145.0463			271.0747	136.0410	D	2207.0338	1104.0206	2190.0073	1095.5073	2189.0233	1095.0153	18
3	386.1380	193.5727			368.1275	184.5674	P	2092.0069	1046.5071	2074.9804	1037.9938	2073.9963	1037.5018	17
4	**500.1810**	250.5941	483.1544	242.0808	482.1704	241.5888	N	***1994.9541***	997.9807	1977.9276	989.4674	1976.9436	988.9754	16
5	601.2286	301.1180	584.2021	292.6047	583.2181	292.1127	T	***1880.9112***	940.9592	1863.8847	932.4460	1862.9007	931.9540	15
6	700.2971	350.6522	683.2705	342.1389	682.2865	341.6469	V	***1779.8635***	890.4354	1762.8370	881.9221	1761.8530	881.4301	14
7	**787.3291**	394.1682	770.3025	385.6549	769.3185	385.1629	S	***1680.7951***	840.9012	1663.7686	832.3879	1662.7846	831.8959	13
8	874.3611	437.6842	857.3346	429.1709	856.3505	428.6789	S	***1593.7631***	797.3852	1576.7365	788.8719	1575.7525	788.3799	12
9	1021.4295	511.2184	1004.4030	502.7051	1003.4190	502.2131	F	***1506.7311***	753.8692	1489.7045	745.3559	1488.7205	744.8639	11
10	1149.4881	575.2477	1132.4616	566.7344	1131.4775	566.2424	Q	***1359.6627***	680.3350	1342.6361	671.8217	1341.6521	671.3297	10
11	**1248.5565**	624.7819	1231.5300	616.2686	1230.5459	615.7766	V	***1231.6041***	616.3057	1214.5775	607.7924	1213.5935	607.3004	9
12	1363.5835	682.2954	1346.5569	673.7821	1345.5729	673.2901	D	***1132.5357***	566.7715	1115.5091	558.2582	1114.5251	557.7662	8
13	1523.6141	762.3107	1506.5876	753.7974	1505.6035	753.3054	C	***1017.5087***	509.2580	1000.4822	500.7447			7
14	1670.6825	835.8449	1653.6560	827.3316	1652.6720	826.8396	F	***857.4781***	429.2427	840.4515	420.7294			6
15	1783.7666	892.3869	1766.7400	883.8737	1765.7560	883.3816	L	***710.4097***	355.7085	693.3831	347.1952			5
16	1969.8459	985.4266	1952.8193	976.9133	1951.8353	976.4213	W	***597.3256***	299.1664	580.2990	290.6532			4
17	2106.9048	1053.9560	2089.8783	1045.4428	2088.8942	1044.9508	H	***411.2463***	206.1268	394.2197	197.6135			3
18	2205.9732	1103.4902	2188.9467	1094.9770	2187.9627	1094.4850	V	274.1874	137.5973	257.1608	129.0840			2
19							R	175.1190	88.0631	158.0924	79.5498			1

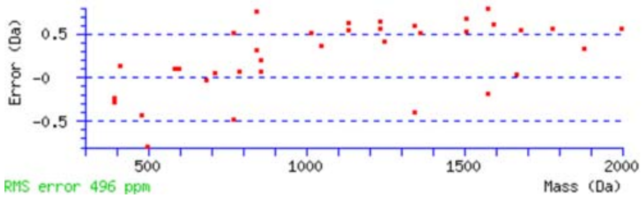

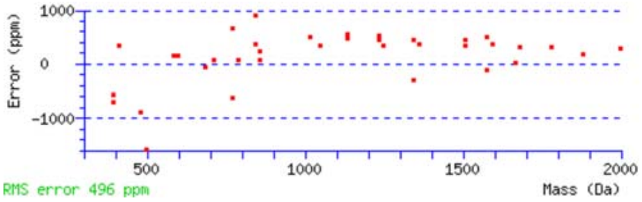

NCBI **BLAST** search of MDPNTVSSFQVDCFLWHVR
(Parameters: blastp, nr protein database, expect=20000, no filter, PAM30)
Other BLAST web gateways

All matches to this query

Score	Mr(calc):	Delta	Sequence
117.3	2379.0777	-0.0023	MDPNTVSSFQVDCFLWHVR
13.4	2379.0817	-0.0063	RMNLSDYLSSNGSFPLEQLK
13.4	2379.0817	-0.0063	RMNLSDYLSSNGSFPLEQLK
11.6	2379.0912	-0.0159	NAYGITPLFVAAQSGQLEALR
10.7	2379.0930	-0.0176	MVNPGSSSQPPPVTAGSLSWKR
10.5	2379.0665	0.0089	KMSTHVSSSPTSQIYSLSSEK
10.3	2379.0912	-0.0159	NAYGITPLFVAAQSGQLEALR
10.0	2379.0665	0.0089	KMSTHVSSSPTSQIYSLSSEK
10.0	2379.0930	-0.0176	MVNPGSSSQPPPVTAGSLSWKR
10.0	2379.0930	-0.0176	MVNPGSSSQPPPVTAGSLSWKR

Mascot: http://www.matrixscience.com/

Mascot Search Results

Peptide View

MS/MS Fragmentation of **MDPNTVSSFQDILMR**
Found in **NEP**, Predicted protein of Fodor Group RG strain of A/WSN/33

Match to Query 1107: 1794.832860 from(898.423706,2+) intensity(1192190.0000)
Title: B110905_011.03375.03375.2
Data file B110905_011.mgf.mascot

Click mouse within plot area to zoom in by factor of two about that point
Or, Plot from 200 to 1700 Da Full range
Label all possible matches ○ Label matches used for scoring ◉

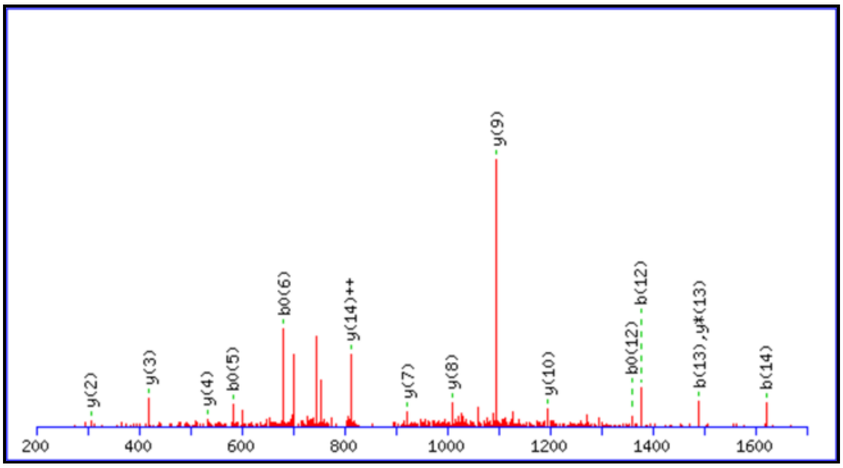

Monoisotopic mass of neutral peptide Mr(calc): 1794.8280
Fixed modifications: Carbamidomethyl (C) (apply to specified residues or termini only)
Variable modifications:
N-term : Acetyl (Protein N-term)
Ions Score: 61 **Expect:** 1.8e-05
Matches : 15/152 fragment ions using 14 most intense peaks (help)

#	b	b++	b*	b*++	b0	b0++	Seq.	y	y++	y*	y*++	y0	y0++	#
1	174.0583	87.5328					M							15
2	289.0853	145.0463			271.0747	136.0410	D	1622.7843	811.8958	1605.7577	803.3825	1604.7737	802.8905	14
3	386.1380	193.5727			368.1275	184.5674	P	1507.7573	754.3823	1490.7308	745.8690	1489.7468	745.3770	13
4	500.1810	250.5941	483.1544	242.0808	482.1704	241.5888	N	1410.7046	705.8559	1393.6780	697.3427	1392.6940	696.8506	12
5	601.2286	301.1180	584.2021	292.6047	583.2181	292.1127	T	1296.6616	648.8345	1279.6351	640.3212	1278.6511	639.8292	11
6	700.2971	350.6522	683.2705	342.1389	682.2865	341.6469	V	***1195.6140***	598.3106	1178.5874	589.7973	1177.6034	589.3053	10
7	787.3291	394.1682	770.3025	385.6549	769.3185	385.1629	S	***1096.5456***	548.7764	1079.5190	540.2631	1078.5350	539.7711	9
8	874.3611	437.6842	857.3346	429.1709	856.3505	428.6789	S	***1009.5135***	505.2604	992.4870	496.7471	991.5030	496.2551	8
9	1021.4295	511.2184	1004.4030	502.7051	1003.4190	502.2131	F	***922.4815***	461.7444	905.4550	453.2311	904.4709	452.7391	7
10	1149.4881	575.2477	1132.4616	566.7344	1131.4775	566.2424	Q	775.4131	388.2102	758.3865	379.6969	757.4025	379.2049	6
11	1264.5150	632.7612	1247.4885	624.2479	1246.5045	623.7559	D	647.3545	324.1809	630.3280	315.6676	629.3439	315.1756	5
12	**1377.5991**	689.3032	1360.5726	680.7899	1359.5885	680.2979	I	***532.3276***	266.6674	515.3010	258.1541			4
13	**1490.6832**	745.8452	1473.6566	737.3319	1472.6726	736.8399	L	***419.2435***	210.1254	402.2170	201.6121			3
14	**1621.7237**	811.3655	1604.6971	802.8522	1603.7131	802.3602	M	***306.1594***	153.5834	289.1329	145.0701			2
15							R	175.1190	88.0631	158.0924	79.5498			1

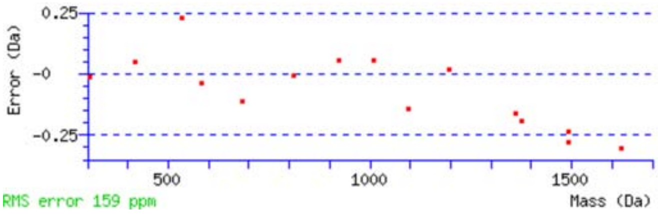

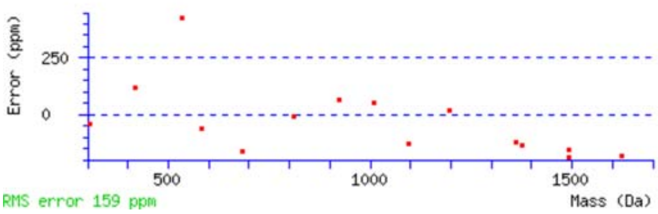

NCBI **BLAST** search of MDPNTVSSFQDILMR
(Parameters: blastp, nr protein database, expect=20000, no filter, PAM30)

Other BLAST web gateways

All matches to this query

Score	Mr(calc):	Delta	Sequence
61.5	1794.8280	0.0048	MDPNTVSSFQDILMR
10.2	1794.8392	-0.0064	NPEISHMLNNPELMR
10.2	1794.8392	-0.0064	NPEISHMLNNPELMR
9.0	1794.8416	-0.0087	EVKYFSFPGELLMR
8.7	1793.8237	1.0092	AIYALGHQELSTQPVS
7.9	1794.8375	-0.0047	IGALNSHMTQILPFR
7.7	1794.8119	0.0210	VLELSASSSDGEESTER
7.3	1792.8140	2.0188	MNGLVTSSPEMFKLK
7.3	1792.8140	2.0188	MNGLVTSSPEMFKLK
7.3	1794.8416	-0.0087	EVKYFSFPGELLMR

Mascot: http://www.matrixscience.com/

NIB74xp (egg-grown)
N-terminal peptide spectra

Mascot Search Results

Peptide View

MS/MS Fragmentation of **MDVNPTLLFLK**
Found in **PB1**, NIB74xp

Match to Query 1365: 1347.711400 from(674.862976,2+) intensity(22311.4000)
Title: B111206_005.04406.04406.2
Data file B111206_005.mgf.mascot

Click mouse within plot area to zoom in by factor of two about that point
Or, Plot from 250 to 1250 Da Full range
Label all possible matches ○ Label matches used for scoring ◉

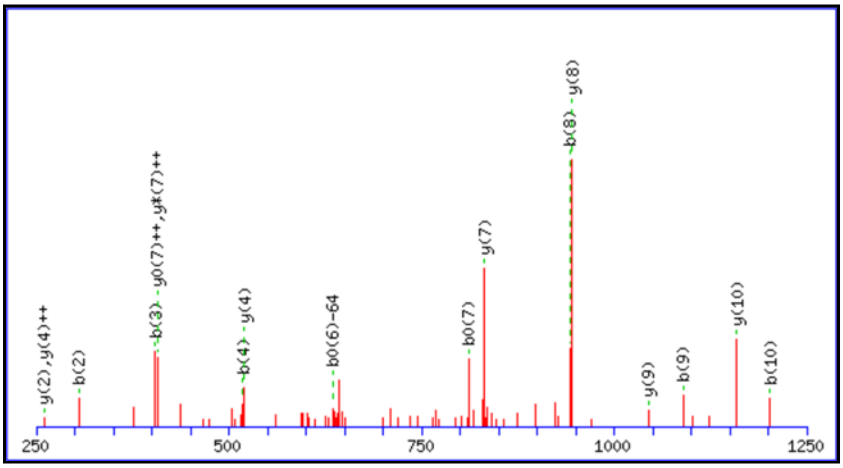

Monoisotopic mass of neutral peptide Mr(calc): 1347.7108
Fixed modifications: Carbamidomethyl (C) (apply to specified residues or termini only)
Variable modifications:
N-term : Acetyl (Protein N-term)
M1 : Oxidation (M), with neutral losses 0.0000(shown in table), 63.9983
Ions Score: 58 **Expect:** 3.1e-05
Matches : 18/154 fragment ions using 19 most intense peaks (help)

#	b	b^{++}	b*	b^{*++}	b^0	b^{0++}	Seq.	y	y^{++}	y*	y^{*++}	y^0	y^{0++}	#
1	190.0532	95.5303					M							11
2	***305.0802***	153.0437			287.0696	144.0384	D	***1159.6721***	580.3397	1142.6456	571.8264	1141.6616	571.3344	10
3	***404.1486***	202.5779			386.1380	193.5727	V	***1044.6452***	522.8262	1027.6186	514.3130	1026.6346	513.8210	9
4	***518.1915***	259.5994	501.1650	251.0861	500.1810	250.5941	N	***945.5768***	473.2920	928.5502	464.7788	927.5662	464.2867	8
5	615.2443	308.1258	598.2177	299.6125	597.2337	299.1205	P	***831.5339***	416.2706	814.5073	407.7573	813.5233	407.2653	7
6	716.2920	358.6496	699.2654	350.1363	698.2814	349.6443	T	734.4811	367.7442	717.4545	359.2309	716.4705	358.7389	6
7	829.3760	415.1917	812.3495	406.6784	811.3655	406.1864	L	633.4334	317.2203	616.4069	308.7071			5
8	***942.4601***	471.7337	925.4335	463.2204	924.4495	462.7284	L	***520.3493***	260.6783	503.3228	252.1650			4
9	***1089.5285***	545.2679	1072.5020	536.7546	1071.5179	536.2626	F	***407.2653***	204.1363	390.2387	195.6230			3
10	***1202.6126***	601.8099	1185.5860	593.2967	1184.6020	592.8046	L	***260.1969***	130.6021	243.1703	122.0888			2
11							K	147.1128	74.0600	130.0863	65.5468			1

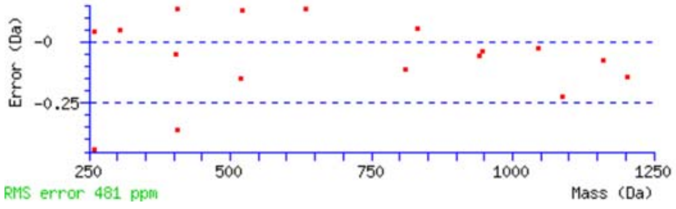

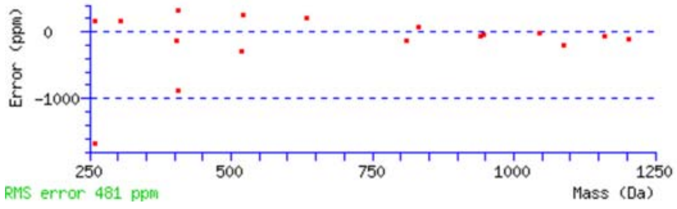

NCBI **BLAST** search of MDVNPTLLFLK
(Parameters: blastp, nr protein database, expect=20000, no filter, PAM30)
Other BLAST web gateways

All matches to this query

Score	Mr(calc):	Delta	Sequence
57.5	1347.7108	0.0006	MDVNPTLLFLK
8.9	1347.6881	0.0233	NTAGTIGKSQELK
6.5	1347.6955	0.0159	NGTILVDNMLIK
4.9	1345.7248	1.9866	QATVMRQNVKR
4.9	1347.7254	-0.0140	MKIMGEVLNGIK
4.5	1345.7136	1.9978	MRPTTVENVKR
3.8	1347.6922	0.0192	AALDFVVEEDLK
3.7	1347.7034	0.0080	EVRQLAEDFLK
3.0	1347.7068	0.0046	SGDPKDTKMLIK
3.0	1347.6951	0.0163	YRISKYPTLK

Mascot: http://www.matrixscience.com/

Mascot Search Results

Peptide View

MS/MS Fragmentation of **SLLTEVETYVLSIIPSGPLK**
Found in **M1**, NIB74xp

Match to Query 3093: 2158.209570 from(1080.112061,2+) intensity(7589.7200)
Title: B111206_005.06447.06447.2
Data file B111206_005.mgf.mascot

Click mouse within plot area to zoom in by factor of two about that point
Or, Plot from 500 to 1600 Da Full range
Label all possible matches ○ Label matches used for scoring ◉

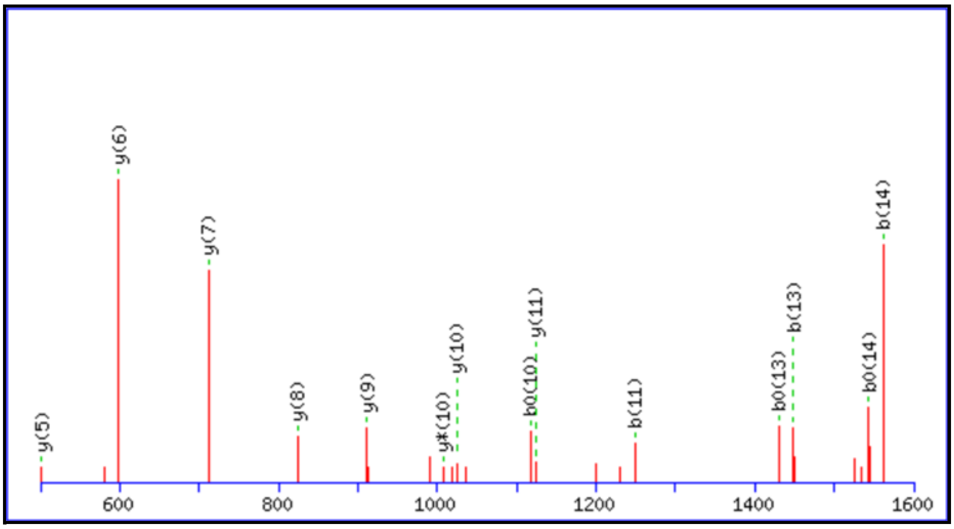

Monoisotopic mass of neutral peptide Mr(calc): 2158.2137
Fixed modifications: Carbamidomethyl (C) (apply to specified residues or termini only)
Ions Score: 54 **Expect:** 0.00027
Matches : 14/182 fragment ions using 17 most intense peaks (help)

#	b	b^{++}	b^0	b^{0++}	Seq.	y	y^{++}	y*	y^{*++}	y^0	y^{0++}	#
1	88.0393	44.5233	70.0287	35.5180	S							20
2	201.1234	101.0653	183.1128	92.0600	L	2072.1889	1036.5981	2055.1624	1028.0848	2054.1784	1027.5928	19
3	314.2074	157.6074	296.1969	148.6021	L	1959.1049	980.0561	1942.0783	971.5428	1941.0943	971.0508	18
4	415.2551	208.1312	397.2445	199.1259	T	1846.0208	923.5140	1828.9943	915.0008	1828.0102	914.5088	17
5	544.2977	272.6525	526.2871	263.6472	E	1744.9731	872.9902	1727.9466	864.4769	1726.9626	863.9849	16
6	643.3661	322.1867	625.3556	313.1814	V	1615.9305	808.4689	1598.9040	799.9556	1597.9200	799.4636	15
7	772.4087	386.7080	754.3981	377.7027	E	1516.8621	758.9347	1499.8356	750.4214	1498.8516	749.9294	14
8	873.4564	437.2318	855.4458	428.2266	T	1387.8195	694.4134	1370.7930	685.9001	1369.8090	685.4081	13
9	1036.5197	518.7635	1018.5092	509.7582	Y	1286.7719	643.8896	1269.7453	635.3763	1268.7613	634.8843	12
10	1135.5881	568.2977	1117.5776	559.2924	V	***1123.7085***	562.3579	1106.6820	553.8446	1105.6980	553.3526	11
11	**1248.6722**	624.8397	1230.6616	615.8345	L	***1024.6401***	512.8237	1007.6136	504.3104	1006.6295	503.8184	10
12	1335.7042	668.3558	1317.6937	659.3505	S	***911.5560***	456.2817	894.5295	447.7684	893.5455	447.2764	9
13	**1448.7883**	724.8978	1430.7777	715.8925	I	***824.5240***	412.7656	807.4975	404.2524	806.5135	403.7604	8
14	**1561.8724**	781.4398	1543.8618	772.4345	I	***711.4400***	356.2236	694.4134	347.7103	693.4294	347.2183	7
15	1658.9251	829.9662	1640.9146	820.9609	P	***598.3559***	299.6816	581.3293	291.1683	580.3453	290.6763	6
16	1745.9571	873.4822	1727.9466	864.4769	S	***501.3031***	251.1552	484.2766	242.6419	483.2926	242.1499	5
17	1802.9786	901.9929	1784.9680	892.9877	G	414.2711	207.6392	397.2445	199.1259			4
18	1900.0314	950.5193	1882.0208	941.5140	P	357.2496	179.1285	340.2231	170.6152			3
19	2013.1154	1007.0614	1995.1049	998.0561	L	260.1969	130.6021	243.1703	122.0888			2

**20**					**K**	147.1128	74.0600	130.0863	65.5468			**1**

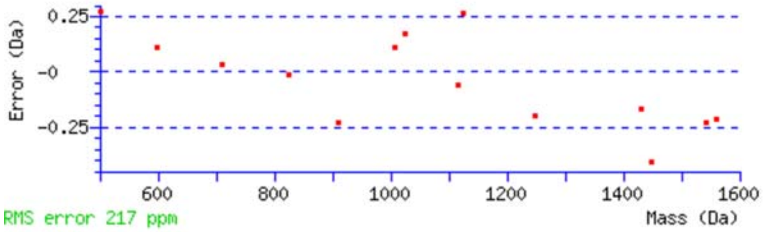

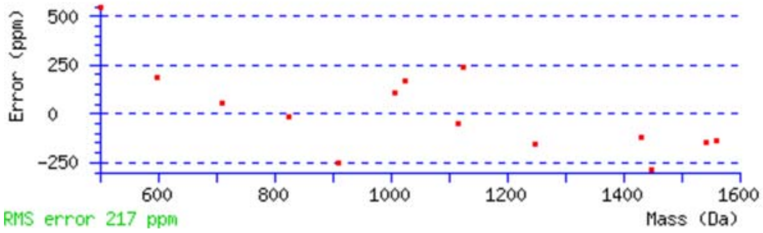

NCBI **BLAST** search of SLLTEVETYVLSIIPSGPLK
(Parameters: blastp, nr protein database, expect=20000, no filter, PAM30)
Other BLAST web gateways

All matches to this query

Score	Mr(calc):	Delta	Sequence
53.7	2158.2137	-0.0041	SLLTEVETYVLSIIPSGPLK
18.3	2158.1971	0.0125	QGVVRGSRPPVSRALPQPEK
12.6	2157.2131	0.9965	QGVVRGSRPPVSRALPQPEK
9.8	2158.1972	0.0123	WRVRLAIIEYMPLLAGQL
8.8	2158.2435	-0.0339	QVITIIILLLLCKASDFR
6.0	2157.1727	1.0369	KKEVLIAGLKPSLAVYLR
6.0	2157.1714	1.0381	NAKPVSILRDLITEAMEIK
4.8	2158.2126	-0.0030	IAQGISGLVRPLLSGLKVSK
4.4	2158.1779	0.0316	KNLQLLRDMQTIQTSLQK
4.4	2158.1779	0.0316	KNLQLLRDMQTIQTSLQK

Mascot: http://www.matrixscience.com/

Mascot Search Results

Peptide View

MS/MS Fragmentation of **SLLTEVETYVLSIIPSGPLK**
Found in **M1**, NIB74xp

Match to Query 3151: 2200.227392 from(1101.120972,2+) intensity(1417000.0000)
Title: B111206_005.06676.06676.2
Data file B111206_005.mgf.mascot

Click mouse within plot area to zoom in by factor of two about that point
Or, Plot from 300 to 1900 Da Full range
Label all possible matches ○ Label matches used for scoring ◉

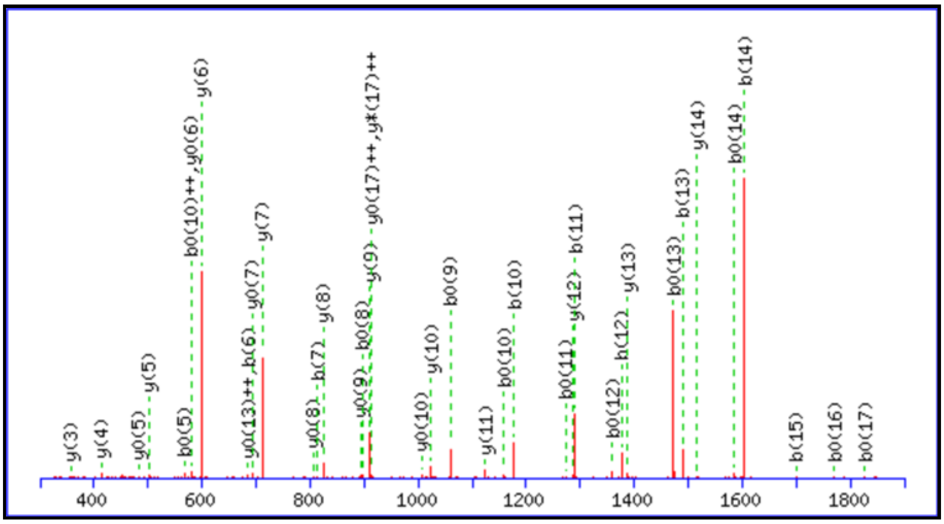

Monoisotopic mass of neutral peptide Mr(calc): 2200.2242
Fixed modifications: Carbamidomethyl (C) (apply to specified residues or termini only)
Variable modifications:
N-term : Acetyl (Protein N-term)
Ions Score: 78 **Expect:** 8.6e-07
Matches : 40/182 fragment ions using 59 most intense peaks (help)

#	b	b^{++}	b^0	b^{0++}	Seq.	y	y^{++}	y*	y^{*++}	y^0	y^{0++}	#
1	130.0499	65.5286	112.0393	56.5233	S							20
2	243.1339	122.0706	225.1234	113.0653	L	2072.1889	1036.5981	2055.1624	1028.0848	2054.1784	1027.5928	19
3	356.2180	178.6126	338.2074	169.6074	L	1959.1049	980.0561	1942.0783	971.5428	1941.0943	971.0508	18
4	457.2657	229.1365	439.2551	220.1312	T	1846.0208	923.5140	1828.9943	915.0008	1828.0102	914.5088	17
5	586.3083	293.6578	568.2977	284.6525	E	1744.9731	872.9902	1727.9466	864.4769	1726.9626	863.9849	16
6	**685.3767**	343.1920	667.3661	334.1867	V	1615.9305	808.4689	1598.9040	799.9556	1597.9200	799.4636	15
7	**814.4193**	407.7133	796.4087	398.7080	E	***1516.8621***	758.9347	1499.8356	750.4214	1498.8516	749.9294	14
8	915.4670	458.2371	897.4564	449.2318	T	***1387.8195***	694.4134	1370.7930	685.9001	1369.8090	685.4081	13
9	1078.5303	539.7688	1060.5197	530.7635	Y	***1286.7719***	643.8896	1269.7453	635.3763	1268.7613	634.8843	12
10	**1177.5987**	589.3030	1159.5881	580.2977	V	***1123.7085***	562.3579	1106.6820	553.8446	1105.6980	553.3526	11
11	**1290.6828**	645.8450	1272.6722	636.8397	L	***1024.6401***	512.8237	1007.6136	504.3104	1006.6295	503.8184	10
12	**1377.7148**	689.3610	1359.7042	680.3558	S	***911.5560***	456.2817	894.5295	447.7684	893.5455	447.2764	9
13	**1490.7989**	745.9031	1472.7883	736.8978	I	***824.5240***	412.7656	807.4975	404.2524	806.5135	403.7604	8
14	**1603.8829**	802.4451	1585.8724	793.4398	I	***711.4400***	356.2236	694.4134	347.7103	693.4294	347.2183	7
15	**1700.9357**	850.9715	1682.9251	841.9662	P	***598.3559***	299.6816	581.3293	291.1683	580.3453	290.6763	6
16	1787.9677	894.4875	1769.9571	885.4822	S	***501.3031***	251.1552	484.2766	242.6419	483.2926	242.1499	5
17	1844.9892	922.9982	1826.9786	913.9929	G	***414.2711***	207.6392	397.2445	199.1259			4
18	1942.0419	971.5246	1924.0314	962.5193	P	***357.2496***	179.1285	340.2231	170.6152			3

**19**	2055.1260	1028.0666	2037.1154	1019.0614	**L**	260.1969	130.6021	243.1703	122.0888			**2**
**20**					**K**	147.1128	74.0600	130.0863	65.5468			**1**

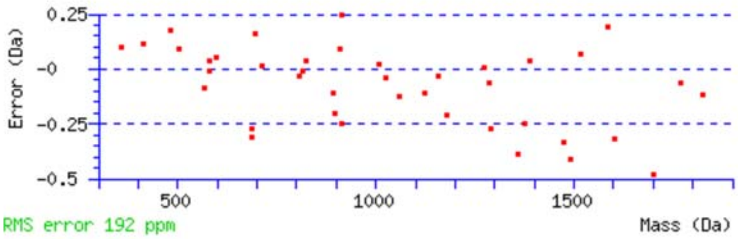

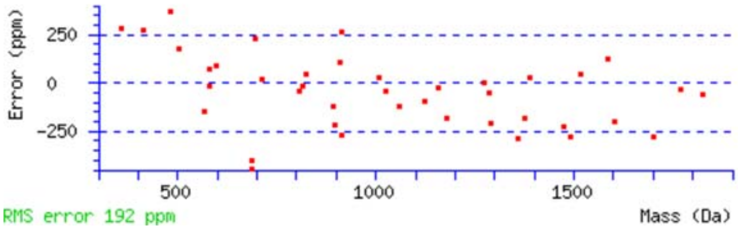

NCBI **BLAST** search of SLLTEVETYVLSIIPSGPLK
(Parameters: blastp, nr protein database, expect=20000, no filter, PAM30)
Other BLAST web gateways

All matches to this query

Score	Mr(calc):	Delta	Sequence
77.5	2200.2242	0.0032	SLLTEVETYVLSIIPSGPLK
12.0	2199.1899	1.0375	AAAGAVRLLVVQPQPEEQPPK
9.9	2199.2099	1.0175	GMAAPGPPQLPWLRLGPRLR
5.5	2199.1816	1.0458	YTRPTPVQKHAIPIIKEK
5.5	2199.1816	1.0458	YTRPTPVQKHAIPIIKEK
5.4	2198.2535	1.9739	IIGEKYQALNSRLLIGRPR
2.5	2198.1976	2.0298	YTRPTPVQKHAIPIIKEK
2.5	2198.1976	2.0298	YTRPTPVQKHAIPIIKEK
2.5	2198.2059	2.0215	AAAGAVRLLVVQPQPEEQPPK
2.5	2198.2059	2.0215	AAAGAVRLLVVQPQPEEQPPK

Mascot: http://www.matrixscience.com/

Mascot Search Results

Peptide View

MS/MS Fragmentation of **SLLTEVETYVLSIIPSGPLKAEIAQR**
Found in **M1**, NIB74xp

Match to Query 4146: 2868.584250 from(957.202026,3+) intensity(18955.3000)
Title: B111206_005.06613.06613.3
Data file B111206_005.mgf.mascot

Click mouse within plot area to zoom in by factor of two about that point
Or, Plot from 400 to 1700 Da Full range
Label all possible matches ○ Label matches used for scoring ◉

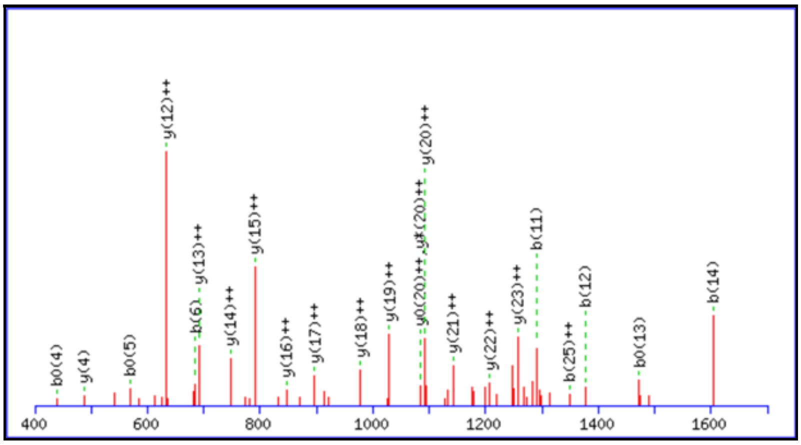

Monoisotopic mass of neutral peptide Mr(calc): 2868.5848
Fixed modifications: Carbamidomethyl (C) (apply to specified residues or termini only)
Variable modifications:
N-term : Acetyl (Protein N-term)
Ions Score: 96 **Expect:** 1.5e-08
Matches : 23/254 fragment ions using 24 most intense peaks (help)

#	b	b++	b*	b*++	b0	b0++	Seq.	y	y++	y*	y*++	y0	y0++	#
1	130.0499	65.5286			112.0393	56.5233	S							26
2	243.1339	122.0706			225.1234	113.0653	L	2740.5495	1370.7784	2723.5230	1362.2651	2722.5389	1361.7731	25
3	356.2180	178.6126			338.2074	169.6074	L	2627.4654	1314.2364	2610.4389	1305.7231	2609.4549	1305.2311	24
4	457.2657	229.1365			439.2551	220.1312	T	2514.3814	1257.6943	2497.3548	1249.1811	2496.3708	1248.6890	23
5	586.3083	293.6578			568.2977	284.6525	E	2413.3337	1207.1705	2396.3072	1198.6572	2395.3231	1198.1652	22
6	**685.3767**	343.1920			667.3661	334.1867	V	2284.2911	1142.6492	2267.2646	1134.1359	2266.2805	1133.6439	21
7	814.4193	407.7133			796.4087	398.7080	E	2185.2227	1093.1150	2168.1961	1084.6017	2167.2121	1084.1097	20
8	915.4670	458.2371			897.4564	449.2318	T	2056.1801	1028.5937	2039.1536	1020.0804	2038.1695	1019.5884	19
9	1078.5303	539.7688			1060.5197	530.7635	Y	1955.1324	978.0699	1938.1059	969.5566	1937.1219	969.0646	18
10	1177.5987	589.3030			1159.5881	580.2977	V	1792.0691	896.5382	1775.0425	888.0249	1774.0585	887.5329	17
11	**1290.6828**	645.8450			1272.6722	636.8397	L	1693.0007	847.0040	1675.9741	838.4907	1674.9901	837.9987	16
12	**1377.7148**	689.3610			1359.7042	680.3558	S	1579.9166	790.4619	1562.8901	781.9487	1561.9061	781.4567	15
13	1490.7989	745.9031			1472.7883	736.8978	I	1492.8846	746.9459	1475.8580	738.4327	1474.8740	737.9407	14
14	**1603.8829**	802.4451			1585.8724	793.4398	I	1379.8005	690.4039	1362.7740	681.8906	1361.7900	681.3986	13
15	1700.9357	850.9715			1682.9251	841.9662	P	1266.7165	633.8619	1249.6899	625.3486	1248.7059	624.8566	12
16	1787.9677	894.4875			1769.9571	885.4822	S	1169.6637	585.3355	1152.6371	576.8222	1151.6531	576.3302	11
17	1844.9892	922.9982			1826.9786	913.9929	G	1082.6317	541.8195	1065.6051	533.3062	1064.6211	532.8142	10
18	1942.0419	971.5246			1924.0314	962.5193	P	1025.6102	513.3087	1008.5837	504.7955	1007.5996	504.3035	9
19	2055.1260	1028.0666			2037.1154	1019.0614	L	928.5574	464.7824	911.5309	456.2691	910.5469	455.7771	8
20	2183.2210	1092.1141	2166.1944	1083.6008	2165.2104	1083.1088	K	815.4734	408.2403	798.4468	399.7271	797.4628	399.2350	7
21	2254.2581	1127.6327	2237.2315	1119.1194	2236.2475	1118.6274	A	687.3784	344.1928	670.3519	335.6796	669.3679	335.1876	6
22	2383.3007	1192.1540	2366.2741	1183.6407	2365.2901	1183.1487	E	616.3413	308.6743	599.3148	300.1610	598.3307	299.6690	5
23	2496.3847	1248.6960	2479.3582	1240.1827	2478.3742	1239.6907	I	487.2987	244.1530	470.2722	235.6397			4
24	2567.4219	1284.2146	2550.3953	1275.7013	2549.4113	1275.2093	A	374.2146	187.6110	357.1881	179.0977			3
25	2695.4804	1348.2439	2678.4539	1339.7306	2677.4699	1339.2386	Q	303.1775	152.0924	286.1510	143.5791			2
26							R	175.1190	88.0631	158.0924	79.5498			1

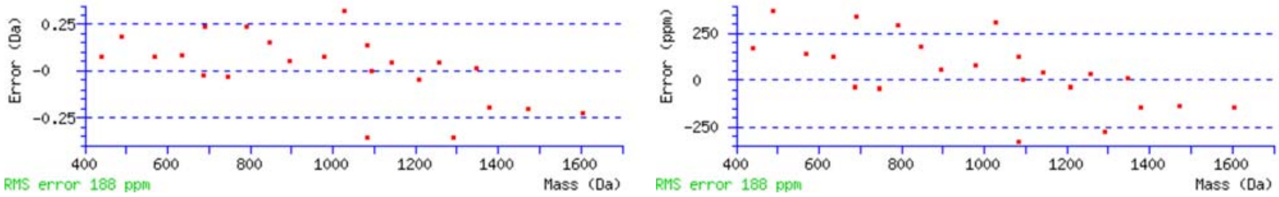

NCBI **BLAST** search of SLLTEVETYVLSIIPSGPLKAEIAQR
(Parameters: blastp, nr protein database, expect=20000, no filter, PAM30)
Other BLAST web gateways

All matches to this query

Score	Mr(calc):	Delta	Sequence
96.4	2868.5848	-0.0005	SLLTEVETYVLSIIPSGPLKAEIAQR
20.4	2868.5722	0.0121	WGPGSHLAAVRSAAELRLLAELLNAXR
5.5	2867.5882	0.9961	WGPGSHLAAVRSAAELRLLAELLNAXR
4.0	2868.6155	-0.0313	EIQQMAICMGIHTVLAVKXLFLVKK
3.7	2868.6185	-0.0342	KQAKENKPLIPQPQILGLTASPGVGGAR
3.6	2867.5426	1.0416	DRPTRGEALLDLVLTNMEEIVKEVK
2.3	2866.6006	1.9836	MVHTTSPLLLLLLLSLALVAPSLSAR
1.8	2866.5569	2.0274	LVAVAQSGLPLRVESLNTVKDLPLPR
1.8	2866.5569	2.0274	LVAVAQSGLPLRVESLNTVKDLPLPR
1.7	2867.6054	0.9789	VHATSPLLLLLLLSLALVAPGLSARK

Mascot: http://www.matrixscience.com/

Mascot Search Results

Peptide View

MS/MS Fragmentation of **SLLTEVETPIR**
Found in **M2**, NIB74xp

Match to Query 1108: 1256.696508 from(629.355530,2+) intensity(107025.0000)
Title: B111206_005.03205.03205.2
Data file B111206_005.mgf.mascot

Click mouse within plot area to zoom in by factor of two about that point
Or, Plot from 200 to 1150 Da Full range
Label all possible matches ○ Label matches used for scoring ◉

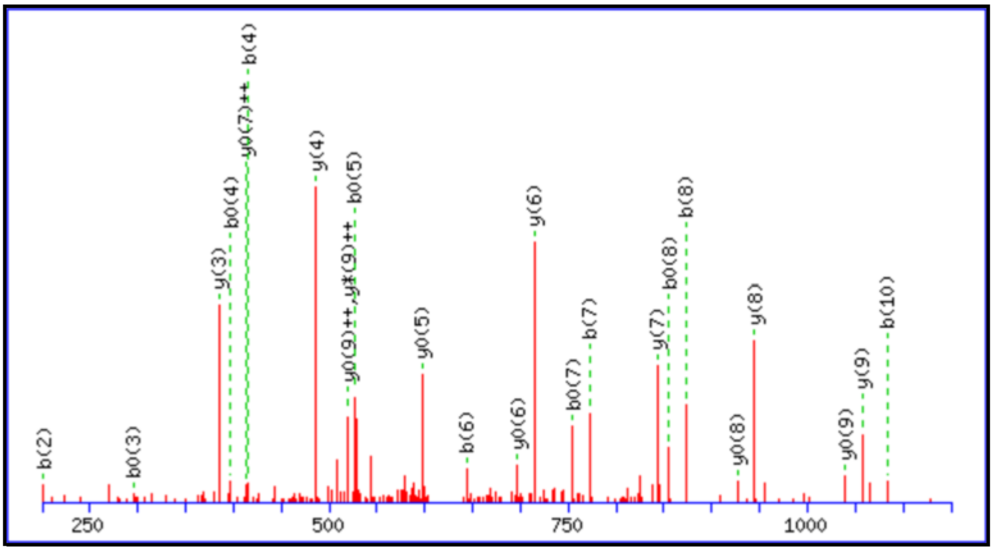

Monoisotopic mass of neutral peptide Mr(calc): 1256.6976
Fixed modifications: Carbamidomethyl (C) (apply to specified residues or termini only)
Ions Score: 57 **Expect:** 0.00011
Matches : 24/94 fragment ions using 28 most intense peaks (help)

#	b	b^{++}	b^{0}	b^{0++}	Seq.	y	y^{++}	y*	y^{*++}	y^{0}	y^{0++}	#
1	88.0393	44.5233	70.0287	35.5180	S							11
2	***201.1234***	101.0653	183.1128	92.0600	L	1170.6729	585.8401	1153.6463	577.3268	1152.6623	576.8348	10
3	314.2074	157.6074	296.1969	148.6021	L	***1057.5888***	529.2980	1040.5623	520.7848	1039.5782	520.2928	9
4	***415.2551***	208.1312	397.2445	199.1259	T	***944.5047***	472.7560	927.4782	464.2427	926.4942	463.7507	8
5	544.2977	272.6525	526.2871	263.6472	E	***843.4571***	422.2322	826.4305	413.7189	825.4465	413.2269	7
6	***643.3661***	322.1867	625.3556	313.1814	V	***714.4145***	357.7109	697.3879	349.1976	696.4039	348.7056	6
7	***772.4087***	386.7080	754.3981	377.7027	E	615.3461	308.1767	598.3195	299.6634	597.3355	299.1714	5
8	***873.4564***	437.2318	855.4458	428.2266	T	***486.3035***	243.6554	469.2769	235.1421	468.2929	234.6501	4
9	970.5092	485.7582	952.4986	476.7529	P	***385.2558***	193.1315	368.2292	184.6183			3
10	***1083.5932***	542.3002	1065.5827	533.2950	I	288.2030	144.6051	271.1765	136.0919			2
11					R	175.1190	88.0631	158.0924	79.5498			1

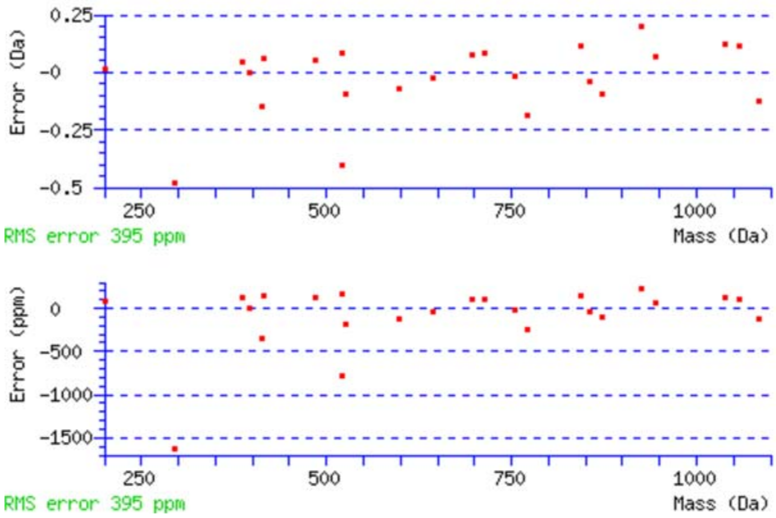

NCBI **BLAST** search of SLLTEVETPIR
(Parameters: blastp, nr protein database, expect=20000, no filter, PAM30)
Other BLAST web gateways

All matches to this query

Score	Mr(calc):	Delta	Sequence
57.0	1256.6976	-0.0011	SLLTEVETPIR
14.0	1256.6837	0.0128	KGQTAVAGQAAQK
7.2	1256.6724	0.0241	AIEAVKNEVER
5.1	1256.6837	0.0128	KGGNQVEIKQR
5.1	1256.6837	0.0128	KGGNQVEIKQR
4.5	1256.6949	0.0016	QQRAQTLRVR
4.0	1256.6949	0.0016	QERAQSLRLR
3.3	1256.7088	-0.0123	SLIVAGLGDGSIR
1.2	1256.6837	0.0129	REEEGIQLRK
1.2	1256.6837	0.0129	REEEGLQLRK

Mascot: http://www.matrixscience.com/

Mascot Search Results

Peptide View

MS/MS Fragmentation of **SLLTEVETPIR**
Found in **M2**, NIB74xp

Match to Query 1058: 1298.707494 from(650.361023,2+) intensity(216550.5781)
Title: B120210_005.04750.04750.2
Data file B120210_005.mgf.mascot

Click mouse within plot area to zoom in by factor of two about that point
Or, Plot from 200 to 1150 Da Full range
Label all possible matches ○ Label matches used for scoring ◉

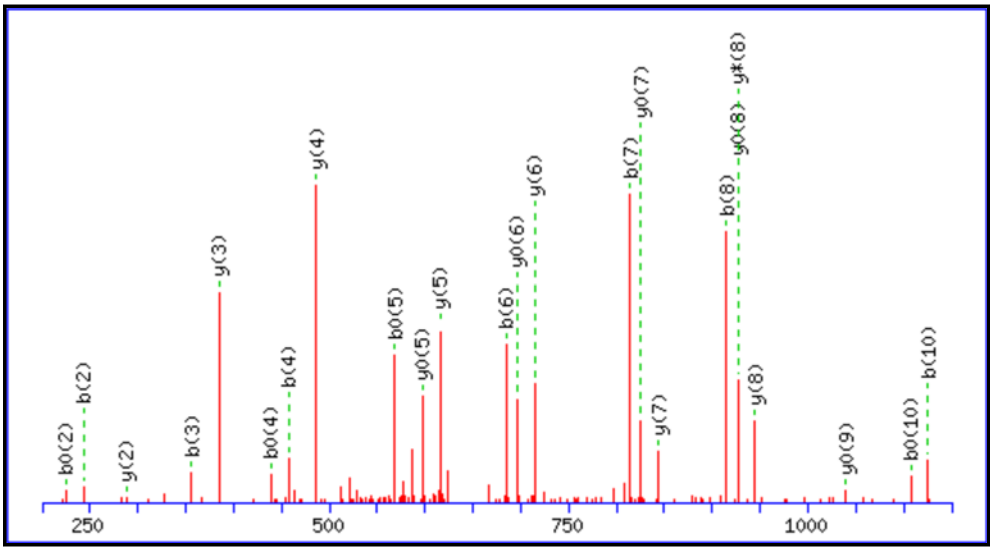

Monoisotopic mass of neutral peptide Mr(calc): 1298.7082
Fixed modifications: Carbamidomethyl (C) (apply to specified residues or termini only)
Variable modifications:
N-term : Acetyl (Protein N-term)
Ions Score: 67 **Expect:** 1.3e-05
Matches : 24/94 fragment ions using 30 most intense peaks (help)

#	b	b^{++}	b^{0}	b^{0++}	Seq.	y	y^{++}	y^{*}	y^{*++}	y^{0}	y^{0++}	#
**1**	130.0499	65.5286	112.0393	56.5233	**S**							**11**
**2**	***243.1339***	122.0706	225.1234	113.0653	**L**	1170.6729	585.8401	1153.6463	577.3268	1152.6623	576.8348	**10**
**3**	***356.2180***	178.6126	338.2074	169.6074	**L**	1057.5888	529.2980	1040.5623	520.7848	1039.5782	520.2928	**9**
**4**	***457.2657***	229.1365	439.2551	220.1312	**T**	***944.5047***	472.7560	927.4782	464.2427	926.4942	463.7507	**8**
**5**	586.3083	293.6578	568.2977	284.6525	**E**	***843.4571***	422.2322	826.4305	413.7189	825.4465	413.2269	**7**
**6**	***685.3767***	343.1920	667.3661	334.1867	**V**	***714.4145***	357.7109	697.3879	349.1976	696.4039	348.7056	**6**
**7**	***814.4193***	407.7133	796.4087	398.7080	**E**	***615.3461***	308.1767	598.3195	299.6634	597.3355	299.1714	**5**
**8**	***915.4670***	458.2371	897.4564	449.2318	**T**	***486.3035***	243.6554	469.2769	235.1421	468.2929	234.6501	**4**
**9**	1012.5197	506.7635	994.5092	497.7582	**P**	***385.2558***	193.1315	368.2292	184.6183			**3**
**10**	***1125.6038***	563.3055	1107.5932	554.3002	**I**	***288.2030***	144.6051	271.1765	136.0919			**2**
**11**					**R**	175.1190	88.0631	158.0924	79.5498			**1**

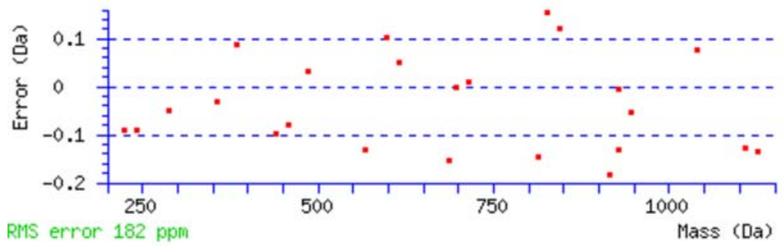

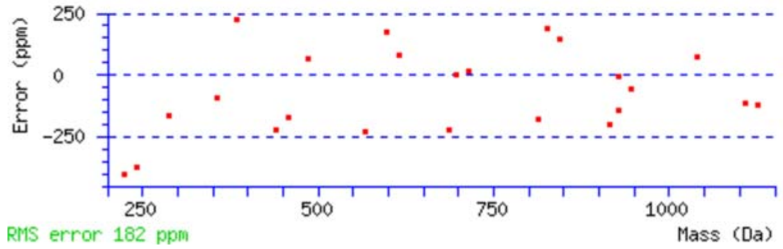

NCBI **BLAST** search of SLLTEVETPIR
(Parameters: blastp, nr protein database, expect=20000, no filter, PAM30)
Other BLAST web gateways

All matches to this query

Score	Mr(calc):	Delta	Sequence
67.4	1298.7082	-0.0007	SLLTEVETPIR
14.2	1297.6990	1.0085	LENKKPNLEGR
11.4	1297.7241	0.9834	DNAALKVTLPQK
10.7	1297.6990	1.0085	TGTLTSDHLVVR
9.8	1298.6904	0.0171	MLIQDIPSIPR
8.9	1298.6942	0.0133	IQENVRNAIGGK
8.7	1298.6830	0.0245	LENKKPNLEGR
8.2	1297.7159	0.9916	RTLSFQLILK
8.2	1297.7159	0.9916	RTLSFQLILK
7.5	1298.6999	0.0076	RTLSFQLILK

Mascot: http://www.matrixscience.com/

Mascot Search Results

Peptide View

MS/MS Fragmentation of **MDPNTVSSFQDILLR**
Found in **NEP**, NIB74xp

Match to Query 2176: 1776.870336 from(889.442444,2+) intensity(9877.0771)
Title: B120210_005.05387.05387.2
Data file B120210_005.mgf.mascot

Click mouse within plot area to zoom in by factor of two about that point
Or, Plot from 200 to 1700 Da Full range
Label all possible matches ○ Label matches used for scoring ◉

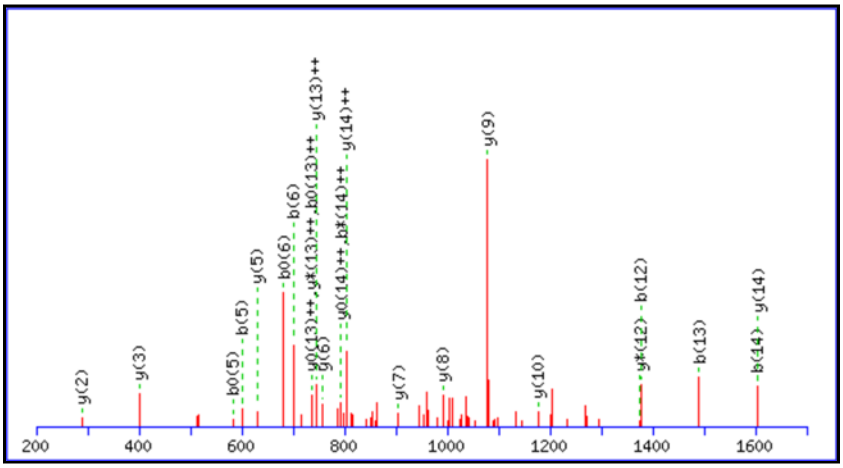

Monoisotopic mass of neutral peptide Mr(calc): 1776.8716
Fixed modifications: Carbamidomethyl (C) (apply to specified residues or termini only)
Variable modifications:
N-term : Acetyl (Protein N-term)
Ions Score: 51 **Expect:** 0.0015
Matches : 24/152 fragment ions using 39 most intense peaks (help)

#	b	b++	b*	b*++	b0	b0++	Seq.	y	y++	y*	y*++	y0	y0++	#
1	174.0583	87.5328					M							15
2	289.0853	145.0463			271.0747	136.0410	D	***1604.8279***	802.9176	1587.8013	794.4043	1586.8173	793.9123	14
3	386.1380	193.5727			368.1275	184.5674	P	1489.8009	745.4041	1472.7744	736.8908	1471.7904	736.3988	13
4	500.1810	250.5941	483.1544	242.0808	482.1704	241.5888	N	1392.7482	696.8777	1375.7216	688.3644	1374.7376	687.8724	12
5	**601.2286**	301.1180	584.2021	292.6047	583.2181	292.1127	T	1278.7052	639.8563	1261.6787	631.3430	1260.6947	630.8510	11
6	**700.2971**	350.6522	683.2705	342.1389	682.2865	341.6469	V	***1177.6575***	589.3324	1160.6310	580.8191	1159.6470	580.3271	10
7	787.3291	394.1682	770.3025	385.6549	769.3185	385.1629	S	***1078.5891***	539.7982	1061.5626	531.2849	1060.5786	530.7929	9
8	874.3611	437.6842	857.3346	429.1709	856.3505	428.6789	S	***991.5571***	496.2822	974.5306	487.7689	973.5465	487.2769	8
9	1021.4295	511.2184	1004.4030	502.7051	1003.4190	502.2131	F	***904.5251***	452.7662	887.4985	444.2529	886.5145	443.7609	7
10	1149.4881	575.2477	1132.4616	566.7344	1131.4775	566.2424	Q	***757.4567***	379.2320	740.4301	370.7187	739.4461	370.2267	6
11	1264.5150	632.7612	1247.4885	624.2479	1246.5045	623.7559	D	***629.3981***	315.2027	612.3715	306.6894	611.3875	306.1974	5
12	**1377.5991**	689.3032	1360.5726	680.7899	1359.5885	680.2979	I	514.3711	257.6892	497.3446	249.1759			4
13	**1490.6832**	745.8452	1473.6566	737.3319	1472.6726	736.8399	L	***401.2871***	201.1472	384.2605	192.6339			3
14	**1603.7672**	802.3873	1586.7407	793.8740	1585.7567	793.3820	L	***288.2030***	144.6051	271.1765	136.0919			2
15							R	175.1190	88.0631	158.0924	79.5498			1

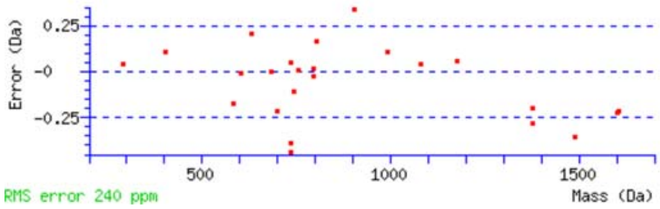

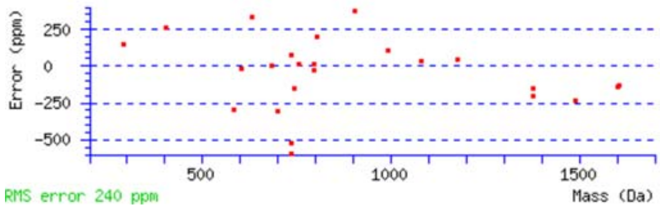

NCBI **BLAST** search of MDPNTVSSFQDILLR
(Parameters: blastp, nr protein database, expect=20000, no filter, PAM30)

Other BLAST web gateways

All matches to this query

Score	Mr(calc):	Delta	Sequence
50.7	1776.8716	-0.0013	MDPNTVSSFQDILLR
19.3	1776.8795	-0.0091	WNSNNVAAGYDIALLR
19.3	1776.8795	-0.0091	WNSNNVAAGYDIALLR
19.3	1776.8795	-0.0091	WNSNNVAAGYDIALLR
18.4	1776.8894	-0.0190	VGDQSLAYIAQGLDGLR
16.3	1776.8885	-0.0182	RMLELGLVKNLTYF
16.3	1776.8885	-0.0182	RMLELGLVKNLTYF
10.9	1776.8771	-0.0068	NDIGVTVHELSRTKK
10.0	1776.8771	-0.0068	NDIGVTVHELSRTKK
9.9	1776.8957	-0.0254	MARPQLQDVLSKLR

Mascot: http://www.matrixscience.com/

Mascot Search Results

Peptide View

MS/MS Fragmentation of **DPNTVSSFQDILLR**
Found in **NEP**, NIB74xp

Match to Query 2110: 1603.793310 from(802.903931,2+) intensity(78360.8000)
Title: B111206_005.03908.03908.2
Data file B111206_005.mgf.mascot

Click mouse within plot area to zoom in by factor of two about that point
Or, Plot from 200 to 1600 Da Full range
Label all possible matches ○ Label matches used for scoring ◉

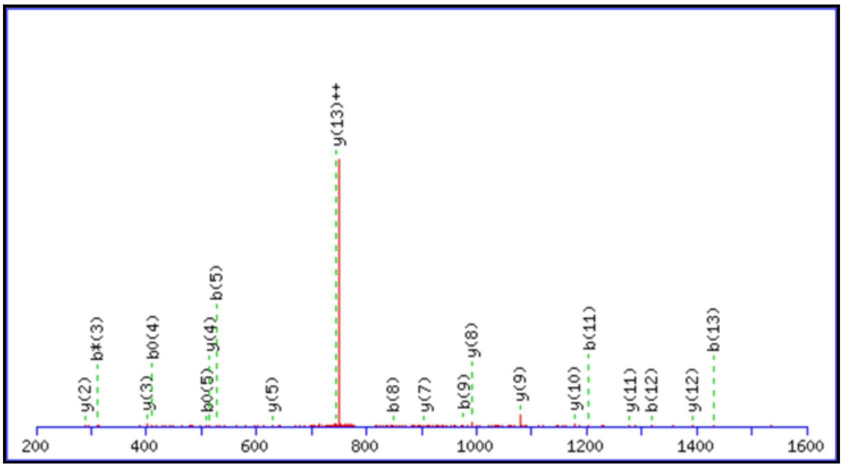

Monoisotopic mass of neutral peptide Mr(calc): 1603.8206
Fixed modifications: Carbamidomethyl (C) (apply to specified residues or termini only)
Ions Score: 43 **Expect:** 0.0071
Matches : 20/144 fragment ions using 47 most intense peaks (help)

#	b	b^{++}	b*	b^{*++}	b^0	b^{0++}	Seq.	y	y^{++}	y*	y^{*++}	y^0	y^{0++}	#
1	116.0342	58.5207			98.0237	49.5155	D							14
2	213.0870	107.0471			195.0764	98.0418	P	1489.8009	745.4041	1472.7744	736.8908	1471.7904	736.3988	13
3	327.1299	164.0686	310.1034	155.5553	309.1193	155.0633	N	**1392.7482**	696.8777	1375.7216	688.3644	1374.7376	687.8724	12
4	428.1776	214.5924	411.1510	206.0792	410.1670	205.5872	T	**1278.7052**	639.8563	1261.6787	631.3430	1260.6947	630.8510	11
5	**527.2460**	264.1266	510.2195	255.6134	509.2354	255.1214	V	**1177.6575**	589.3324	1160.6310	580.8191	1159.6470	580.3271	10
6	614.2780	307.6427	597.2515	299.1294	596.2675	298.6374	S	**1078.5891**	539.7982	1061.5626	531.2849	1060.5786	530.7929	9
7	701.3101	351.1587	684.2835	342.6454	683.2995	342.1534	S	**991.5571**	496.2822	974.5306	487.7689	973.5465	487.2769	8
8	**848.3785**	424.6929	831.3519	416.1796	830.3679	415.6876	F	**904.5251**	452.7662	887.4985	444.2529	886.5145	443.7609	7
9	**976.4371**	488.7222	959.4105	480.2089	958.4265	479.7169	Q	757.4567	379.2320	740.4301	370.7187	739.4461	370.2267	6
10	1091.4640	546.2356	1074.4374	537.7224	1073.4534	537.2304	D	**629.3981**	315.2027	612.3715	306.6894	611.3875	306.1974	5
11	**1204.5481**	602.7777	1187.5215	594.2644	1186.5375	593.7724	I	**514.3711**	257.6892	497.3446	249.1759			4
12	**1317.6321**	659.3197	1300.6056	650.8064	1299.6216	650.3144	L	**401.2871**	201.1472	384.2605	192.6339			3
13	**1430.7162**	715.8617	1413.6896	707.3485	1412.7056	706.8564	L	**288.2030**	144.6051	271.1765	136.0919			2
14							R	175.1190	88.0631	158.0924	79.5498			1

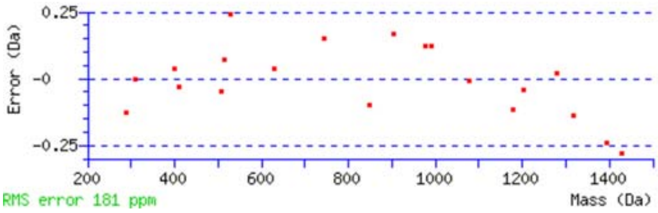

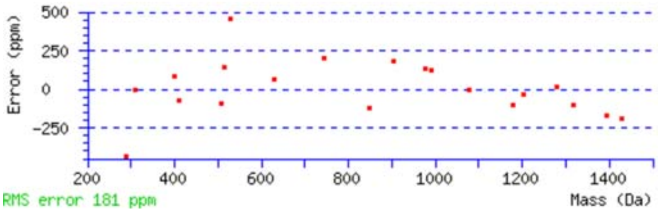

NCBI **BLAST** search of DPNTVSSFQDILLR
(Parameters: blastp, nr protein database, expect=20000, no filter, PAM30)
Other BLAST web gateways

All matches to this query

Score	Mr(calc):	Delta	Sequence
43.3	1603.8206	-0.0273	DPNTVSSFQDILLR
19.2	1603.7831	0.0102	RALGASNTSHELLR
12.7	1603.8127	-0.0194	SMAEQIEADVILLR
11.6	1603.8206	-0.0273	SSLSVQPGFLADLNR
8.6	1603.7793	0.0140	KQLTRMLSDFLR
7.5	1603.7745	0.0188	MMQVMPGDVRRPR
7.5	1603.7745	0.0188	MMQVMPGDVRRPR
7.5	1603.7745	0.0188	MMQVMPGDVRRPR
7.5	1601.7619	2.0314	FTKSKTVYSILR
7.5	1601.7619	2.0314	FTKSKTVYSILR

Mascot: http://www.matrixscience.com/

NIB74xp (MDCK-grown)
N-terminal peptide spectra

Mascot Search Results

Peptide View

MS/MS Fragmentation of **MDVNPTLLFLK**
Found in **PB1**, NIB74xp user_supplied_sequence

Match to Query 1630: 1331.715916 from(666.865234,2+) intensity(174472.0000)
Title: B120210_001.06461.06461.2
Data file B120210_001.mgf.mascot

Click mouse within plot area to zoom in by factor of two about that point
Or, Plot from 250 to 1200 Da Full range
Label all possible matches Label matches used for scoring

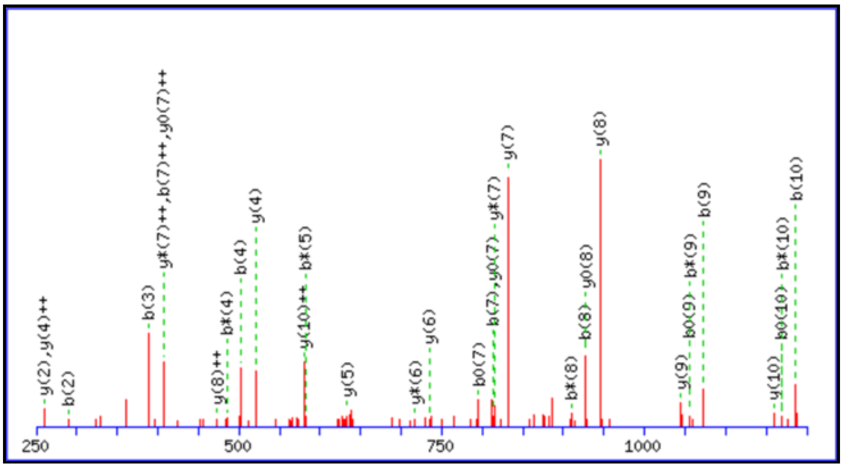

Monoisotopic mass of neutral peptide Mr(calc): 1331.7159
Fixed modifications: Carbamidomethyl (C) (apply to specified residues or termini only)
Variable modifications:
N-term : Acetyl (Protein N-term)
Ions Score: 57 **Expect:** 0.00033
Matches : 34/102 fragment ions using 53 most intense peaks (help)

#	b	b^{++}	b*	b^{*++}	b^{0}	b^{0++}	Seq.	y	y^{++}	y*	y^{*++}	y^{0}	y^{0++}	#
1	174.0583	87.5328					M							11
2	**289.0853**	145.0463			271.0747	136.0410	D	***1159.6721***	580.3397	1142.6456	571.8264	1141.6616	571.3344	10
3	**388.1537**	194.5805			370.1431	185.5752	V	***1044.6452***	522.8262	1027.6186	514.3130	1026.6346	513.8210	9
4	**502.1966**	251.6019	485.1701	243.0887	484.1860	242.5967	N	***945.5768***	473.2920	928.5502	464.7788	927.5662	464.2867	8
5	599.2494	300.1283	582.2228	291.6151	581.2388	291.1230	P	***831.5339***	416.2706	814.5073	407.7573	813.5233	407.2653	7
6	700.2971	350.6522	683.2705	342.1389	682.2865	341.6469	T	***734.4811***	367.7442	717.4545	359.2309	716.4705	358.7389	6
7	**813.3811**	407.1942	796.3546	398.6809	795.3706	398.1889	L	***633.4334***	317.2203	616.4069	308.7071			5
8	**926.4652**	463.7362	909.4386	455.2230	908.4546	454.7309	L	***520.3493***	260.6783	503.3228	252.1650			4
9	**1073.5336**	537.2704	1056.5070	528.7572	1055.5230	528.2652	F	***407.2653***	204.1363	390.2387	195.6230			3
10	**1186.6177**	593.8125	1169.5911	585.2992	1168.6071	584.8072	L	***260.1969***	130.6021	243.1703	122.0888			2
11							K	147.1128	74.0600	130.0863	65.5468			1

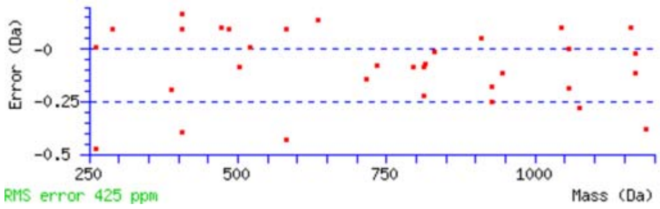

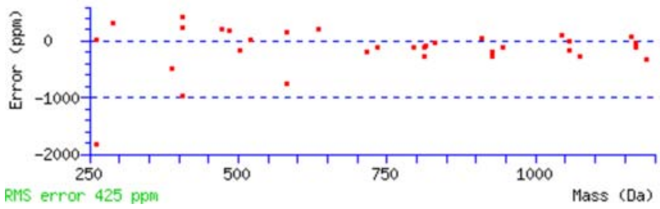

NCBI **BLAST** search of MDVNPTLLFLK
(Parameters: blastp, nr protein database, expect=20000, no filter, PAM30)
Other BLAST web gateways

All matches to this query

Score	Mr(calc):	Delta	Sequence

56.9	1331.7159	0.0000	MDVNPTLLFLK
19.2	1331.6973	0.0186	EFQPTLDLGAIK
16.4	1331.7231	-0.0072	IRIDVNSMQIK
14.6	1331.7118	0.0041	QILMENAELKK
14.5	1331.6924	0.0235	VTIMGIYSIKK
14.3	1331.7118	0.0041	MTVNEAKEKLK
12.8	1331.6924	0.0235	VTIMGIYSIKK
9.5	1331.7271	-0.0112	IWEKMQEKIK
9.1	1331.6932	0.0227	STGLQAEADAKIK
8.1	1331.6962	0.0197	RHKLESELIK

Mascot: http://www.matrixscience.com/

Mascot Search Results

Peptide View

MS/MS Fragmentation of **ASQGTKR**
Found in **NP**, NIB74xp user_supplied_sequence

Match to Query 132: 788.413732 from(395.214142,2+) intensity(8934.5273)
Title: B120210_001.00445.00445.2
Data file B120210_001.mgf.mascot

Click mouse within plot area to zoom in by factor of two about that point
Or, Plot from 100 to 700 Da Full range
Label all possible matches ○ Label matches used for scoring ◉

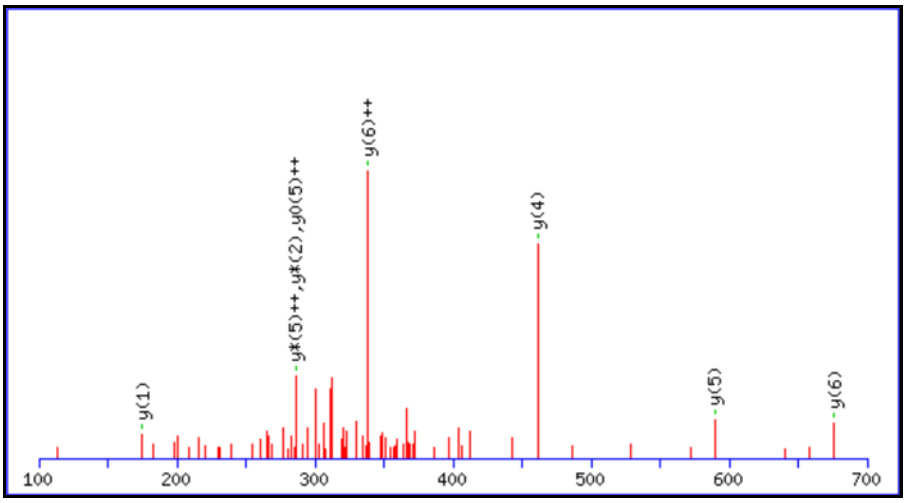

Monoisotopic mass of neutral peptide Mr(calc): 788.4140
Fixed modifications: Carbamidomethyl (C) (apply to specified residues or termini only)
Variable modifications:
N-term : Acetyl (Protein N-term)
Ions Score: 35 **Expect:** 0.039
Matches : 8/62 fragment ions using 6 most intense peaks (help)

#	b	b^{++}	b*	b^{*++}	b^0	b^{0++}	Seq.	y	y^{++}	y*	y^{*++}	y^0	y^{0++}	#
1	114.0550	57.5311					A							7
2	201.0870	101.0471			183.0764	92.0418	S	676.3737	338.6905	659.3471	330.1772	658.3631	329.6852	6
3	329.1456	165.0764	312.1190	156.5631	311.1350	156.0711	Q	589.3416	295.1745	572.3151	286.6612	571.3311	286.1692	5
4	386.1670	193.5872	369.1405	185.0739	368.1565	184.5819	G	461.2831	231.1452	444.2565	222.6319	443.2725	222.1399	4
5	487.2147	244.1110	470.1882	235.5977	469.2041	235.1057	T	404.2616	202.6344	387.2350	194.1212	386.2510	193.6292	3
6	615.3097	308.1585	598.2831	299.6452	597.2991	299.1532	K	303.2139	152.1106	286.1874	143.5973			2
7							R	175.1190	88.0631	158.0924	79.5498			1

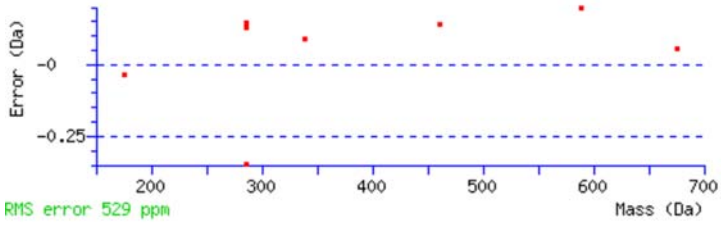

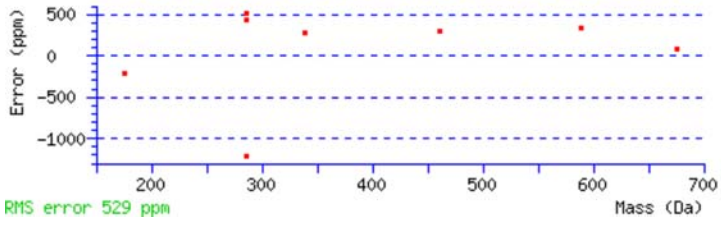

NCBI **BLAST** search of ASQGTKR
(Parameters: blastp, nr protein database, expect=20000, no filter, PAM30)
Other BLAST web gateways

All matches to this query

Score	Mr(calc):	Delta	Sequence
34.6	788.4140	-0.0003	ASQGTKR
18.3	788.4028	0.0109	ISDAIDR
18.3	788.4140	-0.0003	ISERQR
18.3	788.4181	-0.0043	ISWNLR
18.3	788.4028	0.0109	LSDADLR
18.3	788.4140	-0.0003	LSEQRR
18.3	788.4140	-0.0003	LSERER
18.3	788.4181	-0.0043	LSIDWR
18.3	788.4181	-0.0043	LSLNWR
18.3	788.4028	0.0109	LSNIADR

Mascot: http://www.matrixscience.com/

Mascot Search Results

Peptide View

MS/MS Fragmentation of **SLLTEVETYVLSIIPSGPLK**
Found in **M1**, NIB74xp user_supplied_sequence

Match to Query 3311: 2158.216160 from(1080.115356,2+) intensity(46216.4688)
Title: B120210_001.07238.07238.2
Data file B120210_001.mgf.mascot

Click mouse within plot area to zoom in by factor of two about that point
Or, Plot from 300 to 1800 Da Full range
Label all possible matches ○ Label matches used for scoring ◉

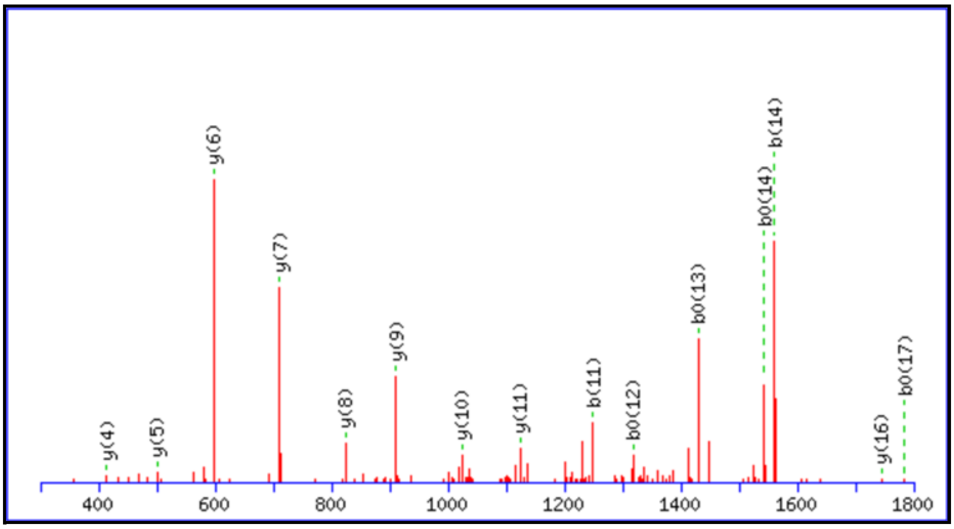

Monoisotopic mass of neutral peptide Mr(calc): 2158.2137
Fixed modifications: Carbamidomethyl (C) (apply to specified residues or termini only)
Ions Score: 89 **Expect:** 1.4e-08
Matches : 15/182 fragment ions using 15 most intense peaks (help)

#	b	b^{++}	b^0	b^{0++}	Seq.	y	y^{++}	y*	y^{*++}	y^0	y^{0++}	#
1	88.0393	44.5233	70.0287	35.5180	S							20
2	201.1234	101.0653	183.1128	92.0600	L	2072.1889	1036.5981	2055.1624	1028.0848	2054.1784	1027.5928	19
3	314.2074	157.6074	296.1969	148.6021	L	1959.1049	980.0561	1942.0783	971.5428	1941.0943	971.0508	18
4	415.2551	208.1312	397.2445	199.1259	T	1846.0208	923.5140	1828.9943	915.0008	1828.0102	914.5088	17
5	544.2977	272.6525	526.2871	263.6472	E	***1744.9731***	872.9902	1727.9466	864.4769	1726.9626	863.9849	16
6	643.3661	322.1867	625.3556	313.1814	V	1615.9305	808.4689	1598.9040	799.9556	1597.9200	799.4636	15
7	772.4087	386.7080	754.3981	377.7027	E	1516.8621	758.9347	1499.8356	750.4214	1498.8516	749.9294	14
8	873.4564	437.2318	855.4458	428.2266	T	1387.8195	694.4134	1370.7930	685.9001	1369.8090	685.4081	13
9	1036.5197	518.7635	1018.5092	509.7582	Y	1286.7719	643.8896	1269.7453	635.3763	1268.7613	634.8843	12
10	1135.5881	568.2977	1117.5776	559.2924	V	***1123.7085***	562.3579	1106.6820	553.8446	1105.6980	553.3526	11
11	**1248.6722**	624.8397	1230.6616	615.8345	L	***1024.6401***	512.8237	1007.6136	504.3104	1006.6295	503.8184	10
12	1335.7042	668.3558	1317.6937	659.3505	S	***911.5560***	456.2817	894.5295	447.7684	893.5455	447.2764	9
13	1448.7883	724.8978	1430.7777	715.8925	I	***824.5240***	412.7656	807.4975	404.2524	806.5135	403.7604	8
14	**1561.8724**	781.4398	1543.8618	772.4345	I	***711.4400***	356.2236	694.4134	347.7103	693.4294	347.2183	7
15	1658.9251	829.9662	1640.9146	820.9609	P	***598.3559***	299.6816	581.3293	291.1683	580.3453	290.6763	6
16	1745.9571	873.4822	1727.9466	864.4769	S	***501.3031***	251.1552	484.2766	242.6419	483.2926	242.1499	5
17	1802.9786	901.9929	1784.9680	892.9877	G	***414.2711***	207.6392	397.2445	199.1259			4
18	1900.0314	950.5193	1882.0208	941.5140	P	357.2496	179.1285	340.2231	170.6152			3
19	2013.1154	1007.0614	1995.1049	998.0561	L	260.1969	130.6021	243.1703	122.0888			2

**20**					**K**	147.1128	74.0600	130.0863	65.5468			**1**

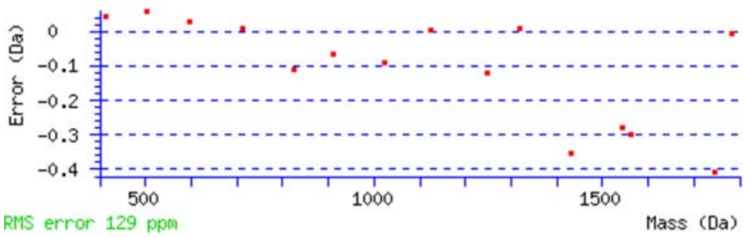

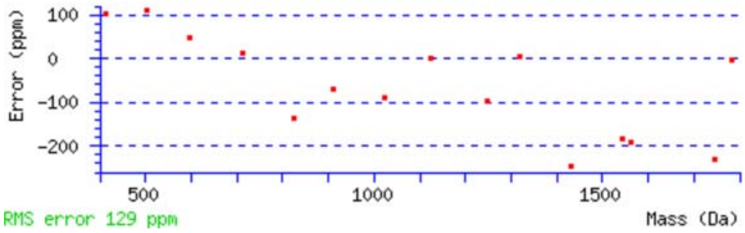

NCBI **BLAST** search of SLLTEVETYVLSIIPSGPLK
(Parameters: blastp, nr protein database, expect=20000, no filter, PAM30)
Other BLAST web gateways

All matches to this query

Score	Mr(calc):	Delta	Sequence
89.4	2158.2137	0.0025	SLLTEVETYVLSIIPSGPLK
5.3	2158.1746	0.0416	DQQILAQLLPLLHGNVNGSK
2.5	2156.1720	2.0442	TLKFFQMNPLILLLQKK
2.4	2157.1840	1.0322	LTTPRVVTKPGHIIKPIK
2.4	2157.1979	1.0183	KALSEFGSKIILLKPIIK
2.3	2158.2238	-0.0077	EQALKLIQILKGQSLLQR
2.0	2157.2045	1.0117	TPPLATIQPKPASLSQLPVR
2.0	2157.2483	0.9679	EMLLKISEITTPGKLFPLK
1.5	2157.1714	1.0447	NAKPVSILRDLITEAMEIK
1.2	2158.1875	0.0287	LKLKRPEQLQEVLDITR

Mascot: http://www.matrixscience.com/

Mascot Search Results

Peptide View

MS/MS Fragmentation of **SLLTEVETYVLSIIPSGPLK**
Found in **M1**, NIB74xp user_supplied_sequence

Match to Query 3370: 2200.225926 from(1101.120239,2+) intensity(423829.9375)
Title: B120210_001.07245.07245.2
Data file B120210_001.mgf.mascot

Click mouse within plot area to zoom in by factor of two about that point
Or, Plot from 300 to 2000 Da Full range
Label all possible matches Label matches used for scoring

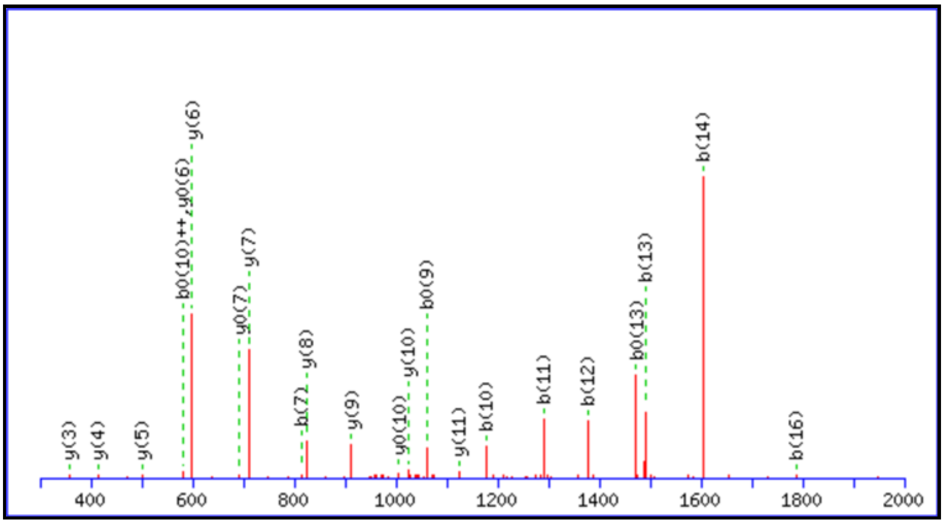

Monoisotopic mass of neutral peptide Mr(calc): 2200.2242
Fixed modifications: Carbamidomethyl (C) (apply to specified residues or termini only)
Variable modifications:
N-term : Acetyl (Protein N-term)
Ions Score: 64 **Expect:** 3e-06
Matches : 22/182 fragment ions using 29 most intense peaks (help)

#	b	b^{++}	b^0	b^{0++}	Seq.	y	y^{++}	y*	y^{*++}	y^0	y^{0++}	#
1	130.0499	65.5286	112.0393	56.5233	S							20
2	243.1339	122.0706	225.1234	113.0653	L	2072.1889	1036.5981	2055.1624	1028.0848	2054.1784	1027.5928	19
3	356.2180	178.6126	338.2074	169.6074	L	1959.1049	980.0561	1942.0783	971.5428	1941.0943	971.0508	18
4	457.2657	229.1365	439.2551	220.1312	T	1846.0208	923.5140	1828.9943	915.0008	1828.0102	914.5088	17
5	586.3083	293.6578	568.2977	284.6525	E	1744.9731	872.9902	1727.9466	864.4769	1726.9626	863.9849	16
6	685.3767	343.1920	667.3661	334.1867	V	1615.9305	808.4689	1598.9040	799.9556	1597.9200	799.4636	15
7	***814.4193***	407.7133	796.4087	398.7080	E	1516.8621	758.9347	1499.8356	750.4214	1498.8516	749.9294	14
8	915.4670	458.2371	897.4564	449.2318	T	1387.8195	694.4134	1370.7930	685.9001	1369.8090	685.4081	13
9	1078.5303	539.7688	1060.5197	530.7635	Y	1286.7719	643.8896	1269.7453	635.3763	1268.7613	634.8843	12
10	***1177.5987***	589.3030	1159.5881	580.2977	V	***1123.7085***	562.3579	1106.6820	553.8446	1105.6980	553.3526	11
11	***1290.6828***	645.8450	1272.6722	636.8397	L	***1024.6401***	512.8237	1007.6136	504.3104	1006.6295	503.8184	10
12	***1377.7148***	689.3610	1359.7042	680.3558	S	***911.5560***	456.2817	894.5295	447.7684	893.5455	447.2764	9
13	***1490.7989***	745.9031	1472.7883	736.8978	I	***824.5240***	412.7656	807.4975	404.2524	806.5135	403.7604	8
14	***1603.8829***	802.4451	1585.8724	793.4398	I	***711.4400***	356.2236	694.4134	347.7103	693.4294	347.2183	7
15	1700.9357	850.9715	1682.9251	841.9662	P	***598.3559***	299.6816	581.3293	291.1683	580.3453	290.6763	6
16	***1787.9677***	894.4875	1769.9571	885.4822	S	***501.3031***	251.1552	484.2766	242.6419	483.2926	242.1499	5
17	1844.9892	922.9982	1826.9786	913.9929	G	***414.2711***	207.6392	397.2445	199.1259			4
18	1942.0419	971.5246	1924.0314	962.5193	P	***357.2496***	179.1285	340.2231	170.6152			3

**19**	2055.1260	1028.0666	2037.1154	1019.0614	**L**	260.1969	130.6021	243.1703	122.0888			**2**
**20**					**K**	147.1128	74.0600	130.0863	65.5468			**1**

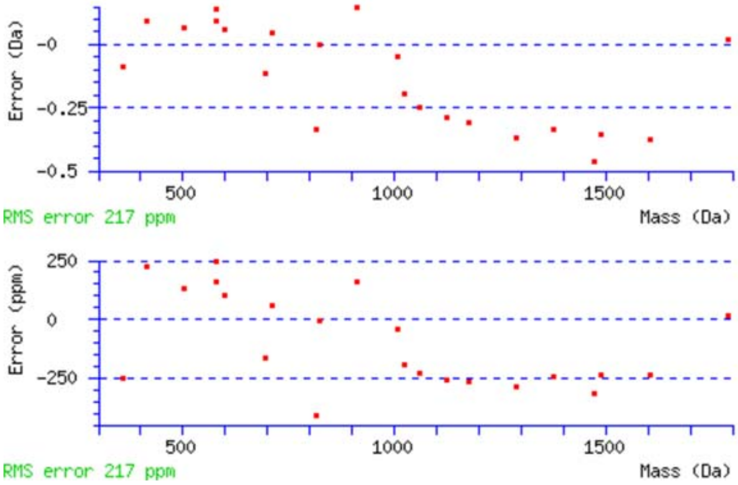

NCBI **BLAST** search of SLLTEVETYVLSIIPSGPLK
(Parameters: blastp, nr protein database, expect=20000, no filter, PAM30)
Other BLAST web gateways

All matches to this query

Score	Mr(calc):	Delta	Sequence
64.4	2200.2242	0.0017	SLLTEVETYVLSIIPSGPLK
5.2	2200.2150	0.0109	RSQPQLPQMSVPLVHQVKK
3.9	2200.2078	0.0181	VSQLLVLASLFYFCSGLKR
3.6	2198.2357	1.9902	KLLQMQPAQHGEIIKHLAK
3.3	2200.1949	0.0311	FLLKLPVDFSNIPTYLLK
2.9	2200.2020	0.0239	GFSLLGPGRPQAPLNLLIKK
2.7	2200.2458	-0.0199	VLSLKAILFVALHLLPGMK
2.5	2200.1949	0.0311	FLLKLPVDFSNIPTYLLK
2.1	2199.2011	1.0248	LQLLQEDYNRTPAQRLLK
2.0	2200.1964	0.0296	RITRYDAQLILENNSGIPK

Mascot: http://www.matrixscience.com/

MATRIX SCIENCE Mascot Search Results

Peptide View

MS/MS Fragmentation of **SLLTEVETYVLSIIPSGPLKAEIAQR**
Found in **M1**, NIB74xp user_supplied_sequence

Match to Query 4405: 2868.586034 from(1435.300293,2+) intensity(693875.7500)
Title: B120210_001.07356.07356.2
Data file B120210_001.mgf.mascot

Click mouse within plot area to zoom in by factor of two about that point
Or, Plot from 400 to 2000 Da Full range
Label all possible matches ○ Label matches used for scoring ◉

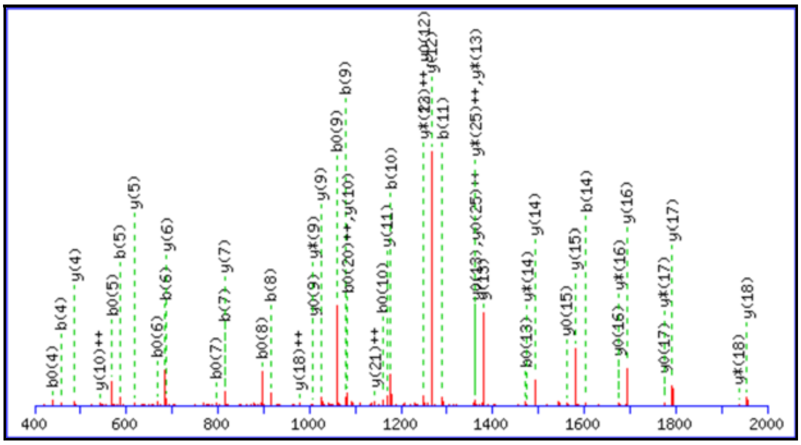


```
Monoisotopic mass of neutral peptide Mr(calc): 2868.5848
Fixed modifications: Carbamidomethyl (C) (apply to specified residues or termini only)
Variable modifications:
N-term : Acetyl (Protein N-term)
Ions Score: 86  Expect: 1.6e-08
Matches : 53/254 fragment ions using 74 most intense peaks   (help)
```

#	b	b^{++}	b^{*}	b^{*++}	b^{0}	b^{0++}	Seq.	y	y^{++}	y^{*}	y^{*++}	y^{0}	y^{0++}	#
1	130.0499	65.5286			112.0393	56.5233	S							26
2	243.1339	122.0706			225.1234	113.0653	L	2740.5495	1370.7784	2723.5230	1362.2651	2722.5389	1361.7731	25
3	356.2180	178.6126			338.2074	169.6074	L	2627.4654	1314.2364	2610.4389	1305.7231	2609.4549	1305.2311	24
4	***457.2657***	229.1365			439.2551	220.1312	T	2514.3814	1257.6943	2497.3548	1249.1811	2496.3708	1248.6890	23
5	***586.3083***	293.6578			568.2977	284.6525	E	2413.3337	1207.1705	2396.3072	1198.6572	2395.3231	1198.1652	22
6	***685.3767***	343.1920			667.3661	334.1867	V	2284.2911	1142.6492	2267.2646	1134.1359	2266.2805	1133.6439	21
7	***814.4193***	407.7133			796.4087	398.7080	E	2185.2227	1093.1150	2168.1961	1084.6017	2167.2121	1084.1097	20
8	***915.4670***	458.2371			897.4564	449.2318	T	2056.1801	1028.5937	2039.1536	1020.0804	2038.1695	1019.5884	19
9	***1078.5303***	539.7688			1060.5197	530.7635	Y	***1955.1324***	978.0699	1938.1059	969.5566	1937.1219	969.0646	18
10	***1177.5987***	589.3030			1159.5881	580.2977	V	***1792.0691***	896.5382	1775.0425	888.0249	1774.0585	887.5329	17
11	***1290.6828***	645.8450			1272.6722	636.8397	L	***1693.0007***	847.0040	1675.9741	838.4907	1674.9901	837.9987	16
12	1377.7148	689.3610			1359.7042	680.3558	S	***1579.9166***	790.4619	1562.8901	781.9487	1561.9061	781.4567	15
13	1490.7989	745.9031			1472.7883	736.8978	I	***1492.8846***	746.9459	1475.8580	738.4327	1474.8740	737.9407	14
14	***1603.8829***	802.4451			1585.8724	793.4398	I	***1379.8005***	690.4039	1362.7740	681.8906	1361.7900	681.3986	13
15	1700.9357	850.9715			1682.9251	841.9662	P	***1266.7165***	633.8619	1249.6899	625.3486	1248.7059	624.8566	12
16	1787.9677	894.4875			1769.9571	885.4822	S	***1169.6637***	585.3355	1152.6371	576.8222	1151.6531	576.3302	11
17	1844.9892	922.9982			1826.9786	913.9929	G	***1082.6317***	541.8195	1065.6051	533.3062	1064.6211	532.8142	10
18	1942.0419	971.5246			1924.0314	962.5193	P	***1025.6102***	513.3087	1008.5837	504.7955	1007.5996	504.3035	9
19	2055.1260	1028.0666			2037.1154	1019.0614	L	928.5574	464.7824	911.5309	456.2691	910.5469	455.7771	8
20	2183.2210	1092.1141	2166.1944	1083.6008	2165.2104	1083.1088	K	***815.4734***	408.2403	798.4468	399.7271	797.4628	399.2350	7
21	2254.2581	1127.6327	2237.2315	1119.1194	2236.2475	1118.6274	A	***687.3784***	344.1928	670.3519	335.6796	669.3679	335.1876	6
22	2383.3007	1192.1540	2366.2741	1183.6407	2365.2901	1183.1487	E	***616.3413***	308.6743	599.3148	300.1610	598.3307	299.6690	5
23	2496.3847	1248.6960	2479.3582	1240.1827	2478.3742	1239.6907	I	***487.2987***	244.1530	470.2722	235.6397			4
24	2567.4219	1284.2146	2550.3953	1275.7013	2549.4113	1275.2093	A	374.2146	187.6110	357.1881	179.0977			3
25	2695.4804	1348.2439	2678.4539	1339.7306	2677.4699	1339.2386	Q	303.1775	152.0924	286.1510	143.5791			2
26							R	175.1190	88.0631	158.0924	79.5498			1

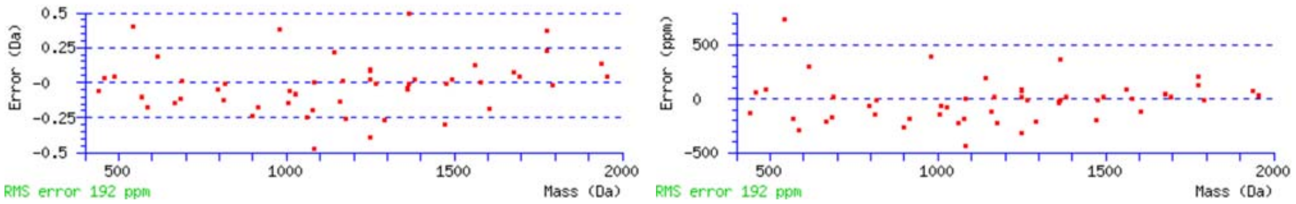

NCBI **BLAST** search of SLLTEVETYVLSIIPSGPLKAEIAQR
(Parameters: blastp, nr protein database, expect=20000, no filter, PAM30)
Other BLAST web gateways

All matches to this query

Score	Mr(calc):	Delta	Sequence
86.1	2868.5848	0.0013	SLLTEVETYVLSIIPSGPLKAEIAQR
2.7	2866.5262	2.0598	EGQNLKELYLVSCKITDYALIAIGR
1.4	2867.5738	1.0122	ANRPKLLIFVITGNPGFSAFYVPFAK

Mascot: http://www.matrixscience.com/

MATRIX SCIENCE Mascot Search Results

Peptide View

MS/MS Fragmentation of **SLLTEVETYVLSIIPSGPLKAEIAQRLEDVFAGK**
Found in **M1**, NIB74xp user_supplied_sequence

Match to Query 4892: 3728.032983 from(1243.684937,3+) intensity(492867.9688)
Title: B120210_001.07405.07405.3
Data file B120210_001.mgf.mascot

Click mouse within plot area to zoom in by factor of two about that point
Or, Plot from 400 to 2000 Da Full range
Label all possible matches ○ Label matches used for scoring ◉

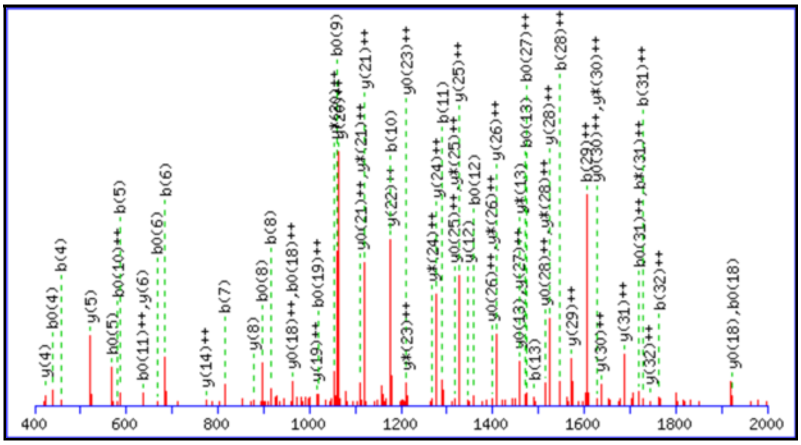

Monoisotopic mass of neutral peptide Mr(calc): 3728.0287
Fixed modifications: Carbamidomethyl (C) (apply to specified residues or termini only)
Variable modifications:
N-term : Acetyl (Protein N-term)
Ions Score: 49 **Expect:** 8.3e-05
Matches : 64/348 fragment ions using 118 most intense peaks (help)

#	b	b^{++}	b^{*}	b^{*++}	b^{0}	b^{0++}	Seq.	y	y^{++}	y^{*}	y^{*++}	y^{0}	y^{0++}	#
1	130.0499	65.5286			112.0393	56.5233	S							34
2	243.1339	122.0706			225.1234	113.0653	L	3599.9935	1800.5004	3582.9669	1791.9871	3581.9829	1791.4951	33
3	356.2180	178.6126			338.2074	169.6074	L	3486.9094	1743.9583	3469.8829	1735.4451	3468.8989	1734.9531	32
4	**457.2657**	229.1365			439.2551	220.1312	T	3373.8254	1687.4163	3356.7988	1678.9030	3355.8148	1678.4110	31
5	**586.3083**	293.6578			568.2977	284.6525	E	3272.7777	1636.8925	3255.7511	1628.3792	3254.7671	1627.8872	30
6	**685.3767**	343.1920			667.3661	334.1867	V	3143.7351	1572.3712	3126.7085	1563.8579	3125.7245	1563.3659	29
7	**814.4193**	407.7133			796.4087	398.7080	E	3044.6667	1522.8370	3027.6401	1514.3237	3026.6561	1513.8317	28
8	**915.4670**	458.2371			897.4564	449.2318	T	2915.6241	1458.3157	2898.5975	1449.8024	2897.6135	1449.3104	27
9	1078.5303	539.7688			1060.5197	530.7635	Y	2814.5764	1407.7918	2797.5498	1399.2786	2796.5658	1398.7866	26
10	**1177.5987**	589.3030			1159.5881	580.2977	V	2651.5131	1326.2602	2634.4865	1317.7469	2633.5025	1317.2549	25
11	**1290.6828**	645.8450			1272.6722	636.8397	L	2552.4447	1276.7260	2535.4181	1268.2127	2534.4341	1267.7207	24
12	1377.7148	689.3610			1359.7042	680.3558	S	2439.3606	1220.1839	2422.3340	1211.6707	2421.3500	1211.1786	23
13	**1490.7989**	745.9031			1472.7883	736.8978	I	2352.3286	1176.6679	2335.3020	1168.1546	2334.3180	1167.6626	22
14	1603.8829	802.4451			1585.8724	793.4398	I	2239.2445	1120.1259	2222.2179	1111.6126	2221.2339	1111.1206	21
15	1700.9357	850.9715			1682.9251	841.9662	P	2126.1604	1063.5839	2109.1339	1055.0706	2108.1499	1054.5786	20
16	1787.9677	894.4875			1769.9571	885.4822	S	2029.1077	1015.0575	2012.0811	1006.5442	2011.0971	1006.0522	19
17	1844.9892	922.9982			1826.9786	913.9929	G	1942.0756	971.5415	1925.0491	963.0282	1924.0651	962.5362	18
18	1942.0419	971.5246			1924.0314	962.5193	P	1885.0542	943.0307	1868.0276	934.5175	1867.0436	934.0254	17
19	2055.1260	1028.0666			2037.1154	1019.0614	L	1788.0014	894.5043	1770.9749	885.9911	1769.9908	885.4991	16
20	2183.2210	1092.1141	2166.1944	1083.6008	2165.2104	1083.1088	K	1674.9173	837.9623	1657.8908	829.4490	1656.9068	828.9570	15
21	2254.2581	1127.6327	2237.2315	1119.1194	2236.2475	1118.6274	A	1546.8224	773.9148	1529.7958	765.4016	1528.8118	764.9095	14
22	2383.3007	1192.1540	2366.2741	1183.6407	2365.2901	1183.1487	E	1475.7853	738.3963	1458.7587	729.8830	1457.7747	729.3910	13
23	2496.3847	1248.6960	2479.3582	1240.1827	2478.3742	1239.6907	I	***1346.7427***	673.8750	1329.7161	665.3617	1328.7321	664.8697	12
24	2567.4219	1284.2146	2550.3953	1275.7013	2549.4113	1275.2093	A	1233.6586	617.3329	1216.6321	608.8197	1215.6480	608.3277	11
25	2695.4804	1348.2439	2678.4539	1339.7306	2677.4699	1339.2386	Q	1162.6215	581.8144	1145.5950	573.3011	1144.6109	572.8091	10
26	2851.5815	1426.2944	2834.5550	1417.7811	2833.5710	1417.2891	R	1034.5629	517.7851	1017.5364	509.2718	1016.5524	508.7798	9
27	2964.6656	1482.8364	2947.6391	1474.3232	2946.6550	1473.8312	L	***878.4618***	439.7345	861.4353	431.2213	860.4512	430.7293	8

**28**	3093.7082	**1547.3577**	3076.6816	1538.8445	3075.6976	1538.3525	**E**	765.3777	383.1925	748.3512	374.6792	747.3672	374.1872	**7**
**29**	3208.7351	**1604.8712**	3191.7086	1596.3579	3190.7246	1595.8659	**D**	***636.3352***	318.6712	619.3086	310.1579	618.3246	309.6659	**6**
**30**	3307.8036	1654.4054	3290.7770	1645.8921	3289.7930	1645.4001	**V**	***521.3082***	261.1577	504.2817	252.6445			**5**
**31**	3454.8720	**1727.9396**	3437.8454	1719.4263	3436.8614	1718.9343	**F**	***422.2398***	211.6235	405.2132	203.1103			**4**
**32**	3525.9091	**1763.4582**	3508.8825	1754.9449	3507.8985	1754.4529	**A**	275.1714	138.0893	258.1448	129.5761			**3**
**33**	3582.9305	1791.9689	3565.9040	1783.4556	3564.9200	1782.9636	**G**	204.1343	102.5708	187.1077	94.0575			**2**
**34**							**K**	147.1128	74.0600	130.0863	65.5468			**1**

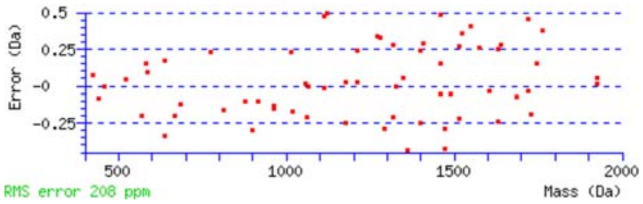

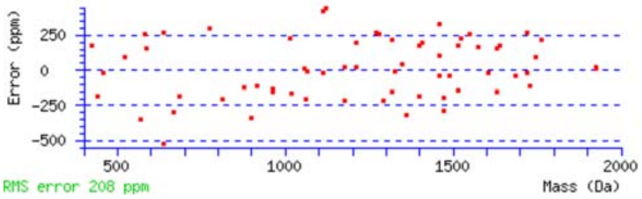

NCBI **BLAST** search of SLLTEVETYVLSIIPSGPLKAEIAQRLEDVFAGK
(Parameters: blastp, nr protein database, expect=20000, no filter, PAM30)
Other BLAST web gateways

All matches to this query

Score	Mr(calc):	Delta	Sequence
49.1	3728.0287	0.0042	SLLTEVETYVLSIIPSGPLKAEIAQRLEDVFAGK
5.2	3725.9644	2.0686	NLALFEEELDIRPKVSSLLGKLVSYTNLTQGAK
5.2	3725.9644	2.0686	NLALFEEELDIRPKVSSLLGKLVSYTNLTQGAK
4.9	3728.0215	0.0115	NSMTPNPGYQPSMNTSDMMGRMSYEPNK
4.1	3728.0215	0.0115	NSMTPNPGYQPSMNTSDMMGRMSYEPNK
1.2	3727.0375	0.9955	NSMTPNPGYQPSMNTSDMMGRMSYEPNK
1.2	3728.0215	0.0115	NSMTPNPGYQPSMNTSDMMGRMSYEPNK
0.9	3726.9824	1.0506	DTLIWSFVGPVGTVIIVNTVIFVLSAKVSCQRK
0.9	3726.9824	1.0506	DTLIWSFVGPVGTVIIVNTVIFVLSAKVSCQRK
0.9	3727.9619	0.0711	KPLSLMVMLSLAVGLSLVQDIIAAEYIILTMNR

Mascot: http://www.matrixscience.com/

Mascot Search Results

Peptide View

MS/MS Fragmentation of **SLLTEVETPIR**
Found in **M2**, NIB74xp user_supplied_sequence

Match to Query 1680: 1298.709202 from(650.361877,2+) intensity(4654521.0000)
Title: B120210_001_120211144847.05670.05670.2
Data file B120210_001_120211144847.mgf.mascot

Click mouse within plot area to zoom in by factor of two about that point
Or, Plot from 200 to 1300 Da Full range
Label all possible matches ○ Label matches used for scoring ◉

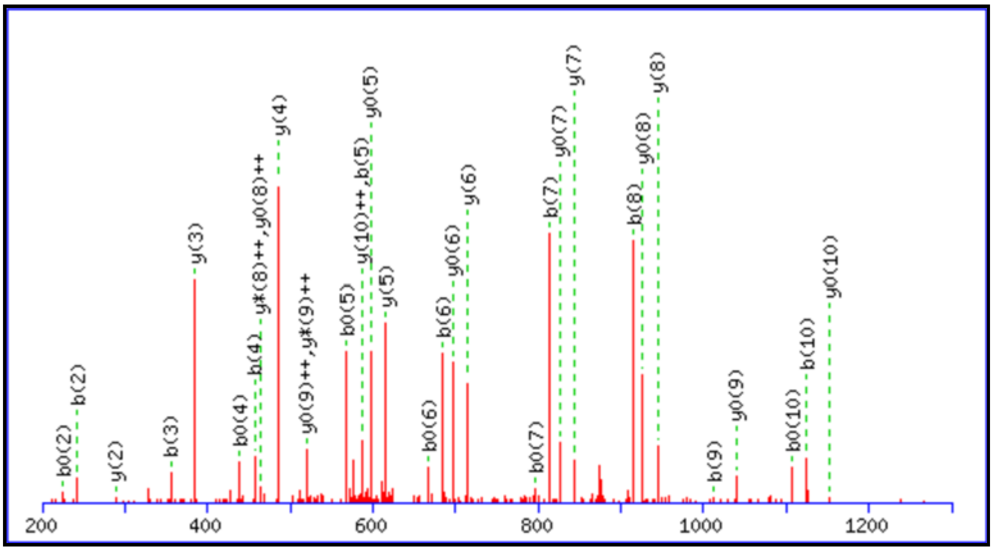

Monoisotopic mass of neutral peptide Mr(calc): 1298.7082
Fixed modifications: Carbamidomethyl (C) (apply to specified residues or termini only)
Variable modifications:
N-term : Acetyl (Protein N-term)
Ions Score: 75 **Expect:** 1.6e-06
Matches : 33/94 fragment ions using 41 most intense peaks (help)

#	b	b^{++}	b^{0}	b^{0++}	Seq.	y	y^{++}	y*	y^{*++}	y^{0}	y^{0++}	#
1	130.0499	65.5286	112.0393	56.5233	S							11
2	***243.1339***	122.0706	225.1234	113.0653	L	1170.6729	585.8401	1153.6463	577.3268	1152.6623	576.8348	10
3	***356.2180***	178.6126	338.2074	169.6074	L	1057.5888	529.2980	1040.5623	520.7848	1039.5782	520.2928	9
4	***457.2657***	229.1365	439.2551	220.1312	T	**944.5047**	472.7560	927.4782	464.2427	926.4942	463.7507	8
5	***586.3083***	293.6578	568.2977	284.6525	E	**843.4571**	422.2322	826.4305	413.7189	825.4465	413.2269	7
6	***685.3767***	343.1920	667.3661	334.1867	V	**714.4145**	357.7109	697.3879	349.1976	696.4039	348.7056	6
7	***814.4193***	407.7133	796.4087	398.7080	E	**615.3461**	308.1767	598.3195	299.6634	597.3355	299.1714	5
8	***915.4670***	458.2371	897.4564	449.2318	T	**486.3035**	243.6554	469.2769	235.1421	468.2929	234.6501	4
9	***1012.5197***	506.7635	994.5092	497.7582	P	**385.2558**	193.1315	368.2292	184.6183			3
10	***1125.6038***	563.3055	1107.5932	554.3002	I	**288.2030**	144.6051	271.1765	136.0919			2
11					R	175.1190	88.0631	158.0924	79.5498			1

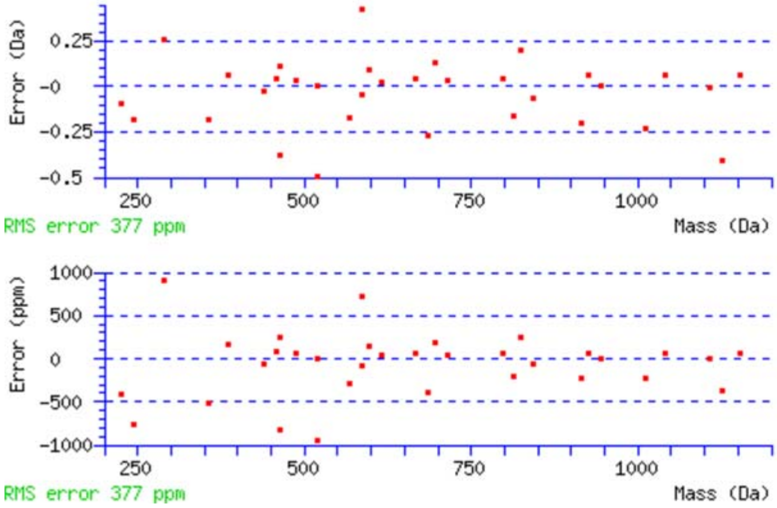

NCBI **BLAST** search of SLLTEVETPIR
(Parameters: blastp, nr protein database, expect=20000, no filter, PAM30)
Other BLAST web gateways

All matches to this query

Score	Mr(calc):	Delta	Sequence
75.3	1298.7082	0.0010	SLLTEVETPIR
12.6	1298.7167	-0.0075	RDLGSRLQAQR
12.5	1298.7241	-0.0149	RELQRMTLPR
10.1	1298.7112	-0.0019	FKTNIVLKTR
9.4	1298.7194	-0.0102	DLASVSLKNPVR
8.7	1297.6990	1.0102	DNPQSRNLLIK
8.6	1298.6886	0.0206	LEYQLLLAKK
7.8	1298.6943	0.0149	VQEEIDRVVGR
7.7	1298.6999	0.0093	FISKEILTIR
7.6	1298.6999	0.0093	TLIQFTVKLR

Mascot: http://www.matrixscience.com/

Mascot Search Results

Peptide View

MS/MS Fragmentation of **SLLTEVETPIRNEWGCR**
Found in **M2**, NIB74xp user_supplied_sequence

Match to Query 3383: 2101.025242 from(1051.519897,2+) intensity(2365194.2500)
Title: B120210_001_120211144847.05886.05886.2
Data file B120210_001_120211144847.mgf.mascot

Click mouse within plot area to zoom in by factor of two about that point
Or, Plot from 300 to 2000 Da Full range
Label all possible matches ○ Label matches used for scoring ◉

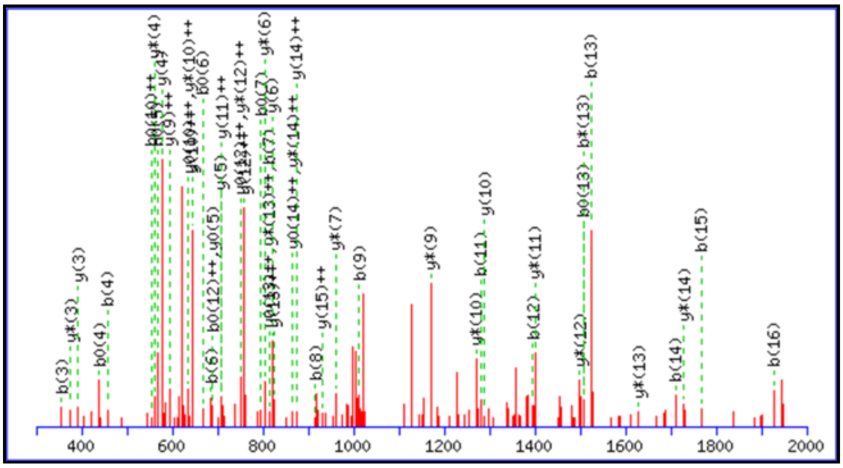

Monoisotopic mass of neutral peptide Mr(calc): 2101.0262
Fixed modifications: Carbamidomethyl (C) (apply to specified residues or termini only)
Variable modifications:
N-term : Acetyl (Protein N-term)
Ions Score: 37 **Expect:** 0.0015
Matches : 51/164 fragment ions using 140 most intense peaks (help)

#	b	b^{++}	b*	b^{*++}	b^0	b^{0++}	Seq.	y	y^{++}	y*	y^{*++}	y^0	y^{0++}	#
1	130.0499	65.5286			112.0393	56.5233	S							17
2	243.1339	122.0706			225.1234	113.0653	L	1972.9909	986.9991	1955.9644	978.4858	1954.9804	977.9938	16
3	***356.2180***	178.6126			338.2074	169.6074	L	1859.9069	930.4571	1842.8803	921.9438	1841.8963	921.4518	15
4	***457.2657***	229.1365			439.2551	220.1312	T	1746.8228	873.9150	1729.7962	865.4018	1728.8122	864.9098	14
5	586.3083	293.6578			568.2977	284.6525	E	1645.7751	823.3912	1628.7486	814.8779	1627.7646	814.3859	13
6	***685.3767***	343.1920			667.3661	334.1867	V	1516.7325	758.8699	1499.7060	750.3566	1498.7220	749.8646	12
7	***814.4193***	407.7133			796.4087	398.7080	E	1417.6641	709.3357	1400.6376	700.8224	1399.6535	700.3304	11
8	***915.4670***	458.2371			897.4564	449.2318	T	**1288.6215**	644.8144	1271.5950	636.3011	1270.6110	635.8091	10
9	***1012.5197***	506.7635			994.5092	497.7582	P	1187.5738	594.2906	1170.5473	585.7773	1169.5633	585.2853	9
10	1125.6038	563.3055			1107.5932	554.3002	I	1090.5211	545.7642	1073.4945	537.2509	1072.5105	536.7589	8
11	***1281.7049***	641.3561	1264.6783	632.8428	1263.6943	632.3508	R	977.4370	489.2221	960.4105	480.7089	959.4264	480.2169	7
12	***1395.7478***	698.3775	1378.7213	689.8643	1377.7373	689.3723	N	**821.3359**	411.1716	804.3093	402.6583	803.3253	402.1663	6
13	***1524.7904***	762.8988	1507.7639	754.3856	1506.7798	753.8936	E	**707.2930**	354.1501	690.2664	345.6368	689.2824	345.1448	5
14	***1710.8697***	855.9385	1693.8432	847.4252	1692.8592	846.9332	W	**578.2504**	289.6288	561.2238	281.1156			4
15	***1767.8912***	884.4492	1750.8646	875.9360	1749.8806	875.4440	G	**392.1711**	196.5892	375.1445	188.0759			3
16	***1927.9218***	964.4646	1910.8953	955.9513	1909.9113	955.4593	C	335.1496	168.0784	318.1231	159.5652			2
17							R	175.1190	88.0631	158.0924	79.5498			1

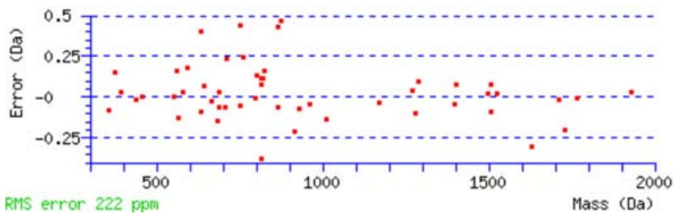

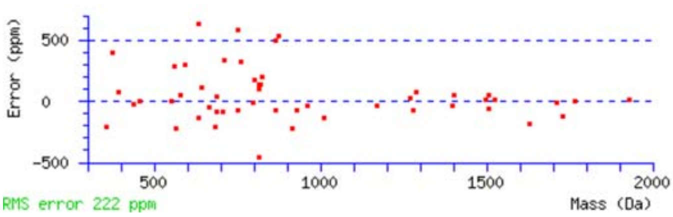

NCBI **BLAST** search of SLLTEVETPIRNEWGCR
(Parameters: blastp, nr protein database, expect=20000, no filter, PAM30)
Other BLAST web gateways

All matches to this query

Score	Mr(calc):	Delta	Sequence
36.6	2101.0262	-0.0010	SLLTEVETPIRNEWGCR
6.0	2101.0123	0.0130	VGLSVSPTVSGAKGPVPHPR
4.6	2101.0123	0.0130	VGLSVSPTVSGAKGPVPHPR
2.8	2098.9928	2.0325	KMQSITPVQTPQPLPFK
1.4	2101.0123	0.0130	VGLSVSPTVSGAKGPVPHPR
0.8	2098.9805	2.0447	YAFPVSNNLPLFAFEYK
0.4	2101.0228	0.0024	SSKPGWWLLNTEVGENQR

Mascot: http://www.matrixscience.com/

X181
N-terminal peptide spectra

Mascot Search Results

Peptide View

MS/MS Fragmentation of **MDVNPTLLFLK**
Found in **PB1**, NYMC X-181

Match to Query 1247: 1331.716894 from(666.865723,2+) intensity(540614.0000)
Title: B111206_007.05336.05336.2
Data file B111206_007.mgf.mascot

Click mouse within plot area to zoom in by factor of two about that point
Or, Plot from 200 to 1200 Da Full range
Label all possible matches ○ Label matches used for scoring ◉

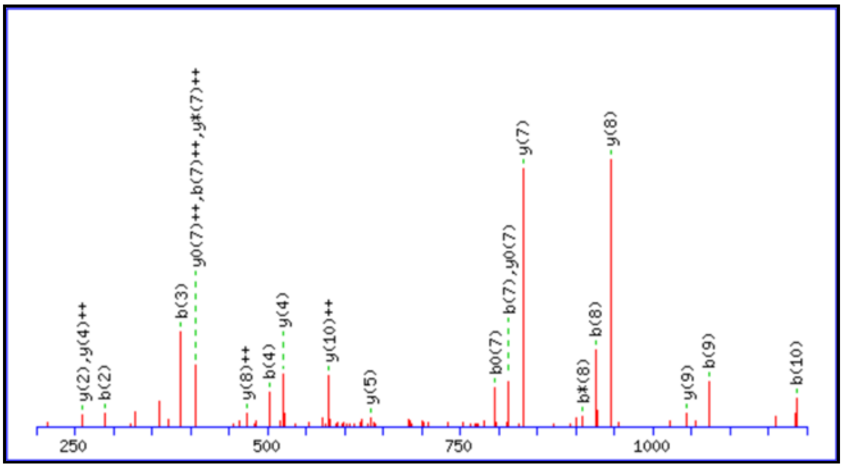

Monoisotopic mass of neutral peptide Mr(calc): 1331.7159
Fixed modifications: Carbamidomethyl (C) (apply to specified residues or termini only)
Variable modifications:
N-term : Acetyl (Protein N-term)
Ions Score: 74 **Expect:** 1.4e-05
Matches : 23/102 fragment ions using 20 most intense peaks (help)

#	b	b^{++}	b*	b^{*++}	b^0	b^{0++}	Seq.	y	y^{++}	y*	y^{*++}	y^0	y^{0++}	#
1	174.0583	87.5328					M							11
2	***289.0853***	145.0463			271.0747	136.0410	D	1159.6721	580.3397	1142.6456	571.8264	1141.6616	571.3344	10
3	***388.1537***	194.5805			370.1431	185.5752	V	***1044.6452***	522.8262	1027.6186	514.3130	1026.6346	513.8210	9
4	***502.1966***	251.6019	485.1701	243.0887	484.1860	242.5967	N	***945.5768***	473.2920	928.5502	464.7788	927.5662	464.2867	8
5	599.2494	300.1283	582.2228	291.6151	581.2388	291.1230	P	***831.5339***	416.2706	814.5073	407.7573	813.5233	407.2653	7
6	700.2971	350.6522	683.2705	342.1389	682.2865	341.6469	T	734.4811	367.7442	717.4545	359.2309	716.4705	358.7389	6
7	***813.3811***	407.1942	796.3546	398.6809	795.3706	398.1889	L	***633.4334***	317.2203	616.4069	308.7071			5
8	***926.4652***	463.7362	909.4386	455.2230	908.4546	454.7309	L	***520.3493***	260.6783	503.3228	252.1650			4
9	***1073.5336***	537.2704	1056.5070	528.7572	1055.5230	528.2652	F	***407.2653***	204.1363	390.2387	195.6230			3
10	***1186.6177***	593.8125	1169.5911	585.2992	1168.6071	584.8072	L	***260.1969***	130.6021	243.1703	122.0888			2
11							K	147.1128	74.0600	130.0863	65.5468			1

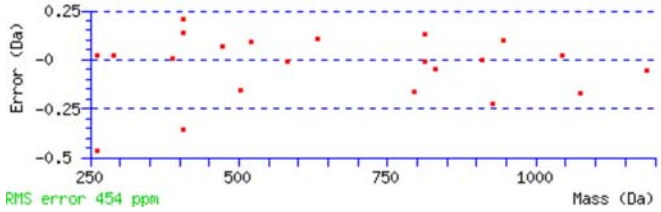

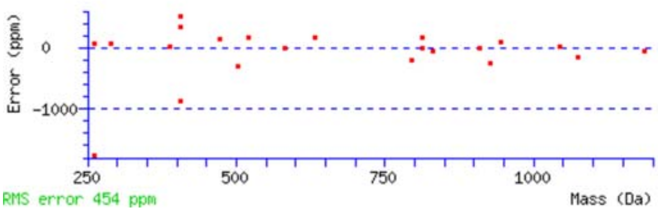

NCBI **BLAST** search of MDVNPTLLFLK
(Parameters: blastp, nr protein database, expect=20000, no filter, PAM30)
Other BLAST web gateways

All matches to this query

Score	Mr(calc):	Delta	Sequence

74.0	1331.7159	0.0010	MDVNPTLLFLK
26.6	1331.7231	-0.0062	KQMNSKLNELK
26.6	1331.6932	0.0237	KQTETLQNQLK
26.6	1331.7006	0.0163	MTPQKVSELLK
17.6	1331.6924	0.0245	VTIMGIYSIKK
17.6	1331.6924	0.0245	VTIMGIYSIKK
16.7	1331.6979	0.0190	EGSNLCQRKIK
15.7	1331.6979	0.0190	EEMGGGLRRALK
14.9	1330.6995	1.0174	CLINFYFLIK
14.3	1331.6932	0.0237	STAQAVALSDELK

Mascot: http://www.matrixscience.com/

{MATRIX SCIENCE} Mascot Search Results

Peptide View

MS/MS Fragmentation of **SLLTEVETYVLSIIPSGPLK**
Found in **M1**, NYMC X-181

Match to Query 2842: 2158.216650 from(1080.115601,2+) intensity(56373.7188)
Title: B120210_009.06659.06659.2
Data file B120210_009.mgf.mascot

Click mouse within plot area to zoom in by factor of two about that point
Or, Plot from 300 to 2000 Da Full range
Label all possible matches ○ Label matches used for scoring ◉

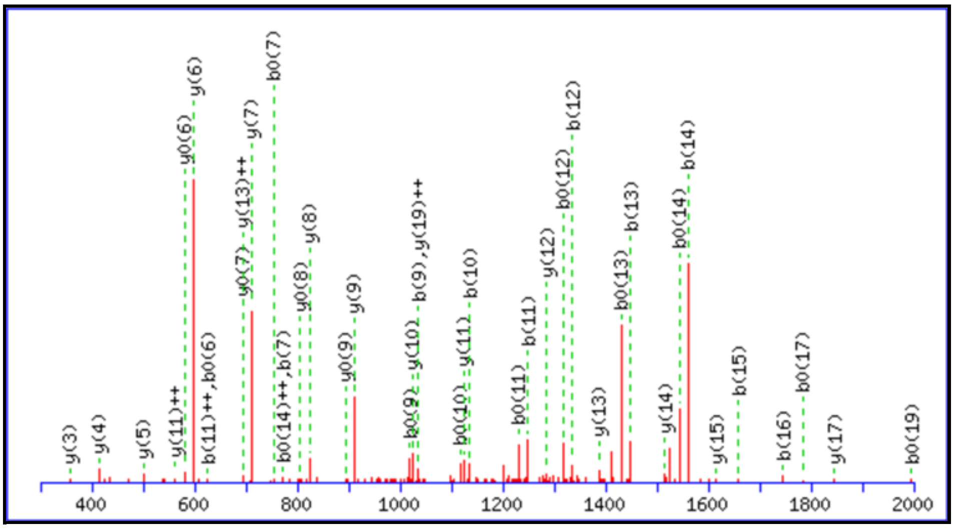

Monoisotopic mass of neutral peptide Mr(calc): 2158.2137
Fixed modifications: Carbamidomethyl (C) (apply to specified residues or termini only)
Ions Score: 90 **Expect:** 5.2e-08
Matches : 43/182 fragment ions using 68 most intense peaks (help)

#	b	b++	b0	b0++	Seq.	y	y++	y*	y*++	y0	y0++	#
1	88.0393	44.5233	70.0287	35.5180	S							20
2	201.1234	101.0653	183.1128	92.0600	L	2072.1889	1036.5981	2055.1624	1028.0848	2054.1784	1027.5928	19
3	314.2074	157.6074	296.1969	148.6021	L	1959.1049	980.0561	1942.0783	971.5428	1941.0943	971.0508	18
4	415.2551	208.1312	397.2445	199.1259	T	1846.0208	923.5140	1828.9943	915.0008	1828.0102	914.5088	17
5	544.2977	272.6525	526.2871	263.6472	E	1744.9731	872.9902	1727.9466	864.4769	1726.9626	863.9849	16
6	643.3661	322.1867	625.3556	313.1814	V	1615.9305	808.4689	1598.9040	799.9556	1597.9200	799.4636	15
7	772.4087	386.7080	754.3981	377.7027	E	1516.8621	758.9347	1499.8356	750.4214	1498.8516	749.9294	14
8	873.4564	437.2318	855.4458	428.2266	T	1387.8195	694.4134	1370.7930	685.9001	1369.8090	685.4081	13
9	1036.5197	518.7635	1018.5092	509.7582	Y	1286.7719	643.8896	1269.7453	635.3763	1268.7613	634.8843	12
10	1135.5881	568.2977	1117.5776	559.2924	V	1123.7085	562.3579	1106.6820	553.8446	1105.6980	553.3526	11
11	1248.6722	624.8397	1230.6616	615.8345	L	1024.6401	512.8237	1007.6136	504.3104	1006.6295	503.8184	10
12	1335.7042	668.3558	1317.6937	659.3505	S	911.5560	456.2817	894.5295	447.7684	893.5455	447.2764	9
13	1448.7883	724.8978	1430.7777	715.8925	I	824.5240	412.7656	807.4975	404.2524	806.5135	403.7604	8
14	1561.8724	781.4398	1543.8618	772.4345	I	711.4400	356.2236	694.4134	347.7103	693.4294	347.2183	7
15	1658.9251	829.9662	1640.9146	820.9609	P	598.3559	299.6816	581.3293	291.1683	580.3453	290.6763	6
16	1745.9571	873.4822	1727.9466	864.4769	S	501.3031	251.1552	484.2766	242.6419	483.2926	242.1499	5
17	1802.9786	901.9929	1784.9680	892.9877	G	414.2711	207.6392	397.2445	199.1259			4
18	1900.0314	950.5193	1882.0208	941.5140	P	357.2496	179.1285	340.2231	170.6152			3
19	2013.1154	1007.0614	1995.1049	998.0561	L	260.1969	130.6021	243.1703	122.0888			2

**20**					**K**	147.1128	74.0600	130.0863	65.5468			**1**

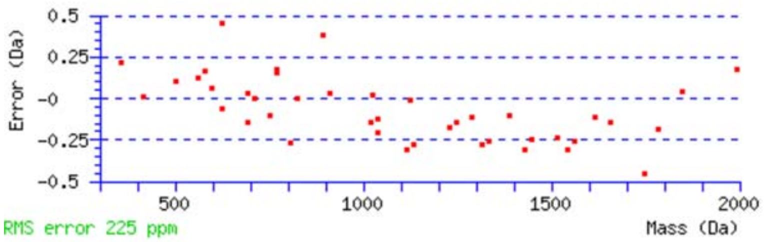

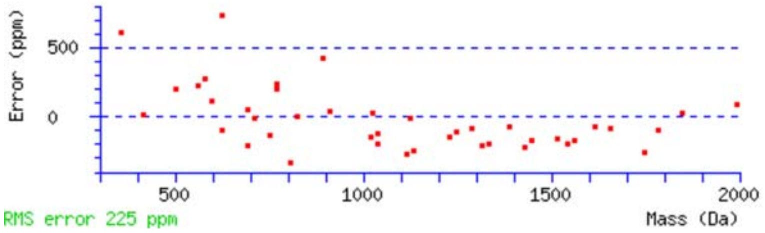

NCBI **BLAST** search of SLLTEVETYVLSIIPSGPLK
(Parameters: blastp, nr protein database, expect=20000, no filter, PAM30)
Other BLAST web gateways

All matches to this query

Score	Mr(calc):	Delta	Sequence
90.2	2158.2137	0.0030	SLLTEVETYVLSIIPSGPLK
15.5	2158.1971	0.0196	QGVVRGSRPPVSRALPQPEK
7.6	2157.2131	1.0036	QGVVRGSRPPVSRALPQPEK
6.5	2158.2126	0.0040	IAQGISGLVRPLLSGLKVSK
4.4	2158.2435	-0.0269	QVITIIILLLLCKASDFR
3.6	2157.1714	1.0452	NAKPVSILRDLITEAMEIK
2.7	2158.1858	0.0309	LQGNLAERSNFLARGASLLK
2.7	2158.1858	0.0309	LQGNLAERSNFLARGASLLK
2.5	2158.1972	0.0194	WRVRLAIIEYMPLLAGQL
2.4	2157.1727	1.0439	KKEVLIAGLKPSLAVYLR

Mascot: http://www.matrixscience.com/

MATRIX SCIENCE Mascot Search Results

Peptide View

MS/MS Fragmentation of **SLLTEVETYVLSIIPSGPLK**
Found in **M1**, NYMC X-181

Match to Query 3472: 2200.224462 from(1101.119507,2+) intensity(238763.0000)
Title: B111206_007.06750.06750.2
Data file B111206_007.mgf.mascot

Click mouse within plot area to zoom in by factor of two about that point
Or, Plot from 300 to 1900 Da Full range
Label all possible matches ○ Label matches used for scoring ◉

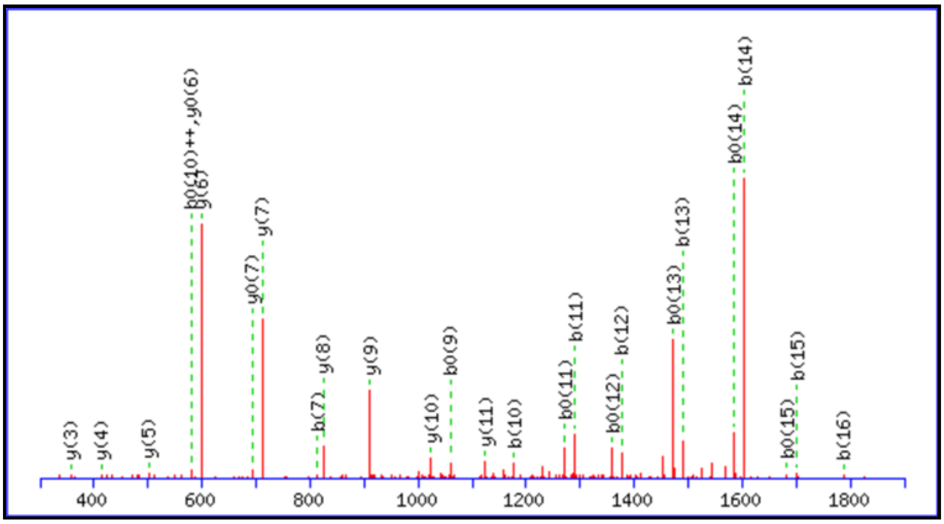

Monoisotopic mass of neutral peptide Mr(calc): 2200.2242
Fixed modifications: Carbamidomethyl (C) (apply to specified residues or termini only)
Variable modifications:
N-term : Acetyl (Protein N-term)
Ions Score: 79 **Expect:** 1.2e-06
Matches : 26/182 fragment ions using 30 most intense peaks (help)

#	b	b^{++}	b^0	b^{0++}	Seq.	y	y^{++}	y*	y^{*++}	y^0	y^{0++}	#
1	130.0499	65.5286	112.0393	56.5233	S							20
2	243.1339	122.0706	225.1234	113.0653	L	2072.1889	1036.5981	2055.1624	1028.0848	2054.1784	1027.5928	19
3	356.2180	178.6126	338.2074	169.6074	L	1959.1049	980.0561	1942.0783	971.5428	1941.0943	971.0508	18
4	457.2657	229.1365	439.2551	220.1312	T	1846.0208	923.5140	1828.9943	915.0008	1828.0102	914.5088	17
5	586.3083	293.6578	568.2977	284.6525	E	1744.9731	872.9902	1727.9466	864.4769	1726.9626	863.9849	16
6	685.3767	343.1920	667.3661	334.1867	V	1615.9305	808.4689	1598.9040	799.9556	1597.9200	799.4636	15
7	***814.4193***	407.7133	796.4087	398.7080	E	1516.8621	758.9347	1499.8356	750.4214	1498.8516	749.9294	14
8	915.4670	458.2371	897.4564	449.2318	T	1387.8195	694.4134	1370.7930	685.9001	1369.8090	685.4081	13
9	1078.5303	539.7688	1060.5197	530.7635	Y	1286.7719	643.8896	1269.7453	635.3763	1268.7613	634.8843	12
10	***1177.5987***	589.3030	1159.5881	580.2977	V	***1123.7085***	562.3579	1106.6820	553.8446	1105.6980	553.3526	11
11	***1290.6828***	645.8450	1272.6722	636.8397	L	***1024.6401***	512.8237	1007.6136	504.3104	1006.6295	503.8184	10
12	***1377.7148***	689.3610	1359.7042	680.3558	S	***911.5560***	456.2817	894.5295	447.7684	893.5455	447.2764	9
13	***1490.7989***	745.9031	1472.7883	736.8978	I	***824.5240***	412.7656	807.4975	404.2524	806.5135	403.7604	8
14	***1603.8829***	802.4451	1585.8724	793.4398	I	***711.4400***	356.2236	694.4134	347.7103	693.4294	347.2183	7
15	***1700.9357***	850.9715	1682.9251	841.9662	P	***598.3559***	299.6816	581.3293	291.1683	580.3453	290.6763	6
16	***1787.9677***	894.4875	1769.9571	885.4822	S	***501.3031***	251.1552	484.2766	242.6419	483.2926	242.1499	5
17	1844.9892	922.9982	1826.9786	913.9929	G	***414.2711***	207.6392	397.2445	199.1259			4
18	1942.0419	971.5246	1924.0314	962.5193	P	***357.2496***	179.1285	340.2231	170.6152			3

**19**	2055.1260	1028.0666	2037.1154	1019.0614	**L**	260.1969	130.6021	243.1703	122.0888			**2**
**20**					**K**	147.1128	74.0600	130.0863	65.5468			**1**

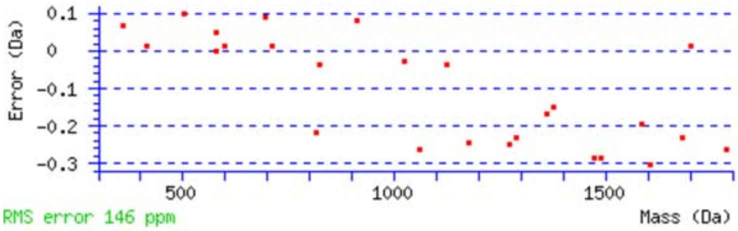

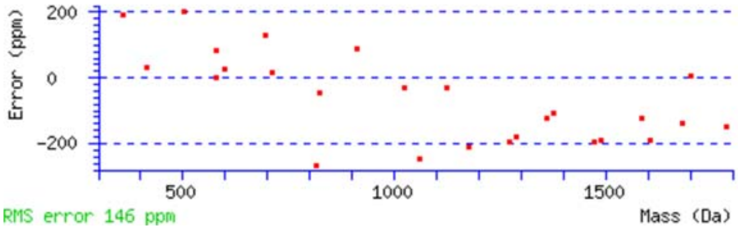

NCBI **BLAST** search of SLLTEVETYVLSIIPSGPLK
(Parameters: blastp, nr protein database, expect=20000, no filter, PAM30)
Other BLAST web gateways

All matches to this query

Score	Mr(calc):	Delta	Sequence
78.9	2200.2242	0.0002	SLLTEVETYVLSIIPSGPLK
14.8	2200.1841	0.0403	RLASTLLRVSSLLVHNDAR
12.3	2199.1899	1.0346	AAAGAVRLLVVQPQPEEQPPK
10.3	2199.1899	1.0346	AAAGAVRLLVVQPQPEEQPPK
10.3	2199.1899	1.0346	AAAGAVRLLVVQPQPEEQPPK
8.1	2199.2099	1.0146	GMAAPGPPQLPWLRLGPRLR
7.2	2198.1824	2.0421	NSILAPAIKTNVHEVKASVK
6.7	2200.2055	0.0190	TLLAVGCGAAGVGIVALPLGLAK
6.6	2198.1976	2.0268	YTRPTPVQKHAIPIIKEK
6.6	2198.1976	2.0268	YTRPTPVQKHAIPIIKEK

Mascot: http://www.matrixscience.com/

Mascot Search Results

Peptide View

MS/MS Fragmentation of **SLLTEVETYVLSIIPSGPLKAEIAQR**
Found in **M1**, NYMC X-181

Match to Query 4707: 2868.607872 from(957.209900,3+) intensity(598344.0000)
Title: B111206_007.07129.07129.3
Data file B111206_007.mgf.mascot

Click mouse within plot area to zoom in by factor of two about that point
Or, Plot from 200 to 1900 Da Full range
Label all possible matches ○ Label matches used for scoring ◉

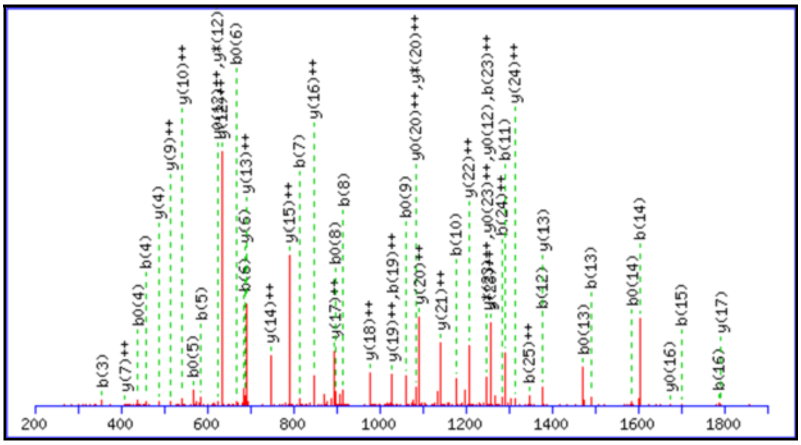

Monoisotopic mass of neutral peptide Mr(calc): 2868.5848
Fixed modifications: Carbamidomethyl (C) (apply to specified residues or termini only)
Variable modifications:
N-term : Acetyl (Protein N-term)
Ions Score: 122 **Expect:** 3.8e-11
Matches : 52/254 fragment ions using 62 most intense peaks (help)

#	b	b^{++}	b*	b^{*++}	b^{0}	b^{0++}	Seq.	y	y^{++}	y*	y^{*++}	y^{0}	y^{0++}	#
1	130.0499	65.5286			112.0393	56.5233	S							26
2	243.1339	122.0706			225.1234	113.0653	L	2740.5495	1370.7784	2723.5230	1362.2651	2722.5389	1361.7731	25
3	**356.2180**	178.6126			338.2074	169.6074	L	2627.4654	1314.2364	2610.4389	1305.7231	2609.4549	1305.2311	24
4	**457.2657**	229.1365			439.2551	220.1312	T	2514.3814	1257.6943	2497.3548	1249.1811	2496.3708	1248.6890	23
5	**586.3083**	293.6578			568.2977	284.6525	E	2413.3337	1207.1705	2396.3072	1198.6572	2395.3231	1198.1652	22
6	**685.3767**	343.1920			667.3661	334.1867	V	2284.2911	1142.6492	2267.2646	1134.1359	2266.2805	1133.6439	21
7	**814.4193**	407.7133			796.4087	398.7080	E	2185.2227	1093.1150	2168.1961	1084.6017	2167.2121	1084.1097	20
8	**915.4670**	458.2371			897.4564	449.2318	T	2056.1801	1028.5937	2039.1536	1020.0804	2038.1695	1019.5884	19
9	1078.5303	539.7688			1060.5197	530.7635	Y	1955.1324	978.0699	1938.1059	969.5566	1937.1219	969.0646	18
10	**1177.5987**	589.3030			1159.5881	580.2977	V	**1792.0691**	896.5382	1775.0425	888.0249	1774.0585	887.5329	17
11	**1290.6828**	645.8450			1272.6722	636.8397	L	1693.0007	847.0040	1675.9741	838.4907	1674.9901	837.9987	16
12	**1377.7148**	689.3610			1359.7042	680.3558	S	1579.9166	790.4619	1562.8901	781.9487	1561.9061	781.4567	15
13	**1490.7989**	745.9031			1472.7883	736.8978	I	1492.8846	746.9459	1475.8580	738.4327	1474.8740	737.9407	14
14	**1603.8829**	802.4451			1585.8724	793.4398	I	**1379.8005**	690.4039	1362.7740	681.8906	1361.7900	681.3986	13
15	**1700.9357**	850.9715			1682.9251	841.9662	P	1266.7165	633.8619	1249.6899	625.3486	1248.7059	624.8566	12
16	**1787.9677**	894.4875			1769.9571	885.4822	S	1169.6637	585.3355	1152.6371	576.8222	1151.6531	576.3302	11
17	1844.9892	922.9982			1826.9786	913.9929	G	1082.6317	541.8195	1065.6051	533.3062	1064.6211	532.8142	10
18	1942.0419	971.5246			1924.0314	962.5193	P	1025.6102	513.3087	1008.5837	504.7955	1007.5996	504.3035	9
19	2055.1260	**1028.0666**			2037.1154	1019.0614	L	928.5574	464.7824	911.5309	456.2691	910.5469	455.7771	8
20	2183.2210	1092.1141	2166.1944	1083.6008	2165.2104	1083.1088	K	815.4734	408.2403	798.4468	399.7271	797.4628	399.2350	7
21	2254.2581	1127.6327	2237.2315	1119.1194	2236.2475	1118.6274	A	**687.3784**	344.1928	670.3519	335.6796	669.3679	335.1876	6
22	2383.3007	1192.1540	2366.2741	1183.6407	2365.2901	1183.1487	E	616.3413	308.6743	599.3148	300.1610	598.3307	299.6690	5
23	2496.3847	**1248.6960**	2479.3582	1240.1827	2478.3742	1239.6907	I	**487.2987**	244.1530	470.2722	235.6397			4
24	2567.4219	**1284.2146**	2550.3953	1275.7013	2549.4113	1275.2093	A	374.2146	187.6110	357.1881	179.0977			3
25	2695.4804	**1348.2439**	2678.4539	1339.7306	2677.4699	1339.2386	Q	303.1775	152.0924	286.1510	143.5791			2
26							R	175.1190	88.0631	158.0924	79.5498			1

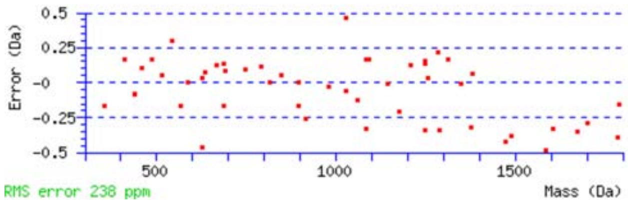

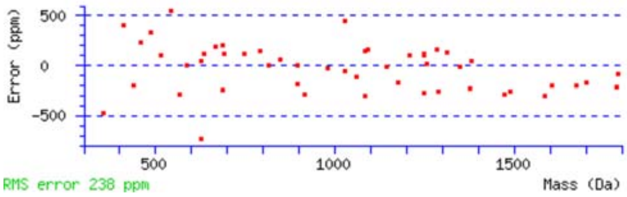

NCBI **BLAST** search of SLLTEVETYVLSIIPSGPLKAEIAQR
(Parameters: blastp, nr protein database, expect=20000, no filter, PAM30)
Other BLAST web gateways

All matches to this query

Score	Mr(calc):	Delta	Sequence
121.7	2868.5848	0.0231	SLLTEVETYVLSIIPSGPLKAEIAQR
10.0	2868.5722	0.0357	WGPGSHLAAVRSAAELRLLAELLNAXR
5.2	2866.6006	2.0072	MVHTTSPLLLLLLLSLALVAPSLSAR
3.2	2866.6006	2.0072	MVHTTSPLLLLLLLSLALVAPSLSAR
3.2	2866.6006	2.0072	MVHTTSPLLLLLLLSLALVAPSLSAR
2.6	2866.5569	2.0510	LVAVAQSGLPLRVESLNTVKDLPLPR
2.6	2866.5569	2.0510	LVAVAQSGLPLRVESLNTVKDLPLPR
2.0	2868.6340	-0.0261	LKFMTTWIIFVISAAVALILTNTFR
2.0	2867.5718	1.0361	IIEDLVDLIDKLTLALATFGNIMHAK
0.6	2868.6155	-0.0077	EIQQMAICMGIHTVLAVKXLFLVKK

Mascot: http://www.matrixscience.com/

Mascot Search Results

Peptide View

MS/MS Fragmentation of **SLLTEVETPIR**
Found in **M2**, NYMC X-181

Match to Query 1013: 1256.696874 from(629.355713,2+) intensity(555767.0000)
Title: B111206_007.03366.03366.2
Data file B111206_007.mgf.mascot

Click mouse within plot area to zoom in by factor of two about that point
Or, Plot from 150 to 1150 Da Full range
Label all possible matches ○ Label matches used for scoring ◉

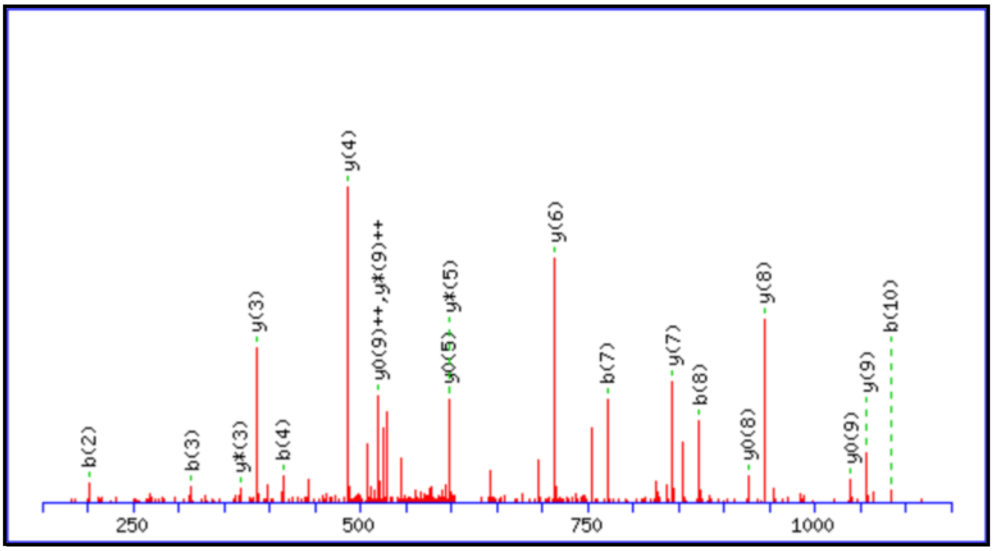

Monoisotopic mass of neutral peptide Mr(calc): 1256.6976
Fixed modifications: Carbamidomethyl (C) (apply to specified residues or termini only)
Ions Score: 60 **Expect:** 2.5e-05
Matches : 19/94 fragment ions using 20 most intense peaks (help)

#	b	b^{++}	b^{0}	b^{0++}	Seq.	y	y^{++}	y*	y^{*++}	y^{0}	y^{0++}	#
1	88.0393	44.5233	70.0287	35.5180	S							11
2	***201.1234***	101.0653	183.1128	92.0600	L	1170.6729	585.8401	1153.6463	577.3268	1152.6623	576.8348	10
3	***314.2074***	157.6074	296.1969	148.6021	L	***1057.5888***	529.2980	1040.5623	520.7848	1039.5782	520.2928	9
4	***415.2551***	208.1312	397.2445	199.1259	T	***944.5047***	472.7560	927.4782	464.2427	926.4942	463.7507	8
5	544.2977	272.6525	526.2871	263.6472	E	***843.4571***	422.2322	826.4305	413.7189	825.4465	413.2269	7
6	643.3661	322.1867	625.3556	313.1814	V	***714.4145***	357.7109	697.3879	349.1976	696.4039	348.7056	6
7	***772.4087***	386.7080	754.3981	377.7027	E	615.3461	308.1767	598.3195	299.6634	597.3355	299.1714	5
8	***873.4564***	437.2318	855.4458	428.2266	T	***486.3035***	243.6554	469.2769	235.1421	468.2929	234.6501	4
9	970.5092	485.7582	952.4986	476.7529	P	***385.2558***	193.1315	368.2292	184.6183			3
10	***1083.5932***	542.3002	1065.5827	533.2950	I	288.2030	144.6051	271.1765	136.0919			2
11					R	175.1190	88.0631	158.0924	79.5498			1

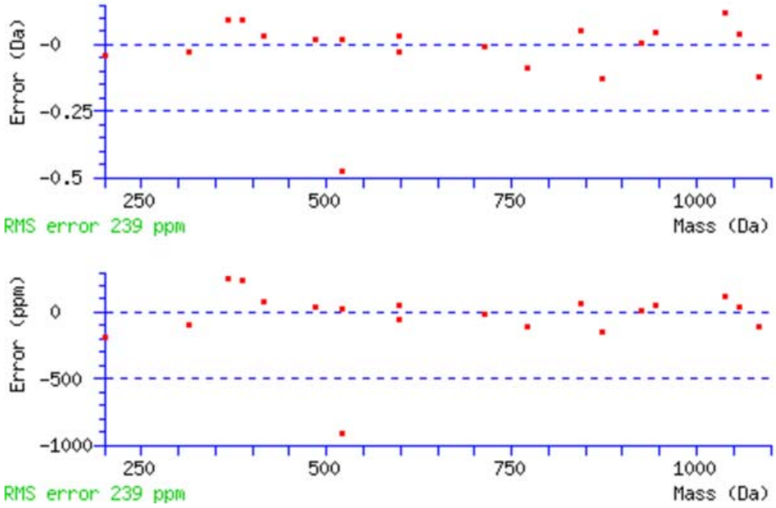

NCBI **BLAST** search of SLLTEVETPIR
(Parameters: blastp, nr protein database, expect=20000, no filter, PAM30)
Other BLAST web gateways

All matches to this query

Score	Mr(calc):	Delta	Sequence
60.2	1256.6976	-0.0007	SLLTEVETPIR
10.4	1256.7088	-0.0119	VTIRELQQLR
10.1	1256.6724	0.0245	AEREQLELLR
9.3	1256.6798	0.0170	AEMDILTVPIR
9.2	1256.6724	0.0244	AIEAVKNEVER
8.7	1256.6724	0.0244	EAESKLGASPLR
8.5	1256.7088	-0.0119	AELALEEARKK
7.6	1256.6837	0.0132	KGQTAVAGQAAQK
6.7	1256.6724	0.0245	AELELREELR
6.7	1256.7088	-0.0119	LSLEQSIAVAAR

Mascot: http://www.matrixscience.com/

Mascot Search Results

Peptide View

MS/MS Fragmentation of **SLLTEVETPIR**
Found in **M2**, NYMC X-181

Match to Query 1136: 1298.708226 from(650.361389,2+) intensity(2209400.0000)
Title: B111206_007.04297.04297.2
Data file B111206_007.mgf.mascot

Click mouse within plot area to zoom in by factor of two about that point
Or, Plot from 200 to 1150 Da Full range
Label all possible matches ○ Label matches used for scoring ◉

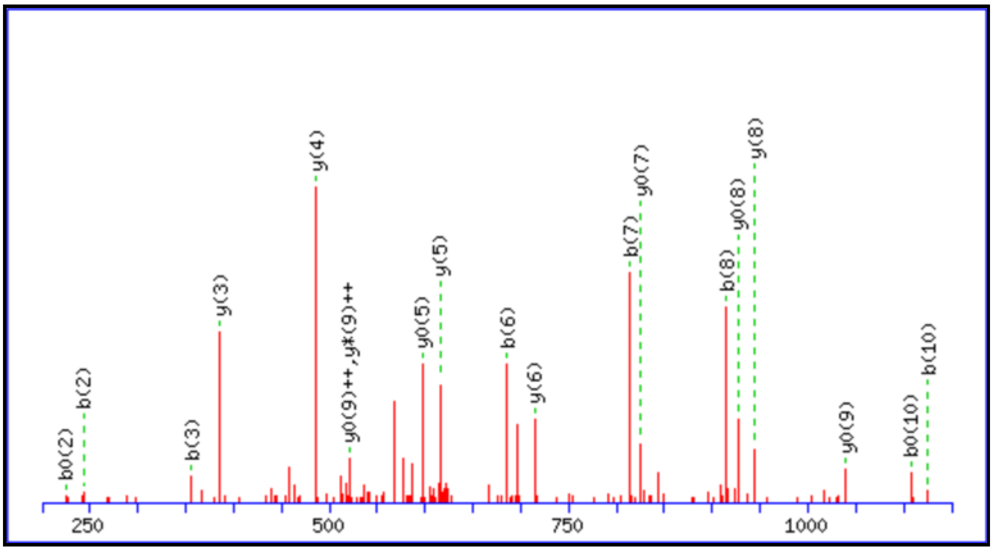

Monoisotopic mass of neutral peptide Mr(calc): 1298.7082
Fixed modifications: Carbamidomethyl (C) (apply to specified residues or termini only)
Variable modifications:
N-term : Acetyl (Protein N-term)
Ions Score: 57 **Expect:** 0.0002
Matches : 19/94 fragment ions using 20 most intense peaks (help)

#	b	b^{++}	b^0	b^{0++}	Seq.	y	y^{++}	y*	y^{*++}	y^0	y^{0++}	#
**1**	130.0499	65.5286	112.0393	56.5233	**S**							**11**
**2**	***243.1339***	122.0706	225.1234	113.0653	**L**	1170.6729	585.8401	1153.6463	577.3268	1152.6623	576.8348	**10**
**3**	***356.2180***	178.6126	338.2074	169.6074	**L**	1057.5888	529.2980	1040.5623	520.7848	1039.5782	520.2928	**9**
**4**	457.2657	229.1365	439.2551	220.1312	**T**	***944.5047***	472.7560	927.4782	464.2427	926.4942	463.7507	**8**
**5**	586.3083	293.6578	568.2977	284.6525	**E**	843.4571	422.2322	826.4305	413.7189	825.4465	413.2269	**7**
**6**	***685.3767***	343.1920	667.3661	334.1867	**V**	***714.4145***	357.7109	697.3879	349.1976	696.4039	348.7056	**6**
**7**	***814.4193***	407.7133	796.4087	398.7080	**E**	***615.3461***	308.1767	598.3195	299.6634	597.3355	299.1714	**5**
**8**	***915.4670***	458.2371	897.4564	449.2318	**T**	***486.3035***	243.6554	469.2769	235.1421	468.2929	234.6501	**4**
**9**	1012.5197	506.7635	994.5092	497.7582	**P**	***385.2558***	193.1315	368.2292	184.6183			**3**
**10**	***1125.6038***	563.3055	1107.5932	554.3002	**I**	288.2030	144.6051	271.1765	136.0919			**2**
**11**					**R**	175.1190	88.0631	158.0924	79.5498			**1**

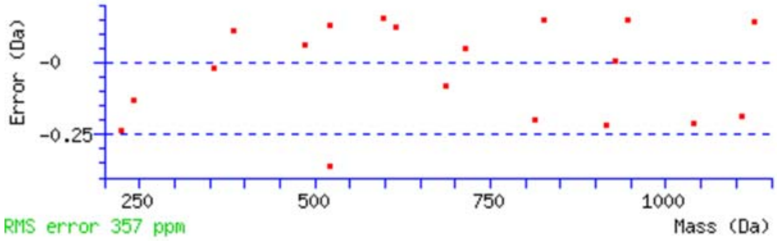

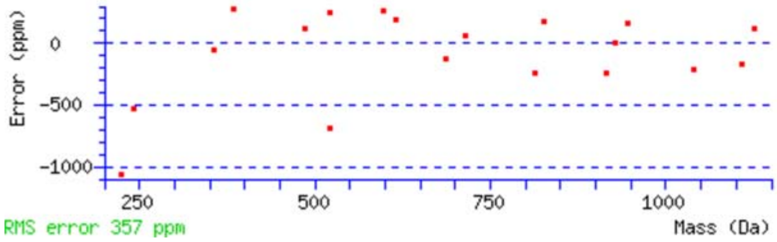

NCBI **BLAST** search of SLLTEVETPIR
(Parameters: blastp, nr protein database, expect=20000, no filter, PAM30)
Other BLAST web gateways

All matches to this query

Score	Mr(calc):	Delta	Sequence
56.7	1298.7082	0.0001	SLLTEVETPIR
16.2	1298.7241	-0.0159	MRPTERLQLR
15.9	1298.6942	0.0140	IQENVRNAIGGK
15.3	1297.7241	0.9841	LQIQINLDAIR
14.9	1298.6990	0.0093	GRGRGLGCTPLR
14.7	1297.7241	0.9841	LQIQINLDAIR
11.0	1297.6990	1.0093	LENKKPNLEGR
10.9	1298.6942	0.0140	SQLAGDLIQQAR
10.8	1298.7194	-0.0112	IETEISNKPLR
10.2	1297.6990	1.0092	SSLPVSGGSAPLAR

Mascot: http://www.matrixscience.com/

X187
N-terminal peptide spectra

Mascot Search Results

Peptide View

MS/MS Fragmentation of **MDVNPTLLFLK**
Found in **PB1**, NYMC X-187

Match to Query 1582: 1347.711522 from(674.863037,2+) intensity(826449.0000)
Title: B111206_009.05064.05064.2
Data file B111206_009.mgf.mascot

Click mouse within plot area to zoom in by factor of two about that point
Or, Plot from 100 to 1300 Da Full range
Label all possible matches ○ Label matches used for scoring ◉

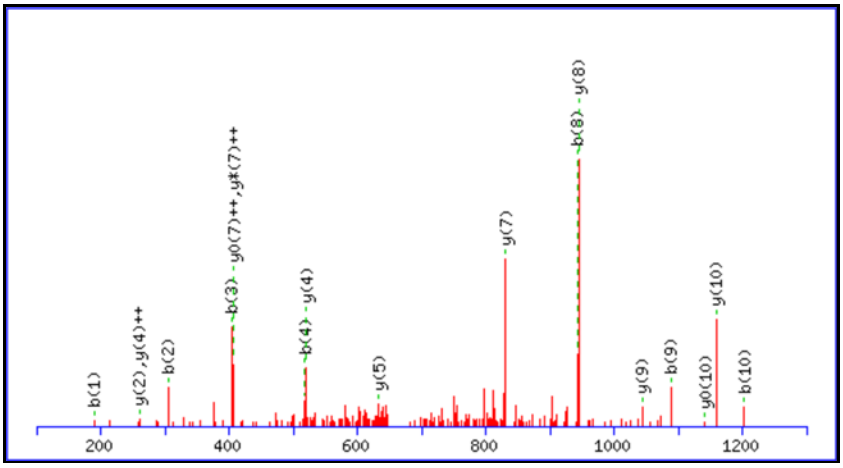

Monoisotopic mass of neutral peptide Mr(calc): 1347.7108
Fixed modifications: Carbamidomethyl (C) (apply to specified residues or termini only)
Variable modifications:
N-term : Acetyl (Protein N-term)
M1 : Oxidation (M), with neutral losses 0.0000(shown in table), 63.9983
Ions Score: 72 **Expect:** 1.1e-05
Matches : 19/154 fragment ions using 21 most intense peaks (help)

#	b	b^{++}	b*	b^{*++}	b^0	b^{0++}	Seq.	y	y^{++}	y*	y^{*++}	y^0	y^{0++}	#
1	**190.0532**	95.5303					M							11
2	**305.0802**	153.0437			287.0696	144.0384	D	**1159.6721**	580.3397	1142.6456	571.8264	1141.6616	571.3344	10
3	**404.1486**	202.5779			386.1380	193.5727	V	**1044.6452**	522.8262	1027.6186	514.3130	1026.6346	513.8210	9
4	**518.1915**	259.5994	501.1650	251.0861	500.1810	250.5941	N	**945.5768**	473.2920	928.5502	464.7788	927.5662	464.2867	8
5	615.2443	308.1258	598.2177	299.6125	597.2337	299.1205	P	**831.5339**	416.2706	814.5073	407.7573	813.5233	407.2653	7
6	716.2920	358.6496	699.2654	350.1363	698.2814	349.6443	T	734.4811	367.7442	717.4545	359.2309	716.4705	358.7389	6
7	829.3760	415.1917	812.3495	406.6784	811.3655	406.1864	L	**633.4334**	317.2203	616.4069	308.7071			5
8	**942.4601**	471.7337	925.4335	463.2204	924.4495	462.7284	L	**520.3493**	260.6783	503.3228	252.1650			4
9	**1089.5285**	545.2679	1072.5020	536.7546	1071.5179	536.2626	F	**407.2653**	204.1363	390.2387	195.6230			3
10	**1202.6126**	601.8099	1185.5860	593.2967	1184.6020	592.8046	L	**260.1969**	130.6021	243.1703	122.0888			2
11							K	147.1128	74.0600	130.0863	65.5468			1

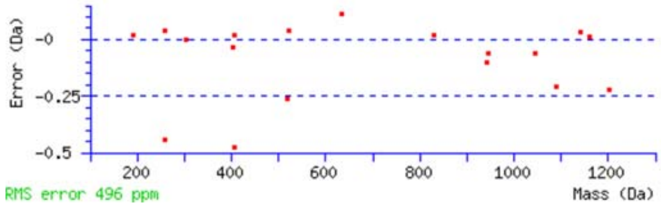

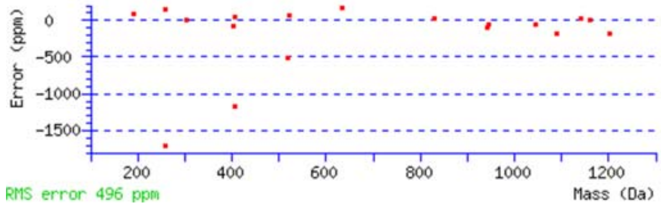

NCBI **BLAST** search of MDVNPTLLFLK
(Parameters: blastp, nr protein database, expect=20000, no filter, PAM30)
Other BLAST web gateways

All matches to this query

Score	Mr(calc):	Delta	Sequence
72.3	1347.7108	0.0007	MDVNPTLLFLK
19.2	1347.6955	0.0160	NGTILVDNMLIK
11.9	1346.7115	1.0000	NGTILVDNMLIK
11.0	1347.7254	-0.0139	MKIMGEVLNGIK
8.7	1347.6895	0.0220	TSSQTFHSGRIK
7.9	1345.6812	2.0303	KCLNDGVHYLK
7.7	1347.7068	0.0047	SGDPKDTKMLIK
7.3	1347.6922	0.0193	AALDFVVEEDLK
5.9	1347.6955	0.0160	NVLDQIKEMLK
4.8	1347.6881	0.0234	NTAGTIGKSQELK

Mascot: http://www.matrixscience.com/

MATRIX SCIENCE Mascot Search Results

Peptide View

MS/MS Fragmentation of **SLLTEVETYVLSIIPSGPLK**
Found in **M1**, NYMC X-187

Match to Query 3612: 2158.213720 from(1080.114136,2+) intensity(188523.0000)
Title: B111206_009.07409.07409.2
Data file B111206_009.mgf.mascot

Click mouse within plot area to zoom in by factor of two about that point
Or, Plot from 300 to 2000 Da Full range
Label all possible matches ○ Label matches used for scoring ◉

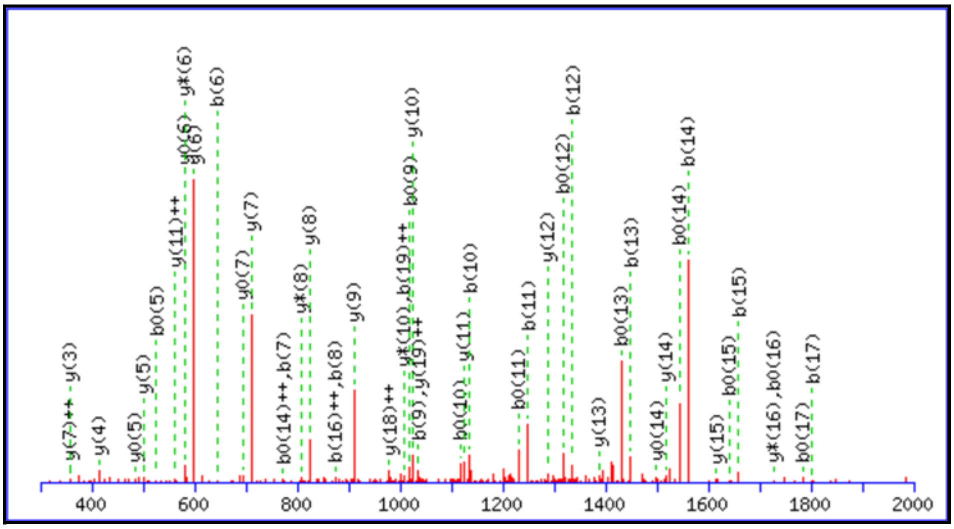

Monoisotopic mass of neutral peptide Mr(calc): 2158.2137
Fixed modifications: Carbamidomethyl (C) (apply to specified residues or termini only)
Ions Score: 47 **Expect:** 0.00031
Matches : 49/182 fragment ions using 122 most intense peaks (help)

#	b	b^{++}	b^{0}	b^{0++}	Seq.	y	y^{++}	y*	y^{*++}	y^{0}	y^{0++}	#
1	88.0393	44.5233	70.0287	35.5180	S							20
2	201.1234	101.0653	183.1128	92.0600	L	2072.1889	1036.5981	2055.1624	1028.0848	2054.1784	1027.5928	19
3	314.2074	157.6074	296.1969	148.6021	L	1959.1049	980.0561	1942.0783	971.5428	1941.0943	971.0508	18
4	415.2551	208.1312	397.2445	199.1259	T	1846.0208	923.5140	1828.9943	915.0008	1828.0102	914.5088	17
5	544.2977	272.6525	526.2871	263.6472	E	1744.9731	872.9902	1727.9466	864.4769	1726.9626	863.9849	16
6	**643.3661**	322.1867	625.3556	313.1814	V	**1615.9305**	808.4689	1598.9040	799.9556	1597.9200	799.4636	15
7	**772.4087**	386.7080	754.3981	377.7027	E	**1516.8621**	758.9347	1499.8356	750.4214	1498.8516	749.9294	14
8	**873.4564**	437.2318	855.4458	428.2266	T	**1387.8195**	694.4134	1370.7930	685.9001	1369.8090	685.4081	13
9	**1036.5197**	518.7635	1018.5092	509.7582	Y	**1286.7719**	643.8896	1269.7453	635.3763	1268.7613	634.8843	12
10	**1135.5881**	568.2977	1117.5776	559.2924	V	**1123.7085**	562.3579	1106.6820	553.8446	1105.6980	553.3526	11
11	**1248.6722**	624.8397	1230.6616	615.8345	L	**1024.6401**	512.8237	1007.6136	504.3104	1006.6295	503.8184	10
12	**1335.7042**	668.3558	1317.6937	659.3505	S	**911.5560**	456.2817	894.5295	447.7684	893.5455	447.2764	9
13	**1448.7883**	724.8978	1430.7777	715.8925	I	**824.5240**	412.7656	807.4975	404.2524	806.5135	403.7604	8
14	**1561.8724**	781.4398	1543.8618	772.4345	I	**711.4400**	356.2236	694.4134	347.7103	693.4294	347.2183	7
15	**1658.9251**	829.9662	1640.9146	820.9609	P	**598.3559**	299.6816	581.3293	291.1683	580.3453	290.6763	6
16	1745.9571	873.4822	1727.9466	864.4769	S	**501.3031**	251.1552	484.2766	242.6419	483.2926	242.1499	5
17	**1802.9786**	901.9929	1784.9680	892.9877	G	**414.2711**	207.6392	397.2445	199.1259			4
18	1900.0314	950.5193	1882.0208	941.5140	P	**357.2496**	179.1285	340.2231	170.6152			3
19	2013.1154	1007.0614	1995.1049	998.0561	L	260.1969	130.6021	243.1703	122.0888			2

**20**					**K**	147.1128	74.0600	130.0863	65.5468			**1**

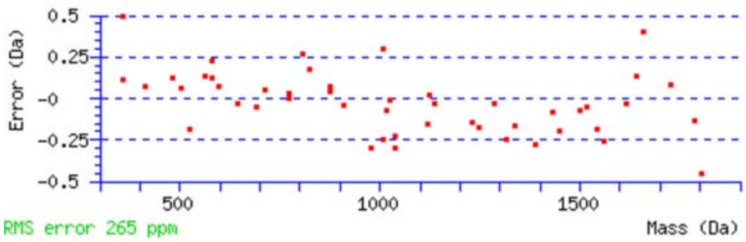

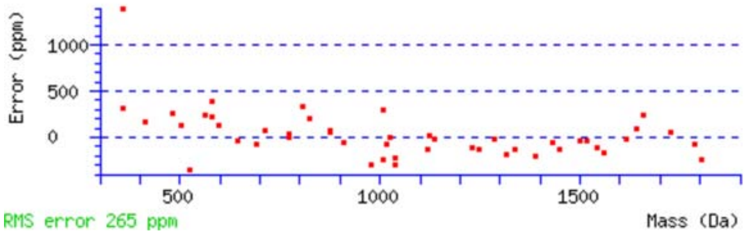

NCBI **BLAST** search of SLLTEVETYVLSIIPSGPLK
(Parameters: blastp, nr protein database, expect=20000, no filter, PAM30)
Other BLAST web gateways

All matches to this query

Score	Mr(calc):	Delta	Sequence
47.4	2158.2137	0.0001	SLLTEVETYVLSIIPSGPLK
9.4	2158.1971	0.0167	QGVVRGSRPPVSRALPQPEK
6.9	2158.2435	-0.0298	QVITIIILLLLCKASDFR
5.8	2157.2131	1.0007	QGVVRGSRPPVSRALPQPEK
5.3	2158.1972	0.0165	WRVRLAIIEYMPLLAGQL
5.3	2158.2150	-0.0013	GPLVFLEQALIQYALQSLR
5.2	2158.2126	0.0011	IAQGISGLVRPLLSGLKVSK
4.3	2157.1762	1.0375	VRTELKPMMQVPARLLSR
1.2	2157.1727	1.0410	KKEVLIAGLKPSLAVYLR
1.1	2157.2132	1.0005	WRVRLAIIEYMPLLAGQL

Mascot: http://www.matrixscience.com/

Mascot Search Results

Peptide View

MS/MS Fragmentation of **SLLTEVETYVLSIIPSGPLK**
Found in **M1**, NYMC X-187

Match to Query 3594: 2200.232762 from(1101.123657,2+) intensity(57296452.0000)
Title: B120210_013.07475.07475.2
Data file B120210_013.mgf.mascot

Click mouse within plot area to zoom in by factor of two about that point
Or, Plot from 300 to 1900 Da Full range
Label all possible matches ○ Label matches used for scoring ◉

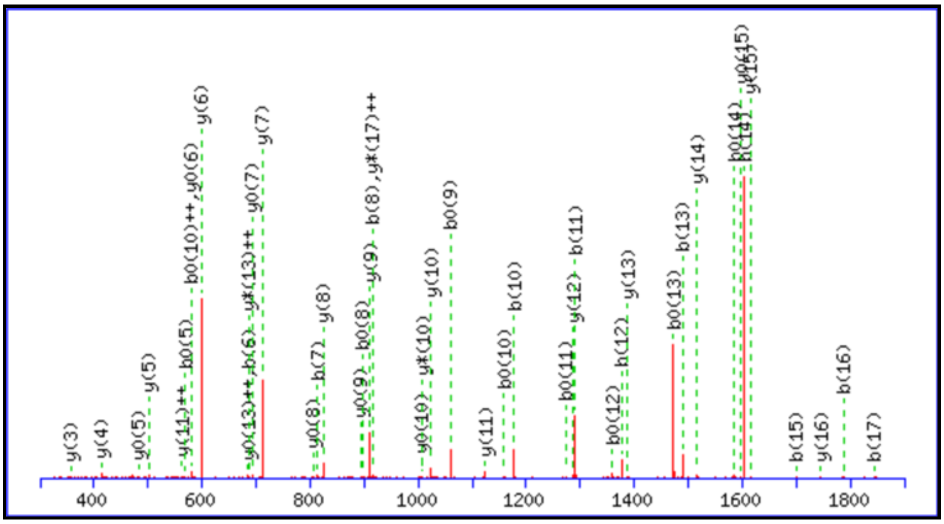

Monoisotopic mass of neutral peptide Mr(calc): 2200.2242
Fixed modifications: Carbamidomethyl (C) (apply to specified residues or termini only)
Variable modifications:
N-term : Acetyl (Protein N-term)
Ions Score: 90 **Expect:** 4.3e-08
Matches : 46/182 fragment ions using 75 most intense peaks (help)

#	b	b^{++}	b^0	b^{0++}	Seq.	y	y^{++}	y*	y^{*++}	y^0	y^{0++}	#
1	130.0499	65.5286	112.0393	56.5233	S							20
2	243.1339	122.0706	225.1234	113.0653	L	2072.1889	1036.5981	2055.1624	1028.0848	2054.1784	1027.5928	19
3	356.2180	178.6126	338.2074	169.6074	L	1959.1049	980.0561	1942.0783	971.5428	1941.0943	971.0508	18
4	457.2657	229.1365	439.2551	220.1312	T	1846.0208	923.5140	1828.9943	915.0008	1828.0102	914.5088	17
5	586.3083	293.6578	568.2977	284.6525	E	1744.9731	872.9902	1727.9466	864.4769	1726.9626	863.9849	16
6	685.3767	343.1920	667.3661	334.1867	V	1615.9305	808.4689	1598.9040	799.9556	1597.9200	799.4636	15
7	814.4193	407.7133	796.4087	398.7080	E	1516.8621	758.9347	1499.8356	750.4214	1498.8516	749.9294	14
8	915.4670	458.2371	897.4564	449.2318	T	1387.8195	694.4134	1370.7930	685.9001	1369.8090	685.4081	13
9	1078.5303	539.7688	1060.5197	530.7635	Y	1286.7719	643.8896	1269.7453	635.3763	1268.7613	634.8843	12
10	1177.5987	589.3030	1159.5881	580.2977	V	1123.7085	562.3579	1106.6820	553.8446	1105.6980	553.3526	11
11	1290.6828	645.8450	1272.6722	636.8397	L	1024.6401	512.8237	1007.6136	504.3104	1006.6295	503.8184	10
12	1377.7148	689.3610	1359.7042	680.3558	S	911.5560	456.2817	894.5295	447.7684	893.5455	447.2764	9
13	1490.7989	745.9031	1472.7883	736.8978	I	824.5240	412.7656	807.4975	404.2524	806.5135	403.7604	8
14	1603.8829	802.4451	1585.8724	793.4398	I	711.4400	356.2236	694.4134	347.7103	693.4294	347.2183	7
15	1700.9357	850.9715	1682.9251	841.9662	P	598.3559	299.6816	581.3293	291.1683	580.3453	290.6763	6
16	1787.9677	894.4875	1769.9571	885.4822	S	501.3031	251.1552	484.2766	242.6419	483.2926	242.1499	5
17	1844.9892	922.9982	1826.9786	913.9929	G	414.2711	207.6392	397.2445	199.1259			4
18	1942.0419	971.5246	1924.0314	962.5193	P	357.2496	179.1285	340.2231	170.6152			3

19	2055.1260	1028.0666	2037.1154	1019.0614	L	260.1969	130.6021	243.1703	122.0888			2
20					K	147.1128	74.0600	130.0863	65.5468			1

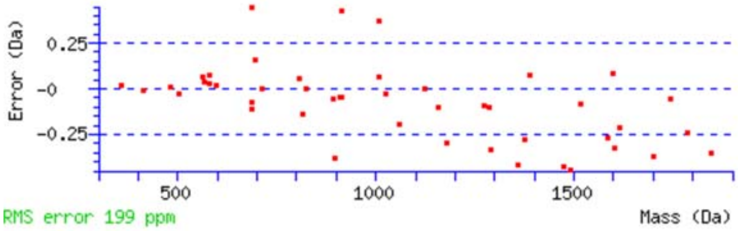

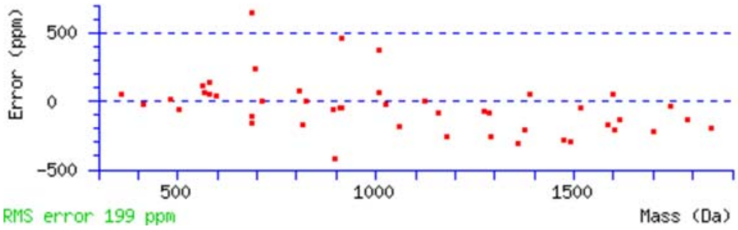

NCBI **BLAST** search of SLLTEVETYVLSIIPSGPLK
(Parameters: blastp, nr protein database, expect=20000, no filter, PAM30)
Other BLAST web gateways

All matches to this query

Score	Mr(calc):	Delta	Sequence
90.3	2200.2242	0.0085	SLLTEVETYVLSIIPSGPLK
11.0	2199.1899	1.0429	AAAGAVRLLVVQPQPEEQPPK
11.0	2199.1899	1.0429	AAAGAVRLLVVQPQPEEQPPK
9.3	2199.1899	1.0429	AAAGAVRLLVVQPQPEEQPPK
5.8	2200.2119	0.0208	LLKEPSGADIALLKLNSPIK
5.3	2198.2535	1.9793	IIGEKYQALNSRLLIGRPR
4.5	2199.2099	1.0229	GMAAPGPPQLPWLRLGPRLR
4.0	2200.2705	-0.0377	VLPALRIRPHGAPQPPTQPR
3.5	2200.2467	-0.0139	GLDELALIYLATIQAVALGTR
2.5	2198.1976	2.0351	YTRPTPVQKHAIPIIKEK

Mascot: http://www.matrixscience.com/

{MATRIX SCIENCE} Mascot Search Results

Peptide View

MS/MS Fragmentation of **SLLTEVETYVLSIIPSGPLKAEIAQR**
Found in **M1**, NYMC X-187

Match to Query 5103: 2868.596154 from(957.205994,3+) intensity(1350380.0000)
Title: B111206_009.07592.07592.3
Data file B111206_009.mgf.mascot

Click mouse within plot area to zoom in by factor of two about that point
Or, Plot from 200 to 1900 Da Full range
Label all possible matches ○ Label matches used for scoring ◉

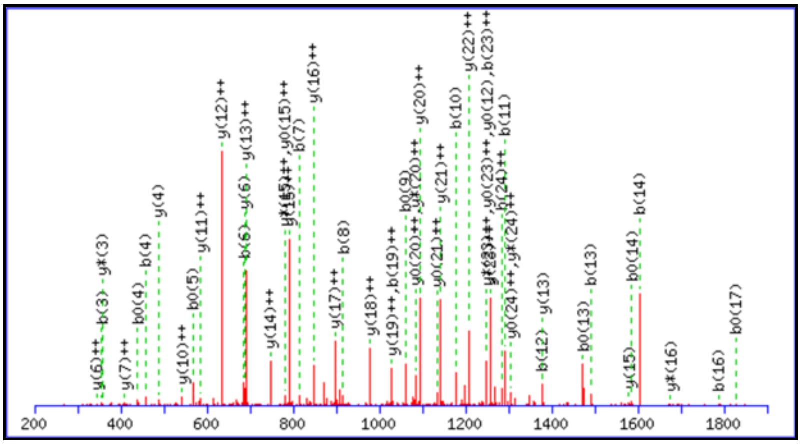

Monoisotopic mass of neutral peptide Mr(calc): 2868.5848
Fixed modifications: Carbamidomethyl (C) (apply to specified residues or termini only)
Variable modifications:
N-term : Acetyl (Protein N-term)
Ions Score: 116 **Expect:** 8.1e-11
Matches : 52/254 fragment ions using 64 most intense peaks (help)

#	b	b^{++}	b*	b^{*++}	b^{0}	b^{0++}	Seq.	y	y^{++}	y*	y^{*++}	y^{0}	y^{0++}	#
1	130.0499	65.5286			112.0393	56.5233	S							26
2	243.1339	122.0706			225.1234	113.0653	L	2740.5495	1370.7784	2723.5230	1362.2651	2722.5389	1361.7731	25
3	**356.2180**	178.6126			338.2074	169.6074	L	2627.4654	1314.2364	2610.4389	1305.7231	2609.4549	1305.2311	24
4	**457.2657**	229.1365			439.2551	220.1312	T	2514.3814	1257.6943	2497.3548	1249.1811	2496.3708	1248.6890	23
5	586.3083	293.6578			568.2977	284.6525	E	2413.3337	1207.1705	2396.3072	1198.6572	2395.3231	1198.1652	22
6	**685.3767**	343.1920			667.3661	334.1867	V	2284.2911	1142.6492	2267.2646	1134.1359	2266.2805	1133.6439	21
7	**814.4193**	407.7133			796.4087	398.7080	E	2185.2227	1093.1150	2168.1961	1084.6017	2167.2121	1084.1097	20
8	**915.4670**	458.2371			897.4564	449.2318	T	2056.1801	1028.5937	2039.1536	1020.0804	2038.1695	1019.5884	19
9	1078.5303	539.7688			1060.5197	530.7635	Y	1955.1324	978.0699	1938.1059	969.5566	1937.1219	969.0646	18
10	**1177.5987**	589.3030			1159.5881	580.2977	V	1792.0691	896.5382	1775.0425	888.0249	1774.0585	887.5329	17
11	**1290.6828**	645.8450			1272.6722	636.8397	L	1693.0007	847.0040	1675.9741	838.4907	1674.9901	837.9987	16
12	**1377.7148**	689.3610			1359.7042	680.3558	S	**1579.9166**	790.4619	1562.8901	781.9487	1561.9061	781.4567	15
13	**1490.7989**	745.9031			1472.7883	736.8978	I	1492.8846	746.9459	1475.8580	738.4327	1474.8740	737.9407	14
14	**1603.8829**	802.4451			1585.8724	793.4398	I	**1379.8005**	690.4039	1362.7740	681.8906	1361.7900	681.3986	13
15	1700.9357	850.9715			1682.9251	841.9662	P	1266.7165	633.8619	1249.6899	625.3486	1248.7059	624.8566	12
16	**1787.9677**	894.4875			1769.9571	885.4822	S	1169.6637	585.3355	1152.6371	576.8222	1151.6531	576.3302	11
17	1844.9892	922.9982			1826.9786	913.9929	G	1082.6317	541.8195	1065.6051	533.3062	1064.6211	532.8142	10
18	1942.0419	971.5246			1924.0314	962.5193	P	1025.6102	513.3087	1008.5837	504.7955	1007.5996	504.3035	9
19	2055.1260	**1028.0666**			2037.1154	1019.0614	L	928.5574	464.7824	911.5309	456.2691	910.5469	455.7771	8
20	2183.2210	1092.1141	2166.1944	1083.6008	2165.2104	1083.1088	K	815.4734	408.2403	798.4468	399.7271	797.4628	399.2350	7
21	2254.2581	1127.6327	2237.2315	1119.1194	2236.2475	1118.6274	A	**687.3784**	344.1928	670.3519	335.6796	669.3679	335.1876	6
22	2383.3007	1192.1540	2366.2741	1183.6407	2365.2901	1183.1487	E	616.3413	308.6743	599.3148	300.1610	598.3307	299.6690	5
23	2496.3847	**1248.6960**	2479.3582	1240.1827	2478.3742	1239.6907	I	**487.2987**	244.1530	470.2722	235.6397			4
24	2567.4219	**1284.2146**	2550.3953	1275.7013	2549.4113	1275.2093	A	374.2146	187.6110	357.1881	179.0977			3
25	2695.4804	1348.2439	2678.4539	1339.7306	2677.4699	1339.2386	Q	303.1775	152.0924	286.1510	143.5791			2
26							R	175.1190	88.0631	158.0924	79.5498			1

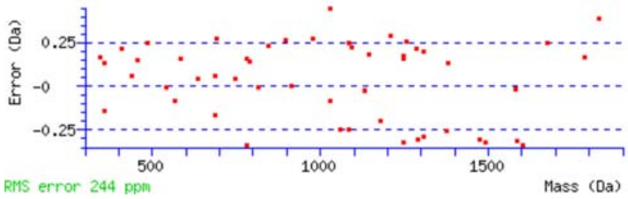

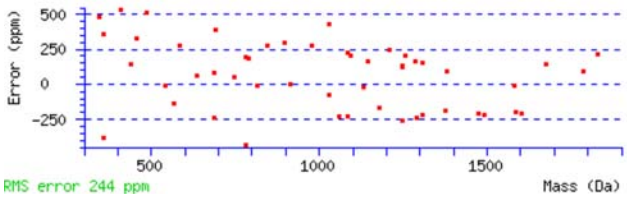

NCBI **BLAST** search of SLLTEVETYVLSIIPSGPLKAEIAQR
(Parameters: blastp, nr protein database, expect=20000, no filter, PAM30)
Other BLAST web gateways

All matches to this query

Score	Mr(calc):	Delta	Sequence
115.8	2868.5848	0.0114	SLLTEVETYVLSIIPSGPLKAEIAQR
7.7	2866.6006	1.9955	MVHTTSPLLLLLLLSLALVAPSLSAR
7.7	2866.6006	1.9955	MVHTTSPLLLLLLLSLALVAPSLSAR
7.0	2867.6054	0.9908	VHATSPLLLLLLLSLALVAPGLSARK
7.0	2867.6054	0.9908	VHATSPLLLLLLLSLALVAPGLSARK
4.7	2868.5722	0.0240	WGPGSHLAAVRSAAELRLLAELLNAXR
2.5	2867.6345	0.9617	KQAKENKPLIPQPQILGLTASPGVGGAR
2.0	2866.5569	2.0393	LVAVAQSGLPLRVESLNTVKDLPLPR
2.0	2866.5569	2.0393	LVAVAQSGLPLRVESLNTVKDLPLPR
1.9	2868.5484	0.0478	RELLGTYIKQGTEIVPVGEALLENPK

Mascot: http://www.matrixscience.com/

{MATRIX SCIENCE} Mascot Search Results

Peptide View

MS/MS Fragmentation of **SLLTEVETYVLSIIPSGPLKAEIAQRLEDVFAGK**
Found in **M1**, NYMC X-187

Match to Query 5011: 3728.034081 from(1243.685303,3+) intensity(13999260.0000)
Title: B120210_013.07469.07469.3
Data file B120210_013.mgf.mascot

Click mouse within plot area to zoom in by factor of two about that point
Or, Plot from 400 to 2000 Da Full range
Label all possible matches ○ Label matches used for scoring ◉

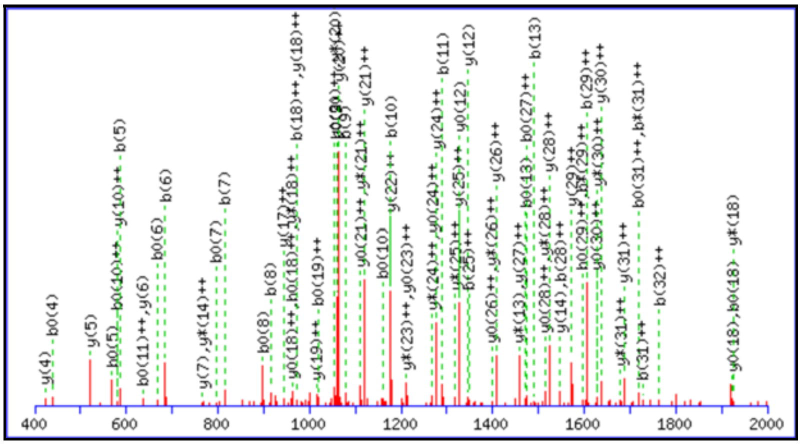

Monoisotopic mass of neutral peptide Mr(calc): 3728.0287
Fixed modifications: Carbamidomethyl (C) (apply to specified residues or termini only)
Variable modifications:
N-term : Acetyl (Protein N-term)
Ions Score: 51 **Expect:** 2.8e-05
Matches : 76/348 fragment ions using 137 most intense peaks (help)

#	b	b^{++}	b*	b^{*++}	b^0	b^{0++}	Seq.	y	y^{++}	y*	y^{*++}	y^0	y^{0++}	#
1	130.0499	65.5286			112.0393	56.5233	S							34
2	243.1339	122.0706			225.1234	113.0653	L	3599.9935	1800.5004	3582.9669	1791.9871	3581.9829	1791.4951	33
3	356.2180	178.6126			338.2074	169.6074	L	3486.9094	1743.9583	3469.8829	1735.4451	3468.8989	1734.9531	32
4	457.2657	229.1365			439.2551	220.1312	T	3373.8254	1687.4163	3356.7988	1678.9030	3355.8148	1678.4110	31
5	**586.3083**	293.6578			568.2977	284.6525	E	3272.7777	1636.8925	3255.7511	1628.3792	3254.7671	1627.8872	30
6	**685.3767**	343.1920			667.3661	334.1867	V	3143.7351	1572.3712	3126.7085	1563.8579	3125.7245	1563.3659	29
7	**814.4193**	407.7133			796.4087	398.7080	E	3044.6667	1522.8370	3027.6401	1514.3237	3026.6561	1513.8317	28
8	**915.4670**	458.2371			897.4564	449.2318	T	2915.6241	1458.3157	2898.5975	1449.8024	2897.6135	1449.3104	27
9	**1078.5303**	539.7688			1060.5197	530.7635	Y	2814.5764	1407.7918	2797.5498	1399.2786	2796.5658	1398.7866	26
10	**1177.5987**	589.3030			1159.5881	580.2977	V	2651.5131	1326.2602	2634.4865	1317.7469	2633.5025	1317.2549	25
11	**1290.6828**	645.8450			1272.6722	636.8397	L	2552.4447	1276.7260	2535.4181	1268.2127	2534.4341	1267.7207	24
12	1377.7148	689.3610			1359.7042	680.3558	S	2439.3606	1220.1839	2422.3340	1211.6707	2421.3500	1211.1786	23
13	**1490.7989**	745.9031			1472.7883	736.8978	I	2352.3286	1176.6679	2335.3020	1168.1546	2334.3180	1167.6626	22
14	1603.8829	802.4451			1585.8724	793.4398	I	2239.2445	1120.1259	2222.2179	1111.6126	2221.2339	1111.1206	21
15	1700.9357	850.9715			1682.9251	841.9662	P	2126.1604	1063.5839	2109.1339	1055.0706	2108.1499	1054.5786	20
16	1787.9677	894.4875			1769.9571	885.4822	S	2029.1077	1015.0575	2012.0811	1006.5442	2011.0971	1006.0522	19
17	1844.9892	922.9982			1826.9786	913.9929	G	1942.0756	971.5415	1925.0491	963.0282	1924.0651	962.5362	18
18	1942.0419	***971.5246***			1924.0314	962.5193	P	1885.0542	943.0307	1868.0276	934.5175	1867.0436	934.0254	17
19	2055.1260	1028.0666			2037.1154	1019.0614	L	1788.0014	894.5043	1770.9749	885.9911	1769.9908	885.4991	16
20	2183.2210	1092.1141	2166.1944	1083.6008	2165.2104	1083.1088	K	1674.9173	837.9623	1657.8908	829.4490	1656.9068	828.9570	15
21	2254.2581	1127.6327	2237.2315	1119.1194	2236.2475	1118.6274	A	***1546.8224***	773.9148	1529.7958	765.4016	1528.8118	764.9095	14
22	2383.3007	1192.1540	2366.2741	1183.6407	2365.2901	1183.1487	E	1475.7853	738.3963	1458.7587	729.8830	1457.7747	729.3910	13
23	2496.3847	1248.6960	2479.3582	1240.1827	2478.3742	1239.6907	I	***1346.7427***	673.8750	1329.7161	665.3617	1328.7321	664.8697	12
24	2567.4219	1284.2146	2550.3953	1275.7013	2549.4113	1275.2093	A	1233.6586	617.3329	1216.6321	608.8197	1215.6480	608.3277	11
25	2695.4804	***1348.2439***	2678.4539	1339.7306	2677.4699	1339.2386	Q	1162.6215	581.8144	1145.5950	573.3011	1144.6109	572.8091	10
26	2851.5815	1426.2944	2834.5550	1417.7811	2833.5710	1417.2891	R	1034.5629	517.7851	1017.5364	509.2718	1016.5524	508.7798	9
27	2964.6656	1482.8364	2947.6391	1474.3232	2946.6550	1473.8312	L	878.4618	439.7345	861.4353	431.2213	860.4512	430.7293	8

**28**	3093.7082	***1547.3577***	3076.6816	1538.8445	3075.6976	1538.3525	**E**	***765.3777***	383.1925	748.3512	374.6792	747.3672	374.1872	**7**
**29**	3208.7351	***1604.8712***	3191.7086	1596.3579	3190.7246	1595.8659	**D**	***636.3352***	318.6712	619.3086	310.1579	618.3246	309.6659	**6**
**30**	3307.8036	1654.4054	3290.7770	1645.8921	3289.7930	1645.4001	**V**	***521.3082***	261.1577	504.2817	252.6445			**5**
**31**	3454.8720	***1727.9396***	3437.8454	1719.4263	3436.8614	1718.9343	**F**	***422.2398***	211.6235	405.2132	203.1103			**4**
**32**	3525.9091	***1763.4582***	3508.8825	1754.9449	3507.8985	1754.4529	**A**	275.1714	138.0893	258.1448	129.5761			**3**
**33**	3582.9305	1791.9689	3565.9040	1783.4556	3564.9200	1782.9636	**G**	204.1343	102.5708	187.1077	94.0575			**2**
**34**							**K**	147.1128	74.0600	130.0863	65.5468			**1**

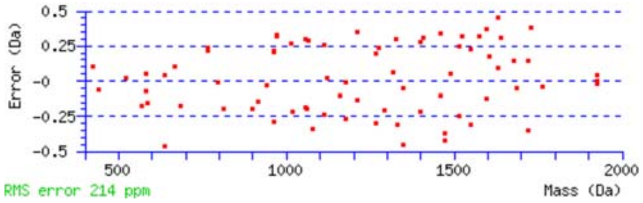

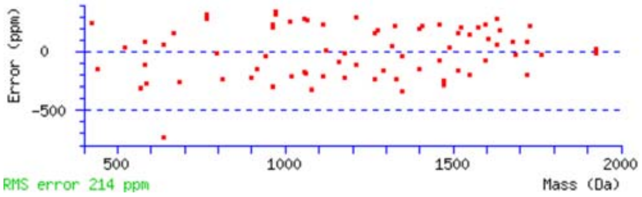

NCBI **BLAST** search of SLLTEVETYVLSIIPSGPLKAEIAQRLEDVFAGK
(Parameters: blastp, nr protein database, expect=20000, no filter, PAM30)
Other BLAST web gateways

All matches to this query

Score	Mr(calc):	Delta	Sequence
50.8	3728.0287	0.0053	SLLTEVETYVLSIIPSGPLKAEIAQRLEDVFAGK
2.0	3726.0597	1.9744	HPPATVDSNGYMMMSPSGSYSPDSGSAGYGK
2.0	3726.0597	1.9744	HPPATVDSNGYMMMSPSGSYSPDSGSAGYGK
0.9	3727.0164	1.0177	EMVYDCTSSSFDGIIAMMSPEDSWVSK
0.4	3726.0740	1.9601	STNGKNPTCIVSHATLNETRSINCSSR
0.2	3728.0215	0.0126	NSMTPNPGYQPSMNTSDMMGRMSYEPNK
0.0	3728.0215	0.0126	NSMTPNPGYQPSMNTSDMMGRMSYEPNK

Mascot: http://www.matrixscience.com/

Mascot Search Results

Peptide View

MS/MS Fragmentation of **SLLTEVETPIR**
Found in **M2**, NYMC X-187

Match to Query 1376: 1298.708836 from(650.361694,2+) intensity(2242170.0000)
Title: B111206_009.04601.04601.2
Data file B111206_009.mgf.mascot

Click mouse within plot area to zoom in by factor of two about that point
Or, Plot from 150 to 1200 Da Full range
Label all possible matches ○ Label matches used for scoring ◉

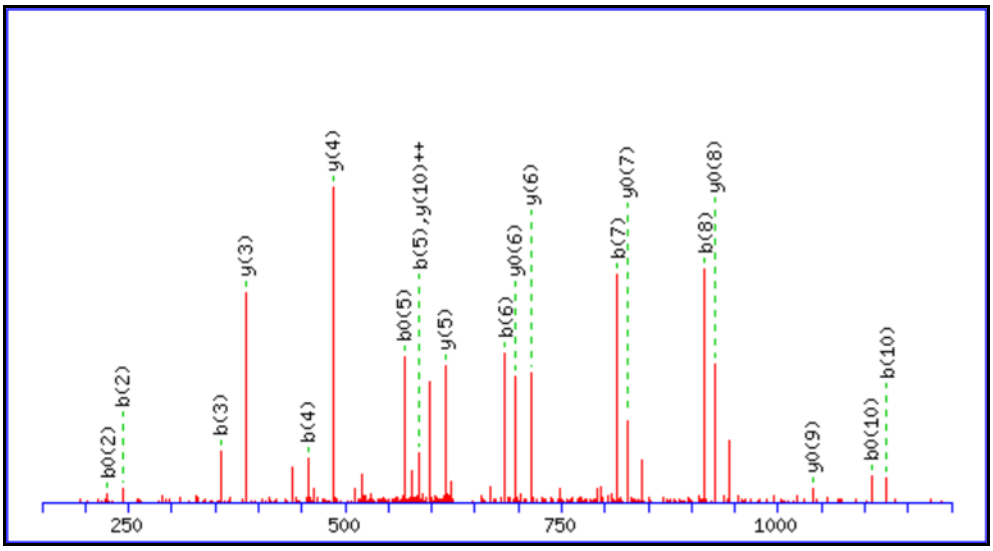

Monoisotopic mass of neutral peptide Mr(calc): 1298.7082
Fixed modifications: Carbamidomethyl (C) (apply to specified residues or termini only)
Variable modifications:
N-term : Acetyl (Protein N-term)
Ions Score: 84 **Expect:** 1.8e-07
Matches : 20/94 fragment ions using 20 most intense peaks (help)

#	b	b^{++}	b^{0}	b^{0++}	Seq.	y	y^{++}	y*	y^{*++}	y^{0}	y^{0++}	#
1	130.0499	65.5286	112.0393	56.5233	S							11
2	***243.1339***	122.0706	225.1234	113.0653	L	1170.6729	585.8401	1153.6463	577.3268	1152.6623	576.8348	10
3	***356.2180***	178.6126	338.2074	169.6074	L	1057.5888	529.2980	1040.5623	520.7848	1039.5782	520.2928	9
4	***457.2657***	229.1365	439.2551	220.1312	T	944.5047	472.7560	927.4782	464.2427	926.4942	463.7507	8
5	***586.3083***	293.6578	568.2977	284.6525	E	843.4571	422.2322	826.4305	413.7189	825.4465	413.2269	7
6	***685.3767***	343.1920	667.3661	334.1867	V	**714.4145**	357.7109	697.3879	349.1976	696.4039	348.7056	6
7	***814.4193***	407.7133	796.4087	398.7080	E	**615.3461**	308.1767	598.3195	299.6634	597.3355	299.1714	5
8	***915.4670***	458.2371	897.4564	449.2318	T	**486.3035**	243.6554	469.2769	235.1421	468.2929	234.6501	4
9	1012.5197	506.7635	994.5092	497.7582	P	**385.2558**	193.1315	368.2292	184.6183			3
10	***1125.6038***	563.3055	1107.5932	554.3002	I	288.2030	144.6051	271.1765	136.0919			2
11					R	175.1190	88.0631	158.0924	79.5498			1

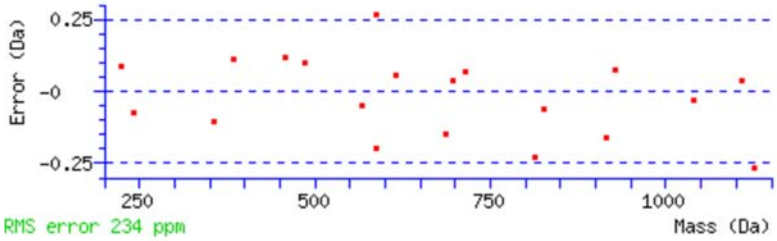

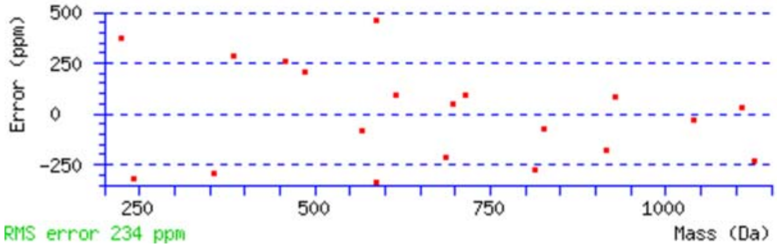

NCBI **BLAST** search of SLLTEVETPIR
(Parameters: blastp, nr protein database, expect=20000, no filter, PAM30)
Other BLAST web gateways

All matches to this query

Score	Mr(calc):	Delta	Sequence
83.8	1298.7082	0.0007	SLLTEVETPIR
11.0	1297.7241	0.9847	DNAALKVTLPQK
10.8	1298.6904	0.0184	MLIQDIPSIPR
10.1	1298.6942	0.0146	IQENVRNAIGGK
8.5	1297.6990	1.0098	TGTLTSDHLVVR
8.3	1296.6826	2.0262	LEASNWLTHVK
7.7	1297.7047	1.0042	FTQDLLKLLK
7.5	1298.6943	0.0146	VQEEIDRVVGR
7.0	1298.6870	0.0218	ELLTDPYAHLK
6.8	1298.7194	-0.0105	SAPQIESKLLGR

Mascot: http://www.matrixscience.com/

MATRIX SCIENCE Mascot Search Results

Peptide View

Influenza B/Brisbane/60/2008
N-terminal peptide spectra

MS/MS Fragmentation of **MDTFITR**
Found in **PA**, B/Brisbane/60/2008

Match to Query 2268: 924.436250 from(463.225401,2+) intensity(7833460.0000)
Title: edflowthruFBV15.22176.22176.2
Data file edflowthruFBV15.mgf.mascot

Click mouse within plot area to zoom in by factor of two about that point
Or, Plot from 50 to 800 Da Full range
Label all possible matches ○ Label matches used for scoring ◉
View spectrum in original style ◉ View spectrum with detailed annotation ○

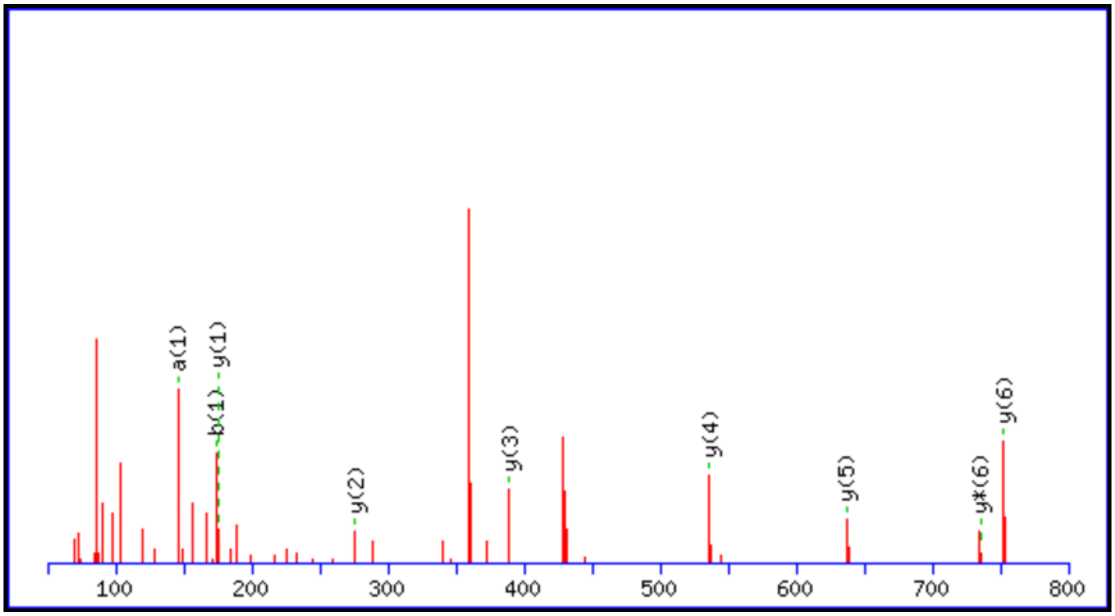

Monoisotopic mass of neutral peptide Mr(calc): 924.4375
Fixed modifications: Carbamidomethyl (C) (apply to specified residues or termini only)
Variable modifications:
N-term : Acetyl (Protein N-term)
Ions Score: 27 **Expect:** 0.029
Matches : 9/48 fragment ions using 26 most intense peaks (help)

#	a	a^{++}	b	b^{++}	Seq.	y	y^{++}	y*	y^{*++}	#
1	**146.0634**	73.5353	**174.0583**	87.5328	M					7
2	261.0904	131.0488	289.0853	145.0463	D	***752.3937***	376.7005	735.3672	368.1872	6
3	362.1380	181.5727	390.1329	195.5701	T	***637.3668***	319.1870	620.3402	310.6738	5
4	509.2064	255.1069	537.2014	269.1043	F	***536.3191***	268.6632	519.2926	260.1499	4
5	622.2905	311.6489	650.2854	325.6464	I	***389.2507***	195.1290	372.2241	186.6157	3
6	723.3382	362.1727	751.3331	376.1702	T	***276.1666***	138.5870	259.1401	130.0737	2
7					R	***175.1190***	88.0631	158.0924	79.5498	1

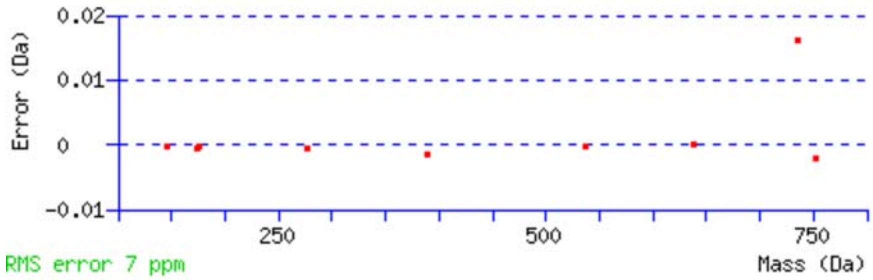

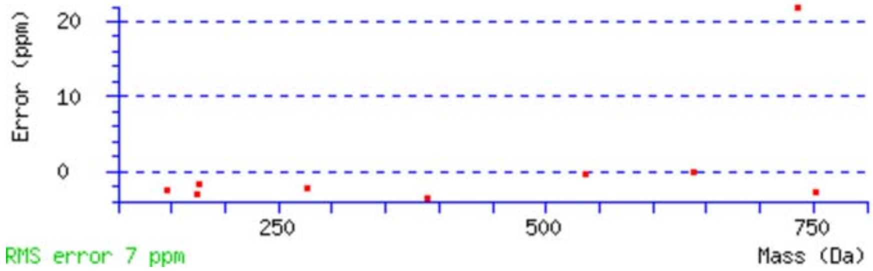

NCBI **BLAST** search of MDTFITR
(Parameters: blastp, nr protein database, expect=20000, no filter, PAM30)
Other BLAST web gateways

All matches to this query

Score	Mr(calc):	Delta	Sequence
27.0	924.4375	-0.0013	MDTFITR
9.9	924.4317	0.0045	LSPVSTK
9.9	924.4317	0.0045	LSPVSTK
8.2	924.4301	0.0061	GSGFGASTK
5.2	924.4408	-0.0046	LMKMEK
5.2	924.4375	-0.0012	LMQFEK
5.2	924.4375	-0.0012	LQMFQK
5.2	924.4375	-0.0012	LQMFQK
4.3	924.4222	0.0140	STLSMEK
3.7	924.4408	-0.0046	LMEMKK

Mascot: http://www.matrixscience.com/

Mascot Search Results

Peptide View

MS/MS Fragmentation of **SNMDIDGINTGTIDKTPEEITSGTSGTTRPIIRPATLAPPSNK**
Found in **NP**, B/Brisbane/60/2008

Match to Query 11238: 4509.250896 from(1128.320000,4+) intensity(14074529.0000)
Title: D120713_002.13013.13013.4
Data file D120713_002.mgf.mascot

Click mouse within plot area to zoom in by factor of two about that point
Or, Plot from 300 to 1800 Da Full range
Label all possible matches ○ Label matches used for scoring ◉
View spectrum in original style ◉ View spectrum with detailed annotation ○

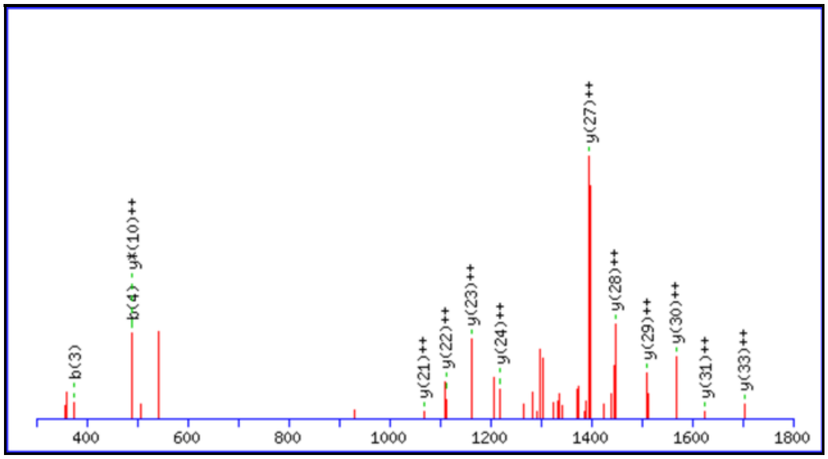

Monoisotopic mass of neutral peptide Mr(calc): 4509.2544
Fixed modifications: Carbamidomethyl (C) (apply to specified residues or termini only)
Variable modifications:
N-term : Acetyl (N-term)
N42 : Deamidated (NQ)
Ions Score: 27 **Expect:** 0.12
Matches : 13/500 fragment ions using 37 most intense peaks (help)

#	a	a^{++}	a*	a^{*++}	b	b^{++}	b*	b^{*++}	Seq.	y	y^{++}	y*	y^{*++}	#
1	102.0550	51.5311			130.0499	65.5286			S					43
2	216.0979	108.5526	199.0713	100.0393	244.0928	122.5500	227.0662	114.0368	N	4381.2192	2191.1132	4364.1926	2182.5999	42
3	347.1384	174.0728	330.1118	165.5595	**375.1333**	188.0703	358.1067	179.5570	M	4267.1762	2134.0918	4250.1497	2125.5785	41
4	462.1653	231.5863	445.1388	223.0730	**490.1602**	245.5837	473.1337	237.0705	D	4136.1357	2068.5715	4119.1092	2060.0582	40
5	575.2494	288.1283	558.2228	279.6150	603.2443	302.1258	586.2177	293.6125	I	4021.1088	2011.0580	4004.0822	2002.5448	39
6	690.2763	345.6418	673.2498	337.1285	718.2712	359.6393	701.2447	351.1260	D	3908.0247	1954.5160	3890.9982	1946.0027	38
7	747.2978	374.1525	730.2712	365.6393	775.2927	388.1500	758.2661	379.6367	G	3792.9978	1897.0025	3775.9712	1888.4893	37
8	860.3818	430.6946	843.3553	422.1813	888.3768	444.6920	871.3502	436.1787	I	3735.9763	1868.4918	3718.9498	1859.9785	36
9	974.4248	487.7160	957.3982	479.2027	1002.4197	501.7135	985.3931	493.2002	N	3622.8923	1811.9498	3605.8657	1803.4365	35
10	1075.4724	538.2399	1058.4459	529.7266	1103.4674	552.2373	1086.4408	543.7240	T	3508.8493	1754.9283	3491.8228	1746.4150	34
11	1132.4939	566.7506	1115.4674	558.2373	1160.4888	580.7481	1143.4623	572.2348	G	3407.8017	1704.4045	3390.7751	1695.8912	33
12	1233.5416	617.2744	1216.5150	608.7612	1261.5365	631.2719	1244.5100	622.7586	T	3350.7802	1675.8937	3333.7536	1667.3805	32
13	1346.6257	673.8165	1329.5991	665.3032	1374.6206	687.8139	1357.5940	679.3006	I	3249.7325	1625.3699	3232.7060	1616.8566	31
14	1461.6526	731.3299	1444.6261	722.8167	1489.6475	745.3274	1472.6210	736.8141	D	3136.6485	1568.8279	3119.6219	1560.3146	30
15	1589.7476	795.3774	1572.7210	786.8641	1617.7425	809.3749	1600.7159	800.8616	K	3021.6215	1511.3144	3004.5950	1502.8011	29
16	1690.7952	845.9013	1673.7687	837.3880	1718.7902	859.8987	1701.7636	851.3854	T	2893.5265	1447.2669	2876.5000	1438.7536	28
17	1787.8480	894.4276	1770.8215	885.9144	1815.8429	908.4251	1798.8164	899.9118	P	2792.4789	1396.7431	2775.4523	1388.2298	27
18	1916.8906	958.9489	1899.8640	950.4357	1944.8855	972.9464	1927.8590	964.4331	E	2695.4261	1348.2167	2678.3996	1339.7034	26
19	2045.9332	1023.4702	2028.9066	1014.9570	2073.9281	1037.4677	2056.9016	1028.9544	E	2566.3835	1283.6954	2549.3570	1275.1821	25
20	2159.0173	1080.0123	2141.9907	1071.4990	2187.0122	1094.0097	2169.9856	1085.4964	I	2437.3409	1219.1741	2420.3144	1210.6608	24

**21**	2260.0649	1130.5361	2243.0384	1122.0228	2288.0598	1144.5336	2271.0333	1136.0203	**T**	2324.2569	1162.6321	2307.2303	1154.1188	**23**
**22**	2347.0970	1174.0521	2330.0704	1165.5388	2375.0919	1188.0496	2358.0653	1179.5363	**S**	2223.2092	1112.1082	2206.1826	1103.5949	**22**
**23**	2404.1184	1202.5629	2387.0919	1194.0496	2432.1133	1216.5603	2415.0868	1208.0470	**G**	2136.1771	1068.5922	2119.1506	1060.0789	**21**
**24**	2505.1661	1253.0867	2488.1396	1244.5734	2533.1610	1267.0841	2516.1345	1258.5709	**T**	2079.1557	1040.0815	2062.1291	1031.5682	**20**
**25**	2592.1981	1296.6027	2575.1716	1288.0894	2620.1930	1310.6002	2603.1665	1302.0869	**S**	1978.1080	989.5576	1961.0815	981.0444	**19**
**26**	2649.2196	1325.1134	2632.1930	1316.6002	2677.2145	1339.1109	2660.1880	1330.5976	**G**	1891.0760	946.0416	1874.0494	937.5284	**18**
**27**	2750.2673	1375.6373	2733.2407	1367.1240	2778.2622	1389.6347	2761.2356	1381.1215	**T**	1834.0545	917.5309	1817.0280	909.0176	**17**
**28**	2851.3150	1426.1611	2834.2884	1417.6478	2879.3099	1440.1586	2862.2833	1431.6453	**T**	1733.0068	867.0071	1715.9803	858.4938	**16**
**29**	3007.4161	1504.2117	2990.3895	1495.6984	3035.4110	1518.2091	3018.3844	1509.6959	**R**	1631.9592	816.4832	1614.9326	807.9699	**15**
**30**	3104.4688	1552.7381	3087.4423	1544.2248	3132.4637	1566.7355	3115.4372	1558.2222	**P**	1475.8580	738.4327	1458.8315	729.9194	**14**
**31**	3217.5529	1609.2801	3200.5263	1600.7668	3245.5478	1623.2775	3228.5213	1614.7643	**I**	1378.8053	689.9063	1361.7787	681.3930	**13**
**32**	3330.6370	1665.8221	3313.6104	1657.3088	3358.6319	1679.8196	3341.6053	1671.3063	**I**	1265.7212	633.3642	1248.6947	624.8510	**12**
**33**	3486.7381	1743.8727	3469.7115	1735.3594	3514.7330	1757.8701	3497.7064	1749.3569	**R**	1152.6371	576.8222	1135.6106	568.3089	**11**
**34**	3583.7908	1792.3991	3566.7643	1783.8858	3611.7857	1806.3965	3594.7592	1797.8832	**P**	996.5360	498.7717	979.5095	490.2584	**10**
**35**	3654.8279	1827.9176	3637.8014	1819.4043	3682.8229	1841.9151	3665.7963	1833.4018	**A**	899.4833	450.2453	882.4567	441.7320	**9**
**36**	3755.8756	1878.4415	3738.8491	1869.9282	3783.8705	1892.4389	3766.8440	1883.9256	**T**	828.4462	414.7267	811.4196	406.2134	**8**
**37**	3868.9597	1934.9835	3851.9331	1926.4702	3896.9546	1948.9809	3879.9281	1940.4677	**L**	727.3985	364.2029	710.3719	355.6896	**7**
**38**	3939.9968	1970.5020	3922.9703	1961.9888	3967.9917	1984.4995	3950.9652	1975.9862	**A**	614.3144	307.6608	597.2879	299.1476	**6**
**39**	4037.0496	2019.0284	4020.0230	2010.5151	4065.0445	2033.0259	4048.0179	2024.5126	**P**	543.2773	272.1423	526.2508	263.6290	**5**
**40**	4134.1023	2067.5548	4117.0758	2059.0415	4162.0972	2081.5523	4145.0707	2073.0390	**P**	446.2245	223.6159	429.1980	215.1026	**4**
**41**	4221.1344	2111.0708	4204.1078	2102.5575	4249.1293	2125.0683	4232.1027	2116.5550	**S**	349.1718	175.0895	332.1452	166.5763	**3**
**42**	4336.1613	2168.5843	4319.1348	2160.0710	4364.1562	2182.5817	4347.1297	2174.0685	**N**	262.1397	131.5735	245.1132	123.0602	**2**
**43**									**K**	147.1128	74.0600	130.0863	65.5468	**1**

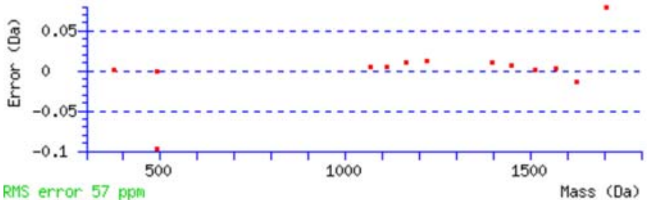

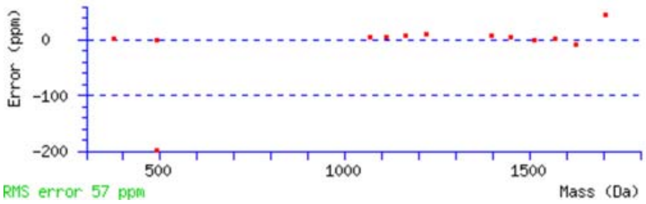

NCBI **BLAST** search of SNMDIDGINTGTIDKTPEEITSGTSGTTRPIIRPATLAPPSNK
(Parameters: blastp, nr protein database, expect=20000, no filter, PAM30)
Other BLAST web gateways

All matches to this query

Score	Mr(calc):	Delta	Sequence
27.4	4509.2544	-0.0035	SNMDIDGINTGTIDKTPEEITSGTSGTTRPIIRPATLAPPSNK
16.7	4507.2557	1.9952	TGDPDVREAKILAESTAMDTNAATVALIISNPNSIFQLILR
13.0	4508.2460	1.0049	MVTDFPYSVVGAVATVTQAIMWSIVIHTRNPDPLMK
13.0	4509.2300	0.0209	MVTDFPYSVVGAVATVTQAIMWSIVIHTRNPDPLMK
11.2	4509.1654	0.0855	TSSELMVKVKNVISAVNAAQLLINNNASLVIVQETK
11.2	4509.1654	0.0855	TSSELMVKVKNVISAVNAAQLLINNNASLVIVQETK
11.2	4509.1654	0.0855	TSSELMVKVKNVISAVNAAQLLINNNASLVIVQETK
11.2	4509.1654	0.0855	TSSELMVKVKNVISAVNAAQLLINNNASLVIVQETK
10.9	4508.2460	1.0049	MVTDFPYSVVGAVATVTQAIMWSIVIHTRNPDPLMK
10.9	4509.2300	0.0209	MVTDFPYSVVGAVATVTQAIMWSIVIHTRNPDPLMK

Mascot: http://www.matrixscience.com/

Mascot Search Results

Peptide View

MS/MS Fragmentation of **MSLFGDTIAYLLSLTEDGEGK**
Found in **M1**, B/Brisbane/60/2008

Match to Query 4204: 2275.087010 from(1138.550781,2+) intensity(66205.9000)
Title: B111206_011.06917.06917.2
Data file B111206_011.mgf.mascot

Click mouse within plot area to zoom in by factor of two about that point
Or, Plot from 300 to 2000 Da Full range
Label all possible matches ○ Label matches used for scoring ◉

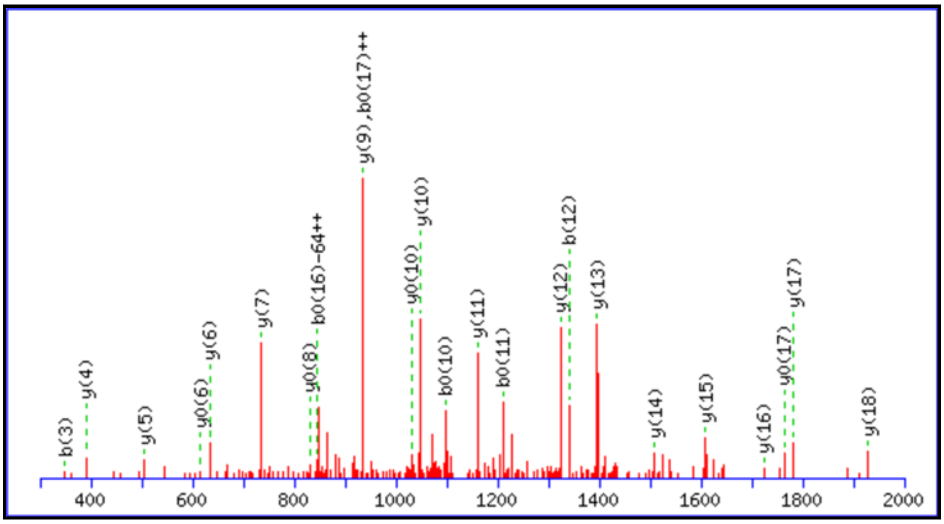


```
Monoisotopic mass of neutral peptide Mr(calc): 2275.0930
Fixed modifications: Carbamidomethyl (C) (apply to specified residues or termini only)
Variable modifications:
M1     : Oxidation (M), with neutral losses 0.0000(shown in table), 63.9983
Ions Score: 115  Expect: 3.3e-10
Matches : 24/272 fragment ions using 32 most intense peaks   (help)
```

#	b	b^{++}	b^{0}	b^{0++}	Seq.	y	y^{++}	y*	y^{*++}	y^{0}	y^{0++}	#
1	148.0427	74.5250			M							21
2	235.0747	118.0410	217.0641	109.0357	S	2129.0649	1065.0361	2112.0383	1056.5228	2111.0543	1056.0308	20
3	**348.1588**	174.5830	330.1482	165.5777	L	2042.0328	1021.5201	2025.0063	1013.0068	2024.0223	1012.5148	19
4	495.2272	248.1172	477.2166	239.1119	F	***1928.9488***	964.9780	1911.9222	956.4647	1910.9382	955.9727	18
5	552.2486	276.6280	534.2381	267.6227	G	***1781.8804***	891.4438	1764.8538	882.9305	1763.8698	882.4385	17
6	667.2756	334.1414	649.2650	325.1361	D	***1724.8589***	862.9331	1707.8323	854.4198	1706.8483	853.9278	16
7	768.3233	384.6653	750.3127	375.6600	T	***1609.8319***	805.4196	1592.8054	796.9063	1591.8214	796.4143	15
8	881.4073	441.2073	863.3968	432.2020	I	***1508.7843***	754.8958	1491.7577	746.3825	1490.7737	745.8905	14
9	952.4444	476.7259	934.4339	467.7206	A	***1395.7002***	698.3537	1378.6737	689.8405	1377.6896	689.3485	13
10	1115.5078	558.2575	1097.4972	549.2522	Y	***1324.6631***	662.8352	1307.6365	654.3219	1306.6525	653.8299	12
11	1228.5918	614.7996	1210.5813	605.7943	L	***1161.5998***	581.3035	1144.5732	572.7902	1143.5892	572.2982	11
12	**1341.6759**	671.3416	1323.6653	662.3363	L	***1048.5157***	524.7615	1031.4891	516.2482	1030.5051	515.7562	10
13	1428.7079	714.8576	1410.6974	705.8523	S	***935.4316***	468.2195	918.4051	459.7062	917.4211	459.2142	9
14	1541.7920	771.3996	1523.7814	762.3944	L	848.3996	424.7034	831.3731	416.1902	830.3890	415.6982	8
15	1642.8397	821.9235	1624.8291	812.9182	T	***735.3155***	368.1614	718.2890	359.6481	717.3050	359.1561	7
16	1771.8823	886.4448	1753.8717	877.4395	E	***634.2679***	317.6376	617.2413	309.1243	616.2573	308.6323	6
17	1886.9092	943.9582	1868.8986	934.9530	D	***505.2253***	253.1163	488.1987	244.6030	487.2147	244.1110	5
18	1943.9307	972.4690	1925.9201	963.4637	G	***390.1983***	195.6028	373.1718	187.0895	372.1878	186.5975	4

**19**	2072.9733	1036.9903	2054.9627	1027.9850	**E**	333.1769	167.0921	316.1503	158.5788	315.1663	158.0868	**3**
**20**	2129.9947	1065.5010	2111.9842	1056.4957	**G**	204.1343	102.5708	187.1077	94.0575			**2**
**21**					**K**	147.1128	74.0600	130.0863	65.5468			**1**

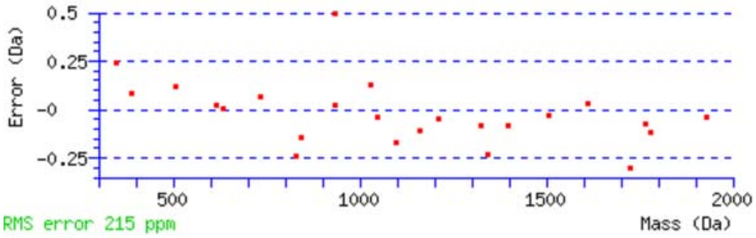

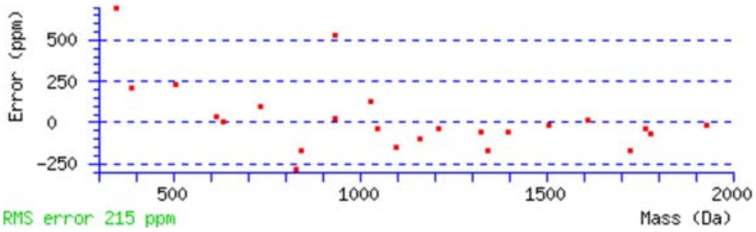

NCBI **BLAST** search of MSLFGDTIAYLLSLTEDGEGK
(Parameters: blastp, nr protein database, expect=20000, no filter, PAM30)
Other BLAST web gateways

All matches to this query

Score	Mr(calc):	Delta	Sequence
115.0	2275.0930	-0.0060	MSLFGDTIAYLLSLTEDGEGK
13.0	2275.0452	0.0418	SLSSSPTQSELSHSLTQDQDK
4.7	2275.0807	0.0063	MVPSTNGIANLESGVPQLIPR
2.6	2274.0984	0.9886	KCSEVEDLMGKIASLQAHNK
2.4	2275.1153	-0.0283	LNLTIVLTSLELWTEKNK
2.3	2275.1002	-0.0132	GKTGGSQDSQPSPLALLAATCSK
1.6	2274.0528	1.0342	WFCLTSREFTYHKQQDK
1.3	2274.0636	1.0234	MSGTLAKIAEIEAEMARTQK
1.2	2274.0636	1.0234	MSGTLAKIAEIEAEMARTQK
0.9	2273.0382	2.0488	LSIQDNNVDLILATPPFSR

Mascot: http://www.matrixscience.com/

MATRIX SCIENCE Mascot Search Results

Peptide View

MS/MS Fragmentation of **MSLFGDTIAYLLSLTEDGEGKAELAEK**
Found in **M1**, B/Brisbane/60/2008

Match to Query 8958: 3022.448172 from(1008.490000,3+) intensity(445011.2500)
Title: D120713_002.17511.17511.3
Data file D120713_002.mgf.mascot

Click mouse within plot area to zoom in by factor of two about that point
Or, Plot from 200 to 2000 Da Full range
Label all possible matches ○ Label matches used for scoring ◉
View spectrum in original style ◉ View spectrum with detailed annotation ○

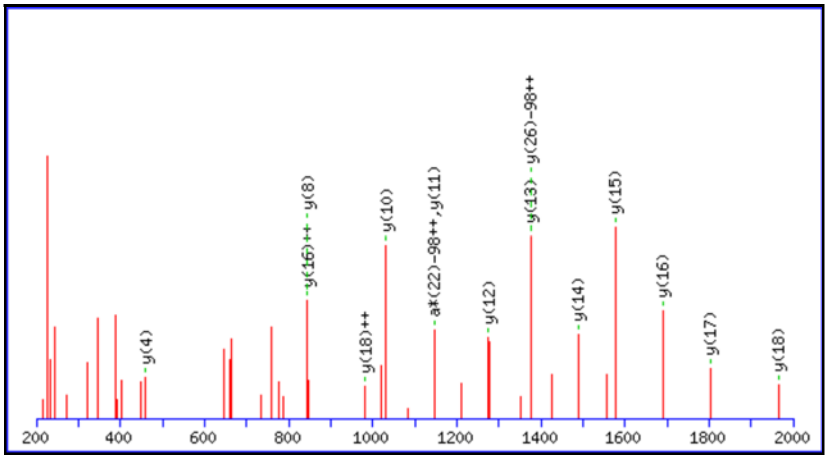

Monoisotopic mass of neutral peptide Mr(calc): 3022.4133
Fixed modifications: Carbamidomethyl (C) (apply to specified residues or termini only)
Variable modifications:
N-term : Acetyl (N-term)
T7 : Phospho (ST), with neutral losses 97.9769(shown in table), 0.0000
Ions Score: 82 **Expect:** 7.5e-06
Matches : 15/360 fragment ions using 16 most intense peaks (help)

#	a	a^{++}	a*	a^{*++}	b	b^{++}	b*	b^{*++}	Seq.	y	y^{++}	y*	y^{*++}	#
1	146.0634	73.5353			174.0583	87.5328			M					27
2	233.0954	117.0514			261.0904	131.0488			S	2752.3927	1376.7000	2735.3662	1368.1867	26
3	346.1795	173.5934			374.1744	187.5908			L	2665.3607	1333.1840	2648.3342	1324.6707	25
4	493.2479	247.1276			521.2428	261.1251			F	2552.2766	1276.6420	2535.2501	1268.1287	24
5	550.2694	275.6383			578.2643	289.6358			G	2405.2082	1203.1078	2388.1817	1194.5945	23
6	665.2963	333.1518			693.2912	347.1493			D	2348.1868	1174.5970	2331.1602	1166.0837	22
7	748.3334	374.6704			776.3284	388.6678			T	2233.1598	1117.0835	2216.1333	1108.5703	21
8	861.4175	431.2124			889.4124	445.2098			I	2150.1227	1075.5650	2133.0962	1067.0517	20
9	932.4546	466.7309			960.4495	480.7284			A	2037.0386	1019.0230	2020.0121	1010.5097	19
10	1095.5179	548.2626			1123.5129	562.2601			Y	***1966.0015***	983.5044	1948.9750	974.9911	18
11	1208.6020	604.8046			1236.5969	618.8021			L	***1802.9382***	901.9727	1785.9117	893.4595	17
12	1321.6861	661.3467			1349.6810	675.3441			L	***1689.8541***	845.4307	1672.8276	836.9174	16
13	1408.7181	704.8627			1436.7130	718.8601			S	***1576.7701***	788.8887	1559.7435	780.3754	15
14	1521.8022	761.4047			1549.7971	775.4022			L	***1489.7380***	745.3727	1472.7115	736.8594	14
15	1622.8498	811.9286			1650.8448	825.9260			T	***1376.6540***	688.8306	1359.6274	680.3174	13
16	1751.8924	876.4499			1779.8874	890.4473			E	***1275.6063***	638.3068	1258.5798	629.7935	12
17	1866.9194	933.9633			1894.9143	947.9608			D	***1146.5637***	573.7855	1129.5372	565.2722	11
18	1923.9408	962.4741			1951.9358	976.4715			G	***1031.5368***	516.2720	1014.5102	507.7587	10
19	2052.9834	1026.9954			2080.9784	1040.9928			E	974.5153	487.7613	957.4888	479.2480	9
20	2110.0049	1055.5061			2137.9998	1069.5035			G	***845.4727***	423.2400	828.4462	414.7267	8

21	2238.0999	1119.5536	2221.0733	1111.0403	2266.0948	1133.5510	2249.0682	1125.0378	K	788.4512	394.7293	771.4247	386.2160	7
22	2309.1370	1155.0721	2292.1104	1146.5589	2337.1319	1169.0696	2320.1053	1160.5563	A	660.3563	330.6818	643.3297	322.1685	6
23	2438.1796	1219.5934	2421.1530	1211.0801	2466.1745	1233.5909	2449.1479	1225.0776	E	589.3192	295.1632	572.2926	286.6499	5
24	2551.2636	1276.1355	2534.2371	1267.6222	2579.2585	1290.1329	2562.2320	1281.6196	L	***460.2766***	230.6419	443.2500	222.1287	4
25	2622.3007	1311.6540	2605.2742	1303.1407	2650.2957	1325.6515	2633.2691	1317.1382	A	347.1925	174.0999	330.1660	165.5866	3
26	2751.3433	1376.1753	2734.3168	1367.6620	2779.3383	1390.1728	2762.3117	1381.6595	E	276.1554	138.5813	259.1288	130.0681	2
27									K	147.1128	74.0600	130.0863	65.5468	1

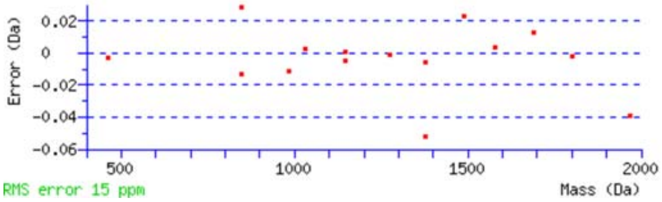

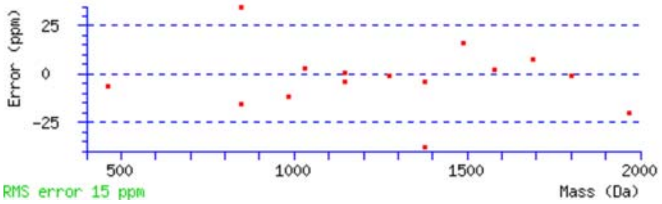

NCBI **BLAST** search of MSLFGDTIAYLLSLTEDGEGKAELAEK
(Parameters: blastp, nr protein database, expect=20000, no filter, PAM30)
Other BLAST web gateways

All matches to this query

Score	Mr(calc):	Delta	Sequence
82.0	3022.4133	0.0348	MSLFGDTIAYLLSLTEDGEGKAELAEK
82.0	3022.4133	0.0348	MSLFGDTIAYLLSLTEDGEGKAELAEK
63.6	3022.4133	0.0348	MSLFGDTIAYLLSLTEDGEGKAELAEK
27.6	3022.4133	0.0348	MSLFGDTIAYLLSLTEDGEGKAELAEK
26.1	3020.4261	2.0221	TTISGQMTLTDETLSAVSKSSKSAVK
26.1	3020.4261	2.0221	TTISGQMTLTDETLSAVSKSSKSAVK
26.1	3020.4261	2.0221	TTISGQMTLTDETLSAVSKSSKSAVK
26.1	3020.4261	2.0221	TTISGQMTLTDETLSAVSKSSKSAVK
26.1	3020.4261	2.0221	TTISGQMTLTDETLSAVSKSSKSAVK
26.1	3020.4261	2.0221	TTISGQMTLTDETLSAVSKSSKSAVK

Mascot: http://www.matrixscience.com/

{MATRIX SCIENCE} Mascot Search Results

Peptide View

MS/MS Fragmentation of **MSLFGDTIAYLLSLTEDGEGKAELAEK**
Found in **M1**, B/Brisbane/60/2008

Match to Query 5205: 2916.432273 from(973.151367,3+) intensity(87118.5000)
Title: B111206_011.06633.06633.3
Data file B111206_011.mgf.mascot

Click mouse within plot area to zoom in by factor of two about that point
Or, Plot from 200 to 1900 Da Full range
Label all possible matches ○ Label matches used for scoring ◉

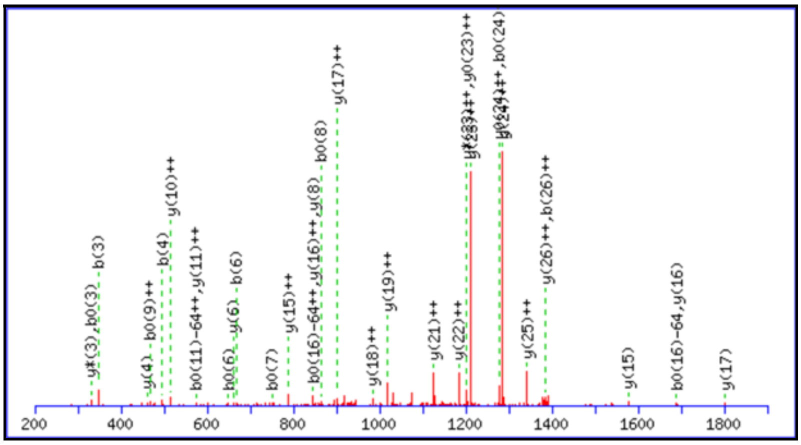

Monoisotopic mass of neutral peptide Mr(calc): 2916.4314
Fixed modifications: Carbamidomethyl (C) (apply to specified residues or termini only)
Variable modifications:
M1 : Oxidation (M), with neutral losses 0.0000(shown in table), 63.9983
Ions Score: 89 **Expect:** 1e-07
Matches : 38/382 fragment ions using 42 most intense peaks (help)

#	b	b^{++}	b*	b^{*++}	b^0	b^{0++}	Seq.	y	y^{++}	y*	y^{*++}	y^0	y^{0++}	#
1	148.0427	74.5250					M							27
2	235.0747	118.0410			217.0641	109.0357	S	2770.4033	1385.7053	2753.3768	1377.1920	2752.3927	1376.7000	26
3	348.1588	174.5830			330.1482	165.5777	L	2683.3713	1342.1893	2666.3447	1333.6760	2665.3607	1333.1840	25
4	495.2272	248.1172			477.2166	239.1119	F	2570.2872	1285.6472	2553.2607	1277.1340	2552.2766	1276.6420	24
5	552.2486	276.6280			534.2381	267.6227	G	2423.2188	1212.1130	2406.1922	1203.5998	2405.2082	1203.1078	23
6	667.2756	334.1414			649.2650	325.1361	D	2366.1973	1183.6023	2349.1708	1175.0890	2348.1868	1174.5970	22
7	768.3233	384.6653			750.3127	375.6600	T	2251.1704	1126.0888	2234.1438	1117.5756	2233.1598	1117.0835	21
8	881.4073	441.2073			863.3968	432.2020	I	2150.1227	1075.5650	2133.0962	1067.0517	2132.1121	1066.5597	20
9	952.4444	476.7259			934.4339	467.7206	A	2037.0386	1019.0230	2020.0121	1010.5097	2019.0281	1010.0177	19
10	1115.5078	558.2575			1097.4972	549.2522	Y	1966.0015	983.5044	1948.9750	974.9911	1947.9910	974.4991	18
11	1228.5918	614.7996			1210.5813	605.7943	L	1802.9382	901.9727	1785.9117	893.4595	1784.9276	892.9675	17
12	1341.6759	671.3416			1323.6653	662.3363	L	1689.8541	845.4307	1672.8276	836.9174	1671.8436	836.4254	16
13	1428.7079	714.8576			1410.6974	705.8523	S	1576.7701	788.8887	1559.7435	780.3754	1558.7595	779.8834	15
14	1541.7920	771.3996			1523.7814	762.3944	L	1489.7380	745.3727	1472.7115	736.8594	1471.7275	736.3674	14
15	1642.8397	821.9235			1624.8291	812.9182	T	1376.6540	688.8306	1359.6274	680.3174	1358.6434	679.8253	13
16	1771.8823	886.4448			1753.8717	877.4395	E	1275.6063	638.3068	1258.5798	629.7935	1257.5957	629.3015	12
17	1886.9092	943.9582			1868.8986	934.9530	D	1146.5637	573.7855	1129.5372	565.2722	1128.5531	564.7802	11
18	1943.9307	972.4690			1925.9201	963.4637	G	1031.5368	516.2720	1014.5102	507.7587	1013.5262	507.2667	10
19	2072.9733	1036.9903			2054.9627	1027.9850	E	974.5153	487.7613	957.4888	479.2480	956.5047	478.7560	9
20	2129.9947	1065.5010			2111.9842	1056.4957	G	845.4727	423.2400	828.4462	414.7267	827.4621	414.2347	8
21	2258.0897	1129.5485	2241.0631	1121.0352	2240.0791	1120.5432	K	788.4512	394.7293	771.4247	386.2160	770.4407	385.7240	7
22	2329.1268	1165.0670	2312.1003	1156.5538	2311.1162	1156.0618	A	660.3563	330.6818	643.3297	322.1685	642.3457	321.6765	6
23	2458.1694	1229.5883	2441.1429	1221.0751	2440.1588	1220.5831	E	589.3192	295.1632	572.2926	286.6499	571.3086	286.1579	5
24	2571.2535	1286.1304	2554.2269	1277.6171	2553.2429	1277.1251	L	460.2766	230.6419	443.2500	222.1287	442.2660	221.6366	4
25	2642.2906	1321.6489	2625.2640	1313.1357	2624.2800	1312.6436	A	347.1925	174.0999	330.1660	165.5866	329.1819	165.0946	3
26	2771.3332	1386.1702	2754.3066	1377.6569	2753.3226	1377.1649	E	276.1554	138.5813	259.1288	130.0681	258.1448	129.5761	2
27							K	147.1128	74.0600	130.0863	65.5468			1

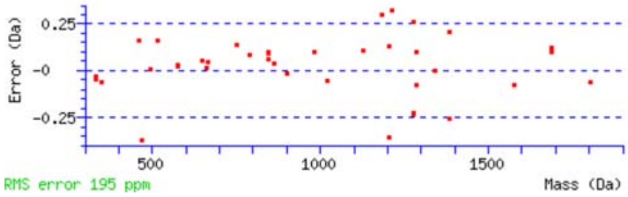

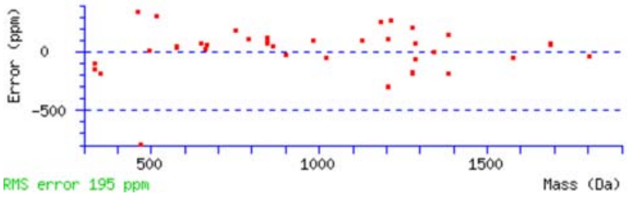

NCBI **BLAST** search of MSLFGDTIAYLLSLTEDGEGKAELAEK
(Parameters: blastp, nr protein database, expect=20000, no filter, PAM30)
Other BLAST web gateways

All matches to this query

Score	Mr(calc):	Delta	Sequence
88.7	2916.4314	0.0009	MSLFGDTIAYLLSLTEDGEGKAELAEK
13.3	2916.4684	-0.0361	TNLLQVCERIPTISTQLKILSTVK
12.5	2916.3914	0.0408	QQIMEKQYGFNEILKHINELSMK
11.5	2915.4844	0.9479	TNLLQVCERIPTISTQLKILSTVK
11.2	2914.4068	2.0254	ELTGDMQLSKGEILRTQMLVTTPEK
10.8	2915.4844	0.9479	TNLLQVCERIPTISTQLKILSTVK
10.8	2916.4684	-0.0361	TNLLQVCERIPTISTQLKILSTVK
10.2	2916.3996	0.0326	NAISLPAIGGSQALMLPLLSPVTFNSK
9.5	2916.4684	-0.0361	TNLLQVCERIPTISTQLKILSTVK
8.2	2914.4598	1.9724	ACIFGTTSLISHCLRKGENIAFVLK

Mascot: http://www.matrixscience.com/

{MATRIX SCIENCE} Mascot Search Results

Peptide View

MS/MS Fragmentation of **SLFGDTIAYLLSLTEDGEGK**
Found in **M1**, B/Brisbane/60/2008

Match to Query 3937: 2128.061132 from(1065.037842,2+) intensity(105798.0000)
Title: B111206_011.06960.06960.2
Data file B111206_011.mgf.mascot

Click mouse within plot area to zoom in by factor of two about that point
Or, Plot from 300 to 2000 Da Full range
Label all possible matches ○ Label matches used for scoring ◉

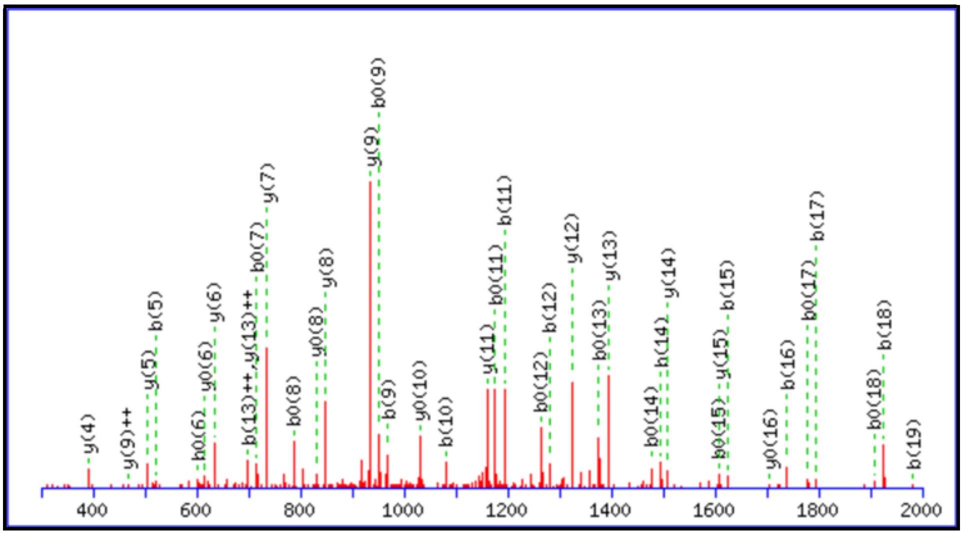

Monoisotopic mass of neutral peptide Mr(calc): 2128.0576
Fixed modifications: Carbamidomethyl (C) (apply to specified residues or termini only)
Ions Score: 95 **Expect:** 9.8e-09
Matches : 40/186 fragment ions using 51 most intense peaks (help)

#	b	b^{++}	b^{0}	b^{0++}	Seq.	y	y^{++}	y*	y^{*++}	y^{0}	y^{0++}	#
1	88.0393	44.5233	70.0287	35.5180	S							20
2	201.1234	101.0653	183.1128	92.0600	L	2042.0328	1021.5201	2025.0063	1013.0068	2024.0223	1012.5148	19
3	348.1918	174.5995	330.1812	165.5942	F	1928.9488	964.9780	1911.9222	956.4647	1910.9382	955.9727	18
4	405.2132	203.1103	387.2027	194.1050	G	1781.8804	891.4438	1764.8538	882.9305	1763.8698	882.4385	17
5	***520.2402***	260.6237	502.2296	251.6184	D	1724.8589	862.9331	1707.8323	854.4198	1706.8483	853.9278	16
6	621.2879	311.1476	603.2773	302.1423	T	***1609.8319***	805.4196	1592.8054	796.9063	1591.8214	796.4143	15
7	734.3719	367.6896	716.3614	358.6843	I	***1508.7843***	754.8958	1491.7577	746.3825	1490.7737	745.8905	14
8	805.4090	403.2082	787.3985	394.2029	A	***1395.7002***	698.3537	1378.6737	689.8405	1377.6896	689.3485	13
9	***968.4724***	484.7398	950.4618	475.7345	Y	***1324.6631***	662.8352	1307.6365	654.3219	1306.6525	653.8299	12
10	***1081.5564***	541.2819	1063.5459	532.2766	L	***1161.5998***	581.3035	1144.5732	572.7902	1143.5892	572.2982	11
11	***1194.6405***	597.8239	1176.6299	588.8186	L	1048.5157	524.7615	1031.4891	516.2482	1030.5051	515.7562	10
12	***1281.6725***	641.3399	1263.6620	632.3346	S	***935.4316***	468.2195	918.4051	459.7062	917.4211	459.2142	9
13	1394.7566	697.8819	1376.7460	688.8767	L	***848.3996***	424.7034	831.3731	416.1902	830.3890	415.6982	8
14	***1495.8043***	748.4058	1477.7937	739.4005	T	***735.3155***	368.1614	718.2890	359.6481	717.3050	359.1561	7
15	***1624.8469***	812.9271	1606.8363	803.9218	E	***634.2679***	317.6376	617.2413	309.1243	616.2573	308.6323	6
16	***1739.8738***	870.4405	1721.8632	861.4353	D	***505.2253***	253.1163	488.1987	244.6030	487.2147	244.1110	5
17	***1796.8953***	898.9513	1778.8847	889.9460	G	***390.1983***	195.6028	373.1718	187.0895	372.1878	186.5975	4
18	***1925.9379***	963.4726	1907.9273	954.4673	E	333.1769	167.0921	316.1503	158.5788	315.1663	158.0868	3

**19**	***1982.9593***	991.9833	1964.9488	982.9780	**G**	204.1343	102.5708	187.1077	94.0575			**2**
**20**					**K**	147.1128	74.0600	130.0863	65.5468			**1**

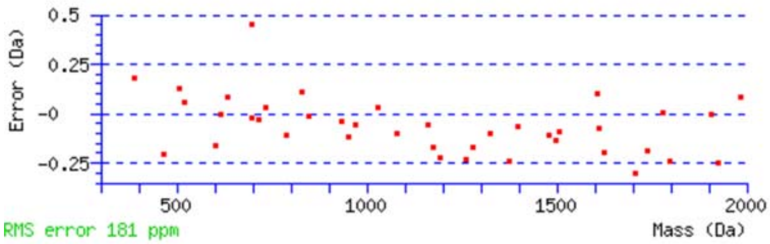

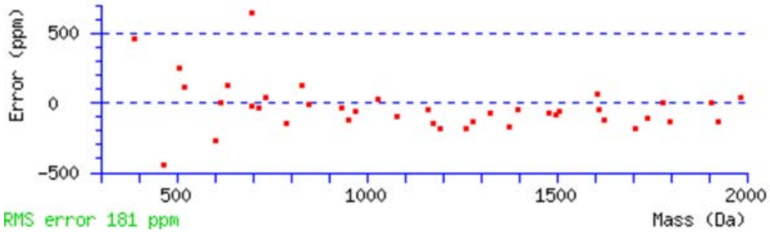

NCBI **BLAST** search of SLFGDTIAYLLSLTEDGEGK
(Parameters: blastp, nr protein database, expect=20000, no filter, PAM30)
Other BLAST web gateways

All matches to this query

Score	Mr(calc):	Delta	Sequence
95.4	2128.0576	0.0036	SLFGDTIAYLLSLTEDGEGK
9.3	2126.0565	2.0046	ISKLFEQMSQTVTDEDKK
9.0	2128.0396	0.0215	SNSDSEIQADLQHSSILRK
8.7	2128.0245	0.0366	ISKLFEQMSQTVTDEDKK
6.2	2128.0806	-0.0195	KVGTSTNALESLVLRRPK
5.8	2128.0806	-0.0195	KVGTSTNALESLVLRRPK
5.8	2128.0806	-0.0195	KVGTSTNALESLVLRRPK
4.8	2128.0670	-0.0058	AFNEMRVSLYLNLSRCR
4.5	2127.0405	1.0206	ISKLFEQMSQTVTDEDKK
4.4	2127.0556	1.0055	SNSDSEIQADLQHSSILRK

Mascot: http://www.matrixscience.com/

Mascot Search Results

Peptide View

MS/MS Fragmentation of **SLFGDTIAYLLSLTEDGEGK**
Found in **M1**, B/Brisbane/60/2008

Match to Query 4009: 2170.074072 from(1086.044312,2+) intensity(95160000.0000)
Title: B111206_011.07475.07475.2
Data file B111206_011.mgf.mascot

Click mouse within plot area to zoom in by factor of two about that point
Or, Plot from 300 to 2000 Da Full range
Label all possible matches ○ Label matches used for scoring ◉

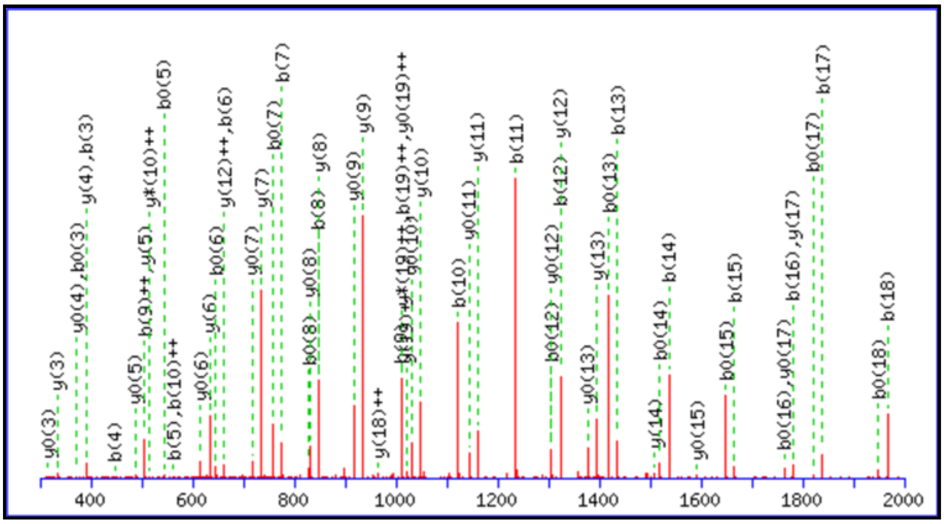

Monoisotopic mass of neutral peptide Mr(calc): 2170.0681
Fixed modifications: Carbamidomethyl (C) (apply to specified residues or termini only)
Variable modifications:
N-term : Acetyl (Protein N-term)
Ions Score: 142 **Expect:** 2.8e-12
Matches : 63/186 fragment ions using 68 most intense peaks (help)

#	b	b^{++}	b^0	b^{0++}	Seq.	y	y^{++}	y*	y^{*++}	y^0	y^{0++}	#
1	130.0499	65.5286	112.0393	56.5233	S							20
2	243.1339	122.0706	225.1234	113.0653	L	2042.0328	1021.5201	2025.0063	1013.0068	2024.0223	1012.5148	19
3	390.2023	195.6048	372.1918	186.5995	F	1928.9488	964.9780	1911.9222	956.4647	1910.9382	955.9727	18
4	447.2238	224.1155	429.2132	215.1103	G	1781.8804	891.4438	1764.8538	882.9305	1763.8698	882.4385	17
5	562.2508	281.6290	544.2402	272.6237	D	1724.8589	862.9331	1707.8323	854.4198	1706.8483	853.9278	16
6	663.2984	332.1529	645.2879	323.1476	T	1609.8319	805.4196	1592.8054	796.9063	1591.8214	796.4143	15
7	776.3825	388.6949	758.3719	379.6896	I	1508.7843	754.8958	1491.7577	746.3825	1490.7737	745.8905	14
8	847.4196	424.2134	829.4090	415.2082	A	1395.7002	698.3537	1378.6737	689.8405	1377.6896	689.3485	13
9	1010.4829	505.7451	992.4724	496.7398	Y	1324.6631	662.8352	1307.6365	654.3219	1306.6525	653.8299	12
10	1123.5670	562.2871	1105.5564	553.2819	L	1161.5998	581.3035	1144.5732	572.7902	1143.5892	572.2982	11
11	1236.6511	618.8292	1218.6405	609.8239	L	1048.5157	524.7615	1031.4891	516.2482	1030.5051	515.7562	10
12	1323.6831	662.3452	1305.6725	653.3399	S	935.4316	468.2195	918.4051	459.7062	917.4211	459.2142	9
13	1436.7672	718.8872	1418.7566	709.8819	L	848.3996	424.7034	831.3731	416.1902	830.3890	415.6982	8
14	1537.8148	769.4111	1519.8043	760.4058	T	735.3155	368.1614	718.2890	359.6481	717.3050	359.1561	7
15	1666.8574	833.9324	1648.8469	824.9271	E	634.2679	317.6376	617.2413	309.1243	616.2573	308.6323	6
16	1781.8844	891.4458	1763.8738	882.4405	D	505.2253	253.1163	488.1987	244.6030	487.2147	244.1110	5
17	1838.9058	919.9566	1820.8953	910.9513	G	390.1983	195.6028	373.1718	187.0895	372.1878	186.5975	4
18	1967.9484	984.4779	1949.9379	975.4726	E	333.1769	167.0921	316.1503	158.5788	315.1663	158.0868	3

**19**	2024.9699	**1012.9886**	2006.9593	1003.9833	**G**	204.1343	102.5708	187.1077	94.0575			**2**
**20**					**K**	147.1128	74.0600	130.0863	65.5468			**1**

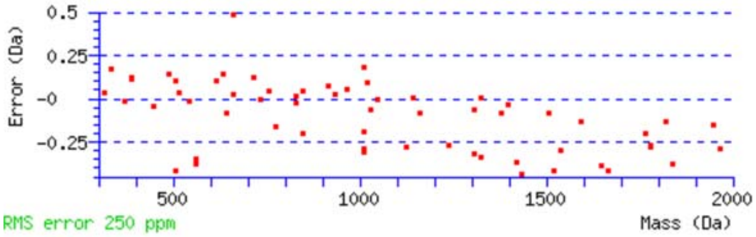

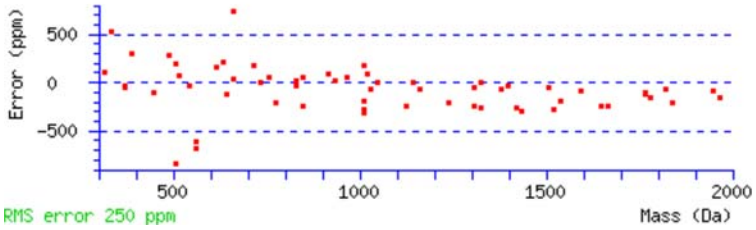

NCBI **BLAST** search of SLFGDTIAYLLSLTEDGEGK
(Parameters: blastp, nr protein database, expect=20000, no filter, PAM30)
Other BLAST web gateways

All matches to this query

Score	Mr(calc):	Delta	Sequence
141.6	2170.0681	0.0059	SLFGDTIAYLLSLTEDGEGK
19.1	2170.1166	-0.0425	AVGAMWAAEIPLLLQCLQGK
9.9	2170.0616	0.0125	NFXWDTVTSKIELNQKMK
6.3	2169.0293	1.0447	MAEVTAEGRMSTITTITDGK
6.0	2170.0438	0.0303	TLPLEIFQMLLSYLSVK
5.7	2168.0970	1.9771	GKMCVVVADISTQLGLYASR
4.6	2168.0724	2.0017	EFLPGEELHALHLVYNMR
4.3	2170.0679	0.0061	VNTWRLLLCEATATIMAK
4.2	2169.0445	1.0296	LLVLTQSSAYVVEMAKIK
4.0	2170.0438	0.0303	TLPLEIFQMLLSYLSVK

Mascot: http://www.matrixscience.com/

Mascot Search Results

Peptide View

MS/MS Fragmentation of **SLFGDTIAYLLSLTEDGEGKAELAEK**
Found in **M1**, B/Brisbane/60/2008

Match to Query 4364: 2811.422934 from(938.148254,3+) intensity(84330344.0000)
Title: B120210_017.07116.07116.3
Data file B120210_017.mgf.mascot

Click mouse within plot area to zoom in by factor of two about that point
Or, Plot from 300 to 2000 Da Full range
Label all possible matches ○ Label matches used for scoring ◉

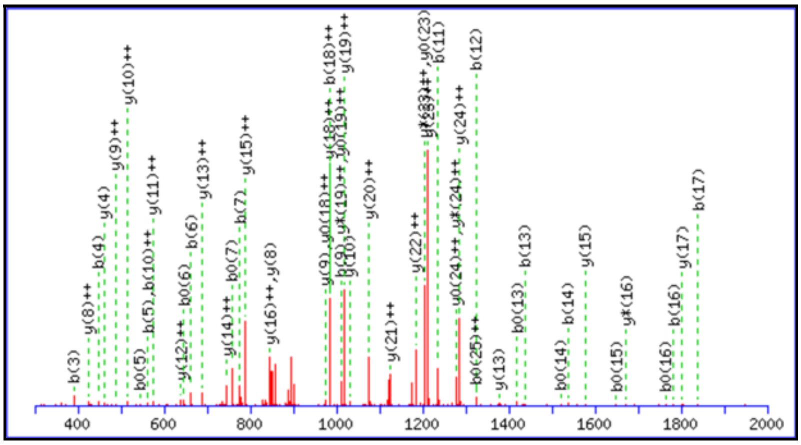

Monoisotopic mass of neutral peptide Mr(calc): 2811.4065
Fixed modifications: Carbamidomethyl (C) (apply to specified residues or termini only)
Variable modifications:
N-term : Acetyl (Protein N-term)
Ions Score: 124 **Expect:** 2.2e-11
Matches : 53/260 fragment ions using 63 most intense peaks (help)

#	b	b^{++}	b*	b^{*++}	b^0	b^{0++}	Seq.	y	y^{++}	y*	y^{*++}	y^0	y^{0++}	#
1	130.0499	65.5286			112.0393	56.5233	S							26
2	243.1339	122.0706			225.1234	113.0653	L	2683.3713	1342.1893	2666.3447	1333.6760	2665.3607	1333.1840	25
3	390.2023	195.6048			372.1918	186.5995	F	2570.2872	1285.6472	2553.2607	1277.1340	2552.2766	1276.6420	24
4	447.2238	224.1155			429.2132	215.1103	G	2423.2188	1212.1130	2406.1922	1203.5998	2405.2082	1203.1078	23
5	562.2508	281.6290			544.2402	272.6237	D	2366.1973	1183.6023	2349.1708	1175.0890	2348.1868	1174.5970	22
6	663.2984	332.1529			645.2879	323.1476	T	2251.1704	1126.0888	2234.1438	1117.5756	2233.1598	1117.0835	21
7	776.3825	388.6949			758.3719	379.6896	I	2150.1227	1075.5650	2133.0962	1067.0517	2132.1121	1066.5597	20
8	847.4196	424.2134			829.4090	415.2082	A	2037.0386	1019.0230	2020.0121	1010.5097	2019.0281	1010.0177	19
9	1010.4829	505.7451			992.4724	496.7398	Y	1966.0015	983.5044	1948.9750	974.9911	1947.9910	974.4991	18
10	1123.5670	562.2871			1105.5564	553.2819	L	1802.9382	901.9727	1785.9117	893.4595	1784.9276	892.9675	17
11	1236.6511	618.8292			1218.6405	609.8239	L	1689.8541	845.4307	1672.8276	836.9174	1671.8436	836.4254	16
12	1323.6831	662.3452			1305.6725	653.3399	S	1576.7701	788.8887	1559.7435	780.3754	1558.7595	779.8834	15
13	1436.7672	718.8872			1418.7566	709.8819	L	1489.7380	745.3727	1472.7115	736.8594	1471.7275	736.3674	14
14	1537.8148	769.4111			1519.8043	760.4058	T	1376.6540	688.8306	1359.6274	680.3174	1358.6434	679.8253	13
15	1666.8574	833.9324			1648.8469	824.9271	E	1275.6063	638.3068	1258.5798	629.7935	1257.5957	629.3015	12
16	1781.8844	891.4458			1763.8738	882.4405	D	1146.5637	573.7855	1129.5372	565.2722	1128.5531	564.7802	11
17	1838.9058	919.9566			1820.8953	910.9513	G	1031.5368	516.2720	1014.5102	507.7587	1013.5262	507.2667	10
18	1967.9484	984.4779			1949.9379	975.4726	E	974.5153	487.7613	957.4888	479.2480	956.5047	478.7560	9
19	2024.9699	1012.9886			2006.9593	1003.9833	G	845.4727	423.2400	828.4462	414.7267	827.4621	414.2347	8
20	2153.0649	1077.0361	2136.0383	1068.5228	2135.0543	1068.0308	K	788.4512	394.7293	771.4247	386.2160	770.4407	385.7240	7
21	2224.1020	1112.5546	2207.0754	1104.0413	2206.0914	1103.5493	A	660.3563	330.6818	643.3297	322.1685	642.3457	321.6765	6
22	2353.1446	1177.0759	2336.1180	1168.5626	2335.1340	1168.0706	E	589.3192	295.1632	572.2926	286.6499	571.3086	286.1579	5
23	2466.2286	1233.6180	2449.2021	1225.1047	2448.2181	1224.6127	L	460.2766	230.6419	443.2500	222.1287	442.2660	221.6366	4
24	2537.2657	1269.1365	2520.2392	1260.6232	2519.2552	1260.1312	A	347.1925	174.0999	330.1660	165.5866	329.1819	165.0946	3
25	2666.3083	1333.6578	2649.2818	1325.1445	2648.2978	1324.6525	E	276.1554	138.5813	259.1288	130.0681	258.1448	129.5761	2
26							K	147.1128	74.0600	130.0863	65.5468			1

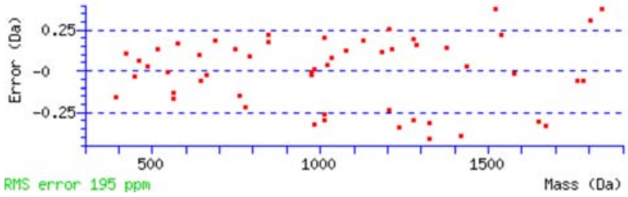

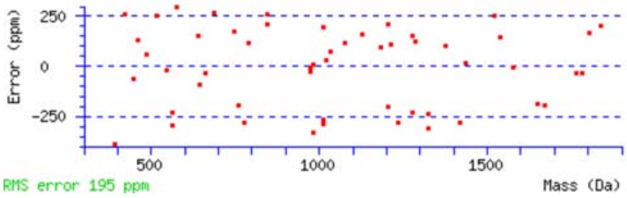

NCBI **BLAST** search of SLFGDTIAYLLSLTEDGEGKAELAEK
(Parameters: blastp, nr protein database, expect=20000, no filter, PAM30)
Other BLAST web gateways

All matches to this query

Score	Mr(calc):	Delta	Sequence
123.6	2811.4065	0.0164	SLFGDTIAYLLSLTEDGEGKAELAEK
9.3	2810.3877	1.0353	KLMTEMHVNSPVQVVINKLPTIK
6.9	2810.4136	1.0094	EDALQAATVANLRAALMSKNSLLSLK
6.0	2811.4559	-0.0329	KIVIATNIAETSITIDDVVFVIDGGK
5.5	2809.4248	1.9981	QLESEWSFLIVTDYLLQCPELVK
5.5	2810.4088	1.0141	QLESEWSFLIVTDYLLQCPELVK
5.0	2811.3717	0.0513	KLMTEMHVNSPVQVVINKLPTIK
5.0	2811.3717	0.0513	KLMTEMHVNSPVQVVINKLPTIK
4.4	2811.4766	-0.0537	ELRGLSLHTNNISSLPEGIFRSLQK
4.1	2811.4559	-0.0329	KIVIATNIAETSITIDDVVFVIDGGK

Mascot: http://www.matrixscience.com/

Mascot Search Results

Peptide View

MS/MS Fragmentation of **ANNNMTTTQIEWR**
Found in **NEP**, B/Brisbane/60/2008

Match to Query 3568: 1620.745448 from(811.380000,2+) intensity(4710422.5000)
Title: D120713_002.13017.13017.2
Data file D120713_002.mgf.mascot

Click mouse within plot area to zoom in by factor of two about that point
Or, Plot from 100 to 1300 Da Full range
Label all possible matches Label matches used for scoring
View spectrum in original style View spectrum with detailed annotation

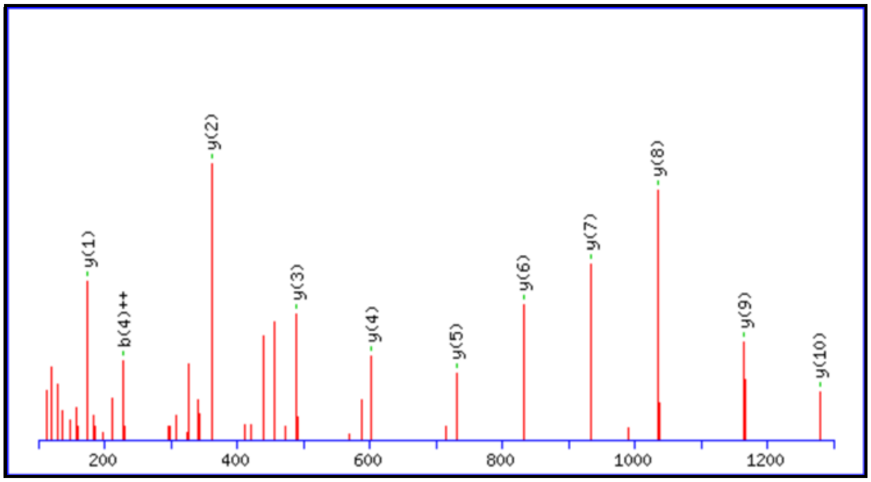

Monoisotopic mass of neutral peptide Mr(calc): 1620.7202
Fixed modifications: Carbamidomethyl (C) (apply to specified residues or termini only)
Variable modifications:
N-term : Acetyl (N-term)
N3 : Deamidated (NQ)
Ions Score: 110 **Expect:** 3.2e-08
Matches : 11/140 fragment ions using 11 most intense peaks (help)

#	a	a^{++}	a*	a^{*++}	b	b^{++}	b*	b^{*++}	Seq.	y	y^{++}	y*	y^{*++}	#
1	86.0600	43.5337			114.0550	57.5311			A					13
2	200.1030	100.5551	183.0764	92.0418	228.0979	114.5526	211.0713	106.0393	N	1508.6798	754.8435	1491.6533	746.3303	12
3	315.1299	158.0686	298.1034	149.5553	343.1248	172.0661	326.0983	163.5528	N	1394.6369	697.8221	1377.6103	689.3088	11
4	429.1728	215.0901	412.1463	206.5768	457.1678	**229.0875**	440.1412	220.5742	N	***1279.6099***	640.3086	1262.5834	631.7953	10
5	560.2133	280.6103	543.1868	272.0970	588.2082	294.6078	571.1817	286.0945	M	***1165.5670***	583.2871	1148.5405	574.7739	9
6	661.2610	331.1341	644.2345	322.6209	689.2559	345.1316	672.2294	336.6183	T	***1034.5265***	517.7669	1017.5000	509.2536	8
7	762.3087	381.6580	745.2821	373.1447	790.3036	395.6554	773.2770	387.1422	T	***933.4789***	467.2431	916.4523	458.7298	7
8	863.3564	432.1818	846.3298	423.6685	891.3513	446.1793	874.3247	437.6660	T	***832.4312***	416.7192	815.4046	408.2060	6
9	991.4149	496.2111	974.3884	487.6978	1019.4099	510.2086	1002.3833	501.6953	Q	***731.3835***	366.1954	714.3570	357.6821	5
10	1104.4990	552.7531	1087.4725	544.2399	1132.4939	566.7506	1115.4674	558.2373	I	***603.3249***	302.1661	586.2984	293.6528	4
11	1233.5416	617.2744	1216.5150	608.7612	1261.5365	631.2719	1244.5100	622.7586	E	***490.2409***	245.6241	473.2143	237.1108	3
12	1419.6209	710.3141	1402.5944	701.8008	1447.6158	724.3115	1430.5893	715.7983	W	***361.1983***	181.1028	344.1717	172.5895	2
13									R	***175.1190***	88.0631	158.0924	79.5498	1

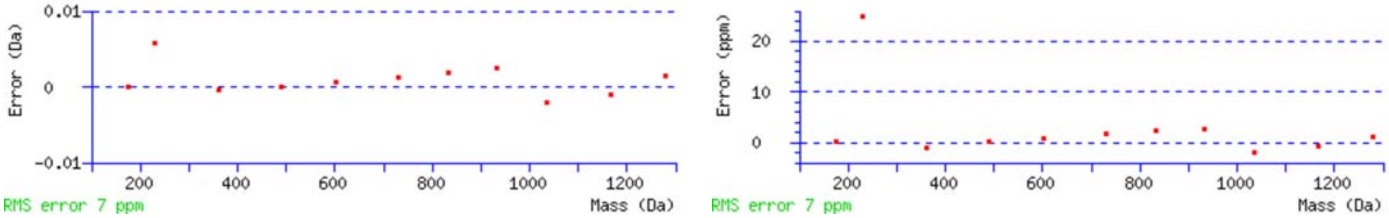

NCBI **BLAST** search of ANNNMTTTQIEWR
(Parameters: blastp, nr protein database, expect=20000, no filter, PAM30)
Other BLAST web gateways

All matches to this query

Score	Mr(calc):	Delta	Sequence
110.2	1620.7202	0.0253	ANNNMTTTQIEWR
110.2	1620.7202	0.0253	ANNNMTTTQIEWR
102.8	1619.7362	1.0093	ANNNMTTTQIEWR
89.4	1620.7202	0.0253	ANNNMTTTQIEWR
74.7	1620.7202	0.0253	ANNNMTTTQIEWR
21.0	1618.7603	1.9851	SLGINPPSQIDLSR
17.1	1619.7288	1.0166	ALNDNDFGQGRTGGR
16.5	1620.7575	-0.0120	MGGAMIGVFAGGMGAPR
16.1	1620.7436	0.0018	GFLSYLEEIQSVR
15.8	1620.7144	0.0310	EGNLGSPNASVLGER

Mascot: http://www.matrixscience.com/

MATRIX SCIENCE Mascot Search Results

Peptide View

MS/MS Fragmentation of **ANNNMTTTQIEVGPGATNATINFEAGILECYER**
Found in **NS1**, B/Brisbane/60/2008

Match to Query 10173: 3643.688172 from(1215.570000,3+) intensity(2188102.5000)
Title: D120713_002.15838.15838.3
Data file D120713_002.mgf.mascot

Click mouse within plot area to zoom in by factor of two about that point
Or, Plot from 200 to 2400 Da Full range
Label all possible matches ○ Label matches used for scoring ◉
View spectrum in original style ◉ View spectrum with detailed annotation ○

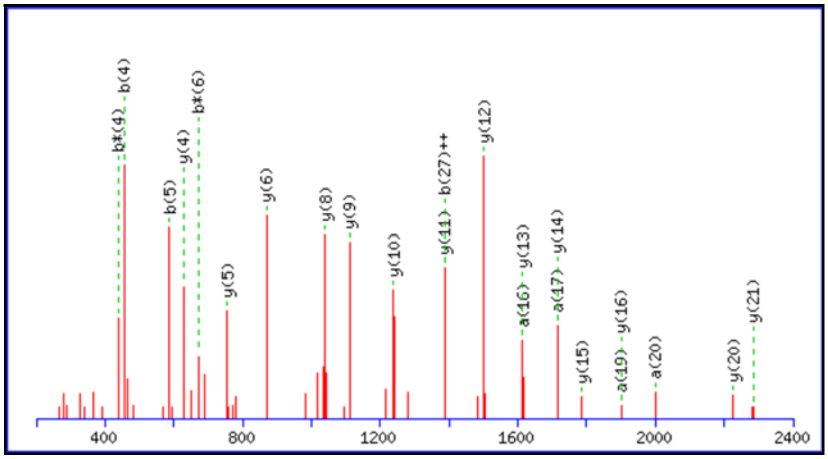


```
Monoisotopic mass of neutral peptide Mr(calc): 3641.6613
Fixed modifications: Carbamidomethyl (C) (apply to specified residues or termini only)
Variable modifications:
N-term : Acetyl (N-term)
N22    : Deamidated (NQ)
Ions Score: 87  Expect: 8.2e-07
Matches : 23/380 fragment ions using 36 most intense peaks   (help)
```

#	a	a++	a*	a*++	b	b++	b*	b*++	Seq.	y	y++	y*	y*++	#
1	86.0600	43.5337			114.0550	57.5311			A					33
2	200.1030	100.5551	183.0764	92.0418	228.0979	114.5526	211.0713	106.0393	N	3529.6210	1765.3141	3512.5944	1756.8009	32
3	314.1459	157.5766	297.1193	149.0633	342.1408	171.5740	325.1143	163.0608	N	3415.5781	1708.2927	3398.5515	1699.7794	31
4	428.1888	214.5980	411.1623	206.0848	**456.1837**	228.5955	439.1572	220.0822	N	3301.5351	1651.2712	3284.5086	1642.7579	30
5	559.2293	280.1183	542.2028	271.6050	**587.2242**	294.1157	570.1977	285.6025	M	3187.4922	1594.2497	3170.4657	1585.7365	29
6	660.2770	330.6421	643.2504	322.1289	688.2719	344.6396	671.2454	336.1263	T	3056.4517	1528.7295	3039.4252	1520.2162	28
7	761.3247	381.1660	744.2981	372.6527	789.3196	395.1634	772.2930	386.6502	T	2955.4040	1478.2057	2938.3775	1469.6924	27
8	862.3723	431.6898	845.3458	423.1765	890.3673	445.6873	873.3407	437.1740	T	2854.3564	1427.6818	2837.3298	1419.1685	26
9	990.4309	495.7191	973.4044	487.2058	1018.4258	509.7166	1001.3993	501.2033	Q	2753.3087	1377.1580	2736.2821	1368.6447	25
10	1103.5150	552.2611	1086.4884	543.7479	1131.5099	566.2586	1114.4834	557.7453	I	2625.2501	1313.1287	2608.2236	1304.6154	24
11	1232.5576	616.7824	1215.5310	608.2692	1260.5525	630.7799	1243.5259	622.2666	E	2512.1660	1256.5867	2495.1395	1248.0734	23
12	1331.6260	666.3166	1314.5994	657.8034	1359.6209	680.3141	1342.5944	671.8008	V	2383.1235	1192.0654	2366.0969	1183.5521	22
13	1388.6475	694.8274	1371.6209	686.3141	1416.6424	708.8248	1399.6158	700.3115	G	***2284.0550***	1142.5312	2267.0285	1134.0179	21
14	1485.7002	743.3537	1468.6737	734.8405	1513.6951	757.3512	1496.6686	748.8379	P	***2227.0336***	1114.0204	2210.0070	1105.5072	20
15	1542.7217	771.8645	1525.6951	763.3512	1570.7166	785.8619	1553.6901	777.3487	G	2129.9808	1065.4940	2112.9543	1056.9808	19
16	**1613.7588**	807.3830	1596.7322	798.8698	1641.7537	821.3805	1624.7272	812.8672	A	2072.9593	1036.9833	2055.9328	1028.4700	18
17	**1714.8065**	857.9069	1697.7799	849.3936	1742.8014	871.9043	1725.7748	863.3911	T	2001.9222	1001.4648	1984.8957	992.9515	17
18	1828.8494	914.9283	1811.8229	906.4151	1856.8443	928.9258	1839.8178	920.4125	N	***1900.8746***	950.9409	1883.8480	942.4276	16
19	**1899.8865**	950.4469	1882.8600	941.9336	1927.8814	964.4444	1910.8549	955.9311	A	***1786.8316***	893.9195	1769.8051	885.4062	15
20	**2000.9342**	1000.9707	1983.9076	992.4575	2028.9291	1014.9682	2011.9026	1006.4549	T	***1715.7945***	858.4009	1698.7680	849.8876	14

21	2114.0183	1057.5128	2096.9917	1048.9995	2142.0132	1071.5102	2124.9866	1062.9970	I	1614.7468	807.8771	1597.7203	799.3638	13
22	2229.0452	1115.0262	2212.0187	1106.5130	2257.0401	1129.0237	2240.0136	1120.5104	N	1501.6628	751.3350	1484.6362	742.8217	12
23	2376.1136	1188.5604	2359.0871	1180.0472	2404.1085	1202.5579	2387.0820	1194.0446	F	1386.6358	693.8216	1369.6093	685.3083	11
24	2505.1562	1253.0817	2488.1297	1244.5685	2533.1511	1267.0792	2516.1246	1258.5659	E	1239.5674	620.2873	1222.5409	611.7741	10
25	2576.1933	1288.6003	2559.1668	1280.0870	2604.1882	1302.5978	2587.1617	1294.0845	A	1110.5248	555.7660	1093.4983	547.2528	9
26	2633.2148	1317.1110	2616.1882	1308.5978	2661.2097	1331.1085	2644.1832	1322.5952	G	1039.4877	520.2475	1022.4612	511.7342	8
27	2746.2989	1373.6531	2729.2723	1365.1398	2774.2938	1387.6505	2757.2672	1379.1372	I	982.4662	491.7368	965.4397	483.2235	7
28	2859.3829	1430.1951	2842.3564	1421.6818	2887.3778	1444.1926	2870.3513	1435.6793	L	869.3822	435.1947	852.3556	426.6815	6
29	2988.4255	1494.7164	2971.3990	1486.2031	3016.4204	1508.7139	2999.3939	1500.2006	E	756.2981	378.6527	739.2716	370.1394	5
30	3148.4562	1574.7317	3131.4296	1566.2184	3176.4511	1588.7292	3159.4245	1580.2159	C	627.2555	314.1314	610.2290	305.6181	4
31	3311.5195	1656.2634	3294.4929	1647.7501	3339.5144	1670.2608	3322.4879	1661.7476	Y	467.2249	234.1161	450.1983	225.6028	3
32	3440.5621	1720.7847	3423.5355	1712.2714	3468.5570	1734.7821	3451.5304	1726.2689	E	304.1615	152.5844	287.1350	144.0711	2
33									R	175.1190	88.0631	158.0924	79.5498	1

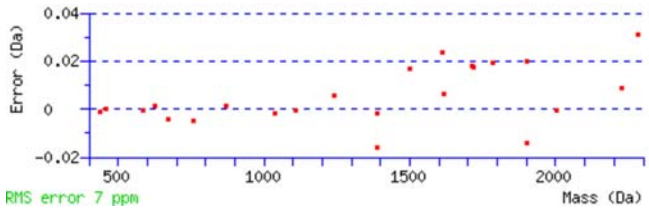

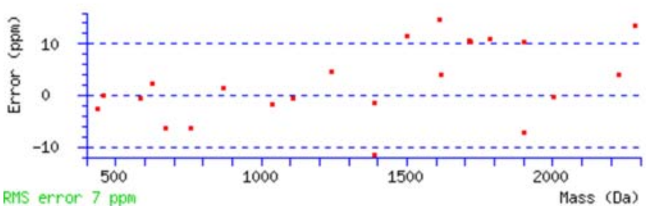

NCBI **BLAST** search of ANNNMTTTQIEVGPGATNATINFEAGILECYER
(Parameters: blastp, nr protein database, expect=20000, no filter, PAM30)
Other BLAST web gateways

All matches to this query

Score	Mr(calc):	Delta	Sequence
87.3	3641.6613	2.0268	ANNNMTTTQIEVGPGATNATINFEAGILECYER
84.8	3641.6613	2.0268	ANNNMTTTQIEVGPGATNATINFEAGILECYER
83.3	3641.6613	2.0268	ANNNMTTTQIEVGPGATNATINFEAGILECYER
83.3	3642.6454	1.0428	ANNNMTTTQIEVGPGATNATINFEAGILECYER
79.6	3642.6454	1.0428	ANNNMTTTQIEVGPGATNATINFEAGILECYER
74.8	3642.6454	1.0428	ANNNMTTTQIEVGPGATNATINFEAGILECYER
74.8	3643.6294	0.0588	ANNNMTTTQIEVGPGATNATINFEAGILECYER
74.8	3642.6454	1.0428	ANNNMTTTQIEVGPGATNATINFEAGILECYER
74.8	3642.6454	1.0428	ANNNMTTTQIEVGPGATNATINFEAGILECYER
73.4	3641.6613	2.0268	ANNNMTTTQIEVGPGATNATINFEAGILECYER

Mascot: http://www.matrixscience.com/

WSN PB2 740-752 unmodified

NSSILTDSQTATK

Charge: 2, Exp. m/z: 683.850, Calc. m/z: 683.347
Data File: D120711_007, Scans: 23634 - 23634

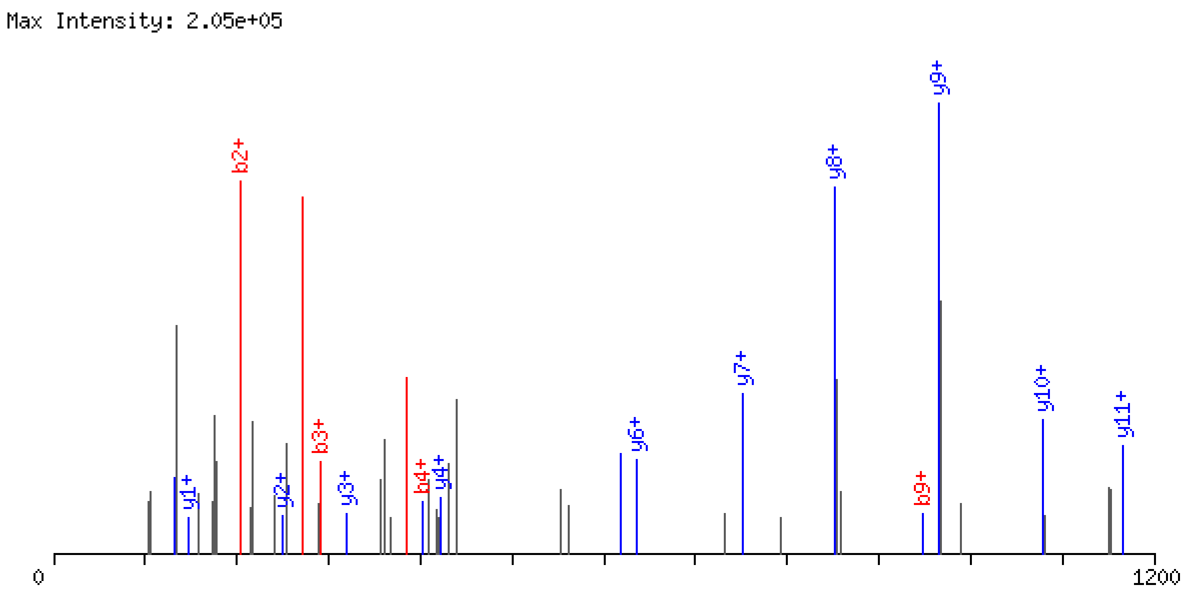

		b+	-H2O	-NH3	y+	-H2O	-NH3		
N	1	115.05		98.02				13	N
S	2	202.08	184.07	185.06	1251.64	1233.63	1234.62	12	S
S	3	289.11	271.10	272.09	1164.61	1146.60	1147.58	11	S
I	4	402.20	384.19	385.17	1077.58	1059.57	1060.55	10	I
L	5	515.28	497.27	498.26	964.49	946.48	947.47	9	L
T	6	616.33	598.32	599.30	851.41	833.40	834.38	8	T
D	7	731.36	713.35	714.33	750.36	732.35	733.34	7	D
S	8	818.39	800.38	801.36	635.34	617.33	618.31	6	S
Q	9	946.45	928.44	929.42	548.30	530.29	531.28	5	Q
T	10	1047.50	1029.48	1030.47	420.25	402.23	403.22	4	T
A	11	1118.53	1100.52	1101.51	319.20	301.19	302.17	3	A
T	12	1219.58	1201.57	1202.55	248.16	230.15	231.13	2	T
K	13				147.11		130.09	1	K

WSN PB2 740-752 phospho

NSS(+79.97)ILTDSQTATK

Charge: 2, Exp. m/z: 723.329, Calc. m/z: 723.332
Data File: D120711_007, Scans: 15996 - 15996

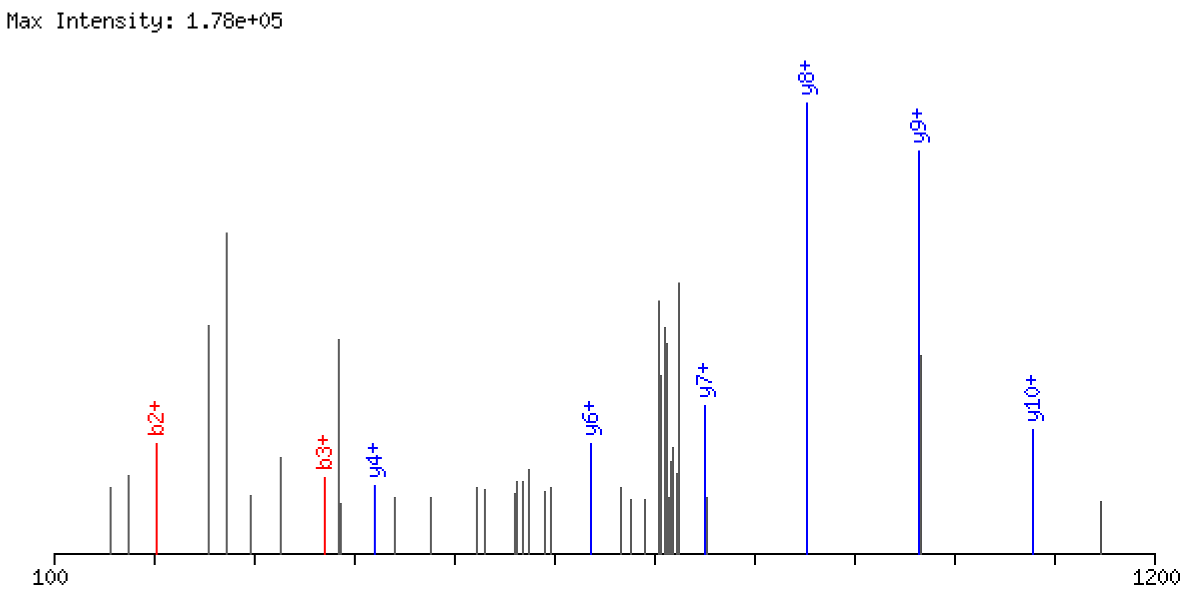

		b+	-H2O	-NH3	y+	-H2O	-NH3		
N	1	115.05		98.02				13	N
S	2	202.08	184.07	185.06	1331.61	1313.60	1314.58	12	S
S	3	369.08	351.07	352.05	1244.58	1226.57	1227.55	11	S
I	4	482.16	464.15	465.14	1077.58	1059.57	1060.55	10	I
L	5	595.25	577.24	578.22	964.49	946.48	947.47	9	L
T	6	696.30	678.29	679.27	851.41	833.40	834.38	8	T
D	7	811.32	793.31	794.30	750.36	732.35	733.34	7	D
S	8	898.36	880.34	881.33	635.34	617.33	618.31	6	S
Q	9	1026.41	1008.40	1009.39	548.30	530.29	531.28	5	Q
T	10	1127.46	1109.45	1110.44	420.25	402.23	403.22	4	T
A	11	1198.50	1180.49	1181.47	319.20	301.19	302.17	3	A
T	12	1299.55	1281.54	1282.52	248.16	230.15	231.13	2	T
K	13				147.11		130.09	1	K

WSN PB1 221-229 M227ox

ALTLNTM(+15.99)TK

Charge: 2, Exp. m/z: 504.773, Calc. m/z: 504.777
Data File: B110620_019, Scans: 440 - 440

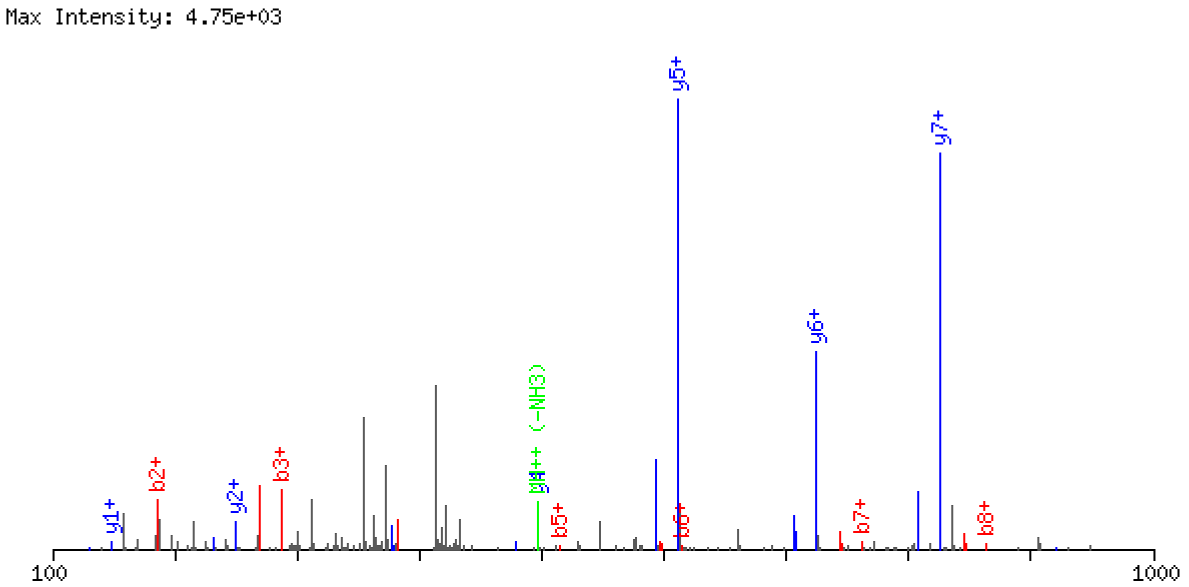

		b+	-H2O	-NH3	y+	-H2O	-NH3		
A	1	72.04						9	A
L	2	185.13			937.50	919.49	920.48	8	L
T	3	286.18	268.17		824.42	806.41	807.39	7	T
L	4	399.26	381.25		723.37	705.36	706.34	6	L
N	5	513.30	495.29	496.28	610.29	592.28	593.26	5	N
T	6	614.35	596.34	597.32	496.24	478.23	479.22	4	T
M	7	761.39	743.38	744.36	395.20	377.19	378.17	3	M
T	8	862.43	844.42	845.41	248.16	230.15	231.13	2	T
K	9				147.11		130.09	1	K

WSN PB1 221-229 unmodified

ALTLNTMTK

Charge: 2, Exp. m/z: 496.776, Calc. m/z: 496.780
Data File: B110620_019, Scans: 536 - 536

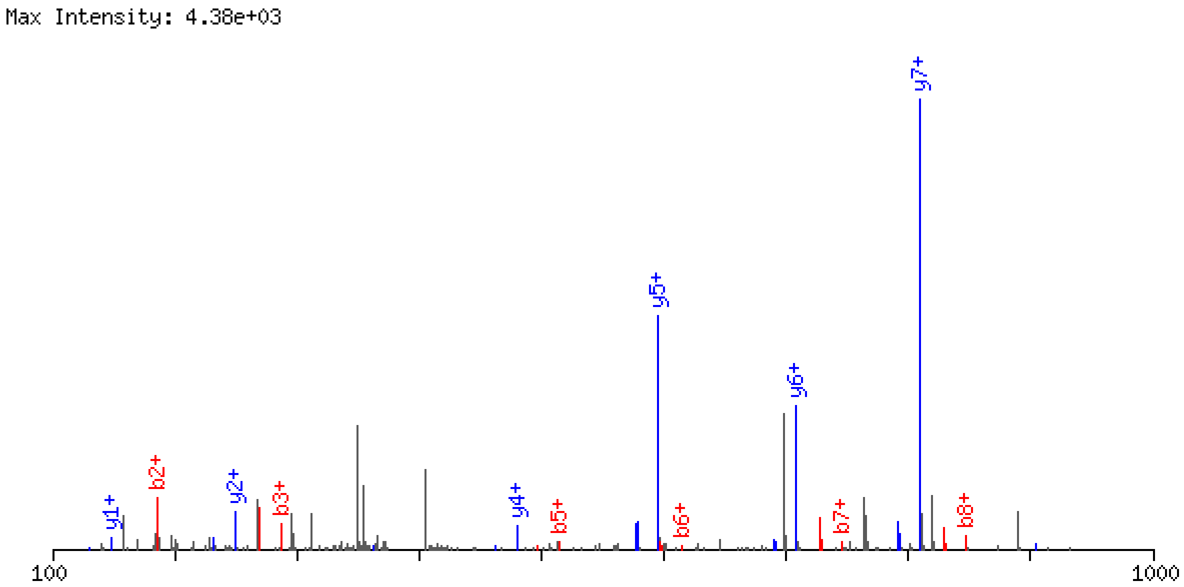

		b+	-H2O	-NH3	y+	-H2O	-NH3		
A	1	72.04						9	A
L	2	185.13			921.51	903.50	904.48	8	L
T	3	286.18	268.17		808.42	790.41	791.40	7	T
L	4	399.26	381.25		707.38	689.37	690.35	6	L
N	5	513.30	495.29	496.28	594.29	576.28	577.27	5	N
T	6	614.35	596.34	597.32	480.25	462.24	463.22	4	T
M	7	745.39	727.38	728.36	379.20	361.19	362.17	3	M
T	8	846.44	828.43	829.41	248.16	230.15	231.13	2	T
K	9				147.11		130.09	1	K

WSN PB1 221-229 phospho M227ox

ALT(+79.97)LNTM(+15.99)TK

Charge: 2, Exp. m/z: 544.756, Calc. m/z: 544.757
Data File: B110620_015, Scans: 539 - 539

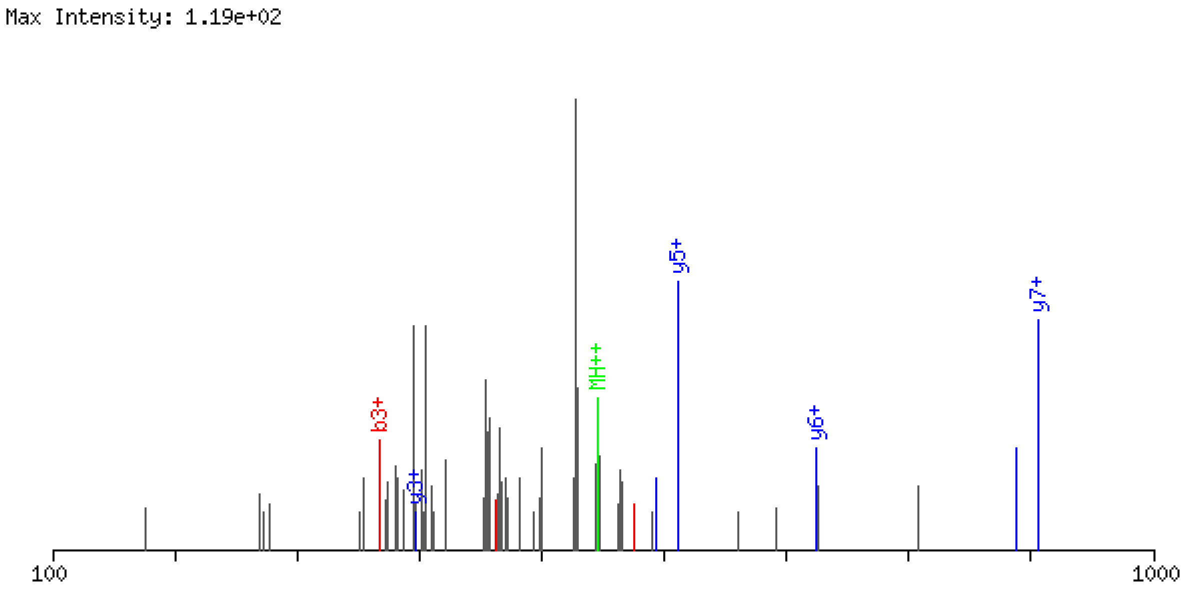

		b+	-H2O	-NH3	y+	-H2O	-NH3		
A	1	72.04						9	A
L	2	185.13			1017.47	999.46	1000.44	8	L
T	3	366.14	348.13		904.38	886.37	887.36	7	T
L	4	479.23	461.22		723.37	705.36	706.34	6	L
N	5	593.27	575.26	576.24	610.29	592.28	593.26	5	N
T	6	694.32	676.31	677.29	496.24	478.23	479.22	4	T
M	7	841.35	823.34	824.33	395.20	377.19	378.17	3	M
T	8	942.40	924.39	925.37	248.16	230.15	231.13	2	T
K	9				147.11		130.09	1	K

WSN PA 214-230 unmodified

LADQSLPPNFSSLENFR

Charge: 3, Exp. m/z: 645.658, Calc. m/z: 645.657
Data File: D120711_007, Scans: 26474 - 26474

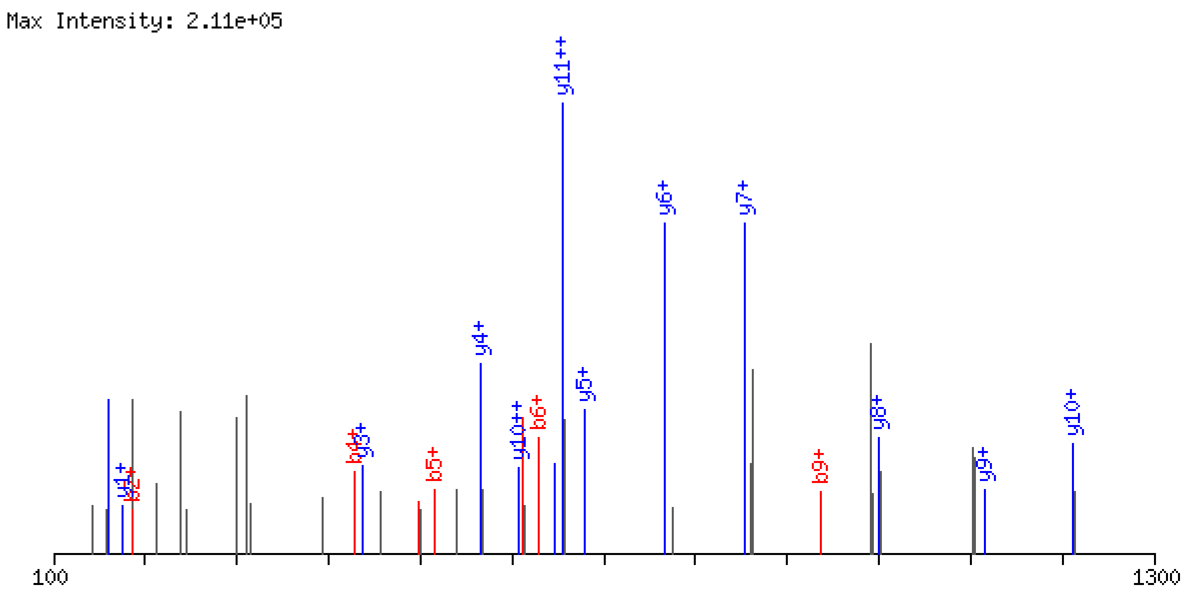

Hiding fragment losses - Click to Show

		b+	b++	y+	y++		
L	1	114.09	57.55			17	L
A	2	185.13	93.07	1821.88	911.44	16	A
D	3	300.16	150.58	1750.84	875.92	15	D
Q	4	428.21	214.61	1635.81	818.41	14	Q
S	5	515.25	258.13	1507.75	754.38	13	S
L	6	628.33	314.67	1420.72	710.86	12	L
P	7	725.38	363.20	1307.64	654.32	11	P
P	8	822.44	411.72	1210.59	605.80	10	P
N	9	936.48	468.74	1113.53	557.27	9	N
F	10	1083.55	542.28	999.49	500.25	8	F
S	11	1170.58	585.79	852.42	426.71	7	S
S	12	1257.61	629.31	765.39	383.20	6	S
L	13	1370.70	685.85	678.36	339.68	5	L
E	14	1499.74	750.37	565.27	283.14	4	E
N	15	1613.78	807.39	436.23	218.62	3	N
F	16	1760.85	880.93	322.19	161.60	2	F
R	17			175.12	88.06	1	R

WSN PA 214-230 phospho + deamidated

LADQSLPPN(+0.98)FSS(+79.97)LENFR

Charge: 2, Exp. m/z: 1008.460, Calc. m/z: 1008.457
Data File: B120705_010, Scans: 928 - 928

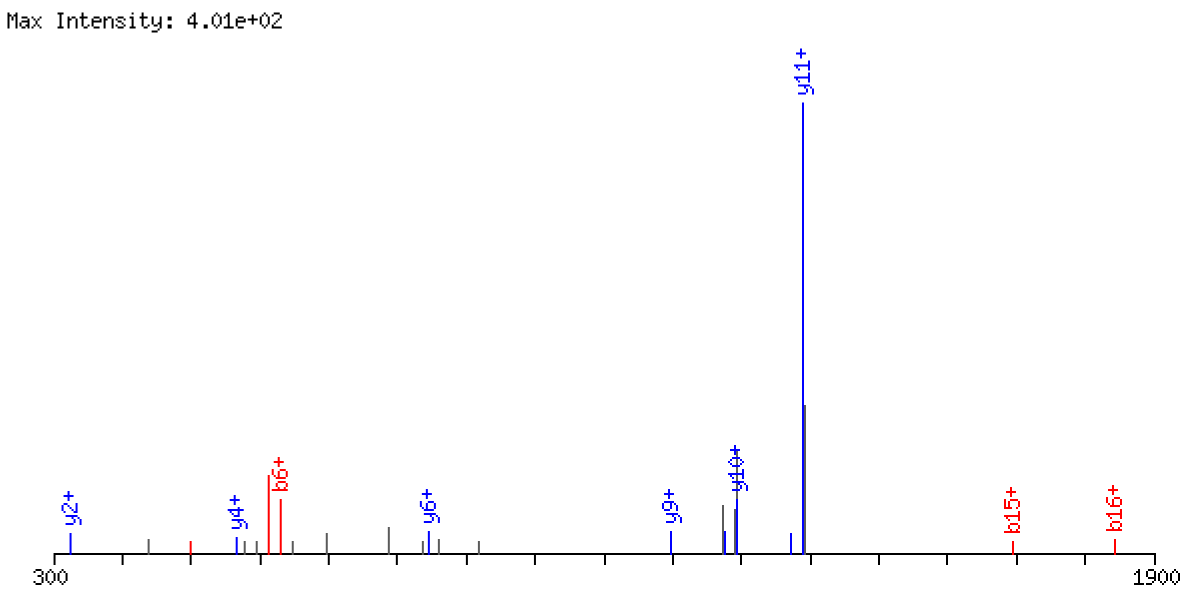

		b+	-H2O	-NH3	y+	-H2O	-NH3		
L	1	114.09						17	L
A	2	185.13			1902.83	1884.82	1885.80	16	A
D	3	300.16	282.14		1831.79	1813.78	1814.76	15	D
Q	4	428.21	410.20		1716.76	1698.75	1699.74	14	Q
S	5	515.25	497.24		1588.70	1570.69	1571.68	13	S
L	6	628.33	610.32		1501.67	1483.66	1484.65	12	L
P	7	725.38	707.37		1388.59	1370.58	1371.56	11	P
P	8	822.44	804.43		1291.54	1273.52	1274.51	10	P
N	9	937.46	919.45	920.44	1194.48	1176.47	1177.46	9	N
F	10	1084.53	1066.52	1067.50	1079.46	1061.45	1062.43	8	F
S	11	1171.56	1153.55	1154.54	932.39	914.38	915.36	7	S
S	12	1338.56	1320.55	1321.53	845.36	827.34	828.33	6	S
L	13	1451.65	1433.63	1434.62	678.36	660.35	661.33	5	L
E	14	1580.69	1562.68	1563.66	565.27	547.26	548.25	4	E
N	15	1694.73	1676.72	1677.70	436.23		419.20	3	N
F	16	1841.80	1823.79	1824.77	322.19		305.16	2	F
R	17				175.12		158.09	1	R

WSN HA 344-382 unmodified

GLFGAIAGFIEGGWTGMIDGWYGYHHQNEQGSGYAADQK

Charge: 3, Exp. m/z: 1396.970, Calc. m/z: 1396.967
Data File: B110818_009, Scans: 1877 - 1877

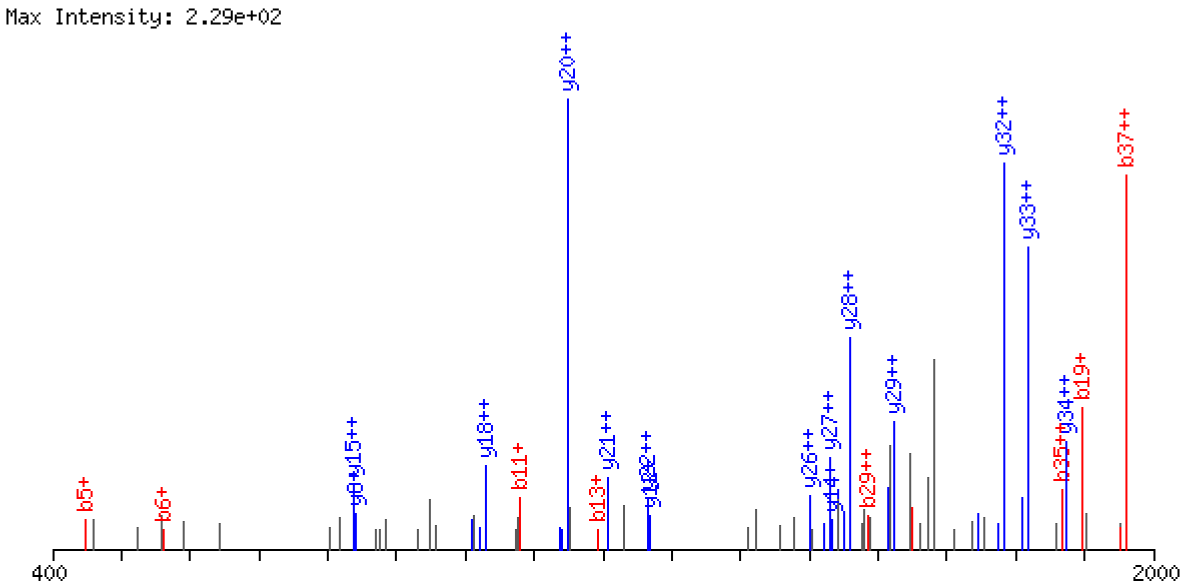

Showing fragment losses - Click to Hide

		b+	-H2O	-NH3	b++	-H2O	-NH3	y+	-H2O	-NH3	y++	-H2O	-NH3		
G	1	58.03			29.52									39	G
L	2	171.11			86.06			4131.87	4113.86	4114.85	2066.44	2057.43	2057.93	38	L
F	3	318.18			159.59			4018.79	4000.78	4001.76	2009.90	2000.89	2001.38	37	F
G	4	375.20			188.10			3871.72	3853.71	3854.69	1936.36	1927.36	1927.85	36	G
A	5	446.24			223.62			3814.70	3796.69	3797.67	1907.85	1898.85	1899.34	35	A
I	6	559.32			280.17			3743.66	3725.65	3726.63	1872.33	1863.33	1863.82	34	I
A	7	630.36			315.68			3630.58	3612.57	3613.55	1815.79	1806.79	1807.28	33	A
G	8	687.38			344.19			3559.54	3541.53	3542.51	1780.27	1771.27	1771.76	32	G
F	9	834.45			417.73			3502.52	3484.51	3485.49	1751.76	1742.76	1743.25	31	F
I	10	947.53			474.27			3355.45	3337.44	3338.42	1678.23	1669.22	1669.72	30	I
E	11	1076.58	1058.57		538.79	529.79		3242.37	3224.36	3225.34	1621.69	1612.68	1613.17	29	E
G	12	1133.60	1115.59		567.30	558.30		3113.32	3095.31	3096.30	1557.17	1548.16	1548.65	28	G
G	13	1190.62	1172.61		595.81	586.81		3056.30	3038.29	3039.28	1528.65	1519.65	1520.14	27	G
W	14	1376.70	1358.69		688.85	679.85		2999.28	2981.27	2982.25	1500.14	1491.14	1491.63	26	W
T	15	1477.75	1459.74		739.38	730.37		2813.20	2795.19	2796.17	1407.10	1398.10	1398.59	25	T
G	16	1534.77	1516.76		767.89	758.88		2712.15	2694.14	2695.13	1356.58	1347.57	1348.07	24	G
M	17	1665.81	1647.80		833.41	824.40		2655.13	2637.12	2638.11	1328.07	1319.06	1319.56	23	M
I	18	1778.89	1760.88		889.95	880.95		2524.09	2506.08	2507.06	1262.55	1253.54	1254.04	22	I
D	19	1893.92	1875.91		947.46	938.46		2411.01	2393.00	2393.98	1206.01	1197.00	1197.49	21	D
G	20	1950.94	1932.93		975.97	966.97		2295.98	2277.97	2278.95	1148.49	1139.49	1139.98	20	G
W	21	2137.02	2119.01		1069.01	1060.01		2238.96	2220.95	2221.93	1119.98	1110.98	1111.47	19	W
Y	22	2300.08	2282.07		1150.55	1141.54		2052.88	2034.87	2035.85	1026.94	1017.94	1018.43	18	Y
G	23	2357.11	2339.10		1179.06	1170.05		1889.82	1871.81	1872.79	945.41	936.41	936.90	17	G
Y	24	2520.17	2502.16		1260.59	1251.58		1832.79	1814.78	1815.77	916.90	907.90	908.39	16	Y
H	25	2657.23	2639.22		1329.12	1320.11		1669.73	1651.72	1652.70	835.37	826.36	826.86	15	H
H	26	2794.29	2776.28		1397.65	1388.64		1532.67	1514.66	1515.65	766.84	757.83	758.33	14	H
Q	27	2922.35	2904.34		1461.68	1452.67		1395.61	1377.60	1378.59	698.31	689.31	689.80	13	Q

		b+	-H2O	-NH3	b++	-H2O	-NH3	y+	-H2O	-NH3	y++	-H2O	-NH3		
N	28	3036.39	3018.38	3019.36	1518.70	1509.69	1510.18	1267.55	1249.54	1250.53	634.28	625.28	625.77	12	N
E	29	3165.43	3147.42	3148.40	1583.22	1574.21	1574.71	1153.51	1135.50	1136.49	577.26	568.25	568.75	11	E
Q	30	3293.49	3275.48	3276.46	1647.25	1638.24	1638.74	1024.47	1006.46	1007.44	512.74	503.73	504.23	10	Q
G	31	3350.51	3332.50	3333.48	1675.76	1666.75	1667.25	896.41	878.40	879.38	448.71	439.70	440.20	9	G
S	32	3437.54	3419.53	3420.52	1719.28	1710.27	1710.76	839.39	821.38	822.36	420.20	411.19	411.69	8	S
G	33	3494.56	3476.55	3477.54	1747.79	1738.78	1739.27	752.36	734.35	735.33	376.68	367.68	368.17	7	G
Y	34	3657.63	3639.62	3640.60	1829.32	1820.31	1820.80	695.34	677.33	678.31	348.17	339.17	339.66	6	Y
A	35	3728.67	3710.65	3711.64	1864.84	1855.83	1856.32	532.27	514.26	515.25	266.64	257.63	258.13	5	A
A	36	3799.70	3781.69	3782.68	1900.35	1891.35	1891.84	461.24	443.22	444.21	231.12	222.12	222.61	4	A
D	37	3914.73	3896.72	3897.70	1957.87	1948.86	1949.35	390.20	372.19	373.17	195.60	186.60	187.09	3	D
Q	38	4042.79	4024.78	4025.76	2021.90	2012.89	2013.38	275.17		258.14	138.09		129.58	2	Q
K	39							147.11		130.09	74.06		65.55	1	K

WSN HA 344-382 phospho

GLFGAIAGFIEGGWT(+79.97)GMIDGWYGYHHQNEQGSGYAADQK

Charge: 3, Exp. m/z: 1423.630, Calc. m/z: 1423.624
Data File: B110818_004, Scans: 1377 - 1377

Max Intensity: 4.24e+02

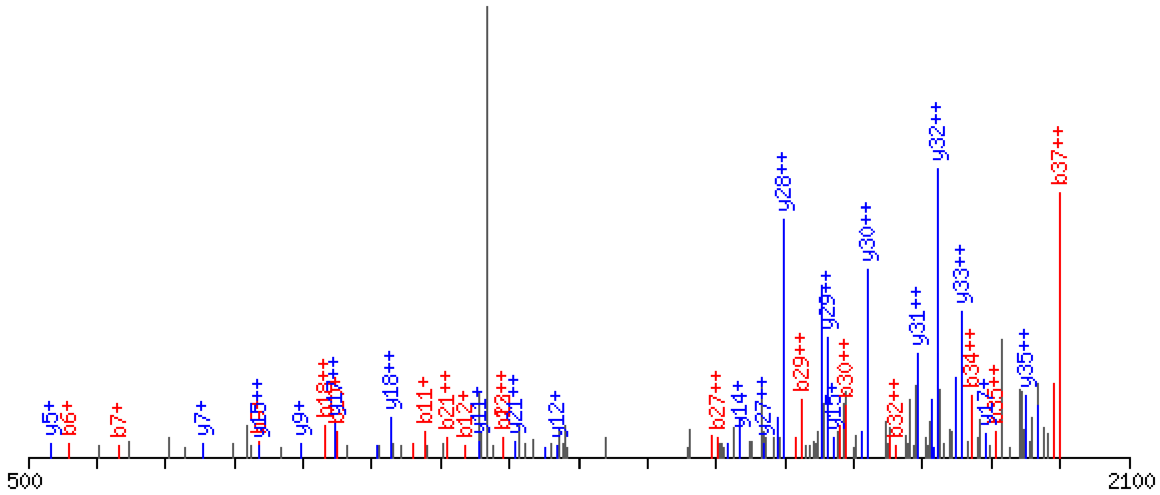

Showing fragment losses - Click to Hide

		b+	-H2O	-NH3	b++	-H2O	-NH3	y+	-H2O	-NH3	y++	-H2O	-NH3		
G	1	58.03			29.52									39	G
L	2	171.11			86.06			4211.84	4193.83	4194.81	2106.42	2097.42	2097.91	38	L
F	3	318.18			159.59			4098.75	4080.74	4081.73	2049.88	2040.88	2041.37	37	F
G	4	375.20			188.10			3951.69	3933.68	3934.66	1976.35	1967.34	1967.83	36	G
A	5	446.24			223.62			3894.66	3876.65	3877.64	1947.84	1938.83	1939.32	35	A
I	6	559.32			280.17			3823.63	3805.62	3806.60	1912.32	1903.31	1903.80	34	I
A	7	630.36			315.68			3710.54	3692.53	3693.52	1855.78	1846.77	1847.26	33	A
G	8	687.38			344.19			3639.51	3621.50	3622.48	1820.26	1811.25	1811.74	32	G
F	9	834.45			417.73			3582.48	3564.47	3565.46	1791.75	1782.74	1783.23	31	F
I	10	947.53			474.27			3435.42	3417.41	3418.39	1718.21	1709.21	1709.70	30	I
E	11	1076.58	1058.57		538.79	529.79		3322.33	3304.32	3305.31	1661.67	1652.66	1653.16	29	E
G	12	1133.60	1115.59		567.30	558.30		3193.29	3175.28	3176.26	1597.15	1588.14	1588.64	28	G
G	13	1190.62	1172.61		595.81	586.81		3136.27	3118.26	3119.24	1568.64	1559.63	1560.12	27	G
W	14	1376.70	1358.69		688.85	679.85		3079.25	3061.24	3062.22	1540.13	1531.12	1531.61	26	W
T	15	1557.71	1539.70		779.36	770.36		2893.17	2875.16	2876.14	1447.09	1438.08	1438.57	25	T
G	16	1614.74	1596.72		807.87	798.87		2712.15	2694.14	2695.13	1356.58	1347.57	1348.07	24	G
M	17	1745.78	1727.77		873.39	864.39		2655.13	2637.12	2638.11	1328.07	1319.06	1319.56	23	M
I	18	1858.86	1840.85		929.93	920.93		2524.09	2506.08	2507.06	1262.55	1253.54	1254.04	22	I
D	19	1973.89	1955.88		987.45	978.44		2411.01	2393.00	2393.98	1206.01	1197.00	1197.49	21	D
G	20	2030.91	2012.90		1015.96	1006.95		2295.98	2277.97	2278.95	1148.49	1139.49	1139.98	20	G
W	21	2216.99	2198.98		1109.00	1099.99		2238.96	2220.95	2221.93	1119.98	1110.98	1111.47	19	W
Y	22	2380.05	2362.04		1190.53	1181.52		2052.88	2034.87	2035.85	1026.94	1017.94	1018.43	18	Y
G	23	2437.07	2419.06		1219.04	1210.03		1889.82	1871.81	1872.79	945.41	936.41	936.90	17	G
Y	24	2600.14	2582.13		1300.57	1291.57		1832.79	1814.78	1815.77	916.90	907.90	908.39	16	Y
H	25	2737.19	2719.18		1369.10	1360.10		1669.73	1651.72	1652.70	835.37	826.36	826.86	15	H
H	26	2874.25	2856.24		1437.63	1428.63		1532.67	1514.66	1515.65	766.84	757.83	758.33	14	H
Q	27	3002.31	2984.30		1501.66	1492.65		1395.61	1377.60	1378.59	698.31	689.31	689.80	13	Q

		b+	-H2O	-NH3	b++	-H2O	-NH3	y+	-H2O	-NH3	y++	-H2O	-NH3		
N	28	3116.35	3098.34	3099.33	1558.68	1549.68	1550.17	1267.55	1249.54	1250.53	634.28	625.28	625.77	12	N
E	29	3245.40	3227.39	3228.37	1623.20	1614.20	1614.69	1153.51	1135.50	1136.49	577.26	568.25	568.75	11	E
Q	30	3373.46	3355.45	3356.43	1687.23	1678.23	1678.72	1024.47	1006.46	1007.44	512.74	503.73	504.23	10	Q
G	31	3430.48	3412.47	3413.45	1715.74	1706.74	1707.23	896.41	878.40	879.38	448.71	439.70	440.20	9	G
S	32	3517.51	3499.50	3500.48	1759.26	1750.25	1750.75	839.39	821.38	822.36	420.20	411.19	411.69	8	S
G	33	3574.53	3556.52	3557.50	1787.77	1778.76	1779.26	752.36	734.35	735.33	376.68	367.68	368.17	7	G
Y	34	3737.59	3719.58	3720.57	1869.30	1860.30	1860.79	695.34	677.33	678.31	348.17	339.17	339.66	6	Y
A	35	3808.63	3790.62	3791.60	1904.82	1895.81	1896.31	532.27	514.26	515.25	266.64	257.63	258.13	5	A
A	36	3879.67	3861.66	3862.64	1940.34	1931.33	1931.82	461.24	443.22	444.21	231.12	222.12	222.61	4	A
D	37	3994.70	3976.68	3977.67	1997.85	1988.85	1989.34	390.20	372.19	373.17	195.60	186.60	187.09	3	D
Q	38	4122.75	4104.74	4105.73	2061.88	2052.88	2053.37	275.17		258.14	138.09		129.58	2	Q
K	39							147.11		130.09	74.06		65.55	1	K

WSN NP 9-19 unmodified

SYEQMETDGER

Charge: 2, Exp. m/z: 672.773, Calc. m/z: 672.777
Data File: B110318_004, Scans: 1748 - 1748

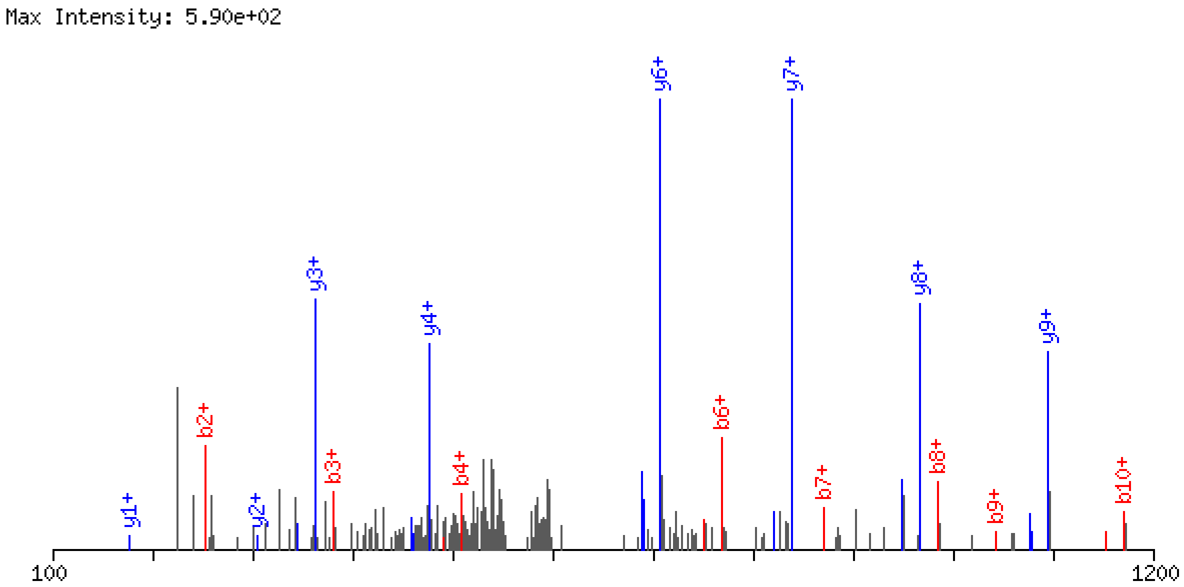

		b+	-H2O	y+	-H2O	-NH3		
S	1	88.04	70.03				11	S
Y	2	251.10	233.09	1257.51	1239.49	1240.48	10	Y
E	3	380.15	362.13	1094.44	1076.43	1077.42	9	E
Q	4	508.20	490.19	965.40	947.39	948.37	8	Q
M	5	639.24	621.23	837.34	819.33	820.31	7	M
E	6	768.29	750.28	706.30	688.29	689.27	6	E
T	7	869.33	851.32	577.26	559.25	560.23	5	T
D	8	984.36	966.35	476.21	458.20	459.18	4	D
G	9	1041.38	1023.37	361.18	343.17	344.16	3	G
E	10	1170.43	1152.42	304.16	286.15	287.14	2	E
R	11			175.12		158.09	1	R

WSN NP 9-19 phospho

S(+79.97)YEQMETDGER

Charge: 2, Exp. m/z: 712.757, Calc. m/z: 712.757
Data File: B110406_003, Scans: 423 - 423

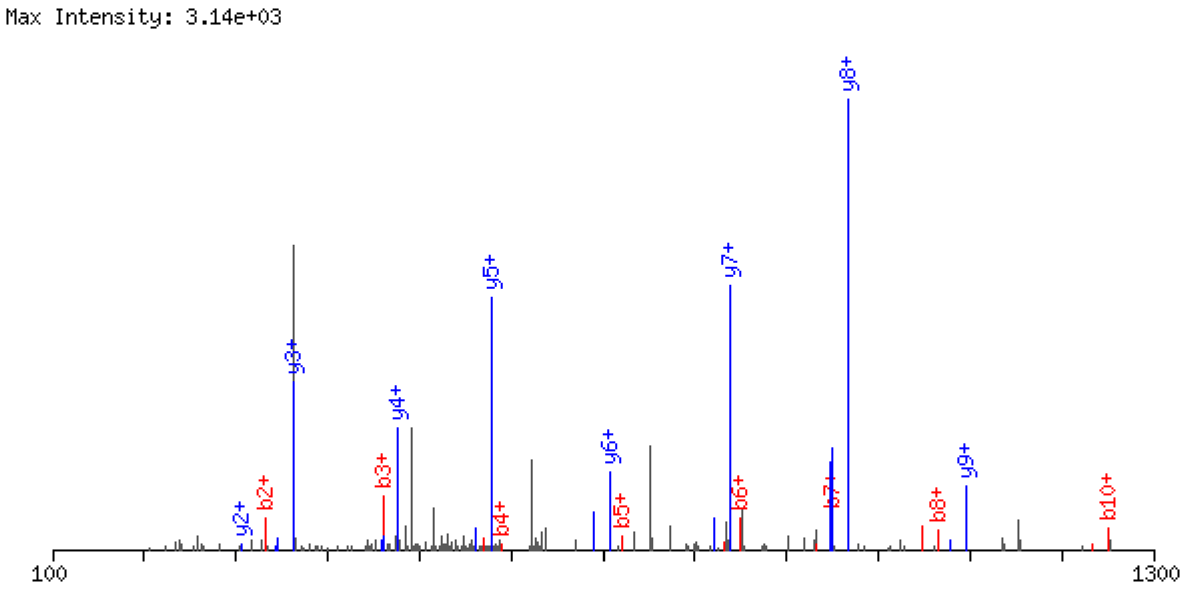

		b+	-H2O	y+	-H2O	-NH3		
S	1	168.01	150.00				11	S
Y	2	331.07	313.06	1257.51	1239.49	1240.48	10	Y
E	3	460.11	442.10	1094.44	1076.43	1077.42	9	E
Q	4	588.17	570.16	965.40	947.39	948.37	8	Q
M	5	719.21	701.20	837.34	819.33	820.31	7	M
E	6	848.25	830.24	706.30	688.29	689.27	6	E
T	7	949.30	931.29	577.26	559.25	560.23	5	T
D	8	1064.33	1046.32	476.21	458.20	459.18	4	D
G	9	1121.35	1103.34	361.18	343.17	344.16	3	G
E	10	1250.39	1232.38	304.16	286.15	287.14	2	E
R	11			175.12		158.09	1	R

WSN NP 163-174 carbamidomethyl

MC(+57.02)SLMQGSTLPR

Charge: 2, Exp. m/z: 690.825, Calc. m/z: 690.827
Data File: B110609_017, Scans: 1450 - 1450

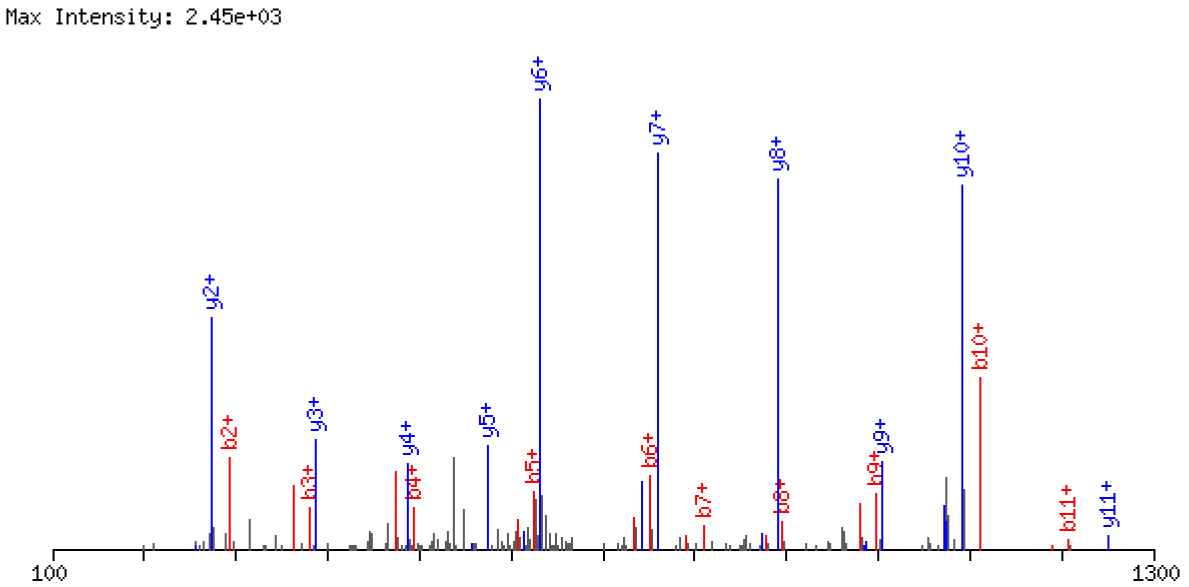

		b+	-H2O	y+	-H2O	-NH3		
M	1	132.05					12	M
C	2	292.08		1249.60	1231.59	1232.58	11	C
S	3	379.11	361.10	1089.57	1071.56	1072.55	10	S
L	4	492.19	474.18	1002.54	984.53	985.51	9	L
M	5	623.24	605.22	889.46	871.45	872.43	8	M
Q	6	751.29	733.28	758.42	740.40	741.39	7	Q
G	7	808.32	790.30	630.36	612.35	613.33	6	G
S	8	895.35	877.34	573.34	555.32	556.31	5	S
T	9	996.39	978.38	486.30	468.29	469.28	4	T
L	10	1109.48	1091.47	385.26		368.23	3	L
P	11	1206.53	1188.52	272.17		255.15	2	P
R	12			175.12		158.09	1	R

WSN NP 163-174 carbamidomethyl and phospho

MC(+57.02)S(+79.97)LMQGSTLPR

Charge: 2, Exp. m/z: 730.810, Calc. m/z: 730.807
Data File: B110609_010, Scans: 755 - 755

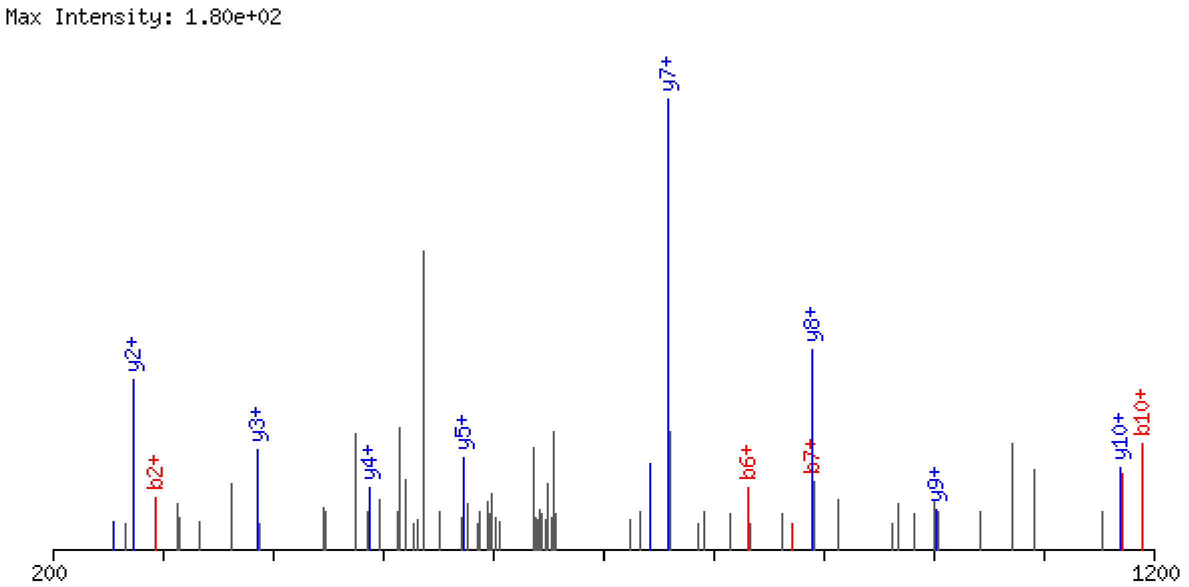

		b+	-H2O	y+	-H2O	-NH3		
M	1	132.05					12	M
C	2	292.08		1329.57	1311.56	1312.54	11	C
S	3	459.08	441.07	1169.54	1151.53	1152.51	10	S
L	4	572.16	554.15	1002.54	984.53	985.51	9	L
M	5	703.20	685.19	889.46	871.45	872.43	8	M
Q	6	831.26	813.25	758.42	740.40	741.39	7	Q
G	7	888.28	870.27	630.36	612.35	613.33	6	G
S	8	975.31	957.30	573.34	555.32	556.31	5	S
T	9	1076.36	1058.35	486.30	468.29	469.28	4	T
L	10	1189.45	1171.43	385.26		368.23	3	L
P	11	1286.50	1268.49	272.17		255.15	2	P
R	12			175.12		158.09	1	R

WSN NP 294-305 phospho

EGYSLVGIDPFR

Charge: 2, Exp. m/z: 676.846, Calc. m/z: 676.847
Data File: B120705_010, Scans: 938 - 938

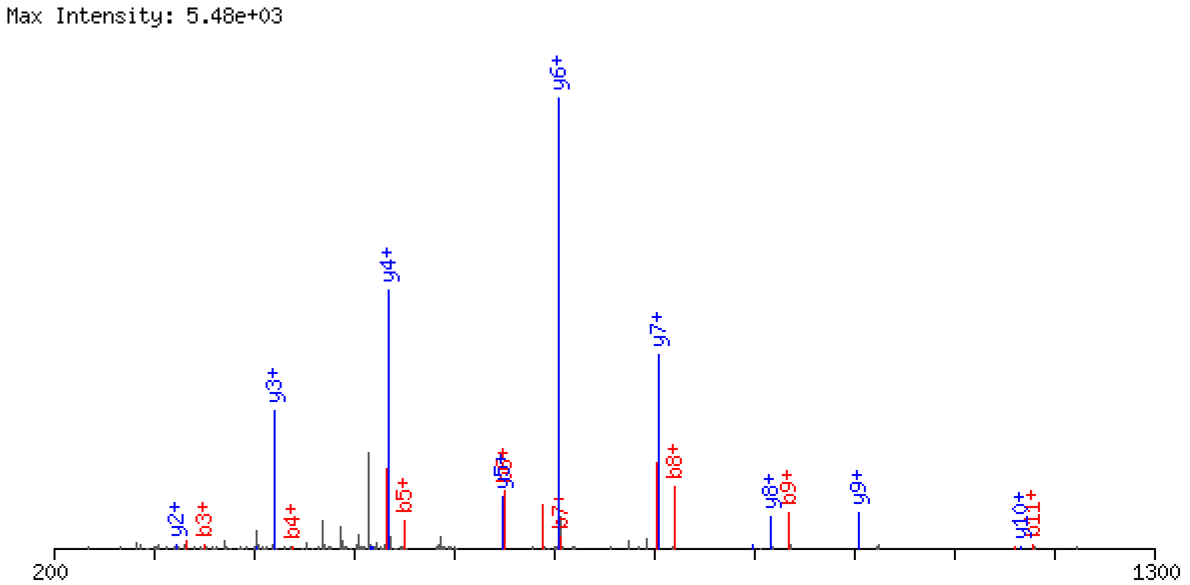

		b+	-H2O	y+	-H2O	-NH3		
E	1	130.05	112.04				12	E
G	2	187.07	169.06	1223.64	1205.63	1206.62	11	G
Y	3	350.13	332.12	1166.62	1148.61	1149.59	10	Y
S	4	437.17	419.16	1003.56	985.55	986.53	9	S
L	5	550.25	532.24	916.53	898.51	899.50	8	L
V	6	649.32	631.31	803.44	785.43	786.41	7	V
G	7	706.34	688.33	704.37	686.36	687.35	6	G
I	8	819.42	801.41	647.35	629.34	630.32	5	I
D	9	934.45	916.44	534.27	516.26	517.24	4	D
P	10	1031.50	1013.49	419.24		402.21	3	P
F	11	1178.57	1160.56	322.19		305.16	2	F
R	12			175.12		158.09	1	R

WSN NP 294-305 phospho

EGYS(+79.97)LVGIDPFR

Charge: 2, Exp. m/z: 716.829, Calc. m/z: 716.827
Data File: B120705_010, Scans: 1005 - 1005

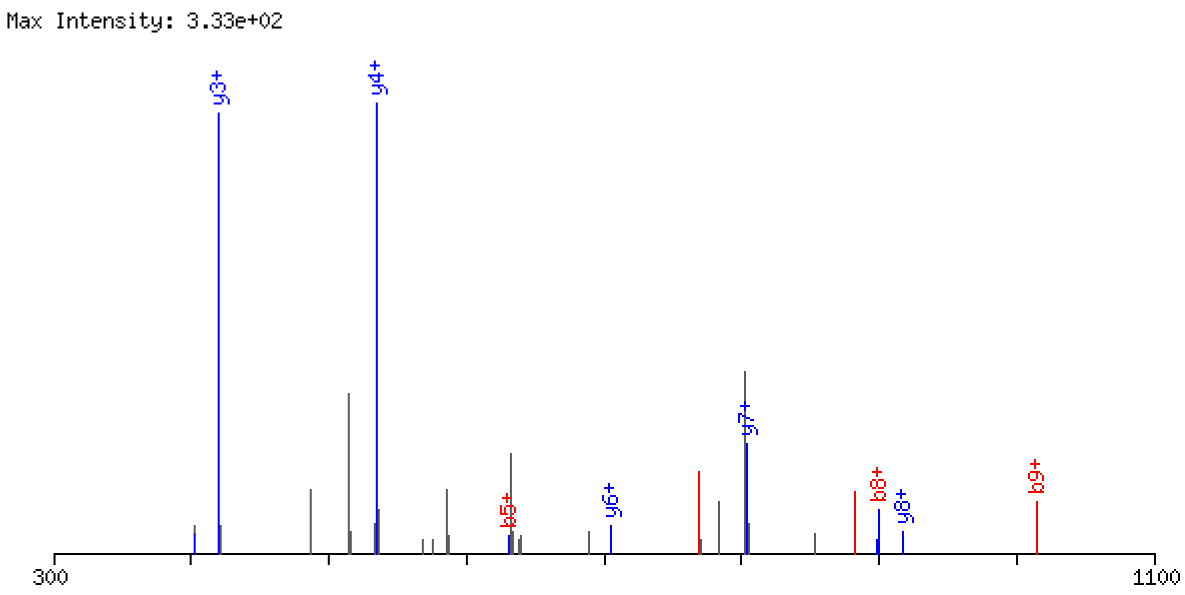

		b+	-H2O	y+	-H2O	-NH3		
E	1	130.05	112.04				12	E
G	2	187.07	169.06	1303.61	1285.60	1286.58	11	G
Y	3	350.13	332.12	1246.59	1228.58	1229.56	10	Y
S	4	517.13	499.12	1083.52	1065.51	1066.50	9	S
L	5	630.22	612.21	916.53	898.51	899.50	8	L
V	6	729.29	711.27	803.44	785.43	786.41	7	V
G	7	786.31	768.30	704.37	686.36	687.35	6	G
I	8	899.39	881.38	647.35	629.34	630.32	5	I
D	9	1014.42	996.41	534.27	516.26	517.24	4	D
P	10	1111.47	1093.46	419.24		402.21	3	P
F	11	1258.54	1240.53	322.19		305.16	2	F
R	12			175.12		158.09	1	R

WSN NP 362-382 modified

GVQIASNENMETMESSTLELR

Charge: 2, Exp. m/z: 1171.040, Calc. m/z: 1170.052
Data File: D120711_007, Scans: 24977 - 24977

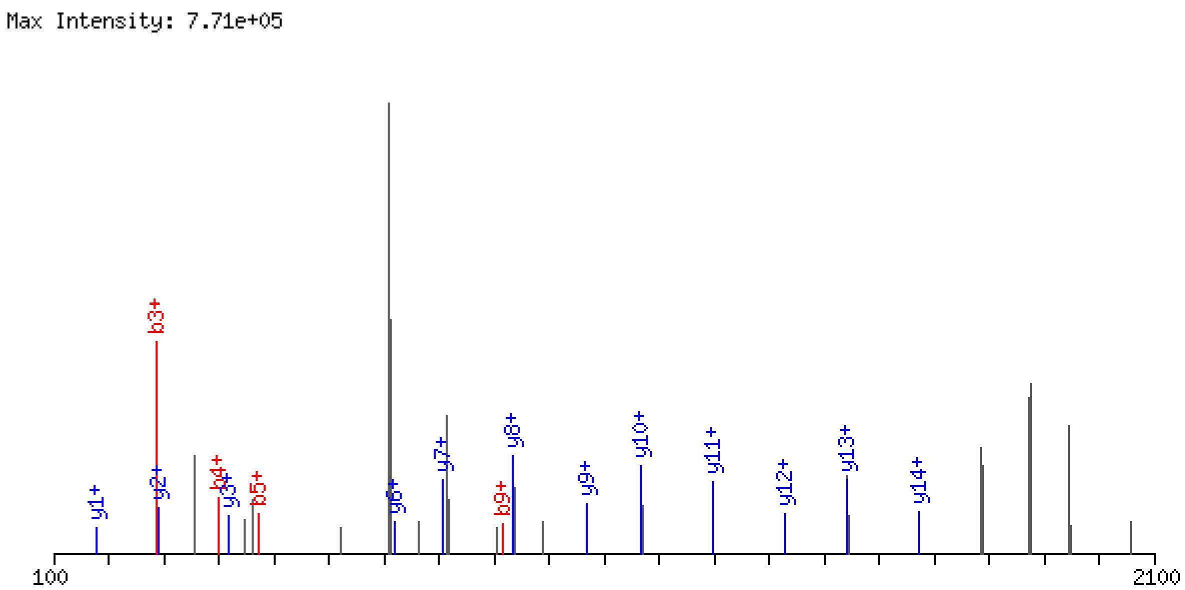

		b+	-H2O	-NH3	y+	-H2O	-NH3		
G	1	58.03						21	G
V	2	157.10			2282.06	2264.05	2265.04	20	V
Q	3	285.16			2183.00	2164.98	2165.97	19	Q
I	4	398.24			2054.94	2036.93	2037.91	18	I
A	5	469.28			1941.85	1923.84	1924.83	17	A
S	6	556.31	538.30		1870.82	1852.81	1853.79	16	S
N	7	670.35	652.34	653.33	1783.78	1765.77	1766.76	15	N
E	8	799.39	781.38	782.37	1669.74	1651.73	1652.71	14	E
N	9	913.44	895.43	896.41	1540.70	1522.69	1523.67	13	N
M	10	1044.48	1026.47	1027.45	1426.66	1408.64	1409.63	12	M
E	11	1173.52	1155.51	1156.49	1295.61	1277.60	1278.59	11	E
T	12	1274.57	1256.56	1257.54	1166.57	1148.56	1149.55	10	T
M	13	1405.61	1387.60	1388.58	1065.52	1047.51	1048.50	9	M
E	14	1534.65	1516.64	1517.62	934.48	916.47	917.46	8	E
S	15	1621.68	1603.67	1604.66	805.44	787.43	788.41	7	S
S	16	1708.72	1690.70	1691.69	718.41	700.40	701.38	6	S
T	17	1809.76	1791.75	1792.74	631.38	613.37	614.35	5	T
L	18	1922.85	1904.84	1905.82	530.33	512.32	513.30	4	L
E	19	2051.89	2033.88	2034.86	417.25	399.24	400.22	3	E
L	20	2164.97	2146.96	2147.95	288.20		271.18	2	L
R	21				175.12		158.09	1	R

WSN NP 362-382 phospho

GVQIASNENMETMESST(+79.97)LELR

Charge: 2, Exp. m/z: 1210.520, Calc. m/z: 1210.032
Data File: D120711_007, Scans: 20755 - 20755

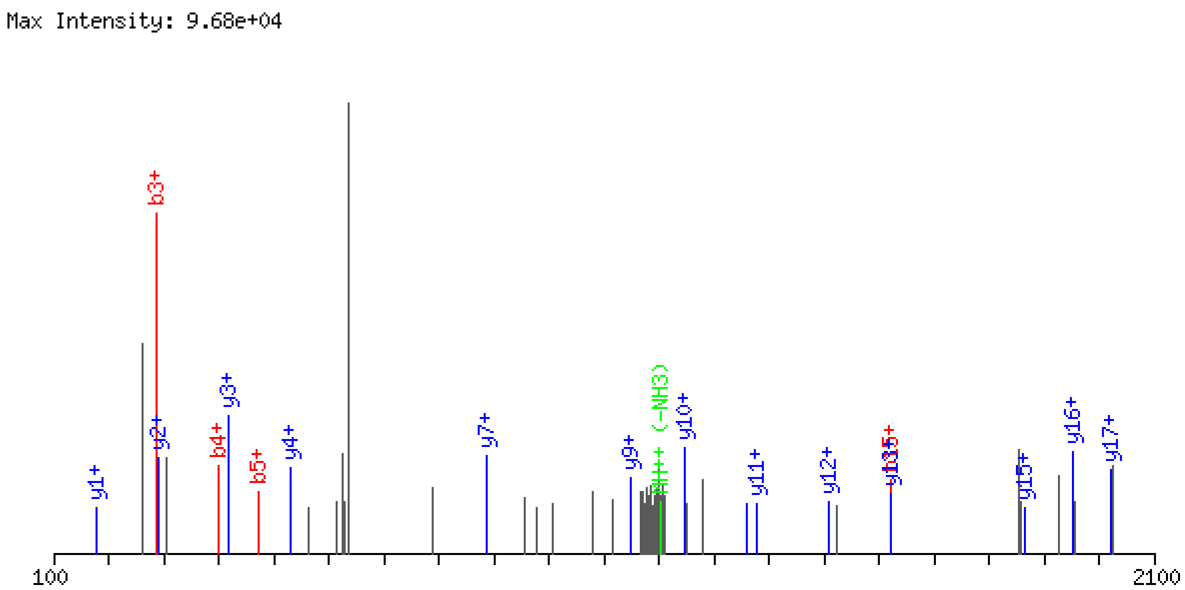

		b+	-H2O	-NH3	y+	-H2O	-NH3		
G	1	58.03						21	G
V	2	157.10			2362.03	2344.02	2345.00	20	V
Q	3	285.16			2262.96	2244.95	2245.94	19	Q
I	4	398.24			2134.90	2116.89	2117.88	18	I
A	5	469.28			2021.82	2003.81	2004.79	17	A
S	6	556.31	538.30		1950.78	1932.77	1933.76	16	S
N	7	670.35	652.34	653.33	1863.75	1845.74	1846.72	15	N
E	8	799.39	781.38	782.37	1749.71	1731.70	1732.68	14	E
N	9	913.44	895.43	896.41	1620.66	1602.65	1603.64	13	N
M	10	1044.48	1026.47	1027.45	1506.62	1488.61	1489.60	12	M
E	11	1173.52	1155.51	1156.49	1375.58	1357.57	1358.55	11	E
T	12	1274.57	1256.56	1257.54	1246.54	1228.53	1229.51	10	T
M	13	1405.61	1387.60	1388.58	1145.49	1127.48	1128.46	9	M
E	14	1534.65	1516.64	1517.62	1014.45	996.44	997.42	8	E
S	15	1621.68	1603.67	1604.66	885.41	867.40	868.38	7	S
S	16	1708.72	1690.70	1691.69	798.38	780.37	781.35	6	S
T	17	1889.73	1871.72	1872.70	711.34	693.33	694.32	5	T
L	18	2002.81	1984.80	1985.79	530.33	512.32	513.30	4	L
E	19	2131.86	2113.85	2114.83	417.25	399.24	400.22	3	E
L	20	2244.94	2226.93	2227.91	288.20		271.18	2	L
R	21				175.12		158.09	1	R

WSN NP 401-416 unmodified

ASSGQISIQPTFSVQR

Charge: 2, Exp. m/z: 853.447, Calc. m/z: 853.447
Data File: B110818_008, Scans: 744 - 744

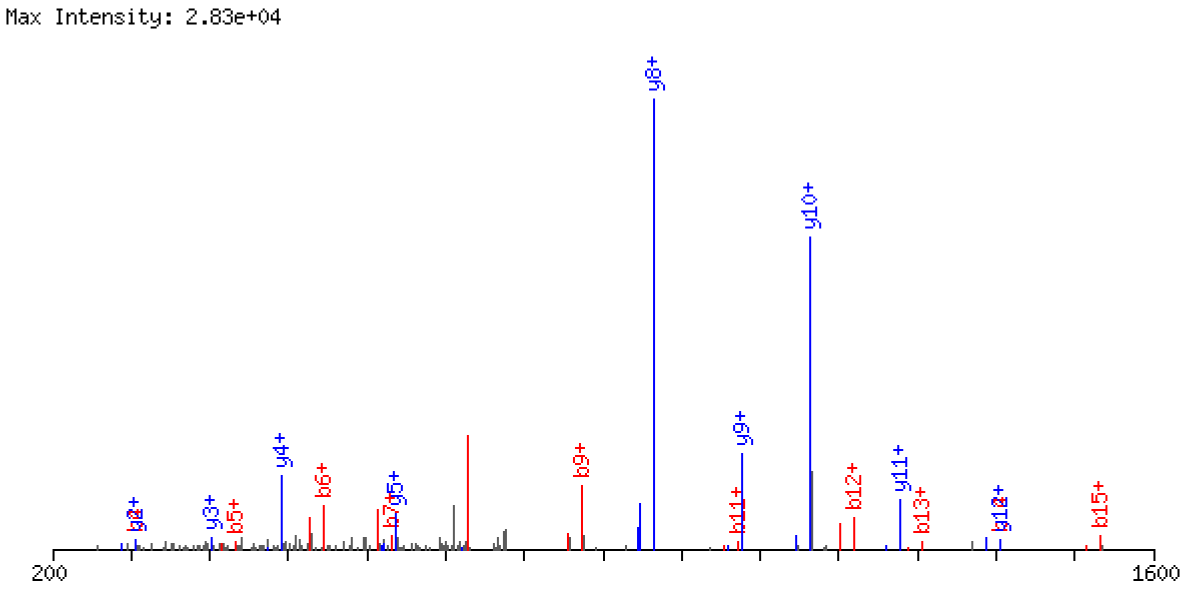

		b+	-H2O	y+	-H2O	-NH3		
A	1	72.04					16	A
S	2	159.08	141.07	1634.85	1616.84	1617.82	15	S
S	3	246.11	228.10	1547.82	1529.81	1530.79	14	S
G	4	303.13	285.12	1460.79	1442.78	1443.76	13	G
Q	5	431.19	413.18	1403.76	1385.75	1386.74	12	Q
I	6	544.27	526.26	1275.71	1257.70	1258.68	11	I
S	7	631.30	613.29	1162.62	1144.61	1145.59	10	S
I	8	744.39	726.38	1075.59	1057.58	1058.56	9	I
Q	9	872.45	854.44	962.51	944.49	945.48	8	Q
P	10	969.50	951.49	834.45	816.44	817.42	7	P
T	11	1070.55	1052.54	737.39	719.38	720.37	6	T
F	12	1217.62	1199.61	636.35	618.34	619.32	5	F
S	13	1304.65	1286.64	489.28	471.27	472.25	4	S
V	14	1403.72	1385.71	402.25		385.22	3	V
Q	15	1531.78	1513.76	303.18		286.15	2	Q
R	16			175.12		158.09	1	R

WSN NP 401-416 phospho

ASS(+79.97)GQISIQPTFSVQR

Charge: 2, Exp. m/z: 893.434, Calc. m/z: 893.432
Data File: B110616_003, Scans: 914 - 914

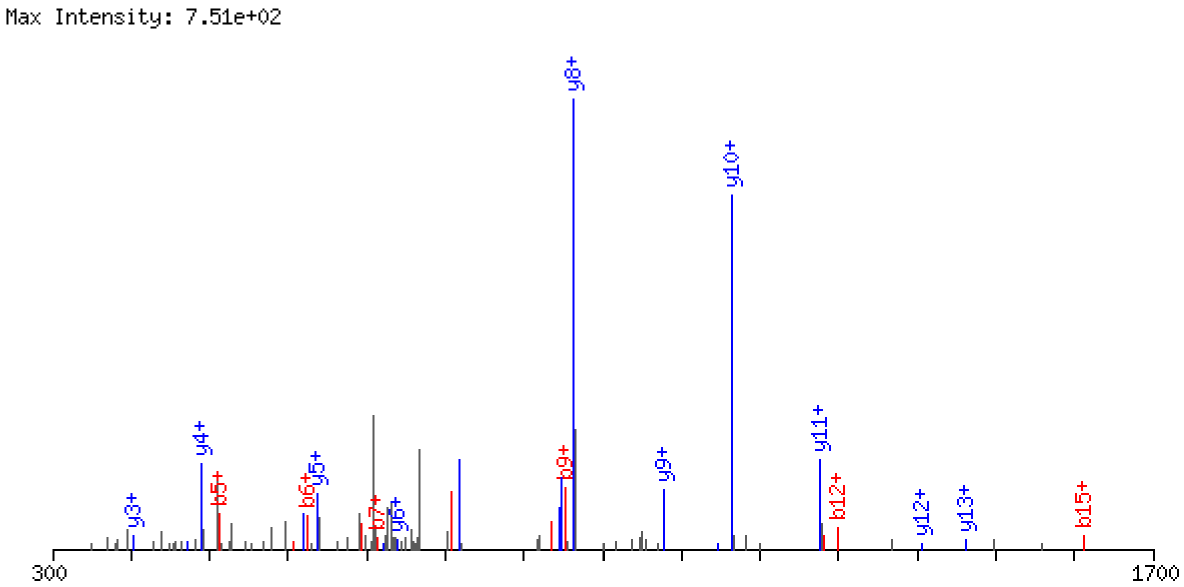

		b+	-H2O	y+	-H2O	-NH3		
A	1	72.04					16	A
S	2	159.08	141.07	1714.82	1696.81	1697.79	15	S
S	3	326.07	308.06	1627.78	1609.77	1610.76	14	S
G	4	383.10	365.09	1460.79	1442.78	1443.76	13	G
Q	5	511.15	493.14	1403.76	1385.75	1386.74	12	Q
I	6	624.24	606.23	1275.71	1257.70	1258.68	11	I
S	7	711.27	693.26	1162.62	1144.61	1145.59	10	S
I	8	824.35	806.34	1075.59	1057.58	1058.56	9	I
Q	9	952.41	934.40	962.51	944.49	945.48	8	Q
P	10	1049.47	1031.46	834.45	816.44	817.42	7	P
T	11	1150.51	1132.50	737.39	719.38	720.37	6	T
F	12	1297.58	1279.57	636.35	618.34	619.32	5	F
S	13	1384.61	1366.60	489.28	471.27	472.25	4	S
V	14	1483.68	1465.67	402.25		385.22	3	V
Q	15	1611.74	1593.73	303.18		286.15	2	Q
R	16			175.12		158.09	1	R

WSN NP 447-461 unmodified

LMESARPEDVSFQGR

Charge: 3, Exp. m/z: 574.616, Calc. m/z: 574.614
Data File: B110318_004, Scans: 2072 - 2072

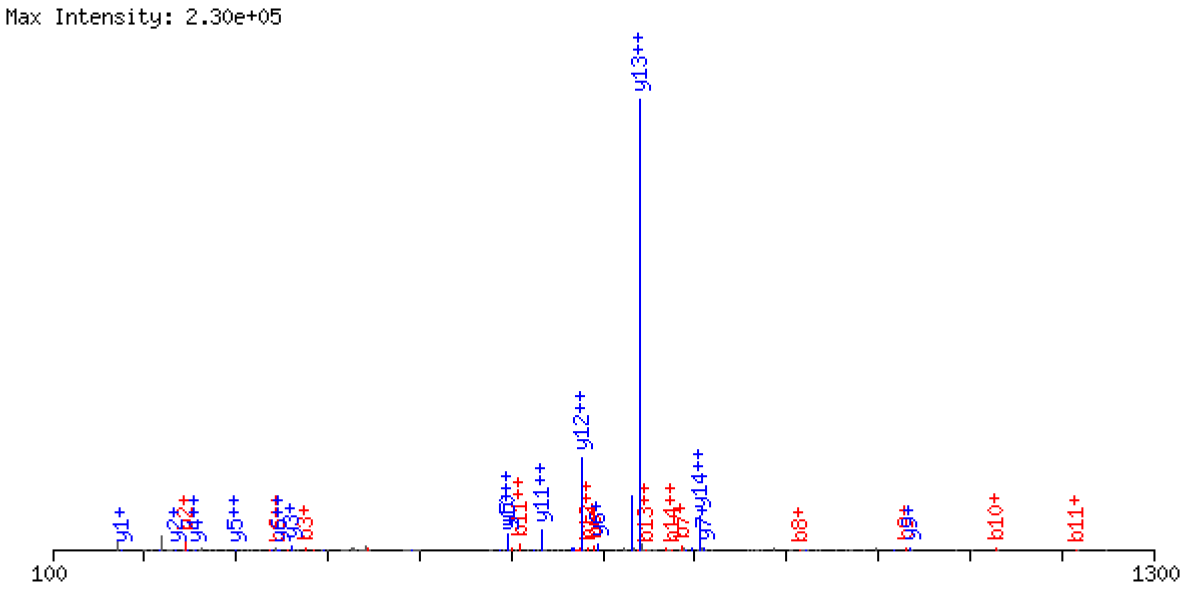

Hiding fragment losses - Click to Show

		b+	b++	y+	y++		
L	1	114.09	57.55			15	L
M	2	245.13	123.07	1608.74	804.88	14	M
E	3	374.17	187.59	1477.70	739.36	13	E
S	4	461.21	231.11	1348.66	674.83	12	S
A	5	532.24	266.63	1261.63	631.32	11	A
R	6	688.34	344.68	1190.59	595.80	10	R
P	7	785.40	393.20	1034.49	517.75	9	P
E	8	914.44	457.72	937.44	469.22	8	E
D	9	1029.47	515.24	808.39	404.70	7	D
V	10	1128.54	564.77	693.37	347.19	6	V
S	11	1215.57	608.29	594.30	297.65	5	S
F	12	1362.64	681.82	507.27	254.14	4	F
Q	13	1490.69	745.85	360.20	180.60	3	Q
G	14	1547.72	774.36	232.14	116.57	2	G
R	15			175.12	88.06	1	R

WSN NP 447-461 phospho

LMESARPEDVS(+79.97)FQGR

Charge: 2, Exp. m/z: 901.402, Calc. m/z: 901.402
Data File: B110609_010, Scans: 549 - 549

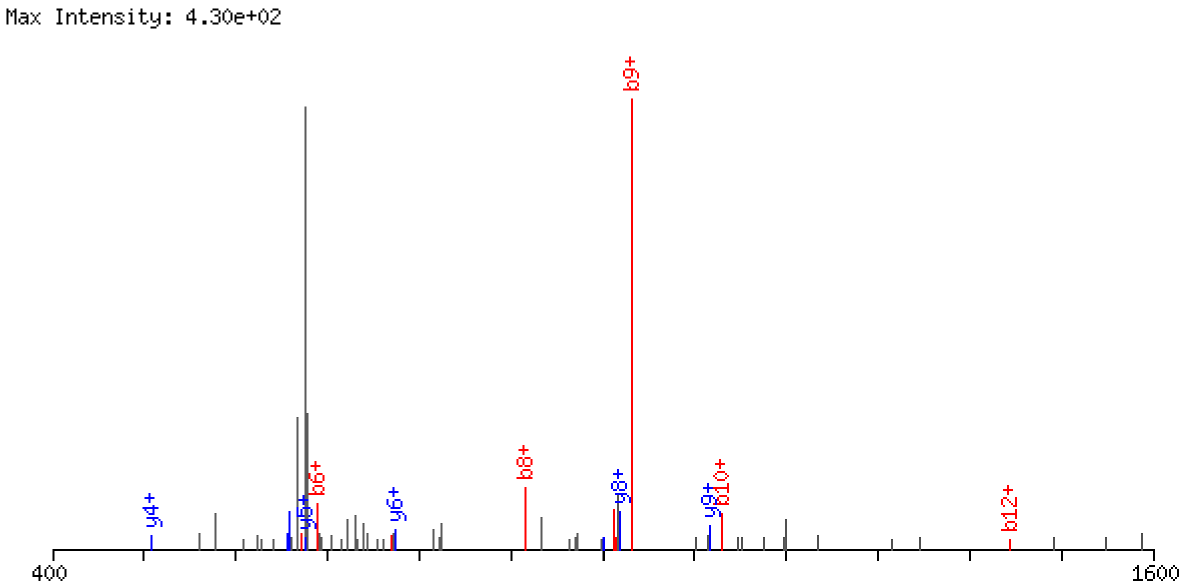

		b+	-H2O	-NH3	y+	-H2O	-NH3		
L	1	114.09						15	L
M	2	245.13			1688.71	1670.70	1671.68	14	M
E	3	374.17	356.16		1557.67	1539.66	1540.64	13	E
S	4	461.21	443.20		1428.63	1410.62	1411.60	12	S
A	5	532.24	514.23		1341.59	1323.58	1324.57	11	A
R	6	688.34	670.33	671.32	1270.56	1252.55	1253.53	10	R
P	7	785.40	767.39	768.37	1114.46	1096.45	1097.43	9	P
E	8	914.44	896.43	897.41	1017.40	999.39	1000.38	8	E
D	9	1029.47	1011.46	1012.44	888.36	870.35	871.33	7	D
V	10	1128.54	1110.52	1111.51	773.33	755.32	756.31	6	V
S	11	1295.53	1277.52	1278.51	674.27	656.26	657.24	5	S
F	12	1442.60	1424.59	1425.58	507.27		490.24	4	F
Q	13	1570.66	1552.65	1553.63	360.20		343.17	3	Q
G	14	1627.68	1609.67	1610.66	232.14		215.11	2	G
R	15				175.12		158.09	1	R

WSN NP 471-492 unmodified

ATSPIVPSFDMSNEGSYFFGDNAEEYDN

Charge: 2, Exp. m/z: 1552.140, Calc. m/z: 1552.142
Data File: B110802_015, Scans: 3349 - 3349

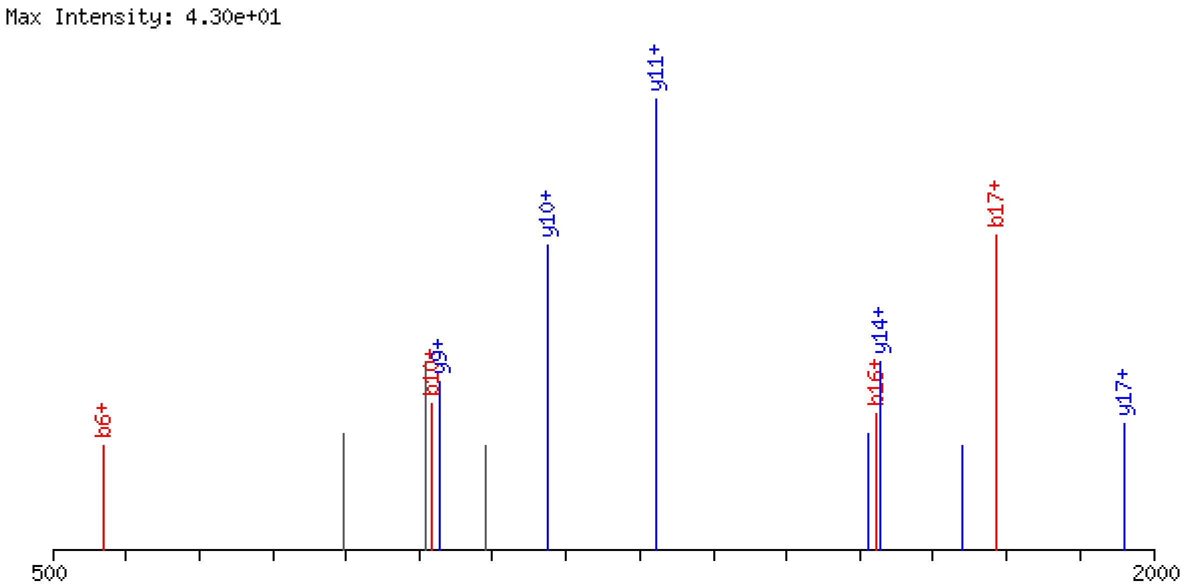

		b+	-H2O	-NH3	y+	-H2O	-NH3		
A	1	72.04						28	A
T	2	173.09	155.08		3032.24	3014.23	3015.21	27	T
S	3	260.12	242.11		2931.19	2913.18	2914.17	26	S
P	4	357.18	339.17		2844.16	2826.15	2827.14	25	P
I	5	470.26	452.25		2747.11	2729.10	2730.08	24	I
V	6	569.33	551.32		2634.02	2616.01	2617.00	23	V
P	7	666.38	648.37		2534.96	2516.95	2517.93	22	P
S	8	753.41	735.40		2437.90	2419.89	2420.88	21	S
F	9	900.48	882.47		2350.87	2332.86	2333.85	20	F
D	10	1015.51	997.50		2203.80	2185.79	2186.78	19	D
M	11	1146.55	1128.54		2088.78	2070.77	2071.75	18	M
S	12	1233.58	1215.57		1957.74	1939.73	1940.71	17	S
N	13	1347.62	1329.61	1330.60	1870.70	1852.69	1853.68	16	N
E	14	1476.67	1458.66	1459.64	1756.66	1738.65	1739.63	15	E
G	15	1533.69	1515.68	1516.66	1627.62	1609.61	1610.59	14	G
S	16	1620.72	1602.71	1603.69	1570.60	1552.59	1553.57	13	S
Y	17	1783.78	1765.77	1766.76	1483.56	1465.55	1466.54	12	Y
F	18	1930.85	1912.84	1913.83	1320.50	1302.49	1303.47	11	F
F	19	2077.92	2059.91	2060.89	1173.43	1155.42	1156.41	10	F
G	20	2134.94	2116.93	2117.92	1026.36	1008.35	1009.34	9	G
D	21	2249.97	2231.96	2232.94	969.34	951.33	952.32	8	D
N	22	2364.01	2346.00	2346.99	854.32	836.31	837.29	7	N
A	23	2435.05	2417.04	2418.02	740.27	722.26	723.25	6	A
E	24	2564.09	2546.08	2547.07	669.24	651.23	652.21	5	E
E	25	2693.13	2675.12	2676.11	540.19	522.18	523.17	4	E
Y	26	2856.20	2838.19	2839.17	411.15	393.14	394.12	3	Y
D	27	2971.23	2953.21	2954.20	248.09	230.08	231.06	2	D
N	28				133.06		116.03	1	N

WSN NP 471-492 phospho

AT(+79.97)SPIVPSFDMSNEGSYFFGDNAEEYDN

Charge: 3, Exp. m/z: 1061.760, Calc. m/z: 1061.754
Data File: B110609_010, Scans: 1418 - 1418

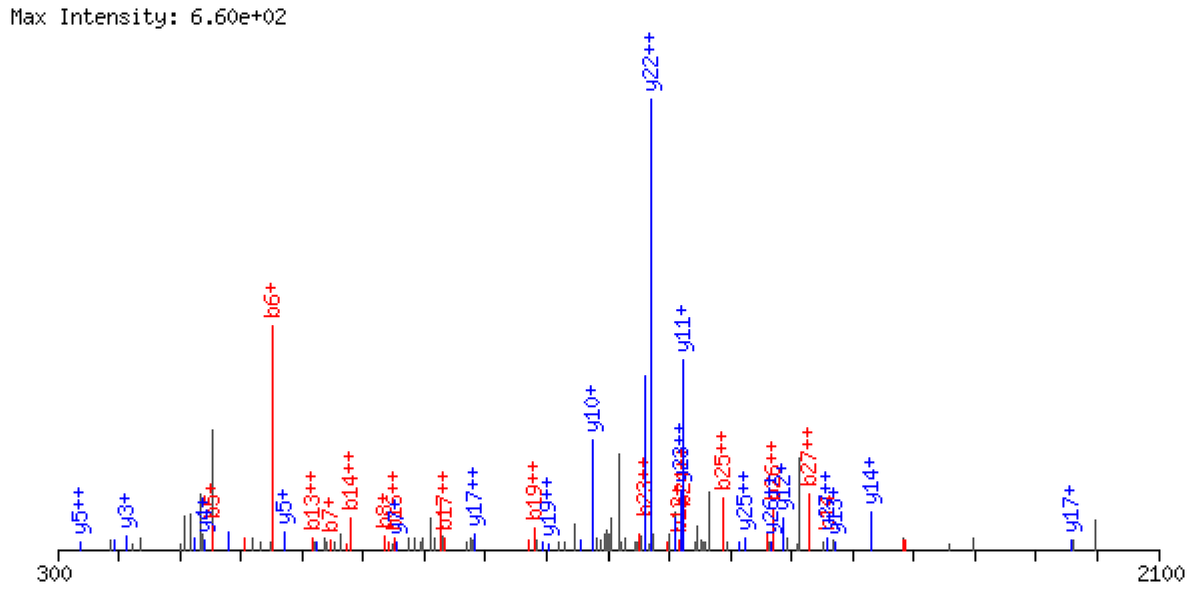

Hiding fragment losses - Click to Show

		b+	b++	y+	y++		
A	1	72.04	36.53			28	A
T	2	253.06	127.03	3112.21	1556.61	27	T
S	3	340.09	170.55	2931.19	1466.10	26	S
P	4	437.14	219.08	2844.16	1422.58	25	P
I	5	550.23	275.62	2747.11	1374.06	24	I
V	6	649.30	325.15	2634.02	1317.52	23	V
P	7	746.35	373.68	2534.96	1267.98	22	P
S	8	833.38	417.19	2437.90	1219.46	21	S
F	9	980.45	490.73	2350.87	1175.94	20	F
D	10	1095.48	548.24	2203.80	1102.41	19	D
M	11	1226.52	613.76	2088.78	1044.89	18	M
S	12	1313.55	657.28	1957.74	979.37	17	S
N	13	1427.59	714.30	1870.70	935.86	16	N
E	14	1556.63	778.82	1756.66	878.83	15	E
G	15	1613.66	807.33	1627.62	814.31	14	G
S	16	1700.69	850.85	1570.60	785.80	13	S
Y	17	1863.75	932.38	1483.56	742.29	12	Y
F	18	2010.82	1005.91	1320.50	660.75	11	F
F	19	2157.89	1079.45	1173.43	587.22	10	F
G	20	2214.91	1107.96	1026.36	513.69	9	G
D	21	2329.94	1165.47	969.34	485.18	8	D
N	22	2443.98	1222.49	854.32	427.66	7	N
A	23	2515.02	1258.01	740.27	370.64	6	A
E	24	2644.06	1322.53	669.24	335.12	5	E
E	25	2773.10	1387.05	540.19	270.60	4	E
Y	26	2936.16	1468.59	411.15	206.08	3	Y
D	27	3051.19	1526.10	248.09	124.55	2	D

		b+	b++	y+	y++		
N	28			133.06	67.03	1	N

WSN NA 158-191 carbamidomethyl

FESVAWSASAC(+57.02)HDGMGWLTIGISGPDDGAVAVLK

Charge: 3, Exp. m/z: 1168.890, Calc. m/z: 1168.891
Data File: B110818_003, Scans: 1333 - 1333

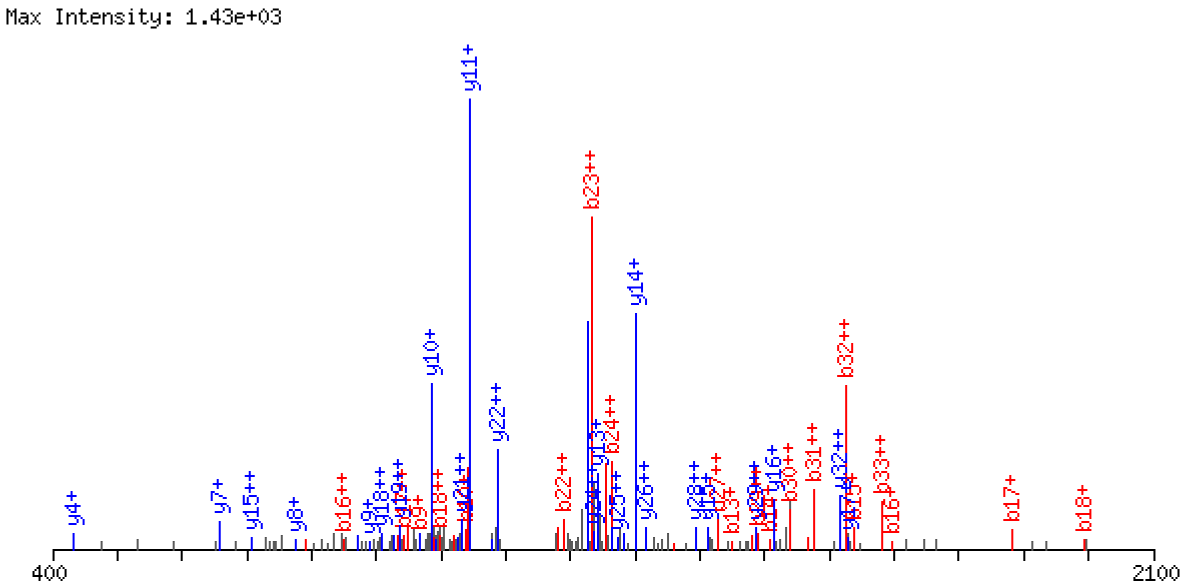

		b+	-H2O	b++	-H2O	y+	-H2O	-NH3	y++	-H2O	-NH3		
F	1	148.08		74.54								34	F
E	2	277.12	259.11	139.06	130.06	3357.59	3339.58	3340.56	1679.30	1670.29	1670.78	33	E
S	3	364.15	346.14	182.58	173.57	3228.55	3210.53	3211.52	1614.78	1605.77	1606.26	32	S
V	4	463.22	445.21	232.11	223.11	3141.51	3123.50	3124.49	1571.26	1562.25	1562.75	31	V
A	5	534.26	516.25	267.63	258.63	3042.44	3024.43	3025.42	1521.73	1512.72	1513.21	30	A
W	6	720.34	702.32	360.67	351.67	2971.41	2953.40	2954.38	1486.21	1477.20	1477.69	29	W
S	7	807.37	789.36	404.19	395.18	2785.33	2767.32	2768.30	1393.17	1384.16	1384.65	28	S
A	8	878.40	860.39	439.71	430.70	2698.30	2680.29	2681.27	1349.65	1340.65	1341.14	27	A
S	9	965.44	947.43	483.22	474.22	2627.26	2609.25	2610.23	1314.13	1305.13	1305.62	26	S
A	10	1036.47	1018.46	518.74	509.74	2540.23	2522.22	2523.20	1270.62	1261.61	1262.10	25	A
C	11	1196.50	1178.49	598.76	589.75	2469.19	2451.18	2452.16	1235.10	1226.09	1226.59	24	C
H	12	1333.56	1315.55	667.29	658.28	2309.16	2291.15	2292.13	1155.08	1146.08	1146.57	23	H
D	13	1448.59	1430.58	724.80	715.79	2172.10	2154.09	2155.07	1086.55	1077.55	1078.04	22	D
G	14	1505.61	1487.60	753.31	744.30	2057.07	2039.06	2040.05	1029.04	1020.04	1020.53	21	G
M	15	1636.65	1618.64	818.83	809.82	2000.05	1982.04	1983.03	1000.53	991.52	992.02	20	M
G	16	1693.67	1675.66	847.34	838.34	1869.01	1851.00	1851.99	935.01	926.00	926.50	19	G
W	17	1879.75	1861.74	940.38	931.37	1811.99	1793.98	1794.96	906.50	897.49	897.99	18	W
L	18	1992.84	1974.83	996.92	987.92	1625.91	1607.90	1608.88	813.46	804.45	804.95	17	L
T	19	2093.88	2075.87	1047.45	1038.44	1512.83	1494.82	1495.80	756.92	747.91	748.40	16	T
I	20	2206.97	2188.96	1103.99	1094.98	1411.78	1393.77	1394.75	706.39	697.39	697.88	15	I
G	21	2263.99	2245.98	1132.50	1123.49	1298.70	1280.68	1281.67	649.85	640.85	641.34	14	G
I	22	2377.07	2359.06	1189.04	1180.04	1241.67	1223.66	1224.65	621.34	612.34	612.83	13	I
S	23	2464.11	2446.10	1232.56	1223.55	1128.59	1110.58	1111.56	564.80	555.79	556.29	12	S
G	24	2521.13	2503.12	1261.07	1252.06	1041.56	1023.55	1024.53	521.28	512.28	512.77	11	G
P	25	2618.18	2600.17	1309.59	1300.59	984.54	966.53	967.51	492.77	483.77	484.26	10	P
D	26	2733.21	2715.20	1367.11	1358.10	887.48	869.47	870.46	444.25	435.24	435.73	9	D
D	27	2848.23	2830.22	1424.62	1415.62	772.46	754.45	755.43	386.73	377.73	378.22	8	D
G	28	2905.26	2887.25	1453.13	1444.13	657.43		640.40	329.22		320.71	7	G
A	29	2976.29	2958.28	1488.65	1479.64	600.41		583.38	300.71		292.19	6	A

		b+	-H2O	b++	-H2O	y+	-H2O	-NH3	y++	-H2O	-NH3		
V	30	3075.36	3057.35	1538.18	1529.18	529.37		512.34	265.19		256.68	5	V
A	31	3146.40	3128.39	1573.70	1564.70	430.30		413.28	215.65		207.14	4	A
V	32	3245.47	3227.46	1623.24	1614.23	359.27		342.24	180.14		171.62	3	V
L	33	3358.55	3340.54	1679.78	1670.77	260.20		243.17	130.60		122.09	2	L
K	34					147.11		130.09	74.06		65.55	1	K

WSN NA 158-191 carbamidomethyl and phosho

FES(+79.97)VAWSASAC(+57.02)HDGMGWLTIGISGPDDGAVAVLK

Charge: 3, Exp. m/z: 1195.550, Calc. m/z: 1195.544
Data File: B110818_004, Scans: 1060 - 1060

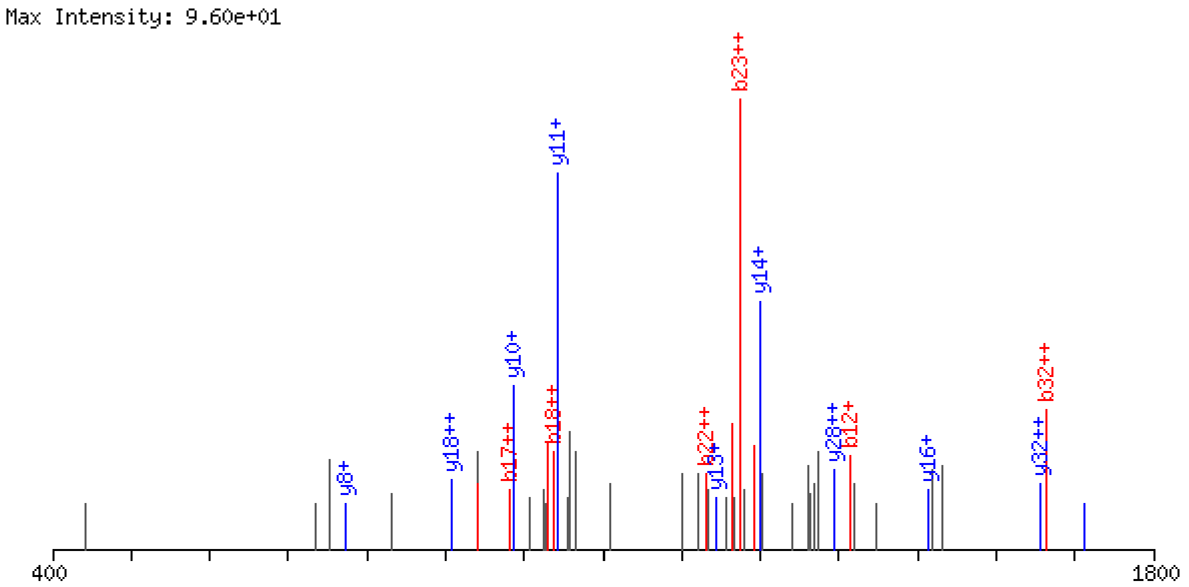

		b+	-H2O	b++	-H2O	y+	-H2O	-NH3	y++	-H2O	-NH3		
F	1	148.08		74.54								34	F
E	2	277.12	259.11	139.06	130.06	3437.55	3419.54	3420.53	1719.28	1710.28	1710.77	33	E
S	3	444.12	426.11	222.56	213.56	3308.51	3290.50	3291.49	1654.76	1645.75	1646.25	32	S
V	4	543.19	525.17	272.10	263.09	3141.51	3123.50	3124.49	1571.26	1562.25	1562.75	31	V
A	5	614.22	596.21	307.61	298.61	3042.44	3024.43	3025.42	1521.73	1512.72	1513.21	30	A
W	6	800.30	782.29	400.65	391.65	2971.41	2953.40	2954.38	1486.21	1477.20	1477.69	29	W
S	7	887.33	869.32	444.17	435.17	2785.33	2767.32	2768.30	1393.17	1384.16	1384.65	28	S
A	8	958.37	940.36	479.69	470.68	2698.30	2680.29	2681.27	1349.65	1340.65	1341.14	27	A
S	9	1045.40	1027.39	523.20	514.20	2627.26	2609.25	2610.23	1314.13	1305.13	1305.62	26	S
A	10	1116.44	1098.43	558.72	549.72	2540.23	2522.22	2523.20	1270.62	1261.61	1262.10	25	A
C	11	1276.47	1258.46	638.74	629.73	2469.19	2451.18	2452.16	1235.10	1226.09	1226.59	24	C
H	12	1413.53	1395.52	707.27	698.26	2309.16	2291.15	2292.13	1155.08	1146.08	1146.57	23	H
D	13	1528.56	1510.55	764.78	755.78	2172.10	2154.09	2155.07	1086.55	1077.55	1078.04	22	D
G	14	1585.58	1567.57	793.29	784.29	2057.07	2039.06	2040.05	1029.04	1020.04	1020.53	21	G
M	15	1716.62	1698.61	858.81	849.81	2000.05	1982.04	1983.03	1000.53	991.52	992.02	20	M
G	16	1773.64	1755.63	887.32	878.32	1869.01	1851.00	1851.99	935.01	926.00	926.50	19	G
W	17	1959.72	1941.71	980.36	971.36	1811.99	1793.98	1794.96	906.50	897.49	897.99	18	W
L	18	2072.80	2054.79	1036.91	1027.90	1625.91	1607.90	1608.88	813.46	804.45	804.95	17	L
T	19	2173.85	2155.84	1087.43	1078.42	1512.83	1494.82	1495.80	756.92	747.91	748.40	16	T
I	20	2286.93	2268.92	1143.97	1134.97	1411.78	1393.77	1394.75	706.39	697.39	697.88	15	I
G	21	2343.96	2325.95	1172.48	1163.48	1298.70	1280.68	1281.67	649.85	640.85	641.34	14	G
I	22	2457.04	2439.03	1229.02	1220.02	1241.67	1223.66	1224.65	621.34	612.34	612.83	13	I
S	23	2544.07	2526.06	1272.54	1263.53	1128.59	1110.58	1111.56	564.80	555.79	556.29	12	S
G	24	2601.09	2583.08	1301.05	1292.05	1041.56	1023.55	1024.53	521.28	512.28	512.77	11	G
P	25	2698.15	2680.14	1349.58	1340.57	984.54	966.53	967.51	492.77	483.77	484.26	10	P
D	26	2813.17	2795.16	1407.09	1398.09	887.48	869.47	870.46	444.25	435.24	435.73	9	D
D	27	2928.20	2910.19	1464.60	1455.60	772.46	754.45	755.43	386.73	377.73	378.22	8	D
G	28	2985.22	2967.21	1493.11	1484.11	657.43		640.40	329.22		320.71	7	G
A	29	3056.26	3038.25	1528.63	1519.63	600.41		583.38	300.71		292.19	6	A

		b+	-H2O	b++	-H2O	y+	-H2O	-NH3	y++	-H2O	-NH3		
V	30	3155.33	3137.32	1578.17	1569.16	529.37		512.34	265.19		256.68	5	V
A	31	3226.36	3208.35	1613.69	1604.68	430.30		413.28	215.65		207.14	4	A
V	32	3325.43	3307.42	1663.22	1654.21	359.27		342.24	180.14		171.62	3	V
L	33	3438.52	3420.51	1719.76	1710.76	260.20		243.17	130.60		122.09	2	L
K	34					147.11		130.09	74.06		65.55	1	K

WSN M1 1-21 unmodified

MSLLTEVETYVLSIVPSGPLK

Charge: 2, Exp. m/z: 1138.630, Calc. m/z: 1138.627
Data File: B110818_003, Scans: 1973 - 1973

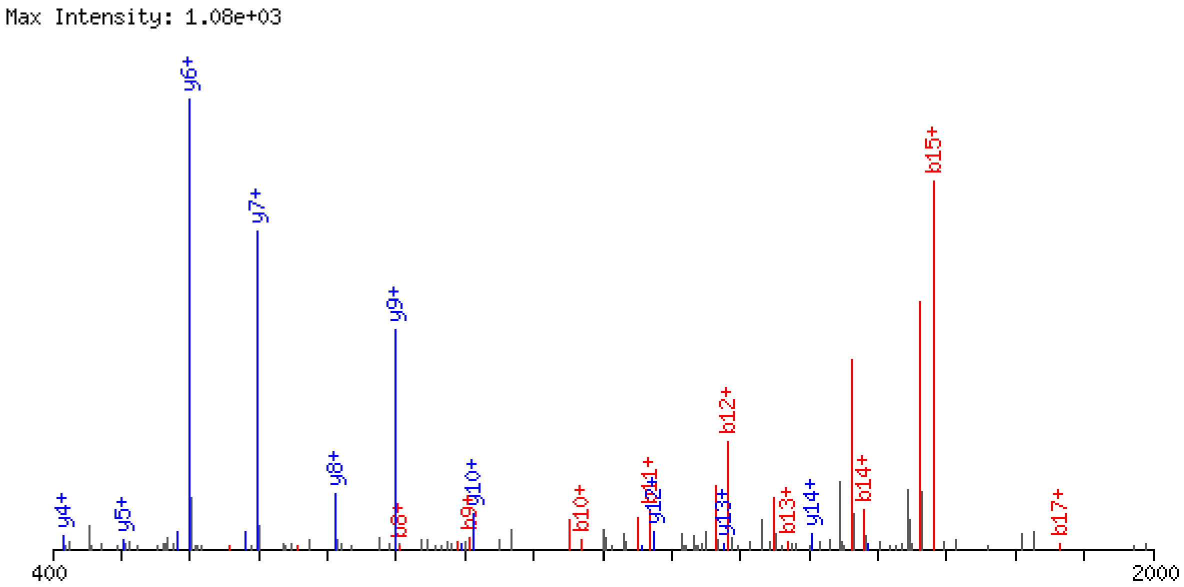

		b+	-H2O	y+	-H2O	-NH3		
M	1	132.05					21	M
S	2	219.08	201.07	2145.21	2127.19	2128.18	20	S
L	3	332.16	314.15	2058.17	2040.16	2041.15	19	L
L	4	445.25	427.24	1945.09	1927.08	1928.06	18	L
T	5	546.30	528.29	1832.01	1813.99	1814.98	17	T
E	6	675.34	657.33	1730.96	1712.95	1713.93	16	E
V	7	774.41	756.40	1601.91	1583.90	1584.89	15	V
E	8	903.45	885.44	1502.85	1484.84	1485.82	14	E
T	9	1004.50	986.49	1373.80	1355.79	1356.78	13	T
Y	10	1167.56	1149.55	1272.76	1254.75	1255.73	12	Y
V	11	1266.63	1248.62	1109.69	1091.68	1092.67	11	V
L	12	1379.71	1361.70	1010.62	992.61	993.60	10	L
S	13	1466.74	1448.73	897.54	879.53	880.51	9	S
I	14	1579.83	1561.82	810.51	792.50	793.48	8	I
V	15	1678.90	1660.89	697.42	679.41	680.40	7	V
P	16	1775.95	1757.94	598.36	580.35	581.33	6	P
S	17	1862.98	1844.97	501.30	483.29	484.28	5	S
G	18	1920.00	1901.99	414.27		397.24	4	G
P	19	2017.06	1999.05	357.25		340.22	3	P
L	20	2130.14	2112.13	260.20		243.17	2	L
K	21			147.11		130.09	1	K

WSN M1 2-21 Met Loss

SLLTEVETYVLSIVPSGPLK

Charge: 2, Exp. m/z: 1073.110, Calc. m/z: 1073.107
Data File: B110818_003, Scans: 1943 - 1943

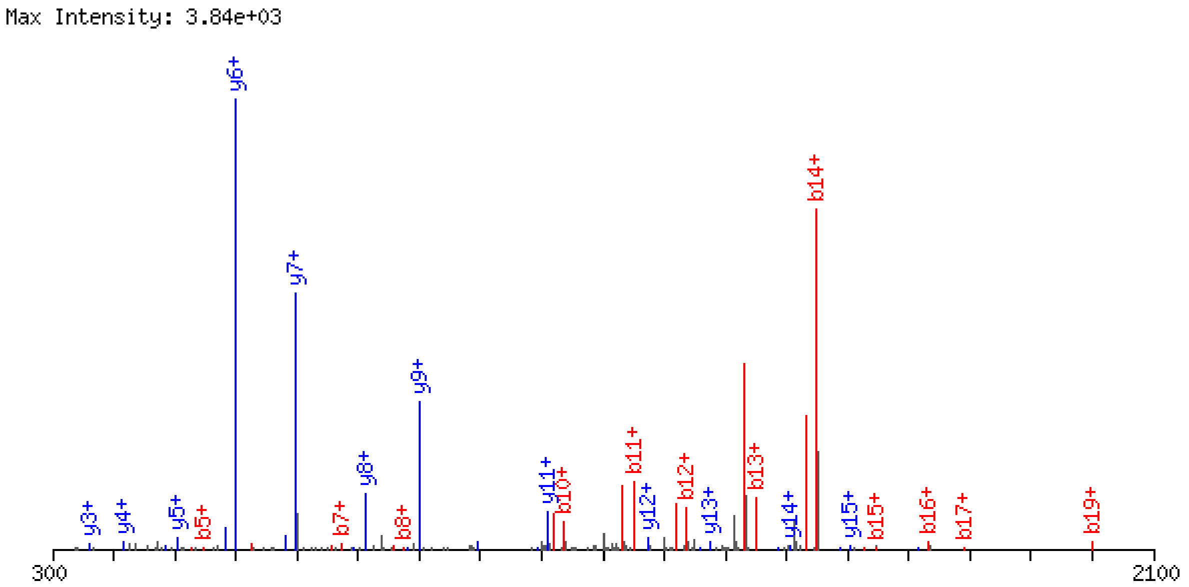

		b+	-H2O	y+	-H2O	-NH3		
S	1	88.04	70.03				20	S
L	2	201.12	183.11	2058.17	2040.16	2041.15	19	L
L	3	314.21	296.20	1945.09	1927.08	1928.06	18	L
T	4	415.26	397.24	1832.01	1813.99	1814.98	17	T
E	5	544.30	526.29	1730.96	1712.95	1713.93	16	E
V	6	643.37	625.36	1601.91	1583.90	1584.89	15	V
E	7	772.41	754.40	1502.85	1484.84	1485.82	14	E
T	8	873.46	855.45	1373.80	1355.79	1356.78	13	T
Y	9	1036.52	1018.51	1272.76	1254.75	1255.73	12	Y
V	10	1135.59	1117.58	1109.69	1091.68	1092.67	11	V
L	11	1248.67	1230.66	1010.62	992.61	993.60	10	L
S	12	1335.70	1317.69	897.54	879.53	880.51	9	S
I	13	1448.79	1430.78	810.51	792.50	793.48	8	I
V	14	1547.86	1529.85	697.42	679.41	680.40	7	V
P	15	1644.91	1626.90	598.36	580.35	581.33	6	P
S	16	1731.94	1713.93	501.30	483.29	484.28	5	S
G	17	1788.96	1770.95	414.27		397.24	4	G
P	18	1886.02	1868.01	357.25		340.22	3	P
L	19	1999.10	1981.09	260.20		243.17	2	L
K	20			147.11		130.09	1	K

WSN M1 2-21 phospho

SLLT(+79.97)EVETYVLSIVPSGPLK

Charge: 2, Exp. m/z: 1113.090, Calc. m/z: 1113.087
Data File: B110818_013, Scans: 2321 - 2321

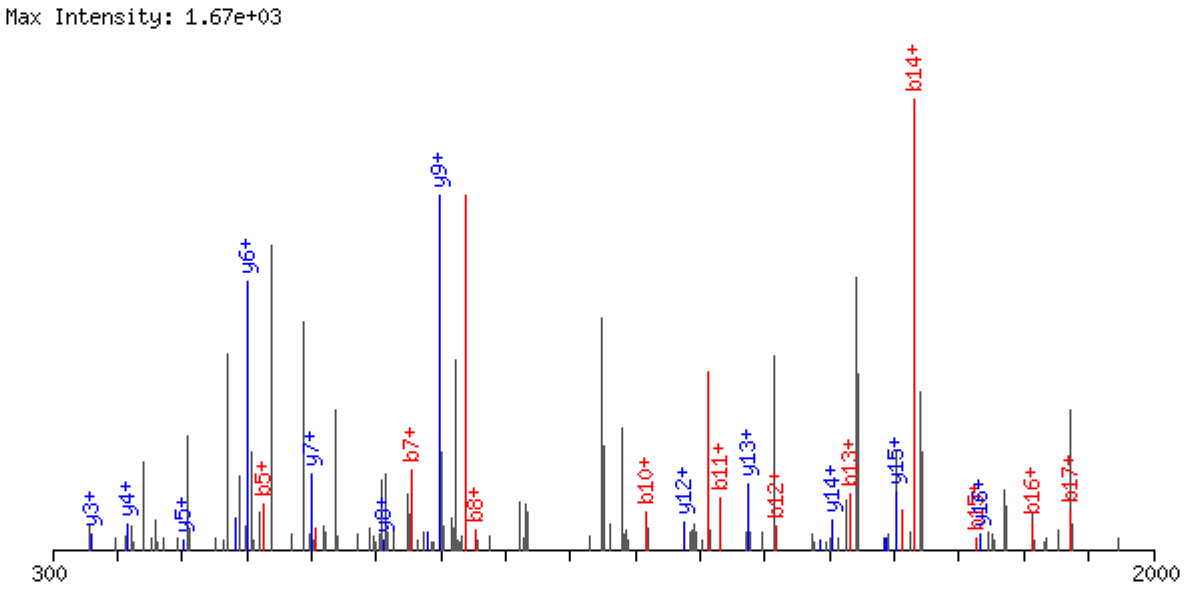

		b+	-H2O	y+	-H2O	-NH3		
S	1	88.04	70.03				20	S
L	2	201.12	183.11	2138.14	2120.13	2121.11	19	L
L	3	314.21	296.20	2025.06	2007.04	2008.03	18	L
T	4	495.22	477.21	1911.97	1893.96	1894.94	17	T
E	5	624.26	606.25	1730.96	1712.95	1713.93	16	E
V	6	723.33	705.32	1601.91	1583.90	1584.89	15	V
E	7	852.38	834.36	1502.85	1484.84	1485.82	14	E
T	8	953.42	935.41	1373.80	1355.79	1356.78	13	T
Y	9	1116.49	1098.48	1272.76	1254.75	1255.73	12	Y
V	10	1215.55	1197.54	1109.69	1091.68	1092.67	11	V
L	11	1328.64	1310.63	1010.62	992.61	993.60	10	L
S	12	1415.67	1397.66	897.54	879.53	880.51	9	S
I	13	1528.75	1510.74	810.51	792.50	793.48	8	I
V	14	1627.82	1609.81	697.42	679.41	680.40	7	V
P	15	1724.88	1706.87	598.36	580.35	581.33	6	P
S	16	1811.91	1793.90	501.30	483.29	484.28	5	S
G	17	1868.93	1850.92	414.27		397.24	4	G
P	18	1965.98	1947.97	357.25		340.22	3	P
L	19	2079.07	2061.06	260.20		243.17	2	L
K	20			147.11		130.09	1	K

WSN M1 2-21 phospho

SLLTEVETY(+79.97)VLSIVPSGPLKAEIAQR

Charge: 3, Exp. m/z: 965.183, Calc. m/z: 965.181
Data File: B110818_008, Scans: 2228 - 2228

Max Intensity: 1.34e+04

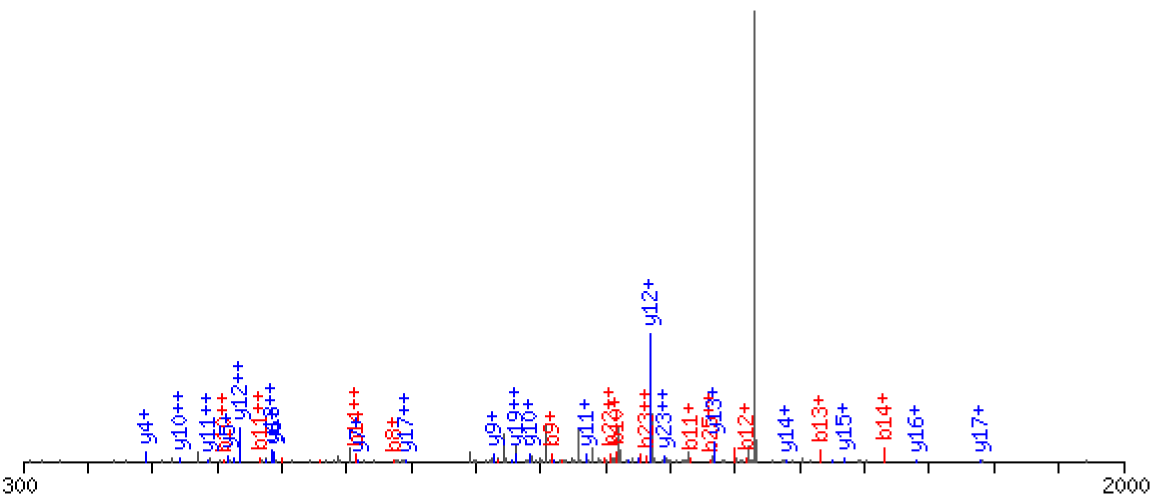

Hiding fragment losses - Click to Show

		b+	b++	y+	y++		
S	1	88.04	44.52			26	S
L	2	201.12	101.07	2806.50	1403.75	25	L
L	3	314.21	157.61	2693.42	1347.21	24	L
T	4	415.26	208.13	2580.33	1290.67	23	T
E	5	544.30	272.65	2479.28	1240.15	22	E
V	6	643.37	322.19	2350.24	1175.62	21	V
E	7	772.41	386.71	2251.17	1126.09	20	E
T	8	873.46	437.23	2122.13	1061.57	19	T
Y	9	1116.49	558.75	2021.08	1011.05	18	Y
V	10	1215.55	608.28	1778.05	889.53	17	V
L	11	1328.64	664.82	1678.99	840.00	16	L
S	12	1415.67	708.34	1565.90	783.45	15	S
I	13	1528.75	764.88	1478.87	739.94	14	I
V	14	1627.82	814.42	1365.78	683.40	13	V
P	15	1724.88	862.94	1266.72	633.86	12	P
S	16	1811.91	906.46	1169.66	585.34	11	S
G	17	1868.93	934.97	1082.63	541.82	10	G
P	18	1965.98	983.49	1025.61	513.31	9	P
L	19	2079.07	1040.04	928.56	464.78	8	L
K	20	2207.16	1104.08	815.47	408.24	7	K
A	21	2278.20	1139.60	687.38	344.19	6	A
E	22	2407.24	1204.12	616.34	308.67	5	E
I	23	2520.32	1260.67	487.30	244.15	4	I
A	24	2591.36	1296.18	374.21	187.61	3	A
Q	25	2719.42	1360.21	303.18	152.09	2	Q
R	26			175.12	88.06	1	R

WSN M1 105-113 unmodified

REITFHGAK

Charge: 3, Exp. m/z: 353.531, Calc. m/z: 353.531
Data File: B111019_043, Scans: 541 - 541

Max Intensity: 1.00e+05

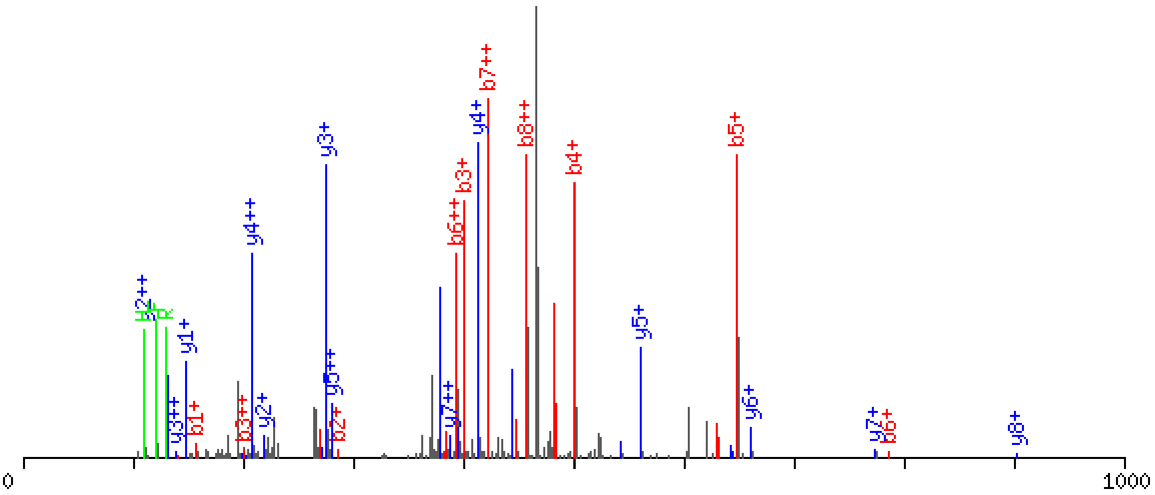

Hiding fragment losses - Click to Show

		b+	b++	y+	y++		
R	1	157.11	79.06			9	R
E	2	286.15	143.58	902.47	451.74	8	E
I	3	399.24	200.12	773.43	387.22	7	I
T	4	500.28	250.65	660.35	330.68	6	T
F	5	647.35	324.18	559.30	280.15	5	F
H	6	784.41	392.71	412.23	206.62	4	H
G	7	841.43	421.22	275.17	138.09	3	G
A	8	912.47	456.74	218.15	109.58	2	A
K	9			147.11	74.06	1	K

WSN M1 pT108

Mascot Search Results

Peptide View

MS/MS Fragmentation of **EITFHGAK**
Found in **M1**, Predicted proteins of Fodor Group RG strain of A/WSN/33

Match to Query 270: 981.433948 from(491.724250,2+)
Title: Elution from: 13.180 to 13.180 period: 0 experiment: 1 cycles: 1 precIntensity: 1179405.0
FinneganScanNumber: 423 MStype: enumIsNormalMS rawFile: B110609_010.RAW
Data file file1_B110609_010.mgf

Click mouse within plot area to zoom in by factor of two about that point
Or, Plot from 100 to 1000 Da Full range
Label all possible matches ○ Label matches used for scoring ◉

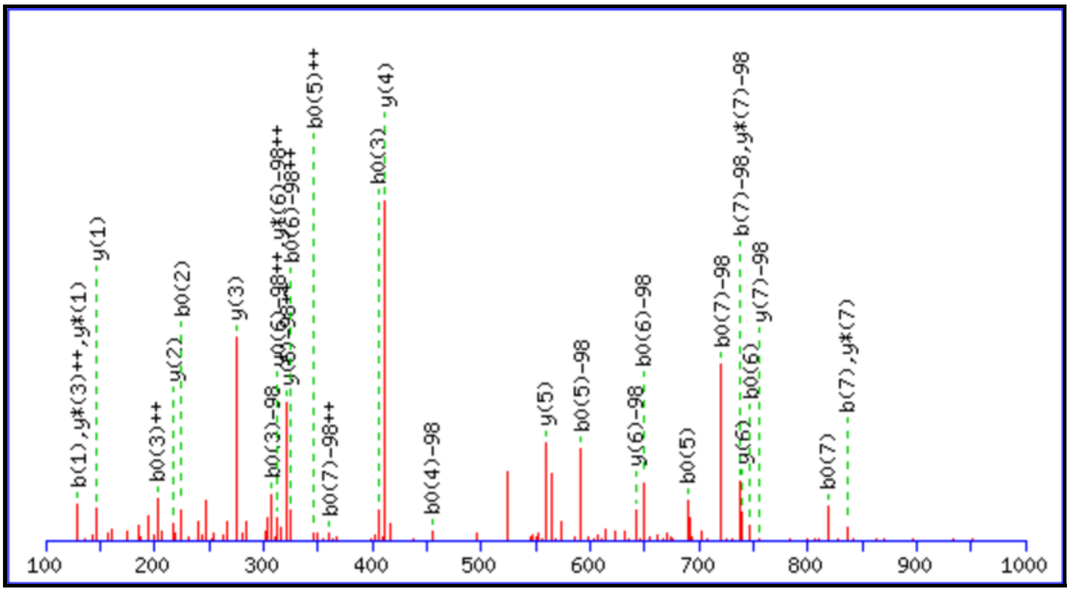

Monoisotopic mass of neutral peptide Mr(calc): 981.4321
Fixed modifications: Carbamidomethyl (C) (apply to specified residues or termini only)
Variable modifications:
T3 : Phospho (ST), with neutral losses 97.9769(shown in table), 0.0000
Ions Score: 26 **Expect:** 0.088
Matches : 32/92 fragment ions using 47 most intense peaks (help)

#	b	b^{++}	b^{0}	b^{0++}	Seq.	y	y^{++}	y*	y^{*++}	y^{0}	y^{0++}	#
1	130.0499	65.5286	112.0393	56.5233	E							8
2	243.1339	122.0706	225.1234	113.0653	I	***755.4199***	378.2136	738.3933	369.7003	737.4093	369.2083	7
3	326.1710	163.5892	308.1605	154.5839	T	***642.3358***	321.6715	625.3093	313.1583	624.3253	312.6663	6
4	473.2395	237.1234	455.2289	228.1181	F	***559.2987***	280.1530	542.2722	271.6397			5
5	610.2984	305.6528	592.2878	296.6475	H	***412.2303***	206.6188	395.2037	198.1055			4
6	667.3198	334.1636	649.3093	325.1583	G	***275.1714***	138.0893	258.1448	129.5761			3
7	738.3570	369.6821	720.3464	360.6768	A	***218.1499***	109.5786	201.1234	101.0653			2
8					K	***147.1128***	74.0600	130.0863	65.5468			1

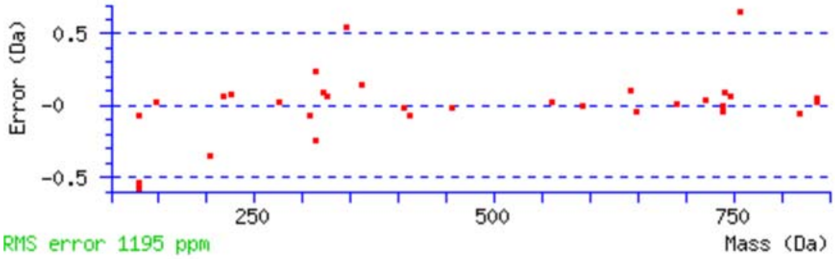

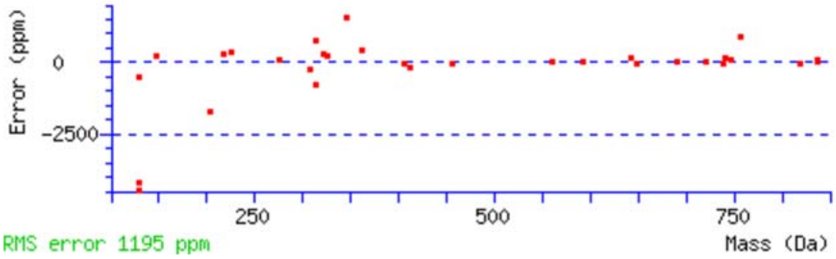

NCBI **BLAST** search of EITFHGAK
(Parameters: blastp, nr protein database, expect=20000, no filter, PAM30)
Other BLAST web gateways

All matches to this query

Score	Mr(calc):	Delta	Sequence
26.1	981.4321	0.0019	EITFHGAK
13.9	981.4168	0.0171	ELSPGSGQK
10.3	981.4532	-0.0193	QLSQLGQK
9.3	981.4532	-0.0193	QSTLPTQK
8.5	981.4532	-0.0193	QSTLPTQK
8.5	981.4532	-0.0193	QSTLPTQK
7.1	981.4420	-0.0080	LEPSDITK
6.9	981.4168	0.0171	ELSPGSGQK
6.6	981.4412	-0.0072	MNEWMKK
6.2	981.4532	-0.0193	QLSQLGQK

Mascot: http://www.matrixscience.com/

WSN M1 164-174 unmodified

QMVTTTNPLIR

Charge: 2, Exp. m/z: 637.350, Calc. m/z: 637.352
Data File: B110609_017, Scans: 1464 - 1464

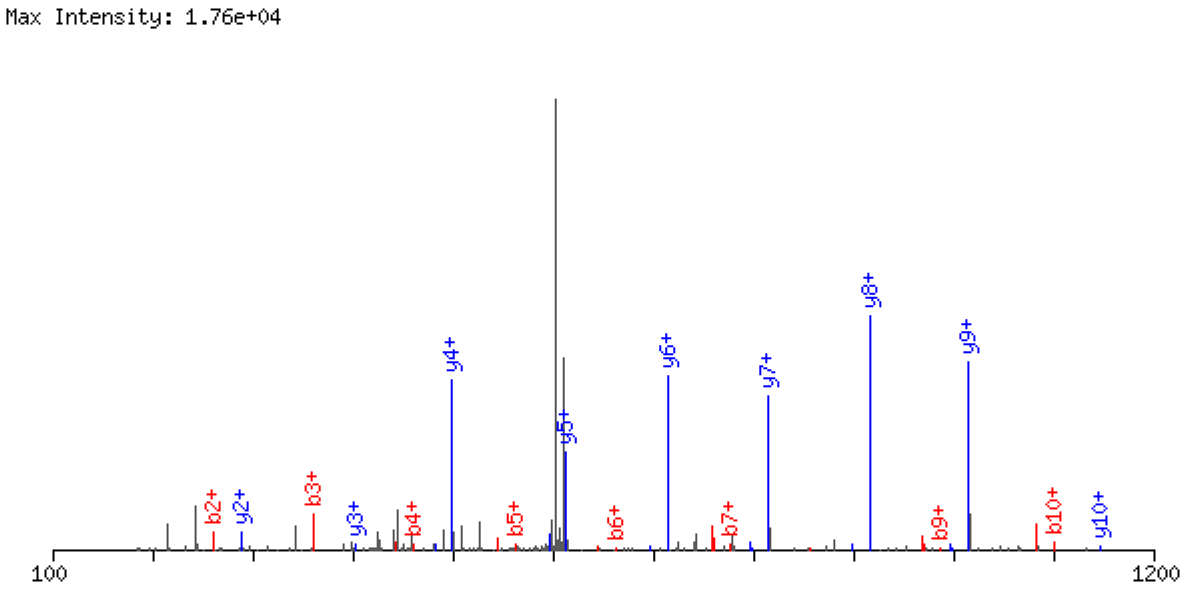

		b+	-H2O	-NH3	y+	-H2O	-NH3		
Q	1	129.07						11	Q
M	2	260.11			1145.63	1127.62	1128.61	10	M
V	3	359.17			1014.59	996.58	997.57	9	V
T	4	460.22	442.21		915.53	897.52	898.50	8	T
T	5	561.27	543.26		814.48	796.47	797.45	7	T
T	6	662.32	644.31		713.43	695.42	696.40	6	T
N	7	776.36	758.35	759.33	612.38		595.36	5	N
P	8	873.41	855.40	856.39	498.34		481.31	4	P
L	9	986.50	968.49	969.47	401.29		384.26	3	L
I	10	1099.58	1081.57	1082.56	288.20		271.18	2	I
R	11				175.12		158.09	1	R

WSN M1 164-174 phospho

QMVTTT(+79.97)NPLIR

Charge: 2, Exp. m/z: 677.334, Calc. m/z: 677.332
Data File: B110609_010, Scans: 758 - 758

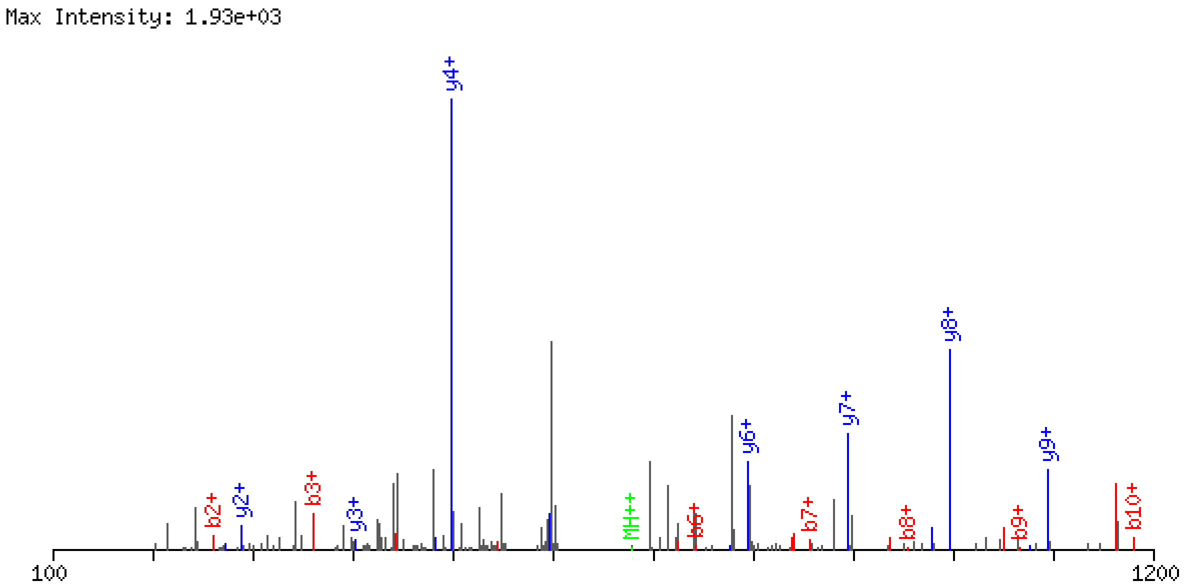

		b+	-H2O	-NH3	y+	-H2O	-NH3		
Q	1	129.07						11	Q
M	2	260.11			1225.60	1207.59	1208.57	10	M
V	3	359.17			1094.56	1076.55	1077.53	9	V
T	4	460.22	442.21		995.49	977.48	978.47	8	T
T	5	561.27	543.26		894.44	876.43	877.42	7	T
T	6	742.28	724.27		793.40	775.39	776.37	6	T
N	7	856.33	838.32	839.30	612.38		595.36	5	N
P	8	953.38	935.37	936.35	498.34		481.31	4	P
L	9	1066.46	1048.45	1049.44	401.29		384.26	3	L
I	10	1179.55	1161.54	1162.52	288.20		271.18	2	I
R	11				175.12		158.09	1	R

WSN M1 188-210 unmodified

AMEQMAGSSEQAAEAMDIASQAR

Charge: 2, Exp. m/z: 1192.020, Calc. m/z: 1192.017
Data File: B110609_017, Scans: 1435 - 1435

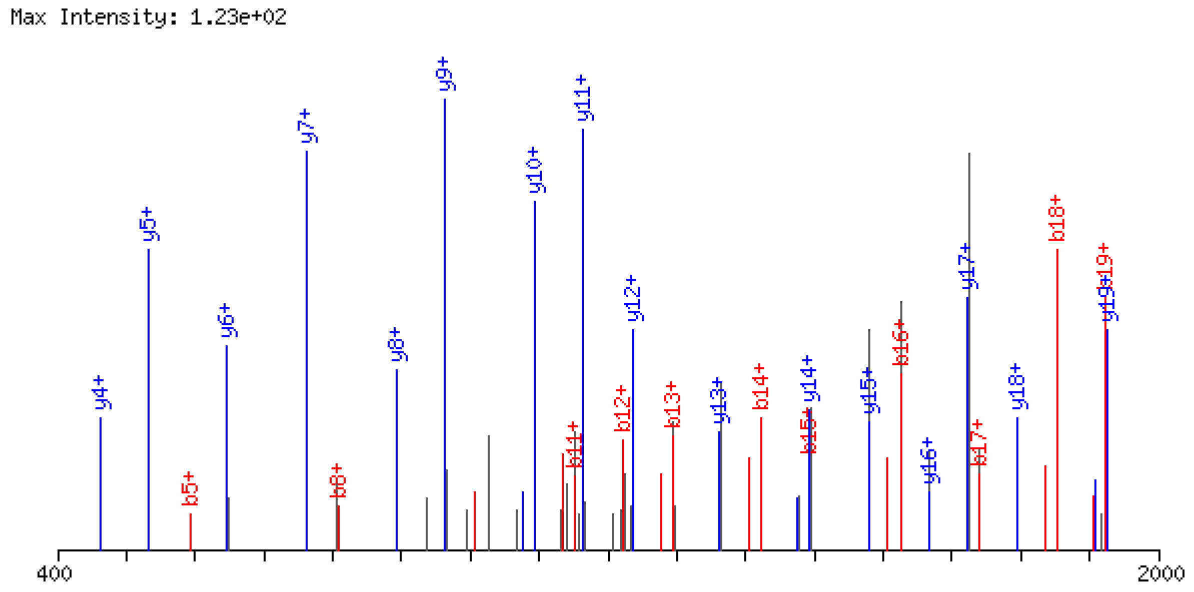

		b+	-H2O	y+	-H2O	-NH3		
A	1	72.04					23	A
M	2	203.08		2312.00	2293.98	2294.97	22	M
E	3	332.13	314.12	2180.95	2162.94	2163.93	21	E
Q	4	460.19	442.18	2051.91	2033.90	2034.89	20	Q
M	5	591.23	573.22	1923.85	1905.84	1906.83	19	M
A	6	662.26	644.25	1792.81	1774.80	1775.79	18	A
G	7	719.29	701.27	1721.78	1703.77	1704.75	17	G
S	8	806.32	788.31	1664.75	1646.74	1647.73	16	S
S	9	893.35	875.34	1577.72	1559.71	1560.70	15	S
E	10	1022.39	1004.38	1490.69	1472.68	1473.66	14	E
Q	11	1150.45	1132.44	1361.65	1343.64	1344.62	13	Q
A	12	1221.49	1203.48	1233.59	1215.58	1216.56	12	A
A	13	1292.52	1274.51	1162.55	1144.54	1145.53	11	A
E	14	1421.57	1403.56	1091.51	1073.50	1074.49	10	E
A	15	1492.60	1474.59	962.47	944.46	945.45	9	A
M	16	1623.64	1605.63	891.44	873.42	874.41	8	M
D	17	1738.67	1720.66	760.39	742.38	743.37	7	D
I	18	1851.76	1833.75	645.37	627.36	628.34	6	I
A	19	1922.79	1904.78	532.28	514.27	515.26	5	A
S	20	2009.82	1991.81	461.25	443.24	444.22	4	S
Q	21	2137.88	2119.87	374.21		357.19	3	Q
A	22	2208.92	2190.91	246.16		229.13	2	A
R	23			175.12		158.09	1	R

WSN M1 188-210 phospho

AMEQMAGS(+79.97)SEQAAEAMDIASQAR

Charge: 3, Exp. m/z: 821.672, Calc. m/z: 821.671
Data File: B110318_003, Scans: 940 - 940

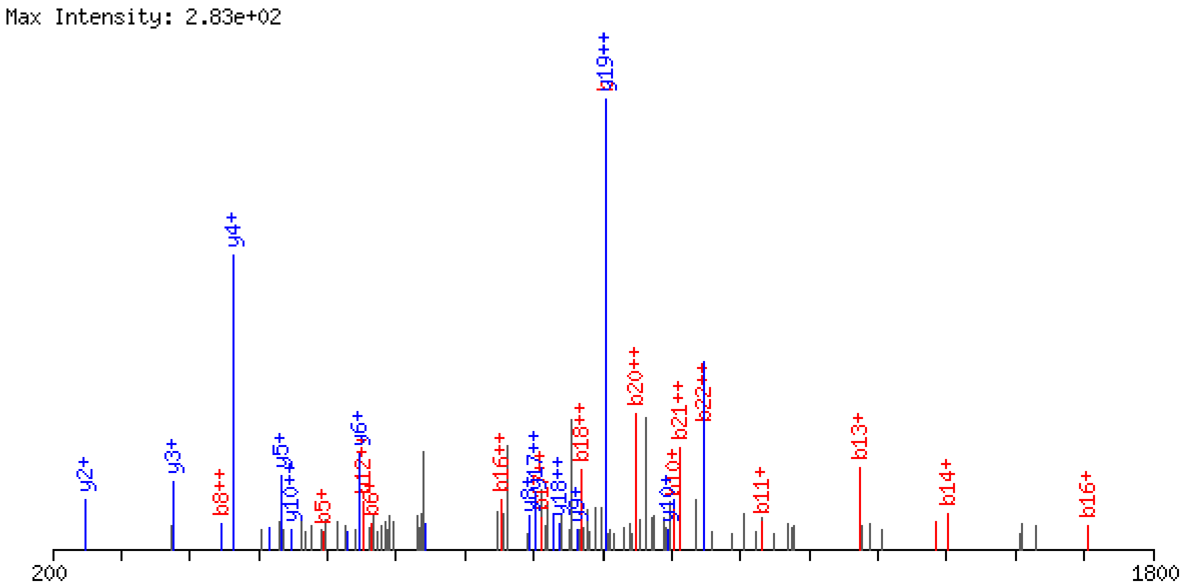

		b+	-H2O	b++	-H2O	y+	-H2O	-NH3	y++	-H2O	-NH3		
A	1	72.04		36.53								23	A
M	2	203.08		102.05		2391.96	2373.95	2374.93	1196.48	1187.48	1187.97	22	M
E	3	332.13	314.12	166.57	157.56	2260.92	2242.91	2243.89	1130.96	1121.96	1122.45	21	E
Q	4	460.19	442.18	230.60	221.59	2131.88	2113.87	2114.85	1066.44	1057.44	1057.93	20	Q
M	5	591.23	573.22	296.12	287.11	2003.82	1985.81	1986.79	1002.41	993.41	993.90	19	M
A	6	662.26	644.25	331.64	322.63	1872.78	1854.77	1855.75	936.89	927.89	928.38	18	A
G	7	719.29	701.27	360.15	351.14	1801.74	1783.73	1784.72	901.37	892.37	892.86	17	G
S	8	886.28	868.27	443.65	434.64	1744.72	1726.71	1727.69	872.86	863.86	864.35	16	S
S	9	973.32	955.30	487.16	478.16	1577.72	1559.71	1560.70	789.36	780.36	780.85	15	S
E	10	1102.36	1084.35	551.68	542.68	1490.69	1472.68	1473.66	745.85	736.84	737.34	14	E
Q	11	1230.42	1212.41	615.71	606.71	1361.65	1343.64	1344.62	681.33	672.32	672.81	13	Q
A	12	1301.45	1283.44	651.23	642.23	1233.59	1215.58	1216.56	617.30	608.29	608.78	12	A
A	13	1372.49	1354.48	686.75	677.74	1162.55	1144.54	1145.53	581.78	572.77	573.27	11	A
E	14	1501.53	1483.52	751.27	742.27	1091.51	1073.50	1074.49	546.26	537.26	537.75	10	E
A	15	1572.57	1554.56	786.79	777.78	962.47	944.46	945.45	481.74	472.73	473.23	9	A
M	16	1703.61	1685.60	852.31	843.30	891.44	873.42	874.41	446.22	437.22	437.71	8	M
D	17	1818.64	1800.63	909.82	900.82	760.39	742.38	743.37	380.70	371.70	372.19	7	D
I	18	1931.72	1913.71	966.36	957.36	645.37	627.36	628.34	323.19	314.18	314.67	6	I
A	19	2002.76	1984.75	1001.88	992.88	532.28	514.27	515.26	266.65	257.64	258.13	5	A
S	20	2089.79	2071.78	1045.40	1036.39	461.25	443.24	444.22	231.13	222.12	222.61	4	S
Q	21	2217.85	2199.84	1109.43	1100.42	374.21		357.19	187.61		179.10	3	Q
A	22	2288.89	2270.88	1144.95	1135.94	246.16		229.13	123.58		115.07	2	A
R	23					175.12		158.09	88.06		79.55	1	R

WSN M1 218-242 unmodified

TIGTHPSSSAGLKDDLLENLQAYQK

Charge: 3, Exp. m/z: 896.126, Calc. m/z: 896.127
Data File: B110818_003, Scans: 743 - 743

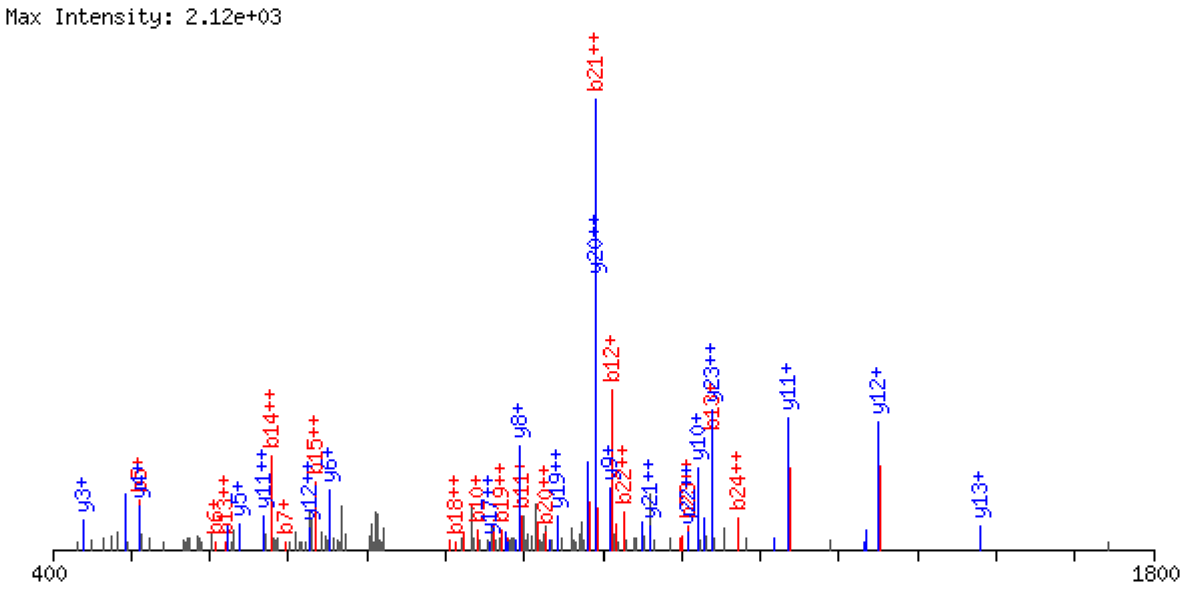

Showing fragment losses - Click to Hide

		b+	-H2O	-NH3	b++	-H2O	-NH3	y+	-H2O	-NH3	y++	-H2O	-NH3		
T	1	102.05	84.04		51.53	42.53								25	T
I	2	215.14	197.13		108.07	99.07		2585.32	2567.31	2568.29	1293.16	1284.16	1284.65	24	I
G	3	272.16	254.15		136.58	127.58		2472.24	2454.23	2455.21	1236.62	1227.62	1228.11	23	G
T	4	373.21	355.20		187.11	178.10		2415.22	2397.20	2398.19	1208.11	1199.11	1199.60	22	T
H	5	510.27	492.26		255.64	246.63		2314.17	2296.16	2297.14	1157.59	1148.58	1149.07	21	H
P	6	607.32	589.31		304.16	295.16		2177.11	2159.10	2160.08	1089.06	1080.05	1080.54	20	P
S	7	694.35	676.34		347.68	338.67		2080.06	2062.05	2063.03	1040.53	1031.53	1032.02	19	S
S	8	781.38	763.37		391.20	382.19		1993.02	1975.01	1976.00	997.02	988.01	988.50	18	S
S	9	868.42	850.41		434.71	425.71		1905.99	1887.98	1888.97	953.50	944.49	944.99	17	S
A	10	939.45	921.44		470.23	461.22		1818.96	1800.95	1801.93	909.98	900.98	901.47	16	A
G	11	996.47	978.46		498.74	489.74		1747.92	1729.91	1730.90	874.46	865.46	865.95	15	G
L	12	1109.56	1091.55		555.28	546.28		1690.90	1672.89	1673.87	845.95	836.95	837.44	14	L
K	13	1237.65	1219.64	1220.63	619.33	610.33	610.82	1577.82	1559.81	1560.79	789.41	780.41	780.90	13	K
D	14	1352.68	1334.67	1335.65	676.84	667.84	668.33	1449.72	1431.71	1432.70	725.36	716.36	716.85	12	D
D	15	1467.71	1449.70	1450.68	734.36	725.35	725.84	1334.70	1316.68	1317.67	667.85	658.85	659.34	11	D
L	16	1580.79	1562.78	1563.76	790.90	781.89	782.39	1219.67	1201.66	1202.64	610.34	601.33	601.82	10	L
L	17	1693.88	1675.86	1676.85	847.44	838.44	838.93	1106.58	1088.57	1089.56	553.80	544.79	545.28	9	L
E	18	1822.92	1804.91	1805.89	911.96	902.96	903.45	993.50	975.49	976.47	497.25	488.25	488.74	8	E
N	19	1936.96	1918.95	1919.93	968.98	959.98	960.47	864.46		847.43	432.73		424.22	7	N
L	20	2050.05	2032.03	2033.02	1025.53	1016.52	1017.01	750.41		733.39	375.71		367.20	6	L
Q	21	2178.10	2160.09	2161.08	1089.56	1080.55	1081.04	637.33		620.30	319.17		310.66	5	Q
A	22	2249.14	2231.13	2232.11	1125.07	1116.07	1116.56	509.27		492.25	255.14		246.63	4	A
Y	23	2412.20	2394.19	2395.18	1206.61	1197.60	1198.09	438.23		421.21	219.62		211.11	3	Y
Q	24	2540.26	2522.25	2523.24	1270.64	1261.63	1262.12	275.17		258.14	138.09		129.58	2	Q
K	25							147.11		130.09	74.06		65.55	1	K

WSN M1 218-242 phospho

TIGTHPSSS(+79.97)AGLKDDLLENLQAYQK

Charge: 3, Exp. m/z: 922.784, Calc. m/z: 922.784
Data File: B110609_010, Scans: 1000 - 1000

Max Intensity: 9.34e+02

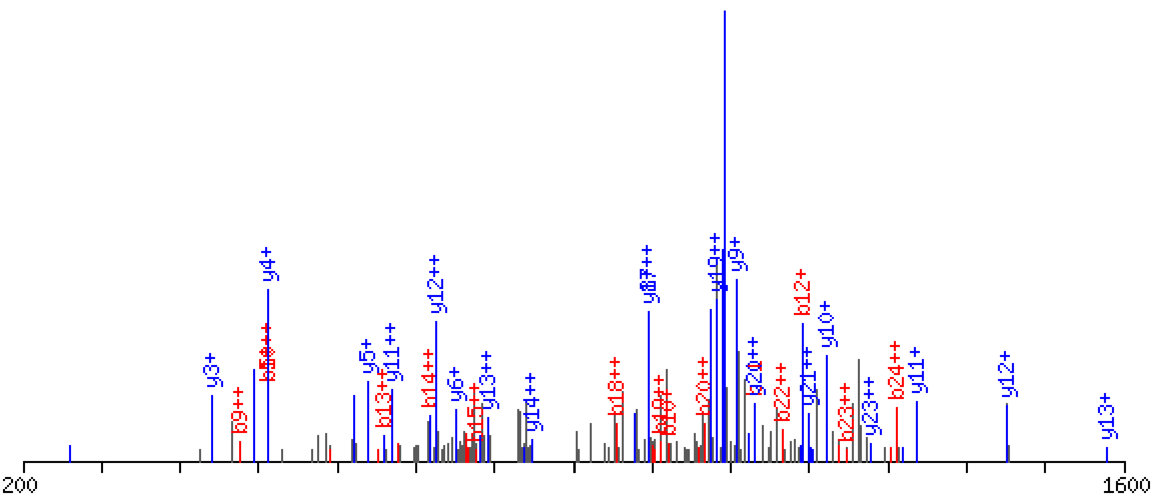

Showing fragment losses - Click to Hide

		b+	-H2O	-NH3	b++	-H2O	-NH3	y+	-H2O	-NH3	y++	-H2O	-NH3		
T	1	102.05	84.04		51.53	42.53								25	T
I	2	215.14	197.13		108.07	99.07		2665.29	2647.28	2648.26	1333.15	1324.14	1324.63	24	I
G	3	272.16	254.15		136.58	127.58		2552.20	2534.19	2535.18	1276.61	1267.60	1268.09	23	G
T	4	373.21	355.20		187.11	178.10		2495.18	2477.17	2478.15	1248.09	1239.09	1239.58	22	T
H	5	510.27	492.26		255.64	246.63		2394.13	2376.12	2377.11	1197.57	1188.57	1189.06	21	H
P	6	607.32	589.31		304.16	295.16		2257.07	2239.06	2240.05	1129.04	1120.04	1120.53	20	P
S	7	694.35	676.34		347.68	338.67		2160.02	2142.01	2143.00	1080.51	1071.51	1072.00	19	S
S	8	781.38	763.37		391.20	382.19		2072.99	2054.98	2055.96	1037.00	1027.99	1028.49	18	S
S	9	948.38	930.37		474.69	465.69		1985.96	1967.95	1968.93	993.48	984.48	984.97	17	S
A	10	1019.42	1001.41		510.21	501.21		1818.96	1800.95	1801.93	909.98	900.98	901.47	16	A
G	11	1076.44	1058.43		538.72	529.72		1747.92	1729.91	1730.90	874.46	865.46	865.95	15	G
L	12	1189.52	1171.51		595.27	586.26		1690.90	1672.89	1673.87	845.95	836.95	837.44	14	L
K	13	1317.62	1299.61	1300.59	659.31	650.31	650.80	1577.82	1559.81	1560.79	789.41	780.41	780.90	13	K
D	14	1432.65	1414.64	1415.62	716.83	707.82	708.31	1449.72	1431.71	1432.70	725.36	716.36	716.85	12	D
D	15	1547.67	1529.66	1530.65	774.34	765.34	765.83	1334.70	1316.68	1317.67	667.85	658.85	659.34	11	D
L	16	1660.76	1642.75	1643.73	830.88	821.88	822.37	1219.67	1201.66	1202.64	610.34	601.33	601.82	10	L
L	17	1773.84	1755.83	1756.82	887.42	878.42	878.91	1106.58	1088.57	1089.56	553.80	544.79	545.28	9	L
E	18	1902.88	1884.87	1885.86	951.95	942.94	943.43	993.50	975.49	976.47	497.25	488.25	488.74	8	E
N	19	2016.93	1998.92	1999.90	1008.97	999.96	1000.45	864.46		847.43	432.73		424.22	7	N
L	20	2130.01	2112.00	2112.98	1065.51	1056.50	1057.00	750.41		733.39	375.71		367.20	6	L
Q	21	2258.07	2240.06	2241.04	1129.54	1120.53	1121.03	637.33		620.30	319.17		310.66	5	Q
A	22	2329.11	2311.10	2312.08	1165.06	1156.05	1156.54	509.27		492.25	255.14		246.63	4	A
Y	23	2492.17	2474.16	2475.14	1246.59	1237.58	1238.08	438.23		421.21	219.62		211.11	3	Y
Q	24	2620.23	2602.22	2603.20	1310.62	1301.61	1302.10	275.17		258.14	138.09		129.58	2	Q
K	25							147.11		130.09	74.06		65.55	1	K

WSN M2 61-73 unmodified

RGPSTEGVPESMR

Charge: 3, Exp. m/z: 468.229, Calc. m/z: 468.231
Data File: B110818_008, Scans: 397 - 397

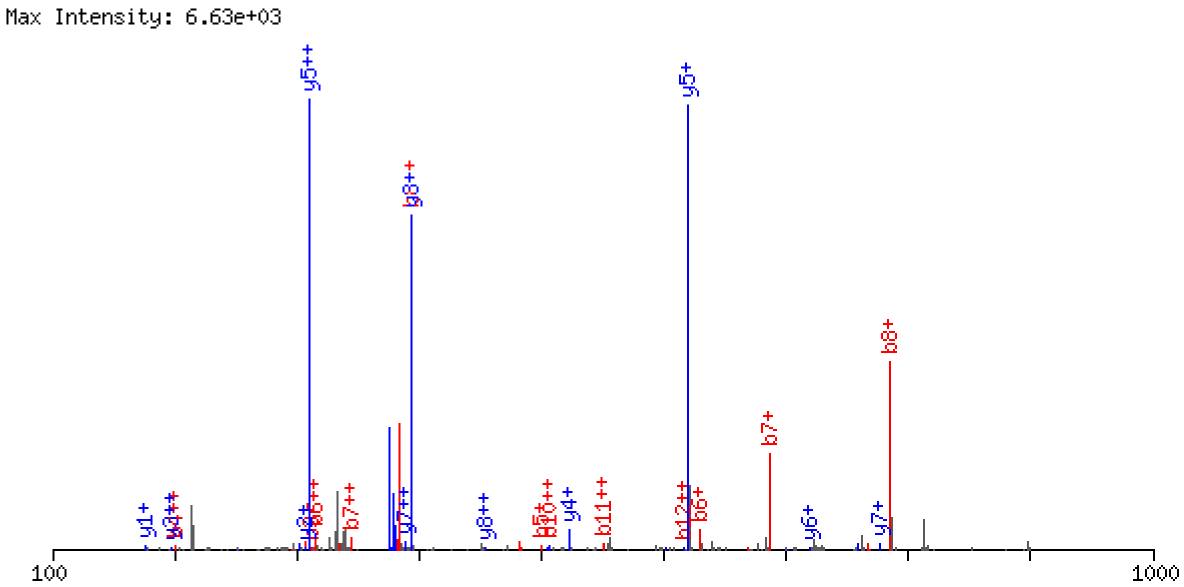

Hiding fragment losses - Click to Show

		b+	b++	y+	y++		
R	1	157.11	79.06			13	R
G	2	214.13	107.57	1246.57	623.79	12	G
P	3	311.18	156.09	1189.55	595.28	11	P
S	4	398.21	199.61	1092.50	546.75	10	S
T	5	499.26	250.13	1005.47	503.24	9	T
E	6	628.30	314.66	904.42	452.71	8	E
G	7	685.33	343.17	775.38	388.19	7	G
V	8	784.39	392.70	718.36	359.68	6	V
P	9	881.45	441.23	619.29	310.15	5	P
E	10	1010.49	505.75	522.23	261.62	4	E
S	11	1097.52	549.26	393.19	197.10	3	S
M	12	1228.56	614.78	306.16	153.58	2	M
R	13			175.12	88.06	1	R

WSN M2 61-73 phospho

RGPS(+79.97)TEGVPESMR

Charge: 2, Exp. m/z: 741.827, Calc. m/z: 741.827
Data File: B110406_003, Scans: 452 - 452

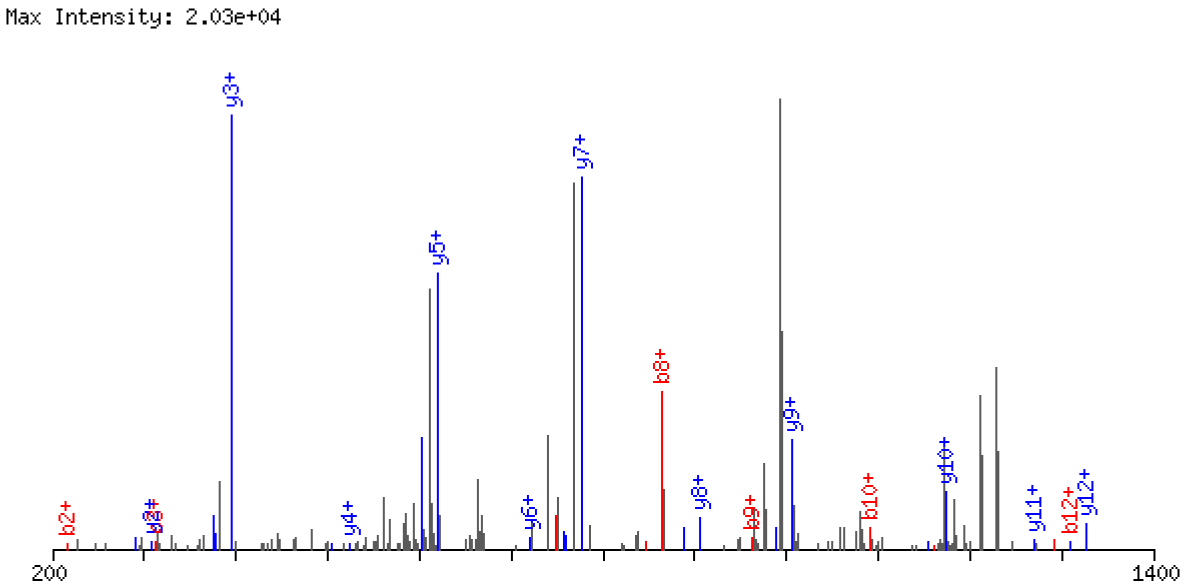

		b+	-H2O	-NH3	y+	-H2O	-NH3		
R	1	157.11		140.08				13	R
G	2	214.13		197.10	1326.54	1308.53	1309.51	12	G
P	3	311.18		294.16	1269.52	1251.51	1252.49	11	P
S	4	478.18	460.17	461.15	1172.47	1154.45	1155.44	10	S
T	5	579.23	561.22	562.20	1005.47	987.46	988.44	9	T
E	6	708.27	690.26	691.24	904.42	886.41	887.39	8	E
G	7	765.29	747.28	748.27	775.38	757.37	758.35	7	G
V	8	864.36	846.35	847.33	718.36	700.34	701.33	6	V
P	9	961.41	943.40	944.39	619.29	601.28	602.26	5	P
E	10	1090.46	1072.45	1073.43	522.23	504.22	505.21	4	E
S	11	1177.49	1159.48	1160.46	393.19	375.18	376.16	3	S
M	12	1308.53	1290.52	1291.50	306.16		289.13	2	M
R	13				175.12		158.09	1	R

WSN M2 61-77 phospho

RGPST(+79.97)EGVPESMREEYR

Charge: 3, Exp. m/z: 687.304, Calc. m/z: 687.304
Data File: B110406_003, Scans: 518 - 518

Max Intensity: 1.11e+05

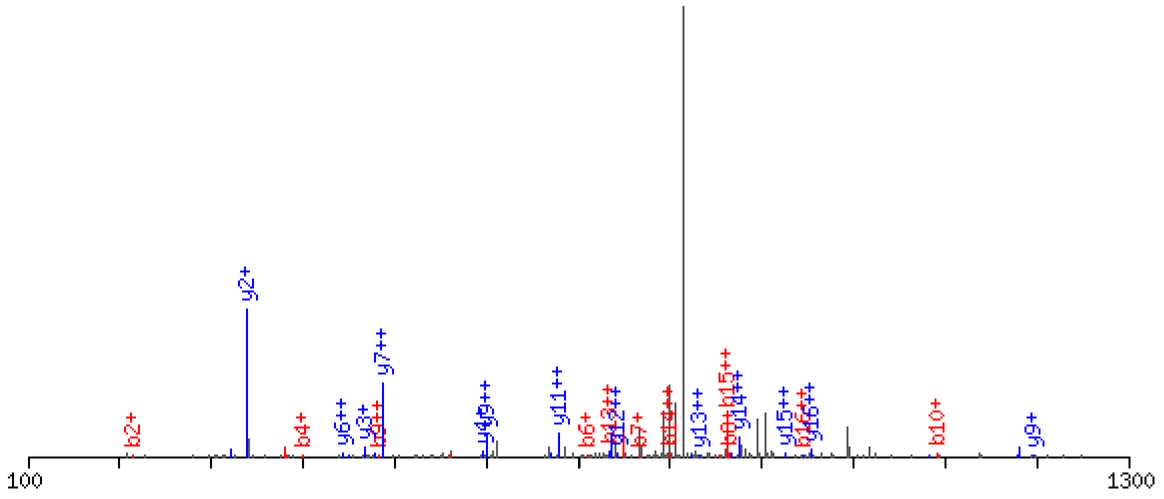

Hiding fragment losses - Click to Show

		b+	b++	y+	y++		
R	1	157.11	79.06			17	R
G	2	214.13	107.57	1903.79	952.40	16	G
P	3	311.18	156.09	1846.77	923.89	15	P
S	4	398.21	199.61	1749.71	875.36	14	S
T	5	579.23	290.12	1662.68	831.85	13	T
E	6	708.27	354.64	1481.67	741.34	12	E
G	7	765.29	383.15	1352.63	676.82	11	G
V	8	864.36	432.68	1295.60	648.31	10	V
P	9	961.41	481.21	1196.54	598.77	9	P
E	10	1090.46	545.73	1099.48	550.25	8	E
S	11	1177.49	589.25	970.44	485.72	7	S
M	12	1308.53	654.77	883.41	442.21	6	M
R	13	1464.63	732.82	752.37	376.69	5	R
E	14	1593.67	797.34	596.27	298.64	4	E
E	15	1722.72	861.86	467.22	234.12	3	E
Y	16	1885.78	943.39	338.18	169.59	2	Y
R	17			175.12	88.06	1	R

WSN M2 61-73 phospho x2

RGPS(+79.97)T(+79.97)EGVPESMR

Charge: 2, Exp. m/z: 781.808, Calc. m/z: 781.812
Data File: B110609_010, Scans: 467 - 467

Max Intensity: 5.28e+03

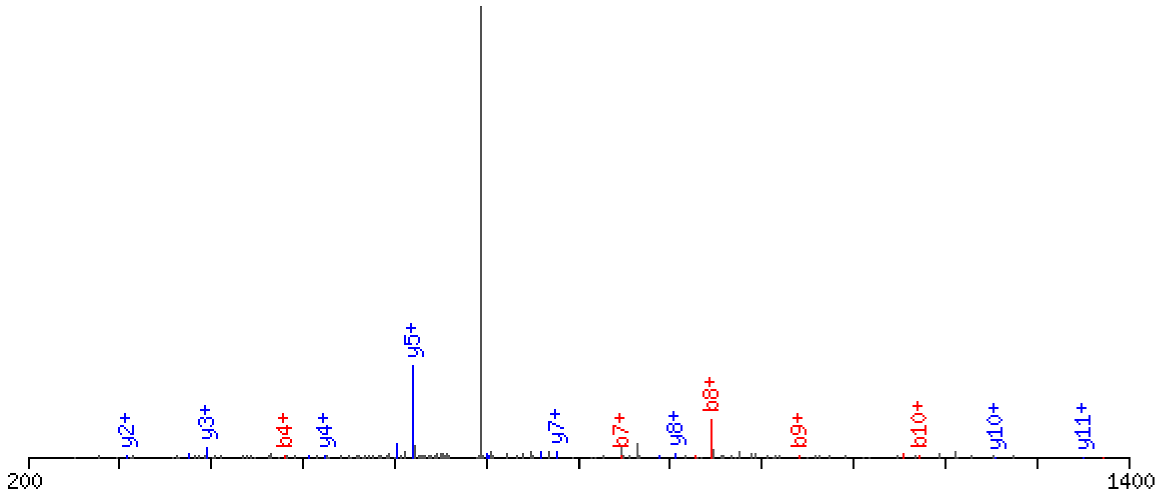

		b+	-H2O	-NH3	y+	-H2O	-NH3		
R	1	157.11		140.08				13	R
G	2	214.13		197.10	1406.51	1388.50	1389.48	12	G
P	3	311.18		294.16	1349.48	1331.47	1332.46	11	P
S	4	478.18	460.17	461.15	1252.43	1234.42	1235.41	10	S
T	5	659.19	641.18	642.17	1085.43	1067.42	1068.41	9	T
E	6	788.24	770.23	771.21	904.42	886.41	887.39	8	E
G	7	845.26	827.25	828.23	775.38	757.37	758.35	7	G
V	8	944.33	926.32	927.30	718.36	700.34	701.33	6	V
P	9	1041.38	1023.37	1024.35	619.29	601.28	602.26	5	P
E	10	1170.42	1152.41	1153.40	522.23	504.22	505.21	4	E
S	11	1257.45	1239.44	1240.43	393.19	375.18	376.16	3	S
M	12	1388.50	1370.48	1371.47	306.16		289.13	2	M
R	13				175.12		158.09	1	R

WSN NS1 25-39 unmodified

GRGSTLGLDIETATR

Charge: 3, Exp. m/z: 516.282, Calc. m/z: 516.277
Data File: B120629_004, Scans: 784 - 784

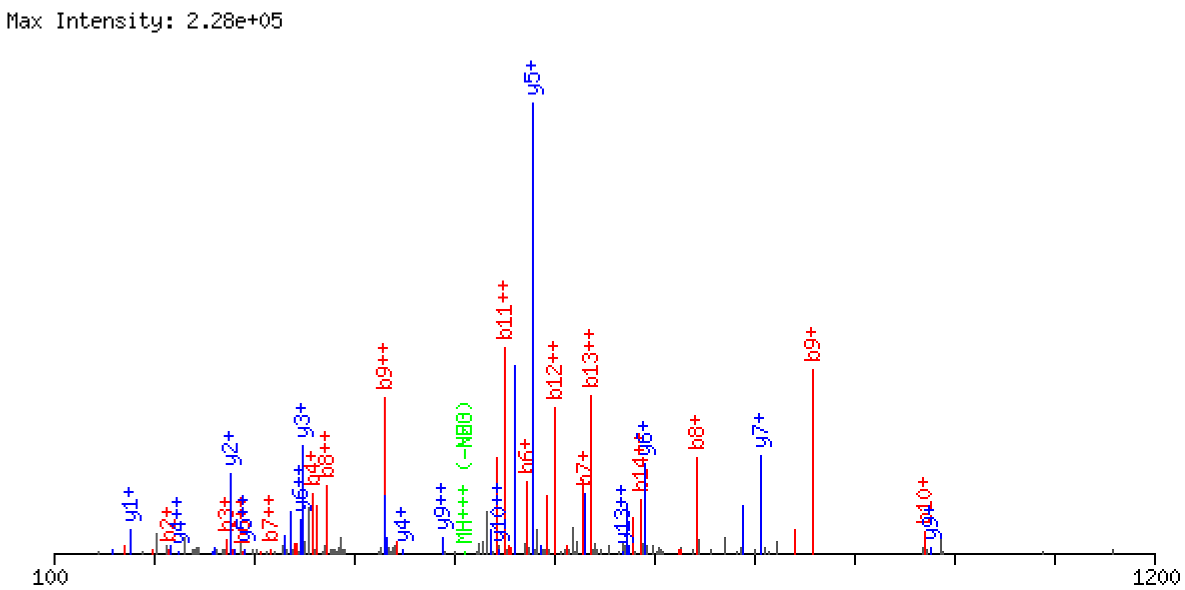

Hiding fragment losses - Click to Show

		b+	b++	y+	y++		
G	1	58.03	29.52			15	G
R	2	214.13	107.57	1489.80	745.40	14	R
G	3	271.15	136.08	1333.70	667.35	13	G
S	4	358.18	179.60	1276.67	638.84	12	S
T	5	459.23	230.12	1189.64	595.32	11	T
L	6	572.32	286.66	1088.59	544.80	10	L
G	7	629.34	315.17	975.51	488.26	9	G
L	8	742.42	371.71	918.49	459.75	8	L
D	9	857.45	429.23	805.41	403.21	7	D
I	10	970.53	485.77	690.38	345.69	6	I
E	11	1099.57	550.29	577.29	289.15	5	E
T	12	1200.62	600.81	448.25	224.63	4	T
A	13	1271.66	636.33	347.20	174.11	3	A
T	14	1372.71	686.86	276.17	138.59	2	T
R	15			175.12	88.06	1	R

WSN NS1 25-39 phospho

GRGS(+79.97)TLGLDIETATR

Charge: 2, Exp. m/z: 813.900, Calc. m/z: 813.897
Data File: B120629_004, Scans: 838 - 838

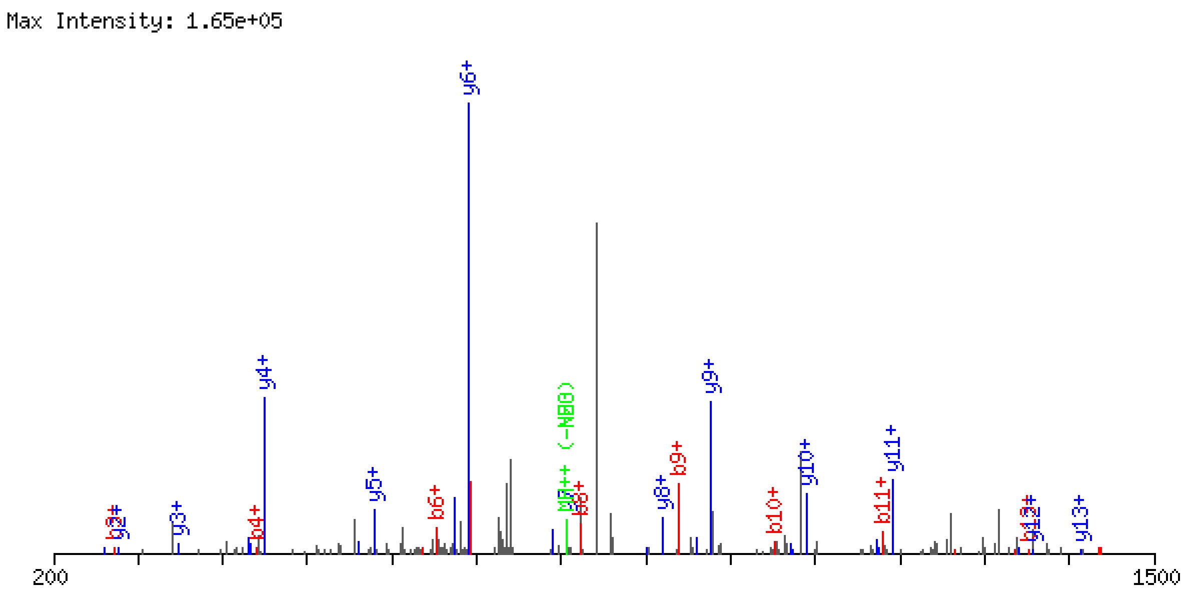

		b+	-H2O	-NH3	y+	-H2O	-NH3		
G	1	58.03						15	G
R	2	214.13		197.10	1569.76	1551.75	1552.74	14	R
G	3	271.15		254.12	1413.66	1395.65	1396.64	13	G
S	4	438.15	420.14	421.12	1356.64	1338.63	1339.61	12	S
T	5	539.20	521.19	522.17	1189.64	1171.63	1172.62	11	T
L	6	652.28	634.27	635.25	1088.59	1070.58	1071.57	10	L
G	7	709.30	691.29	692.28	975.51	957.50	958.48	9	G
L	8	822.39	804.38	805.36	918.49	900.48	901.46	8	L
D	9	937.41	919.40	920.39	805.41	787.39	788.38	7	D
I	10	1050.50	1032.49	1033.47	690.38	672.37	673.35	6	I
E	11	1179.54	1161.53	1162.51	577.29	559.28	560.27	5	E
T	12	1280.59	1262.58	1263.56	448.25	430.24	431.22	4	T
A	13	1351.63	1333.61	1334.60	347.20	329.19	330.18	3	A
T	14	1452.67	1434.66	1435.65	276.17	258.16	259.14	2	T
R	15				175.12		158.09	1	R

WSN NS1 194-200 unmodified

VSETLQR

Charge: 2, Exp. m/z: 416.730, Calc. m/z: 416.734
Data File: B110318_004, Scans: 275 - 275

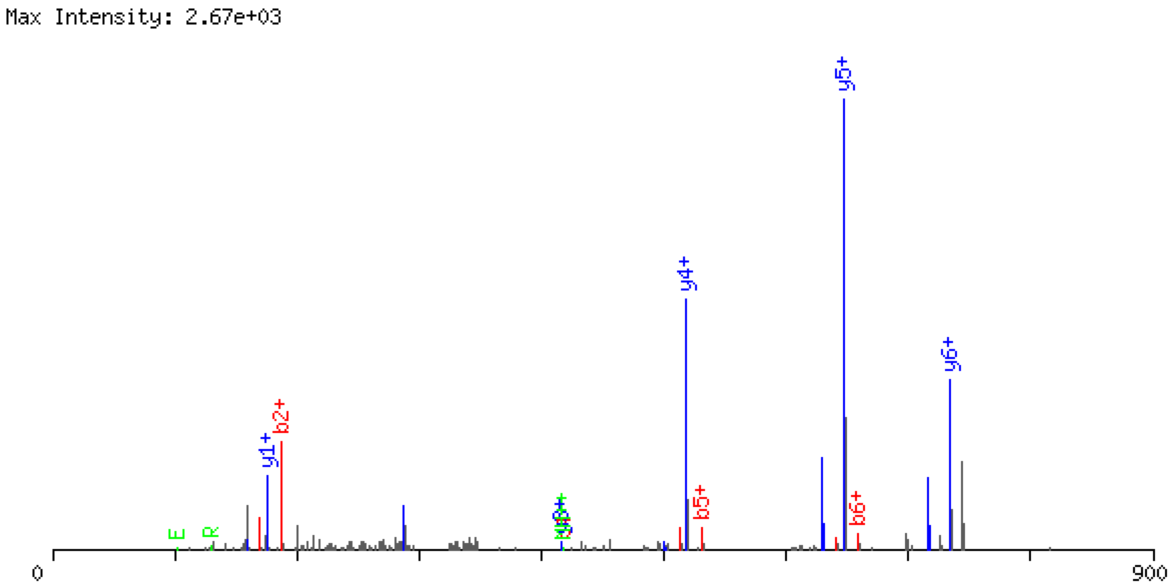

		b+	-H2O	y+	-H2O	-NH3		
V	1	100.08					7	V
S	2	187.11	169.10	733.38	715.37	716.36	6	S
E	3	316.15	298.14	646.35	628.34	629.33	5	E
T	4	417.20	399.19	517.31	499.30	500.28	4	T
L	5	530.28	512.27	416.26		399.24	3	L
Q	6	658.34	640.33	303.18		286.15	2	Q
R	7			175.12		158.09	1	R

WSN NS1 194-200 phospho

VSET(+79.97)LQR

Charge: 2, Exp. m/z: 456.714, Calc. m/z: 456.717
Data File: B110318_003, Scans: 322 - 322

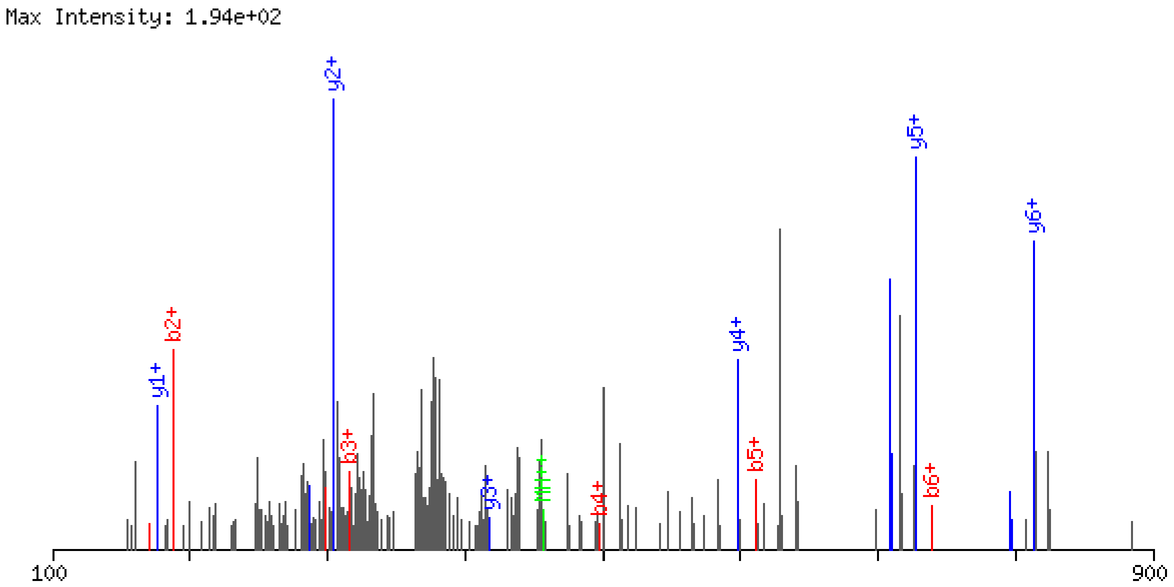

		b+	-H2O	y+	-H2O	-NH3		
V	1	100.08					7	V
S	2	187.11	169.10	813.35	795.34	796.32	6	S
E	3	316.15	298.14	726.32	708.31	709.29	5	E
T	4	497.16	479.15	597.28	579.27	580.25	4	T
L	5	610.25	592.24	416.26		399.24	3	L
Q	6	738.31	720.30	303.18		286.15	2	Q
R	7			175.12		158.09	1	R

WSN NS1 205-217 unmodified

SSNEN(+0.98)GRPPLTPK

Charge: 2, Exp. m/z: 699.355, Calc. m/z: 699.357
Data File: B120705_021, Scans: 812 - 812

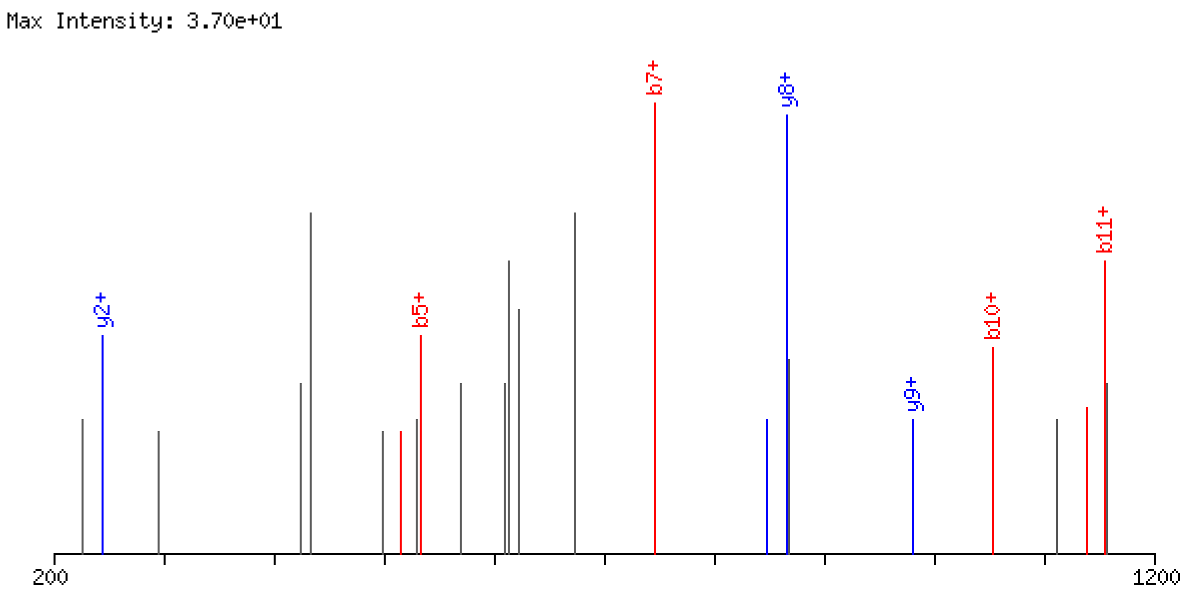

		b+	-H2O	-NH3	y+	-H2O	-NH3		
S	1	88.04	70.03					13	S
S	2	175.07	157.06		1310.67	1292.66	1293.64	12	S
N	3	289.11	271.10	272.09	1223.64	1205.63	1206.61	11	N
E	4	418.16	400.15	401.13	1109.59	1091.58	1092.57	10	E
N	5	533.18	515.17	516.16	980.55	962.54	963.53	9	N
G	6	590.21	572.19	573.18	865.53	847.51	848.50	8	G
R	7	746.31	728.30	729.28	808.50	790.49	791.48	7	R
P	8	843.36	825.35	826.33	652.40	634.39	635.38	6	P
P	9	940.41	922.40	923.39	555.35	537.34	538.32	5	P
L	10	1053.50	1035.49	1036.47	458.30	440.29	441.27	4	L
T	11	1154.54	1136.53	1137.52	345.21	327.20	328.19	3	T
P	12	1251.60	1233.59	1234.57	244.17		227.14	2	P
K	13				147.11		130.09	1	K

WSN NS1 205-217 phospho

SSNENGRPPLT(+79.97)PK

Charge: 2, Exp. m/z: 738.845, Calc. m/z: 738.847
Data File: B120705_021_120711175724, Scans: 634 - 634

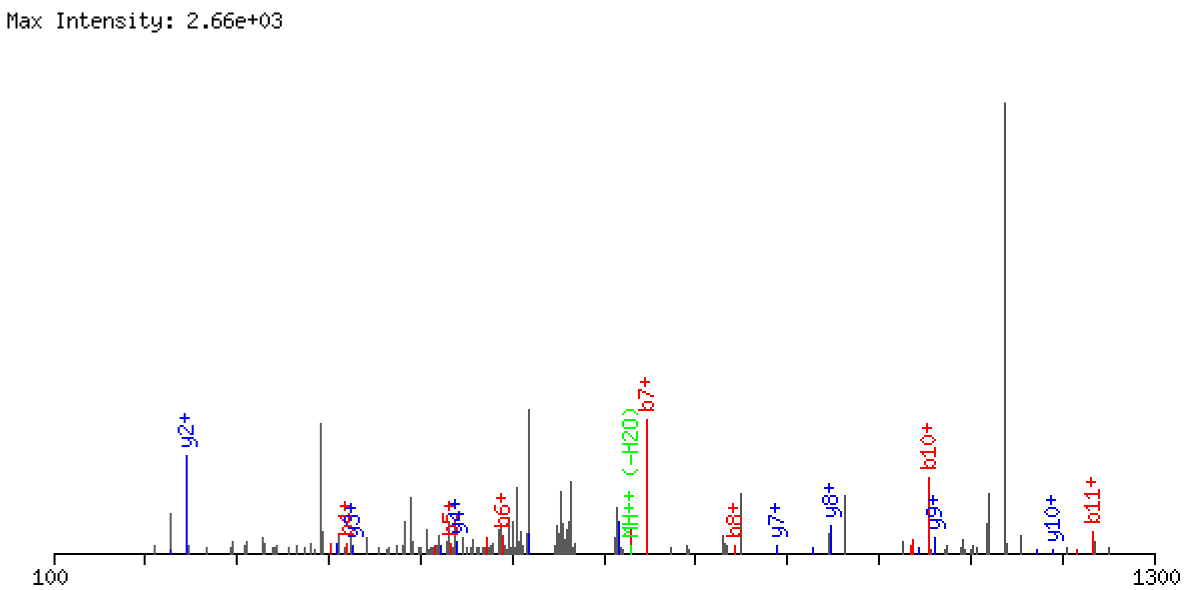

		b+	-H2O	-NH3	y+	-H2O	-NH3		
S	1	88.04	70.03					13	S
S	2	175.07	157.06		1389.65	1371.64	1372.63	12	S
N	3	289.11	271.10	272.09	1302.62	1284.61	1285.59	11	N
E	4	418.16	400.15	401.13	1188.58	1170.57	1171.55	10	E
N	5	532.20	514.19	515.17	1059.53	1041.52	1042.51	9	N
G	6	589.22	571.21	572.19	945.49	927.48	928.47	8	G
R	7	745.32	727.31	728.30	888.47	870.46	871.44	7	R
P	8	842.38	824.36	825.35	732.37	714.36	715.34	6	P
P	9	939.43	921.42	922.40	635.32	617.31	618.29	5	P
L	10	1052.51	1034.50	1035.49	538.26	520.25	521.24	4	L
T	11	1233.53	1215.52	1216.50	425.18	407.17	408.15	3	T
P	12	1330.58	1312.57	1313.55	244.17		227.14	2	P
K	13				147.11		130.09	1	K

WSN NS1 205-217 unmodified

SSNENGRPPLTPK

Charge: 2, Exp. m/z: 698.863, Calc. m/z: 698.862
Data File: B110210_012, Scans: 647 - 647

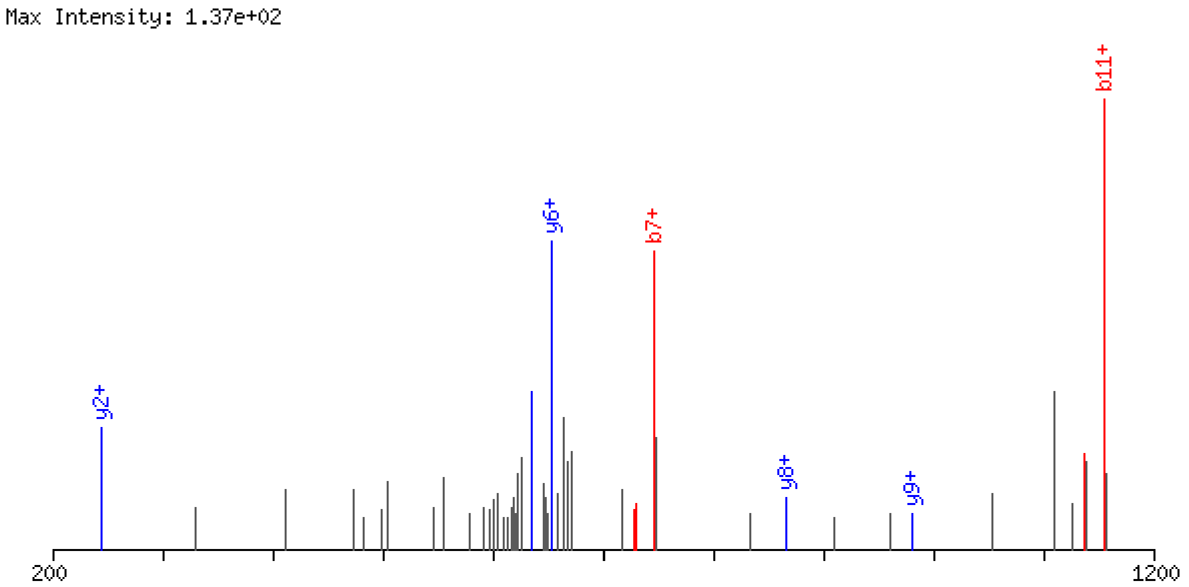

		b+	-H2O	-NH3	y+	-H2O	-NH3		
S	1	88.04	70.03					13	S
S	2	175.07	157.06		1309.69	1291.68	1292.66	12	S
N	3	289.11	271.10	272.09	1222.65	1204.64	1205.63	11	N
E	4	418.16	400.15	401.13	1108.61	1090.60	1091.58	10	E
N	5	532.20	514.19	515.17	979.57	961.56	962.54	9	N
G	6	589.22	571.21	572.19	865.53	847.51	848.50	8	G
R	7	745.32	727.31	728.30	808.50	790.49	791.48	7	R
P	8	842.38	824.36	825.35	652.40	634.39	635.38	6	P
P	9	939.43	921.42	922.40	555.35	537.34	538.32	5	P
L	10	1052.51	1034.50	1035.49	458.30	440.29	441.27	4	L
T	11	1153.56	1135.55	1136.53	345.21	327.20	328.19	3	T
P	12	1250.61	1232.60	1233.59	244.17		227.14	2	P
K	13				147.11		130.09	1	K

WSN NS1 205-217 phospho and deamidated

SSNEN(+0.98)GRPPLT(+79.97)PK

Charge: 2, Exp. m/z: 739.339, Calc. m/z: 739.337
Data File: B110318_003, Scans: 461 - 461

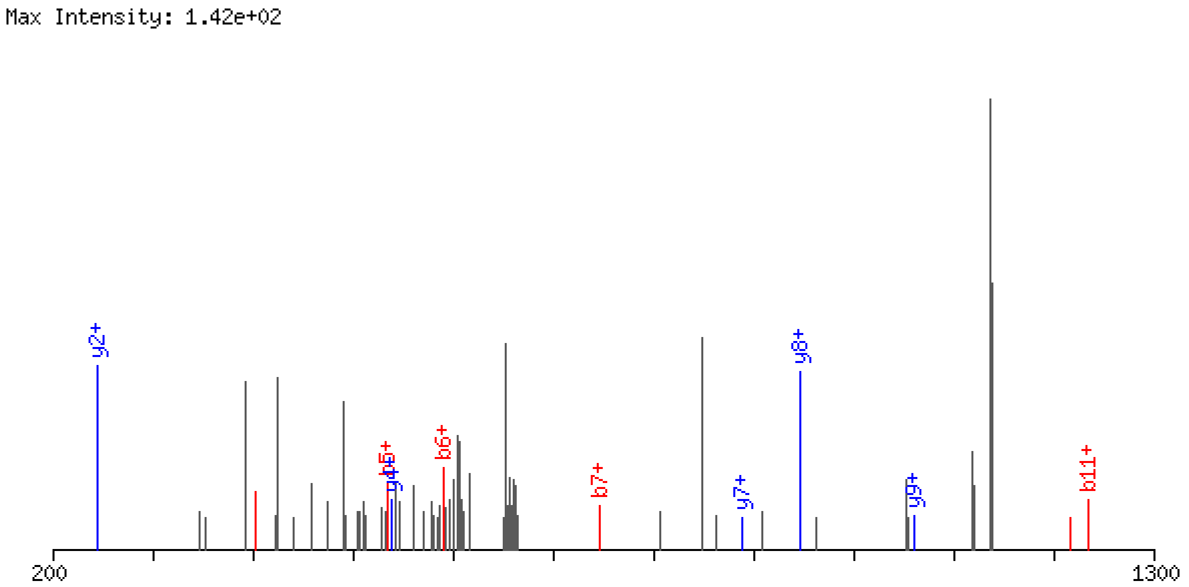

		b+	-H2O	-NH3	y+	-H2O	-NH3		
S	1	88.04	70.03					13	S
S	2	175.07	157.06		1390.64	1372.63	1373.61	12	S
N	3	289.11	271.10	272.09	1303.60	1285.59	1286.58	11	N
E	4	418.16	400.15	401.13	1189.56	1171.55	1172.53	10	E
N	5	533.18	515.17	516.16	1060.52	1042.51	1043.49	9	N
G	6	590.21	572.19	573.18	945.49	927.48	928.47	8	G
R	7	746.31	728.30	729.28	888.47	870.46	871.44	7	R
P	8	843.36	825.35	826.33	732.37	714.36	715.34	6	P
P	9	940.41	922.40	923.39	635.32	617.31	618.29	5	P
L	10	1053.50	1035.49	1036.47	538.26	520.25	521.24	4	L
T	11	1234.51	1216.50	1217.48	425.18	407.17	408.15	3	T
P	12	1331.56	1313.55	1314.54	244.17		227.14	2	P
K	13				147.11		130.09	1	K

WSN NEP 19-39 unmodified

MQLGSSSEDLNGIITQFESLK

Charge: 2, Exp. m/z: 1149.070, Calc. m/z: 1149.072
Data File: B110609_010, Scans: 1461 - 1461

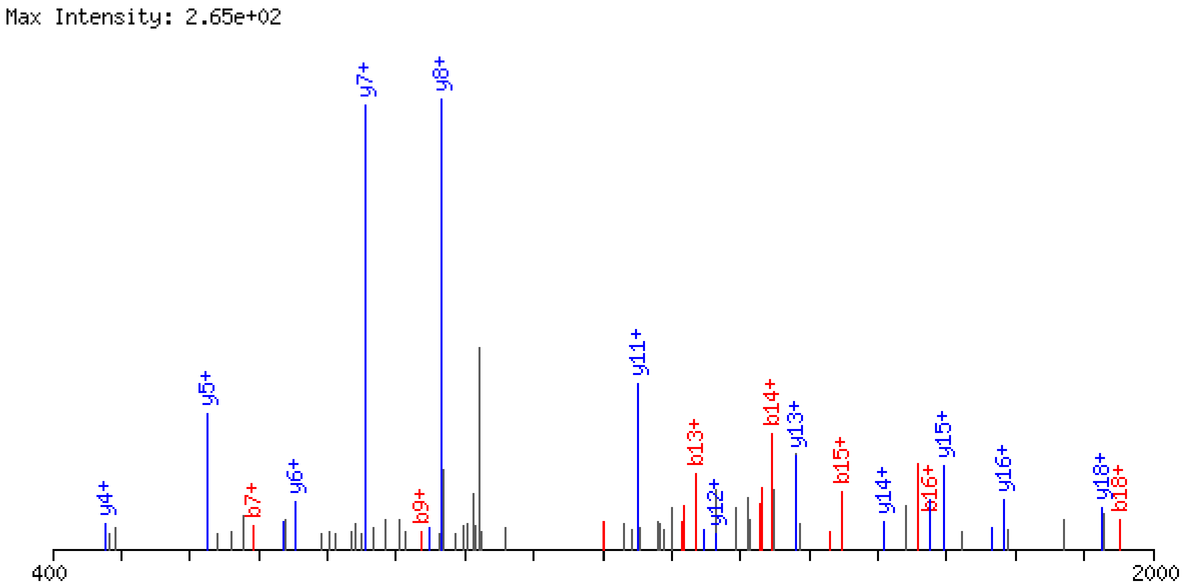

		b+	-H2O	-NH3	y+	-H2O	-NH3		
M	1	132.05						21	M
Q	2	260.11			2166.09	2148.08	2149.07	20	Q
L	3	373.19			2038.03	2020.02	2021.01	19	L
G	4	430.21			1924.95	1906.94	1907.92	18	G
S	5	517.24	499.23		1867.93	1849.92	1850.90	17	S
S	6	604.28	586.27		1780.90	1762.89	1763.87	16	S
S	7	691.31	673.30		1693.86	1675.85	1676.84	15	S
E	8	820.35	802.34		1606.83	1588.82	1589.81	14	E
D	9	935.38	917.37		1477.79	1459.78	1460.76	13	D
L	10	1048.46	1030.45		1362.76	1344.75	1345.74	12	L
N	11	1162.50	1144.49	1145.48	1249.68	1231.67	1232.65	11	N
G	12	1219.53	1201.52	1202.50	1135.64	1117.63	1118.61	10	G
I	13	1332.61	1314.60	1315.58	1078.61	1060.60	1061.59	9	I
I	14	1445.69	1427.68	1428.67	965.53	947.52	948.50	8	I
T	15	1546.74	1528.73	1529.72	852.45	834.44	835.42	7	T
Q	16	1674.80	1656.79	1657.77	751.40	733.39	734.37	6	Q
F	17	1821.87	1803.86	1804.84	623.34	605.33	606.31	5	F
E	18	1950.91	1932.90	1933.88	476.27	458.26	459.24	4	E
S	19	2037.94	2019.93	2020.92	347.23	329.22	330.20	3	S
L	20	2151.03	2133.02	2134.00	260.20		243.17	2	L
K	21				147.11		130.09	1	K

WSN NEP 19-39 phospho and deamidated

MQLGSS(+79.97)SEDLN(+0.98)GIITQFESLK

Charge: 2, Exp. m/z: 1189.550, Calc. m/z: 1189.547
Data File: B110609_010, Scans: 1359 - 1359

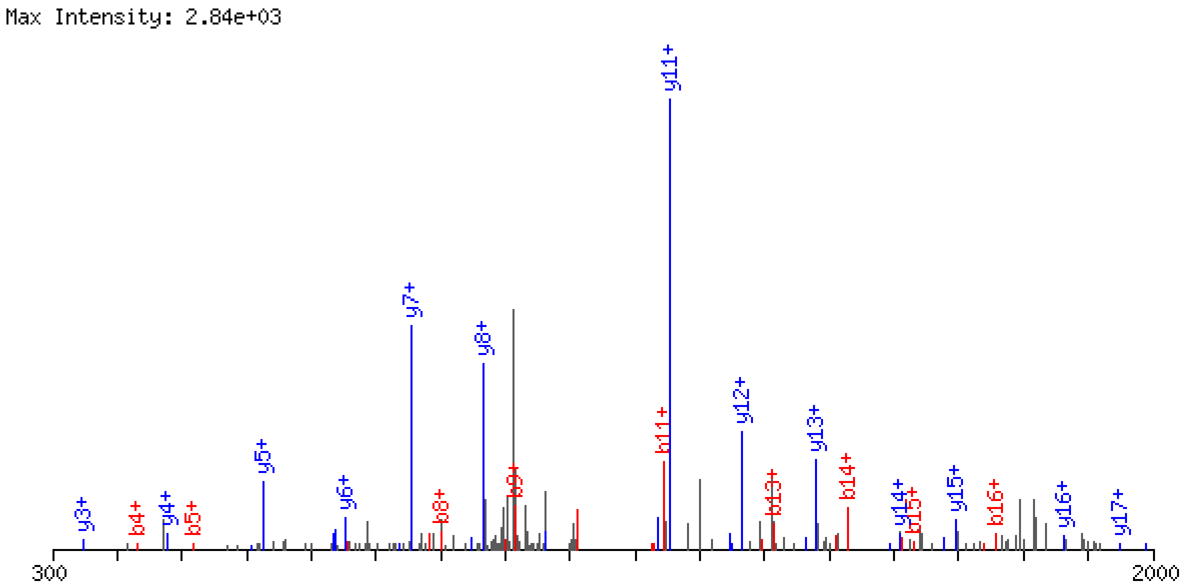

		b+	-H2O	-NH3	y+	-H2O	-NH3		
M	1	132.05						21	M
Q	2	260.11			2247.04	2229.03	2230.02	20	Q
L	3	373.19			2118.98	2100.97	2101.96	19	L
G	4	430.21			2005.90	1987.89	1988.87	18	G
S	5	517.24	499.23		1948.88	1930.87	1931.85	17	S
S	6	684.24	666.23		1861.85	1843.84	1844.82	16	S
S	7	771.27	753.26		1694.85	1676.84	1677.82	15	S
E	8	900.32	882.31		1607.82	1589.81	1590.79	14	E
D	9	1015.34	997.33		1478.77	1460.76	1461.75	13	D
L	10	1128.43	1110.42		1363.75	1345.74	1346.72	12	L
N	11	1243.45	1225.44	1226.43	1250.66	1232.65	1233.64	11	N
G	12	1300.48	1282.47	1283.45	1135.64	1117.63	1118.61	10	G
I	13	1413.56	1395.55	1396.53	1078.61	1060.60	1061.59	9	I
I	14	1526.64	1508.63	1509.62	965.53	947.52	948.50	8	I
T	15	1627.69	1609.68	1610.67	852.45	834.44	835.42	7	T
Q	16	1755.75	1737.74	1738.72	751.40	733.39	734.37	6	Q
F	17	1902.82	1884.81	1885.79	623.34	605.33	606.31	5	F
E	18	2031.86	2013.85	2014.84	476.27	458.26	459.24	4	E
S	19	2118.89	2100.88	2101.87	347.23	329.22	330.20	3	S
L	20	2231.98	2213.97	2214.95	260.20		243.17	2	L
K	21				147.11		130.09	1	K

WSN NEP 19-39 phospho

MQLGSS(+79.97)SEDLNGIITQFESLK

Charge: 2, Exp. m/z: 1189.050, Calc. m/z: 1189.052
Data File: B110609_010, Scans: 1530 - 1530

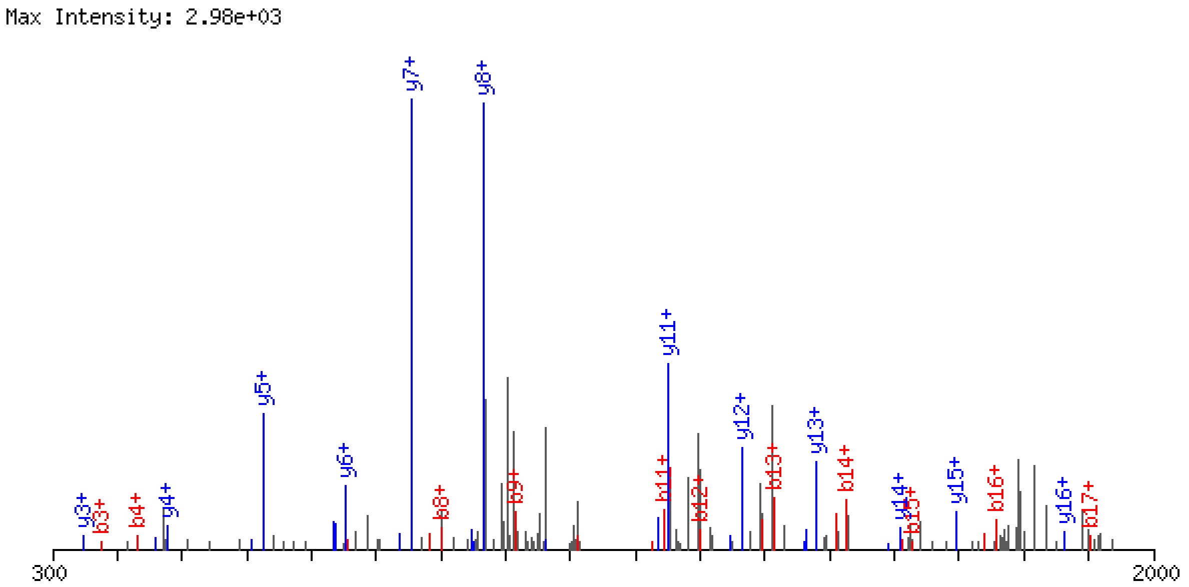

		b+	-H2O	-NH3	y+	-H2O	-NH3		
M	1	132.05						21	M
Q	2	260.11			2246.06	2228.05	2229.03	20	Q
L	3	373.19			2118.00	2099.99	2100.97	19	L
G	4	430.21			2004.92	1986.91	1987.89	18	G
S	5	517.24	499.23		1947.89	1929.88	1930.87	17	S
S	6	684.24	666.23		1860.86	1842.85	1843.84	16	S
S	7	771.27	753.26		1693.86	1675.85	1676.84	15	S
E	8	900.32	882.31		1606.83	1588.82	1589.81	14	E
D	9	1015.34	997.33		1477.79	1459.78	1460.76	13	D
L	10	1128.43	1110.42		1362.76	1344.75	1345.74	12	L
N	11	1242.47	1224.46	1225.44	1249.68	1231.67	1232.65	11	N
G	12	1299.49	1281.48	1282.47	1135.64	1117.63	1118.61	10	G
I	13	1412.58	1394.57	1395.55	1078.61	1060.60	1061.59	9	I
I	14	1525.66	1507.65	1508.63	965.53	947.52	948.50	8	I
T	15	1626.71	1608.70	1609.68	852.45	834.44	835.42	7	T
Q	16	1754.77	1736.76	1737.74	751.40	733.39	734.37	6	Q
F	17	1901.84	1883.82	1884.81	623.34	605.33	606.31	5	F
E	18	2030.88	2012.87	2013.85	476.27	458.26	459.24	4	E
S	19	2117.91	2099.90	2100.88	347.23	329.22	330.20	3	S
L	20	2230.99	2212.98	2213.97	260.20		243.17	2	L
K	21				147.11		130.09	1	K

PR8 NP 401-416 unmodified

ASAGQISIQPTFSVQR

Charge: 2, Exp. m/z: 845.451, Calc. m/z: 845.452
Data File: B120210_001_120211144847, Scans: 4422 - 4422

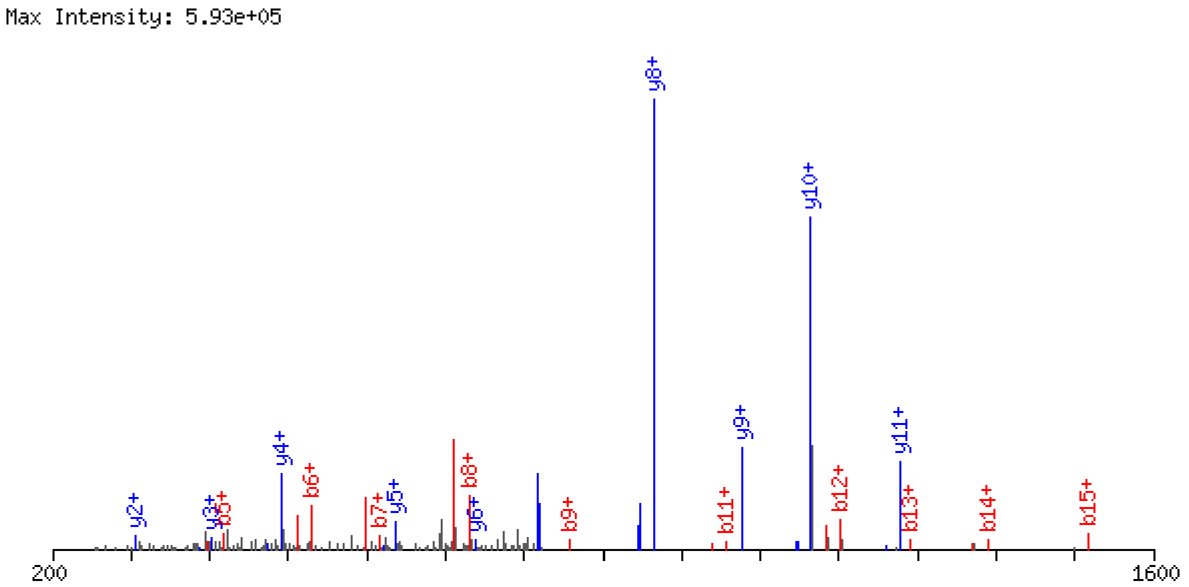

		b+	-H2O	y+	-H2O	-NH3		
A	1	72.04					16	A
S	2	159.08	141.07	1618.85	1600.84	1601.83	15	S
A	3	230.11	212.10	1531.82	1513.81	1514.80	14	A
G	4	287.13	269.12	1460.79	1442.78	1443.76	13	G
Q	5	415.19	397.18	1403.76	1385.75	1386.74	12	Q
I	6	528.28	510.27	1275.71	1257.70	1258.68	11	I
S	7	615.31	597.30	1162.62	1144.61	1145.59	10	S
I	8	728.39	710.38	1075.59	1057.58	1058.56	9	I
Q	9	856.45	838.44	962.51	944.49	945.48	8	Q
P	10	953.51	935.49	834.45	816.44	817.42	7	P
T	11	1054.55	1036.54	737.39	719.38	720.37	6	T
F	12	1201.62	1183.61	636.35	618.34	619.32	5	F
S	13	1288.65	1270.64	489.28	471.27	472.25	4	S
V	14	1387.72	1369.71	402.25		385.22	3	V
Q	15	1515.78	1497.77	303.18		286.15	2	Q
R	16			175.12		158.09	1	R

PR8 NP 401-416 phospho

AS(+79.97)AGQISIQPTFSVQR

Charge: 2, Exp. m/z: 885.432, Calc. m/z: 885.432
Data File: B120210_001_120211144847, Scans: 4748 - 4748

Max Intensity: 1.31e+04

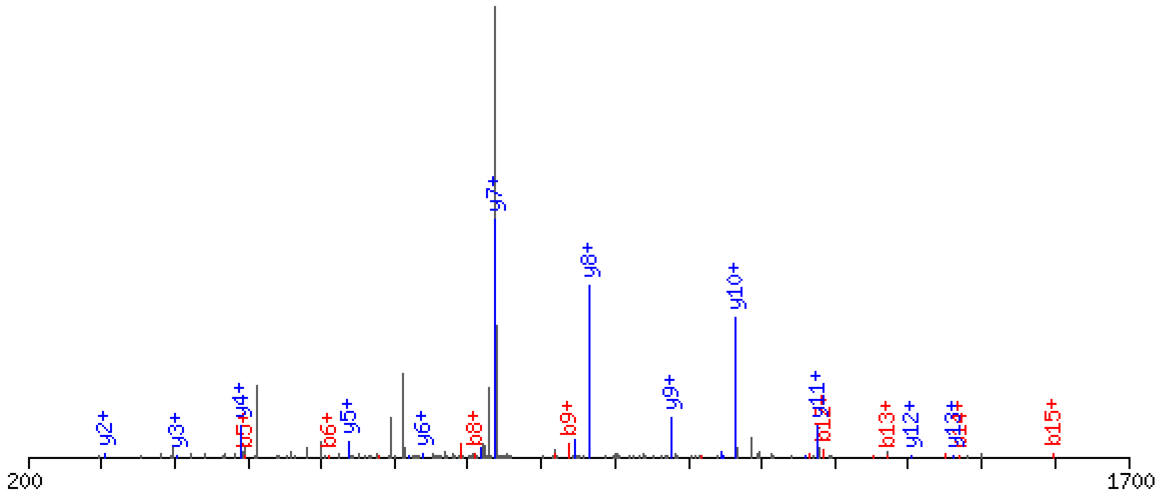

		b+	-H2O	y+	-H2O	-NH3		
A	1	72.04					16	A
S	2	239.04	221.03	1698.82	1680.81	1681.79	15	S
A	3	310.08	292.07	1531.82	1513.81	1514.80	14	A
G	4	367.10	349.09	1460.79	1442.78	1443.76	13	G
Q	5	495.16	477.15	1403.76	1385.75	1386.74	12	Q
I	6	608.24	590.23	1275.71	1257.70	1258.68	11	I
S	7	695.28	677.27	1162.62	1144.61	1145.59	10	S
I	8	808.36	790.35	1075.59	1057.58	1058.56	9	I
Q	9	936.42	918.41	962.51	944.49	945.48	8	Q
P	10	1033.47	1015.46	834.45	816.44	817.42	7	P
T	11	1134.52	1116.51	737.39	719.38	720.37	6	T
F	12	1281.59	1263.58	636.35	618.34	619.32	5	F
S	13	1368.62	1350.61	489.28	471.27	472.25	4	S
V	14	1467.69	1449.68	402.25		385.22	3	V
Q	15	1595.75	1577.74	303.18		286.15	2	Q
R	16			175.12		158.09	1	R

PR8 M1 28-47 unmodified

LEDVFAGKNTDLEVLMEWLK

Charge: 3, Exp. m/z: 784.072, Calc. m/z: 784.071
Data File: B120210_001_120211144847, Scans: 6574 - 6574

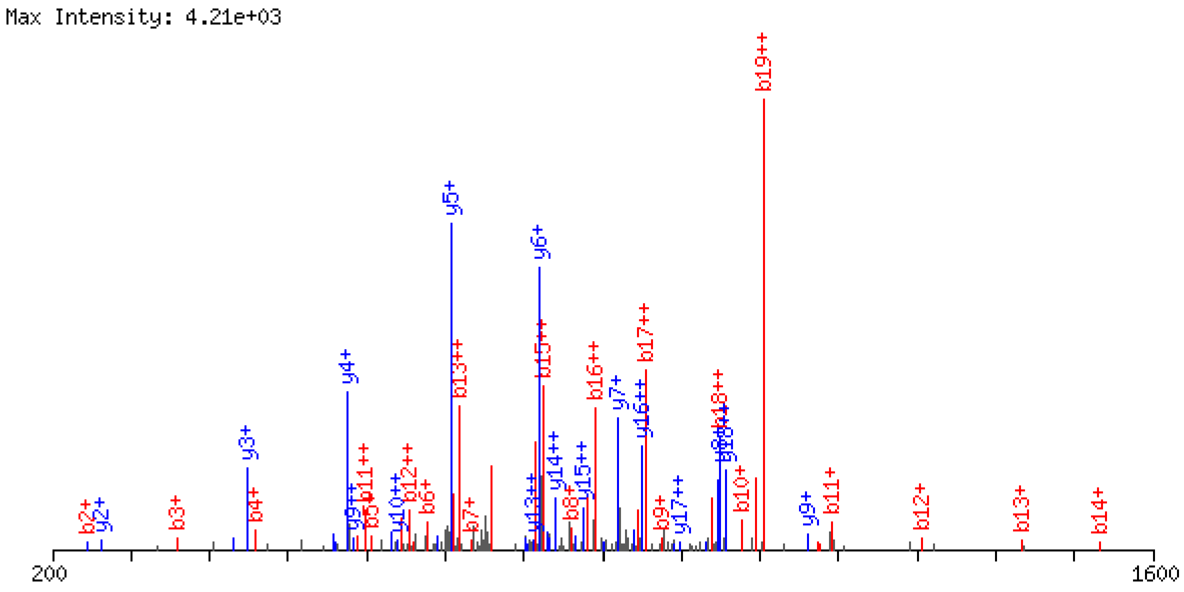

Hiding fragment losses - Click to Show

		b+	b++	y+	y++		
L	1	114.09	57.55			20	L
E	2	243.13	122.07	2237.12	1119.06	19	E
D	3	358.16	179.58	2108.07	1054.54	18	D
V	4	457.23	229.12	1993.05	997.03	17	V
F	5	604.30	302.65	1893.98	947.49	16	F
A	6	675.33	338.17	1746.91	873.96	15	A
G	7	732.36	366.68	1675.87	838.44	14	G
K	8	860.45	430.73	1618.85	809.93	13	K
N	9	974.49	487.75	1490.76	745.88	12	N
T	10	1075.54	538.27	1376.71	688.86	11	T
D	11	1190.57	595.79	1275.67	638.34	10	D
L	12	1303.65	652.33	1160.64	580.82	9	L
E	13	1432.70	716.85	1047.55	524.28	8	E
V	14	1531.76	766.39	918.51	459.76	7	V
L	15	1644.85	822.93	819.44	410.23	6	L
M	16	1775.89	888.45	706.36	353.68	5	M
E	17	1904.93	952.97	575.32	288.16	4	E
W	18	2091.01	1046.01	446.28	223.64	3	W
L	19	2204.09	1102.55	260.20	130.60	2	L
K	20			147.11	74.06	1	K

PR8 M1 28-47 phospho

LEDVFAGKNT(+79.97)DLEVLMEWLK

Charge: 3, Exp. m/z: 811.066, Calc. m/z: 810.727
Data File: B120210_001_120211144847, Scans: 6560 - 6560

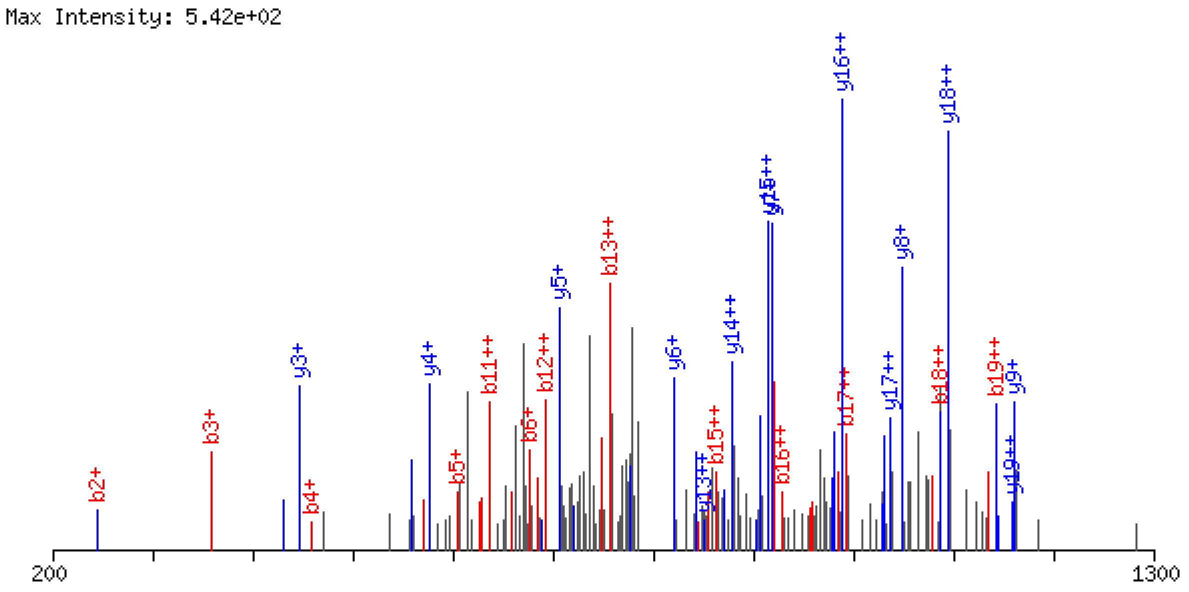

Hiding fragment losses - Click to Show

		b+	b++	y+	y++		
L	1	114.09	57.55			20	L
E	2	243.13	122.07	2317.08	1159.04	19	E
D	3	358.16	179.58	2188.04	1094.52	18	D
V	4	457.23	229.12	2073.01	1037.01	17	V
F	5	604.30	302.65	1973.94	987.48	16	F
A	6	675.33	338.17	1826.88	913.94	15	A
G	7	732.36	366.68	1755.84	878.42	14	G
K	8	860.45	430.73	1698.82	849.91	13	K
N	9	974.49	487.75	1570.72	785.86	12	N
T	10	1155.51	578.26	1456.68	728.84	11	T
D	11	1270.54	635.77	1275.67	638.34	10	D
L	12	1383.62	692.31	1160.64	580.82	9	L
E	13	1512.66	756.83	1047.55	524.28	8	E
V	14	1611.73	806.37	918.51	459.76	7	V
L	15	1724.81	862.91	819.44	410.23	6	L
M	16	1855.85	928.43	706.36	353.68	5	M
E	17	1984.90	992.95	575.32	288.16	4	E
W	18	2170.98	1085.99	446.28	223.64	3	W
L	19	2284.06	1142.53	260.20	130.60	2	L
K	20			147.11	74.06	1	K

B/Bris HA 134-151 unmodified

LSTHNVINAENAPGGPYK

Charge: 3, Exp. m/z: 627.986, Calc. m/z: 627.987
Data File: D120713_081, Scans: 12573 - 12573

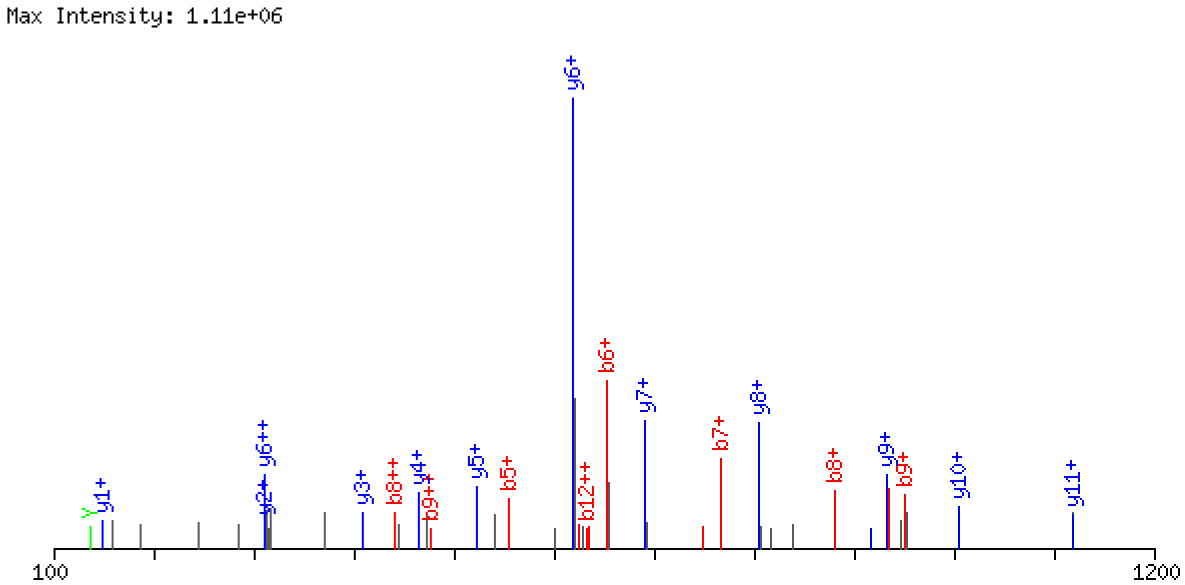

Hiding fragment losses - Click to Show

		b+	b++	y+	y++		
L	1	114.09	57.55			18	L
S	2	201.12	101.07	1768.86	884.93	17	S
T	3	302.17	151.59	1681.83	841.42	16	T
H	4	439.23	220.12	1580.78	790.89	15	H
N	5	553.27	277.14	1443.72	722.36	14	N
V	6	652.34	326.67	1329.68	665.34	13	V
I	7	765.43	383.22	1230.61	615.81	12	I
N	8	879.47	440.24	1117.53	559.27	11	N
A	9	950.51	475.76	1003.48	502.25	10	A
E	10	1079.55	540.28	932.45	466.73	9	E
N	11	1193.59	597.30	803.40	402.21	8	N
A	12	1264.63	632.82	689.36	345.18	7	A
P	13	1361.68	681.34	618.32	309.67	6	P
G	14	1418.70	709.85	521.27	261.14	5	G
G	15	1475.72	738.37	464.25	232.63	4	G
P	16	1572.78	786.89	407.23	204.12	3	P
Y	17	1735.84	868.42	310.18	155.59	2	Y
K	18			147.11	74.06	1	K

B/Bris HA 134-151 phospho

LST(+79.97)HNVINAENAPGGPYK

Charge: 3, Exp. m/z: 654.980, Calc. m/z: 654.644
Data File: D120713_081, Scans: 12834 - 12834

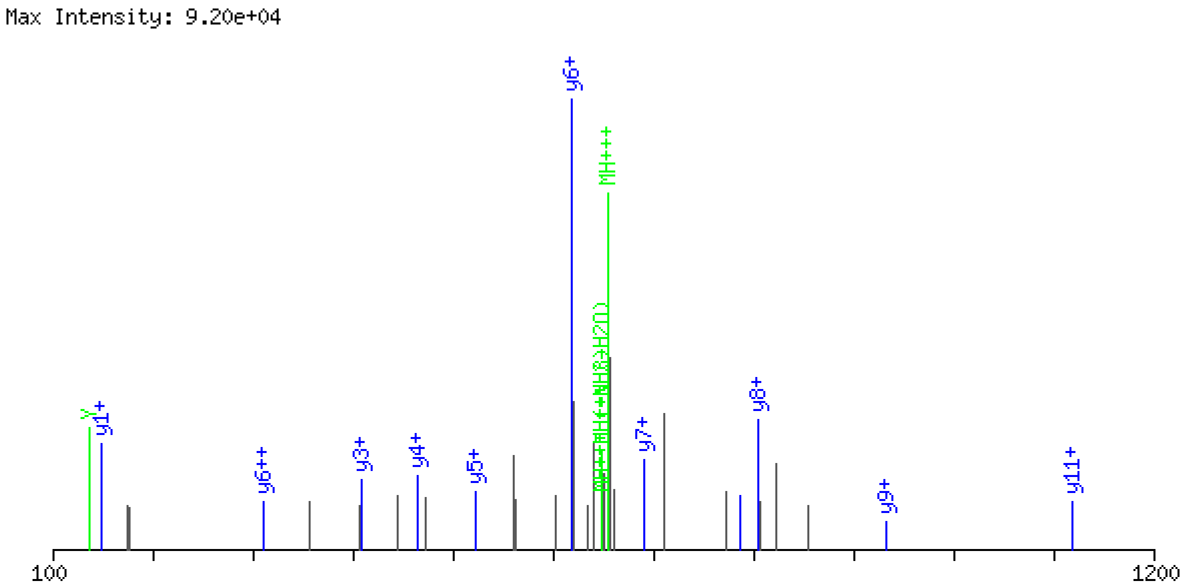

Hiding fragment losses - Click to Show

		b+	b++	y+	y++		
L	1	114.09	57.55			18	L
S	2	201.12	101.07	1848.83	924.92	17	S
T	3	382.14	191.57	1761.80	881.40	16	T
H	4	519.20	260.10	1580.78	790.89	15	H
N	5	633.24	317.12	1443.72	722.36	14	N
V	6	732.31	366.66	1329.68	665.34	13	V
I	7	845.39	423.20	1230.61	615.81	12	I
N	8	959.43	480.22	1117.53	559.27	11	N
A	9	1030.47	515.74	1003.48	502.25	10	A
E	10	1159.51	580.26	932.45	466.73	9	E
N	11	1273.56	637.28	803.40	402.21	8	N
A	12	1344.59	672.80	689.36	345.18	7	A
P	13	1441.65	721.33	618.32	309.67	6	P
G	14	1498.67	749.84	521.27	261.14	5	G
G	15	1555.69	778.35	464.25	232.63	4	G
P	16	1652.74	826.88	407.23	204.12	3	P
Y	17	1815.81	908.41	310.18	155.59	2	Y
K	18			147.11	74.06	1	K

B/Bris HA 451-482 unmodified

ADTISSQIELAVLLSNEGIINSEDEHLLALER

Charge: 3, Exp. m/z: 1164.940, Calc. m/z: 1164.941
Data File: edflowthruFBV30, Scans: 39598 - 39598

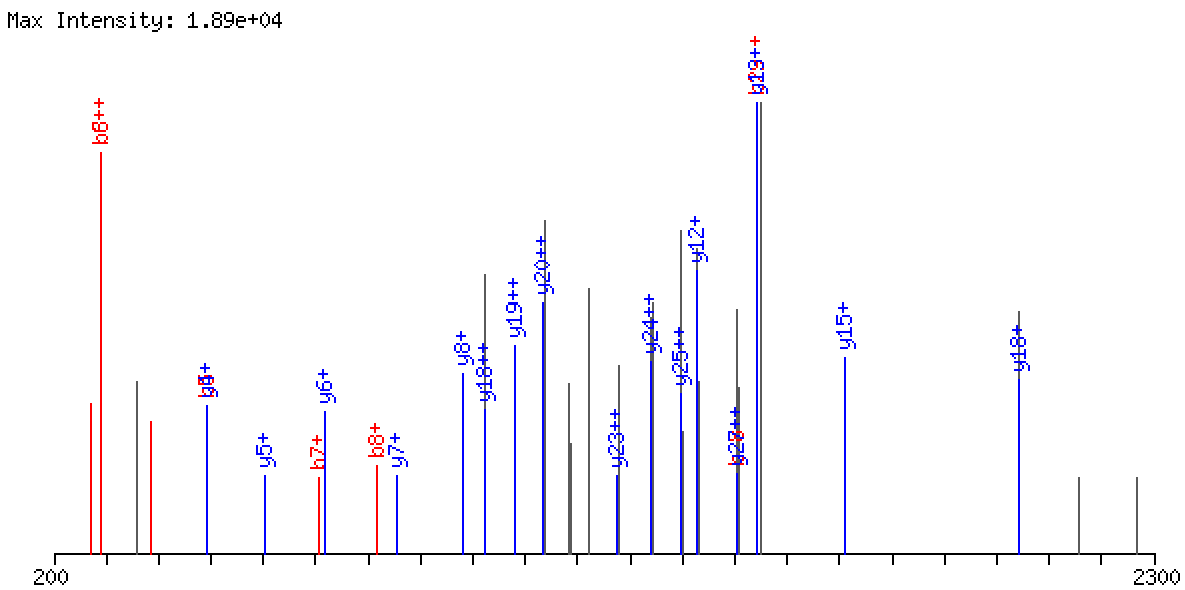

Hiding fragment losses - Click to Show

		b+	b++	y+	y++		
A	1	72.04	36.53			32	A
D	2	187.07	94.04	3421.77	1711.39	31	D
T	3	288.12	144.56	3306.74	1653.88	30	T
I	4	401.20	201.11	3205.70	1603.35	29	I
S	5	488.24	244.62	3092.61	1546.81	28	S
S	6	575.27	288.14	3005.58	1503.29	27	S
Q	7	703.33	352.17	2918.55	1459.78	26	Q
I	8	816.41	408.71	2790.49	1395.75	25	I
E	9	945.45	473.23	2677.40	1339.21	24	E
L	10	1058.54	529.77	2548.36	1274.68	23	L
A	11	1129.57	565.29	2435.28	1218.14	22	A
V	12	1228.64	614.82	2364.24	1182.62	21	V
L	13	1341.73	671.37	2265.17	1133.09	20	L
L	14	1454.81	727.91	2152.09	1076.55	19	L
S	15	1541.84	771.42	2039.00	1020.01	18	S
N	16	1655.89	828.45	1951.97	976.49	17	N
E	17	1784.93	892.97	1837.93	919.47	16	E
G	18	1841.95	921.48	1708.89	854.95	15	G
I	19	1955.03	978.02	1651.86	826.44	14	I
I	20	2068.12	1034.56	1538.78	769.89	13	I
N	21	2182.16	1091.58	1425.70	713.35	12	N
S	22	2269.19	1135.10	1311.65	656.33	11	S
E	23	2398.23	1199.62	1224.62	612.81	10	E

		b+	b++	y+	y++		
D	24	2513.26	1257.13	1095.58	548.29	9	D
E	25	2642.30	1321.66	980.55	490.78	8	E
H	26	2779.36	1390.19	851.51	426.26	7	H
L	27	2892.45	1446.73	714.45	357.73	6	L
L	28	3005.53	1503.27	601.37	301.19	5	L
A	29	3076.57	1538.79	488.28	244.65	4	A
L	30	3189.65	1595.33	417.25	209.13	3	L
E	31	3318.70	1659.85	304.16	152.58	2	E
R	32			175.12	88.06	1	R

B/Bris HA 451-482 phospho

ADTISSQIELAVLLS(+79.97)NEGIINSEDEHLLALER

Charge: 3, Exp. m/z: 1191.600, Calc. m/z: 1191.597
Data File: edflowthruFBV15, Scans: 32285 - 32285

Max Intensity: 4.10e+04

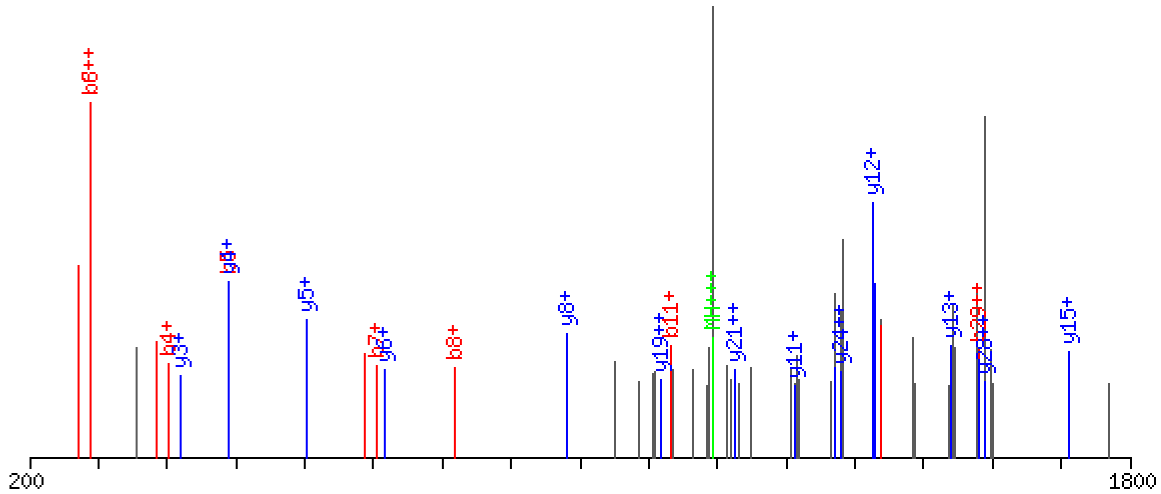

Hiding fragment losses - Click to Show

		b+	b++	y+	y++		
A	1	72.04	36.53			32	A
D	2	187.07	94.04	3501.74	1751.37	31	D
T	3	288.12	144.56	3386.71	1693.86	30	T
I	4	401.20	201.11	3285.66	1643.33	29	I
S	5	488.24	244.62	3172.58	1586.79	28	S
S	6	575.27	288.14	3085.55	1543.28	27	S
Q	7	703.33	352.17	2998.51	1499.76	26	Q
I	8	816.41	408.71	2870.45	1435.73	25	I
E	9	945.45	473.23	2757.37	1379.19	24	E
L	10	1058.54	529.77	2628.33	1314.67	23	L
A	11	1129.57	565.29	2515.24	1258.13	22	A
V	12	1228.64	614.82	2444.21	1222.61	21	V
L	13	1341.73	671.37	2345.14	1173.07	20	L
L	14	1454.81	727.91	2232.05	1116.53	19	L
S	15	1621.81	811.41	2118.97	1059.99	18	S
N	16	1735.85	868.43	1951.97	976.49	17	N
E	17	1864.89	932.95	1837.93	919.47	16	E
G	18	1921.92	961.46	1708.89	854.95	15	G
I	19	2035.00	1018.00	1651.86	826.44	14	I
I	20	2148.08	1074.55	1538.78	769.89	13	I
N	21	2262.13	1131.57	1425.70	713.35	12	N
S	22	2349.16	1175.08	1311.65	656.33	11	S
E	23	2478.20	1239.60	1224.62	612.81	10	E

		b+	b++	y+	y++		
D	24	2593.23	1297.12	1095.58	548.29	9	D
E	25	2722.27	1361.64	980.55	490.78	8	E
H	26	2859.33	1430.17	851.51	426.26	7	H
L	27	2972.41	1486.71	714.45	357.73	6	L
L	28	3085.50	1543.25	601.37	301.19	5	L
A	29	3156.53	1578.77	488.28	244.65	4	A
L	30	3269.62	1635.31	417.25	209.13	3	L
E	31	3398.66	1699.83	304.16	152.58	2	E
R	32			175.12	88.06	1	R

B/Bris NP 48-64 unmodified

NPSPERATTSSEDDVGR

Charge: 3, Exp. m/z: 606.613, Calc. m/z: 606.617
Data File: D120713_002, Scans: 7901 - 7901

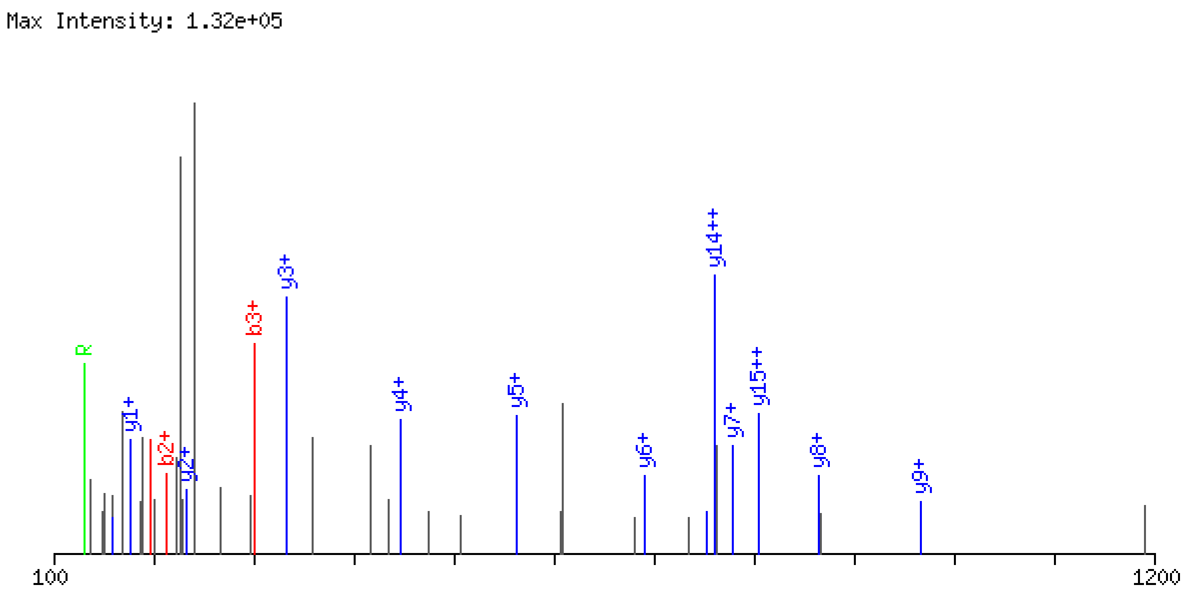

Hiding fragment losses - Click to Show

		b+	b++	y+	y++		
N	1	115.05	58.03			17	N
P	2	212.10	106.56	1703.78	852.40	16	P
S	3	299.13	150.07	1606.73	803.87	15	S
P	4	396.19	198.60	1519.70	760.35	14	P
E	5	525.23	263.12	1422.65	711.83	13	E
R	6	681.33	341.17	1293.60	647.31	12	R
A	7	752.37	376.69	1137.50	569.25	11	A
T	8	853.42	427.21	1066.46	533.74	10	T
T	9	954.46	477.74	965.42	483.21	9	T
S	10	1041.50	521.25	864.37	432.69	8	S
S	11	1128.53	564.77	777.34	389.17	7	S
E	12	1257.57	629.29	690.31	345.66	6	E
D	13	1372.60	686.80	561.26	281.14	5	D
D	14	1487.62	744.32	446.24	223.62	4	D
V	15	1586.69	793.85	331.21	166.11	3	V
G	16	1643.71	822.36	232.14	116.57	2	G
R	17			175.12	88.06	1	R

B/Bris NP 48-64 phospho

NPS(+79.97)PERATTSSEDDVGR

Charge: 3, Exp. m/z: 633.269, Calc. m/z: 633.271
Data File: B111206_011, Scans: 1372 - 1372

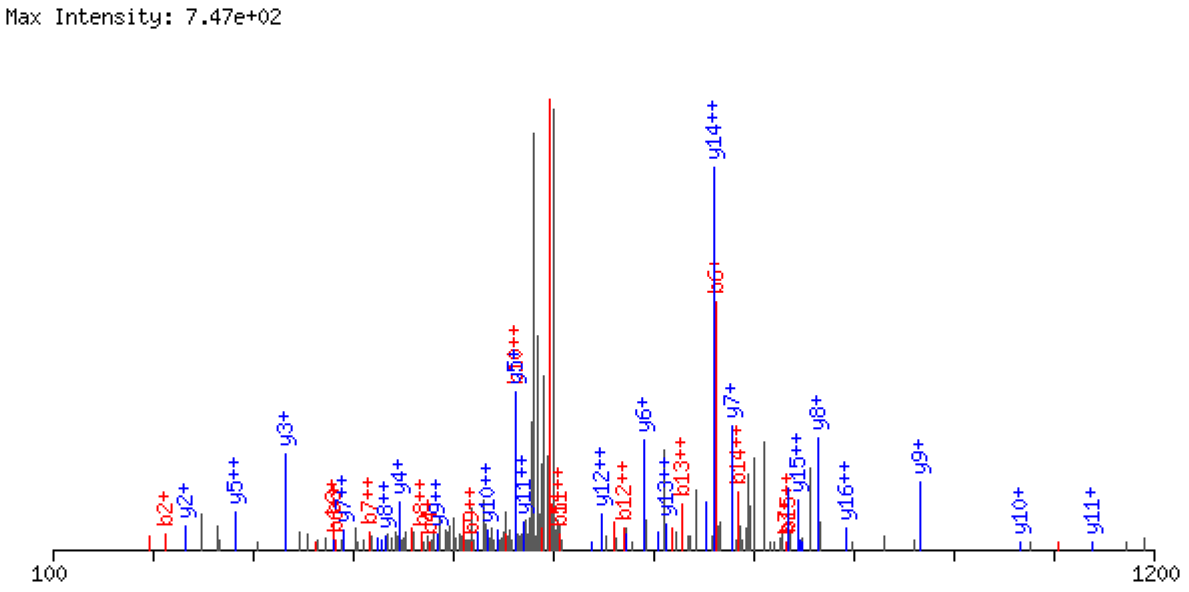

Hiding fragment losses - Click to Show

		b+	b++	y+	y++		
N	1	115.05	58.03			17	N
P	2	212.10	106.56	1783.75	892.38	16	P
S	3	379.10	190.05	1686.70	843.85	15	S
P	4	476.15	238.58	1519.70	760.35	14	P
E	5	605.20	303.10	1422.65	711.83	13	E
R	6	761.30	381.15	1293.60	647.31	12	R
A	7	832.33	416.67	1137.50	569.25	11	A
T	8	933.38	467.19	1066.46	533.74	10	T
T	9	1034.43	517.72	965.42	483.21	9	T
S	10	1121.46	561.23	864.37	432.69	8	S
S	11	1208.49	604.75	777.34	389.17	7	S
E	12	1337.54	669.27	690.31	345.66	6	E
D	13	1452.56	726.79	561.26	281.14	5	D
D	14	1567.59	784.30	446.24	223.62	4	D
V	15	1666.66	833.83	331.21	166.11	3	V
G	16	1723.68	862.34	232.14	116.57	2	G
R	17			175.12	88.06	1	R

B/Bris NP 46-64 phospho

TRNPS(+79.97)PERATTSSEDDVGR

Charge: 3, Exp. m/z: 718.985, Calc. m/z: 718.987
Data File: B120210_017, Scans: 2388 - 2388

Max Intensity: 3.63e+04

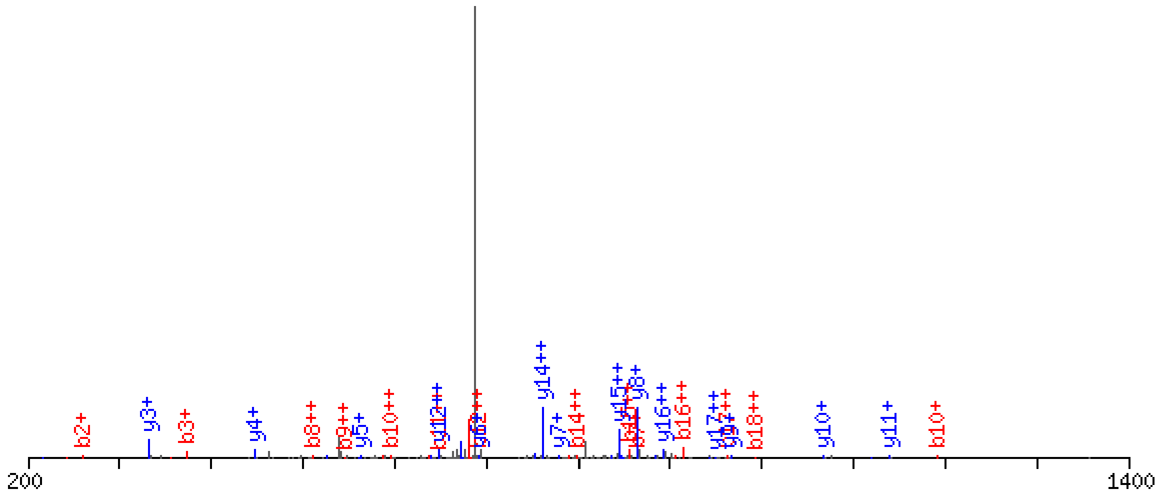

Hiding fragment losses - Click to Show

		b+	b++	y+	y++		
T	1	102.05	51.53			19	T
R	2	258.16	129.58	2053.89	1027.45	18	R
N	3	372.20	186.60	1897.79	949.40	17	N
P	4	469.25	235.13	1783.75	892.38	16	P
S	5	636.25	318.63	1686.70	843.85	15	S
P	6	733.30	367.16	1519.70	760.35	14	P
E	7	862.35	431.68	1422.65	711.83	13	E
R	8	1018.45	509.73	1293.60	647.31	12	R
A	9	1089.48	545.25	1137.50	569.25	11	A
T	10	1190.53	595.77	1066.46	533.74	10	T
T	11	1291.58	646.29	965.42	483.21	9	T
S	12	1378.61	689.81	864.37	432.69	8	S
S	13	1465.64	733.33	777.34	389.17	7	S
E	14	1594.69	797.85	690.31	345.66	6	E
D	15	1709.71	855.36	561.26	281.14	5	D
D	16	1824.74	912.87	446.24	223.62	4	D
V	17	1923.81	962.41	331.21	166.11	3	V
G	18	1980.83	990.92	232.14	116.57	2	G
R	19			175.12	88.06	1	R

B/Bris NP 48-65 phospho

NPSPERAT(+79.97)TSSEDDVGRK

Charge: 3, Exp. m/z: 675.967, Calc. m/z: 675.967
Data File: B120210_017, Scans: 2364 - 2364

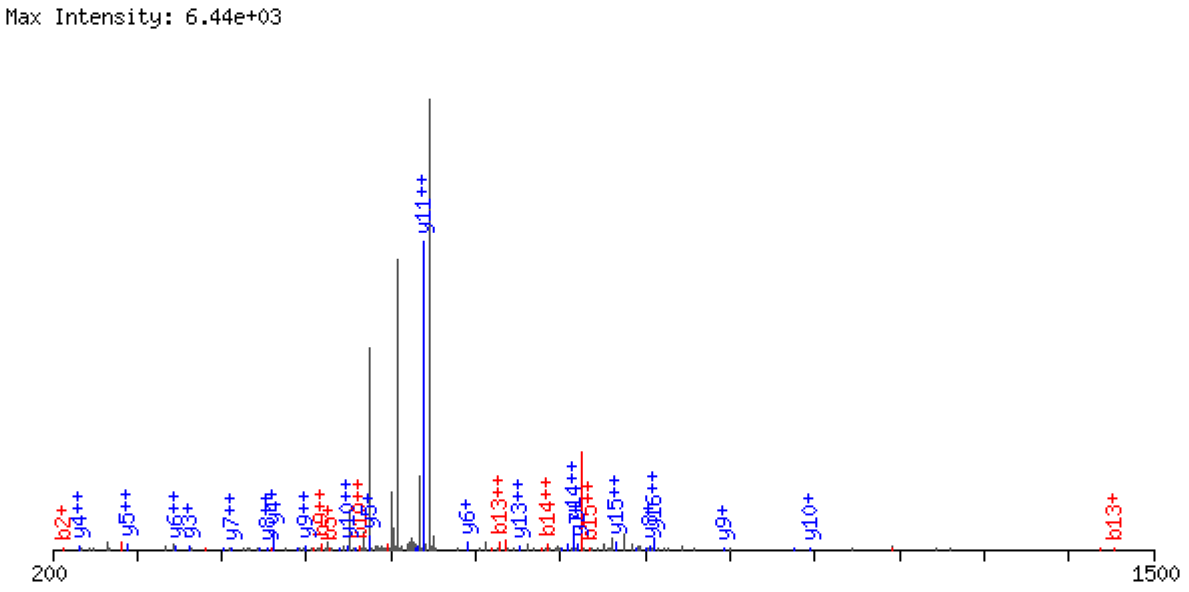

Hiding fragment losses - Click to Show

		b+	b++	y+	y++		
N	1	115.05	58.03			18	N
P	2	212.10	106.56	1911.84	956.43	17	P
S	3	299.13	150.07	1814.79	907.90	16	S
P	4	396.19	198.60	1727.76	864.38	15	P
E	5	525.23	263.12	1630.71	815.86	14	E
R	6	681.33	341.17	1501.66	751.34	13	R
A	7	752.37	376.69	1345.56	673.29	12	A
T	8	933.38	467.19	1274.53	637.77	11	T
T	9	1034.43	517.72	1093.51	547.26	10	T
S	10	1121.46	561.23	992.46	496.74	9	S
S	11	1208.49	604.75	905.43	453.22	8	S
E	12	1337.54	669.27	818.40	409.70	7	E
D	13	1452.56	726.79	689.36	345.18	6	D
D	14	1567.59	784.30	574.33	287.67	5	D
V	15	1666.66	833.83	459.30	230.16	4	V
G	16	1723.68	862.34	360.24	180.62	3	G
R	17	1879.78	940.39	303.21	152.11	2	R
K	18			147.11	74.06	1	K

B/Bris NP 54-64 unmodified

ATTSSEDDVGR

Charge: 2, Exp. m/z: 569.250, Calc. m/z: 569.257
Data File: D120713_003, Scans: 6302 - 6302

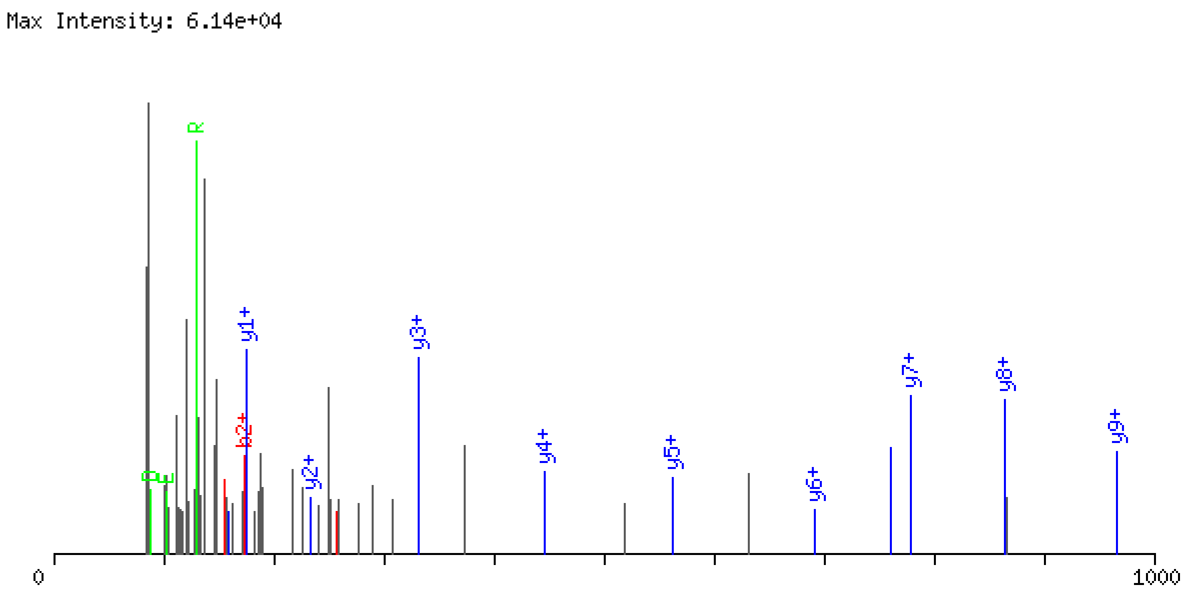

		b+	-H2O	y+	-H2O	-NH3		
A	1	72.04					11	A
T	2	173.09	155.08	1066.46	1048.45	1049.44	10	T
T	3	274.14	256.13	965.42	947.41	948.39	9	T
S	4	361.17	343.16	864.37	846.36	847.34	8	S
S	5	448.20	430.19	777.34	759.33	760.31	7	S
E	6	577.25	559.24	690.31	672.29	673.28	6	E
D	7	692.27	674.26	561.26	543.25	544.24	5	D
D	8	807.30	789.29	446.24	428.23	429.21	4	D
V	9	906.37	888.36	331.21		314.18	3	V
G	10	963.39	945.38	232.14		215.11	2	G
R	11			175.12		158.09	1	R

B/Bris NP 54-64 phospho

ATTS(+79.97)SEDDVGR

Charge: 2, Exp. m/z: 609.237, Calc. m/z: 609.242
Data File: D120713_002, Scans: 7282 - 7282

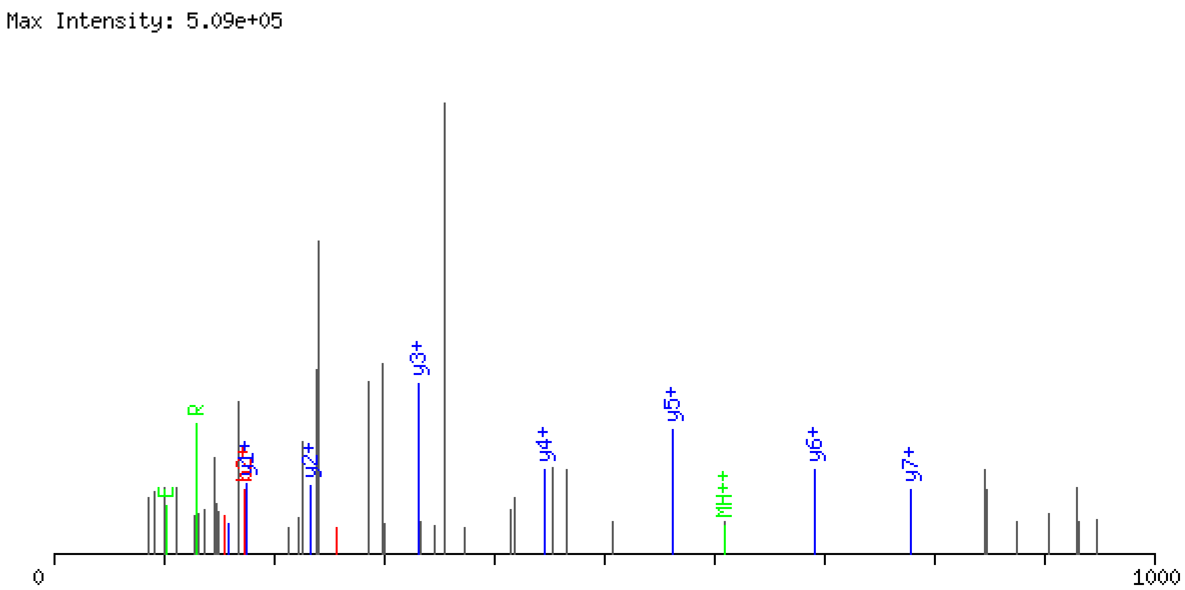

		b+	-H2O	y+	-H2O	-NH3		
A	1	72.04					11	A
T	2	173.09	155.08	1146.43	1128.42	1129.40	10	T
T	3	274.14	256.13	1045.38	1027.37	1028.36	9	T
S	4	441.14	423.13	944.34	926.33	927.31	8	S
S	5	528.17	510.16	777.34	759.33	760.31	7	S
E	6	657.21	639.20	690.31	672.29	673.28	6	E
D	7	772.24	754.23	561.26	543.25	544.24	5	D
D	8	887.27	869.26	446.24	428.23	429.21	4	D
V	9	986.34	968.32	331.21		314.18	3	V
G	10	1043.36	1025.35	232.14		215.11	2	G
R	11			175.12		158.09	1	R

B/Bris NP 218-235 unmodified

VGLDPSLISTFAGSTVPR

Charge: 2, Exp. m/z: 909.000, Calc. m/z: 908.997
Data File: edflowthruFBV30, Scans: 25823 - 25823

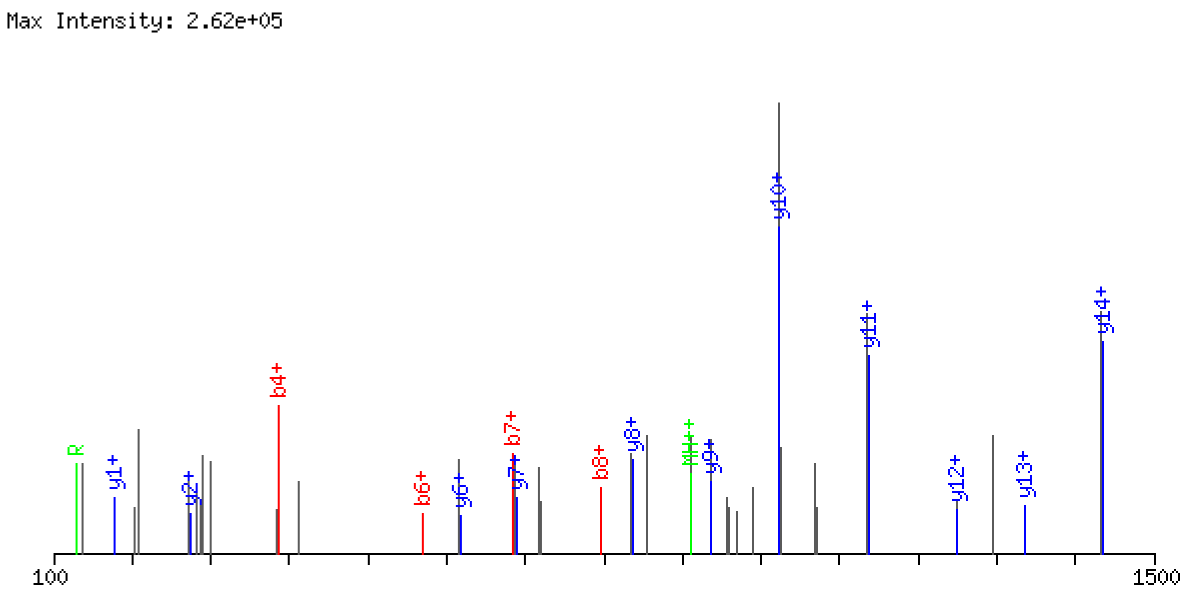

		b+	-H2O	y+	-H2O	-NH3		
V	1	100.08					18	V
G	2	157.10		1717.91	1699.90	1700.89	17	G
L	3	270.18		1660.89	1642.88	1643.86	16	L
D	4	385.21	367.20	1547.81	1529.80	1530.78	15	D
P	5	482.26	464.25	1432.78	1414.77	1415.75	14	P
S	6	569.29	551.28	1335.73	1317.72	1318.70	13	S
L	7	682.38	664.37	1248.69	1230.68	1231.67	12	L
I	8	795.46	777.45	1135.61	1117.60	1118.58	11	I
S	9	882.49	864.48	1022.53	1004.52	1005.50	10	S
T	10	983.54	965.53	935.49	917.48	918.47	9	T
F	11	1130.61	1112.60	834.45	816.44	817.42	8	F
A	12	1201.65	1183.64	687.38	669.37	670.35	7	A
G	13	1258.67	1240.66	616.34	598.33	599.31	6	G
S	14	1345.70	1327.69	559.32	541.31	542.29	5	S
T	15	1446.75	1428.74	472.29	454.28	455.26	4	T
V	16	1545.82	1527.81	371.24		354.21	3	V
P	17	1642.87	1624.86	272.17		255.15	2	P
R	18			175.12		158.09	1	R

B/Bris NP 218-235 phospho

VGLDPS(+79.97)LISTFAGSTVPR

Charge: 2, Exp. m/z: 949.480, Calc. m/z: 948.977
Data File: D120713_079, Scans: 13241 - 13241

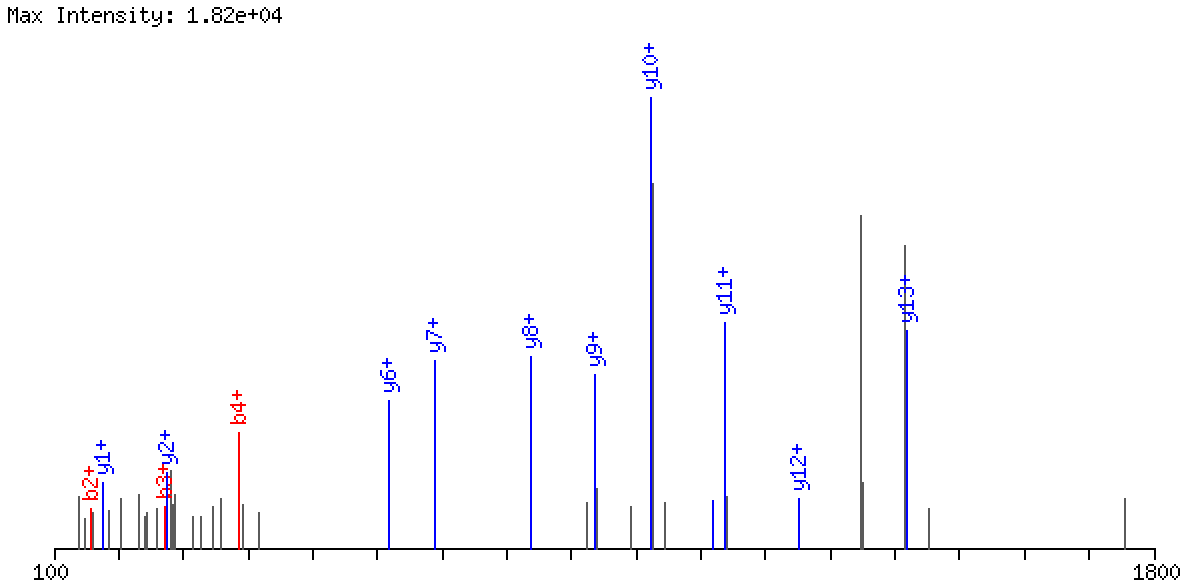

		b+	-H2O	y+	-H2O	-NH3		
V	1	100.08					18	V
G	2	157.10		1797.88	1779.87	1780.85	17	G
L	3	270.18		1740.86	1722.85	1723.83	16	L
D	4	385.21	367.20	1627.77	1609.76	1610.75	15	D
P	5	482.26	464.25	1512.75	1494.74	1495.72	14	P
S	6	649.26	631.25	1415.69	1397.68	1398.67	13	S
L	7	762.34	744.33	1248.69	1230.68	1231.67	12	L
I	8	875.43	857.42	1135.61	1117.60	1118.58	11	I
S	9	962.46	944.45	1022.53	1004.52	1005.50	10	S
T	10	1063.51	1045.50	935.49	917.48	918.47	9	T
F	11	1210.58	1192.56	834.45	816.44	817.42	8	F
A	12	1281.61	1263.60	687.38	669.37	670.35	7	A
G	13	1338.63	1320.62	616.34	598.33	599.31	6	G
S	14	1425.67	1407.66	559.32	541.31	542.29	5	S
T	15	1526.71	1508.70	472.29	454.28	455.26	4	T
V	16	1625.78	1607.77	371.24		354.21	3	V
P	17	1722.83	1704.82	272.17		255.15	2	P
R	18			175.12		158.09	1	R

B/Bris NP 342-373 unmodified

IPQLGFNVEEYSMVGYEAMALYNMATPVSILR

Charge: 3, Exp. m/z: 1203.600, Calc. m/z: 1202.927
Data File: D120713_081, Scans: 30063 - 30063

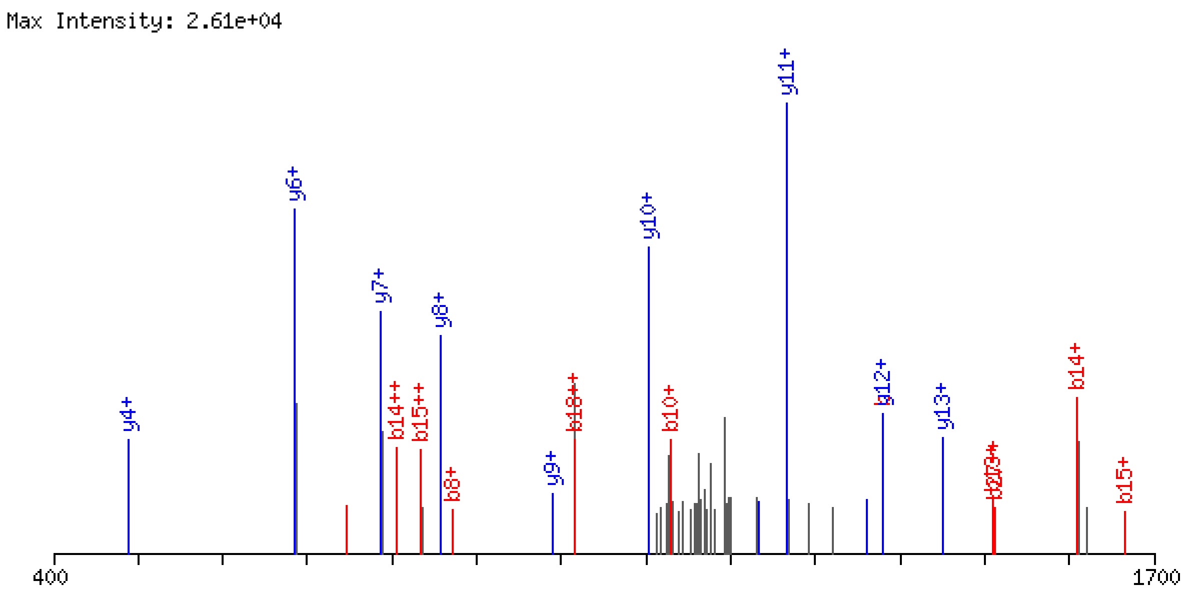

Hiding fragment losses - Click to Show

		b+	b++	y+	y++		
I	1	114.09	57.55			32	I
P	2	211.14	106.08	3493.68	1747.35	31	P
Q	3	339.20	170.10	3396.63	1698.82	30	Q
L	4	452.29	226.65	3268.57	1634.79	29	L
G	5	509.31	255.16	3155.49	1578.25	28	G
F	6	656.38	328.69	3098.47	1549.74	27	F
N	7	770.42	385.71	2951.40	1476.20	26	N
V	8	869.49	435.25	2837.36	1419.18	25	V
E	9	998.53	499.77	2738.29	1369.65	24	E
E	10	1127.57	564.29	2609.24	1305.13	23	E
Y	11	1290.64	645.82	2480.20	1240.60	22	Y
S	12	1377.67	689.34	2317.14	1159.07	21	S
M	13	1508.71	754.86	2230.11	1115.56	20	M
V	14	1607.78	804.39	2099.07	1050.04	19	V
G	15	1664.80	832.90	2000.00	1000.50	18	G
Y	16	1827.86	914.43	1942.98	971.99	17	Y
E	17	1956.90	978.96	1779.91	890.46	16	E
A	18	2027.94	1014.47	1650.87	825.94	15	A
M	19	2158.98	1079.99	1579.83	790.42	14	M
A	20	2230.02	1115.51	1448.79	724.90	13	A
L	21	2343.10	1172.06	1377.76	689.38	12	L
Y	22	2506.17	1253.59	1264.67	632.84	11	Y
N	23	2620.21	1310.61	1101.61	551.31	10	N

		b+	b++	y+	y++		
M	24	2751.25	1376.13	987.57	494.29	9	M
A	25	2822.29	1411.65	856.53	428.77	8	A
T	26	2923.34	1462.17	785.49	393.25	7	T
P	27	3020.39	1510.70	684.44	342.72	6	P
V	28	3119.46	1560.23	587.39	294.20	5	V
S	29	3206.49	1603.75	488.32	244.66	4	S
I	30	3319.57	1660.29	401.29	201.15	3	I
L	31	3432.66	1716.83	288.20	144.61	2	L
R	32			175.12	88.06	1	R

B/Bris NP 342-373 phospho

IPQLGFNVEEY(+79.97)SMVGYEAMALYNMATPVSILR

Charge: 3, Exp. m/z: 1229.920, Calc. m/z: 1229.581
Data File: D120713_081, Scans: 27112 - 27112

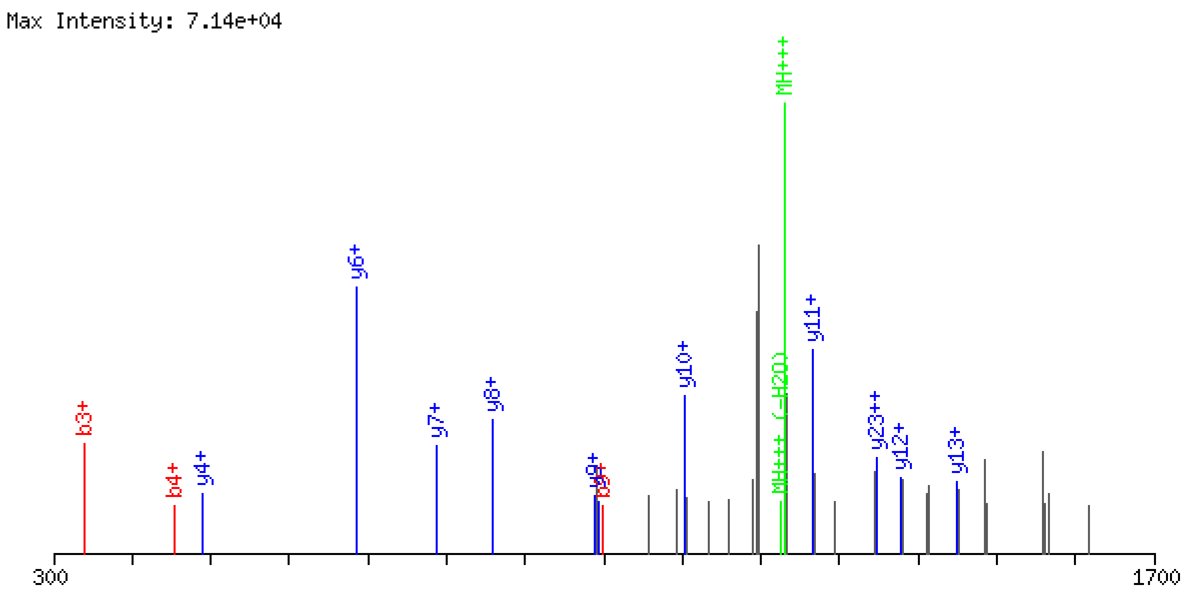

Hiding fragment losses - Click to Show

		b+	b++	y+	y++		
I	1	114.09	57.55			32	I
P	2	211.14	106.08	3573.65	1787.33	31	P
Q	3	339.20	170.10	3476.60	1738.80	30	Q
L	4	452.29	226.65	3348.54	1674.77	29	L
G	5	509.31	255.16	3235.45	1618.23	28	G
F	6	656.38	328.69	3178.43	1589.72	27	F
N	7	770.42	385.71	3031.37	1516.19	26	N
V	8	869.49	435.25	2917.32	1459.16	25	V
E	9	998.53	499.77	2818.25	1409.63	24	E
E	10	1127.57	564.29	2689.21	1345.11	23	E
Y	11	1370.60	685.81	2560.17	1280.59	22	Y
S	12	1457.63	729.32	2317.14	1159.07	21	S
M	13	1588.68	794.84	2230.11	1115.56	20	M
V	14	1687.74	844.38	2099.07	1050.04	19	V
G	15	1744.77	872.89	2000.00	1000.50	18	G
Y	16	1907.83	954.42	1942.98	971.99	17	Y
E	17	2036.87	1018.94	1779.91	890.46	16	E
A	18	2107.91	1054.46	1650.87	825.94	15	A
M	19	2238.95	1119.98	1579.83	790.42	14	M
A	20	2309.99	1155.50	1448.79	724.90	13	A
L	21	2423.07	1212.04	1377.76	689.38	12	L
Y	22	2586.13	1293.57	1264.67	632.84	11	Y
N	23	2700.18	1350.59	1101.61	551.31	10	N

		b+	b++	y+	y++		
M	24	2831.22	1416.11	987.57	494.29	9	M
A	25	2902.25	1451.63	856.53	428.77	8	A
T	26	3003.30	1502.15	785.49	393.25	7	T
P	27	3100.35	1550.68	684.44	342.72	6	P
V	28	3199.42	1600.21	587.39	294.20	5	V
S	29	3286.45	1643.73	488.32	244.66	4	S
I	30	3399.54	1700.27	401.29	201.15	3	I
L	31	3512.62	1756.82	288.20	144.61	2	L
R	32			175.12	88.06	1	R

B/Bris NP 448-478 carbamidomethyl

SGGNEVGGDGGSGQISC(+57.02)SPVFAVERPIALSK

Charge: 3, Exp. m/z: 1011.490, Calc. m/z: 1011.494
Data File: D120713_079, Scans: 13567 - 13567

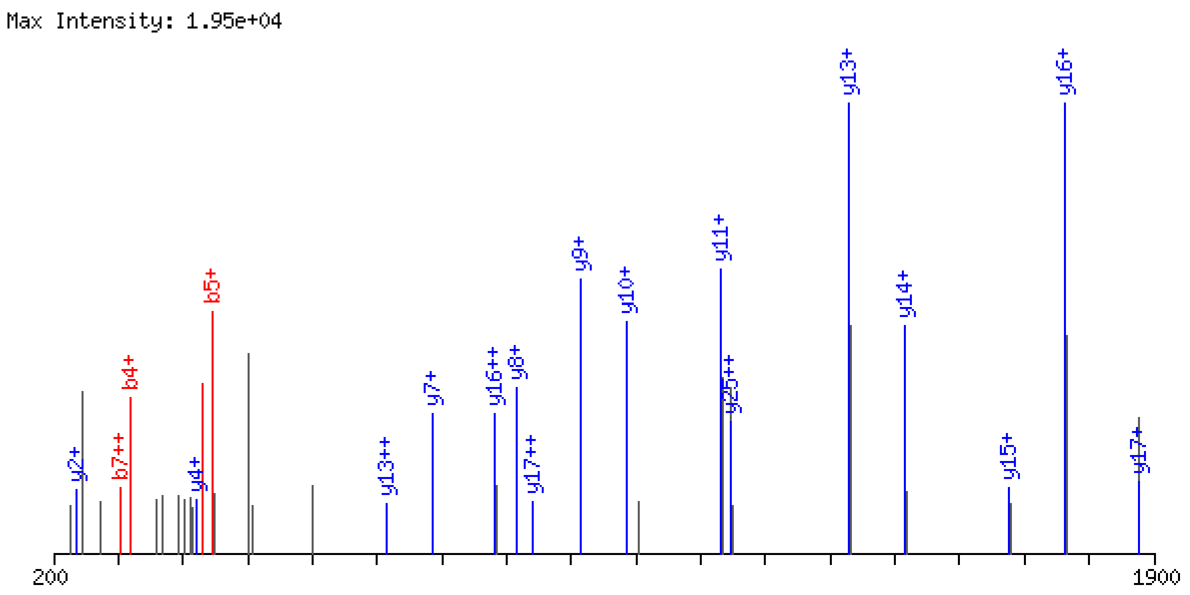

Hiding fragment losses - Click to Show

		b+	b++	y+	y++		
S	1	88.04	44.52			31	S
G	2	145.06	73.03	2945.44	1473.22	30	G
G	3	202.08	101.54	2888.42	1444.71	29	G
N	4	316.13	158.57	2831.40	1416.20	28	N
E	5	445.17	223.09	2717.36	1359.18	27	E
V	6	544.24	272.62	2588.31	1294.66	26	V
G	7	601.26	301.13	2489.25	1245.13	25	G
G	8	658.28	329.64	2432.22	1216.62	24	G
D	9	773.31	387.16	2375.20	1188.10	23	D
G	10	830.33	415.67	2260.18	1130.59	22	G
G	11	887.35	444.18	2203.15	1102.08	21	G
S	12	974.38	487.69	2146.13	1073.57	20	S
G	13	1031.40	516.20	2059.10	1030.05	19	G
Q	14	1159.46	580.23	2002.08	1001.54	18	Q
I	15	1272.55	636.78	1874.02	937.51	17	I
S	16	1359.58	680.29	1760.94	880.97	16	S
C	17	1519.61	760.31	1673.90	837.46	15	C
S	18	1606.64	803.82	1513.87	757.44	14	S
P	19	1703.69	852.35	1426.84	713.92	13	P
V	20	1802.76	901.88	1329.79	665.40	12	V
F	21	1949.83	975.42	1230.72	615.86	11	F
A	22	2020.87	1010.94	1083.65	542.33	10	A
V	23	2119.93	1060.47	1012.61	506.81	9	V

		b+	b++	y+	y++		
E	24	2248.98	1124.99	913.55	457.28	8	E
R	25	2405.08	1203.04	784.50	392.76	7	R
P	26	2502.13	1251.57	628.40	314.71	6	P
I	27	2615.22	1308.11	531.35	266.18	5	I
A	28	2686.25	1343.63	418.27	209.64	4	A
L	29	2799.34	1400.17	347.23	174.12	3	L
S	30	2886.37	1443.69	234.14	117.58	2	S
K	31			147.11	74.06	1	K

B/Bris NP 448-478 phospho + carbamidomethyl

SGGNEVGGDGGS(+79.97)GQISC(+57.02)SPVFAVERPIALSK

Charge: 3, Exp. m/z: 1038.150, Calc. m/z: 1038.151
Data File: D120713_079, Scans: 11615 - 11615

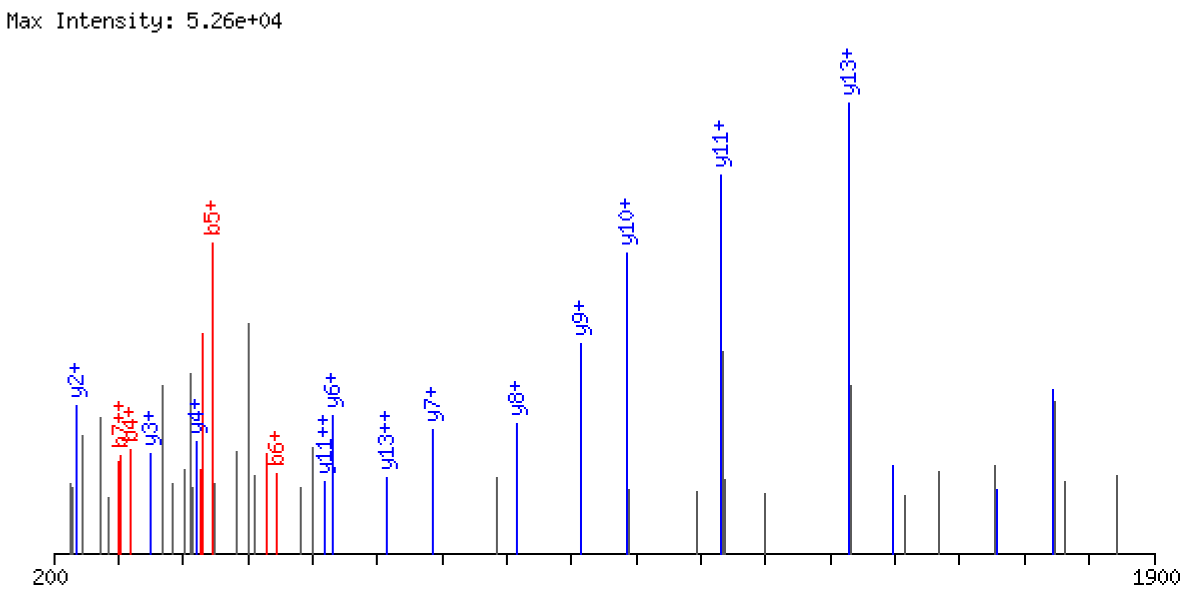

Hiding fragment losses - Click to Show

		b+	b++	y+	y++		
S	1	88.04	44.52			31	S
G	2	145.06	73.03	3025.41	1513.21	30	G
G	3	202.08	101.54	2968.39	1484.70	29	G
N	4	316.13	158.57	2911.37	1456.19	28	N
E	5	445.17	223.09	2797.32	1399.16	27	E
V	6	544.24	272.62	2668.28	1334.64	26	V
G	7	601.26	301.13	2569.21	1285.11	25	G
G	8	658.28	329.64	2512.19	1256.60	24	G
D	9	773.31	387.16	2455.17	1228.09	23	D
G	10	830.33	415.67	2340.14	1170.57	22	G
G	11	887.35	444.18	2283.12	1142.06	21	G
S	12	1054.35	527.68	2226.10	1113.55	20	S
G	13	1111.37	556.19	2059.10	1030.05	19	G
Q	14	1239.43	620.22	2002.08	1001.54	18	Q
I	15	1352.51	676.76	1874.02	937.51	17	I
S	16	1439.54	720.28	1760.94	880.97	16	S
C	17	1599.57	800.29	1673.90	837.46	15	C
S	18	1686.61	843.81	1513.87	757.44	14	S
P	19	1783.66	892.33	1426.84	713.92	13	P
V	20	1882.73	941.87	1329.79	665.40	12	V
F	21	2029.80	1015.40	1230.72	615.86	11	F
A	22	2100.83	1050.92	1083.65	542.33	10	A
V	23	2199.90	1100.45	1012.61	506.81	9	V

		b+	b++	y+	y++		
E	24	2328.94	1164.98	913.55	457.28	8	E
R	25	2485.04	1243.03	784.50	392.76	7	R
P	26	2582.10	1291.55	628.40	314.71	6	P
I	27	2695.18	1348.09	531.35	266.18	5	I
A	28	2766.22	1383.61	418.27	209.64	4	A
L	29	2879.30	1440.16	347.23	174.12	3	L
S	30	2966.34	1483.67	234.14	117.58	2	S
K	31			147.11	74.06	1	K

B/Bris NP 484-492 unmodified

MLSMNIEGR

Charge: 2, Exp. m/z: 525.757, Calc. m/z: 525.762
Data File: D120713_079, Scans: 10453 - 10453

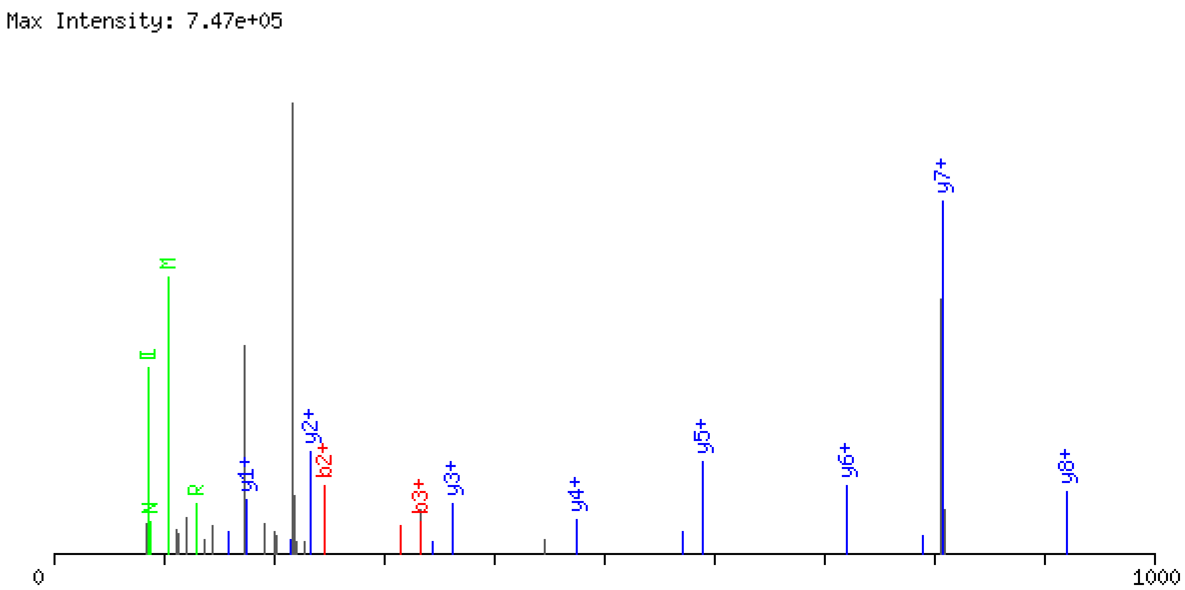

		b+	-H2O	-NH3	y+	-H2O	-NH3		
M	1	132.05						9	M
L	2	245.13			919.47	901.46	902.44	8	L
S	3	332.16	314.15		806.38	788.37	789.36	7	S
M	4	463.20	445.19		719.35	701.34	702.32	6	M
N	5	577.25	559.24	560.22	588.31	570.30	571.28	5	N
I	6	690.33	672.32	673.30	474.27	456.26	457.24	4	I
E	7	819.37	801.36	802.35	361.18	343.17	344.16	3	E
G	8	876.40	858.38	859.37	232.14		215.11	2	G
R	9				175.12		158.09	1	R

B/Bris NP 484-492 phospho

MLS(+79.97)MNIEGR

Charge: 2, Exp. m/z: 565.740, Calc. m/z: 565.742
Data File: D120713_002, Scans: 12742 - 12742

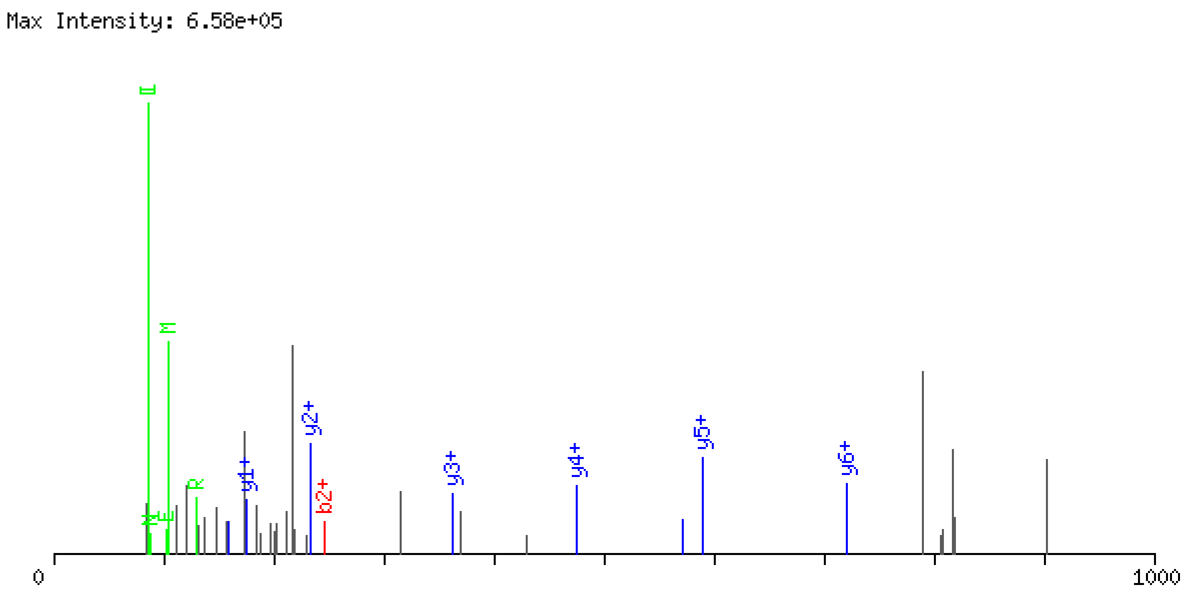

		b+	-H2O	-NH3	y+	-H2O	-NH3		
M	1	132.05						9	M
L	2	245.13			999.43	981.42	982.41	8	L
S	3	412.13	394.12		886.35	868.34	869.32	7	S
M	4	543.17	525.16		719.35	701.34	702.32	6	M
N	5	657.21	639.20	640.19	588.31	570.30	571.28	5	N
I	6	770.30	752.29	753.27	474.27	456.26	457.24	4	I
E	7	899.34	881.33	882.31	361.18	343.17	344.16	3	E
G	8	956.36	938.35	939.34	232.14		215.11	2	G
R	9				175.12		158.09	1	R

B/Bris M1 2-27 unmodified

SLFGDTIAYLLSLTEDGEGKAELAEK

Charge: 3, Exp. m/z: 924.470, Calc. m/z: 924.141
Data File: edflowthruFBV15, Scans: 33018 - 33018

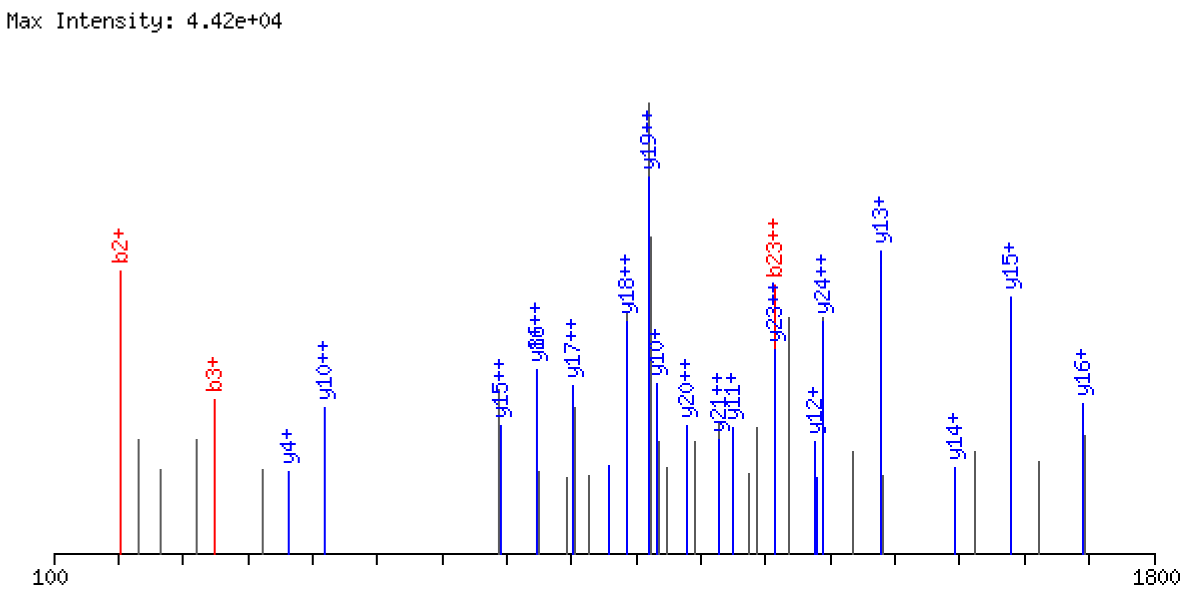

Hiding fragment losses - Click to Show

		b+	b++	y+	y++		
S	1	88.04	44.52			26	S
L	2	201.12	101.07	2683.37	1342.19	25	L
F	3	348.19	174.60	2570.29	1285.65	24	F
G	4	405.21	203.11	2423.22	1212.11	23	G
D	5	520.24	260.62	2366.20	1183.60	22	D
T	6	621.29	311.15	2251.17	1126.09	21	T
I	7	734.37	367.69	2150.12	1075.56	20	I
A	8	805.41	403.21	2037.04	1019.02	19	A
Y	9	968.47	484.74	1966.00	983.50	18	Y
L	10	1081.56	541.28	1802.94	901.97	17	L
L	11	1194.64	597.82	1689.85	845.43	16	L
S	12	1281.67	641.34	1576.77	788.89	15	S
L	13	1394.76	697.88	1489.74	745.37	14	L
T	14	1495.80	748.41	1376.65	688.83	13	T
E	15	1624.85	812.93	1275.61	638.31	12	E
D	16	1739.87	870.44	1146.56	573.79	11	D
G	17	1796.90	898.95	1031.54	516.27	10	G
E	18	1925.94	963.47	974.52	487.76	9	E
G	19	1982.96	991.98	845.47	423.24	8	G
K	20	2111.05	1056.03	788.45	394.73	7	K
A	21	2182.09	1091.55	660.36	330.68	6	A
E	22	2311.13	1156.07	589.32	295.16	5	E
L	23	2424.22	1212.61	460.28	230.64	4	L

		b+	b++	y+	y++		
A	24	2495.26	1248.13	347.19	174.10	3	A
E	25	2624.30	1312.65	276.16	138.58	2	E
K	26			147.11	74.06	1	K

B/Bris M1 2-27 phospho

S(+79.97)LFGDTIAYLLSLTEDGEGKAELAEK

Charge: 4, Exp. m/z: 713.850, Calc. m/z: 713.347
Data File: D120713_081, Scans: 30399 - 30399

Max Intensity: 1.95e+05

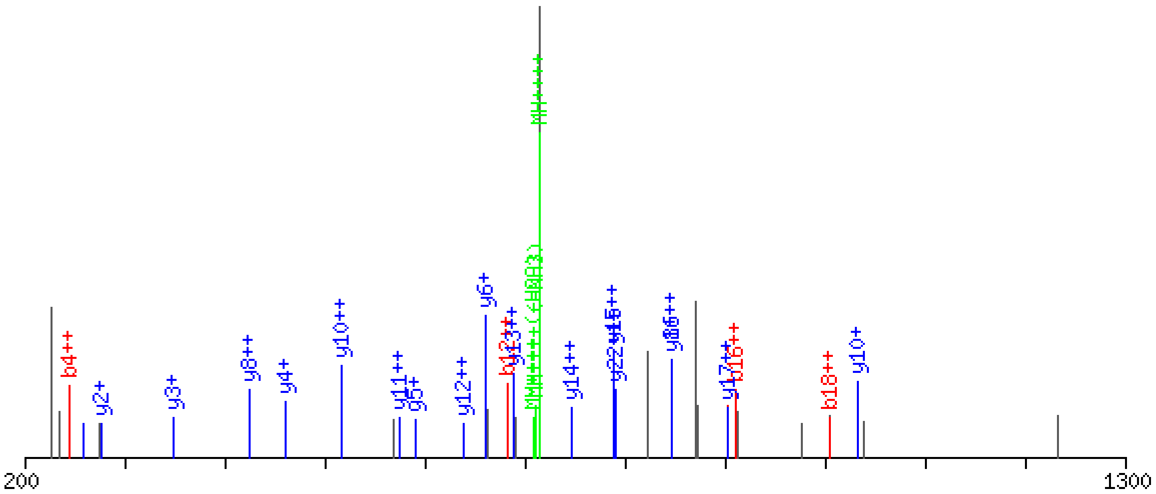

Hiding fragment losses - Click to Show

		b+	b++	b+++	y+	y++	y+++		
S	1	168.01	84.51	56.67				26	S
L	2	281.09	141.05	94.37	2683.37	1342.19	895.13	25	L
F	3	428.16	214.58	143.39	2570.29	1285.65	857.43	24	F
G	4	485.18	243.09	162.40	2423.22	1212.11	808.41	23	G
D	5	600.21	300.61	200.74	2366.20	1183.60	789.40	22	D
T	6	701.25	351.13	234.42	2251.17	1126.09	751.06	21	T
I	7	814.34	407.67	272.12	2150.12	1075.56	717.38	20	I
A	8	885.38	443.19	295.80	2037.04	1019.02	679.68	19	A
Y	9	1048.44	524.72	350.15	1966.00	983.50	656.01	18	Y
L	10	1161.52	581.27	387.85	1802.94	901.97	601.65	17	L
L	11	1274.61	637.81	425.54	1689.85	845.43	563.96	16	L
S	12	1361.64	681.32	454.55	1576.77	788.89	526.26	15	S
L	13	1474.72	737.87	492.25	1489.74	745.37	497.25	14	L
T	14	1575.77	788.39	525.93	1376.65	688.83	459.56	13	T
E	15	1704.81	852.91	568.94	1275.61	638.31	425.87	12	E
D	16	1819.84	910.42	607.28	1146.56	573.79	382.86	11	D
G	17	1876.86	938.93	626.29	1031.54	516.27	344.52	10	G
E	18	2005.90	1003.46	669.31	974.52	487.76	325.51	9	E
G	19	2062.93	1031.97	688.31	845.47	423.24	282.50	8	G
K	20	2191.02	1096.01	731.01	788.45	394.73	263.49	7	K
A	21	2262.06	1131.53	754.69	660.36	330.68	220.79	6	A
E	22	2391.10	1196.05	797.70	589.32	295.16	197.11	5	E
L	23	2504.18	1252.60	835.40	460.28	230.64	154.10	4	L

		b+	b++	b+++	y+	y++	y+++		
A	24	2575.22	1288.11	859.08	347.19	174.10	116.40	3	A
E	25	2704.26	1352.64	902.09	276.16	138.58	92.72	2	E
K	26				147.11	74.06	49.71	1	K

B/Bris M1 2-27 Met loss

SLFGDTIAYLLSLTEDGEGKAELAEK

Charge: 3, Exp. m/z: 924.137, Calc. m/z: 924.141
Data File: B120210_017, Scans: 6875 - 6875

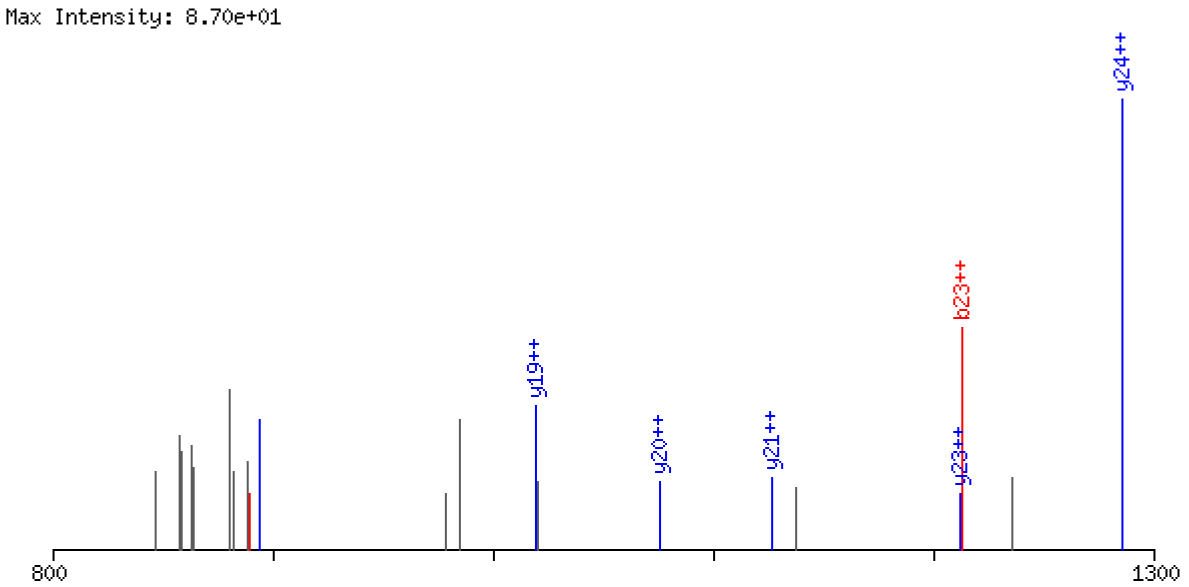

Hiding fragment losses - Click to Show

		b+	b++	y+	y++		
S	1	88.04	44.52			26	S
L	2	201.12	101.07	2683.37	1342.19	25	L
F	3	348.19	174.60	2570.29	1285.65	24	F
G	4	405.21	203.11	2423.22	1212.11	23	G
D	5	520.24	260.62	2366.20	1183.60	22	D
T	6	621.29	311.15	2251.17	1126.09	21	T
I	7	734.37	367.69	2150.12	1075.56	20	I
A	8	805.41	403.21	2037.04	1019.02	19	A
Y	9	968.47	484.74	1966.00	983.50	18	Y
L	10	1081.56	541.28	1802.94	901.97	17	L
L	11	1194.64	597.82	1689.85	845.43	16	L
S	12	1281.67	641.34	1576.77	788.89	15	S
L	13	1394.76	697.88	1489.74	745.37	14	L
T	14	1495.80	748.41	1376.65	688.83	13	T
E	15	1624.85	812.93	1275.61	638.31	12	E
D	16	1739.87	870.44	1146.56	573.79	11	D
G	17	1796.90	898.95	1031.54	516.27	10	G
E	18	1925.94	963.47	974.52	487.76	9	E
G	19	1982.96	991.98	845.47	423.24	8	G
K	20	2111.05	1056.03	788.45	394.73	7	K
A	21	2182.09	1091.55	660.36	330.68	6	A
E	22	2311.13	1156.07	589.32	295.16	5	E
L	23	2424.22	1212.61	460.28	230.64	4	L
A	24	2495.26	1248.13	347.19	174.10	3	A
E	25	2624.30	1312.65	276.16	138.58	2	E
K	26			147.11	74.06	1	K

B/Bris M1 2-27 Met loss and phospho

SLFGDT(+79.97)IAYLLSLTEDGEGKAELAEK

Charge: 4, Exp. m/z: 713.345, Calc. m/z: 713.347
Data File: B120210_017, Scans: 7140 - 7140

Max Intensity: 1.77e+03

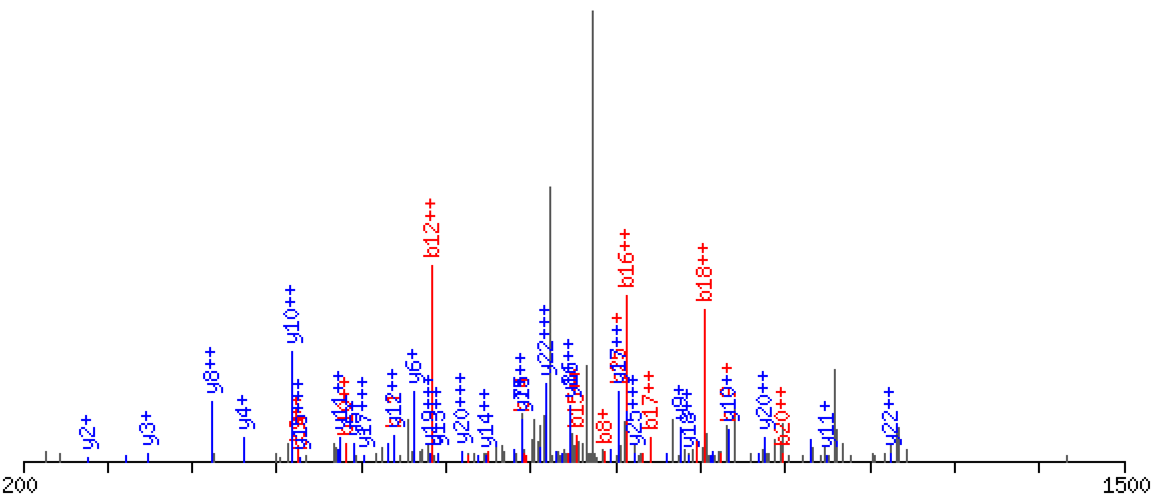

Hiding fragment losses - Click to Show

		b+	b++	b+++	y+	y++	y+++		
S	1	88.04	44.52	30.02				26	S
L	2	201.12	101.07	67.71	2763.34	1382.17	921.78	25	L
F	3	348.19	174.60	116.74	2650.25	1325.63	884.09	24	F
G	4	405.21	203.11	135.74	2503.19	1252.10	835.07	23	G
D	5	520.24	260.62	174.08	2446.16	1223.59	816.06	22	D
T	6	701.25	351.13	234.42	2331.14	1166.07	777.72	21	T
I	7	814.34	407.67	272.12	2150.12	1075.56	717.38	20	I
A	8	885.38	443.19	295.80	2037.04	1019.02	679.68	19	A
Y	9	1048.44	524.72	350.15	1966.00	983.50	656.01	18	Y
L	10	1161.52	581.27	387.85	1802.94	901.97	601.65	17	L
L	11	1274.61	637.81	425.54	1689.85	845.43	563.96	16	L
S	12	1361.64	681.32	454.55	1576.77	788.89	526.26	15	S
L	13	1474.72	737.87	492.25	1489.74	745.37	497.25	14	L
T	14	1575.77	788.39	525.93	1376.65	688.83	459.56	13	T
E	15	1704.81	852.91	568.94	1275.61	638.31	425.87	12	E
D	16	1819.84	910.42	607.28	1146.56	573.79	382.86	11	D
G	17	1876.86	938.93	626.29	1031.54	516.27	344.52	10	G
E	18	2005.90	1003.46	669.31	974.52	487.76	325.51	9	E
G	19	2062.93	1031.97	688.31	845.47	423.24	282.50	8	G
K	20	2191.02	1096.01	731.01	788.45	394.73	263.49	7	K
A	21	2262.06	1131.53	754.69	660.36	330.68	220.79	6	A
E	22	2391.10	1196.05	797.70	589.32	295.16	197.11	5	E
L	23	2504.18	1252.60	835.40	460.28	230.64	154.10	4	L
A	24	2575.22	1288.11	859.08	347.19	174.10	116.40	3	A
E	25	2704.26	1352.64	902.09	276.16	138.58	92.72	2	E
K	26				147.11	74.06	49.71	1	K

B/Bris M1 36-47 unmodified

EFDLDSALEWIK

Charge: 2, Exp. m/z: 733.360, Calc. m/z: 733.367
Data File: D120713_081, Scans: 29106 - 29106

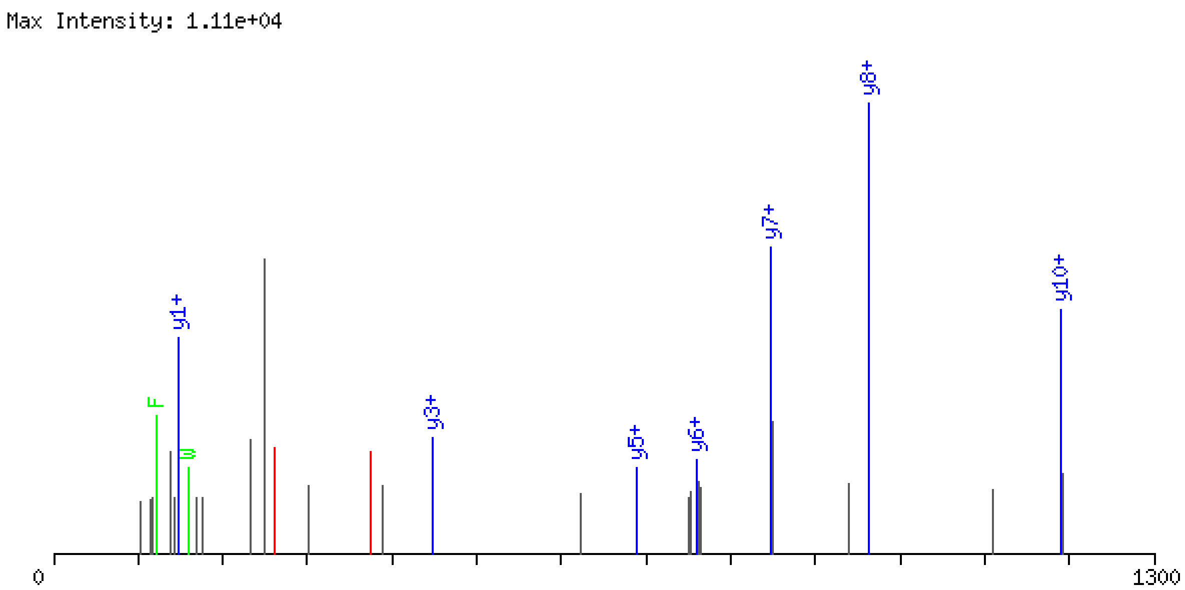

		b+	-H2O	y+	-H2O	-NH3		
E	1	130.05	112.04				12	E
F	2	277.12	259.11	1336.68	1318.67	1319.65	11	F
D	3	392.15	374.13	1189.61	1171.60	1172.58	10	D
L	4	505.23	487.22	1074.58	1056.57	1057.56	9	L
D	5	620.26	602.25	961.50	943.49	944.47	8	D
S	6	707.29	689.28	846.47	828.46	829.45	7	S
A	7	778.33	760.31	759.44	741.43	742.41	6	A
L	8	891.41	873.40	688.40	670.39	671.38	5	L
E	9	1020.45	1002.44	575.32	557.31	558.29	4	E
W	10	1206.53	1188.52	446.28		429.25	3	W
I	11	1319.62	1301.60	260.20		243.17	2	I
K	12			147.11		130.09	1	K

B/Bris M1 36-47 phospho

EFDLDS(+79.97)ALEWIK

Charge: 2, Exp. m/z: 773.850, Calc. m/z: 773.352
Data File: D120713_002, Scans: 16293 - 16293

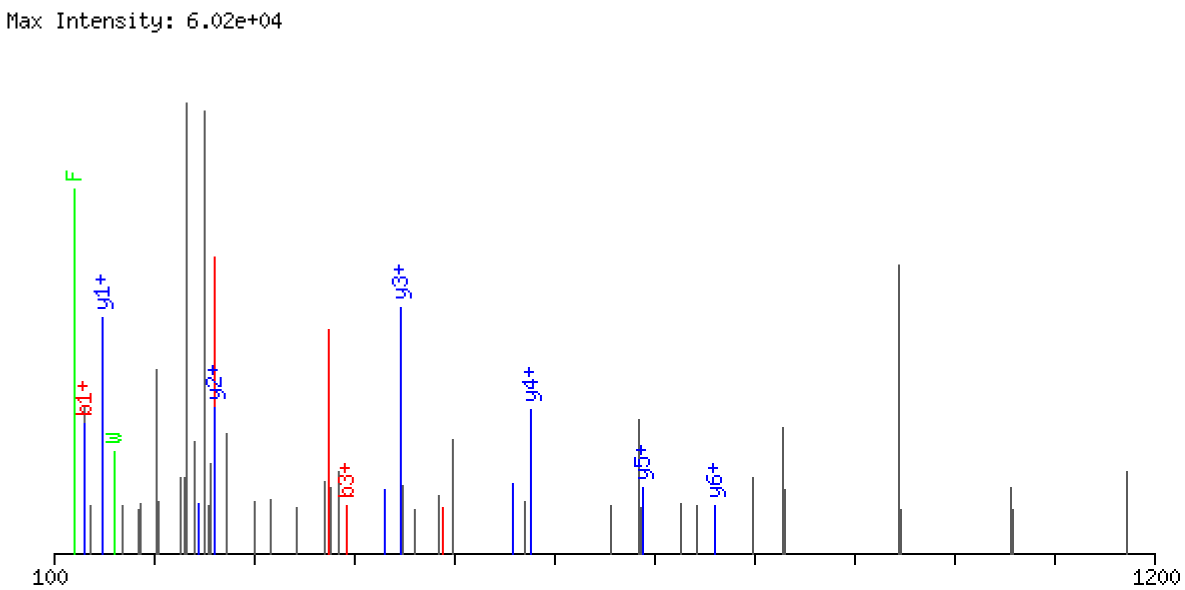

		b+	-H2O	y+	-H2O	-NH3		
E	1	130.05	112.04				12	E
F	2	277.12	259.11	1416.64	1398.63	1399.62	11	F
D	3	392.15	374.13	1269.58	1251.57	1252.55	10	D
L	4	505.23	487.22	1154.55	1136.54	1137.52	9	L
D	5	620.26	602.25	1041.47	1023.45	1024.44	8	D
S	6	787.25	769.24	926.44	908.43	909.41	7	S
A	7	858.29	840.28	759.44	741.43	742.41	6	A
L	8	971.38	953.37	688.40	670.39	671.38	5	L
E	9	1100.42	1082.41	575.32	557.31	558.29	4	E
W	10	1286.50	1268.49	446.28		429.25	3	W
I	11	1399.58	1381.57	260.20		243.17	2	I
K	12			147.11		130.09	1	K

B/Bris M1 78-92 unmodified

FITEPLSGMGTTATK

Charge: 2, Exp. m/z: 777.396, Calc. m/z: 777.402
Data File: D120713_079, Scans: 11005 - 11005

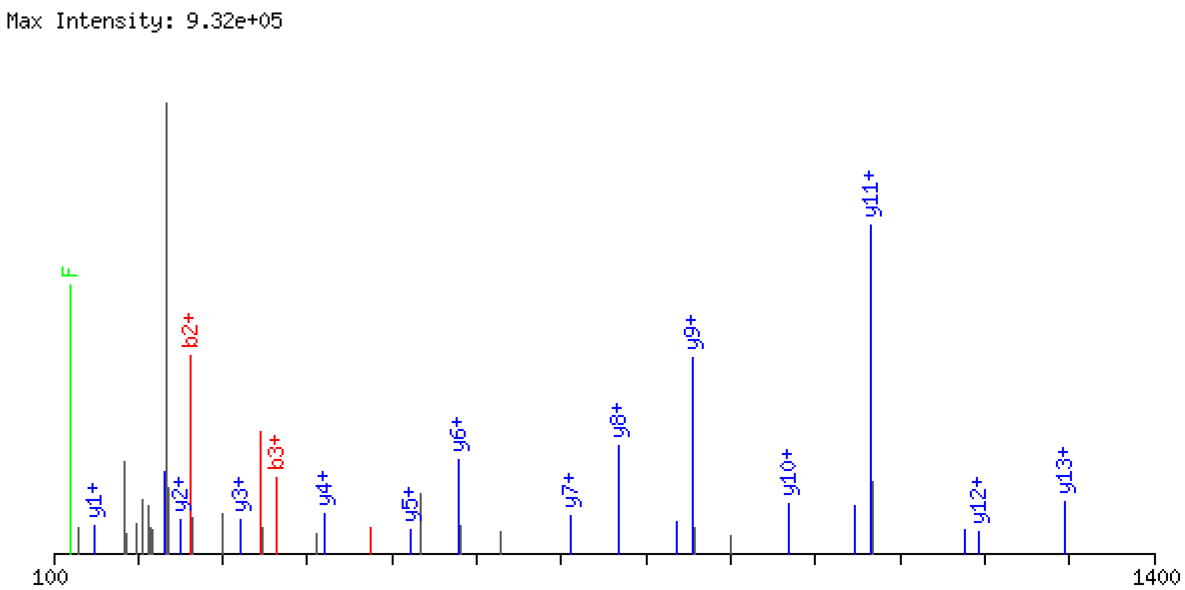

		b+	-H2O	y+	-H2O	-NH3		
F	1	148.08					15	F
I	2	261.16		1406.72	1388.71	1389.69	14	I
T	3	362.21	344.20	1293.64	1275.62	1276.61	13	T
E	4	491.25	473.24	1192.59	1174.58	1175.56	12	E
P	5	588.30	570.29	1063.55	1045.53	1046.52	11	P
L	6	701.39	683.38	966.49	948.48	949.47	10	L
S	7	788.42	770.41	853.41	835.40	836.38	9	S
G	8	845.44	827.43	766.38	748.37	749.35	8	G
M	9	976.48	958.47	709.35	691.34	692.33	7	M
G	10	1033.50	1015.49	578.31	560.30	561.29	6	G
T	11	1134.55	1116.54	521.29	503.28	504.27	5	T
T	12	1235.60	1217.59	420.25	402.23	403.22	4	T
A	13	1306.63	1288.62	319.20	301.19	302.17	3	A
T	14	1407.68	1389.67	248.16	230.15	231.13	2	T
K	15			147.11		130.09	1	K

B/Bris M1 78-92 phospho

FITEPLSGMGT(+79.97)TATK

Charge: 2, Exp. m/z: 817.380, Calc. m/z: 817.382
Data File: D120713_079, Scans: 11424 - 11424

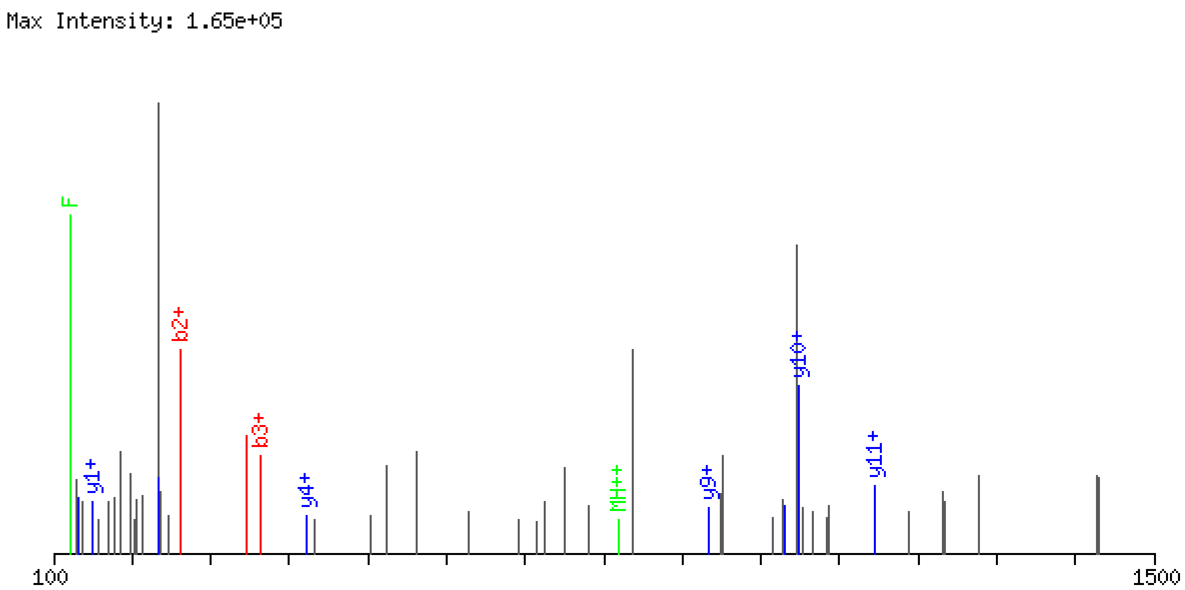

		b+	-H2O	y+	-H2O	-NH3		
F	1	148.08					15	F
I	2	261.16		1486.69	1468.68	1469.66	14	I
T	3	362.21	344.20	1373.60	1355.59	1356.58	13	T
E	4	491.25	473.24	1272.55	1254.54	1255.53	12	E
P	5	588.30	570.29	1143.51	1125.50	1126.48	11	P
L	6	701.39	683.38	1046.46	1028.45	1029.43	10	L
S	7	788.42	770.41	933.37	915.36	916.35	9	S
G	8	845.44	827.43	846.34	828.33	829.32	8	G
M	9	976.48	958.47	789.32	771.31	772.29	7	M
G	10	1033.50	1015.49	658.28	640.27	641.25	6	G
T	11	1214.52	1196.51	601.26	583.25	584.23	5	T
T	12	1315.56	1297.55	420.25	402.23	403.22	4	T
A	13	1386.60	1368.59	319.20	301.19	302.17	3	A
T	14	1487.65	1469.64	248.16	230.15	231.13	2	T
K	15			147.11		130.09	1	K

B/Bris M1 188-213 unmodified

TMNGMGKGEDVQKLAEELQSNIGVLR

Charge: 3, Exp. m/z: 940.150, Calc. m/z: 939.814
Data File: D120713_002, Scans: 14525 - 14525

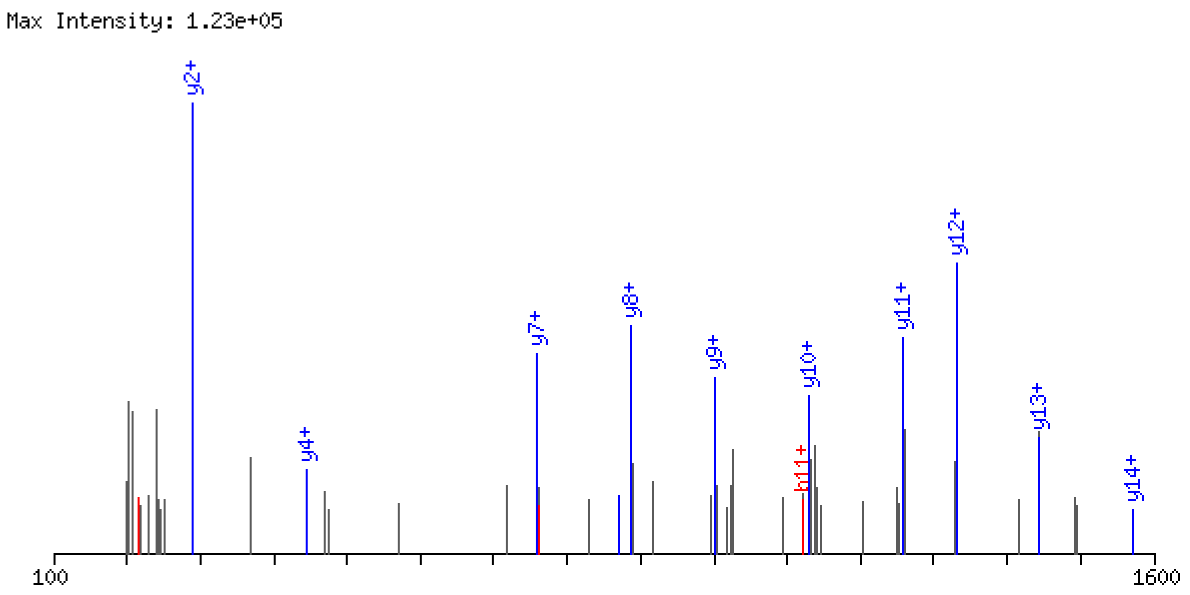

Hiding fragment losses - Click to Show

		b+	b++	y+	y++		
T	1	102.05	51.53			26	T
M	2	233.10	117.05	2716.38	1358.69	25	M
N	3	347.14	174.07	2585.34	1293.17	24	N
G	4	404.16	202.58	2471.29	1236.15	23	G
M	5	535.20	268.10	2414.27	1207.64	22	M
G	6	592.22	296.61	2283.23	1142.12	21	G
K	7	720.32	360.66	2226.21	1113.61	20	K
G	8	777.34	389.17	2098.11	1049.56	19	G
E	9	906.38	453.69	2041.09	1021.05	18	E
D	10	1021.41	511.21	1912.05	956.53	17	D
V	11	1120.48	560.74	1797.02	899.02	16	V
Q	12	1248.53	624.77	1697.95	849.48	15	Q
K	13	1376.63	688.82	1569.90	785.45	14	K
L	14	1489.71	745.36	1441.80	721.40	13	L
A	15	1560.75	780.88	1328.72	664.86	12	A
E	16	1689.79	845.40	1257.68	629.34	11	E
E	17	1818.84	909.92	1128.64	564.82	10	E
L	18	1931.92	966.46	999.59	500.30	9	L
Q	19	2059.98	1030.49	886.51	443.76	8	Q
S	20	2147.01	1074.01	758.45	379.73	7	S
N	21	2261.05	1131.03	671.42	336.21	6	N
I	22	2374.14	1187.57	557.38	279.19	5	I
G	23	2431.16	1216.08	444.29	222.65	4	G

		b+	b++	y+	y++		
V	24	2530.23	1265.62	387.27	194.14	3	V
L	25	2643.31	1322.16	288.20	144.61	2	L
R	26			175.12	88.06	1	R

B/Bris M1 188-213 phospho

T(+79.97)MNGMGK(+114.04)GEDVQKLAEELQSNIGVLR

Charge: 3, Exp. m/z: 1005.160, Calc. m/z: 1004.481
Data File: D120713_003, Scans: 14505 - 14505

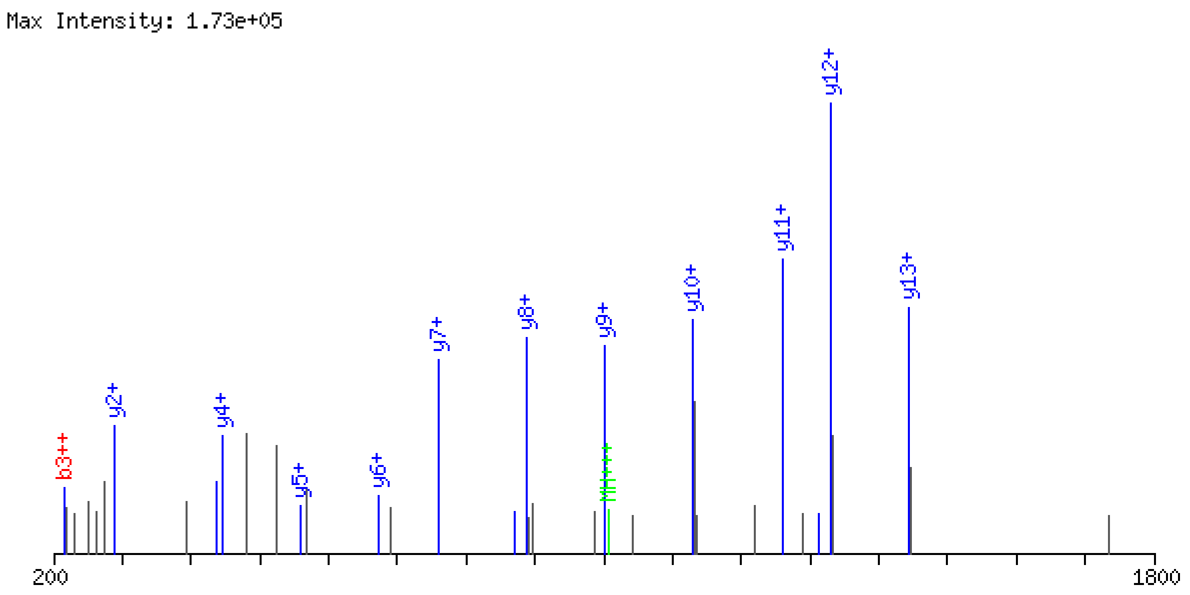

Hiding fragment losses - Click to Show

		b+	b++	y+	y++		
T	1	182.02	91.51			26	T
M	2	313.06	157.03	2830.42	1415.71	25	M
N	3	427.10	214.06	2699.38	1350.19	24	N
G	4	484.13	242.57	2585.34	1293.17	23	G
M	5	615.17	308.09	2528.31	1264.66	22	M
G	6	672.19	336.60	2397.27	1199.14	21	G
K	7	914.33	457.67	2340.25	1170.63	20	K
G	8	971.35	486.18	2098.11	1049.56	19	G
E	9	1100.39	550.70	2041.09	1021.05	18	E
D	10	1215.42	608.21	1912.05	956.53	17	D
V	11	1314.49	657.75	1797.02	899.02	16	V
Q	12	1442.54	721.78	1697.95	849.48	15	Q
K	13	1570.64	785.82	1569.90	785.45	14	K
L	14	1683.72	842.37	1441.80	721.40	13	L
A	15	1754.76	877.88	1328.72	664.86	12	A
E	16	1883.80	942.41	1257.68	629.34	11	E
E	17	2012.85	1006.93	1128.64	564.82	10	E
L	18	2125.93	1063.47	999.59	500.30	9	L
Q	19	2253.99	1127.50	886.51	443.76	8	Q
S	20	2341.02	1171.01	758.45	379.73	7	S
N	21	2455.06	1228.04	671.42	336.21	6	N
I	22	2568.15	1284.58	557.38	279.19	5	I
G	23	2625.17	1313.09	444.29	222.65	4	G

		b+	b++	y+	y++		
V	24	2724.24	1362.62	387.27	194.14	3	V
L	25	2837.32	1419.16	288.20	144.61	2	L
R	26			175.12	88.06	1	R

B/Bris M1 214-227 unmodified

SLGASQKNGEGIAK

Charge: 2, Exp. m/z: 680.365, Calc. m/z: 680.367
Data File: D120713_003, Scans: 7524 - 7524

Max Intensity: 1.83e+05

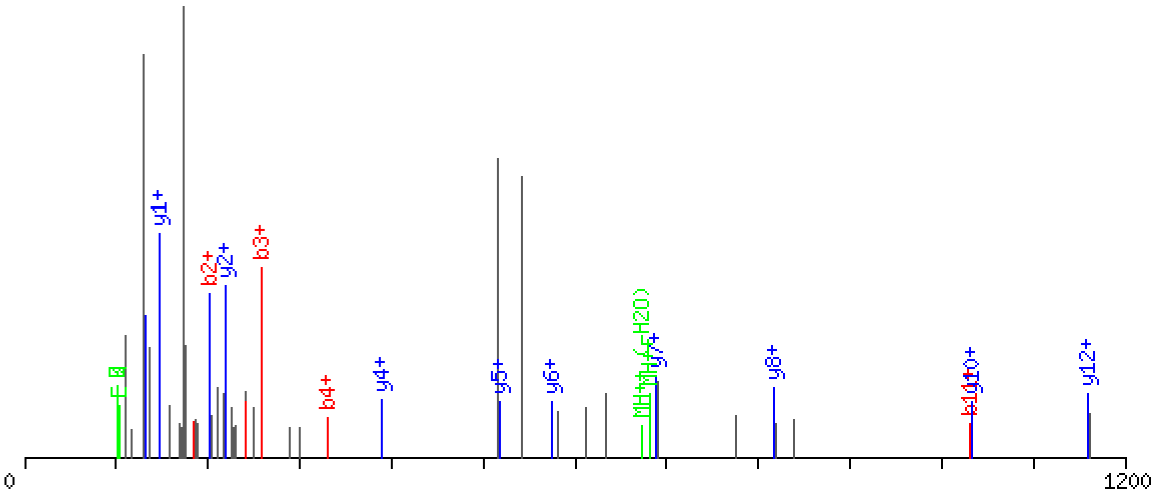

		b+	-H2O	-NH3	y+	-H2O	-NH3		
S	1	88.04	70.03					14	S
L	2	201.12	183.11		1272.69	1254.68	1255.66	13	L
G	3	258.14	240.13		1159.61	1141.60	1142.58	12	G
A	4	329.18	311.17		1102.59	1084.57	1085.56	11	A
S	5	416.21	398.20		1031.55	1013.54	1014.52	10	S
Q	6	544.27	526.26		944.52	926.51	927.49	9	Q
K	7	672.37	654.36	655.34	816.46	798.45	799.43	8	K
N	8	786.41	768.40	769.38	688.36	670.35	671.34	7	N
G	9	843.43	825.42	826.41	574.32	556.31	557.29	6	G
E	10	972.47	954.46	955.45	517.30	499.29	500.27	5	E
G	11	1029.50	1011.49	1012.47	388.26		371.23	4	G
I	12	1142.58	1124.57	1125.55	331.23		314.21	3	I
A	13	1213.62	1195.61	1196.59	218.15		201.12	2	A
K	14				147.11		130.09	1	K

B/Bris M1 214-227 phospho + deamidated

SLGAS(+79.97)QKN(+0.98)GEGIAK

Charge: 2, Exp. m/z: 720.840, Calc. m/z: 720.842
Data File: D120713_003, Scans: 8468 - 8468

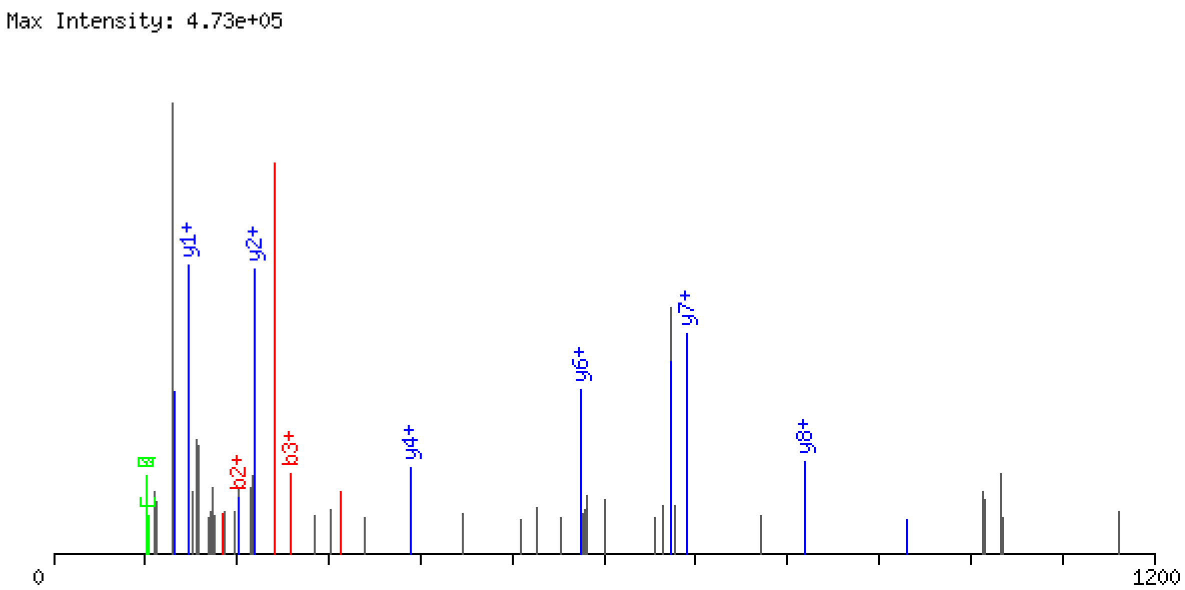

		b+	-H2O	-NH3	y+	-H2O	-NH3		
S	1	88.04	70.03					14	S
L	2	201.12	183.11		1353.64	1335.63	1336.61	13	L
G	3	258.14	240.13		1240.56	1222.55	1223.53	12	G
A	4	329.18	311.17		1183.54	1165.52	1166.51	11	A
S	5	496.18	478.17		1112.50	1094.49	1095.47	10	S
Q	6	624.24	606.23		945.50	927.49	928.47	9	Q
K	7	752.33	734.32	735.31	817.44	799.43	800.41	8	K
N	8	867.36	849.35	850.33	689.35	671.34	672.32	7	N
G	9	924.38	906.37	907.36	574.32	556.31	557.29	6	G
E	10	1053.42	1035.41	1036.40	517.30	499.29	500.27	5	E
G	11	1110.45	1092.44	1093.42	388.26		371.23	4	G
I	12	1223.53	1205.52	1206.50	331.23		314.21	3	I
A	13	1294.57	1276.56	1277.54	218.15		201.12	2	A
K	14				147.11		130.09	1	K

B/Bris M1 235-245 unmodified

QSSMGNSALVK

Charge: 2, Exp. m/z: 561.285, Calc. m/z: 561.287
Data File: D120713_079, Scans: 8296 - 8296

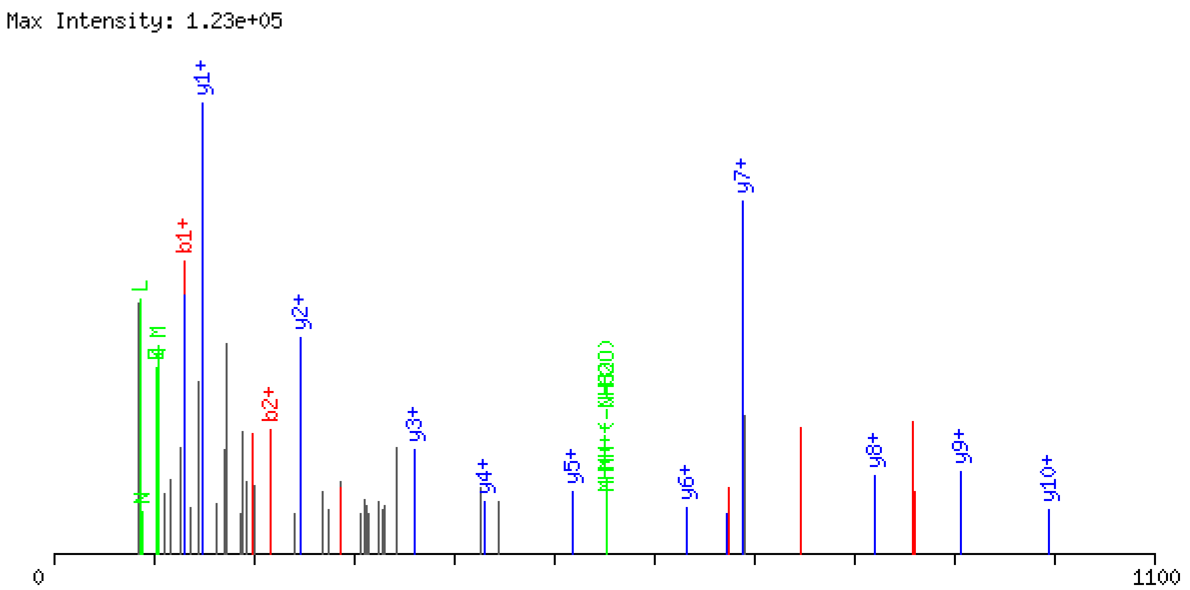

		b+	-H2O	-NH3	y+	-H2O	-NH3		
Q	1	129.07						11	Q
S	2	216.10	198.09		993.50	975.49	976.48	10	S
S	3	303.13	285.12		906.47	888.46	889.44	9	S
M	4	434.17	416.16		819.44	801.43	802.41	8	M
G	5	491.19	473.18		688.40	670.39	671.37	7	G
N	6	605.23	587.22	588.21	631.38	613.37	614.35	6	N
S	7	692.27	674.26	675.24	517.33	499.32	500.31	5	S
A	8	763.30	745.29	746.28	430.30		413.28	4	A
L	9	876.39	858.38	859.36	359.27		342.24	3	L
V	10	975.46	957.45	958.43	246.18		229.15	2	V
K	11				147.11		130.09	1	K

B/Bris M1 235-245 phospho

QSS(+79.97)MGNSALVK

Charge: 2, Exp. m/z: 601.270, Calc. m/z: 601.272
Data File: D120713_079, Scans: 8879 - 8879

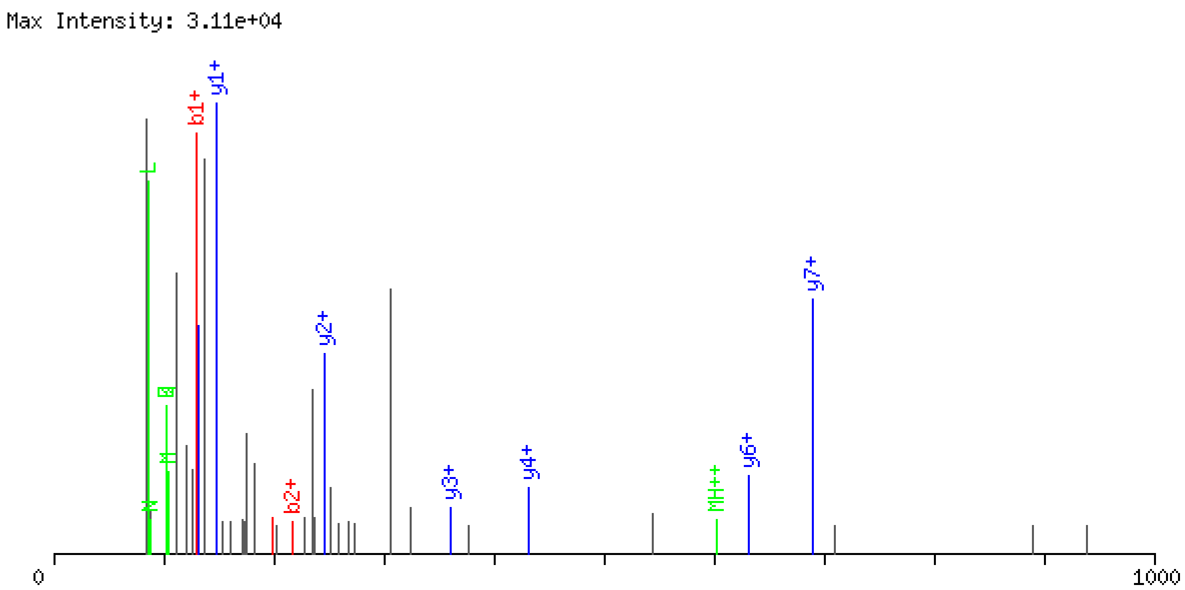

		b+	-H2O	-NH3	y+	-H2O	-NH3		
Q	1	129.07						11	Q
S	2	216.10	198.09		1073.47	1055.46	1056.44	10	S
S	3	383.10	365.09		986.44	968.43	969.41	9	S
M	4	514.14	496.13		819.44	801.43	802.41	8	M
G	5	571.16	553.15		688.40	670.39	671.37	7	G
N	6	685.20	667.19	668.17	631.38	613.37	614.35	6	N
S	7	772.23	754.22	755.21	517.33	499.32	500.31	5	S
A	8	843.27	825.26	826.24	430.30		413.28	4	A
L	9	956.35	938.34	939.33	359.27		342.24	3	L
V	10	1055.42	1037.41	1038.40	246.18		229.15	2	V
K	11				147.11		130.09	1	K
