# Supplementary material for: Mapping the Phosphoproteome of Influenza A and B Viruses by Mass Spectrometry
Source: PLoS Pathog. 2012 Nov 8;8(11):e1002993. doi: 10.1371/journal.ppat.1002993 (PMC3493474; doi:10.1371/journal.ppat.1002993)
Supplement: Table S1 — Protein sequence coverage. (DOC) [file ppat.1002993.s004.doc]

**Table S1: Protein sequence coverage**

| **Virus** | **Protein** | **Peptide spectrum matches** | **Peptides** | **% coverage of protein** | **Average depth of coverage^a^** | **Protein identification probability** |
| --- | --- | --- | --- | --- | --- | --- |
| WSN (without enrichment for phosphopeptides) | | | | |  |  |
|  | PB2 | 276 | 54 | 69.7 | 5.1 | 1.000 |
|  | PB1 | 260 | 45 | 68.4 | 5.8 | 1.000 |
|  | PA | 342 | 57 | 73.2 | 6.0 | 1.000 |
|  | HA | 1116 | 52 | 79.8 | 21.5 | 1.000 |
|  | NP | 1095 | 57 | 88.6 | 19.2 | 1.000 |
|  | NA | 284 | 22 | 64.9 | 12.9 | 1.000 |
|  | M1 | 2395 | 36 | 95.2 | 66.5 | 1.000 |
|  | M2 | 20 | 6 | 55.7 | 3.3 | 1.000 |
|  | NS1 | 48 | 17 | 75.2 | 2.8 | 1.000 |
|  | NEP | 13 | 5 | 60.3 | 2.6 | 1.000 |
| WSN (with enrichment for phosphopeptides) | | | | | | |
|  | PB2 | 109 | 37 | 55.5 | 2.9 | 1.000 |
|  | PB1 | 85 | 32 | 58.9 | 2.7 | 1.000 |
|  | PA | 91 | 37 | 57.3 | 2.5 | 1.000 |
|  | HA | 596 | 45 | 67.3 | 13.2 | 1.000 |
|  | NP | 1242 | 56 | 86.1 | 22.2 | 1.000 |
|  | NA | 156 | 20 | 55.8 | 7.8 | 1.000 |
|  | M1 | 2106 | 31 | 93.7 | 67.9 | 1.000 |
|  | M2 | 24 | 5 | 48.5 | 4.8 | 1.000 |
|  | NS1 | 33 | 13 | 69.6 | 2.5 | 1.000 |
|  | NEP | 23 | 5 | 61.2 | 4.6 | 1.000 |
| X-181 (without enrichment for phosphopeptides) | | | | | | |
|  | PB2 | 50 | 26 | 42.3 | 1.9 | 1.000 |
|  | PB1 | 38 | 17 | 26.2 | 2.2 | 1.000 |
|  | PA | 79 | 29 | 41.5 | 2.7 | 1.000 |
|  | HA | 280 | 34 | 50.4 | 8.2 | 1.000 |
|  | NP | 240 | 37 | 72.9 | 6.5 | 1.000 |
|  | NA | 122 | 15 | 60.8 | 8.1 | 1.000 |
|  | M1 | 418 | 25 | 91.7 | 16.7 | 1.000 |
|  | M2 | 4 | 3 | 41.2 | 1.3 | 1.000 |
|  | NS1 | 26 | 10 | 50.0 | 2.6 | 1.000 |
|  | NEP | 8 | 3 | 36.4 | 2.7 | 1.000 |
| X-187 (without enrichment for phosphopeptides) | | | | | | |
|  | PB2 | 44 | 27 | 39.5 | 1.6 | 1.000 |
|  | PB1 | 28 | 18 | 26.4 | 1.6 | 1.000 |
|  | PA | 54 | 27 | 39.1 | 2.0 | 1.000 |
|  | HA | 246 | 36 | 45.9 | 6.8 | 1.000 |
|  | NP | 247 | 39 | 71.7 | 6.3 | 1.000 |
|  | NA | 50 | 14 | 33.7 | 3.6 | 1.000 |
|  | M1 | 399 | 28 | 84.1 | 14.3 | 1.000 |
|  | M2 | 4 | 2 | 29.9 | 2.0 | 1.000 |
|  | NS1 | 31 | 14 | 63.0 | 2.2 | 1.000 |
|  | NEP | 4 | 2 | 27.3 | 2.0 | 1.000 |
| NIB-74xp (egg-grown; without enrichment for phosphopeptides) | | | | | | |
|  | PB2 | 31 | 17 | 29.8 | 1.8 | 1.000 |
|  | PB1 | 16 | 12 | 20.5 | 1.3 | 1.000 |
|  | PA | 29 | 20 | 32.5 | 1.5 | 1.000 |
|  | HA | 197 | 36 | 46.6 | 5.5 | 1.000 |
|  | NP | 189 | 32 | 69.3 | 5.9 | 1.000 |
|  | NA | 53 | 13 | 52.0 | 4.1 | 1.000 |
|  | M1 | 362 | 24 | 89.7 | 15.1 | 1.000 |
|  | M2 | 5 | 3 | 41.2 | 1.7 | 1.000 |
|  | NS1 | 37 | 12 | 53.9 | 3.1 | 1.000 |
|  | NEP | 1 | 1 | 5.8 | 1.0 | 0.990 |
| NIB-74xp (MDCK-grown; without enrichment for phosphopeptides) | | | | | | |
|  | PB2 | 74 | 34 | 48.2 | 2.2 | 1.000 |
|  | PB1 | 59 | 25 | 38.6 | 2.4 | 1.000 |
|  | PA | 71 | 31 | 48.2 | 2.3 | 1.000 |
|  | HA | 404 | 47 | 60.2 | 8.6 | 1.000 |
|  | NP | 438 | 46 | 81.1 | 9.5 | 1.000 |
|  | NA | 70 | 14 | 45.6 | 5.0 | 1.000 |
|  | M1 | 860 | 30 | 93.3 | 28.7 | 1.000 |
|  | M2 | 7 | 2 | 29.9 | 3.5 | 1.000 |
|  | NS1 | 32 | 11 | 58.7 | 2.9 | 1.000 |
|  | NEP | 8 | 4 | 43.0 | 2.0 | 1.000 |
| B/Brisbane/60/2008 (with and without enrichment for phosphopeptides) | | | | | | |
|  | PB2 | 316 | 47 | 66.1 | 6.7 | 1.000 |
|  | PB1 | 260 | 41 | 70.0 | 6.3 | 1.000 |
|  | PA | 261 | 42 | 59.9 | 6.2 | 1.000 |
|  | HA | 2097 | 42 | 62.4 | 49.9 | 1.000 |
|  | NP | 2153 | 83 | 93.0 | 25.9 | 1.000 |
|  | NA | 208 | 25 | 59.2 | 8.3 | 1.000 |
|  | NB | 23 | 3 | 43.0 | 7.7 | 1.000 |
|  | M1 | 2272 | 46 | 80.2 | 49.4 | 1.000 |
|  | BM2 | 91 | 4 | 48.6 | 22.8 | 1.000 |
|  | NS1 | 93 | 14 | 65.6 | 6.6 | 1.000 |
|  | NEP | 56 | 11 | 84.6 | 5.1 | 1.000 |
| TAP-purified WSN proteins (with enrichment for phosphopeptides) | | | | | | |
|  | PB2 | 187 | 49 | 65.5 | 3.8 | 1.000 |
|  | PB1-TAP | 202 | 47 | 62.9 | 4.3 | 1.000 |
|  | PA | 226 | 54 | 67.3 | 4.2 | 1.000 |
|  | TAP-NP | 542 | 34 | 77.7 | 15.9 | 1.000 |
| Strep-purified WSN proteins (with enrichment for phosphopeptides) | | | | | | |
|  | PB2-Strep | 148 | 56 | 76.7 | 2.6 | 1.000 |
|  | PB1 | 131 | 47 | 61.8 | 2.8 | 1.000 |
|  | PA | 86 | 44 | 62.4 | 2.0 | 1.000 |
|  | HA | 8 | 8 | 12.6 | 1.0 | 1.000 |
|  | NP | 250 | 34 | 64.9 | 7.4 | 1.000 |
|  | M1 | 11 | 6 | 31.3 | 1.8 | 1.000 |
|  | NS1 | 32 | 14 | 69.6 | 2.3 | 1.000 |
| WSN proteins from 293 T cell lysates | | | | | | |
|  | NP | 15 | 13 | 37.6 | 1.2 | 1.000 |
|  | M1 | 15 | 11 | 46.0 | 1.4 | 1.000 |
|  | M2 | 2 | 2 | 37.1 | 1.0 | 1.000 |
|  | NS1 | 32 | 13 | 66.5 | 2.5 | 1.000 |
| WSN proteins from MDBK cell lysates | | | | | | |
|  | M1 | 3 | 2 | 7.1 | 1.5 | 1.000 |
|  | NP | 7 | 6 | 19.3 | 1.2 | 1.000 |
|  | NS1 | 14 | 4 | 20.4 | 3.5 | 1.000 |

NB: Only proteins containing at least one unique peptide (matched to a mass spectrum) are shown.

^a^Mean peptide spectrum matches per peptide.
