# Supplementary material for: Mapping the Phosphoproteome of Influenza A and B Viruses by Mass Spectrometry
Source: PLoS Pathog. 2012 Nov 8;8(11):e1002993. doi: 10.1371/journal.ppat.1002993 (PMC3493474; doi:10.1371/journal.ppat.1002993)
Supplement: Table S2 — Summary of phosphopeptides. (DOC) [file ppat.1002993.s005.doc]

**Table S2: Summary of phosphopeptides**

| **Virus** | **Protein** | **Putative phosphorylation site** | **PSMs with modification** | **Mass Accuracy (ppm)^a^** | **Peptide Identification Probability^a^** | | **PTMScore^a^** | **Next best PTM score^a^** | **Residue Conservation (%)** | **Kinase predictions** |
| --- | --- | --- | --- | --- | --- | --- | --- | --- | --- | --- |
| WSN (without enrichment for phosphopeptides) | | | | | | | | | | |
|  | NP | S402 | 2 | -0.23 | 0.996 | | 82 | 45 (S407) | 99.9 | PKC |
|  |  | S403 |  |  |  | | 82 |  | 0.1 | PKA |
|  | M1 | T9 | 1 | 0.76 | 1 | | 80 | 62 (T5) | 98.5 | N |
|  |  | Y10 |  |  |  | | 80 |  | 98.5 | N |
|  | NEP | S23 | 2 | 1.23 | 1 | | 137 | 35 (T33) | 99.2 | CKII |
|  |  | S24 |  |  |  | | 153 |  | 99.8 | P |
|  |  | S25 |  |  |  | | 137 |  | 99.7 | CKII |
| WSN (with enrichment for phosphopeptides) | | | | | | | | | | |
|  | HA | T358 | 2 | 4.23 | 1.000 | 110 | | 26 (Y365) | 99.6 | N |
|  | NP | S9 | 3 | 0.70 | 1.000 | 142 | | 30 (T15) | 99.7 | PKA,CKI |
|  |  | Y10 |  |  |  | 142 | |  | 99.2 | INSR |
|  |  | S165^b^ | 1 | 2.01 | 0.999 | 101 | | 35 (S170) | 99.98 | PKA |
|  |  | S402 | 6 | 0.78 | 0.999 | 105 | | 71 (S407) | 99.9 | PKC |
|  |  | S403 |  |  |  | 108 | |  | 0.1 | PKA |
|  |  | S457 | 1 | 1.78 | 0.970 | 67 | | 17 (S450) | 99.97 | N |
|  |  | T472 | 2 | 2.15 | 1.000 | 129 | | 104 (S477) | 96.3 | N |
|  |  | S473 |  |  |  | 129 | |  | 2.84 | GSK3 |
|  | NA | S160^b^ | 1 | 3.07 | 0.991 | 40 | | 27 (T176) | 99.8 | RSK,PKA |
|  |  | S164^b^ |  |  |  | 37 | |  | 99.98 | N |
|  |  | S166^b^ |  |  |  | 38 | |  | 99.8 | N |
|  | M1 | S2 | 2^c^ | 0.44 | 0.968 | 152 | | 89 (T9) | 99.97 | PKA |
|  |  | T5 |  |  |  | 148 | |  | 99.91 | N |
|  |  | T9 | 1 | 0.76 | 1.000 | 133 | | 131 (T5) | 98.5 | N |
|  |  | Y10 |  |  |  | 146 | |  | 98.5 | N |
|  |  | T108 | 1 | 1.73 | 0.978 | Unambiguous | |  | 99.97 | CKI |
|  |  | T168 | 1 | 0.74 | 0.981 | 81 | | 66 (T167) | 93.1 | P |
|  |  | T169 |  |  |  | 81 | |  | 99.9 | N |
|  |  | S195 | 7 | 1.69 | 1.000 | 131 | | 18 (S207) | 99.9 | CKI |
|  |  | S196 |  |  |  | 140 | |  | 99.8 | N |
|  |  | S224 | 1 | 0.79 | 1.000 | 111 | | 97 (T218) | 92.3 | N |
|  |  | S225 |  |  |  | 111 | |  | 98.7 | Cdc2 |
|  |  | S226 |  |  |  | 121 | |  | 99.97 | P |
|  | M2 | S64 | 42 | 2.04 | 0.998 | 125^d^ | | 22 (S71) | 98.4 | PKA |
|  |  | T65 |  |  |  | 97 | |  | 99.4 | P |
|  |  | S64 and T65 | 1 | 0.83 | 0.995 | 85 | | 34 (S64 and S71) | |  |
|  | NS1 | T197 | 1 | 1.97 | 0.996 | 59 | | 25 (S195) | 37.8 | N |
|  |  | T215^e^ | 1 | 1.35 | 0.604 | 61 | | 15 (S205) | 27.1 | p38MAPK, GSK3, Cdk5 |
|  | NEP | S23^e^ | 9 | 1.34 | 1.000 | 123 | | 35 (T33) | 99.2 | CKII |
|  |  | S24^e^ |  |  |  | 151 | |  | 99.8 | P |
|  |  | S25^e^ |  |  |  | 137 | |  | 99.8 | CKII |
| Egg-grown influenza A CVVs (without enrichment for phosphopeptides) | | | | | | | | | | |
|  | NP (NIB74xp, X-181 and X-187) | S402 | 3 | -0.79 | 1.000 | | 95 | 55 (S407) | 99.9 | PKC |
|  | M1 (X-181) | S2 | 1 | -4.79 | 1.000 | | 63 | 24 (S13) | 99.97 | PKA |
|  |  | T5 |  |  |  | | 66 |  | 99.91 | CKII |
|  |  | T9 |  |  |  | | 56 |  | 99.97 | N |
|  |  | Y10 |  |  |  | | 56 |  | 98.5 | N |
|  | M1 (NIB74xp) | S224 | 1 | 0.71 | 0.976 | | 80 | 40 (T221) | 92.3 | N |
|  |  | S225 |  |  |  | | 80 |  | 98.7 | Cdc2 |
|  |  | S226 |  |  |  | | 80 |  | 99.97 | N |
|  | M2 (NIB74xp) | S64 | 1 | -0.99 | 0.982 | | 23 | None | 98.4 | P |
|  |  | T65 |  |  |  | | 30 |  | 99.4 | PKC |
| NIB-74xp (MDCK-grown; without enrichment for phosphopeptides) | | | | | | | | | | |
|  | NP | S402 | 2 | -1.10 | 1.000 | | 118 | 72 (S407) | 99.9 | PKC |
|  | M1 | S2 | 1 | -4.79 | 1.000 | | 76 | 39 (S13) | 99.97 | PKA |
|  |  | T5 |  |  |  | | 79 |  | 99.91 | CKII |
|  |  | T9 |  |  |  | | 72 |  | 99.95 | N |
|  |  | Y10 |  |  |  | | 64 |  | 99.97 | P |
|  |  | T37 | 2 | 3.72 | 1.000 | | Unambiguous | None | 98.6 | PKG,CKII |
|  | M2 | S64 | 2 | 0.2 | 0.971 | | 71 | None | 98.4 | P |
|  |  | T65 |  |  |  | | 62 |  | 99.4 | PKC |
| B/Brisbane/60/2008 (without enrichment for phosphopeptides) | | | | | | | | | | |
|  | NP | S50 | 8 | -0.91 | 0.999 | | 123 | 77 (T46) | 100 | GSK3, Cdk5 |
|  | M1 | S2 | 1 | -3.86 | 0.983 | | 50 | 36 (S13) | 100 | CKI, PKA |
|  |  | T7 |  |  |  | | 52 |  | 100 | N |
|  |  | Y10 |  |  |  | | 44 |  | 100 | N |
| B/Brisbane/60/2008 (with enrichment for phosphopeptides) | | | | | | | | | | |
|  | HA | S135 | 1 | 7.04 | 0.913 | | 108 | 10 (Y150) | 100 | PKA, PKC |
|  |  | T136 |  |  |  | | 109 |  | 98.2 | PKC |
|  |  | S465 | 6 | 3.08 | 1.000 | | 117 | 93 (S472) | 100 | ATM, CKI |
|  | NP | S50 | 11 | -0.51 | 1.000 | | 124 | 87 (T55) | 100 | Cdk5, GSK3 |
|  |  | T55 | 5 | -0.49 | 0.999 | | 65 | None | 99.6 | Cdc2 |
|  |  | T56 |  |  |  | | 65 |  | 99.4 | CKII, PKG |
|  |  | S57 |  |  |  | | 65 |  | 79.7 | P |
|  |  | S58 |  |  |  | | 50 |  | 88.8 | CKI, CKII |
|  |  | S223 | 1 | -1.03 | 1.000 | | 170 | 109 (S226) | 100 | N |
|  |  | Y352 | 1 | 1.32 | 0.926 | | 118 | 57 (T373) | 100 | P |
|  |  | S353^f^ |  |  |  | | 118 |  | 100 | Cdc2, CKII |
|  |  | Y357 |  |  |  | | 118 |  | 100 | P |
|  |  | Y363 |  |  |  | | 117 |  | 100 | N |
|  |  | S459^b^ | 2 | -0.63 | 1.000 | | 129 | 110 (S448) | 100 | N |
|  |  | S463^b^ |  |  |  | | 129 |  | 99.6 | N |
|  |  | S465^b^ |  |  |  | | 129 |  | 99.8 | Cdc2, Cdk5, p38MAPK |
|  |  | S486 | 9 | -0.53 | 1.000 | | 80 | None | 98.9 | PKA, RSK |
|  | M1 | S2 | 5 | -2.83 | 0.997 | | 117 | T5 (S13) | 100 | CKI, PKA |
|  |  | T7 |  |  |  | | 108 |  | 100 | N |
|  |  | Y10 |  |  |  | | 109 |  | 100 | N |
|  |  | S41 | 1 | 3.50 | 0.922 | | 49 | None | 100 | CKI, CKII |
|  |  | S84 | 1 | -0.31 | 0.862 | | 57 | 15 (T80) | 100 | Cdc2 |
|  |  | T88 |  |  |  | | 57 |  | 100 | PKC |
|  |  | T89 |  |  |  | | 44 |  | 100 | Cdc2, PKC |
|  |  | T91 |  |  |  | | 44 |  | 100 | PKC |
|  |  | T188^g^ | 2 | 5.60 | 0.999 | | 153 | 20 (S207) | 99.8 | PKC |
|  |  | S214^e^ | 4 | 0.35 | 0.996 | | 94 | None | 100 | PKC |
|  |  | S218^e^ |  |  |  | | 98 |  | 99.8 | ATM |
|  |  | S236 | 1 | 3.75 | 0.898 | | 104 | 68 (S241) | 100 | Cdc2 |
|  |  | S237 |  |  |  | | 106 |  | 100 | PKA |
| TAP-purified WSN proteins (with enrichment for phosphopeptides) | | | | | | | | | | |
|  | PB1-TAP | T223^h^ | 1 | -0.92 | 0.985 | | 34 | 12 (T228) | 99.8 | N |
|  | TAP-NP | S402 | 1 | -0.55 | 0.998 | | 98 | 67 (S407) | 99.9 | PKC |
|  |  | S403 |  |  |  | | 98 |  | 0.1 | PKA |
| Strep-purified WSN proteins (with enrichment for phosphopeptides) | | | | | | | | | | |
|  | PB2-CStrep | S741 | 1 | -2.11 | 1.000 | | 73 | S77 (40) | 99.8 | PKA |
|  |  | S742 |  |  |  | | 96 |  | 99.9 | PKA, PKB, RSK |
|  | PA | S224^e^ | 1 | -2.48 | 0.998 | | 49 | 15 (S218) | 99.5 | N |
|  |  | S225^e^ |  |  |  | | 62 |  | 69.2 | N |
|  | NP | S9 | 3 | -0.28 | 1.000 | | 77 | 16 (T15) | 99.7 | PKA,CKI |
|  |  | Y10 |  |  |  | | 77 |  | 99.2 | INSR |
|  |  | S165 | 1 | -3.43 | 1.000 | | 119 | 80 (T171) | 99.98 | PKA |
|  |  | Y296 | 3 | 0.70 | 1.000 | | 47 | None | 99.9 | P |
|  |  | S297 |  |  |  | | 52 |  | 99.98 | Cdc2 |
|  |  | S376 | 1 | 7.86 | 0.798 | | 193 | 170 (T373) | 99.9 | CKII |
|  |  | S377 |  |  |  | | 218 |  | 58.6 | CKII |
|  |  | T378 |  |  |  | | 218 |  | 99.95 | N |
|  |  | S402 | 2 | 1.71 | 1.000 | | 145 | 95 (S407) | 99.9 | PKC |
|  |  | S403 |  |  |  | | 142 |  | 0.1 | PKA |
| WSN proteins from 293T cell lysates | | | | | | | | | | |
|  | NS1 | S28 | 3 | 4.96 | 1.000 | | 132 | 10 (T36) | 45.1 | PKA |
|  |  | T49 |  |  |  | | 116 |  | 99.9 | PKB |
|  |  | T215 | 4 | 6.23 | 1.000 | | 57 | 11 (S205/S206) | 27.1 | Cdk5, GSK3, p38MAPK |
| WSN proteins from MDBK cell lysates | | | | | | | | | | |
|  | NS1 | T215 | 5 | -0.47 | 1.000 | | 49 | 8 (S205/S206) | 27.1 | Cdk5, GSK3, p38MAPK |

P = phosphorylation predicted by NetPhos 2.0, with no kinase predicted by NetPhosK 1.0

N = no phosphorylation predicted

^a^of clearest peptide spectral match

^b^with artefactual carbamidomethylation

^c^co-eluted near to an isobaric peptide

^d^in other high-scoring spectra S64 and T65 are ambiguous

^e^with artefactual deamidation

^f^No 98 Da neutral loss observed, so manually assigned as phosphotyrosine

^g^with ubiquitination of K194 or K200 (residue conservation 100% or 99.1% respectively)

^h^with artefactual oxidation of M227
