# Supplementary material for: Mapping the Phosphoproteome of Influenza A and B Viruses by Mass Spectrometry
Source: PLoS Pathog. 2012 Nov 8;8(11):e1002993. doi: 10.1371/journal.ppat.1002993 (PMC3493474; doi:10.1371/journal.ppat.1002993)
Supplement: Table S3 — N-terminal modifications. (DOC) [file ppat.1002993.s006.doc]

**Table S3: N-terminal modifications**

|  | **PB1** | **PA** | **NP** | **M1** | **M2** | **NS1** | **NEP** |
| --- | --- | --- | --- | --- | --- | --- | --- |
| **WSN (virus)** | N |  |  | X, M, N, MN | M, MN | N | N |
| **WSN (TAP-purified protein)** | N | N |  |  |  |  |  |
| **X-181** | N |  |  | M, N, MN | M, MN |  |  |
| **X-187** | N |  |  | M, MN | MN |  |  |
| **NIB-74xp (egg-grown)** | N |  |  | M, MN | M, MN |  | M, N |
| **NIB-74xp (MDCK-grown)** | N |  | MN | M, MN | MN |  |  |
| **B/Brisbane/60/2008** | | N | MN | X, M, N, MN |  | MN | MN |

X: No modification

M: Methionine excision

N: N-terminal acetylation

MN: Methionine excision and N-terminal acetylation
