# Supplementary material for: Mapping the Phosphoproteome of Influenza A and B Viruses by Mass Spectrometry
Source: PLoS Pathog. 2012 Nov 8;8(11):e1002993. doi: 10.1371/journal.ppat.1002993 (PMC3493474; doi:10.1371/journal.ppat.1002993)
Supplement: Table S4 — Number of sequences used in alignments. (DOC) [file ppat.1002993.s007.doc]

**Table S4: Number of sequences used in alignments**

| Influenza A | PB2 | 14732 |
| --- | --- | --- |
|  | PB1 | 13470 |
|  | PA | 15590 |
|  | HA - H1 | 13640 |
|  | HA - H2 | 382 |
|  | HA - H3 | 5500 |
|  | HA - H4 | 430 |
|  | HA - H5 | 2724 |
|  | HA - H6 | 932 |
|  | HA - H7 | 1021 |
|  | HA - H8 | 57 |
|  | HA - H9 | 834 |
|  | HA - H10 | 166 |
|  | HA - H11 | 165 |
|  | HA - H12 | 67 |
|  | HA - H13 | 47 |
|  | HA - H14 | 6 |
|  | HA - H15 | 12 |
|  | HA - H16 | 22 |
|  | HA - H17 | 3 |
|  | NP | 14660 |
|  | NA - N1 | 4942 |
|  | NA - N2 | 10465 |
|  | NA - N3 | 492 |
|  | NA - N4 | 106 |
|  | NA - N5 | 129 |
|  | NA - N6 | 670 |
|  | NA - N7 | 446 |
|  | NA - N8 | 708 |
|  | NA - N9 | 199 |
|  | M1 | 20120 |
|  | M2 | 20390 |
|  | NS1 | 16040 |
|  | NEP | 16040 |
| Influenza B | PB2 | 623 |
|  | PB1 | 614 |
|  | PA | 658 |
|  | HA (Victoria) | 1843 |
|  | HA (Yamagata) | 1052 |
|  | NP | 526 |
|  | NA (Victoria) | 534 |
|  | NA (Yamagata) | 321 |
|  | NB (Victoria) | 536 |
|  | NB (Yamagata) | 326 |
|  | M1 | 549 |
|  | M2 | 551 |
|  | NEP | 1426 |
| Influenza C | PB2 | 6 |
|  | PB1 | 4 |
|  | P3 | 3 |

Sequences were downloaded from GISAID or the NCBI influenza virus resource between 06/03/2012 and 26/07/2012.
